# Probing Regio- and Enantioselectivity in the Formal [2 + 2] Cycloaddition of C(1)-Alkyl Ammonium Enolates with β- and α,β-Substituted Trifluoromethylenones

**DOI:** 10.1021/acs.joc.2c02688

**Published:** 2023-05-15

**Authors:** Yihong Wang, Claire M. Young, David B. Cordes, Alexandra M. Z. Slawin, Andrew D. Smith

**Affiliations:** EaStCHEM, School of Chemistry, University of St Andrews, North Haugh, St Andrews, Fife, KY16 9ST, U.K.

## Abstract

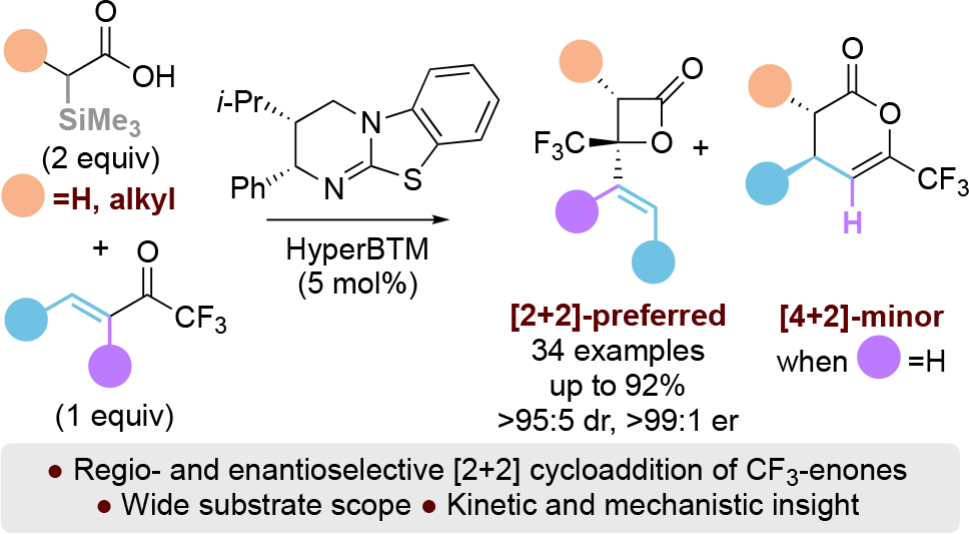

The isothiourea-catalyzed regio- and enantioselective formal [2 + 2] cycloaddition of C(1)-alkyl and C(1)-unsubstituted ammonium enolates with β- and α,β-substituted trifluoromethylenones has been developed. In all cases, preferential [2 + 2]-cycloaddition over the alternative [4 + 2]-cycloaddition is observed, giving β-lactones with excellent diastereo- and enantioselectivity (34 examples, up to >95:5 dr, >99:1 er). The regioselectivity of the process was dictated by the nature of the substituents on both reaction components. Solely [2 + 2] cycloaddition products are observed when using α,β-substituted trifluoromethylenones or α-trialkylsilyl acetic acid derivatives; both [2 + 2] and [4 + 2] cycloaddition products are observed when using β-substituted trifluoromethylenones and α-alkyl-α-trialkylsilyl acetic acids as reactants, with the [2 + 2] cycloaddition as the major reaction product. The beneficial role of the α-silyl substituent within the acid component in this protocol has been demonstrated by control experiments.

## Introduction

**Scheme 1:**
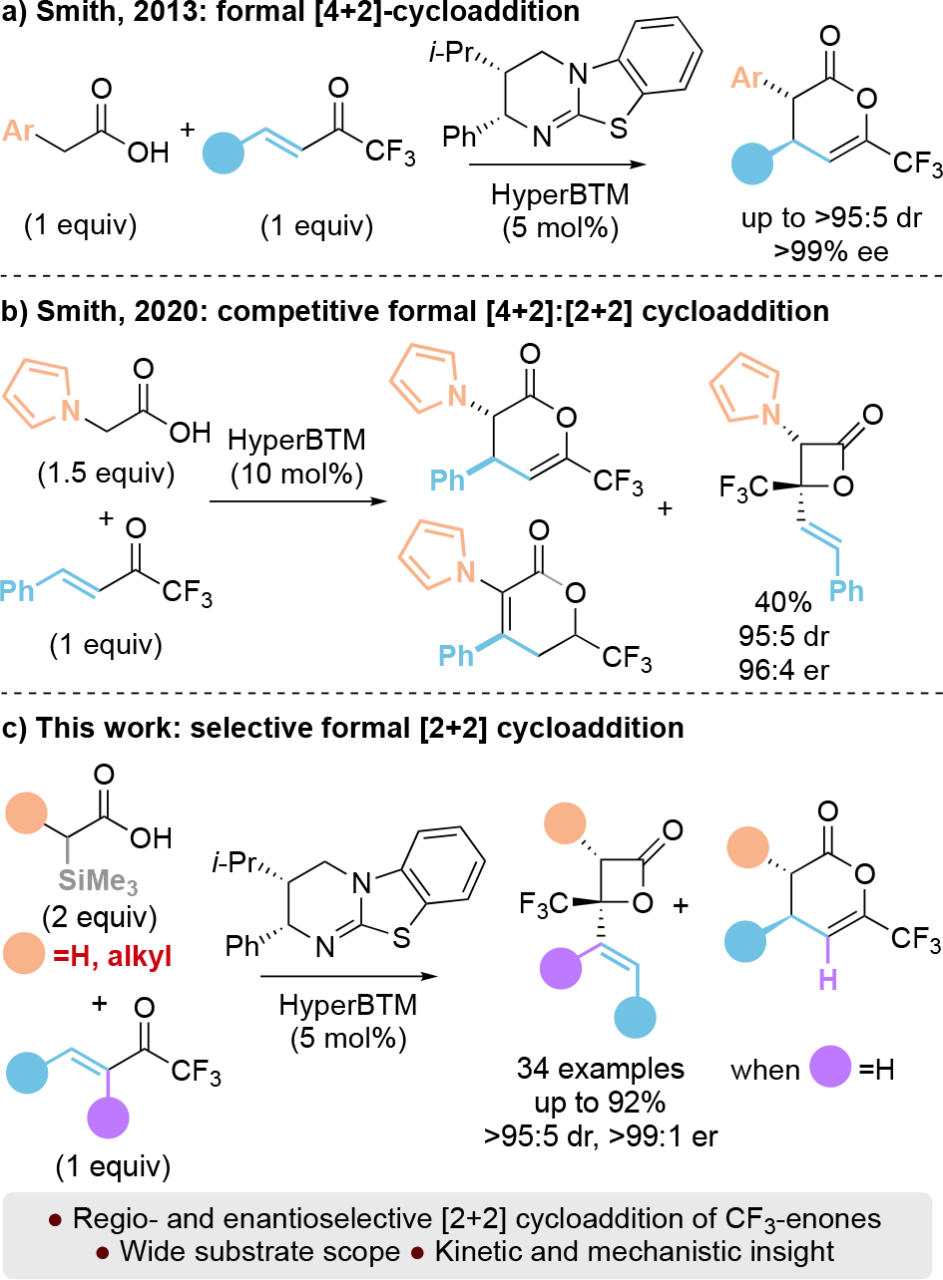
Isothiourea-Catalyzed Enantioselective Cycloadditions with Trifluoromethylenones

The asymmetric synthesis of β-lactones has attracted considerable interest in organic chemistry due to their versatility as synthetic intermediates as well as their prevalence in a wide range of biologically active molecules.^1^ Enantioenriched β-lactones can be accessed in a number of ways, with Lewis acid- or Lewis base-catalyzed formal cycloadditions being the most common.^2,3^ Lewis base-catalyzed approaches typically proceed through the formal [2 + 2] cycloaddition of ammonium enolates with ketenes, aldehydes, or highly reactive ketones.^4−8^ In related Lewis base-catalyzed processes, trifluoromethylenones have been extensively explored as electrophiles in formal [4 + 2] cycloadditions such as the isothiourea-catalyzed reaction of trifluoromethylenones with arylacetic acid derivatives (Scheme 1a).^9,10^ In this case, exclusive formation of [4 + 2]-products was observed, giving C(6)-trifluoromethyldihydropyranones in high yields and excellent enantioselectivity. However, use of 2-(pyrrol-1-yl)acetic acid in this protocol notably gave a 50:50 ratio of products arising from formal [4 + 2] and [2 + 2] cycloaddition reactions (Scheme 1b),^11^ indicating that regioselective reaction directly with the carbonyl of the α,β-unsaturated system to generate the corresponding β-lactone is feasible and is dependent upon the C(1)-substitution of the ammonium enolate. Intrigued by this observation, in this manuscript we report the regio- and enantioselective addition of a range of C(1)-alkyl substituted or unsubstituted ammonium enolates, prepared through a recently reported desilylation process,^12^ to trifluoromethylenones.

Systematic variation of the substituents within both the trifluoromethylenone and the C(1)-alkyl substituted or unsubstituted ammonium enolate provide preferential, and in some cases exclusive, access to highly functionalized β-lactones with high enantioselectivity.

## Results and Discussion

### Investigation of Optimal Reaction Conditions

An initial trial was performed using α-trimethylsilyl acetic acid **1** as a C(1)-ammonium enolate precursor with β-phenyl trifluoromethylenone **2** (Table 1). Treatment of acid **1** with pivaloyl chloride (3 equiv) in MTBE to generate the corresponding mixed anhydride, followed by addition of (2*S*,3*R*)-HyperBTM **4** (5 mol %) and enone **2** at room temperature, gave exclusively the formal [2 + 2]-cycloaddition product, β-lactone **3**, in high yield (75%) and excellent enantioselectivity (92:8 er). Attempted optimization varied a range of reaction parameters, including solvent, catalyst, temperature, auxiliary base, and acid chloride. A range of polar and nonpolar solvents were tested, but in all cases led to reduced yield and enantioselectivity compared with MTBE (see SI). Using (*R*)-BTM **5** gave significantly reduced conversion to the product, giving 12% isolated yield of **3** in 75:25 er (entry 2), while (*S*)-tetramisole **6** gave no conversion to the product (entry 3). Further variation of base showed that using triethylamine instead of *N*,*N*-diisopropylethylamine did not affect the enantioselectivity, but gave significantly decreased yield (entry 4), while inorganic bases Cs_2_CO_3_ and NaHCO_3_ led to poor reactivity (entries 5–6). Using benzoyl chloride and *para*-nitrobenzoyl chloride to generate the corresponding mixed anhydride resulted in reduced product yields and enantioselectivity (entries 7–8). The use of 1 equiv of acid **1** led to reduced product conversion (entry 9) while reducing the temperature to 0 °C gave the product with slightly reduced er (entry 10).

**Table 1:**
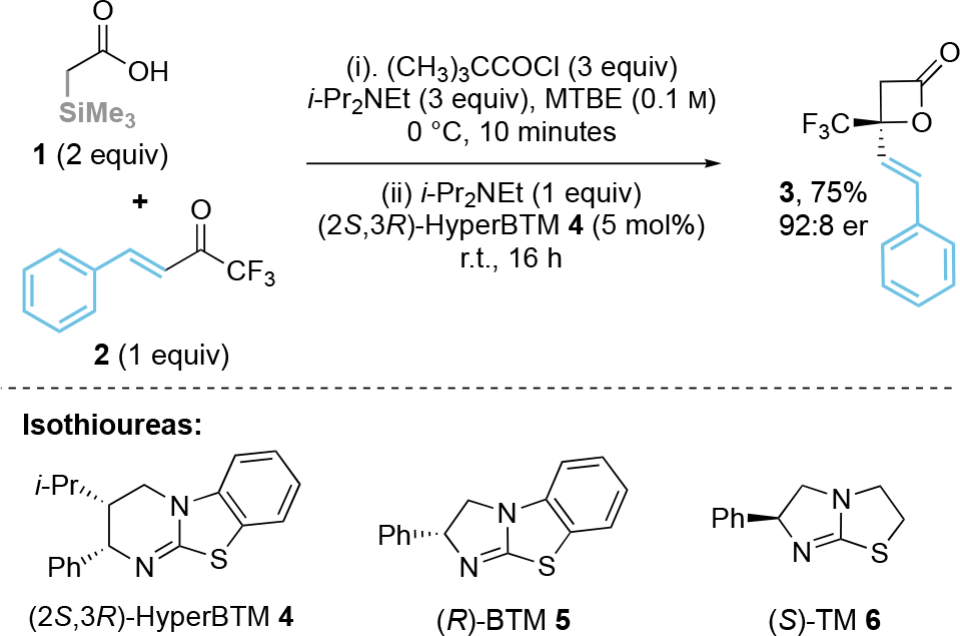
Optimization of Reaction Conditions^a^

entry	variant	yield^b^ (%)	er^c^
1	none	75	92:8
2	(*R*)-BTM **5** as catalyst	12	75:25
3	(*S*)-TM **6** as catalyst	0	–
4	Et_3_N as base	45	92:8
5	NaHCO_3_ as base	30	–
6	Cs_2_CO_3_ as base	30	–
7	PhC(O)Cl	57	89:11
8	4-NO_2_C_6_H_4_C(O)Cl	48	89:11
9	**1** (1 equiv)	56^d^	–
10	0 °C for (ii)	80	89:11

a (CH_3_)_3_CC(O)Cl (1.2 mmol), *i*-Pr_2_NEt (1.2 mmol) and acid **1** (0.8 mmol) in MTBE (4 mL, 0.1 M) was stirred at 0 °C for 10 min before addition of *i*-Pr_2_NEt (0.4 mmol), enone **2** (0.4 mmol), and (2*S*,3*R*)-HyperBTM **4** (5 mol %) at r.t. for 16 h. MTBE = methyl *tert*-butyl ether. r.t. = room temperature (18 °C). BTM = benzotetramisole. TM = tetramisole.

b Isolated yield.

c Determined by HPLC analysis on a chiral stationary phase.

d Determined by ^1^H NMR analysis of the crude reaction mixture.

### Scope, Limitations, and Derivatizations

With the optimal conditions in hand, the substrate scope of this process was explored, initially using **1** as the ammonium enolate precursor, with the effect of variation in product distribution between [2 + 2]- and [4 + 2]-cycloaddition considered (Table 2). In all cases, β-substituted trifluoromethylenones gave exclusive [2 + 2]-cycloaddition, giving C(3)-unsubstituted β-lactones **7**–**13**. Products **7**–**9**, bearing a *para*-Br, *ortho*-Br, and *meta*-OMe substituents on the β-aryl group, respectively, were obtained in good yields (65% to 78%) and enantioselectivities (89:11 er to 92:8 er). α,β-Disubstituted trifluoromethylenones gave access to β-lactones **10**–**13** with overall good yield (52% to 92%) and high levels of enantioselectivity (92:8 to 94:6 er). However, no reactivity was observed when employing α-benzyl-β-phenyl substituted trifluoromethylenone **14a** or β-Me substituted enone **14b** as the electrophile with only unreacted enones returned (Table 2).

**Table 2:**
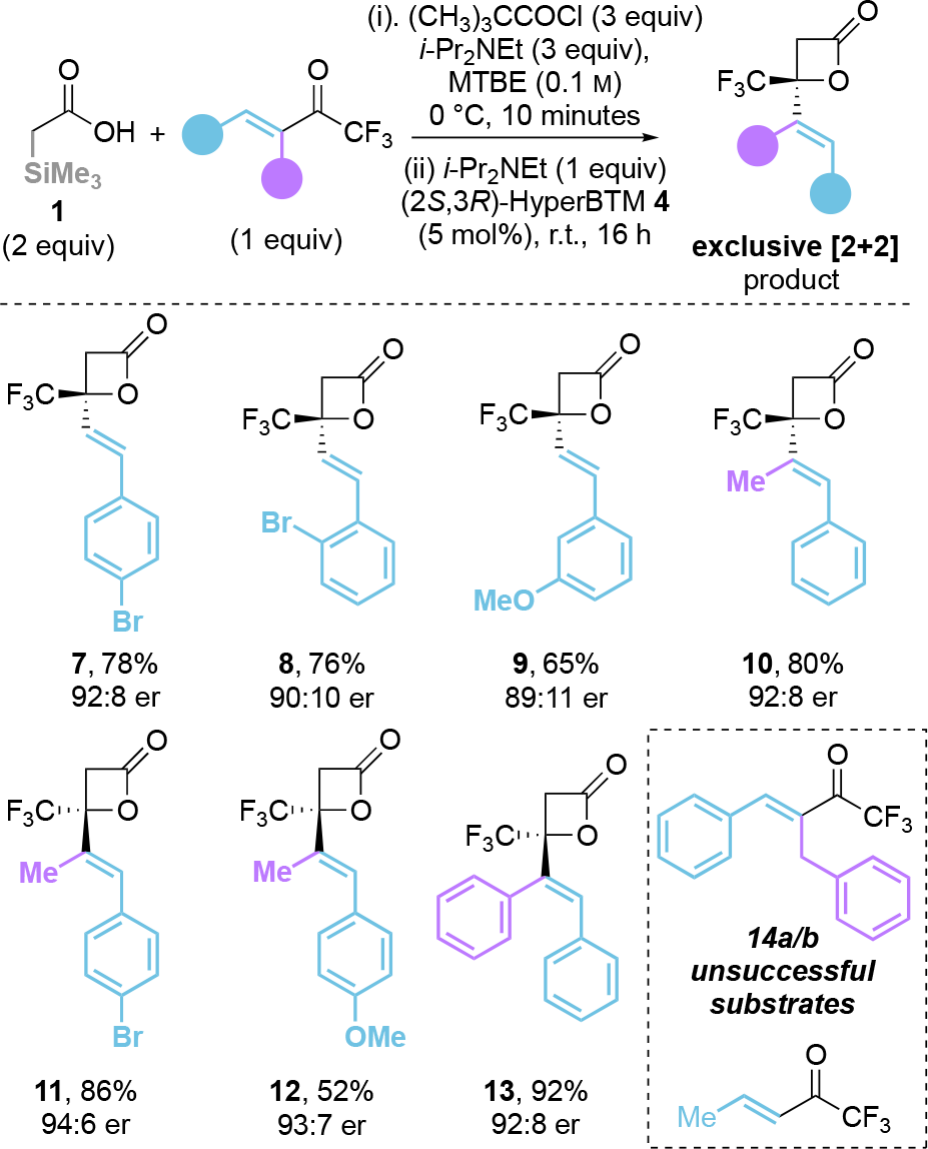
Scope and Limitations: α-Trimethylsilyl Acid **1** and Trifluoromethylenone Variation^a^

a (CH_3_)_3_CC(O)Cl (1.2 mmol), *i*-Pr_2_NEt (1.2 mmol), and acid **1** (0.8 mmol) in MTBE (4 mL, 0.1 M) was stirred at 0 °C for 10 min before addition of *i*-Pr_2_NEt (0.4 mmol), enone **2** (0.4 mmol), and (2*S*,3*R*)-HyperBTM **4** (5 mol %) at r.t. for 16 h. All reported isolated yields all product er’s determined by HPLC analysis on a chiral stationary phase.

Further substrate exploration extended this protocol to the variation of α-silyl-α-alkyl substituted acids and β-substituted trifluoromethylenones (Table 3A). Interestingly, the incorporation of an α-alkyl substituent into the α-silyl acid component led to the formation of 5–20% of the [4 + 2]-cycloaddition product in this series, although the [2 + 2]-cycloaddition was still favored. For example, using α-methyl-α-trimethylsilyl substituted acid as the anhydride precursor and β-phenyltrifluoromethylenone as reactant gave **15** in 75% yield (85:15 dr) in excellent enantioselectivity (99:1 er), and 85:15 ratio of ([2 + 2]:[4 + 2] products). Variation of the β-substituent within the enone through the incorporation of *para*-Br, *para*-Cl, *para*-F, and *para*-CF_3_ substituents gave **16**–**19** with excellent diastereo- and enantioselectivity (up to 90:10 dr, >99:1 er) in good yield (59% to 76%), and with preferential [2 + 2]-cycloaddition ([2 + 2]:[4 + 2] = 85:15 to >95:5). The absolute configurations of products **17** and **18** were confirmed by X-ray crystallographic analysis, with all other products assigned by analogy. *meta*-Substitution within the enone gave β-lactones **20**–**22** in diminished but acceptable yields (51% to 64%), with good diastereoselectivity and exceptional enantiocontrol (85:15 dr, up to >99:1 er), and preferential [2 + 2]-regioselectivity ([2 + 2]:[4 + 2] = 82:18 to 94:6). Incorporation of a β-1- or 2-naphthyl substituent within the trifluoromethylenone gave products **23** and **24** in moderate yield (54% and 64%) but excellent diastereo- and enantioselectivity (both 90:10 dr, >99:1 er), with 1-naphthyl substituted [2 + 2]-product **24** obtained exclusively. The use of trifluoromethylenones bearing *ortho*-substituted-β-aryl substituents also led to improved selectivity for [2 + 2] over [4 + 2] products, giving β-lactones **25** and **26** in 60% and 46% yield and >99:1 er, with ([2 + 2]:[4 + 2] = 90:10 and >95:5 respectively). Variation of the alkyl substituent within the α-silyl acid was next explored with β-phenyl trifluoromethylenone **2** used as a standard electrophile. Ethyl, allyl, alkynyl, benzyl, and 2-naphthylmethyl substituted acid derivatives demonstrated good reactivity. The desired [2 + 2] products **27**–**31** were isolated in 52% to 60% yield, 80:20 to 90:10 dr, up to >99:1 er, and with preferential [2 + 2]-cycloaddition ([2 + 2]:[4 + 2] = 80:20 to 86:14). Further exploration of the substrate scope focused on the reactivity of α-trimethylsilyl-α-methyl substituted acid with a range of α-methyl substituted β-aryl trifluoromethylenones (Table 3B). Notably, the introduction of this substitution pattern led to exclusive [2 + 2] cycloaddition in all cases. Formal [2 + 2] cycloaddition products **32**–**35** were obtained in good yields (up to 80%) with exceptional diastereoselectivity (>95:5 dr) and excellent enantioselectivity (92:8 er to >99:1 er). The absolute configuration of product **34** was confirmed by X-ray crystallographic analysis. Variation of the α-alkyl substituent within the α-trimethylsilyl acid gave products **36**–**40** in good yields (up to 78%), and excellent enantioselectivity (up to >99:1 er for the major enantiomer, 98:2 er for the minor enantiomer), although with moderate diastereoselectivity (67:33 dr to 75:25 dr). In contrast to the unsubstituted α-trimethylsilyl acetic acid **1**, α-alkyl substituted α-trimethylsilyl acids showed no reactivity with α,β-diphenyl substituted trifluoromethylenone **41**. The use of α-isopropyl-α-trimethylsilyl acid **42** also led to returned starting materials with α-methyl-β-phenyl trifluoromethylenone.

**Table 3:**
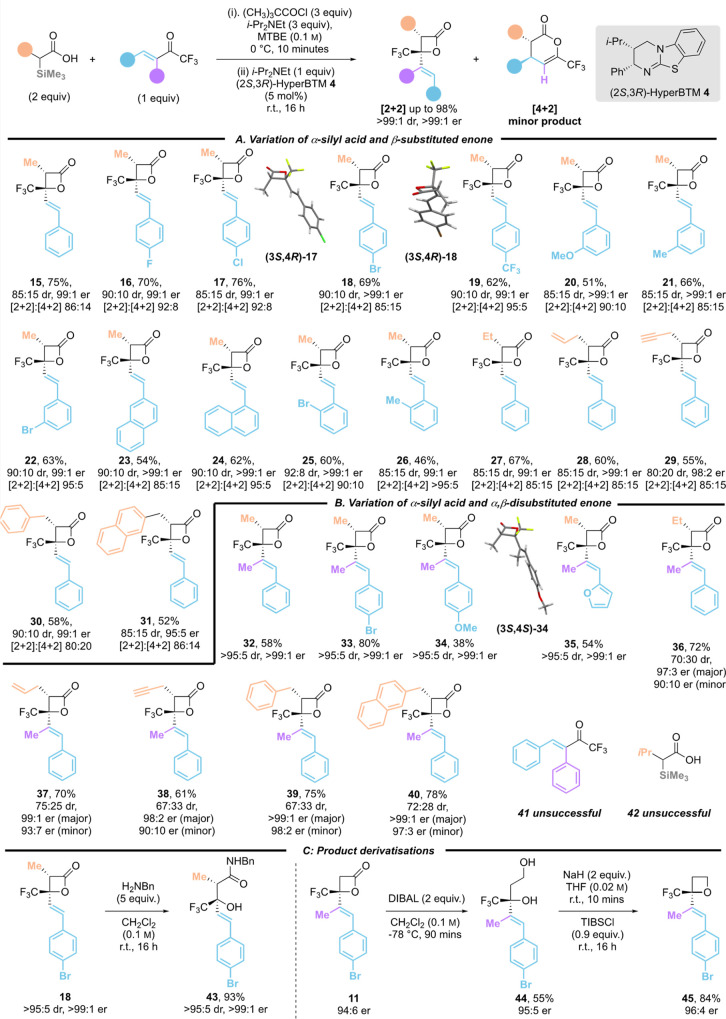
Scope and Limitations: Formal [2 + 2] Cycloaddition of α-Trimethylsilyl Acids with Trifluoromethylenones^a^^,^^b^

a Isolated yields.

b dr determined by ^1^H NMR and ^19^F NMR analysis of the crude reaction product; er determined by HPLC analysis on a chiral stationary phase.

Derivatization of β-lactone products **11** and **18** was next investigated to broaden the utility of this methodology (Table 3C). Ring-opening occurs readily with benzylamine as a nucleophile to give the enantioenriched amide product **43** in excellent yield (93%). Alternatively, transformation to an oxetane was achieved through a two-step procedure. Reduction of **11** with DIBAL-H gave diol **44** in 55% yield, followed by ring closure upon treatment with 2,4,6-triisopropylbenzenesulfonyl chloride (TIBS-Cl) to give oxetane product **45** in 84% yield without loss of enantiopurity (96:4 er).

### Mechanistic Investigations

To demonstrate the advantage of using α-silyl substituted C(1)-acids as ammonium enolate precursors, control experiments probed the relative reactivity of using acetic acid **46** or acetic anhydride **47** as starting materials. In each case, poor conversion to the corresponding β-lactone **3** was observed, resulting in low isolated product yields although promising er, demonstrating the beneficial reactivity of the silyl-acid starting material (Table 4a). Further experiments compared the reactivity of alternative α-silyl substituents. α-Trimethylsilyl acetic acid and α-trimethylsilyl propionic acid demonstrated excellent reactivity with enone **2** and furnished β-lactones **3** and **15** with exceptional enantioselectivity (92:8 er and 99:1 er respectively). Acids bearing an α-dimethylphenylsilyl group or α-diphenylmethylsilyl group were also tested, giving the corresponding products with competitive enantioselectivity and diastereoselectivity (85:15 to 88:12 dr, 96:4 to >99:1 er) (Table 4b). The product yield decreased with increased steric bulk of the silyl-substituent: for example, both α-dimethylphenylsilyl propionic acid **51** and α-diphenylmethylsilyl propionic acid **52** gave product **15** with similar enantioselectivity (96:4 er and >99:1 er), but in reduced yield (52%). Finally, the use of α-phenyl-α-trimethylsilyl acid **53** was tested, giving exclusively the formal [4 + 2]-cycloaddition product **54** in 52% yield, 87:13 dr, and 99:1 er (Table 4c). Reaction of α-phenyl-α-trimethylsilyl acid **53** with α-methyl-β-phenyl trifluoromethylenone **55** returned only starting materials.

**Table 4:**
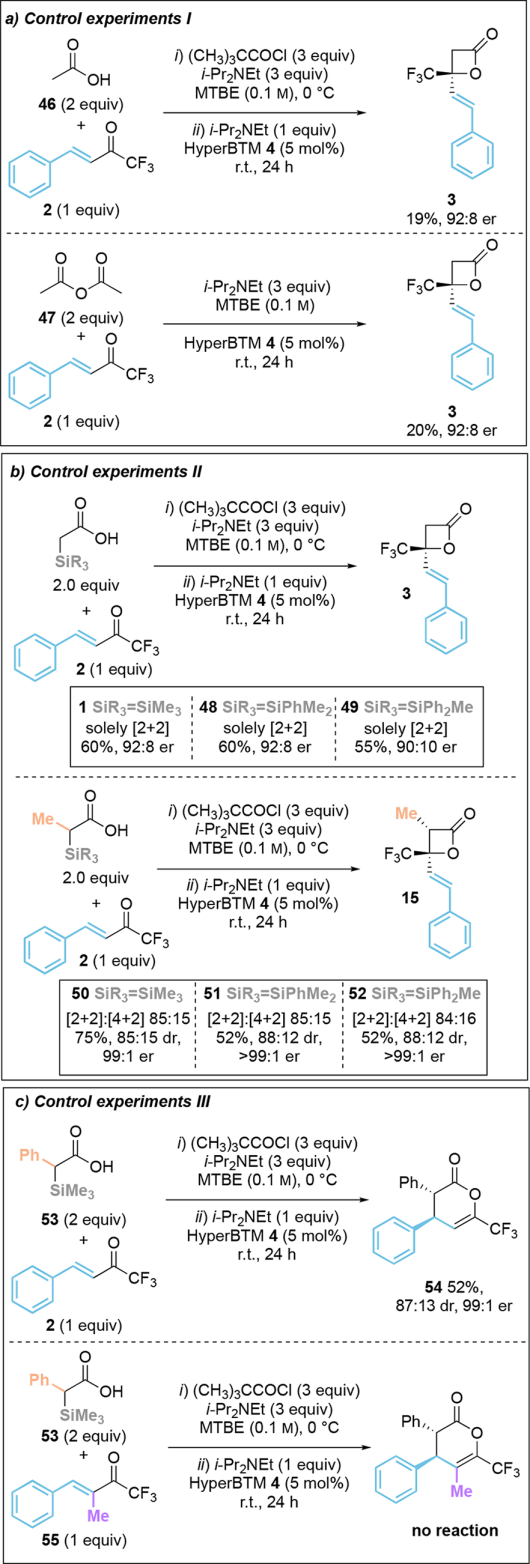
Mechanistic Control Experiments^a^^,^^b^

a Isolated yield.

b dr determined by ^1^H NMR and ^19^F NMR analysis of the crude reaction mixture; er determined by HPLC analysis on a chiral stationary phase.

Further mechanistic studies were conducted by using enantiomerically enriched acid (*R*)-**51** with 10 mol % of each enantiomer of HyperBTM **4** separately under standard conditions (Table 5a).^13^ Consistent with our previous observations,^12^ the relative rates of product formation with enantiomeric catalysts differed significantly, although identical levels of product diastereo- and enantioselectivity were observed throughout these processes. In the mismatched case, treatment of the anhydride generated from (*R*)-**51** with (2*R*,3*S*)-HyperBTM **4** led to relatively slow conversion (35% by ^19^F NMR after 480 min) to product β-lactone **15** (88:12 dr, 96:4 er, [2 + 2]:[4 + 2] = 85:15). In the matched case, treatment of the anhydride generated from (*R*)-**51** with (2*S*,3*R*)-HyperBTM **4** led to the same stereo- and regioselectivity, but with significantly enhanced conversion (70% by ^19^F NMR after 480 min) to product β-lactone **15** (88:12 dr, 96:4 er, [2 + 2]:[4 + 2] = 85:15) (Table 5a). Kinetic analysis using racemic acid **51** and **2** as the electrophile catalyzed by 10 mol % of (2*S*,3*R*)-HyperBTM **4** was monitored using ^19^F NMR under standard reaction conditions (Table 5b). The rate of formation of product **15** and the rate of consumption of enone **2** both demonstrated linear profiles consistent with a pseudo-zero-order reaction, with identical ratios of β-lactone **15** and [4 + 2] cycloaddition product **56** observed throughout the experiment, consistent with the regioselectivity being kinetically controlled rather than through product interconversion.

**Table 5:**
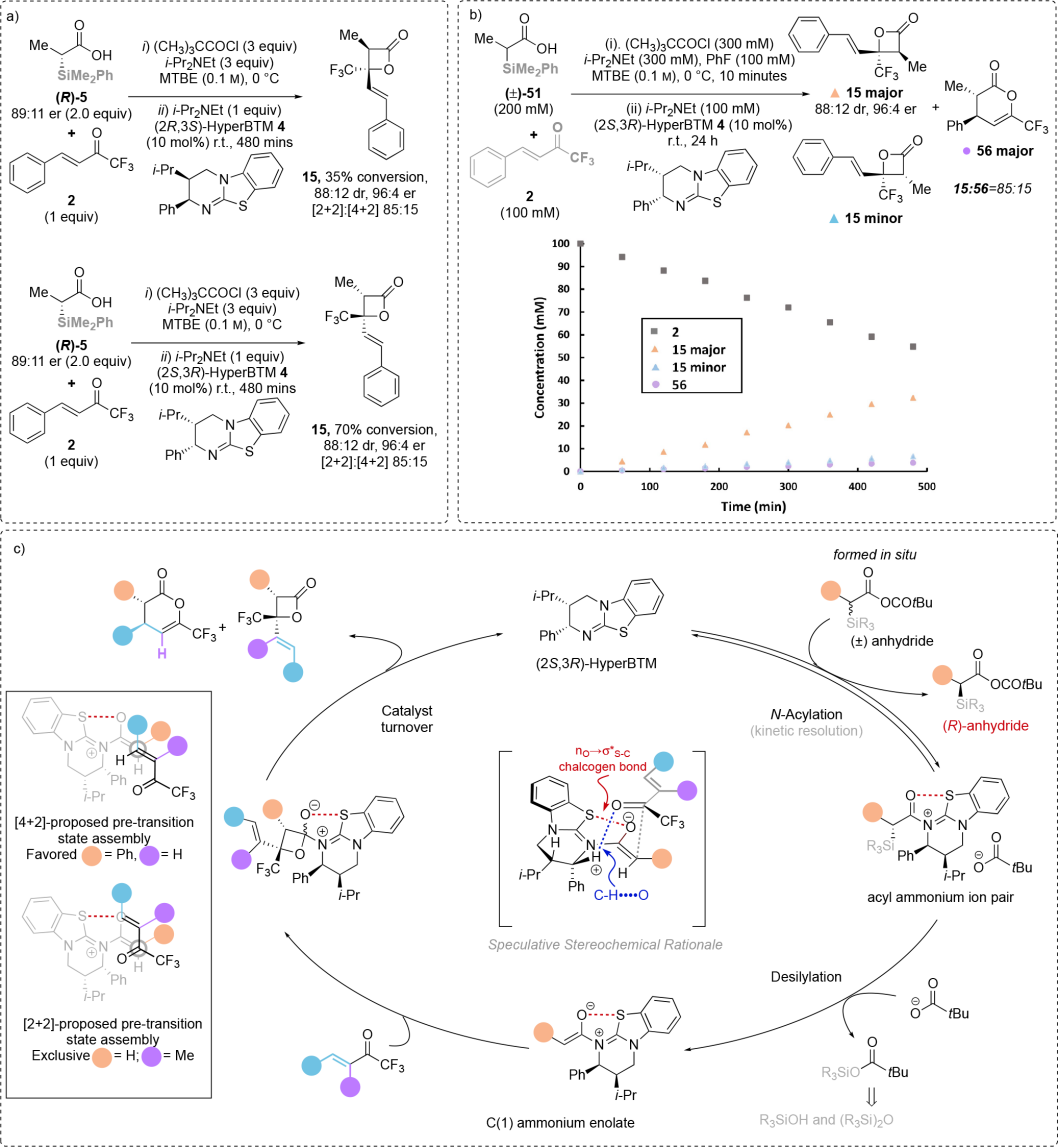
(a) Matched and Mismatched Cases; (b) Reaction Profile; (c) Proposed Catalytic Cycle^a^^,^^b^

a Isolated yield.

b dr determined by ^1^H NMR and ^19^F NMR analysis of the crude reaction mixture; er determined by HPLC analysis on a chiral stationary phase.

Building upon these observations and our previous work, the proposed mechanistic cycle involves initial N-acylation of HyperBTM with the in situ generated mixed anhydride to generate the corresponding acyl ammonium ion pair in a kinetic resolution process.^12^ Subsequent desilylation generates the C(1)-ammonium enolate that can undergo either concerted asynchronous [2 + 2] cycloaddition or [4 + 2] cycloaddition with the trifluoromethylenone.^14^ The regioselectivity of this process is dictated by steric factors within both reaction components. When α-substituted-β-aryl trifluoromethylenones are used, exclusive [2 + 2] cycloaddition to give the β-lactone products is observed. When α-unsubstituted-β-aryl trifluoromethylenone and α-alkyl-α-silyl acids are used, the C(1)-ammonium enolate can undergo both concerted asynchronous [2 + 2] cycloaddition and [4 + 2] cycloaddition, furnishing β-lactones as the major product accompanied by [4 + 2] cycloaddition as the minor product. Key to the observed stereochemical outcome is a stabilizing 1,5-O···S chalcogen bonding interaction (n_O_ to σ*_S–C_).^15−18^ This provides a conformational bias and ensures coplanarity between the 1,5-O- and S-atoms within the (*Z*)-enolate, with preferential addition *anti*- to the stereodirecting phenyl substituent within the catalyst.

## Conclusion

To conclude, a protocol for the diastereo-, enantio-, and regioselective [2 + 2] cycloaddition of β-aryl trifluoromethylenones with α-silyl carboxylic acids catalyzed by the isothiourea HyperBTM under mild and operationally simple conditions has been developed. A broad substrate scope of enantiomerically enriched β-lactone products (34 examples, up to >95:5 dr and >99:1 er) and significantly extended reactivity of C(1)-ammonium enolates has been demonstrated. Control experiments indicate that the α-substituents of the trifluoromethylenone and the α-silyl carboxylic acid play a crucial role in dictating the regioselectivity of this transformation. Solely [2 + 2] cycloaddition was observed when α-silyl acetic acids and α-methyl or α-phenyl substituted β-aryl trifluoromethylenones were used. Both [2 + 2] cycloaddition and Michael addition-lactonization reactions were observed when α-substituted-α-silyl carboxylic acids were used in conjunction with β-aryl trifluoromethylenones lacking a second α-substituent. The bench stable β-lactones are readily derivatized through ring-opening or can be transformed into the corresponding oxetanes without compromising stereochemical integrity.

## Experimental Section

### General Information

Reactions involving moisture sensitive reagents were carried out in flame-dried glassware under a nitrogen atmosphere using standard vacuum line techniques and using anhydrous solvents. HyperBTM **4** and benzotetramisole (BTM) **5** were synthesized in house. Tetramisole·HCl **6** was obtained from Sigma-Aldrich. Anhydrous solvents (CH_2_Cl_2_, PhMe) was obtained after passing through an alumina column (Mbraun SPS-800). Anhydrous MTBE and MeCN was obtained by treatment with activated 4 Å molecular sieves. Petrol is defined as petroleum ether 40–60 °C. All other solvents and commercial reagents were used as supplied without further purification unless otherwise stated. EtOAc, Et_2_O, CH_2_Cl_2_, and petrol for purification purposes were used as obtained from suppliers without further purification. Room temperature (r.t.) refers to 20–25 °C. Temperatures of 0 °C and −78 °C were obtained using ice/water and CO_2_(*s*)/acetone baths, respectively. Reactions involving heating were performed using a DrySyn block and a contact thermocouple. In vacuo refers to the use either a Büchi Rotavapor R-200 with a Büchi V-491 heating bath and Büchi V-800 vacuum controller; a Büchi Rotavapor R-210 with a Büchi V-491 heating bath and Büchi V-850 vacuum controller; a Heidolph Laborota 4001 with vacuum controller; an IKA RV10 rotary evaporator with an IKA HB10 heating bath and ILMVAC vacuum controller; or an IKA RV10 rotary evaporator with an IKA HB10 heating bath and Vacuubrand CVC3000 vacuum controller. Rotary evaporator condensers are fitted to Julabo FL601 Recirculating Coolers filled with ethylene glycol set to −6 °C. Analytical thin layer chromatography was performed on precoated aluminum plates (Kieselgel 60 F254 silica). TLC visualization was carried out with ultraviolet light (254 nm), followed by staining with a 1% aqueous KMnO_4_ solution. Automated chromatography was performed on a Biotage Isolera Four running Biotage OS578 with a UV–vis detector using the method stated and cartridges filled with Kieselgel 60 silica. Melting points were recorded on an Electrothermal 9100 melting point apparatus and are uncorrected. Optical rotations were measured on a PerkinElmer Precisly/Model-341 polarimeter operating at the sodium D line with a 100 mm path cell at 20 °C. HPLC analyses were obtained using either a Shimadzu HPLC consisting of a DGU-20A5 degassing unit, LC-20AT liquid chromatography pump, SIL-20AHT autosampler, CMB-20A communications bus module, SPD-M20A diode array detector and a CTO-20A column oven; or a Shimadzu HPLC consisting of a DGU-20A5R degassing unit, LC-20AD liquid chromatography pump, SIL-20AHT autosampler, SPD-20A UV–vis detector and a CTO-20A column oven. Separation was achieved using DAICEL CHIRALPAK AS-H, AD-H, and IB columns, CHIRALCEL OD-H and OJ-H using the method stated. HPLC traces of enantiomerically enriched compounds were compared with authentic racemic spectra. ^1^H, ^13^C{^1^H}, and ^19^F{^1^H} nuclear magnetic resonance (NMR) spectra were acquired on either a Bruker Avance II 400 (^1^H 400 MHz; ^13^C{^1^H} 101 MHz; ^19^F{^1^H} 376 MHz) or a Bruker Avance II 500 (^1^H 500 MHz; ^13^C{^1^H} 126 MHz; ^19^F{^1^H} 471 MHz) spectrometer at ambient temperature in the deuterated solvent stated. All chemical shifts are quoted in parts per million (ppm) and referenced to the residual solvent peak. All coupling constants, *J*, are quoted in Hz. Multiplicities are indicated by s (singlet), d (doublet), t (triplet), q (quartet), dd (doublet of doublets), dt (doublet of triplets), dq (doublet of quartets), tt (triplet of triplets), ddd (doublet of doublet of doublets), and m (multiplet). The abbreviation Ar is used to denote aromatic, Ph to denote phenyl, Bn to denote benzyl, br to denote broad, and app to denote apparent. NMR peak assignments were confirmed using 2D ^1^H correlated spectroscopy (COSY), ^1^H–^13^C heteronuclear single quantum coherence (HSQC), and 2D ^1^H–^13^C heteronuclear multiple-bond correlation spectroscopy (HMBC) where necessary. Infrared spectra were recorded on a Shimadzu IRAffinity-1 Fourier transform IR spectrophotometer fitted with a Specac Quest ATR accessory (diamond puck). Spectra were recorded of either thin films or solids, with characteristic absorption wavenumbers (ν_max_) reported in cm^–1^. Mass spectrometry (HRMS) data were acquired by electrospray ionization (ESI) at either the University of St Andrews Mass Spectrometry Facility or at the EPSRC UK National Mass Spectrometry Facility at Swansea University.

### General Procedures

#### General Experimental Procedure A: Synthesis of α-Silyl Acids

According to a procedure reported by Rogers et al.,^19^ diisopropylamine (9.6 mmol, 2.1 equiv) was dissolved in THF (10 mL) under an N_2_-atmosphere. The solution was cooled to −78 °C and *n*-BuLi (9.6 mmol, 2.1 equiv) was added. The mixture was warmed to r.t. for 15 min before being cooled to −78 °C again. 2-(Trimethylsilyl) acetic acid (4.5 mmol, 1.0 equiv) was added and the mixture was stirred at 0 °C for 1 h, followed by 1.5 h at r.t. Subsequently the specified halide (4.7 mmol, 1.05 equiv) was added at 0 °C and the mixture was stirred additional 30 min at 0 °C. Then the reaction was quenched by the addition of HCl (1 M) and the pH adjusted to 2. The aqueous layer was extracted with Et_2_O (3 × 15 mL). The combined organic layers were dried over MgSO_4_, filtered, and the solvent was removed under reduced pressure. The crude residue was triturated from pentane to give the desired product.

#### General Experimental Procedure B: Synthesis of Alternative α-Silyl Acids

According to a procedure reported by Becker et al.,^20^ to an oven-dried round-bottomed flask (250 mL) equipped with a magnetic stirring bar were added diisopropylamine (24.0 mmol, 1.15 equiv) and anhydrous THF (40 mL). The mixture was cooled to −78 °C, and then *n*-BuLi 1.6 M (24.0 mmol, 1.15 equiv) was added dropwise. The mixture was warmed to r.t. for 15 min and cooled again to −78 °C. Trimethylsilyl acetate (CH_3_CO_2_SiMe_3_) (21.0 mmol, 1.0 equiv) was added dropwise to the cooled solution of LDA over 15 min and the reaction mixture was stirred for 2 h at −78 °C. Then chlorosilane (24.0 mmol, 1.15 equiv) in anhydrous THF (5 mL) was added dropwise to the solution over 10 min. The reaction mixture was then stirred at −78 °C for 2 additional hours and allowed to reach room temperature overnight. A solution of saturated aqueous NaCl solution (30 mL) was added, and the pH was adjusted to 3 using 1 M aqueous HCl. The aqueous layer was extracted with Et_2_O (3 × 30 mL) and the combined organic extracts were washed with water, dried over MgSO_4_, filtered, and concentrated under reduced pressure. The residual crude product was dissolved in THF (30 mL) and saturated aqueous NH_4_Cl solution (20 mL) was added. The reaction mixture was then stirred at room temperature for 1 h. Afterward, the aqueous layer was extracted with Et_2_O (3 × 30 mL) and the combined organic extracts were washed with water (30 mL), dried over MgSO_4_, filtered, and concentrated under reduced pressure. The crude residue was crystallized from hexane to give the desired product.

#### General Experimental Procedure C: Synthesis of Trifluoromethylenones

According to a procedure reported by Davies et al.,^9b^ the requisite aldehyde (1.0 equiv), piperidine (1.0 equiv), and acetic acid (1.5 equiv) were dissolved in toluene (0.5 M) at 0 °C. A solution of trifluoromethyl ketone (2.0–4.0 equiv) in toluene (2–4 M) was added and the reaction was stirred for 2 h at 0 °C, followed by heating at 50 °C for 16 h. The reaction was cooled to r.t. and quenched with saturated aqueous NH_4_Cl solution. The organic layer was washed with water, dried over Na_2_SO_4_, filtered, and concentrated under reduced pressure to leave the crude product, which was purified by flash column chromatography on silica.

#### General Experimental Procedure D: Synthesis of β-Lactones

In a flame-dried Schlenk tube under an N_2_ atmosphere, *N*,*N*-diisopropylethylamine (3.0 equiv) and pivaloyl chloride (3.0 equiv) were added sequentially to a solution of appropriate acid (2.0 equiv) in anhydrous MTBE (0.1 M) at 0 °C. The mixture was allowed to stir for 15 min at 0 °C, followed by the sequential addition of the specified ketone (1.0 equiv), (2*S*,3*R*)-HyperBTM (5 mol %), and *N*,*N*-diisopropylethylamine (1.0 equiv). The mixture was allowed to stir for the specified time at r.t. The solvent was then removed under reduced pressure, and the crude residue purified by Biotage automated column chromatography in the stated solvent system to give the desired product.

##### (*S*,*E*)-4-Styryl-4-(trifluoromethyl)oxetan-2-one **(3)**

Following General Procedure D, 2-(trimethylsilyl) acetic acid (66 mg, 0.5 mmol), *N*,*N*-diisopropylethylamine (132 μL, 0.75 mmol), pivaloyl chloride (92 μL, 0.75 mmol), and MTBE (3 mL) for 15 min, followed by (*E*)-1,1,1-trifluoro-4-phenylbut-3-en-2-one (50 mg, 0.25 mmol), (2*S*,3*R*)-HyperBTM (4 mg, 12.5 μmol), and *N*,*N*-diisopropylethylamine (44 μL, 0.25 mmol) for 16 h gave, after purification by Biotage Isolera 4 [SNAP KP-Sil 25 g, 36 mL min^–1^, petrol:Et_2_O (98:2 4 CV, 98:2 to 90:10 30 CV)], the title compound (45 mg, 75%) as a bright yellow oil. [α]_D_^20^ −19.3 (*c* 1.4, CHCl_3_); Chiral HPLC analysis, Chiralpak IB (99.3:0.7 hexane:IPA, flow rate 1.0 mL min^–1^, 254 nm, 30 °C), *t*_R_ (major): 16.1 min, *t*_R_ (minor): 24.9 min, 92:8 er. IR ν_max_ (film) 1852 (C=O), 1167 (C–O), 972 (C=C). ^1^H NMR (400 MHz, CDCl_3_) δ_H_ 7.57–7.31 (5H, m, Ph*H*), 7.02 (1H, d, *J* 16.0, CH=C*H*Ph), 6.41 (1H, d, *J* 16.0, C*H*=CHPh), 3.90 (1H, d, *J* 16.4, C(3)H_A_*H*_B_), 3.64–3.59 (1H, dq, *J* 16.6, 2.2, C(3)*H*_A_H_B_). ^19^F NMR (376 MHz, CDCl_3_) δ_F_ −79.9 (C*F*_3_). ^13^C{^1^H} NMR (126 MHz, CDCl_3_) δ_C_ 163.6 (*C*(2)), 136.8 (*C*H=CHPh), 134.2 (Ph*C*(1)), 129.5 (Ph*C*(4)H), 128.9 (Ph*C*(3,5)H), 127.2 (Ph*C*(2,6)H), 123.3 (q, *J* 281.0, *C*F_3_), 117.4 (CH=*C*HPh), 74.2 (q, *J* 34.2, *C*(4)), 46.0 (*C*(3)H_2_). HRMS (ESI+) *m*/*z* [M + Na]^+^ calcd for C_12_H_9_O_2_F_3_Na 265.0447, found 265.0445.

##### (*S*,*E*)-4-(4-Bromostyryl)-4-(trifluoromethyl)oxetan-2-one **(7)**

Following General Procedure D, 2-(trimethylsilyl) acetic acid (66 mg, 0.5 mmol), *N*,*N*-diisopropylethylamine (132 μL, 0.75 mmol), pivaloyl chloride (92 μL, 0.75 mmol), and MTBE (3 mL) for 15 min, followed by (*E*)-4-(4-bromophenyl)-1,1,1-trifluorobut-3-en-2-one (70 mg, 0.25 mmol), (2*S*,3*R*)-HyperBTM (4 mg, 12.5 μmol), and *N*,*N*-diisopropylethylamine (44 μL, 0.25 mmol) for 16 h gave, after purification by Biotage Isolera 4 [SNAP KP-Sil 25 g, 36 mL min^–1^, petrol:Et_2_O (98:2 4 CV, 98:2 to 92:8 40 CV)], the title compound (63 mg, 78%) as a a white solid. mp 52–54 °C. [α]_D_^20^ −33.2 (*c*, 0.5, CHCl_3_). Chiral HPLC analysis, Chiralpak IB (99:1 hexane:IPA, flow rate 1.0 mL min^–1^, 254 nm, 30 °C), *t*_R_ (major): 12.7 min, *t*_R_ (minor): 15.6 min, 92:8 er. IR ν_max_ (film) 1854 (C=O), 1165 (C–O), 972 (C=C). ^1^H NMR (400 MHz, CDCl_3_) δ_H_ 7.56–7.53 (2H, m, ArC(3,5)*H*), 7.36–7.33 (2H, m, ArC(2,6)*H*), 6.97 (1H, d, *J* 16.0, CH=C*H*Ar), 6.39 (1H, d, *J* 16.0, C*H*=CHAr), 3.90 (1H, d, *J* 16.6, C(3)H_A_*H*_B_), 3.60 (1H, dq, *J* 16.5, 1.2, C(3)*H*_A_H_B_). ^19^F NMR (376 MHz, CDCl_3_) δ_F_ −79.9 (C*F*_3_). ^13^C{^1^H} NMR (101 MHz, CDCl_3_) δ_C_ 163.4 (*C*(2)), 135.6 (*C*H=CHAr), 133.2 (Ph*C*(1)), 132.1 (Ar*C*(3,5)H), 128.7 (Ar*C*(2,6)H), 123.6 (Ar*C*(4)), 123.1 (q, *J* 280.6, *C*F_3_), 118.2 (CH=*C*HAr), 74.1 (q, *J* 34.2, *C*(4)), 46.1 (*C*(3)). HRMS (ESI+) *m*/*z* [M + OH]^−^ calcd for C_12_H_9_BrF_3_O_3_ 336.9693 (−0.3 ppm), found 336.9692.

##### (*S*,*E*)-4-(2-Bromostyryl)-4-(trifluoromethyl)oxetan-2-one **(8)**

Following General Procedure D, 2-(trimethylsilyl) acetic acid (66 mg, 0.5 mmol), *N*,*N*-diisopropylethylamine (132 μL, 0.75 mmol), pivaloyl chloride (92 μL, 0.75 mmol), and MTBE (3 mL) for 15 min, followed by (*E*)-4-(2-bromophenyl)-1,1,1-trifluorobut-3-en-2-one (70 mg, 0.25 mmol), (2*S*,3*R*)-HyperBTM (4 mg, 12.5 μmol), and *N*,*N*-diisopropylethylamine (44 μL, 0.25 mmol) for 16 h gave, after purification by Biotage Isolera 4 [SNAP KP-Sil 25 g, 36 mL min^–1^, petrol:Et_2_O (98:2 4 CV, 98:2 to 92:8 40 CV)], the title compound (61 mg, 76%) as a bright yellow oil. [α]_D_^20^ −16.8 (*c*, 0.5, CHCl_3_). Chiral HPLC analysis, Chiralpak IB (99:1 hexane:IPA, flow rate 1.0 mL min^–1^, 254 nm, 30 °C), *t*_R_ (minor): 13.5 min, *t*_R_ (major): 16.6 min, 90:10 er. IR ν_max_ (film) 1852 (C=O), 1167 (C–O), 970 (C=C). ^1^H NMR (400 MHz, CDCl_3_) δ_H_ 7.64–7.62 (1H, m, ArC(3)*H*), 7.57–7.55 (1H, m, ArC(5)*H*), 7.38 (1H, d, *J* 16.0, CH=C*H*Ar), 7.38–7.34 (1H, m, ArC(4)*H*), 7.26–7.22 (1H, m, ArC(6)*H*), 6.36 (1H, d, *J* 16.2, C*H*=CHAr), 3.92 (1H, d, *J* 16.6, C(3)H_A_*H*_B_), 3.67 (1H, dq, *J* 16.6, 1.1, C(3)*H*_A_H_B_). ^19^F NMR (376 MHz, CDCl_3_) δ_F_ −79.8 (C*F*_3_). ^13^C{^1^H} NMR (101 MHz, CDCl_3_) δ_C_ 163.4 (*C*(2)), 136.0 (*C*H=CHAr), 134.4 (Ar*C*(1)), 133.3 (Ar*C*(3)H), 130.7 (Ar*C*(5)H), 127.8 (Ar*C*(6)H), 127.5 (Ar*C*(4)H), 124.3 (Ar*C*(2)), 123.2 (q, *J* 282.1, *C*F_3_), 120.5 (CH=*C*HAr), 74.1 (q, *J* 34.6, *C*(4)), 45.9 (*C*(3)H_2_). HRMS (ESI+) *m*/*z* [M + OH]^−^ calcd for C_12_H_9_BrF_3_O_3_ 336.9693, found 336.9694.

##### (*S*,*E*)-4-(3-Methoxystyryl)-4-(trifluoromethyl)oxetan-2-one **(9)**

Following General Procedure D, 2-(trimethylsilyl) acetic acid (66 mg, 0.5 mmol), *N*,*N*-diisopropylethylamine (132 μL, 0.75 mmol), pivaloyl chloride (92 μL, 0.75 mmol), and MTBE (3 mL) for 15 min, followed by (*E*)-1,1,1-trifluoro-4-(3-methoxyphenyl)but-3-en-2-one (58 mg, 0.25 mmol), (2*S*,3*R*)-HyperBTM (4 mg, 12.5 μmol), and *N*,*N*-diisopropylethylamine (44 μL, 0.25 mmol) for 16 h gave, after purification by Biotage Isolera 4 [SNAP KP-Sil 25 g, 36 mL min^–1^, petrol:Et_2_O (98:2 4 CV, 98:2 to 92:8 40 CV)], the title compound (44 mg, 65%) as a bright yellow oil. [α]_D_^20^ −7.6 (*c*, 0.2, CHCl_3_). Chiral HPLC analysis, Chiralpak AS-H (99.5:0.5 hexane:IPA, flow rate 1.0 mL min^–1^, 254 nm, 30 °C), *t*_R_ (minor): 11.1 min, *t*_R_ (major): 15.3 min, 89:11 er; IR ν_max_ (film) 1856 (C=O), 1165 (C–O), 974 (C=C); ^1^H NMR (400 MHz, CDCl_3_) δ_H_ 7.35–7.31 (1H, m, ArC(5)*H*), 7.08–7.06 (1H, m, ArC(6)*H*), 6.99 (1H, d, *J* 16.0, CH=C*H*Ph), 6.99–6.98 (1H, m, ArC(2)*H*), 6.95–6.92 (1H, m, ArC(4)*H*), 6.39 (1H, d, *J* 16.0, C*H*=CHAr), 3.90 (1H, d, *J* 16.5, C(3)H_A_*H*_B_), 3.86 (3H, s, OC*H*_3_), 3.61 (1H, dq, *J* 16.5, 1.1, C(3)*H*_A_H_B_). ^19^F NMR (376 MHz, CDCl_3_) δ_F_ −79.8 (C*F*_3_). ^13^C{^1^H} NMR (101 MHz, CDCl_3_) δ_C_ 163.6 (*C*(2)), 160.0 Ar*C*(3)), 136.7 (*C*H=CHAr), 135.6 (Ar*C*(1)), 130.0 (Ar*C*(5)H), 123.2 (q, *J* 281.2, *C*F_3_), 119.7 (Ar*C*(6)H), 117.7 (CH=*C*HPh), 115.2 (Ar*C*(4)H), 112.4 (Ar*C*(2)H), 74.2 (q, *J* 34.4, *C*(4)), 55.4 (O*C*H_3_), 46.0 (*C*(3)H_2_). HRMS (ESI+) *m*/*z* [M + Na]^+^ calcd for C_13_H_11_F_3_NaO_3_ 295.0552, found 295.0548.

##### (*S*,*E*)-4-(1-Phenylprop-1-en-2-yl)-4-(trifluoromethyl)oxetan-2-one **(10)**

Following General Procedure D, 2-(trimethylsilyl)acetic acid (53 mg, 0.4 mmol), *N*,*N*-diisopropylethylamine (105 μL, 0.6 mmol), pivaloyl chloride (66 μL, 0.6 mmol), and MTBE (2 mL) for 15 min, followed by (*E*)-1,1,1-trifluoro-3-methyl-4-phenylbut-3-en-2-one (40 mg, 0.2 mmol), (2*S*,3*R*)-HyperBTM (3 mg, 10.0 μmol), and *N*,*N*-diisopropylethylamine (35 μL, 0.2 mmol) for 16 h gave, after purification by Biotage Isolera 4 [SNAP KP-Sil 10 g, 36 mL min^–1^, petrol:Et_2_O (99:1 4 CV, 99:1 to 90:10 20 CV)], the title compound (41 mg, 80%) as a colorless oil. [α]_D_^20^ −1.6 (*c*, 0.5, CHCl_3_). Chiral HPLC analysis, Chiralcel OJ-H (99.8:0.2 hexane:IPA, flow rate 1.0 mL min^–1^, 254 nm, 30 °C), *t*_R_ (minor): 34.5 min, *t*_R_ (major): 38.3 min, 92:8 er. IR ν_max_ (film) 1852 (C=O), 1175 (C–O). ^1^H NMR (500 MHz, CDCl_3_) δ_H_ 7.44–7.41 (2H, m, Ph*H*), 7.35–7.33 (3H, m, Ar*H*), 6.84 (1H, s, C*H*Ph), 3.90 (1H, d, *J* 16.6, C(3)H_A_*H*_B_), 3.64 (1H, dq, *J* 16.5, 1.1, C(3)*H*_A_H_B_), 2.06 (3H, m, C*H*_3_). ^19^F NMR (471 MHz, CDCl_3_) δ_F_ −78.2 (s, C*F*_3_). ^13^C{^1^H} NMR (126 MHz, CDCl_3_) δ_C_ 163.9 (*C*O), 135.2 (Ph*C*(1)), 132.9 (Ar*C*H), 129.1 (Ph*C*(3,5)H), 128.5 (Ph*C*(2,6)H), 128.0 (Ph*C*(4)H), 127.8 (PhCH=*C*CH_3_), 123.6 (q, *J* 284.6, *C*F_3_), 76.8 (q, *J* 32.9, *C*CF_3_), 45.4 (C(3)), 14.2 (*C*H_3_). HRMS (ESI+) *m*/*z* [M + OH]^−^ calcd for C_13_H_12_F_3_O_3_ 273.0744, found 273.0742.

##### (*S*,*E*)-4-(1-(4-Bromophenyl)prop-1-en-2-yl)-4-(trifluoromethyl)oxetan-2-one **(11)**

Following General Procedure D, 2-(trimethylsilyl)acetic acid (265 mg, 2.0 mmol), *N*,*N*-diisopropylethylamine (522 μL, 3.0 mmol), pivaloyl chloride (367 μL, 3.0 mmol), and MTBE (5 mL) for 15 min, followed by (*E*)-4-(4-bromophenyl)-1,1,1-trifluoro-3-methylbut-3-en-2-one (293 mg, 1.0 mmol), (2*S*,3*R*)-HyperBTM (15 mg, 100.0 μmol), and *N*,*N*-diisopropylethylamine (241 μL, 1.5 mmol) for 16 h gave, after purification by Biotage Isolera 4 [SNAP KP-Sil 10 g, 36 mL min^–1^, petrol:Et_2_O (99:1 4 CV, 99:1 to 90:10 30 CV)], the title compound (288 mg, 86%) as a colorless oil; [α]_D_^20^ +1.0 (*c*, 0.4, CHCl_3_); Chiral HPLC analysis, Chiralpak AS-H (99.5:0.5 hexane:IPA, flow rate 1.0 mL min^–1^, 254 nm, 30 °C), *t*_R_ (minor): 7.9 min, *t*_R_ (major): 9.7 min, 94:6 er; IR ν_max_ (film) 1848 (C=O), 1173 (C–O); ^1^H NMR (400 MHz, CDCl_3_) δ_H_ 7.56–7.53 (2H, m, ArC(3,5)*H*), 7.22–7.19 (2H, m, ArC(2,6)*H*), 6.77 (1H, s, C*H*Ar), 3.90 (1H, d, *J* 16.4, C(3)H_A_*H*_B_), 3.64–3.60 (1H, dq, *J* 16.5, 1.1, C(3)*H*_A_H_B_), 2.02 (3H, t, *J* 1.2, C*H*_3_). ^19^F NMR (377 MHz, CDCl_3_) δ_F_ −78.2 (s, C*F*_3_). ^13^C{^1^H} NMR (101 MHz, CDCl_3_) δ_C_ 163.7 (*C*O), 134.1 (Ar*C*(1)), 131.7 (Ar*C*H=CCH_3_, Ar*C*(3,5)H), 130.7 (Ar*C*(2,6)H), 128.6 (ArCH=*C*CH_3_), 123.5 (q, *J* 282.9, *C*F_3_), 122.1 (Ar*C*(4)), 76.7 (q, *J* 33.2, *C*CF_3_), 45.4 (C(3)), 14.2 (*C*H_3_). HRMS (ESI+) *m*/*z* [M + H]^+^ calcd for C_13_H_11_BrF_3_O_2_ 334.9889, found 334.9888.

##### (*S*,*E*)-4-(1-(4-Methoxyphenyl)prop-1-en-2-yl)-4-(trifluoromethyl)oxetan-2-one **(12)**

Following General Procedure D, 2-(trimethylsilyl)acetic acid (106 mg, 0.8 mmol), *N*,*N*-diisopropylethylamine (210 μL, 1.2 mmol), pivaloyl chloride (132 μL, 1.2 mmol), and MTBE (4 mL) for 15 min, followed by (*E*)-1,1,1-trifluoro-4-(4-methoxyphenyl)-3-methylbut-3-en-2-one (98 mg, 0.4 mmol), (2*S*,3*R*)-HyperBTM (6 mg, 20.0 μmol), and *N*,*N*-diisopropylethylamine (70 μL, 0.4 mmol) for 16 h gave, after purification by Biotage Isolera 4 [SNAP KP-Sil 10 g, 36 mL min^–1^, petrol:Et_2_O (99:1 4 CV, 99:1 to 90:10 30 CV)], the title compound (60 mg, 52%) as a colorless oil; [α]_D_^20^ +8.0 (*c*, 0.2, CHCl_3_); Chiral HPLC analysis, Chiralcel OD-H (99.5:0.5 hexane:IPA, flow rate 1.0 mL min^–1^, 211 nm, 30 °C), *t*_R_ (minor): 29.0 min, *t*_R_ (major): 35.4 min, 93:7 er; IR ν_max_ (film) 1850 (C=O), 1175 (C–O); ^1^H NMR (400 MHz, CDCl_3_) δ_H_ 7.32–7.28 (2H, m, ArC(2,6)*H*), 6.96–6.93 (2H, m, ArC(3,5)*H*), 6.75 (1H, s, C*H*Ph), 3.88 (1H, d, *J* 16.4, C(3)H_A_*H*_B_), 3.86 (3H, s, OC*H*_3_), 3.64–3.59 (1H, dq, *J* 16.4, 1.1, C(3)*H*_A_H_B_), 2.06 (3H, t, *J* 1.2, C*H*_3_). ^19^F NMR (377 MHz, CDCl_3_) δ_F_ −78.3 (s, C*F*_3_). ^13^C{^1^H} NMR (101 MHz, CDCl_3_) δ_C_ 163.9 (*C*O), 159.3 (Ar*C*(4)), 132.3 (Ar*C*H=CCH_3_), 130.6 (Ar*C*(2,6)H), 127.8 (PhCH=*C*CH_3_), 125.7 (Ph*C*(1)), 123.6 (q, *J* 282.6, *C*F_3_), 113.9 (Ar*C*(3,5)H), 76.9 (q, *J* 32.2, *C*CF_3_), 55.3 (O*C*H_3_), 45.3 (C(3)), 14.2 (*C*H_3_). HRMS (ESI+) *m*/*z* [M + H]^+^ calcd for C_14_H_14_F_3_O_3_ 287.0890, found 287.0887.

##### (*S*,*E*)-4-(1,2-Diphenylvinyl)-4-(trifluoromethyl)oxetan-2-one **(13)**

Following General Procedure D, 2-(trimethylsilyl) acetic acid (105 mg, 0.8 mmol), *N*,*N*-diisopropylethylamine (210 μL, 1.2 mmol), pivaloyl chloride (132 μL, 1.2 mmol), and MTBE (4.5 mL) for 15 min, followed by (*E*)-1,1,1-trifluoro-3,4-diphenylbut-3-en-2-one (110 mg, 0.4 mmol), (2*S*,3*R*)-HyperBTM (6 mg, 20.0 μmol), and *N*,*N*-diisopropylethylamine (71 μL, 0.4 mmol) for 16 h gave, after purification by Biotage Isolera 4 [SNAP KP-Sil 25 g, 36 mL min^–1^, petrol:Et_2_O (98:2 4 CV, 98:2 to 92:8 40 CV)], the title compound (117 mg, 92%) as a colorless oil; [α]_D_^20^ +2.1 (*c*, 0.2, CHCl_3_); Chiral HPLC analysis, Chiralpak IB (99:1 hexane:IPA, flow rate 1.0 mL min^–1^, 211 nm, 30 °C), *t*_R_ (major): 12.6 min, *t*_R_ (minor): 14.5 min, 93:7 er; IR ν_max_ (film) 1854 (C=O), 1152 (C–O); ^1^H NMR (500 MHz, CDCl_3_) δ_H_ 7.43–7.41 (3H, m, CHPhC(2,4,6)), 7.26–7.23 (2H, m, CHPhC(3,5)), 7.22–7.15 (3H, m, CPhC(3,4,5)*H*), 7.09 (1H, s, PhC*H*=C), 6.97–6.94 (2H, m, CPhC(2,6)*H*), 3.82 (1H, d, *J* 16.8, C(3) H_A_*H*_B_), 3.75 (1H, d, *J* 16.8, C(3)*H*_A_H_B_). ^19^F NMR (471 MHz, CDCl_3_) δ_F_ −76.6 (C*F*_3_). ^13^C{^1^H} NMR (126 MHz, CDCl_3_) δ_C_ 163.8 (*C*(2)), 135.1 (*C*HPh), 134.9 (*C*Ph), 134.4 (CPh*C*(1)), 131.6 (CHPh*C*(1)), 129.9 (CPh*C*(3,5)H), 129.7 (CHPh*C*(3,5)H), 129.2 (CHPh*C*(2,6)H), 128.7 (CPh*C*(4)H), 128.5 (CHPh*C*(4)H), 128.2 (CPh*C*(2,6)H), 123.5 (q, *J* 281.7, *C*F_3_), 76.3 (q, *J* 33.0, *C*(4)), 45.6 (*C*(3)). HRMS (ESI+) *m*/*z* [M + OH]^−^ calcd for C_18_H_14_F_3_O_3_ 335.0901, found 335.0902.

##### (3*S*,4*R*)-3-Methyl-4-((*E*)-styryl)-4-(trifluoromethyl)oxetan-2-one **(15)**

Following General Procedure D, 2-(trimethylsilyl)propanoic acid (73 mg, 0.5 mmol), *N*,*N*-diisopropylethylamine (132 μL, 0.75 mmol), pivaloyl chloride (92 μL, 0.75 mmol), and MTBE (3 mL) for 15 min, followed by (*E*)-1,1,1-trifluoro-4-phenylbut-3-en-2-one (50 mg, 0.25 mmol), (2*S*,3*R*)-HyperBTM (4 mg, 12.5 μmol), and *N*,*N*-diisopropylethylamine (44 μL, 0.25 mmol) for 20 h gave, after purification by Biotage Isolera 4 [SNAP KP-Sil 25 g, 36 mL min^–1^, petrol:Et_2_O (98:2 4 CV, 98:2 to 95:5 40 CV)], the title compound (48 mg, 75%) as a colorless oil; [α]_D_^20^ −118.4 (*c* 1.1, CHCl_3_); Chiral HPLC analysis, Chiralpak IB (99.5:0.5 hexane:IPA, flow rate 0.7 mL min^–1^, 211 nm, 30 °C), major diastereoisomer: *t*_R_ (minor): 7.4 min, *t*_R_ (major): 8.7 min, 94:6 er; minor diastereoisomer: *t*_R_ (major): 12.1 min, *t*_R_ (minor): 13.9 min, 77:23 er; IR ν_max_ (film) 1852 (C=O), 1165 (C–O); ^1^H NMR (400 MHz, CDCl_3_) δ_H_ 7.50–7.48 (2H, m, PhC(2,6)*H*), 7.47–7.40 (3H, m, PhC(3,4,5)*H*), 7.05 (1H, d, *J* 16.0, CH=C*H*Ph), 6.21 (1H, d, *J* 16.0, C*H*=CHPh), 4.10 (1H, q, *J* 7.7, C(3)*H*), 1.36 (3H, d, *J* 7.7, C(3)C*H*_3_). ^19^F NMR (376 MHz, CDCl_3_) δ_F_ −79.7 (C*F*_3_). ^13^C{^1^H} NMR (126 MHz, CDCl_3_) δ_C_ 163.6 (*C*(2)), 136.8 (*C*H=CHPh), 134.2 (Ph*C*(1)), 129.5 (Ph*C*(4)H), 128.9 (Ph*C*(3,5)H), 127.2 (Ph*C*(2,6)H), 123.3 (q, *J* 281.0, *C*F_3_), 117.4 (CH=*C*HPh), 78.3 (q, *J* 33.2, *C*(4)), 52.0 (*C*(3)H), 9.7 (C(3)*C*H_3_); HRMS (ESI+) *m*/*z* [M + H]^+^ calcd for C_13_H_12_F_3_O_2_ 257.0784, found 257.0784.

##### (3*S*,4*R*)-4-((*E*)-4-Fluorostyryl)-3-methyl-4-(trifluoromethyl)oxetan-2-one **(16)**

Following General Procedure D, 2-(trimethylsilyl)propanoic acid (117 mg, 0.8 mmol), *N*,*N*-diisopropylethylamine (210 μL, 1.2 mmol), pivaloyl chloride (132 μL, 1.2 mmol), and MTBE (4.5 mL) for 15 min, followed by (*E*)-1,1,1-trifluoro-4-(4-fluorophenyl)but-3-en-2-one (87 mg, 0.4 mmol), (2*S*,3*R*)-HyperBTM (6 mg, 20.0 μmol), and *N*,*N*-diisopropylethylamine (71 μL, 0.4 mmol) for 16 h gave, after purification by Biotage Isolera 4 [SNAP KP-Sil 25 g, 36 mL min^–1^, petrol:Et_2_O (98:2 4 CV, 98:2 to 92:8 40 CV)], the title compound (77 mg, 70%) as a colorless oil; [α]_D_^20^ −102.9 (*c*, 0.1, CHCl_3_); Chiral HPLC analysis, Chiralcel OJ-H (99.5:0.5 hexane:IPA, flow rate 1.0 mL min^–1^, 254 nm, 30 °C), *t*_R_(minor): 15.3 min, *t*_R_(major): 25.6 min, 99:1 er; IR ν_max_ (film) 1850 (C=O), 1161 (C–O); ^1^H NMR (500 MHz, CDCl_3_) δ_H_ 7.49–7.45 (2H, m, ArC(2,6)*H*), 7.12–7.08 (2H, m, ArC(3,5)*H*), 7.01 (1H, d, *J* 16.0, ArC*H*=CH), 6.12 (1H, d, *J* 16.1, ArCH=C*H*), 4.10 (1H, q, *J* 7.7, C(3)*H*), 1.35 (3H, d, *J* 7.7, C(3)C*H*_3_). ^19^F NMR (471 MHz, CDCl_3_) δ_F_ −79.7 (C*F*_3_), −111.4 (Ar*F*). ^13^C{^1^H} NMR (126 MHz, CDCl_3_) δ_C_ 167.9 (*C*(2)), 163.3 (d, *J* 247.9, Ar*C*(4)), 136.5 (ArCH=*C*H), 130.7 (d, *J* 3.3, Ar*C*(1)), 128.8 (Ar*C*(2,6)H), 123.4 (q, *J* 282.0, *C*F_3_), 116.0 (d, *J* 21.8, Ar*C*(3,5)H), 114.7 (Ar*C*H=CH), 78.2 (q, *J* 33.2, *C*(4)), 52.0 (*C*(3)), 9.7 (C*C*H_3_). HRMS (ESI+) *m*/*z* [M + OH]^−^ calcd for C_13_H_11_F_4_O_3_ 291.0650, found 291.0650.

##### (3*S*,4*R*)-4-((*E*)-4-Chlorostyryl)-3-methyl-4-(trifluoromethyl)oxetan-2-one **(17)**

Following General Procedure D, 2-(trimethylsilyl)propanoic acid (117 mg, 0.8 mmol), *N*,*N*-diisopropylethylamine (210 μL, 1.2 mmol), pivaloyl chloride (132 μL, 1.2 mmol), and MTBE (4.5 mL) for 15 min, followed by (*E*)-4-(4-chlorophenyl)-1,1,1-trifluorobut-3-en-2-one (94 mg, 0.4 mmol), (2*S*,3*R*)-HyperBTM (6 mg, 20.0 μmol), and *N*,*N*-diisopropylethylamine (71 μL, 0.4 mmol) for 16 h gave, after purification by Biotage Isolera 4 [SNAP KP-Sil 25 g, 36 mL min^–1^, petrol:Et_2_O (98:2 4 CV, 98:2 to 92:8 40 CV)], the title compound (88 mg, 76%) as a colorless solid; mp 70–72 °C; [α]_D_^20^ −108.7 (*c*, 0.3, CHCl_3_); Chiral HPLC analysis, Chiralcel OJ-H (99.5:0.5 hexane:IPA, flow rate 1.0 mL min^–1^, 254 nm, 30 °C), *t*_R_ (minor): 22.4 min, *t*_R_ (major): 34.0 min, 99:1 er; IR ν_max_ (film) 1840 (C=O), 1159 (C–O); ^1^H NMR (500 MHz, CDCl_3_) δ_H_ 7.44–7.36 (4H, m, Ar*H*), 7.01 (1H, d, *J* 16.1, ArC*H*=CH), 6.18 (1H, d, *J* 16.1, ArCH=C*H*), 4.11 (1H, q, *J* 7.7, C(3)*H*), 1.35 (3H, d, *J* 7.7, C(3)C*H*_3_). ^19^F NMR (376 MHz, CDCl_3_) δ_F_ −79.6 (C*F*_3_). ^13^C{^1^H} NMR (126 MHz, CDCl_3_) δ_C_ 167.8 (*C*(2)), 136.4 (Ar*C*H=CH), 135.2 (Ar*C*(4)), 133.0 (Ar*C*(1)), 129.1 (Ar*C*(3,5)H), 128.3 (Ar*C*(2,6)H), 123.4 (q, *J* 280.5, *C*F_3_), 115.6 (ArCH=*C*H), 78.2 (q, *J* 33.5, *C*(4)), 52.1 (*C*(3)), 9.7 (C*C*H_3_). HRMS (ESI+) *m*/*z* [M + OH]^−^ calcd for C_13_H_11_ClF_3_O_3_ 307.0354, found 307.0359.

##### (3*S*,4*R*)-4-((*E*)-4-Bromostyryl)-3-methyl-4-(trifluoromethyl)oxetan-2-one **(18)**

Following General Procedure D, 2-(trimethylsilyl)propanoic acid (117 mg, 0.8 mmol), *N*,*N*-diisopropylethylamine (210 μL, 1.2 mmol), pivaloyl chloride (132 μL, 1.2 mmol), and MTBE (4.5 mL) for 15 min, followed by (*E*)-4-(4-bromophenyl)-1,1,1-trifluorobut-3-en-2-one (112 mg, 0.4 mmol), (2*S*,3*R*)-HyperBTM (6 mg, 20.0 μmol), and *N*,*N*-diisopropylethylamine (71 μL, 0.4 mmol) for 16 h gave, after purification by Biotage Isolera 4 [SNAP KP-Sil 25 g, 36 mL min^–1^, petrol:Et_2_O (98:2 4 CV, 98:2 to 92:8 40 CV)], the title compound (93 mg, 69%) as a white solid; mp 74–76 °C; [α]_D_^20^ −107.3 (*c*, 0.2, CHCl_3_); Chiral HPLC analysis, Chiralcel OJ-H (99.5:0.5 hexane:IPA, flow rate 1.0 mL min^–1^, 254 nm, 30 °C), *t*_R_ (minor): 28.6 min, *t*_R_ (major): 42.1 min, >99:1 er; IR ν_max_ (film) 1836 (C=O), 1157 (C–O); ^1^H NMR (400 MHz, CDCl_3_) δ_H_ 7.56–7.52 (2H, m, ArC(3,5)*H*), 7.37–7.33 (2H, m, ArC(2,6)*H*), 6.99 (1H, d, *J* 16.0, ArC*H*=CH), 6.20 (1H, d, *J* 16.0, ArCH=C*H*), 4.11 (1H, q, *J* 7.7, C(3)*H*), 1.35 (3H, d, *J* 7.7, C(3)C*H*_3_). ^19^F NMR (376 MHz, CDCl_3_) δ_F_ −79.6 (C*F*_3_). ^13^C{^1^H} NMR (126 MHz, CDCl_3_) δ_C_ 167.8 (*C*(2)), 136.5 (Ar*C*H=CH), 133.4 (Ar*C*(4)), 132.1 (Ar*C*(3,5)H), 128.6 (Ar*C*(2,6)H), 123.4 (Ar*C*(1)), 123.3 (q, *J* 280.5, *C*F_3_), 115.8 (ArCH=*C*H), 78.2 (q, *J* 33.6, *C*(4)), 52.1 (*C*(3)), 9.7 (C*C*H_3_). HRMS (ESI+) *m*/*z* [M + OH]^−^ calcd for C_13_H_11_BrF_3_O_3_ 350.9849, found 350.9851.

##### (3*S*,4*R*)-3-Methyl-4-(trifluoromethyl)-4-((*E*)-4-(trifluoromethyl)styryl)oxetan-2-one **(19)**

Following General Procedure D, 2-(trimethylsilyl)propanoic acid (73 mg, 0.5 mmol), *N*,*N*-diisopropylethylamine (132 μL, 0.75 mmol), pivaloyl chloride (83 μL, 0.75 mmol), and MTBE (3 mL) for 15 min, followed by (*E*)-1,1,1-trifluoro-4-(4-(trifluoromethyl)phenyl)but-3-en-2-one (67 mg, 0.25 mmol), (2*S*,3*R*)-HyperBTM (3.75 mg, 12.6 μmol), and *N*,*N*-diisopropylethylamine (45 μL, 0.25 mmol) for 16 h gave, after purification by Biotage Isolera 4 [SNAP KP-Sil 25 g, 36 mL min^–1^, petrol:Et_2_O (98:2 4 CV, 98:2 to 92:8 40 CV)], the title compound (51 mg, 62%) as a pale yellow solid; mp 48–50 °C; [α]_D_^20^ −86.0 (*c*, 0.6, CHCl_3_); Chiral HPLC analysis, Chiralcel OJ-H (99.5:0.5 hexane:IPA, flow rate 1.0 mL min^–1^, 254 nm, 30 °C), *t*_R_ (minor): 18.1 min, *t*_R_ (major): 20.7 min, 99:1 er; IR ν_max_ (film) 1852 (C=O), 1163 (C–O); ^1^H NMR (400 MHz, CDCl_3_) δ_H_ 7.67 (2H, d, *J* 8.2, ArC(3,5)*H*), 7.59 (2H, d, *J* 8.4, C(2,6)*H*), 7.10 (1H, d, *J* 16.0, ArC*H*=CH), 6.31 (1H, d, *J* 16.0, ArCH=C*H*), 4.14 (1H, q, *J* 7.7, C(3)*H*), 1.37 (3H, d, *J* 7.7, C(3)C*H*_3_). ^19^F NMR (376 MHz, CDCl_3_) δ_F_ −62.8 (ArC*F*_3_), −79.5 (C*F*_3_). ^13^C{^1^H} NMR (126 MHz, CDCl_3_) δ_C_ 167.6 (*C*(2)), 137.8 (Ar*C*(1)), 136.3 (ArCH=*C*H), 131.1 (q, *J* 32.6, Ar*C*(4)), 127.4 (Ar*C*(2,6)H), 125.9 (q, *J* 3.5, Ar*C*(3,5)H), 124.1 (q, *J* 271.6, Ar*C*F_3_), 123.3 (q, *J* 282.0, *C*F_3_), 117.8 (Ar*C*H=CH), 78.2 (q, *J* 33.4, *C*(4)), 52.2 (*C*(3)), 9.7 (C*C*H_3_). HRMS (ESI+) *m*/*z* [M + OH]^−^ calcd for C_14_H_11_F_6_O_3_ 341.0618, found 341.0617.

##### (3*S*,4*R*)-4-((*E*)-3-Methoxystyryl)-3-methyl-4-(trifluoromethyl)oxetan-2-one **(20)**

Following General Procedure D, 2-(trimethylsilyl)propanoic acid (117 mg, 0.8 mmol), *N*,*N*-diisopropylethylamine (210 μL, 1.2 mmol), pivaloyl chloride (132 μL, 1.2 mmol), and MTBE (4.5 mL) for 15 min, followed by (*E*)-1,1,1-trifluoro-4-(3-methoxyphenyl)but-3-en-2-one (92 mg, 0.4 mmol), (2*S*,3*R*)-HyperBTM (6 mg, 20.0 μmol), and *N*,*N*-diisopropylethylamine (71 μL, 0.4 mmol) for 16 h gave, after purification by Biotage Isolera 4 [SNAP KP-Sil 25 g, 36 mL min^–1^, petrol:Et_2_O (98:2 4 CV, 98:2 to 92:8 40 CV)], the title compound (58 mg, 51%) as a colorless oil; [α]_D_^20^ −90.5 (*c*, 0.3, CHCl_3_); Chiral HPLC analysis, Chiralcel OJ-H (99.5:0.5 hexane:IPA, flow rate 1.0 mL min^–1^, 254 nm, 30 °C), *t*_R_ (minor): 21.5 min, *t*_R_ (major): 33.8 min, >99:1 er; IR ν_max_ (film) 1850 (C=O), 1163 (C–O); ^1^H NMR (400 MHz, CDCl_3_) δ_H_ 7.33 (1H, t, *J* 7.9, ArC(5)*H*), 7.08 (1H, d, *J* 7.6, ArC(6)*H*), 7.02 (1H, d, *J* 16.0, ArC*H*=CH), 7.00 (1H, m, ArC(2)*H*), 6.92 (1H, dd, *J* 8.3, 2.4 ArC(4)*H*), 6.19 (1H, d, *J* 16.0, ArCH=C*H*), 4.10 (1H, q, *J* 7.8, C(3)*H*), 3.87 (3H, s, O*C*H_3_), 1.35 (3H, d, *J* 7.7, C(3)C*H*_3_). ^19^F NMR (376 MHz, CDCl_3_) δ_F_ −79.7 (C*F*_3_). ^13^C{^1^H} NMR (126 MHz, CDCl_3_) δ_C_ 168.0 (*C*(2)), 160.0 (Ar*C*(3)), 137.6 (ArCH=*C*H), 135.9 (Ar*C*(1)), 129.9 (Ar*C*(5)H), 123.4 (q, *J* 280.5, *C*F_3_), 119.6 (Ar*C*H=CH), 115.3 (Ar*C*(6)H), 114.9 (Ar*C*(4)H), 112.5 (Ar*C*(2)H), 78.2 (q, *J* 33.3, *C*(4)), 55.4 (O*C*H_3_), 52.1 (*C*(3)), 9.7 (C*C*H_3_). HRMS (ESI+) *m*/*z* [M + OH]^−^ calcd for C_14_H_14_F_3_O_4_ 303.0850, found 303.0846.

##### (3*S*,4*R*)-3-Methyl-4-((*E*)-3-methylstyryl)-4-(trifluoromethyl)oxetan-2-one **(21)**

Following General Procedure D, 2-(trimethylsilyl)propanoic acid (117 mg, 0.8 mmol), *N*,*N*-diisopropylethylamine (210 μL, 1.2 mmol), pivaloyl chloride (132 μL, 1.2 mmol), and MTBE (4.5 mL) for 15 min, followed by (*E*)-1,1,1-trifluoro-4-(m-tolyl)but-3-en-2-one (86 mg, 0.4 mmol), (2*S*,3*R*)-HyperBTM (6 mg, 20.0 μmol), and *N*,*N*-diisopropylethylamine (71 μL, 0.4 mmol) for 16 h gave, after purification by Biotage Isolera 4 [SNAP KP-Sil 25 g, 36 mL min^–1^, petrol:Et_2_O (98:2 4 CV, 98:2 to 92:8 40 CV)], the title compound (71 mg, 66%) as a white solid; mp 34–35 °C; [α]_D_^20^ −118.1 (*c*, 0.2, CHCl_3_); Chiral HPLC analysis, Chiralcel OJ-H (99.5:0.5 hexane:IPA, flow rate 1.0 mL min^–1^, 254 nm, 30 °C), *t*_R_ (minor): 10.2 min, *t*_R_ (major): 15.2 min, >99:1 er; IR ν_max_ (film) 1852 (C=O), 1161 (C–O); ^1^H NMR (400 MHz, CDCl_3_) δ_H_ 7.31–7.29 (3H, m, ArC(2,5,6)*H*), 7.21–7.19 (1H, m, ArC(4)*H*), 7.02 (1H, d, *J* 16.0, ArC*H*=CH), 6.19 (1H, d, *J* 16.0, ArCH=C*H*), 4.09 (1H, q, *J* 7.7, C(3)*H*), 3.40 (3H, s, Ar*C*H_3_), 1.35 (3H, d, *J* 7.7, C(3)C*H*_3_). ^19^F NMR (376 MHz, CDCl_3_) δ_F_ −79.7 (C*F*_3_). ^13^C{^1^H} NMR (126 MHz, CDCl_3_) δ_C_ 168.1 (*C*(2)), 138.6 (Ar*C*(1)), 137.7 (ArCH=*C*H), 134.5 (Ar*C*(3)), 130.1 (Ar*C*(5)H), 128.8 (Ar*C*(4)H), 127.7 (Ar*C*H=CH), 124.3 (Ar*C*(2)H), 123.4 (q, *J* 281.2, *C*F_3_), 114.7 (Ar*C*(6)H), 78.3 (q, *J* 33.5, *C*(4)), 52.0 (*C*(3)), 21.4 (Ar*C*H_3_), 9.7 (C*C*H_3_). HRMS (ESI+) *m*/*z* [M + OH]^−^ calcd for C_14_H_14_F_3_O_3_ 287.0901, found 287.0901.

##### (3*S*,4*R*)-4-((*E*)-3-Bromostyryl)-3-methyl-4-(trifluoromethyl)oxetan-2-one **(22)**

Following General Procedure D, 2-(trimethylsilyl)propanoic acid (117 mg, 0.8 mmol), *N*,*N*-diisopropylethylamine (210 μL, 1.2 mmol), pivaloyl chloride (132 μL, 1.2 mmol), and MTBE (4.5 mL) for 15 min, followed by (*E*)-4-(3-bromophenyl)-1,1,1-trifluorobut-3-en-2-one (112 mg, 0.4 mmol), (2*S*,3*R*)-HyperBTM (6 mg, 20.0 μmol), and *N*,*N*-diisopropylethylamine (71 μL, 0.4 mmol) for 16 h gave, after purification by Biotage Isolera 4 [SNAP KP-Sil 25 g, 36 mL min^–1^, petrol:Et_2_O (98:2 4 CV, 98:2 to 92:8 40 CV)], the title compound (84 mg, 63%) as a colorless oil; [α]_D_^20^ −95.3 (*c*, 0.5, CHCl_3_); Chiral HPLC analysis, Chiralcel OJ-H (99.5:0.5 hexane:IPA, flow rate 1.0 mL min^–1^, 254 nm, 30 °C), *t*_R_ (minor): 24.2 min, *t*_R_ (major): 43.1 min, 99:1 er; IR ν_max_ (film) 1850 (C=O), 1163 (C–O); ^1^H NMR (500 MHz, CDCl_3_) δ_H_ 7.65–7.64 (1H, m, ArC(6)*H*), 7.52–7.49 (1H, m, ArC(2)*H*), 7.40–7.38 (1H, m, ArC(4)*H*), 7.30–7.26 (1H, m, C(5)*H*), 6.99 (1H, d, *J* 16.0, ArC*H*=CH), 6.21 (1H, d, *J* 16.0, ArCH=C*H*), 4.12 (1H, q, *J* 7.7, C(3)*H*), 1.36 (3H, d, *J* 7.8, C(3)C*H*_3_). ^19^F NMR (376 MHz, CDCl_3_) δ_F_ −79.6 (C*F*_3_). ^13^C{^1^H} NMR (126 MHz, CDCl_3_) δ_C_ 167.7 (*C*(2)), 136.6 (Ar*C*(1)), 136.3 (ArCH=*C*H), 132.2 (Ar*C*(4)H), 130.4 (Ar*C*(2)H), 129.7 (Ar*C*(5)H), 126.0 (Ar*C*(6)H), 123.3 (q, *J* 280.8, *C*F_3_), 123.1 (Ar*C*(3)), 116.7 (Ar*C*H=CH), 78.2 (q, *J* 33.5, *C*(4)), 52.2 (*C*(3)), 9.7 (C*C*H_3_). HRMS (ESI+) *m*/*z* [M + OH]^−^ calcd for C_13_H_11_BrF_3_O_3_ 350.9849, found 350.9846.

##### (3*S*,4*R*)-3-Methyl-4-((*E*)-2-(naphthalen-2-yl)vinyl)-4-(trifluoromethyl)oxetan-2-one **(23)**

Following General Procedure D, 2-(trimethylsilyl)propanoic acid (117 mg, 0.8 mmol), *N*,*N*-diisopropylethylamine (210 μL, 1.2 mmol), pivaloyl chloride (132 μL, 1.2 mmol), and MTBE (4.5 mL) for 15 min, followed by (*E*)-1,1,1-trifluoro-4-(naphthalen-2-yl)but-3-en-2-one (100 mg, 0.4 mmol), (2*S*,3*R*)-HyperBTM (6 mg, 20.0 μmol), and *N*,*N*-diisopropylethylamine (71 μL, 0.4 mmol) for 16 h gave, after purification by Biotage Isolera 4 [SNAP KP-Sil 25 g, 36 mL min^–1^, petrol:Et_2_O (98:2 4 CV, 98:2 to 92:8 40 CV)], the title compound (66 mg, 54%) as a pale yellow solid; mp 102–104 °C; [α]_D_^20^ −73.0 (*c*, 0.2, CHCl_3_); Chiral HPLC analysis, Chiralcel OJ-H (99.5:0.5 hexane:IPA, flow rate 1.0 mL min^–1^, 254 nm, 30 °C), *t*_R_ (minor): 20.7 min, *t*_R_ (major): 40.4 min, >99:1 er; IR ν_max_ (film) 1846 (C=O), 1144 (C–O); ^1^H NMR (500 MHz, CDCl_3_) δ_H_ 7.89–7.85 (4H, m, ArC(1,4,6,9)*H*), 7.65 (1H, dd, *J* 8.8, 1.7, ArC(3)*H*), 7.55–7.53 (2H, m, ArC(7,8)*H*), 7.22 (1H, d, *J* 16.0, ArC*H*=CH), 6.32 (1H, d, *J* 16.0, ArCH=C*H*), 4.13 (1H, q, *J* 7.7, C(3)*H*), 1.39 (3H, d, *J* 7.7, C(3)C*H*_3_). ^19^F NMR (376 MHz, CDCl_3_) δ_F_ −79.6 (C*F*_3_). ^13^C{^1^H} NMR (126 MHz, CDCl_3_) δ_C_ 168.0 (*C*(2)), 137.7 (ArCH=*C*H), 133.7 (Ar*C*(4a)), 133.4 (Ar*C*(2)), 131.9 (Ar*C*(5)H), 128.7 (Ar*C*(4)H), 128.3 (Ar*C*(8a)), 128.2 (Ar*C*(1)H), 127.8 (Ar*C*(6)H), 126.9 (Ar*C*H=CH), 126.8 (Ar*C*(8)H), 123.5 (q, *J* 281.8, *C*F_3_), 123.2 (Ar*C*(7)H), 115.1 (Ar*C*(3)), 78.4 (q, *J* 33.7, *C*(4)), 52.1 (*C*(3)), 9.8 (C*C*H_3_). HRMS (ESI+) *m*/*z* [M + OH]^−^ calcd for C_17_H_14_F_3_O_3_ 323.0901, found 323.0900.

##### (3*S*,4*R*)-3-Methyl-4-((*E*)-2-(naphthalen-1-yl)vinyl)-4-(trifluoromethyl)oxetan-2-one **(24)**

Following General Procedure D, 2-(trimethylsilyl)propanoic acid (117 mg, 0.8 mmol), *N*,*N*-diisopropylethylamine (210 μL, 1.2 mmol), pivaloyl chloride (132 μL, 1.2 mmol), and MTBE (4.5 mL) for 15 min, followed by (*E*)-1,1,1-trifluoro-4-(naphthalen-1-yl)but-3-en-2-one (100 mg, 0.4 mmol), (2*S*,3*R*)-HyperBTM (6 mg, 20.0 μmol), and *N*,*N*-diisopropylethylamine (71 μL, 0.4 mmol) for 16 h gave, after purification by Biotage Isolera 4 [SNAP KP-Sil 25 g, 36 mL min^–1^, petrol:Et_2_O (98:2 4 CV, 98:2 to 92:8 40 CV)], the title compound (76 mg, 62%) as a white solid; mp 56–58 °C; [α]_D_^20^ −24.9 (*c*, 0.3, CHCl_3_); Chiral HPLC analysis, Chiralcel OJ-H (99.5:0.5 hexane:IPA, flow rate 1.0 mL min^–1^, 211 nm, 30 °C), *t*_R_ (minor): 23.3 min, *t*_R_ (major): 32.8 min, >99:1 er; IR ν_max_ (film) 1848 (C=O), 1159 (C–O); ^1^H NMR (500 MHz, CDCl_3_) δ_H_ 8.10 (1H, m, ArC(4)*H*), 7.93–7.90 (2H, m, ArC(5,8)*H*), 7.85 (1H, d, *J* 15.8, ArC*H*=CH), 7.66 (1H, dt, *J* 7.1, 0.9, ArC(2)*H*), 7.63–7.55 (2H, m, ArC(3,7)*H*), 7.53–7.50 (1H, m, ArC(6)*H*), 6.27 (1H, d, *J* 15.8, ArCH=C*H*), 4.16 (1H, q, *J* 7.7, C(3)*H*), 1.44 (3H, d, *J* 7.7, C(3)C*H*_3_). ^19^F NMR (376 MHz, CDCl_3_) δ_F_ −79.6 (C*F*_3_). ^13^C{^1^H} NMR (126 MHz, CDCl_3_) δ_C_ 168.0 (*C*(2)), 135.4 (Ar*C*H=CH), 133.6 (Ar*C*(1)), 132.5 (Ar*C*(4a)), 131.0 (Ar*C*(8a)), 129.6 (Ar*C*(5)H), 128.7 (Ar*C*(4)H), 126.8 (Ar*C*(3)H), 126.3 (Ar*C*(7)H), 125.5 (Ar*C*(6)H), 124.4 (Ar*C*(8)H), 123.47 (q, *J* 281.9, *C*F_3_), 123.45 (Ar*C*(2)H), 118.4 (ArCH=*C*H), 78.3 (q, *J* 32.9, *C*(4)), 52.1 (*C*(3)), 9.9 (C*C*H_3_). HRMS (ESI+) *m*/*z* [M + H]^+^ calcd for C_17_H_14_F_3_O_2_ 307.0940, found 307.0936.

##### (3*S*,4*R*)-4-((*E*)-2-Bromostyryl)-3-methyl-4-(trifluoromethyl)oxetan-2-one **(25)**

Following General Procedure D, 2-(trimethylsilyl)propanoic acid (117 mg, 0.8 mmol), *N*,*N*-diisopropylethylamine (210 μL, 1.2 mmol), pivaloyl chloride (132 μL, 1.2 mmol), and MTBE (4.5 mL) for 15 min, followed by (*E*)-4-(2-bromophenyl)-1,1,1-trifluorobut-3-en-2-one (111 mg, 0.4 mmol), (2*S*,3*R*)-HyperBTM (6 mg, 20.0 μmol), and *N*,*N*-diisopropylethylamine (71 μL, 0.4 mmol) for 16 h gave, after purification by Biotage Isolera 4 [SNAP KP-Sil 25 g, 36 mL min^–1^, petrol:Et_2_O (98:2 4 CV, 98:2 to 92:8 40 CV)], the title compound (80 mg, 60%) as a colorless oil; [α]_D_^20^ −29.6 (*c*, 0.3, CHCl_3_); Chiral HPLC analysis, Chiralcel OD-H (99.9:0.1 hexane:IPA, flow rate 1.0 mL min^–1^, 254 nm, 30 °C), *t*_R_ (minor): 12.0 min, *t*_R_ (major): 15.0 min, 99:1 er; IR ν_max_ (film) 1852 (C=O), 1165 (C–O); ^1^H NMR (400 MHz, CDCl_3_) δ_H_ 7.63 (1H, dd, *J* 8.0, 1.3, ArC(3)*H*), 7.55 (1H, dd, *J* 7.8, 1.7, ArC(5)*H*), 7.39 (1H, d, *J* 16.0, ArC*H*=CH), 7.36 (1H, td, *J* 7.5, 1.3, ArC(4)*H*), 7.24 (1H, td, *J* 7.8, 1.8, ArC(6)*H*), 6.15 (1H, d, *J* 16.0, ArCH=C*H*), 4.12 (1H, q, *J* 7.7, C(3)*H*), 1.39 (3H, d, *J* 7.7, C(3)C*H*_3_). ^19^F NMR (376 MHz, CDCl_3_) δ_F_ −79.5 (C*F*_3_). ^13^C{^1^H} NMR (126 MHz, CDCl_3_) δ_C_ 167.8 (*C*(2)), 136.9 (ArCH=*C*H), 135.0 (Ar*C*(1)), 133.3 (Ar*C*(3)H), 130.4 (Ar*C*(5)H), 127.7 (Ar*C*(6)H), 127.5 (Ar*C*(4)H), 124.1 (Ar*C*(2)), 123.2 (q, *J* 282.2, *C*F_3_), 118.4 (Ar*C*H=CH), 78.1 (q, *J* 33.6, *C*(4)), 52.1 (*C*(3)), 9.9 (C*C*H_3_). HRMS (ESI+) *m*/*z* [M + OH]^−^ calcd for C_13_H_11_F_3_BrO_3_ 350.9849, found 350.9848.

##### (3*S*,4*R*)-3-Methyl-4-((*E*)-2-methylstyryl)-4-(trifluoromethyl)oxetan-2-one **(26)**

Following General Procedure D, 2-(trimethylsilyl)propanoic acid (117 mg, 0.8 mmol), *N*,*N*-diisopropylethylamine (210 μL, 1.2 mmol), pivaloyl chloride (132 μL, 1.2 mmol), and MTBE (4.5 mL) for 15 min, followed by (*E*)-1,1,1-trifluoro-4-(o-tolyl)but-3-en-2-one (86 mg, 0.4 mmol), (2*S*,3*R*)-HyperBTM (6 mg, 20.0 μmol), and *N*,*N*-diisopropylethylamine (71 μL, 0.4 mmol) for 16 h gave, after purification by Biotage Isolera 4 [SNAP KP-Sil 25 g, 36 mL min^–1^, petrol:Et_2_O (98:2 4 CV, 98:2 to 92:8 40 CV)], the title compound (50 mg, 46%) as a colorless oil; [α]_D_^20^ −92.3 (*c*, 0.3, CHCl_3_); Chiral HPLC analysis, Chiralcel OJ-H (99.5:0.5 hexane:IPA, flow rate 1.0 mL min^–1^, 254 nm, 30 °C), *t*_R_ (minor): 9.6 min, *t*_R_ (major): 11.0 min, 98:2 er; IR ν_max_ (film) 1850 (C=O), 1165 (C–O); ^1^H NMR (400 MHz, CDCl_3_) δ_H_ 7.49–7.47 (1H, m, Ar*H*), 7.48 (1H, d, *J* 16.0, ArC*H*=CH), 7.28 (1H, d, *J* 16.0, ArC*H*=CH), 7.29–7.22 (3H, m, Ar*H*), 6.09 (1H, d, *J* 16.0, ArCH=C*H*), 4.11 (1H, q, *J* 7.8, C(3)*H*), 2.41 (3H, s, Ar*C*H_3_), 1.38 (3H, d, *J* 7.8, C(3)C*H*_3_). ^19^F NMR (376 MHz, CDCl_3_) δ_F_ −79.7 (C*F*_3_). ^13^C{^1^H} NMR (126 MHz, CDCl_3_) δ_C_ 168.1 (*C*(2)), 136.4 (Ar*C*(1)), 135.7 (ArCH=*C*H), 134.0 (Ar*C*(2)), 130.7 (Ar*C*(4)H), 129.2 (Ar*C*(3)H), 126.3 (Ar*C*(6)H), 125.9 (Ar*C*H=CH), 123.5 (q, *J* 281.3, *C*F_3_), 116.5 (Ar*C*(5)H), 78.3 (q, *J* 33.0, *C*(4)), 51.9 (*C*(3)), 19.8 (Ar*C*H_3_), 9.8 (C*C*H_3_). HRMS (ESI+) *m*/*z* [M + OH]^−^ calcd for C_14_H_14_F_3_O_3_ 287.0901, found 287.0903.

##### (3*S*,4*R*)-3-Ethyl-4-((*E*)-styryl)-4-(trifluoromethyl)oxetan-2-one **(27)**

Following General Procedure D, 2-(trimethylsilyl)butanoic acid (64 mg, 0.4 mmol), *N*,*N*-diisopropylethylamine (105 μL, 0.6 mmol), pivaloyl chloride (66 μL, 0.6 mmol), and MTBE (2 mL) for 15 min, followed by (*E*)-1,1,1-trifluoro-4-phenylbut-3-en-2-one (40 mg, 0.2 mmol), (2*S*,3*R*)-HyperBTM (3 mg, 10.0 μmol), and *N*,*N*-diisopropylethylamine (35 μL, 0.2 mmol) for 18 h gave, after purification by Biotage Isolera 4 [SNAP KP-Sil 10 g, 36 mL min^–1^, petrol:Et_2_O (98:2 4 CV, 98:2 to 94:6 30 CV)], the title compound (36 mg, 67%) as a colorless oil; [α]_D_^20^ −135.6 (*c*, 0.5, CHCl_3_); Chiral HPLC analysis, Chiralpak IB (99.5:0.5 hexane:IPA, flow rate 1.0 mL min^–1^, 254 nm, 30 °C), *t*_R_ (minor): 4.7 min, *t*_R_ (major): 5.4 min, 99:1 er; IR ν_max_ (film) 1846 (C=O), 1161 (C–O); ^1^H NMR (500 MHz, CDCl_3_) δ_H_ 7.49–7.36 (5H, m, Ar*H*), 7.06 (1H, d, *J* 16.0, CH=C*H*Ph), 6.24 (1H, d, *J* 16.0, C*H*=CHPh), 3.89 (1H, dd, *J* 8.8, 7.9, C(3)*H*), 1.90–1.81 (1H, m, C(3)CH_A_*H*_B_), 1.79–1.70 (1H, m, C(3)C*H*_A_H_B_), 1.09 (3H, t, *J* 7.5, C(3)CH_2_C*H*_3_). ^19^F NMR (471 MHz, CDCl_3_) δ_F_ −79.8 (m, C*F*_3_). ^13^C{^1^H} NMR (126 MHz, CDCl_3_) δ_C_ 167.5 (*C*O), 137.5 (ArCH=*C*H), 134.6 (Ph*C*(1)), 129.3 (Ph*C*(4)H), 128.9 (Ph*C*(3,5)H), 128.0 (Ar*C*H=CH), 127.1 (Ph*C*(2,6)H), 123.4 (q, *J* 282.1, *C*F_3_), 114.9 (Ar*C*H=CH), 78.3 (q, *J* 33.3, *C*CF_3_), 58.8 (C(3)), 18.7 (*C*H_2_CH_3_), 11.5 (CH_2_*C*H_3_). HRMS (ESI+) *m*/*z* [M + H]^+^ calcd for C_14_H_14_F_3_O_2_ 271.0940, found 271.0941.

##### (3*S*,4*R*)-3-Allyl-4-((*E*)-styryl)-4-(trifluoromethyl)oxetan-2-one **(28**)

Following General Procedure D, 2-(trimethylsilyl)pent-4-enoic acid (69 mg, 0.4 mmol), *N*,*N*-diisopropylethylamine (105 μL, 0.6 mmol), pivaloyl chloride (66 μL, 0.6 mmol), and MTBE (2 mL) for 15 min, followed by (*E*)-1,1,1-trifluoro-4-phenylbut-3-en-2-one (40 mg, 0.2 mmol), (2*S*, 3*R*)-HyperBTM (3 mg, 10.0 μmol), and *N*,*N*-diisopropylethylamine (35 μL, 0.2 mmol) for 16 h gave, after purification by Biotage Isolera 4 [SNAP KP-Sil 10 g, 36 mL min^–1^, petrol:Et_2_O (98:2 4 CV, 98:2 to 94:6 30 CV)], the title compound (34 mg, 60%) as a white solid; mp 54–56 °C; [α]_D_^20^ −111.2 (*c*, 0.2, CHCl_3_); Chiral HPLC analysis, Chiralcel OJ-H (99.8:0.2 hexane:IPA, flow rate 0.5 mL min^–1^, 254 nm, 30 °C), *t*_R_ (minor): 21.6 min, *t*_R_ (major): 25.3 min, >99:1 er; IR ν_max_ (film) 1856 (C=O), 1163 (C–O); ^1^H NMR (500 MHz, CDCl_3_) δ_H_ 7.48–7.38 (5H, m, Ar*H*), 7.05 (1H, d, *J* 16.0, CH=C*H*Ph), 6.24 (1H, d, *J* 16.1, C*H*=CHPh), 5.85–5.72 (1H, m, C*H*=CH_2_), 5.24–5.15 (2H, m, CH=C*H*_2_), 4.10 (1H, dd, *J* 10.1, 6.4, C(3)*H*), 2.66–2.43 (2H, m, C(3)C*H*_2_). ^19^F NMR (282 MHz, CDCl_3_) δ_F_ −79.6 (s, C*F*_3_). ^13^C{^1^H} NMR (126 MHz, CDCl_3_) δ_C_ 166.8 (*C*O), 137.4 (Ph*C*H=CH), 134.5 (Ph*C*(1)), 132.4 (Ph*C*(4)H), 129.3 (*C*H=CH_2_), 128.9 (Ph*C*(3,5)H), 127.1 (Ph*C*(2,6)H), 123.3 (q, *J* 281.8, *C*F_3_), 118.4 (CH=*C*H_2_), 114.9 (Ph*C*H=CH), 78.3 (q, *J* 33.2, *C*CF_3_), 56.3 (C(3)), 29.0 (C(3)*C*H_2_). HRMS (ESI+) *m*/*z* [M + OH]^−^ calcd for C_15_H_14_F_3_O_3_ 299.0901, found 299.0899.

##### (3*S*,4*R*)-3-(Prop-2-yn-1-yl)-4-((*E*)-styryl)-4-(trifluoromethyl)oxetan-2-one **(29)**

Following General Procedure D, 2-(trimethylsilyl)pent-4-ynoic acid (68 mg, 0.4 mmol), *N*,*N*-diisopropylethylamine (105 μL, 0.6 mmol), pivaloyl chloride (66 μL, 0.6 mmol), and MTBE (2 mL) for 15 min, followed by (*E*)-1,1,1-trifluoro-4-phenylbut-3-en-2-one (40 mg, 0.2 mmol), (2*S*, 3*R*)-HyperBTM (3 mg, 10.0 μmol), and *N*,*N*-diisopropylethylamine (35 μL, 0.2 mmol) for 16 h gave, after purification by Biotage Isolera 4 [SNAP KP-Sil 10 g, 36 mL min^–1^, petrol:Et_2_O (98:2 4 CV, 98:2 to 94:6 30 CV)], the title compound (31 mg, 55%) as a colorless solid; mp 36–38 °C; [α]_D_^20^ +19.4 (*c*, 0.5, CHCl_3_); Chiral HPLC analysis, Chiralcel OD-H (99.5:0.5 hexane:IPA, flow rate 1.0 mL min^–1^, 254 nm, 30 °C), *t*_R_ (minor): 7.4 min, *t*_R_ (major): 10.0 min, 98:2 er; IR ν_max_ (film) 3300 (C≡C), 1856 (C=O), 1161 (C–O); ^1^H NMR (500 MHz, CDCl_3_) δ_H_ 7.52–7.37 (5H, m, Ar*H*), 7.09 (1H, d, *J* 16.0, CH=C*H*Ph), 6.42 (1H, d, *J* 16.1, C*H*=CHPh), 4.22 (1H, dd, *J* 11.2, 5.2, C≡C*H*), 2.78–2.55 (2H, m, C(3)C*H*_2_), 2.18 (1H, t, *J* 2.7, C(3)*H*). ^19^F NMR (282 MHz, CDCl_3_) δ_F_ −79.7 (s, C*F*_3_). ^13^C{^1^H} NMR (126 MHz, CDCl_3_) δ_C_ 165.2 (*C*O), 137.9 (Ph*C*H=CH), 134.5 (Ph*C*(1)), 129.5 (Ph*C*(4)H), 128.9 (Ph*C*(3,5)H), 127.2 (Ph*C*(2,6)H), 123.5 (q, *J* 282.6, *C*F_3_), 114.3 (Ph*C*H=CH), 78.3 (q, *J* 33.2, *C*CF_3_), 71.6 (*C*≡CH), 56.0 (C≡*C*H, C(3)), 15.1 (C(3)*C*H_2_). HRMS (ESI+) *m*/*z* [M + OH]^−^ calcd for C_15_H_12_F_3_O_3_ 297.0744, found 297.0743.

##### (3*S*,4*R*)-3-Benzyl-4-((*E*)-styryl)-4-(trifluoromethyl)oxetan-2-one **(30)**

Following General Procedure D, 3-phenyl-2-(trimethylsilyl)propanoic acid (112 mg, 0.5 mmol), *N*,*N*-diisopropylethylamine (132 μL, 0.75 mmol), pivaloyl chloride (92 μL, 0.75 mmol), and MTBE (3 mL) for 15 min, followed by (*E*)-1,1,1-trifluoro-4-phenylbut-3-en-2-one (50 mg, 0.25 mmol), (2*S*,3*R*)-HyperBTM (4 mg, 12.5 μmol), and *N*,*N*-diisopropylethylamine (44 μL, 0.25 mmol) for 20 h gave, after purification by Biotage Isolera 4 [SNAP KP-Sil 25 g, 36 mL min^–1^, petrol:Et_2_O (98:2 4 CV, 98:2 to 95:5 40 CV)], the title compound (42 mg, 58%) as a colorless oil; [α]_D_^20^ +25.9 (*c* 0.8, CHCl_3_); Chiral HPLC analysis, Chiralpak IB (99.5:0.5 hexane:IPA, flow rate 0.7 mL min^–1^, 254 nm, 30 °C), *t*_R_ (minor): 16.7 min, *t*_R_ (major):22.4 min, 99:1 er; IR ν_max_ (film) 1854 (C=O), 1161 (C–O); ^1^H NMR (500 MHz, CDCl_3_) δ_H_ 7.48–7.18 (10H, m, Ph*H*), 7.08 (1H, d, *J* 16.0, CH=C*H*Ph), 6.05 (1H, d, *J* 16.0, C*H*=CHPh), 4.39 (1H, dd, *J* 10.0, 6.8, C(3)*H*), 3.23 (1H, dd, *J* 15.0, 6.7, C(3)H_A_*H*_B_), 2.98 (1H, dd, *J* 15.0, 10.0, C(3)C*H*_A_H_B_). ^19^F NMR (376 MHz, CDCl_3_) δ_F_ −79.6 (C*F*_3_). ^13^C{^1^H} NMR (126 MHz, CDCl_3_) δ_C_ 163.6 (*C*(2)), 137.1 (*C*H=CHPh), 135.8 (CH=CHPh*C*(1)), 134.4 (CH=CHPh*C*(1)), 129.4 (CH=CHPh*C*(4)H), 129.0 (C(3)CH_2_Ph*C*(3,5)H), CH=CHPh*C*(2,6)H), 128.4 (CH=CHPh*C*(3,5)H), 127.4 (C(3)CH_2_Ph*C*(4)H), 127.2 (C(3)CH_2_Ph*C*(2,6)H), 123.2 (q, *J* 283.1, *C*F_3_), 115.1 (CH=*C*HPh), 78.6 (q, *J* 33.5, *C*(4)), 57.9 (*C*(3)H), 30.9 (C(3)*C*H_2_). HRMS (ESI+) *m*/*z* [M + Na]^+^ calcd for C_19_H_15_F_3_NaO_2_ 355.0916, found 355.0922.

##### (3*S*,4*R*)-3-(Naphthalen-2-ylmethyl)-4-((*E*)-styryl)-4-(trifluoromethyl)oxetan-2-one **(31**)

Following General Procedure D, 3-(naphthalen-2-yl)-2-(trimethylsilyl)propanoic acid (114 mg, 0.4 mmol), *N*,*N*-diisopropylethylamine (105 μL, 0.6 mmol), pivaloyl chloride (66 μL, 0.6 mmol), and MTBE (2 mL) for 15 min, followed by (*E*)-1,1,1-trifluoro-4-phenylbut-3-en-2-one (40 mg, 0.2 mmol), (2*S*, 3*R*)-HyperBTM (3 mg, 10.0 μmol), and *N*,*N*-diisopropylethylamine (35 μL, 0.2 mmol) for 16 h gave, after purification by Biotage Isolera 4 [SNAP KP-Sil 10 g, 36 mL min^–1^, petrol:Et_2_O (98:2 4 CV, 98:2 to 94:6 30 CV)], the title compound (50 mg, 52%) as a colorless oil; [α]_D_^20^ +107.5 (*c*, 0.3, CHCl_3_); Chiral HPLC analysis, Chiralcel OD-H (95:5 hexane:IPA, flow rate 1.0 mL min^–1^, 254 nm, 30 °C), *t*_R_ (minor): 19.4 min, *t*_R_ (major): 34.5 min, 95:5 er; IR ν_max_ (film) 1852 (C=O), 1163 (C–O); ^1^H NMR (500 MHz, CDCl_3_) δ_H_ 7.88–7.86 (2H, m, ArC*H*), 7.79–7.78 (1H, m, ArC*H*), 7.62 (1H, s, Ar*H*), 7.55–7.51 (2H, m, ArC*H*), 7.45–7.40 (5H, m, ArC*H*), 7.33–7.31 (1H, m, CH=CHPh(4)*H*), 7.11 (1H, d, *J* 16.0, CH=C*H*Ph), 6.05 (1H, d, *J* 16.0, C*H*=CHPh), 4.51 (1H, dd, *J* 10.5, 6.6, C(3)*H*), 3.40 (1H, dd, *J* 15.0, 6.4, C(3)CH_A_*H*_B_), 3.16 (1H, dd, *J* 15.0, 10.4, C(3)C*H*_A_H_B_). ^19^F NMR (282 MHz, CDCl_3_) δ_F_ −79.6 (s, C*F*_3_). ^13^C{^1^H} NMR (126 MHz, CDCl_3_) δ_C_ 166.8 (*C*O), 137.1 (ArCH=*C*H), 134.4 (Ar*C*(2)), 133.4 (Ph*C*(1)), 133.1 (Ar*C*(8a)), 132.6 (Ar*C*(4a)), 129.4 (Ar*C*(1)H), 129.0 (Ph*C*(3,5)H), 128.9 (Ar*C*(3)H), 127.8 (Ar*C*(8)H), 127.6 (Ph*C*(4)H), 127.25 (Ar*C*(4)H), 127.20 (Ph*C*(2,6)H), 126.6 (Ar*C*(5)H), 126.1 (Ar*C*(6,7)H), 123.3 (q, *J* 281.8, *C*F_3_), 115.2 (Ar*C*H=CH), 78.7 (q, *J* 33.2, *C*CF_3_), 57.8 (C(3)), 31.2 (*C*H_2_Ar). HRMS (ESI+) *m*/*z* [M + Na]^+^ calcd for C_23_H_17_F_3_NaO_2_ 405.1073, found 405.1067.

##### (3*S*,4*S*)-3-Methyl-4-((*E*)-1-phenylprop-1-en-2-yl)-4-(trifluoromethyl)oxetan-2-one **(32**)

Following General Procedure D, 2-(trimethylsilyl)propanoic acid (117 mg, 0.8 mmol), *N*,*N*-diisopropylethylamine (210 μL, 1.2 mmol), pivaloyl chloride (132 μL, 1.2 mmol), and MTBE (4.5 mL) for 15 min, followed by (*E*)-1,1,1-trifluoro-3-methyl-4-phenylbut-3-en-2-one (86 mg, 0.4 mmol), (2*S*,3*R*)-HyperBTM (6 mg, 20.0 μmol), and *N*,*N*-diisopropylethylamine (71 μL, 0.4 mmol) for 16 h gave, after purification by Biotage Isolera 4 [SNAP KP-Sil 25 g, 36 mL min^–1^, petrol:Et_2_O (98:2 4 CV, 98:2 to 96:4 40 CV)], the title compound (63 mg, 58%) as a colorless oil; [α]_D_^20^ +32.3 (*c*, 1.1, CHCl_3_); Chiral HPLC analysis, Chiralcel OJ-H (99.8:0.2 hexane:IPA, flow rate 1.0 mL min^–1^, 254 nm, 30 °C), *t*_R_ (minor): 13.1 min, *t*_R_ (major): 17.4 min, >99:1 er; IR ν_max_ (film) 1846 (C=O), 1171 (C–O); ^1^H NMR (500 MHz, CDCl_3_) δ_H_ 7.44–7.40 (2H, m, PhC(3,5)*H*), 7.36–7.32 (3H, m, PHC(2,4,6)*H*), 6.94 (1H, s, ArC*H*), 4.07 (1H, q, *J* 7.8, C(3)*H*), 2.00 (3H, t, *J* 1.4, CC*H*_3_), 1.41 (3H, d, *J* 7.7, C(3)*H*_A_H_B_). ^19^F NMR (376 MHz, CDCl_3_) δ_F_ −76.5 (C*F*_3_). ^13^C{^1^H} NMR (126 MHz, CDCl_3_) δ_C_ 168.3 (*C*(2)), 135.5 (PhCH=*C*), 132.6 (Ph*C*H=C), 129.2 (Ph*C*(3,5)H), 128.4 (Ph*C*(2,6)H), 127.8 (Ph*C*(4)H), 125.4 (PH*C*(1)), 123.8 (q, *J* 283.6, *C*F_3_), 80.7 (t, *J* 31.6, *C*(4)), 51.5 (*C*(3)), 15.2 (C(3)*C*H_3_), 9.7 (C*C*H_3_). HRMS (ESI+) *m*/*z* [M + H]^+^ calcd for C_14_H_14_F_3_O_2_ 271.0940, found 271.0936.

##### (3*S*,4*S*)-4-((*E*)-1-(4-Bromophenyl)prop-1-en-2-yl)-3-methyl-4-(trifluoromethyl)oxetan-2-one **(33)**

Following General Procedure D, 2-(trimethylsilyl)propanoic acid (117 mg, 0.8 mmol), *N*,*N*-diisopropylethylamine (210 μL, 1.2 mmol), pivaloyl chloride (132 μL, 1.2 mmol), and MTBE (4.5 mL) for 15 min, followed by (*E*)-4-(4-bromophenyl)-1,1,1-trifluoro-3-methylbut-3-en-2-one (117 mg, 0.4 mmol), (2*S*,3*R*)-HyperBTM (6 mg, 20.0 μmol), and *N*,*N*-diisopropylethylamine (71 μL, 0.4 mmol) for 16 h gave, after purification by Biotage Isolera 4 [SNAP KP-Sil 25 g, 36 mL min^–1^, petrol:Et_2_O (98:2 4 CV, 98:2 to 92:8 40 CV)], the title compound (112 mg, 80%) as a white solid; mp 38–40 °C; [α]_D_^20^ −2.1 (*c*, 0.3, CHCl_3_); Chiral HPLC analysis, Chiralcel OJ-H (99.5:0.5 hexane:IPA, flow rate 1.0 mL min^–1^, 254 nm, 30 °C), *t*_R_ (minor): 24.6 min, *t*_R_ (major): 39.4 min, >99:1 er; IR ν_max_ (film) 1848 (C=O), 1173 (C–O); ^1^H NMR (500 MHz, CDCl_3_) δ_H_ 7.56–7.53 (2H, m, ArC(3,5)*H*), 7.23–7.20 (2H, m, ArC(2,6)*H*), 6.87 (1H, s, C=C*H*), 4.07 (1H, q, *J* 7.7, C(3)*H*), 1.97 (3H, s, CH=CC*H*_3_), 1.40 (3H, d, *J* 7.7, C(3)C*H*_3_). ^19^F NMR (376 MHz, CDCl_3_) δ_F_ −76.4 (C*F*_3_). ^13^C{^1^H} NMR (126 MHz, CDCl_3_) δ_C_ 168.0 (*C*(2)), 134.3 (ArCH=*C*), 131.6 (Ar*C*(3,5)H), 131.5 (Ar*C*H=C), 130.8 (Ar*C*(2,6)H), 126.4 (Ar*C*(4)), 123.7 (q, *J* 282.5, *C*F_3_), 121.9 (Ar*C*(1)), 80.7 (q, *J* 31.3, *C*(4)), 51.5 (*C*(3)), 15.2 (C(3)*C*H_3_), 9.7 (C*C*H_3_). HRMS (ESI+) *m*/*z* [M + OH]^−^ calcd for C_14_H_13_BrF_3_O_3_ 365.0006, found 365.0010.

##### (3*S*,4*S*)-4-((*E*)-1-(4-Methoxyphenyl)prop-1-en-2-yl)-3-methyl-4-(trifluoromethyl)oxetan-2-one **(34)**

Following General Procedure D, 2-(trimethylsilyl)propanoic acid (117 mg, 0.8 mmol), *N*,*N*-diisopropylethylamine (210 μL, 1.2 mmol), pivaloyl chloride (132 μL, 1.2 mmol), and MTBE (4.5 mL) for 15 min, followed by (*E*)-1,1,1-trifluoro-4-(4-methoxyphenyl)-3-methylbut-3-en-2-one (98 mg, 0.4 mmol), (2*S*,3*R*)-HyperBTM (6 mg, 20.0 μmol), and *N*,*N*-diisopropylethylamine (71 μL, 0.4 mmol) for 16 h gave, after purification by Biotage Isolera 4 [SNAP KP-Sil 25 g, 36 mL min^–1^, petrol:Et_2_O (98:2 4 CV, 98:2 to 92:8 40 CV)], the title compound (46 mg, 38%) as a white solid; mp 62–64 °C; [α]_D_^20^ +13.6 (*c*, 0.3, CHCl_3_); Chiral HPLC analysis, Chiralpak AS-H (99.5:0.5 hexane:IPA, flow rate 1.0 mL min^–1^, 254 nm, 30 °C), *t*_R_ (minor): 6.0 min, *t*_R_ (major): 7.8 min, 99:1 er; IR ν_max_ (film) 1844 (C=O), 1169 (C–O); ^1^H NMR (500 MHz, CDCl_3_) δ_H_ 7.33–7.30 (2H, m, ArC(2,6)*H*), 6.96–6.93 (2H, m, ArC(3,5)*H*), 6.85 (1H, s, C=C*H*), 4.05 (1H, q, *J* 7.7, C(3)*H*), 3.86 (3H, s, OC*H*_3_), 2.00 (3H, t, *J* 1.5, CH=CC*H*_3_), 1.39 (3H, d, *J* 7.7, C(3)C*H*_3_). ^19^F NMR (376 MHz, CDCl_3_) δ_F_ −76.6 (C*F*_3_). ^13^C{^1^H} NMR (126 MHz, CDCl_3_) δ_C_ 168.4 (*C*(2)), 159.1 (Ar*C*(4)), 132.0 (Ar*C*H=C), 130.7 (Ar*C*(2,6)H), 128.0 (ArCH=*C*), 123.9 (q, *J* 283.9, *C*F_3_), 123.3 (Ar*C*(1)), 113.8 (Ar*C*(3,5)H), 80.9 (q, *J* 31.6, *C*(4)), 55.3 (O*C*H_3_), 51.5 (*C*(3)), 15.3 (C(3)*C*H_3_), 9.8 (C*C*H_3_). HRMS (ESI+) *m*/*z* [M + H]^+^ calcd for C_15_H_16_F_3_O_3_ 301.1046, found 301.1042.

##### (3*S*,4*S*)-4-((*E*)-1-(Furan-2-yl)prop-1-en-2-yl)-3-methyl-4-(trifluoromethyl)oxetan-2-one **(35)**

Following General Procedure D, 2-(trimethylsilyl)propanoic acid (117 mg, 0.8 mmol), *N*,*N*-diisopropylethylamine (210 μL, 1.2 mmol), pivaloyl chloride (132 μL, 1.2 mmol), and MTBE (4.5 mL) for 15 min, followed by (*E*)-1,1,1-trifluoro-4-(furan-2-yl)-3-methylbut-3-en-2-one (81 mg, 0.4 mmol), (2*S*,3*R*)-HyperBTM (6 mg, 20.0 μmol), and *N*,*N*-diisopropylethylamine (71 μL, 0.4 mmol) for 16 h gave, after purification by Biotage Isolera 4 [SNAP KP-Sil 25 g, 36 mL min^–1^, petrol:Et_2_O (98:2 4 CV, 98:2 to 96:4 40 CV)], the title compound (56 mg, 54%) as a yellow oil; [α]_D_^20^ +39.5 (*c*, 1.6, CHCl_3_); Chiral HPLC analysis, Chiralpak AD-H (99.9:0.1 hexane:IPA, flow rate 1.0 mL min^–1^, 254 nm, 30 °C), *t*_R_ (major): 6.4 min, >99:1 er; IR ν_max_ (film) 1848 (C=O), 1175 (C–O); ^1^H NMR (500 MHz, CDCl_3_) δ_H_ 7.51 (1H, d, *J* 1.1, ArC(5)*H*), 6.70 (1H, s, ArC*H*), 6.50–6.48 (2H, m, ArC(3,4)*H*), 4.05 (1H, q, *J* 7.8, C(3)*H*), 2.14 (3H, s, CC*H*_3_), 1.39 (3H, d, *J* 7.6, C(3)C*H*_3_). ^19^F NMR (376 MHz, CDCl_3_) δ_F_ −76.5 (C*F*_3_). ^13^C{^1^H} NMR (126 MHz, CDCl_3_) δ_C_ 168.1 (*C*(2)), 151.4 (Ar*C*(2)), 142.9 (Ar*C*(5)H), 123.7 (q, *J* 283.8, *C*F_3_), 122.5 (ArCH=*C*), 120.4 (Ar*C*(4)H), 112.3 (Ar*C*H=C), 111.6 (Ar*C*(3)H), 80.8 (q, *J* 31.8, *C*(4)), 51.5 (*C*(3)), 15.5 (C(3)*C*H_3_), 9.8 (C*C*H_3_). HRMS (ESI+) *m*/*z* [M + H]^+^ calcd for C_12_H_12_F_3_O_3_ 261.0733, found 261.0730.

##### (3*S*,4*S*)-3-Ethyl-4-((*E*)-1-phenylprop-1-en-2-yl)-4-(trifluoromethyl)oxetan-2-one **(36)**

Following General Procedure D, 2-(trimethylsilyl)butanoic acid (64 mg, 0.4 mmol), *N*,*N*-diisopropylethylamine (105 μL, 0.6 mmol), pivaloyl chloride (66 μL, 0.6 mmol), and MTBE (2 mL) for 15 min, followed by (*E*)-1,1,1-trifluoro-3-methyl-4-phenylbut-3-en-2-one (40 mg, 0.2 mmol), (2*S*,3*R*)-HyperBTM (3 mg, 10.0 μmol), and *N*,*N*-diisopropylethylamine (35 μL, 0.2 mmol) for 16 h gave, after purification by Biotage Isolera 4 [SNAP KP-Sil 10 g, 36 mL min^–1^, petrol:Et_2_O (99:1 4 CV, 99:1 to 94:6 20 CV)], the title compound (42 mg, 72%) as a mixture of diastereoisomers in the ratio of 75:25, colorless oil; [α]_D_^20^ −28.7 (*c*, 0.4, CHCl_3_); Chiral HPLC analysis, Chiralcel OD-H (99.9:0.1 hexane:IPA, flow rate 1.0 mL min^–1^, 254 nm, 30 °C), major diastereoisomer: *t*_R_ (major): 7.0 min, *t*_R_ (minor): 7.4 min, 97:3 er; minor diastereoisomer: *t*_R_(major): 13.9 min, *t*_R_(minor): 20.2 min,90:10 er; IR ν_max_ (film) 1850 (C=O), 1151(C–O); ^1^H NMR (500 MHz, CDCl_3_) major diastereoisomer: δ_H_ 7.43–7.40 (2H, m, Ph*H*), 7.34–7.32 (3H, m, Ph*H*), 6.93 (1H, s, C*H*Ph), 3.89 (1H, dd, *J* 9.6, 7.2, C(3)*H*), 2.01 (3H, m, CH=CC*H*_2_), 1.93–1.79 (2H, m, C*H*_2_CH_3_), 1.18 (3H, t, *J* 7.5, CH_2_C*H*_3_); minor diastereoisomer: δ_H_ 7.43–7.40 (2H, m, Ph*H*), 7.34–7.32 (3H, m, Ph*H*), 6.84 (1H, s, C*H*Ph), 3.70 (1H, dd, *J* 9.6, 7.2, C(3)*H*), 2.04–2.03 (3H, m, CH=CC*H*_2_), 1.93–1.79 (2H, m, C*H*_2_CH_3_), 1.22 (3H, t, *J* 7.5, CH_2_C*H*_3_). ^19^F NMR (376 MHz, CDCl_3_) major diastereoisomer: δ_F_ −72.5 (s, C*F*_3_); minor diastereoisomer: δ_F_ −76.5 (s, C*F*_3_). ^13^C{^1^H} NMR (126 MHz, CDCl_3_) major diastereoisomer: δ_C_ 167.7 (*C*O), 135.6 (Ph*C*(1)), 132.2 (*C*H=C), 129.2 (Ph*C*(3,5)H), 128.4 (Ph*C*(2,6)H), 127.7 (Ph*C*(4)H), 125.8 (PhCH=*C*CH_3_), 123.8 (q, *J* 283.2, *C*F_3_), 80.4 (q, *J* 31.5, *C*CF_3_), 71.9 (*C*≡CH), 57.9 (C(3)), 26.5 (C*C*H_3_), 18.9 (*C*H_2_CH_3_), 11.3 (CH_2_*C*H_3_); minor diastereoisomer: δ_C_ 167.9 (*C*O), 135.4 (Ph*C*(1)), 131.4 (Ar*C*H), 129.1 (Ph*C*(3,5)H), 128.4 (Ph*C*(2,6)H), 127.8 (Ph*C*(4)H), 125.8 (PhCH=*C*CH_3_), 123.6 (q, *J* 283.2, *C*F_3_), 62.0 (C(3)), 23.2 (C*C*H_3_), 15.1 (*C*H_2_CH_3_), 10.1 (CH_2_*C*H_3_). HRMS (ESI+) *m*/*z* [M + H]^+^ calcd for C_15_H_16_F_3_O_2_ 285.1097, found 285.1099.

##### (3*S*,4*S*)-3-Allyl-4-((*E*)-1-phenylprop-1-en-2-yl)-4-(trifluoromethyl)oxetan-2-one **(37)**

Following General Procedure D, 2-(trimethylsilyl)pent-4-enoic acid (69 mg, 0.4 mmol), *N*,*N*-diisopropylethylamine (105 μL, 0.6 mmol), pivaloyl chloride (66 μL, 0.6 mmol), and MTBE (3 mL) for 15 min, followed by (*E*)-1,1,1-trifluoro-3-methyl-4-phenylbut-3-en-2-one (43 mg, 0.2 mmol), (2*S*,3*R*)-HyperBTM (3 mg, 10.0 μmol), and *N*,*N*-diisopropylethylamine (35 μL, 0.2 mmol) for 16 h gave, after purification by Biotage Isolera 4 [SNAP KP-Sil 10 g, 36 mL min^–1^, petrol:Et_2_O (99:1 4 CV, 99:1 to 95:5 30 CV)], the title compound (42 mg, 70%) as a mixture of diastereoisomers in the ratio of 75:25, colorless oil; [α]_D_^20^ −23.5 (*c*, 0.4, CHCl_3_); Chiral HPLC analysis, Chiralcel OJ-H (99.8:0.2 hexane:IPA, flow rate 1.0 mL min^–1^, 254 nm, 30 °C), major diastereoisomer: *t*_R_ (minor): 10.2 min, *t*_R_ (major): 13.1 min, 99:1 er; minor diastereoisomer: *t*_R_ (major): 16.6 min, *t*_R_ (minor): 19.1 min,93:7 er; IR ν_max_ (film) 1854 (C=O), 1173 (C–O), 937 (C=C); ^1^H NMR (400 MHz, CDCl_3_) major diastereoisomer: δ_H_ 7.44–7.32 (5H, m, Ar*H*), 6.95 (1H, s, C*H*Ph), 5.93–5.84 (1H, m, C*H*=CH_2_), 5.24–5.19 (2H, m, CH=C*H*_2_), 4.07 (1H, t, *J* 7.8, C(3)*H*), 2.59–2.56 (2H, m, C(3)C*H*_2_), 2.02–2.01 (3H, m, C*H*_3_); minor diastereoisomer: δ_H_ 7.44–7.32 (5H, m, Ar*H*), 6.84 (1H, d, *J* 16.1, C*H*Ph), 5.93–5.84 (1H, m, C*H*=CCH_3_), 5.32–5.21 (2H, m, CH=C*H*_2_), 3.85 (1H, t, *J* 8.1, C(3)*H*), 2.88–2.70 (2H, m, C(3)C*H*_2_), 2.03 (3H, m, C*H*_3_). ^19^F NMR (376 MHz, CDCl_3_) major diastereoisomer: δ_F_ −72.4 (s, C*F*_3_); minor diastereoisomer: δ_F_ −76.5 (s, C*F*_3_). ^13^C{^1^H} NMR (126 MHz, CDCl_3_) major diastereoisomer: δ_C_ 167.1 (*C*O), 135.5 (Ph*C*(1)), 132.6 (*C*H=CH_2_), 132.4 (Ph*C*(4)H), 129.2 (Ph*C*(3,5)H), 128.4 (Ph*C*(2,6)H), 127.8 (Ph*C*H), 125.6 (PhCH=*C*CH_3_), 123.8 (q, *J* 283.8, *C*F_3_), 118.6 (CH=*C*H_2_), 80.5 (q, *J* 32.1, *C*CF_3_), 55.8 (C(3)), 29.1 (C(3)*C*H_2_), 15.2 (*C*H_3_); minor diastereoisomer: δ_C_ 167.2 (*C*O), 135.3 (Ph*C*(1)), 132.7 (*C*H=CH_2_), 131.7 (Ph*C*(4)H), 129.1 (Ph*C*(3,5)H), 128.4 (Ph*C*(2,6)H), 127.9 (Ph*C*H), 127.0 (PhCH=*C*CH_3_), 123.6 (q, *J* 283.8, *C*F_3_), 118.9 (CH=*C*H_2_), 60.0 (C(3)), 29.4 (C(3)*C*H_2_), 14.2 (*C*H_3_). HRMS (ESI+) *m*/*z* [M + OH]^−^ calcd for C_16_H_16_F_3_O_3_ 313.1057, found 313.1058.

##### (3*S*,4*S*)-4-((*E*)-1-Phenylprop-1-en-2-yl)-3-(prop-2-yn-1-yl)-4-(trifluoromethyl)oxetan-2-one **(38)**

Following General Procedure D, 2-(trimethylsilyl)pent-4-ynoic acid (68 mg, 0.4 mmol), *N*,*N*-diisopropylethylamine (105 μL, 0.6 mmol), pivaloyl chloride (66 μL, 0.6 mmol), and MTBE (2 mL) for 15 min, followed by (*E*)-1,1,1-trifluoro-3-methyl-4-phenylbut-3-en-2-one (40 mg, 0.2 mmol), (2*S*,3*R*)-HyperBTM (3 mg, 10.0 μmol), and *N*,*N*-diisopropylethylamine (35 μL, 0.2 mmol) for 16 h gave, after purification by Biotage Isolera 4 [SNAP KP-Sil 10 g, 36 mL min^–1^, petrol:Et_2_O (99:1 4 CV, 99:1 to 95:5 30 CV)], the title compound (36 mg, 61%) as a mixture of diastereoisomers in the ratio of 70:30, colorless oil; [α]_D_^20^ +9.8 (*c*, 0.6, CHCl_3_); Chiral HPLC analysis, Chiralcel OJ-H (99.8:0.2 hexane:IPA, flow rate 1.0 mL min^–1^, 254 nm, 30 °C), major diastereoisomer: *t*_R_ (major): 26.5 min, *t*_R_ (minor): 31.3 min, 98:2 er; minor diastereoisomer: Chiralcel OD-H (99.9:0.1 hexane:IPA, flow rate 1.0 mL min^–1^, 254 nm, 30 °C), *t*_R_ (minor): 24.8 min, *t*_R_ (major): 28.9 min,90:10 er; IR ν_max_ (film) 1855 (C=O), 1173 (C–O), 937 (C=C).

^1^H NMR (500 MHz, CDCl_3_) major diastereoisomer: δ_H_ 7.44–7.33 (5H, m, Ph*H*), 6.96 (1H, s, C*H*Ph), 4.20 (1H, dd, *J* 8.4, 5.5, C≡C*H*), 2.79–2.68 (2H, m, C(3)C*H*_2_), 2.16 (1H, t, *J* 2.7, C(3)*H*), 2.11–2.10 (3H, m, C*H*_3_); minor diastereoisomer: δ_H_ 7.44–7.33 (5H, m, Ph*H*), 6.96 (1H, s, C*H*Ph), 4.06 (1H, dd, *J* 10.1, 6.0, C≡C*H*), 3.01–2.88 (2H, m, C(3)C*H*_2_), 2.20 (1H, t, *J* 2.7, C(2)*H*), 2.11–2.10 (3H, m, C*H*_3_). ^19^F NMR (376 MHz, CDCl_3_) major diastereoisomer: δ_F_ −72.5 (s, C*F*_3_); minor diastereoisomer: δ_F_ −76.9 (s, C*F*_3_). ^13^C{^1^H} NMR (126 MHz, CDCl_3_) major diastereoisomer: δ_C_ 165.6 (*C*O), 135.4 (Ph*C*(1)), 133.0 (Ar*C*H), 129.2 (Ph*C*(3,5)H), 128.5 (Ph*C*(2,6)H), 127.9 (Ph*C*(4)H), 125.4 (PhCH=*C*CH_3_), 123.5 (q, *J* 283.4, *C*F_3_), 80.5 (q, *J* 32.1, *C*CF_3_), 71.9 (*C*≡CH), 58.9 (C≡*C*H), 55.7 (C(3)), 15.2 (C(3)*C*H_2_), 14.2 (*C*H_3_); minor diastereoisomer: δ_C_ 165.7 (*C*O), 135.3 (Ph*C*(1)), 132.4 (Ar*C*H), 129.1 (Ph*C*(3,5)H), 128.5 (Ph*C*(2,6)H), 128.0 (Ph*C*(4)H), 125.2 (PhCH=*C*CH_3_), 123.5 (q, *J* 283.4, *C*F_3_), 71.4 (*C*≡CH), 58.9 (C≡*C*H), 55.7 (C(3)), 15.2 (C(3)*C*H_2_), 14.0 (*C*H_3_). HRMS (ESI+) *m*/*z* [M + Na]^+^ calcd for C_16_H_13_NaF_3_O_2_ 317.0760, found 317.0757.

##### (3*S*,4*S*)-3-Benzyl-4-((*E*)-1-phenylprop-1-en-2-yl)-4-(trifluoromethyl)oxetan-2-one **(39)**

Following General Procedure D, 3-phenyl-2-(trimethylsilyl)propanoic acid (90 mg, 0.4 mmol), *N*,*N*-diisopropylethylamine (105 μL, 0.6 mmol), pivaloyl chloride (66 μL, 0.6 mmol), and MTBE (2 mL) for 15 min, followed by (*E*)-1,1,1-trifluoro-3-methyl-4-phenylbut-3-en-2-one (40 mg, 0.2 mmol), (2*S*,3*R*)-HyperBTM (3 mg, 10.0 μmol), and *N*,*N*-diisopropylethylamine (35 μL, 0.2 mmol) for 16 h gave, after purification by Biotage Isolera 4 [SNAP KP-Sil 10 g, 36 mL min^–1^, petrol:Et_2_O (99:1 4 CV, 99:1 to 94:6 20 CV)], the title compound (53 mg, 75%) as a mixture of diastereoisomers in the ratio of 76:24, white solid; mp 66–68 °C; [α]_D_^20^ +3.3 (*c*, 0.5, CHCl_3_); Chiral HPLC analysis, Chiralcel OD-H (99.9:0.1 hexane:IPA, flow rate 0.5 mL min^–1^, 211 nm, 30 °C), major diastereoisomer: *t*_R_ (major): 83.6 min, *t*_R_ (minor): 101.5 min, >99:1 er; minor diastereoisomer: Chiralcel OD-H (99.9:0.1 hexane:IPA, flow rate 0.5 mL min^–1^, 254 nm, 30 °C), *t*_R_(major): 64.1 min, *t*_R_(minor): 108.2 min,96:4 er; IR ν_max_ (film) 1850 (C=O), 1173 (C–O); ^1^H NMR (500 MHz, CDCl_3_) major diastereoisomer: δ_H_ 7.44–7.29 (7H, m, Ph*H*), 7.25–7.23 (3H, m, Ph*H*), 6.98 (1H, s, C*H*Ph), 4.34 (1H, dd, *J* 8.7, 7.5, C(3)*H*), 3.18–3.07 (2H, m, C(3)C*H*_2_), 1.93–1.92 (3H, m, C*H*_3_); minor diastereoisomer: 7.44–7.29 (10H, m, Ph*H*), 6.65 (1H, s, C*H*Ph), 4.07 (1H, t, *J* 7.6, C(3)*H*), 3.48–3.19 (2H, m, C(3)C*H*_2_), 1.90 (3H, m, C*H*_3_). ^19^F NMR (376 MHz, CDCl_3_) major diastereoisomer: δ_F_ −72.3 (s, C*F*_3_); minor diastereoisomer: δ_F_ −76.2 (s, C*F*_3_). ^13^C{^1^H} NMR (126 MHz, CDCl_3_) major diastereoisomer: δ_C_ 167.1 (*C*(2)), 136.7 (C(3)CH_2_Ph*C*(1)), 135.5 (C=CHPh*C*(1)), 132.7 (Ph*C*H), 129.2 (C=CHPh*C*(4)H), 128.9 (C=CHPh*C*(2,6)H), 128.48 (C(3)CH_2_Ph*C*(3,5)H), 128.46 (C=CHPh*C*(3,5)H), 127.9 (C(3)CH_2_Ph*C*(4)H), 127.4 (C(3)CH_2_Ph*C*(2,6)H), 125.6 (PhCH=*C*CH_3_), 123.8 (q, *J* 284.0, *C*F_3_), 79.2 (q, *J* 33.1, *C*CF_3_), 30.9 (*C*H_2_Ar), 57.0 (C(3)), 15.3 (*C*H_3_); minor diastereoisomer: δ_C_ 167.2 (*C*(2)), 135.7 (C(3)CH_2_Ph*C*(1)), 135.2 (C=CHPh*C*(1)), 131.8 (Ph*C*H), 129.1 (C=CHPh*C*(4)), 129.0 (C=CHPh*C*(2,6)H), 128.8 (C(3)CH_2_Ph*C*(3,5)H), 128.4 (C=CHPh*C*(3,5)H), 127.9 (C(3)CH_2_Ph*C*(4)), 127.4 (C(3)CH_2_Ph*C*(2,6)H), 125.6 (PhCH=*C*CH_3_), 123.8 (q, *J* 284.0, *C*F_3_), 61.9 (*C*(3)), 31.1 (*C*H_2_Ar), 14.1 (*C*H_3_). HRMS (ESI+) *m*/*z* [M + Na]^+^ calcd for C_20_H_17_NaF_3_O_2_ 369.1073, found 369.1072.

##### (3*S*,4*S*)-3-(Naphthalen-2-ylmethyl)-4-((*E*)-1-phenylprop-1-en-2-yl)-4-(trifluoromethyl)oxetan-2-one **(40)**

Following General Procedure D, 3-(naphthalen-2-yl)-2-(trimethylsilyl)propanoic acid (114 mg, 0.4 mmol), *N*,*N*-diisopropylethylamine (105 μL, 0.6 mmol), pivaloyl chloride (66 μL, 0.6 mmol), and MTBE (2 mL) for 15 min, followed by (*E*)-1,1,1-trifluoro-3-methyl-4-phenylbut-3-en-2-one (40 mg, 0.2 mmol), (2*S*,3*R*)-HyperBTM (3 mg, 10.0 μmol), and *N*,*N*-diisopropylethylamine (35 μL, 0.2 mmol) for 16 h gave, after purification by Biotage Isolera 4 [SNAP KP-Sil 10 g, 36 mL min^–1^, petrol:Et_2_O (99:1 4 CV, 99:1 to 95:5 30 CV)], the title compound (63 mg, 78%) as a mixture of diastereoisomers in the ratio of 70:30, white solid; mp 68–70 °C; [α]_D_^20^ +8.0 (*c*, 0.2, CHCl_3_); Chiral HPLC analysis, Chiralcel OD-H (95:5 hexane:IPA, flow rate 1.0 mL min^–1^, 254 nm, 30 °C), major diastereoisomer: *t*_R_ (major): 17.2 min, *t*_R_ (minor): 47.7 min, >99:1 er; minor diastereoisomer: *t*_R_ (major): 10.5 min, *t*_R_ (minor): 13.1 min, 97:3 er; IR ν_max_ (film) 1850 (C=O), 1173 (C–O), 934 (C=C); ^1^H NMR (500 MHz, CDCl_3_) major diastereoisomer: δ_H_ 7.87–7.79 (3H, m, Ar*H*), 7.70 (1H, s, Ar*H*), 7.51–7.49 (2H, m, Ar*H*), 7.45–7.42 (2H, m, Ar*H*), 7.38–7.34 (3H, m, Ar*H*), 7.22–7.20 (1H, m, Ar*H*), 7.01 (1H, s, C*H*Ph), 4.46 (1H, dd, *J* 8.4, 7.6, C(3)*H*), 3.30 (2H, qd, *J* 15.0, 8.4, C(3)C*H*_2_), 1.95 (3H, m, C*H*_3_); minor diastereoisomer: (Selected signal)δ_H_ 6.70 (1H, s, C*H*Ph), 4.19 (1H, t, *J* 7.6, C(3)*H*), 3.64–3.37 (2H, m, C(3)C*H*_2_), 1.90 (3H, m, C*H*_3_). ^19^F NMR (376 MHz, CDCl_3_) major diastereoisomer: δ_F_ −72.2 (s, C*F*_3_); minor diastereoisomer: δ_F_ −76.1 (s, C*F*_3_). ^13^C{^1^H} NMR (126 MHz, CDCl_3_) major diastereoisomer: δ_C_ 167.1 (*C*O), 135.5 (Ar*C*(2)), 133.4 (Ph*C*(1)), 133.2 (Ar*C*(8a)), 132.8 (Ar*C*(1)H), 132.5 (Ar*C*(4a)), 129.3 (Ph*C*(3,5)H), 128.5 (Ar*C*(3)H), 127.9 (Ar*C*(8)H), 127.7 (Ph*C*(4)H), 127.6 (Ph*C*(2,6)H), 127.2 (Ar*C*(4)H), 126.5 (Ar*C*(5)H), 126.4 (Ar*C*(6,7)H), 126.1 (Ar*C*H=CCH_3_), 125.6 (ArCH=*C*CH_3_), 123.9 (q, *J* 283.8, *C*F_3_), 80.8 (q, *J* 31.8, *C*CF_3_), 57.0 (C(3)), 31.1 (*C*H_2_Ar), 15.3 (*C*H_3_); minor diastereoisomer: δ_C_ 167.2 (*C*O), 135.2 (Ar*C*(2)), 134.0 (Ph*C*(1)), 133.5 (Ar*C*(8a)), 132.5 (Ar*C*(4a)), 131.8 (Ar*C*(1)H), 129.1 (Ph*C*(3,5)H), 128.8 (Ar*C*(3)H), 128.4 (Ar*C*(8)H), 127.8 (Ph*C*(4)H), 127.7 (Ph*C*(2,6)H), 127.6 (Ar*C*(4)H), 126.6 (Ar*C*(5)H), 126.5 (Ar*C*(6,7)H), 126.1 (Ar*C*H=CCH_3_), 125.6 (ArCH=*C*CH_3_), 123.8 (q, *J* 283.8, *C*F_3_), 61.7 (C(3)), 31.3 (*C*H_2_Ar), 14.2 (*C*H_3_). HRMS (ESI+) *m*/*z* [M + Na]^+^ calcd for C_24_H_19_NaF_3_O_2_ 419.1229, found 419.1219.

##### (2*S*,*3*R,*E*)-*N*-Benzyl-5-(4-bromophenyl)-3-hydroxy-2-methyl-3-(trifluoromethyl)pent-4-enamide **(43)**

β-Lactone **18** (47 mg, 0.14 mmol) and benzylamine (75 μL, 0.70 mmol) in dichloromethane (3.0 mL) at r.t. for 16 h gave, after purification by Biotage Isolera 4 [SNAP KP-Sil 10 g, 36 mL min^–1^, CH_2_Cl_2_:MeOH (100:0 to 95:5, 30 CV)], the title compound (58 mg, 93%) as a white solid; mp 128–130 °C; [α]_D_^20^ −47.3 (*c*, 0.4, CHCl_3_); Chiral HPLC analysis, Chiralcel OD-H (95:5 hexane:IPA, flow rate 1.0 mL min^–1^, 254 nm, 30 °C), *t*_R_ (minor): 46.9 min, *t*_R_ (major): 51.7 min, >99:1 er; IR ν_max_ (film) 3298 (OH); ^1^H NMR (400 MHz, CDCl_3_) δ_H_ 7.50–7.29 (9H, m, Ar*H*), 7.03 (1H, d, *J* 15.6, CH=C*H*Ph), 6.54 (1H, s, O*H*), 6.03 (1H, t, *J* 5.6, N*H*), 5.92 (1H, d, *J* 15.8, C*H*=CHPh), 4.50 (2H, m, NHC*H*_2_), 2.57 (1H, d, *J* 7.0, C(2)*H*), 1.28 (3H, d, *J* 7.2, C(2)C*H*_3_). ^19^F NMR (376 MHz, CDCl_3_) δ_F_ −78.4 (s, C*F*_3_). ^13^C{^1^H} NMR (101 MHz, CDCl_3_) δ_C_ 175.4 (*C*O), 137.0 (Ph*C*(1)), 134.7 (Ar*C*(1)), 133.2 (ArCH=*C*H), 131.8 (Ar*C*(3,5)H), 129.0 (Ar*C*(2,6)H), 128.4 (Ph*C*(3,5)H), 128.0 (Ar*C*H=CH), 127.9 (Ph*C*(2,6)H), 125.5 (q, *J* 286.9, *C*F_3_), 123.2 (Ph*C*(4)H), 122.2 (Ar*C*(4)Br), 77.7 (q, *J* 27.6, *C*CF_3_), 43.8 (N*C*H_2_), 40.7 (C(2)), 14.0 (C*C*H_3_). HRMS (ESI+) *m*/*z* [M + Na]^+^ calcd for C_20_H_19_NNaBrF_3_O_2_ 464.0443, found 464.0437.

##### (*S*,*E*)-5-(4-Bromophenyl)-4-methyl-3-(trifluoromethyl)pent-4-ene-1,3-diol **(44)**

1 M *i*-Bu_2_AlH (DIBAL) 1 M solution in hexane (1.74 mL, 2 equiv) was added dropwise to a solution of a β-lactone (**11**) (292 mg, 0.87 mmol) (1 equiv) in CH_2_Cl_2_ (0.1 M) at −78 °C under an inert atmosphere, and the reaction was allowed to stir for 90 min. Aqueous NH_4_Cl was added and the mixture was allowed to warm to r.t. The aqueous layer was extracted with CH_2_Cl_2_ (2 × 20 mL) and the combined organic layers were dried (MgSO_4_), filtered, and concentrated in vacuo to give a residue, which after purification by Biotage Isolera 4 [SNAP KP-Sil 10 g, 36 mL min^–1^, petrol:Et_2_O (90:10 4 CV, 90:10 to 50:50 30 CV)] gave the title compound (161 mg, 55%) as a white solid; mp 42–44 °C; [α]_D_^20^ −37.5 (*c*, 0.2, CHCl_3_); Chiral HPLC analysis, Chiralpak AS-H (98:2 hexane:IPA, flow rate 1.0 mL min^–1^, 254 nm, 30 °C), *t*_R_ (minor): 14.1 min, *t*_R_ (major): 17.0 min, 95:5 er; IR ν_max_ (film) 3362(OH), 1171 (C–O); ^1^H NMR (400 MHz, CDCl_3_) δ_H_ 7.52–7.48 (2H, m, ArC(3,5)*H*), 7.18–7.16 (2H, m, ArC(2,6)*H*), 7.01 (1H, s, C*H*Ar), 4.61 (1H, s, CO*H*), 4.07–3.92 (2H, m, C*H*_2_OH), 2.34–2.17 (2H, m, CC*H*_2_), 1.99 (1H, s, CH_2_O*H*), 1.90 (3H, t, *J* 1.2, C*H*_3_). ^19^F NMR (377 MHz, CDCl_3_) δ_F_ −78.2 (s, C*F*_3_). ^13^C{^1^H} NMR (101 MHz, CDCl_3_) δ_C_ 136.2 (Ar*C*(1)), 134.4 (ArCH=*C*CH_3_), 131.3 (Ar*C*(3,5)H), 130.8 (Ar*C*(2,6)H), 129.1 (Ar*C*H=CCH_3_), 125.2 (q, *J* 286.2, *C*F_3_), 120.8 (Ar*C*(4)), 79.3 (q, *J* 27.2, *C*CF_3_), 59.6 (*C*H_2_OH), 33.3 (C*C*H_2_), 14.7 (*C*H_3_). HRMS (ESI+) *m*/*z* [M + Na]^+^ calcd for C_13_H_14_NaBrF_3_O_2_ 361.0021, found 361.0019.

##### (*S*,*E*)-2-(1-(4-Bromophenyl)prop-1-en-2-yl)-2-(trifluoromethyl)oxetane **(45)**

NaH 60% in mineral oil (24 mg, 0.6 mmol, 2 equiv) was added to a solution of a diol **44** (102 mg, 0.3 mmol, 1 equiv) in THF (0.02 M) under an inert atmosphere and the mixture was stirred at r.t. for 10 min. 2,4,6-Triisopropylbenzenesulfonyl chloride (82 mg, 0.27 mmol, 0.9 equiv) was added, and the mixture stirred for 16 h. Aqueous NH_4_Cl was added and the aqueous phase extracted with EtOAc (2 × 10 mL). The combined organic layers were dried (MgSO_4_), filtered, and concentrated in vacuo to give a residue, which after purification by Biotage Isolera 4 [SNAP KP-Sil 10 g, 36 mL min^–1^, petrol:Et_2_O (98:2 4 CV, 98:2 to 80:20 20 CV)] gave the title compound (81 mg, 84%) as a colorless oil; [α]_D_^20^ −15.4 (*c*, 0.4, CHCl_3_); Chiral HPLC analysis, Chiralpak AD-H (99:0.2 hexane:IPA, flow rate 1.0 mL min^–1^, 254 nm, 30 °C), *t*_R_ (minor): 5.7 min, *t*_R_ (major): 6.0 min, 96:4 er; IR ν_max_ (film) 1157(OH); ^1^H NMR (400 MHz, CDCl_3_) δ_H_ 7.53–7.50 (2H, m, ArC(3,5)*H*), 7.23–7.21 (2H, m, ArC(2,6)*H*), 6.74 (1H, s, C*H*Ar), 4.80–4.75 (1H, m, CH_A_*H*_B_OH), 4.58–4.53 (1H, m, C*H*_A_H_B_OH), 3.07–3.00 (1H, m, CCH_A_*H*_B_), 2.82–2.75 (1H, m, CC*H*_A_H_B_), 1.89 (3H, t, *J* 1.2, C*H*_3_). ^19^F NMR (377 MHz, CDCl_3_) δ_F_ −81.5 (s, C*F*_3_). ^13^C{^1^H} NMR (101 MHz, CDCl_3_) δ_C_ 135.4 (Ar*C*(1)), 134.0 (ArCH=*C*CH_3_), 131.4 (Ar*C*(3,5)H), 130.7 (Ar*C*(2,6)H), 127.7 (Ar*C*H=CCH_3_), 124.9 (q, *J* 284.6, *C*F_3_), 121.1 (Ar*C*(4)), 86.2 (q, *J* 30.4, *C*CF_3_), 66.7 (*C*H_2_OH), 27.6 (C*C*H_2_), 12.9 (*C*H_3_). HRMS (ESI+) *m*/*z* [M + H]^+^ calcd for C_13_H_13_BrF_3_O 321.0096, found 321.0096.

## Data Availability

The data underlying this study are available in the published article, in its Supporting Information, and openly available in the St Andrews PURE repository that can be accessed at DOI: 10.17630/ec54103f-32b7-422b-8e9a-f2c1f623065c.

## Supporting Information Available

Full experimental procedures, characterization data, NMR spectra, and HPLC chromatograms for all new compounds, as well as crystallographic data for **17**, **18**, and **34** (PDF)
