# Supplementary material for: Probing Regio- and Enantioselectivity in the Formal [2 + 2] Cycloaddition of C(1)-Alkyl Ammonium Enolates with β- and α,β-Substituted Trifluoromethylenones
Source: J Org Chem. 2023 May 15;88(12):7784–99. doi: 10.1021/acs.joc.2c02688 (PMC10278128; doi:10.1021/acs.joc.2c02688)
Supplement: Supplementary file 1 — jo2c02688_si_001.pdf [file jo2c02688_si_001.pdf]

Supporting Information

Probing Regio- and Enantioselectivity in the Formal [2+2] Cycloaddition of C(1)-alkyl ammonium enolates with β- and α,β-substituted trifluoromethylenones

Yihong Wang, Claire M. Young, David. B. Cordes, Alexandra M. Z. Slawin and Andrew D. Smith*

EaStCHEM, School of Chemistry, University of St Andrews, North Haugh, St Andrews, Fife, KY16 9ST, U.K.

E-mail for Andrew D. Smith: ads10@st-andrews.ac.uk

Contents

1. Full optimization table S1

2. Mechanistic Investigations S2

3. Determination of Product Configuration by X-ray Crystallography......S5

Appendix I. 1H, 19F and 13C{1H} NMR Spectra......S9

Appendix II. HPLC Traces S110

1. Full optimization table

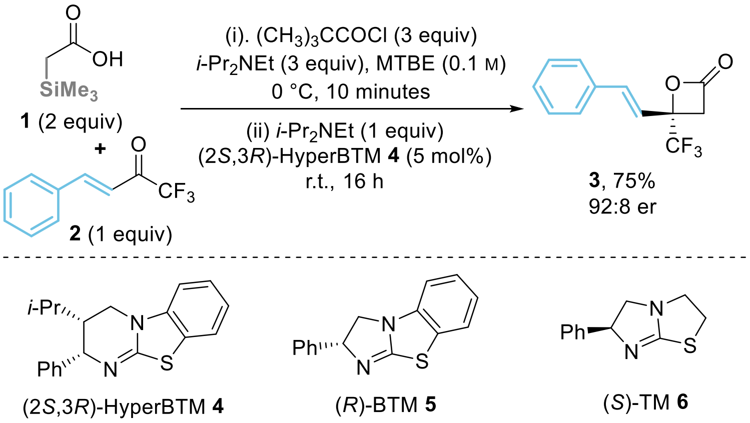

Entry	Variant	yield[b](%)	er[c]
1	None	75	92:8
2	CH_2Cl_2	18	76:24
3	EtOAc	52	86:14
4	CPME	45	90:10
5	THF	13	–
6	Toluene	80	87:13
7	DMF	46	65:35
8	MeCN	18	60:40
9	(*R*)-BTM **5**, 20 h	12	75:25
10	(−)-TM **6**	<1[d]	–
11	Et_3N	45	92:8
12	$NaHCO_3$	30	–
13	Cs_2CO_3	30	–
14	TsCl	<1[d]	–
15	PhCOCl	57	89:11
16	PNPCOCl	48	89:11

a) $(CH_3)_3CCOCl$ (1.2 mmol), i-Pr_2NEt (1.2 mmol) and acid 1 (0.8 mmol) in MTBE (4 mL, 0.1 m) was stirred at 0°C for 10 minutes before addition of i-Pr_2NEt (0.4 mmol), enone 2 (0.4 mmol) and (2S,3R)-HyperBTM 4 (5 mol%) at r.t. for 16 hours. MTBE = methyl tert-butyl ether. r.t. = room temperature (18 ℃). BTM = benzotetramisole. TM = tetramisole.b) Isolated yield; c) Determined by chiral HPLC analysis; d) Determined by 1H NMR of crude reaction mixture.

2. Mechanistic Investigations

General Considerations: Stock solutions were prepared by adding reagents directly to volumetric glassware. The required stock solution volume was measured using a gas-tight 100 μL microsyringe. 50 μL of Sample were taken throughout the reaction mixture dissolved in 0.3 mL $CDCl_3$ directly and added to the NMR tube for each kinetic experiment. Data were collected at 376 MHz, kinetic experiments (arrays of spectra) were implemented using Bruker Topspin software. The spectra were phase and baseline corrected in MestReNova, each spectrum was processed manually. Aliquots (50 microL) were removed from the reaction mixture for analysis every 60 minutes for first 480 minutes of reaction, the sample for the last measurement was removed at 1440 minutes. Control experiment indicated that the reaction stopped when the reaction mixture dissolved in 0.3 mL $CDCl_3$. Parameters for $^{19}F\{^1H\}$ spectra: 60 ppm sweep width (−60 to −120 ppm), number scans (ns) = 4, spectral centre (o1p) = −90 ppm, d1 relaxation delay = 25 s. The internal standard fluorobenzene was referenced to −113.175 ppm and integrated as 1000. The same integral range was used throughout for each species: fluorobenzene (−113.173 to −113.265 ppm) (*E*)-1,1,1-trifluoro-4-phenylbut-3-en-2-one **2** (−77.690 to −77.755 ppm), product major diastereoisomer **15 major** (−79.819 to −79.832 ppm), product minor diastereoisomer **15 minor** (−72.258 to −72.289 ppm), product major diastereoisomer **56 major** (−72.390 to −72.422 ppm).

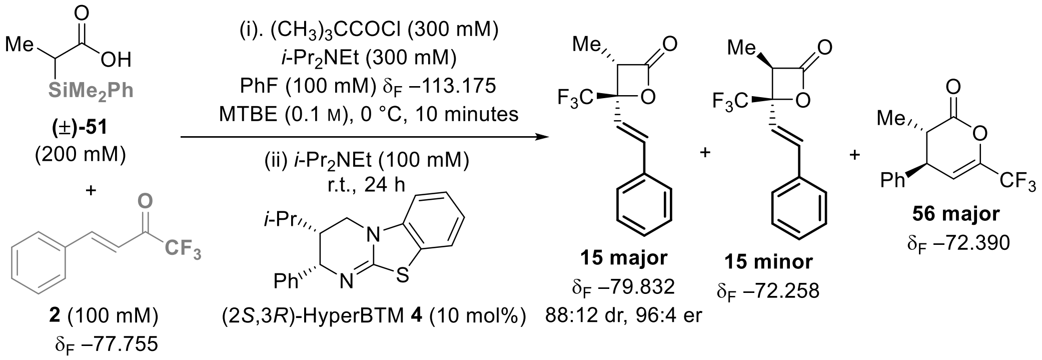

Scheme S1. General reaction scheme for NMR analysis.

The following stock solutions were prepared at the start of the day on which the experiment was performed:

Stock solution A: 2-(Dimethyl(phenyl)silyl)propanoic acid (±)-**51** (166.7 mg, 0.8 mmol, 200 mM) and fluorobenzene (113 μL, 1.2 mmol) in 2 mL MTBE.

Stock solution B: (*E*)-1,1,1-trifluoro-4-phenylbut-3-en-2-one **2** (80.0 mg, 0.4 mmol, 100 mM), *N*,*N*-diisopropylethylamine (70 µL, 0.4 mmol, 100 mM) in 2 mL MTBE.

Stock solution A (2 mL) was transferred to a flame-dried Schlenk tube under N_2, pivaloyl chloride (147 µL, 1.2 mmol, 300 mM) and *N*,*N*-diisopropylethylamine (209 µL, 1.2 mmol, 300 mM) were added at 0 °C and the mixture was stirred for 15 min. Stock solution B was then added to the reaction mixture and an initial $^{19}F\{^{1}H\}$ spectrum was acquired. (2*R*,3*S*)-HyperBTM **4** (12.3 mg, 0.04 mmol, 10 mM) was added to the reaction mixture and the reaction was stirred for the specified time at r.t..

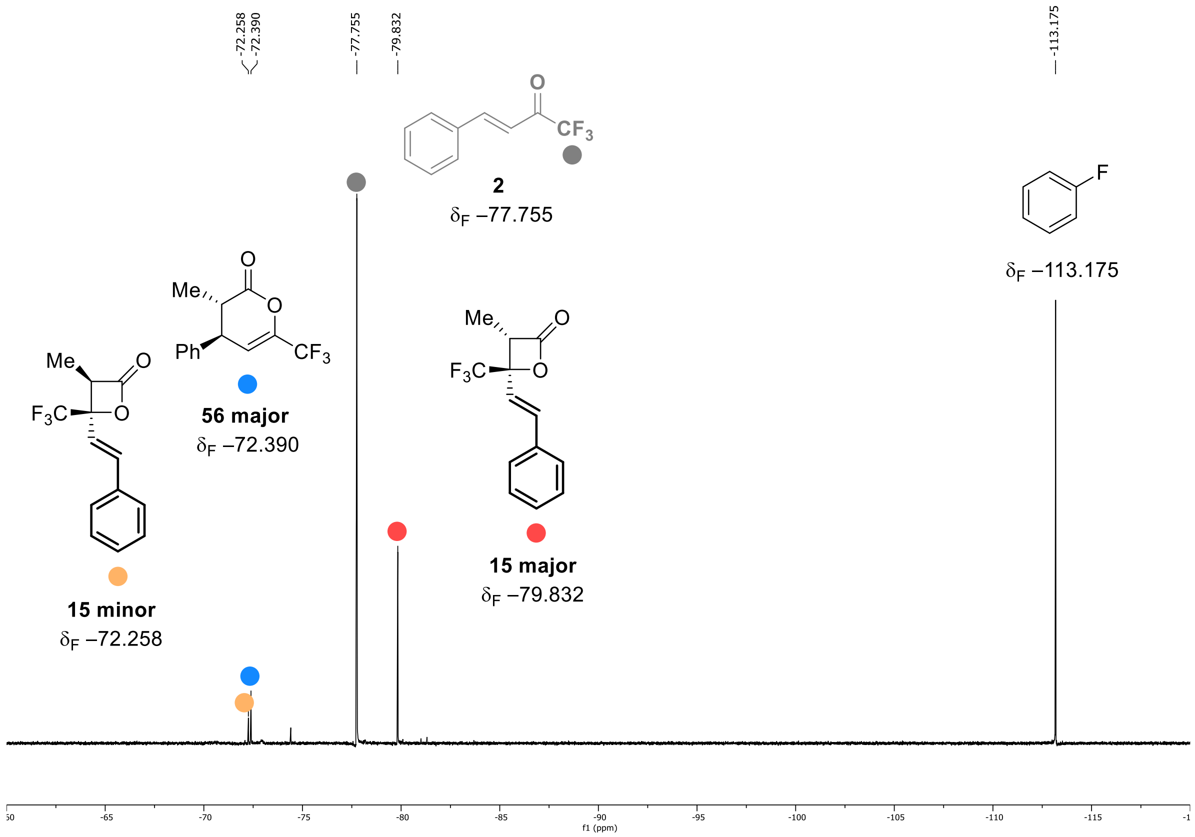

Figure S1. Superimposed reaction spectra for standard reaction conditions.

Concentration data for standard reaction:

Replicate	**Time** (minutes)	0	60	120	180	240	300	360	420	480	1440
**1**	**2 (mM)**	100.09	94.15	88.20	83.69	76.29	72.03	65.48	59.16	54.81	11.41
	**15 major (mM)**	0	4.39	8.55	11.75	17.14	20.24	24.93	29.57	32.32	63.19

	**15 minor (mM)**	0	0.42	0.99	1.36	1.90	2.30	3.02	3.36	3.78	7.67
	**56 major (mM)**	0	0.83	1.71	2.36	3.40	4.00	4.86	5.76	6.64	12.92
**2**	**2 (mM)**	100.20	94.16	88.25	83.35	76.24	72.72	65.59	60.73	57.12	13.41
	**15 major (mM)**	0	4.38	8.44	12.01	17.08	19.57	24.74	28.23	30.55	61.08
	**15 minor (mM)**	0	0.42	0.98	1.37	2.00	2.28	3.05	3.29	3.66	7.30
	**56 major (mM)**	0	0.82	1.72	2.38	3.37	3.96	4.90	5.66	6.33	12.71
**3**	**2 (mM)**	100.36	94.78	88.99	84.10	78.26	74.45	66.31	60.42	56.80	15.02
	**15 major (mM)**	0	4.02	8.12	11.19	16.38	18.62	24.42	28.48	31.50	60.20
	**15 minor (mM)**	0	0.40	0.97	1.34	1.98	2.28	3.00	3.32	3.57	7.56
	**56 major (mM)**	0	0.78	1.78	2.33	3.32	3.96	4.87	5.65	6.25	12.56

3. Determination of Product Configuration by X-ray Crystallography

Crystals of compounds **17**, **18** and **34** were grown by diffusion of petroleum ether into a chloroform solution of the compound. X-ray diffraction data for all three compounds were collected using a Rigaku MM-007HF High Brilliance RA generator/confocal optics with XtaLAB P100 or XtaLAB P200 diffractometer [Cu Kα radiation (λ = 1.54187 Å)]. Intensity data were collected using both ω and φ steps or just ω steps, accumulating area detector images spanning at least a hemisphere of reciprocal space. Data for all compounds were collected using CrystalClear[1] and processed (including correction for Lorentz, polarization and absorption) using CrysAlisPro.[2] Structures were solved by dual-space methods (SHELXT[3]) and refined by full-matrix least-squares against F^2 (SHELXL-2018/3[4]). Non-hydrogen atoms were refined anisotropically, and hydrogen atoms were refined using a riding model. Crystals of **18** showed some signs of being slightly polycrystalline, resulting in elevated values of R_{int}, R_1 and wR_2. Attempts to treat the data for twinning were unsuccessful. Crystals of **34** showed weaker diffraction at high angles, and some signs of being slightly polycrystalline, resulting in elevated values of R_{int}, R_1 and wR_2, and a somewhat ambiguous Flack parameter. Attempts to treat the data for twinning were unsuccessful. The same absolute structure was seen across all data-collections, each showing a Flack parameter favouring this structure, but indicating uncertainty about the enantiomeric ratio. However, the presence of a majority of compound of a single absolute structure was confirmed by chiral HPLC, indicating that this absolute structure is correct. All calculations were performed using the Olex2[5] interface. Selected crystallographic data are presented in Tables #-#. CCDC 2217572-2217574 contains the supplementary crystallographic data for this paper. These data can be obtained free of charge from The Cambridge Crystallographic Data Centre via www.ccdc.cam.ac.uk/structures.

References

1. *CrystalClear-SM Exper*t v2.1. Rigaku Americas, *The Woodlands, Texas, USA*, and Rigaku Corporation, *Tokyo, Japan*, 2015.
2. *CrysAlisPro* v1.171.38.46. Rigaku Oxford Diffraction, Rigaku Corporation, *Oxford, U.K.*, 2015.
3. Sheldrick, G. M. SHELXT – Integrated space-group and crystal structure determination. *Acta Crystallogr., Sect. A: Found. Adv.* **2015**, *71*, 3-8. doi: 10.1107/S2053273314026370
4. Sheldrick, G. M. Crystal structure refinement with SHELXL. *Acta Crystallogr., Sect. C: Struct. Chem.* **2015**, *71*, 3-8. Doi: 10.1107/S2053229614024218
5. Dolomanov, O. V.; Bourhis, L. J.; Gildea, R. J.; Howard, J. A. K.; Puschmann, H. OLEX2: a complete structure solution, refinement and analysis program. *J. Appl. Crystallogr*. **2009**, *42*, 339-341. doi: 10.1107/S0021889808042726

	**(3*S*,4*R*)-17**
CDCC	2217572
empirical formula	$C_{13}H_{10}ClF_3O_2$
crystal size [mm]	0.27×0.12×0.01
fw	290.66
crystal description	Colourless plate
Temperature [K]	173
space group	$P2_12_12_1$
a [Å]	6.01417(18)
b [Å]	7.5796(2)
c [Å]	27.9389(8)
vol [Å]^3	1273.59(6)
Z	4
ρ (calc) [g/cm^3]	1.516
μ [mm^{-1}]	2.989
F(000)	592.0
reflections collected	16870
independent reflections (R_{int})	2313 (0.0475)
parameters/restraints	173/0
GOF on F^2	1.052
R_1 [I>2σ(I)]	0.0320
wR_2 (all data)	0.0864
largest diff. peak/hole [e/ Å^3]	0.157, -0.372
Flack parameter	-0.014(7)

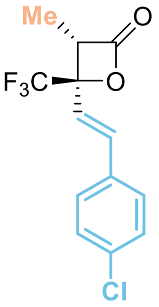

(3*S*,4*R*)-17

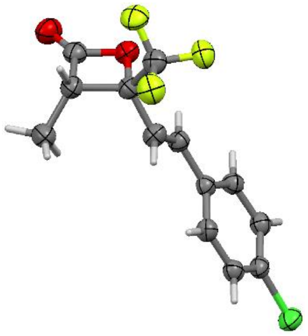

(3*S*,4*R*)-**17**, 50% probability level

	**(3*S*,4*R*)-18**
CDCC	2217573
empirical formula	$C_{13}H_{10}BrF_3O_2$
crystal size [mm]	0.41×0.04×0.01
fw	335.11
crystal description	Colourless plate
Temperature [K]	173
space group	$P2_12_12_1$
a [Å]	7.5922(3)
b [Å]	28.4267(15)
c [Å]	31.0417(15)
vol [Å]^3	6699.5(5)
Z	20
ρ (calc) [g/cm^3]	1.661
μ [mm^{-1}]	4.501
F(000)	3320.0
reflections collected	67285
independent reflections (R_{int})	12199 (0.1769)
parameters/restraints	802/695
GOF on F^2	1.794
R_1 [I>2σ(I)]	0.1522
wR_2 (all data)	0.4063
largest diff. peak/hole [e/ Å^3]	2.362, -0.979
Flack parameter	-0.095(19)

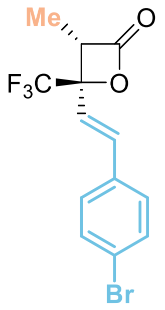

(3*S*,4*R*)-18

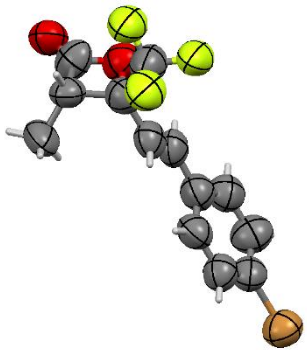

(3*S*,4*R*)-**18**, 50% probability level

	**(3*S*,4*S*)-34**
CDCC	2217574
empirical formula	$C_{15}H_{15}F_3O_3$
crystal size [mm]	0.06×0.04×0.02
fw	300.27
crystal description	Colourless plate
Temperature [K]	125
space group	$P2_1$
a [Å]	7.8378(5)
b [Å]	6.3453(3)
c [Å]	28.666(2)
vol [Å]^3	1418.47(15)
Z	4
ρ (calc) [g/cm^3]	1.406
μ [mm^{-1}]	1.062
F(000)	624.0
reflections collected	15851
independent reflections (R_{int})	5530 (0.1096)
parameters/restraints	386/1
GOF on F^2	1.035
R_1 [I>2σ(I)]	0.0823
wR_2 (all data)	0.2560
largest diff. peak/hole [e/ Å^3]	0.341, -0.370
Flack parameter	0.1(2)

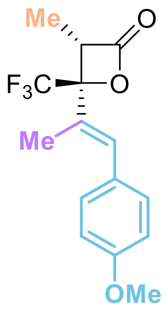

(3*S*,4*S*)-34

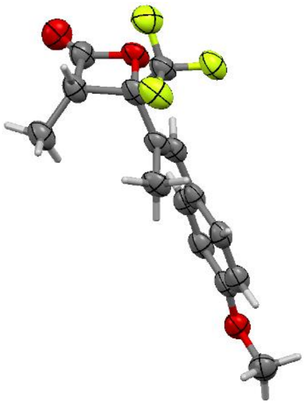

(3*S*,4*S*)-**34**, 50% probability level

Appendix I. 1H, 19F and 13C{1H} NMR Spectra

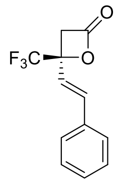

3

^{1}H, $CDCl_3$, 500 MHz

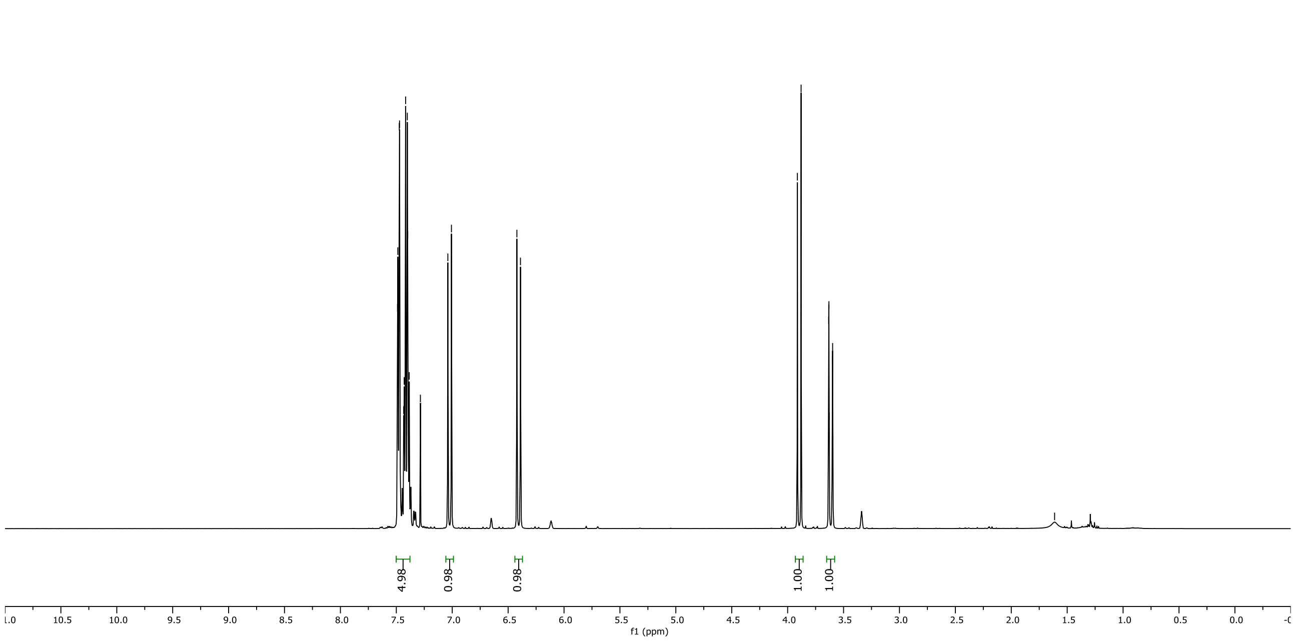

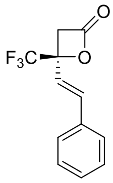

3

^{19}F, $CDCl_3$, 376 MHz

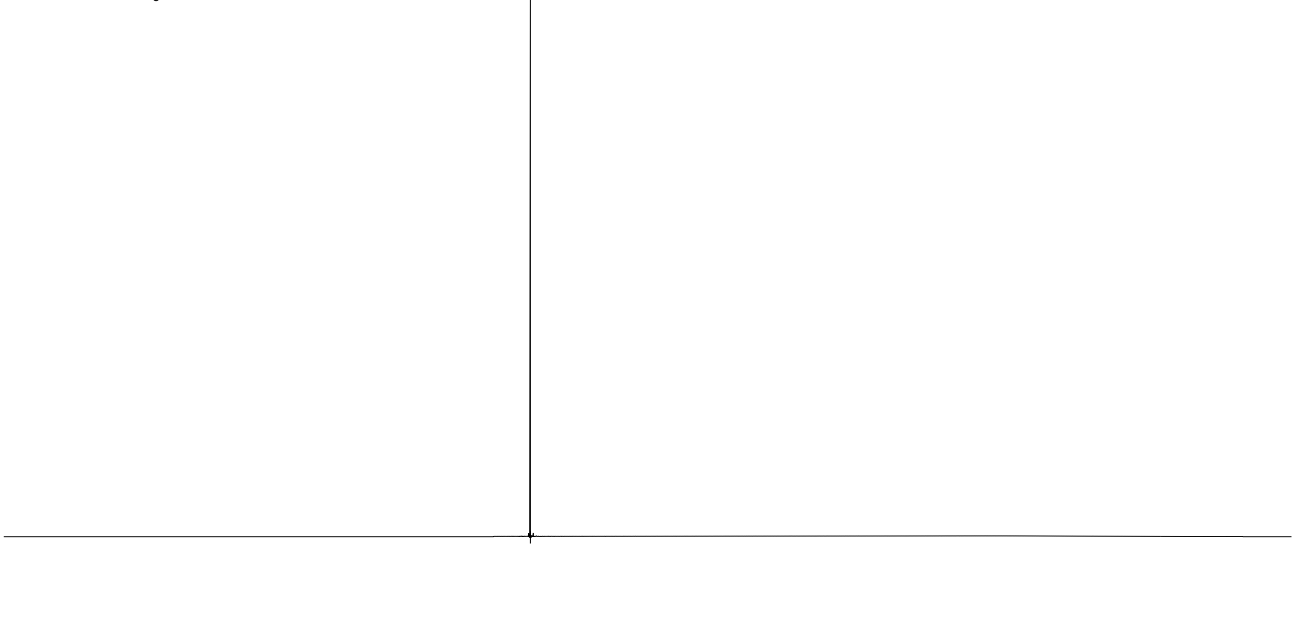

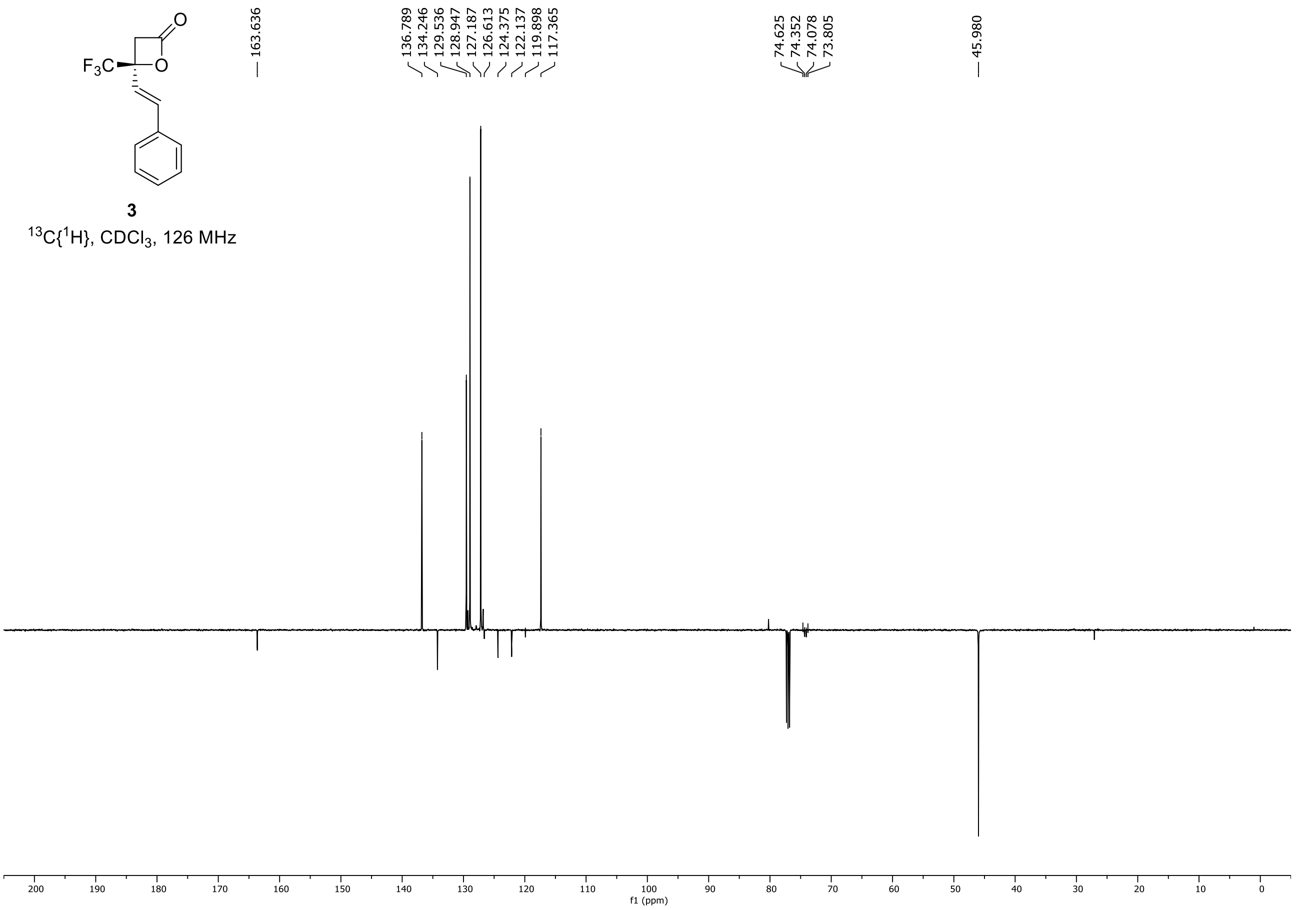
O
F3C
O
3
13C{1H}, CDCl3, 126 MHz
163.636
136.789
134.246
129.536
128.947
127.187
126.613
124.375
122.137
119.898
117.365
74.625
74.352
74.078
73.805
45.980
200 190 180 170 160 150 140 130 120 110 100 90 80 70 60 50 40 30 20 10 0
f1 (ppm)

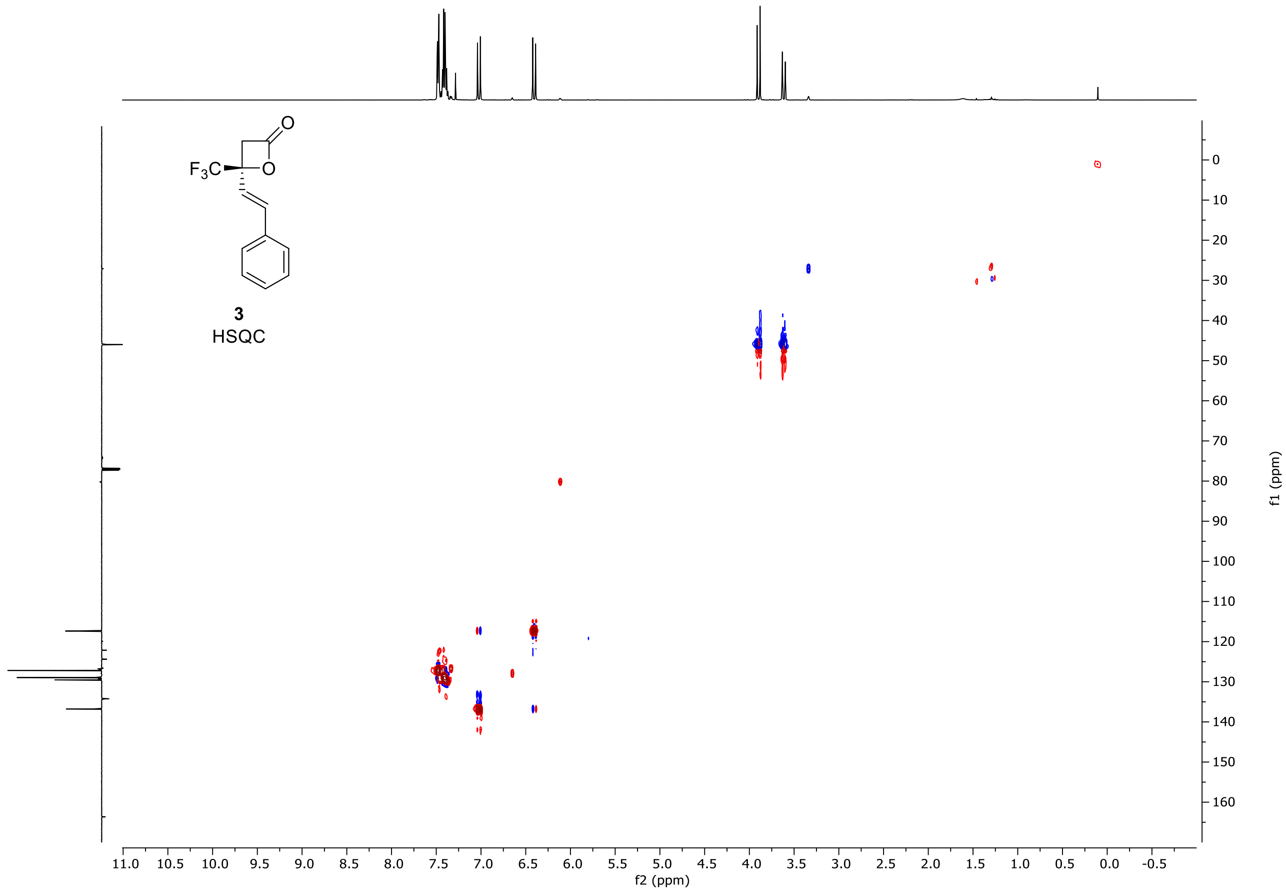
O
F3C
O
3
HSQC
11.0 10.5 10.0 9.5 9.0 8.5 8.0 7.5 7.0 6.5 6.0 5.5 5.0 4.5 4.0 3.5 3.0 2.5 2.0 1.5 1.0 0.5 0.0 -0.5
f2 (ppm)
0 10 20 30 40 50 60 70 80 90 100 110 120 130 140 150 160
f1 (ppm)

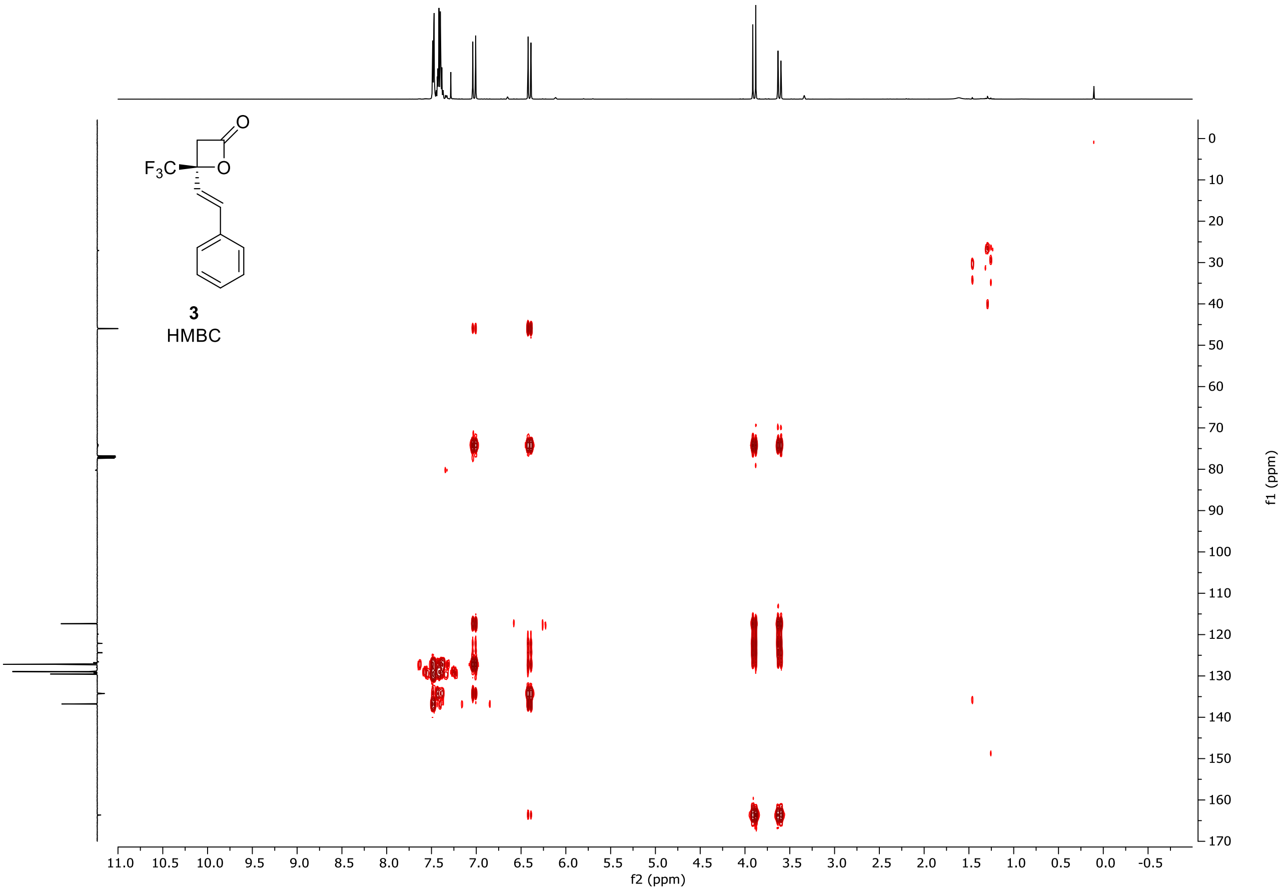

F3C
O
O
3
HMBC
f2 (ppm)
f1 (ppm)

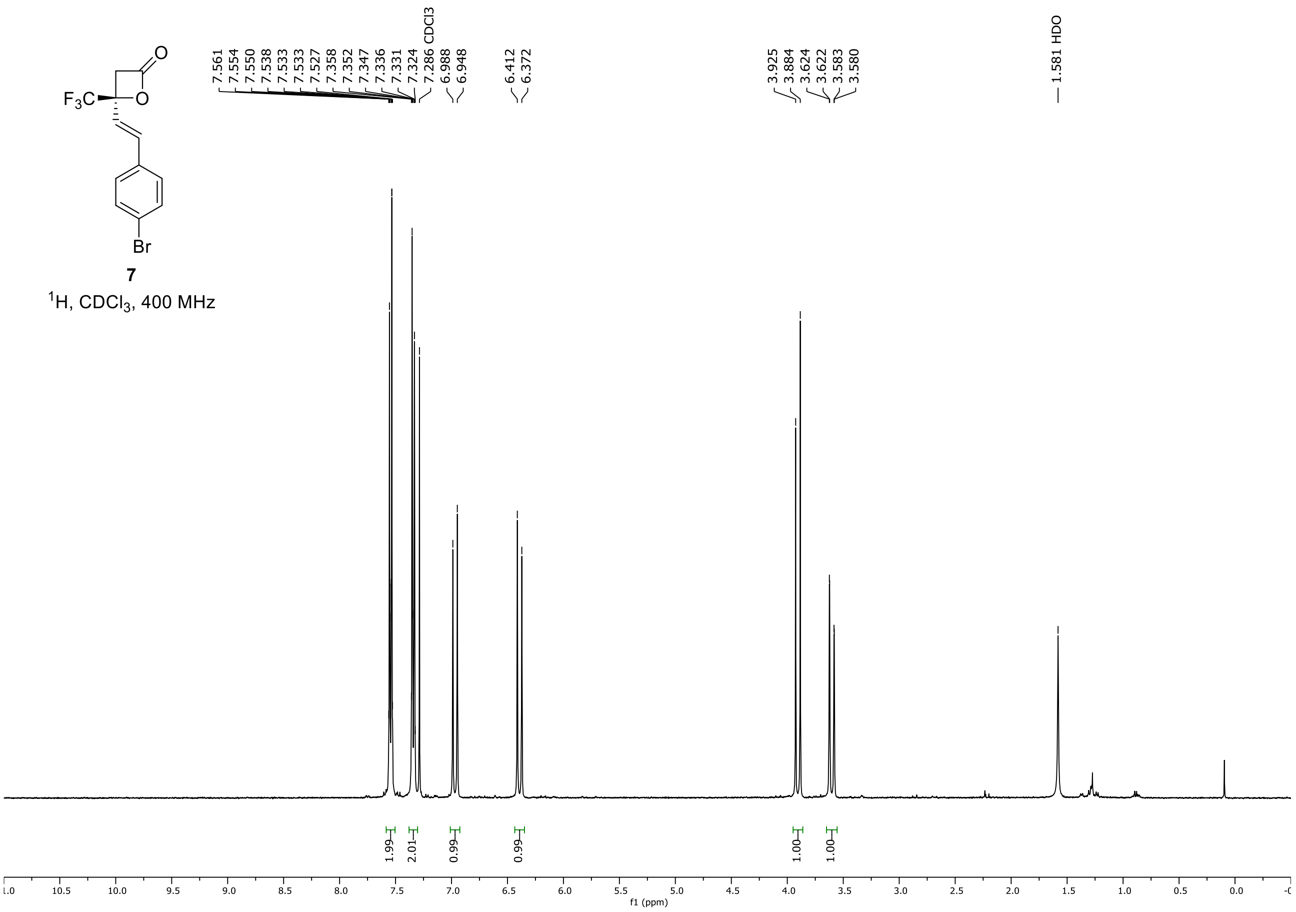

F3C
O
O
Br
7
1H, CDCl3, 400 MHz
7.561
7.554
7.550
7.538
7.533
7.533
7.527
7.358
7.352
7.347
7.336
7.331
7.324
7.286 CDCl3
6.988
6.948
6.412
6.372
3.925
3.884
3.624
3.622
3.583
3.580
1.581 HDO
1.99
2.01
0.99
0.99
1.00
1.00
f1 (ppm)

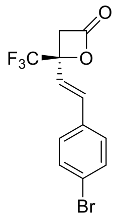

7

^{19}F, $CDCl_3$, 376 MHz

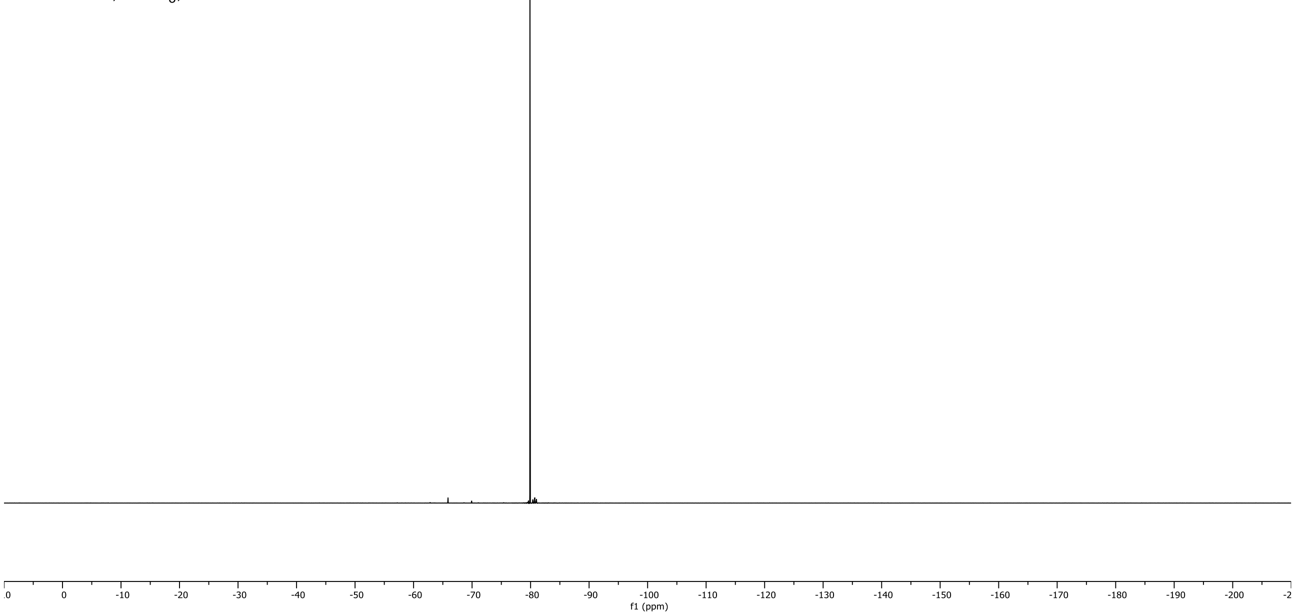

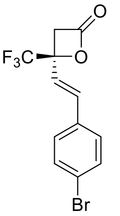

7

$^{13}C\{^1H\}$, $CDCl_3$, 101 MHz

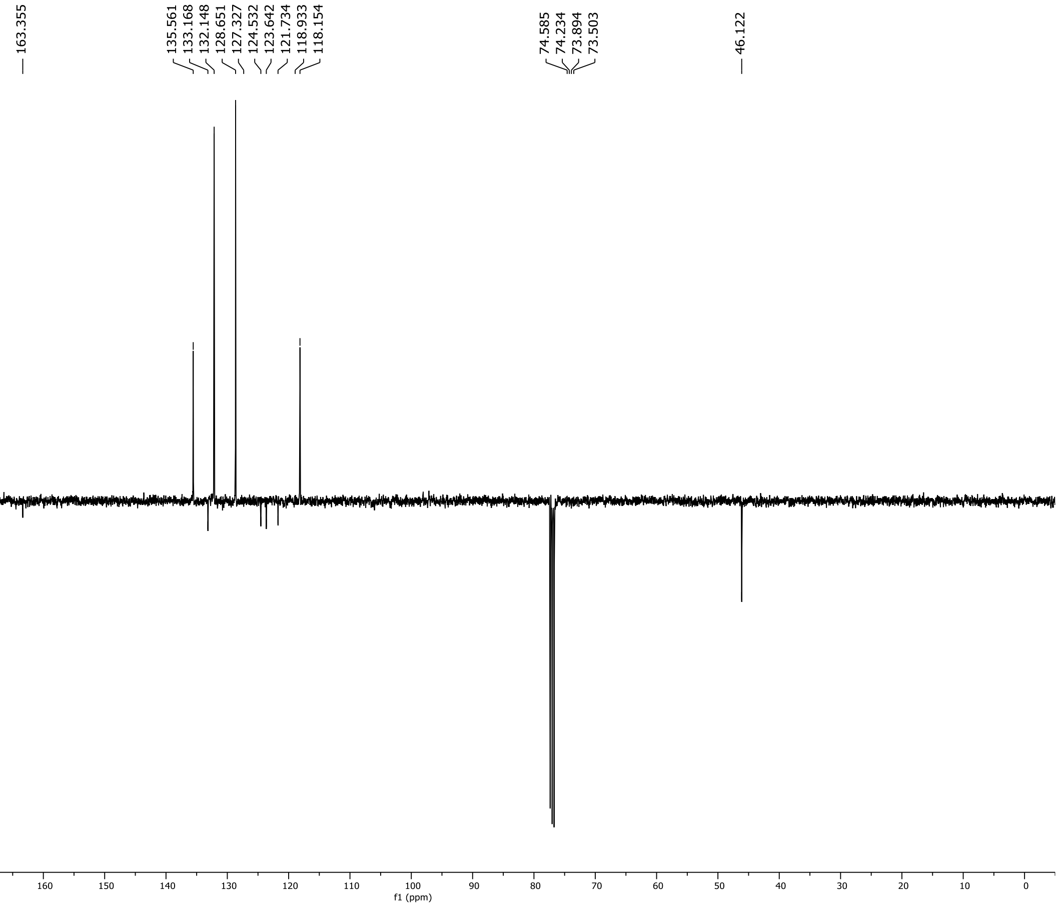

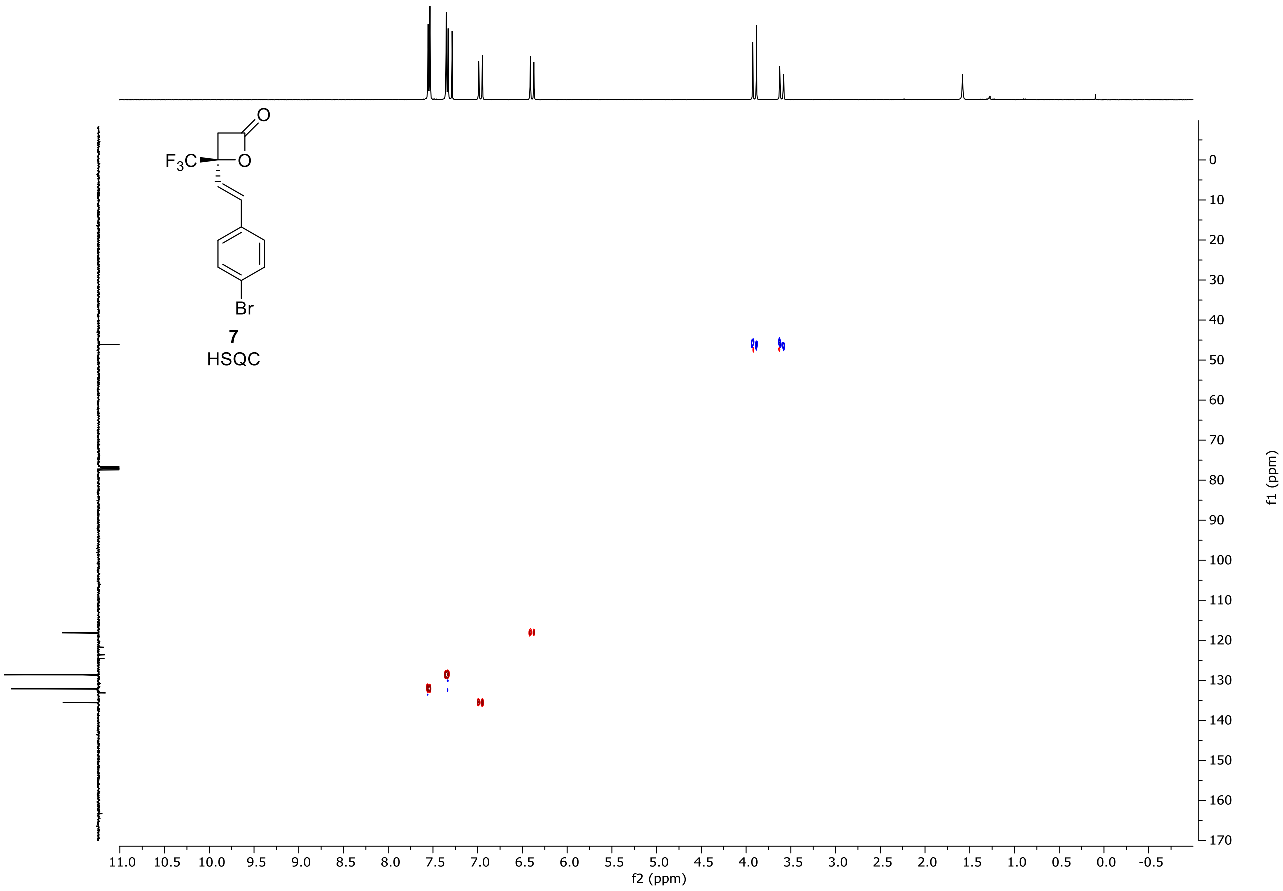
F3C
O
O
Br
7
HSQC
f2 (ppm)
f1 (ppm)

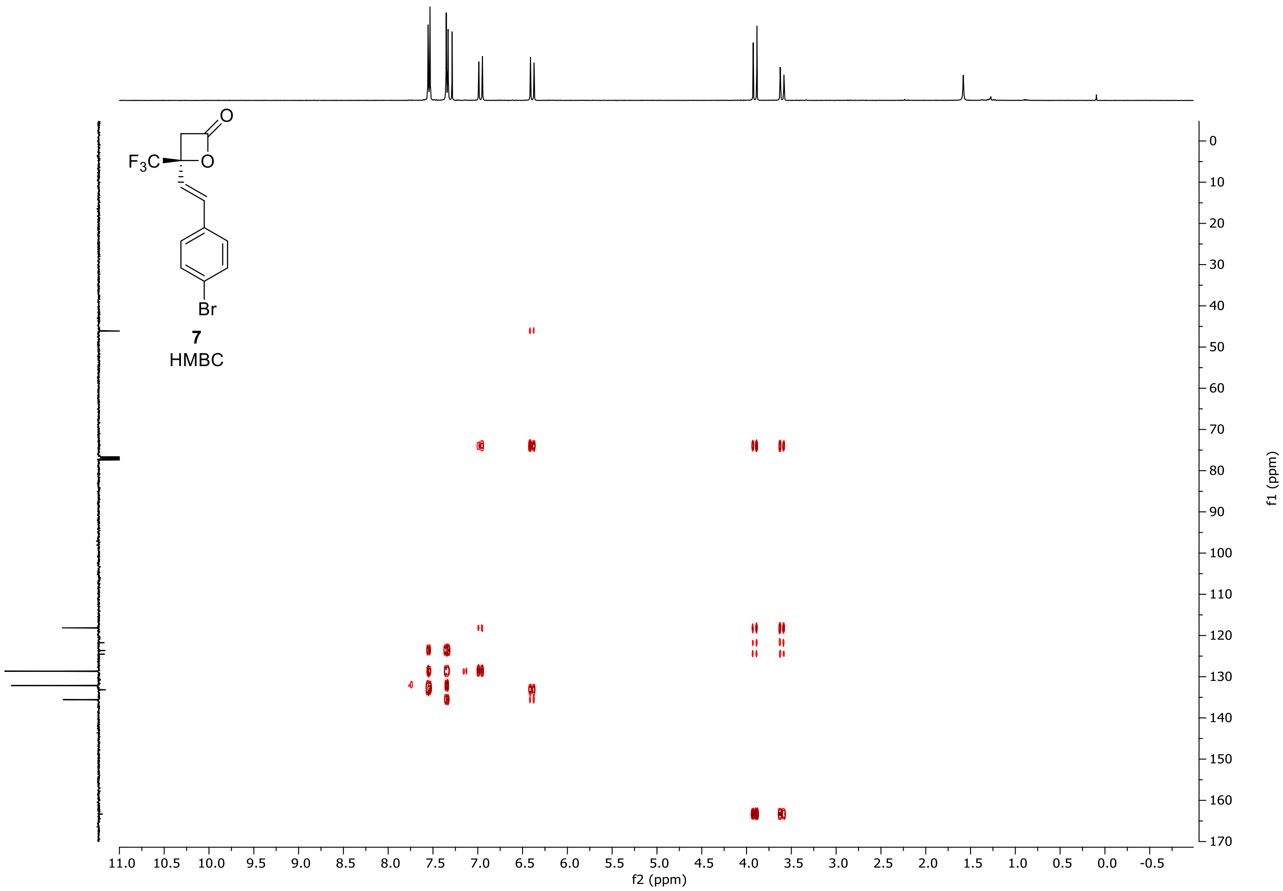
F3C
O
O
Br
7
HMBC
f2 (ppm)
f1 (ppm)

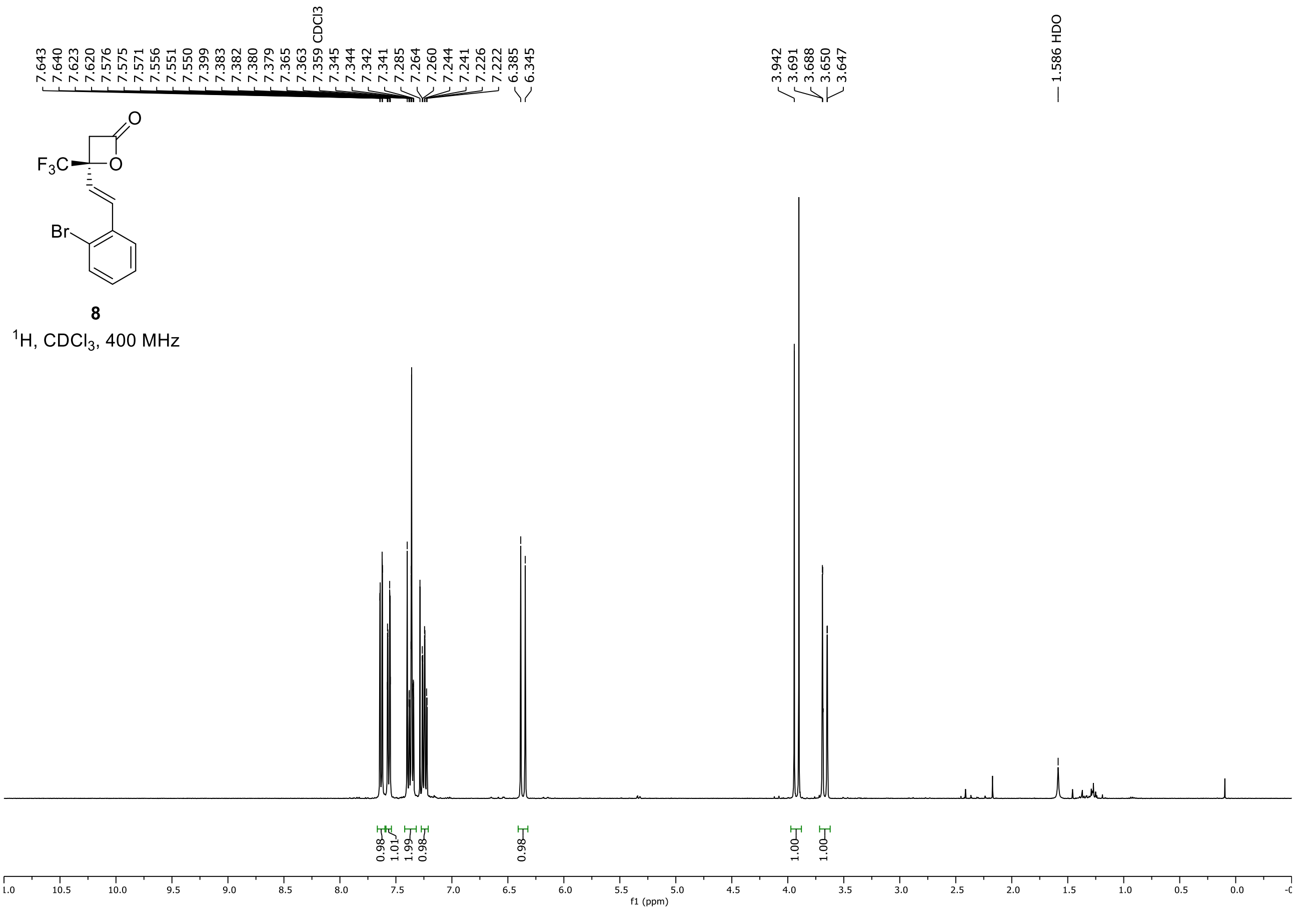
7.643
7.640
7.623
7.620
7.576
7.575
7.571
7.556
7.551
7.550
7.399
7.383
7.382
7.380
7.379
7.365
7.363
7.359 CDCl3
7.345
7.344
7.342
7.341
7.285
7.264
7.260
7.244
7.241
7.226
7.222
6.385
6.345
3.942
3.691
3.688
3.650
3.647
1.586 HDO
F3C
O
O
Br
8
1H, CDCl3, 400 MHz
0.98
1.01
1.99
0.98
0.98
1.00
1.00
f1 (ppm)

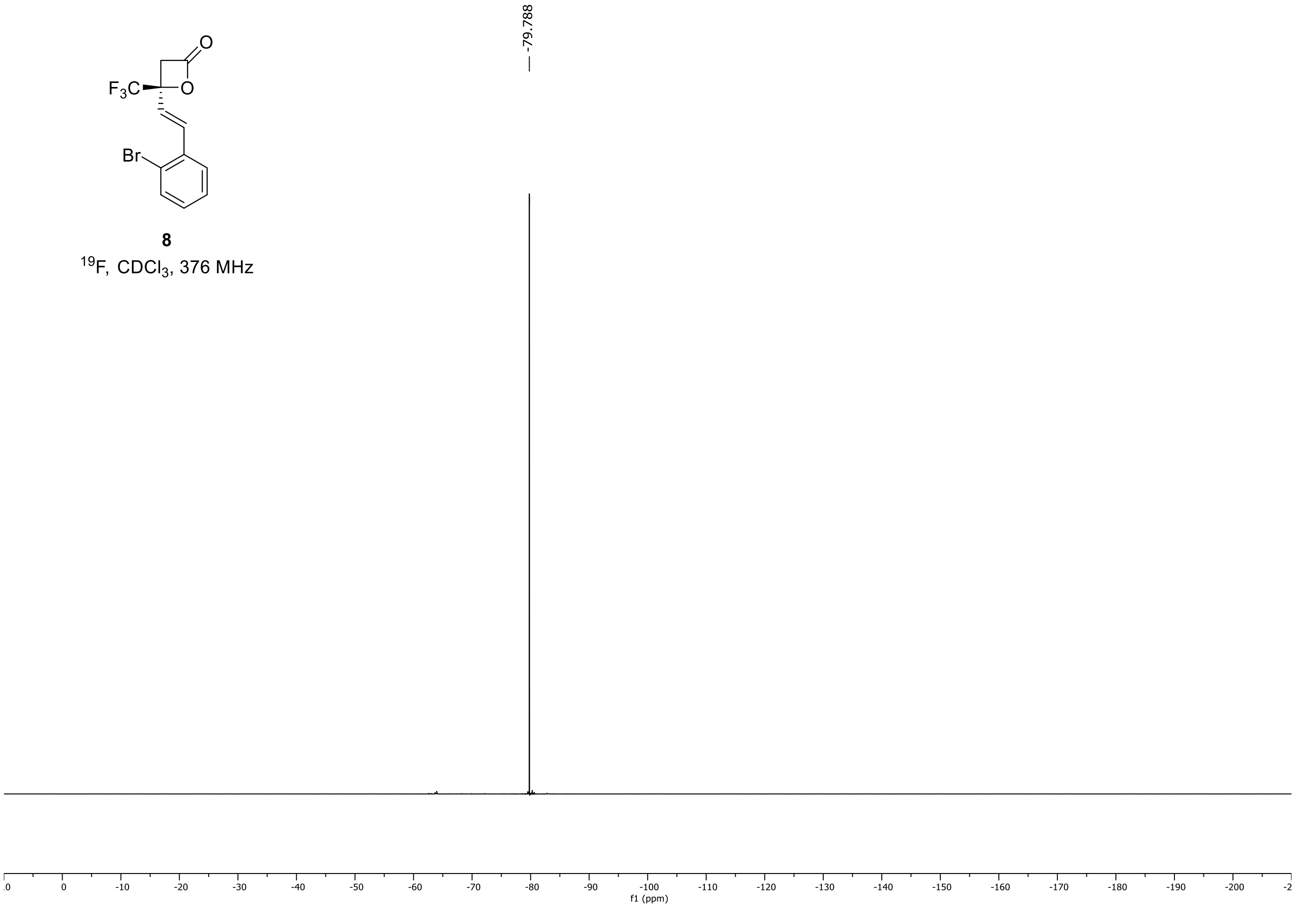
-79.788
F3C
O
O
Br
8
19F, CDCl3, 376 MHz
f1 (ppm)

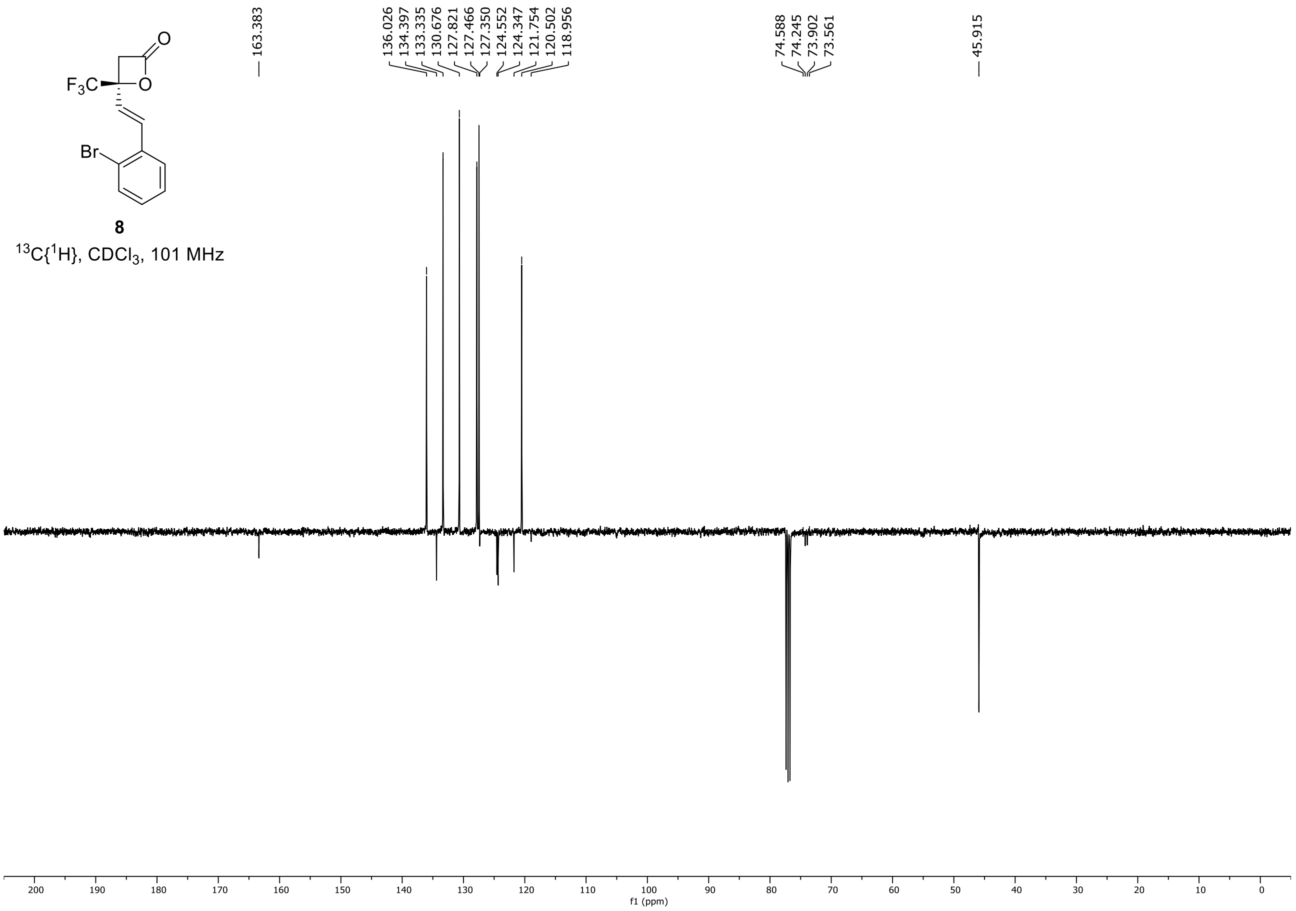
163.383
136.026
134.397
133.335
130.676
127.821
127.466
127.350
124.552
124.347
121.754
120.502
118.956
74.588
74.245
73.902
73.561
45.915
F3C
O
O
Br
8
13C{1H}, CDCl3, 101 MHz
f1 (ppm)

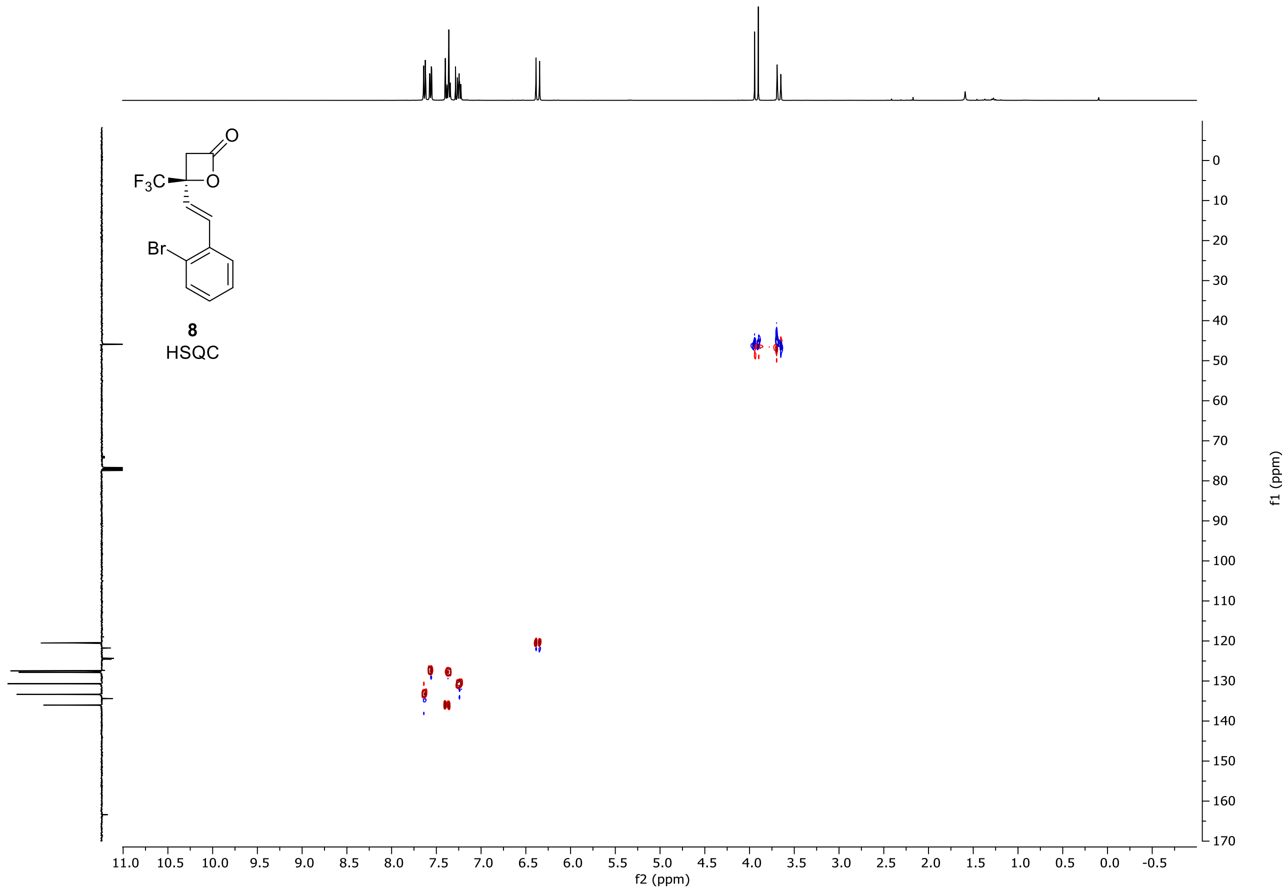
F3C
O
O
Br
8
HSQC
f2 (ppm)
f1 (ppm)

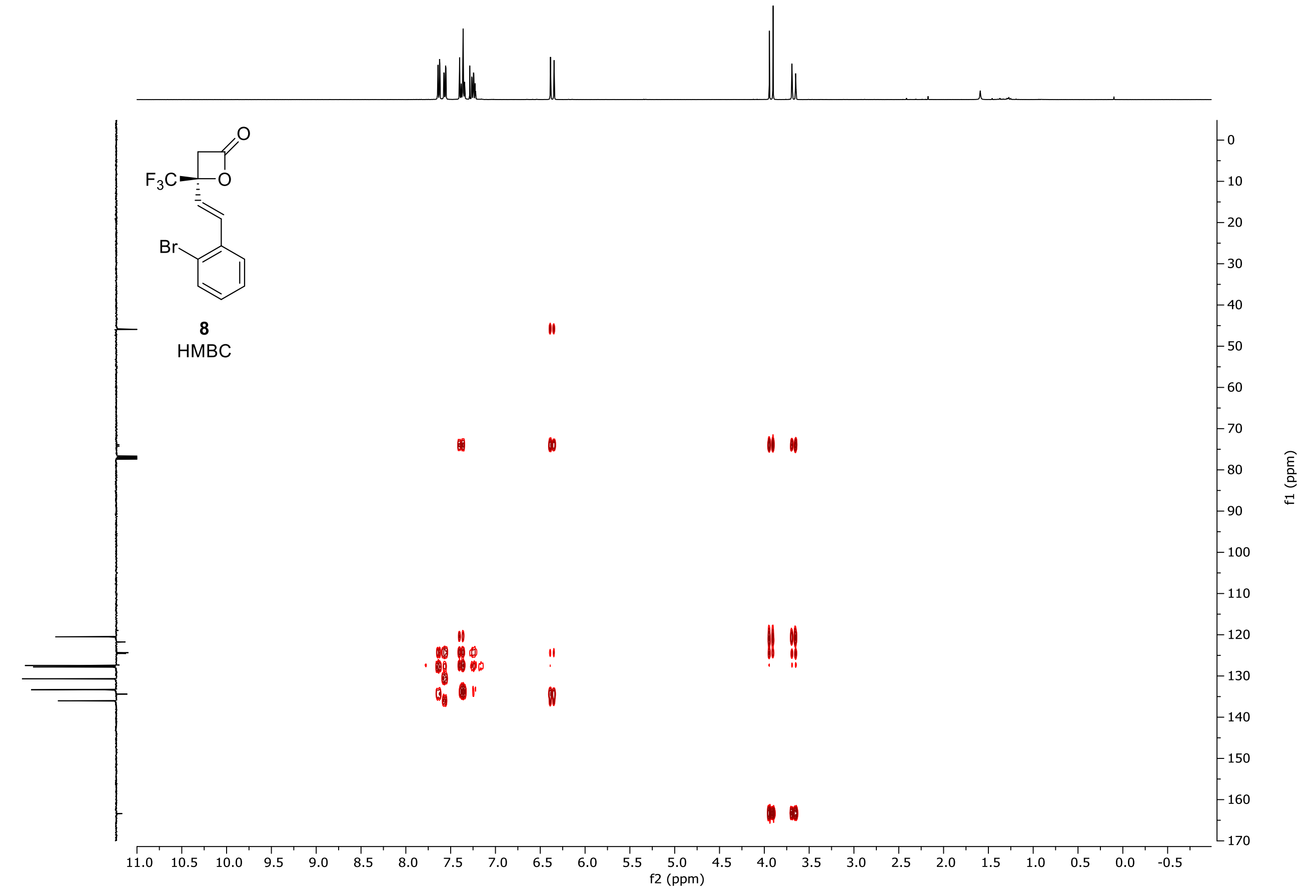
8
HMBC
f2 (ppm)
f1 (ppm)

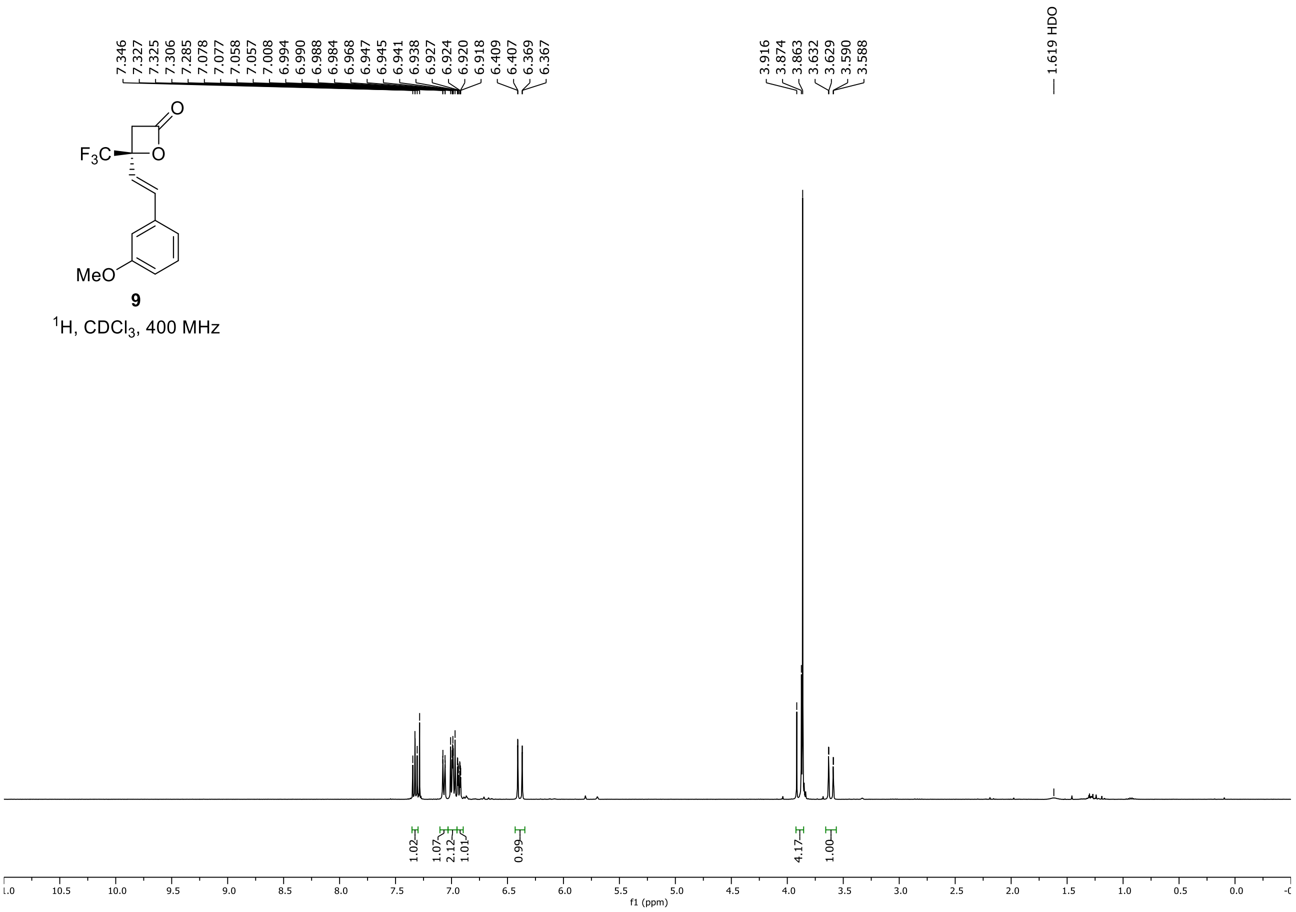
9
1H, CDCl3, 400 MHz
1.619 HDO
f1 (ppm)

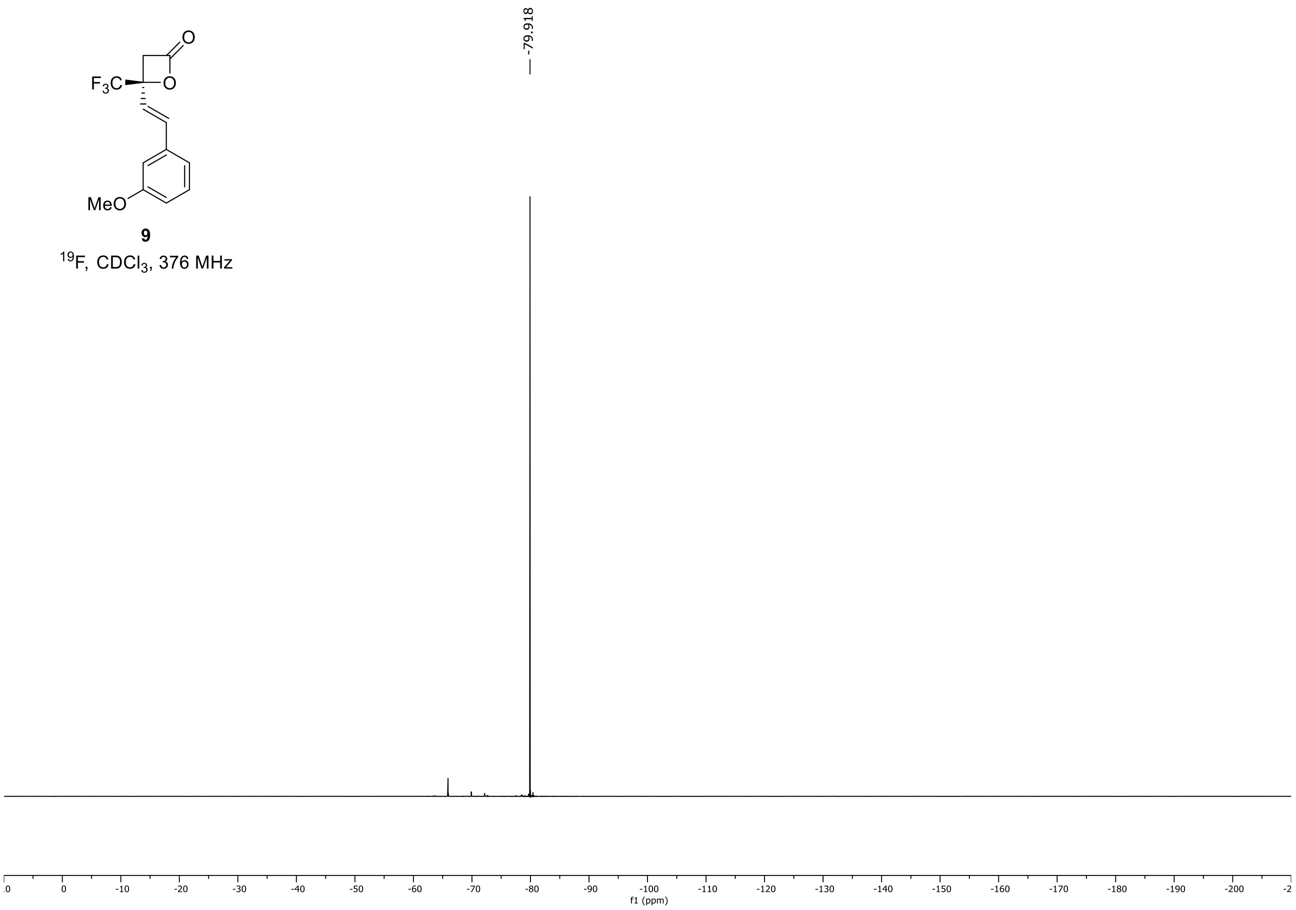
9
^{19}F, CDCl$_3$, 376 MHz
-79.918
f1 (ppm)

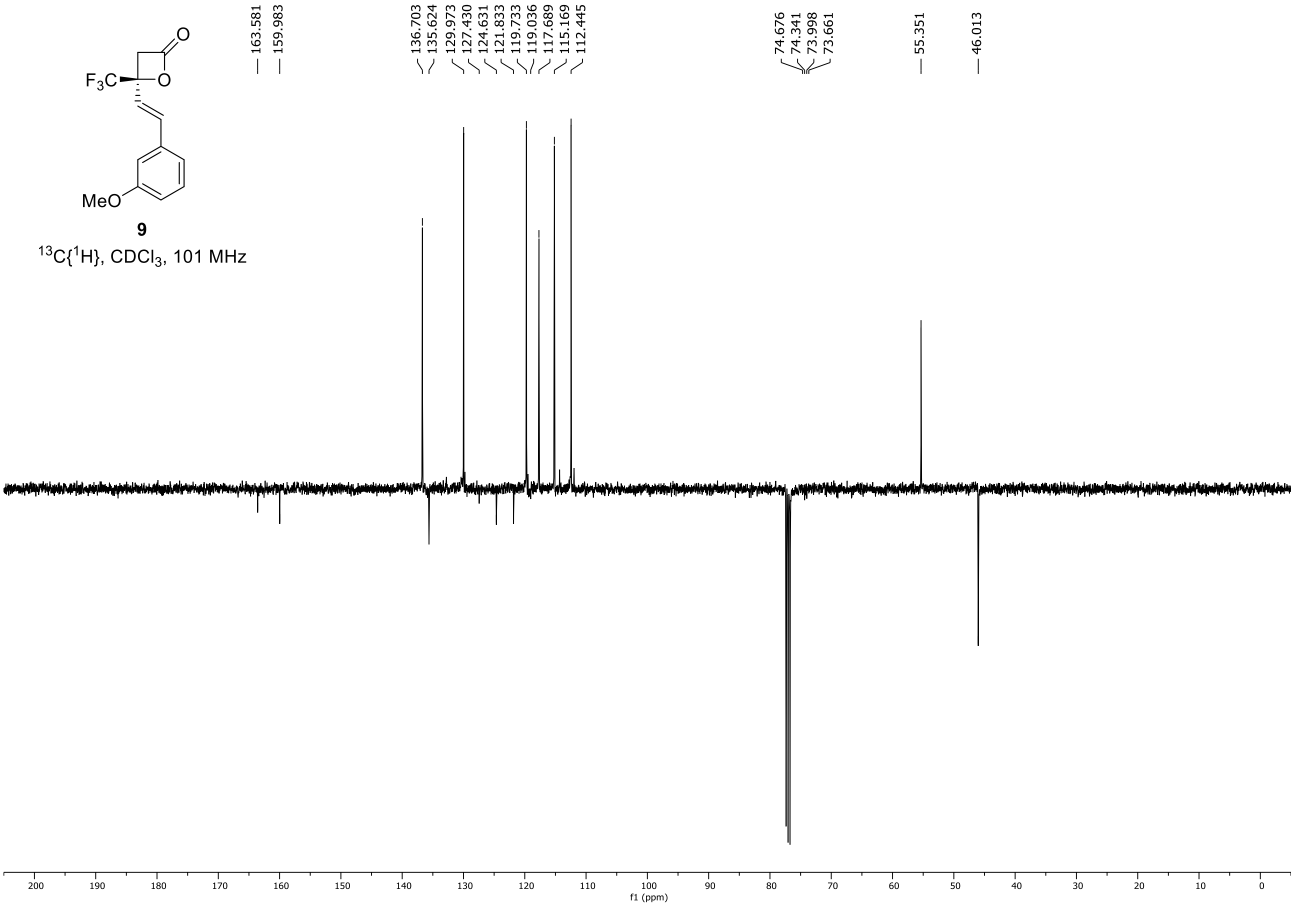
9
^{13}C{^{1}H}, CDCl$_3$, 101 MHz
163.581
159.983
136.703
135.624
129.973
127.430
124.631
121.833
119.733
119.036
117.689
115.169
112.445
74.676
74.341
73.998
73.661
55.351
46.013
f1 (ppm)

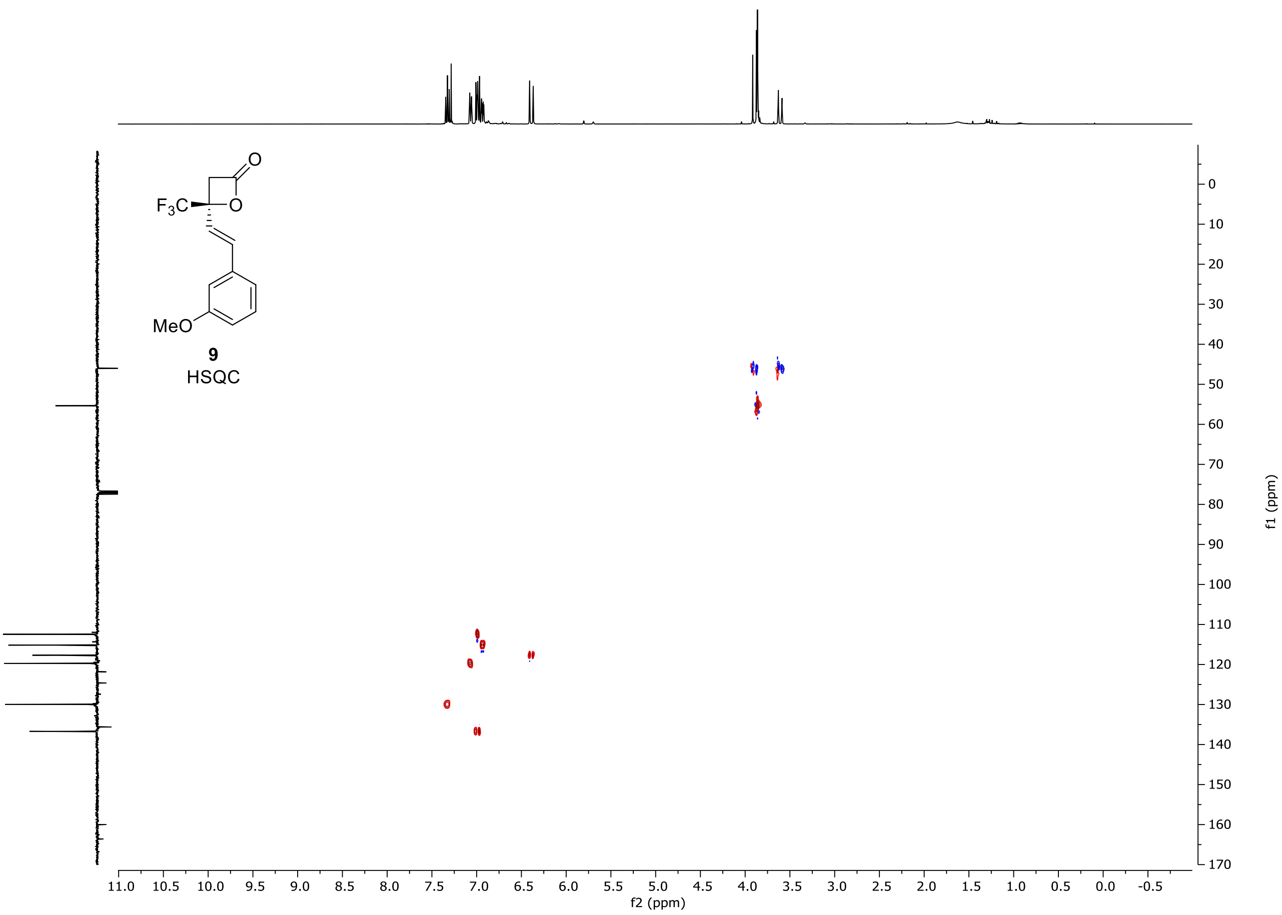
F3C
O
O
MeO
9
HSQC
f1 (ppm)
f2 (ppm)

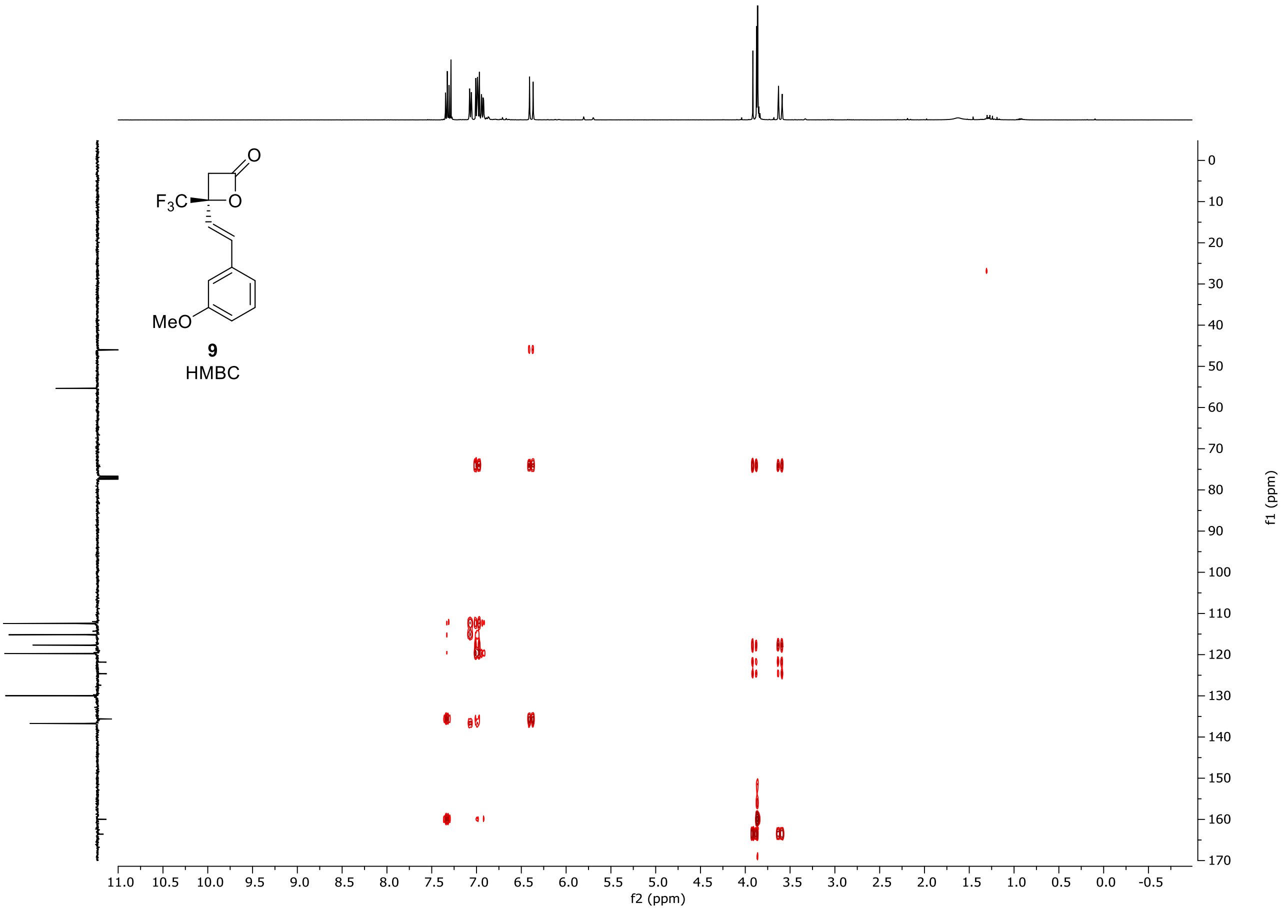
F3C
O
O
MeO
9
HMBC
f1 (ppm)
f2 (ppm)

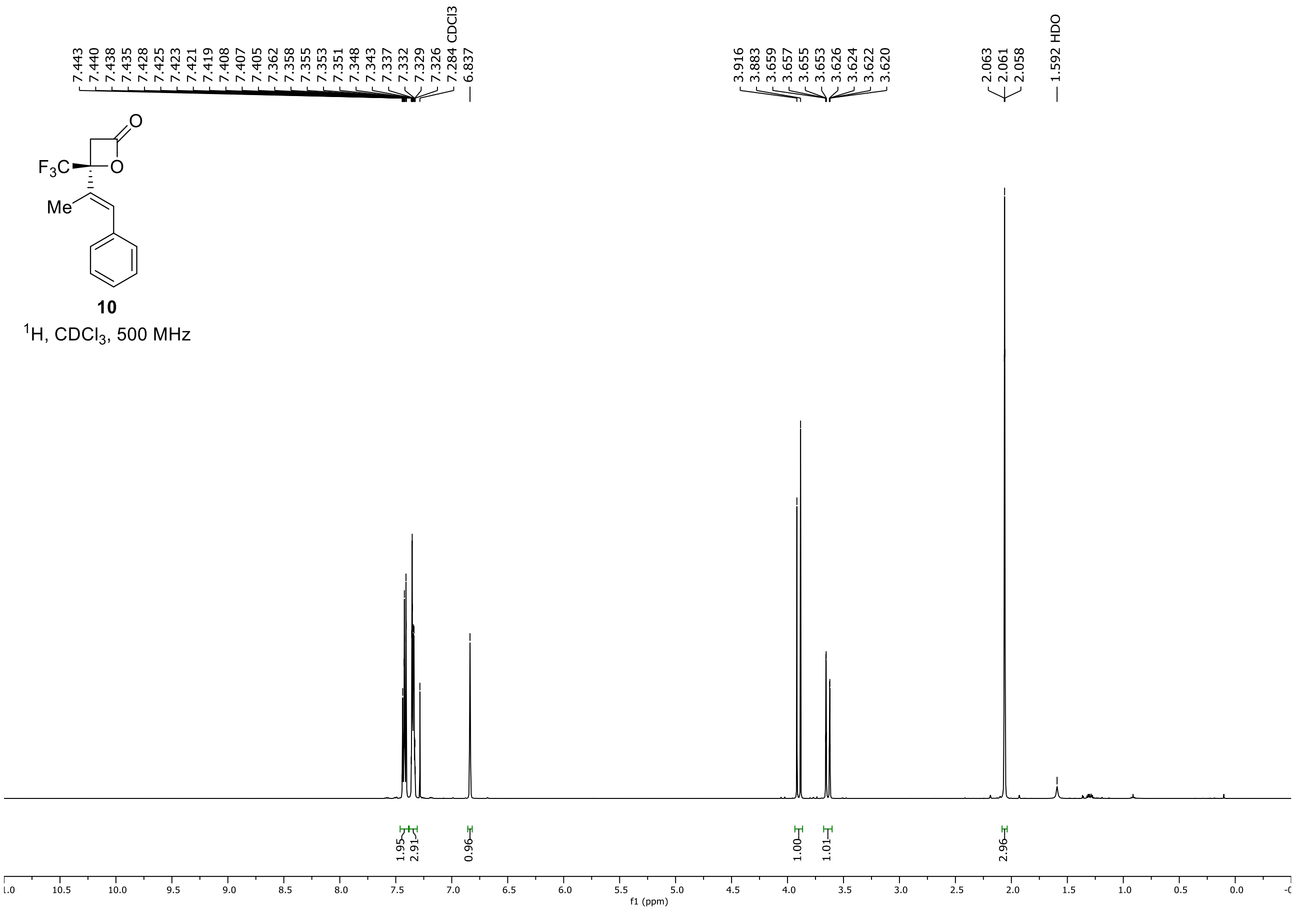

10

^{1}H, $CDCl_3$, 500 MHz

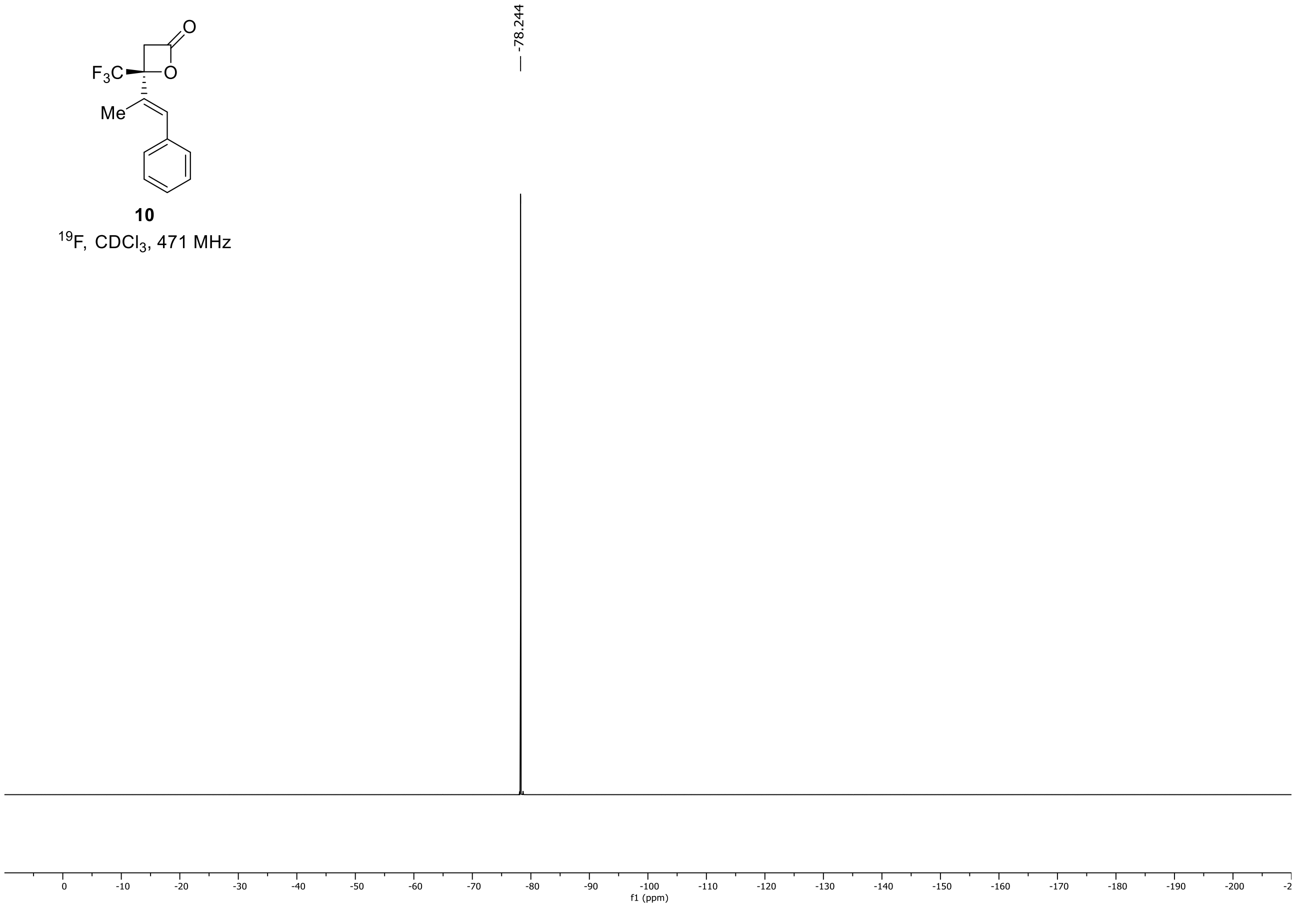

10

^{19}F, $CDCl_3$, 471 MHz

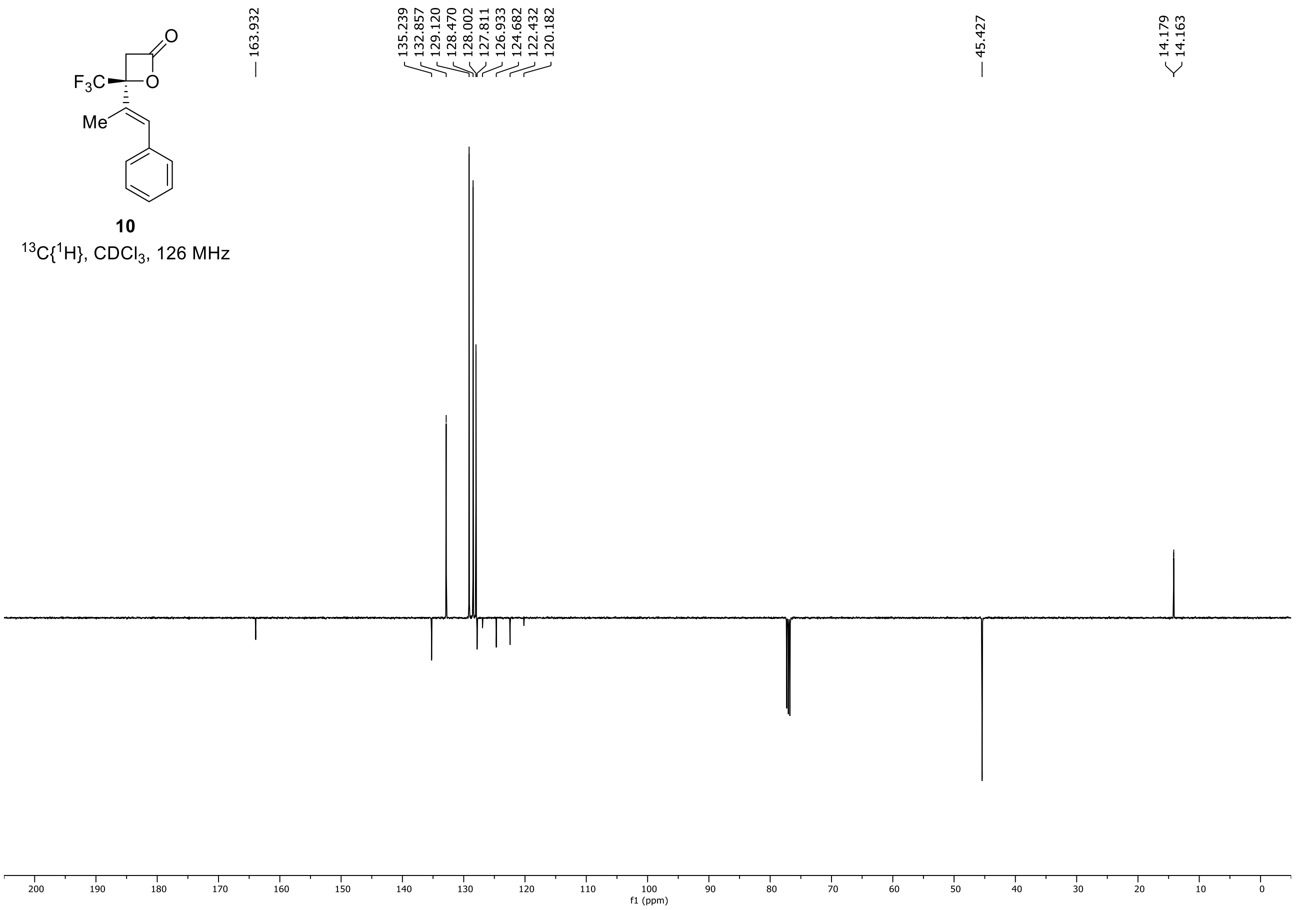
F3C
O
O
Me
10
13C{1H}, CDCl3, 126 MHz
163.932
135.239
132.857
129.120
128.470
128.002
127.811
126.933
124.682
122.432
120.182
45.427
14.179
14.163
f1 (ppm)

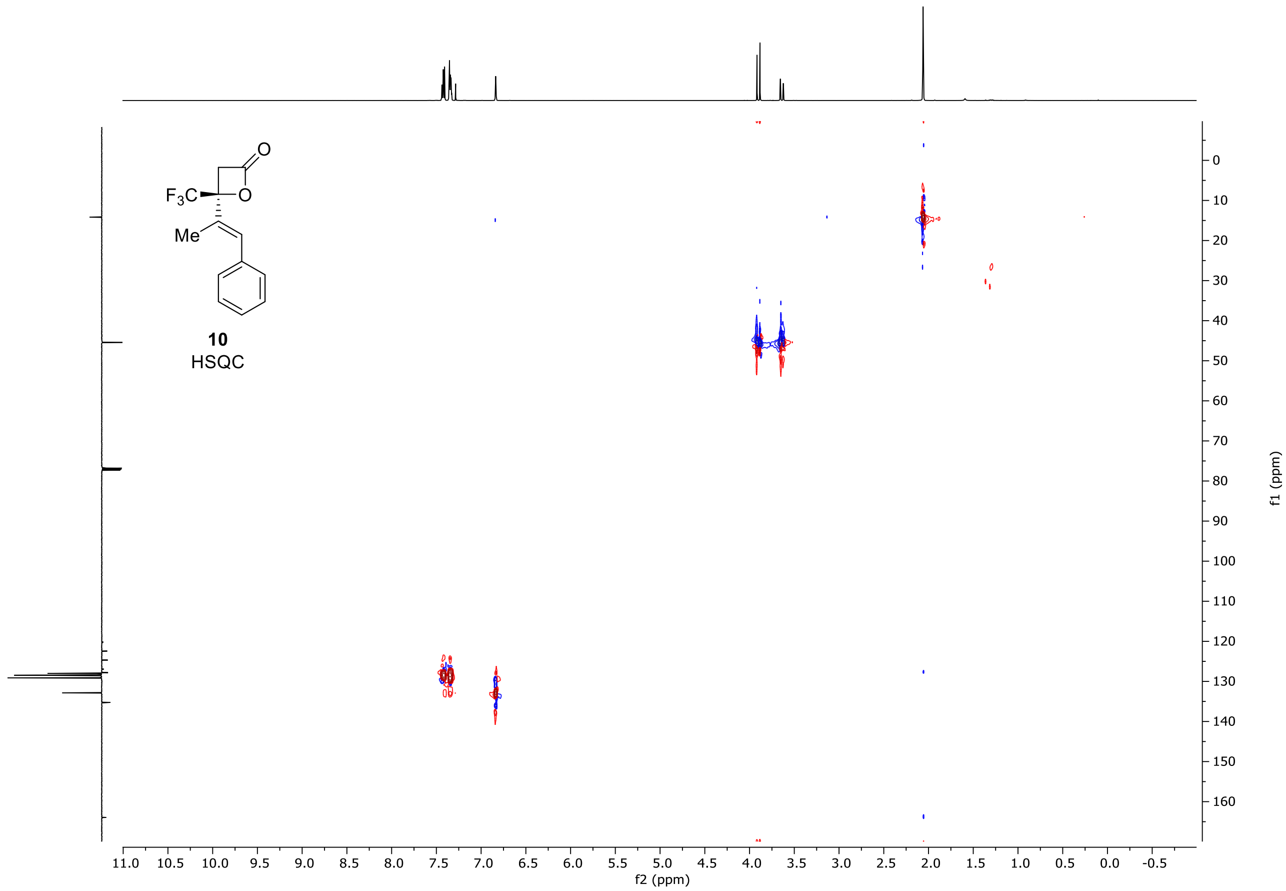
F3C
O
O
Me
10
HSQC
f2 (ppm)
f1 (ppm)

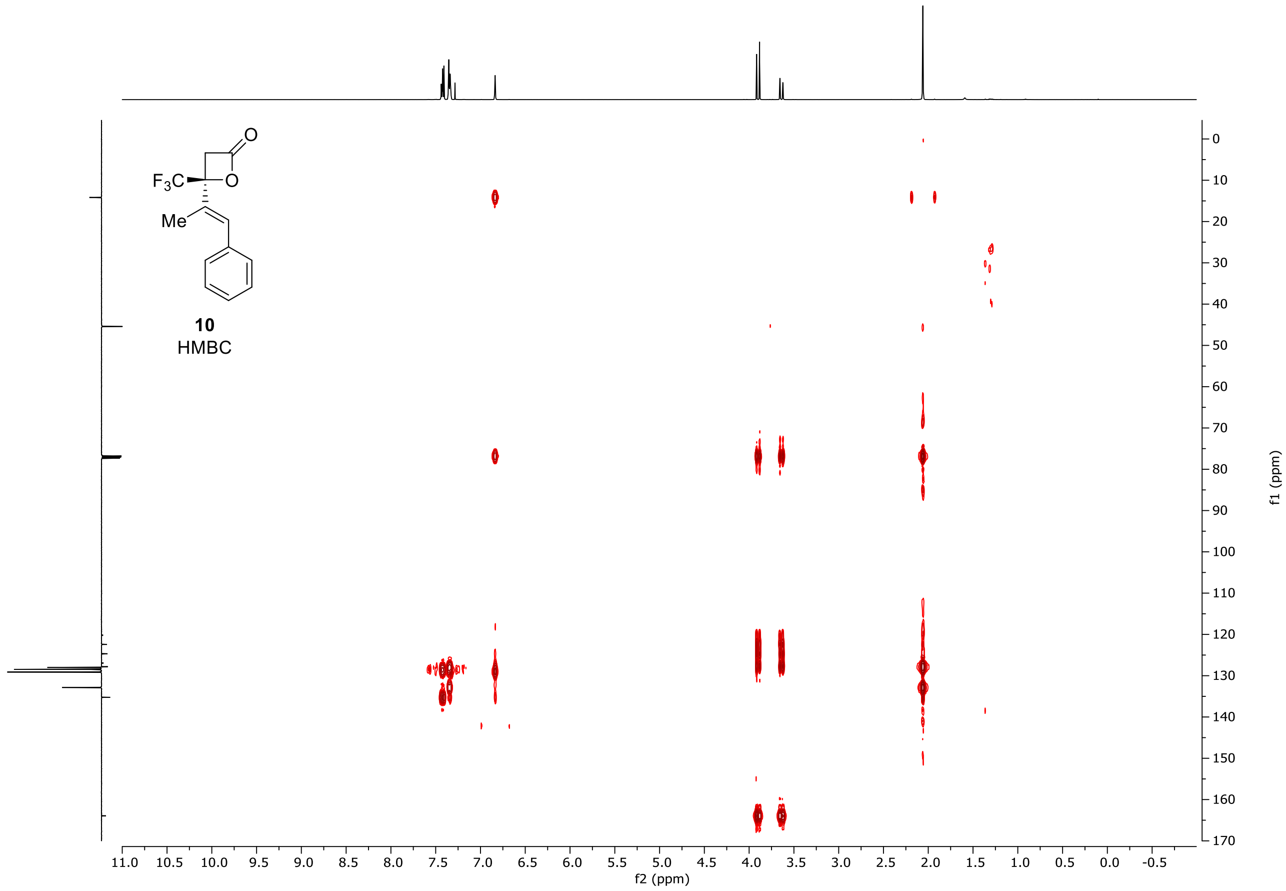
F3C
O
O
Me
10
HMBC
f2 (ppm)
f1 (ppm)

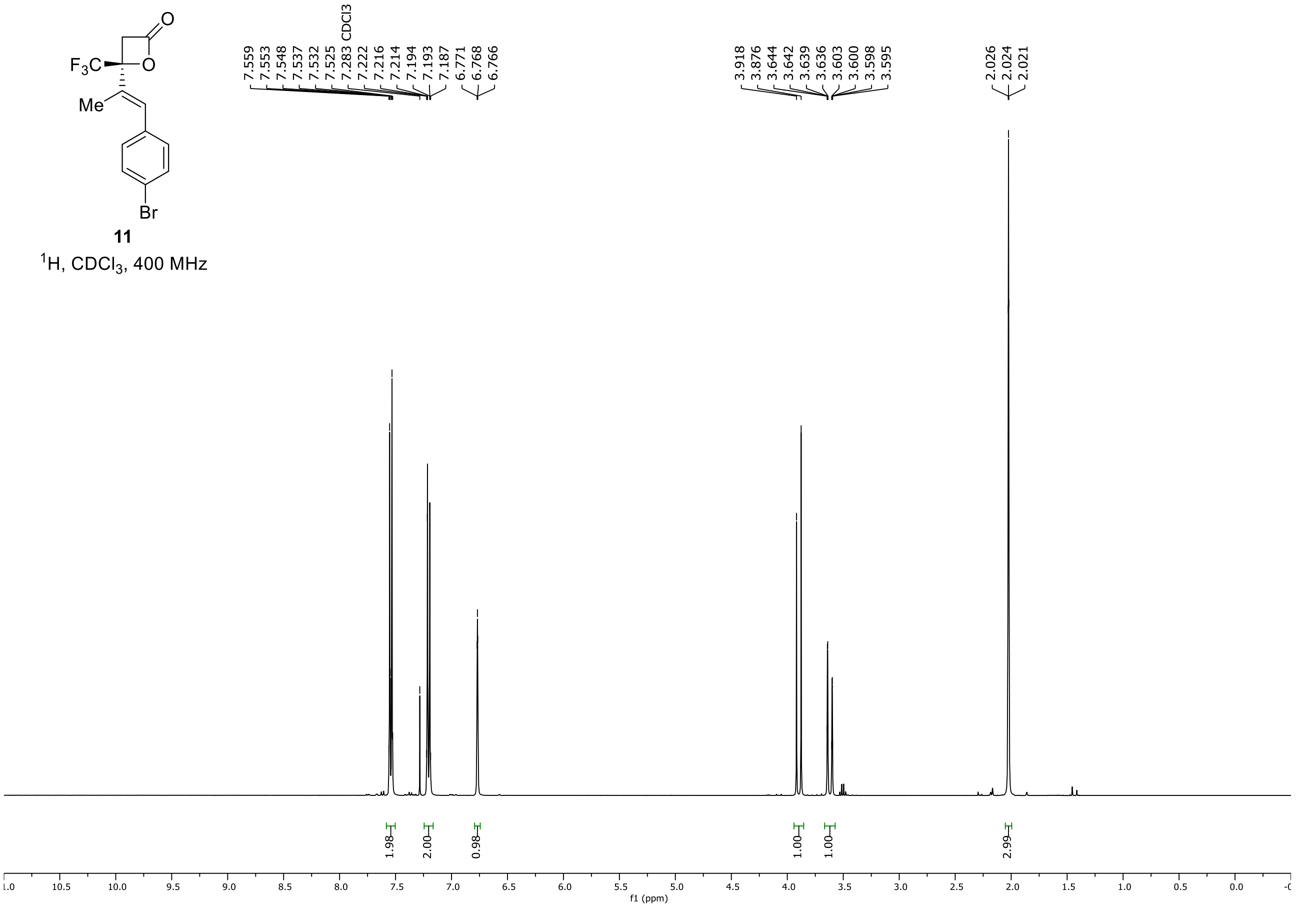
F3C
O
O
Me
Br
11
1H, CDCl3, 400 MHz
7.559
7.553
7.548
7.537
7.532
7.525
7.283 CDCl3
7.222
7.216
7.214
7.194
7.193
7.187
6.771
6.768
6.766
3.918
3.876
3.644
3.642
3.639
3.636
3.603
3.600
3.598
3.595
2.026
2.024
2.021
1.98
2.00
0.98
1.00
1.00
2.99
f1 (ppm)

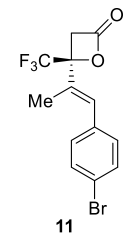

11

^{19}F, $CDCl_3$, 377 MHz

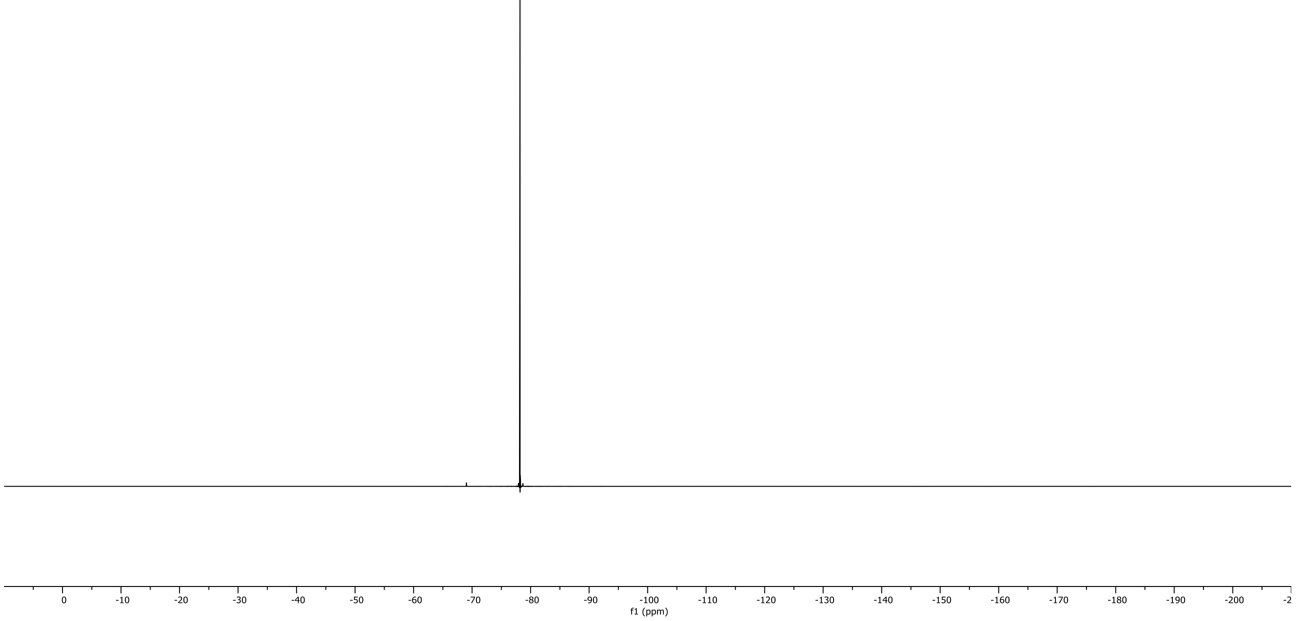

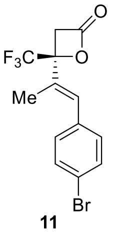

11

$^{13}C\{^1H\}$, $CDCl_3$, 101 MHz

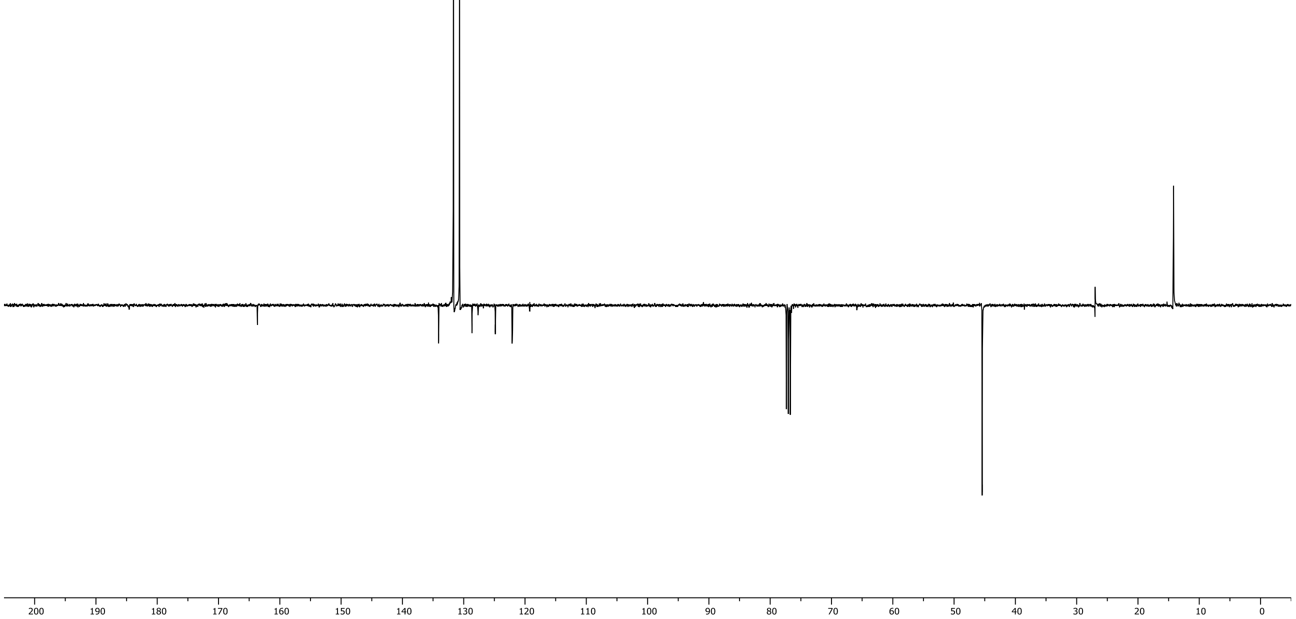

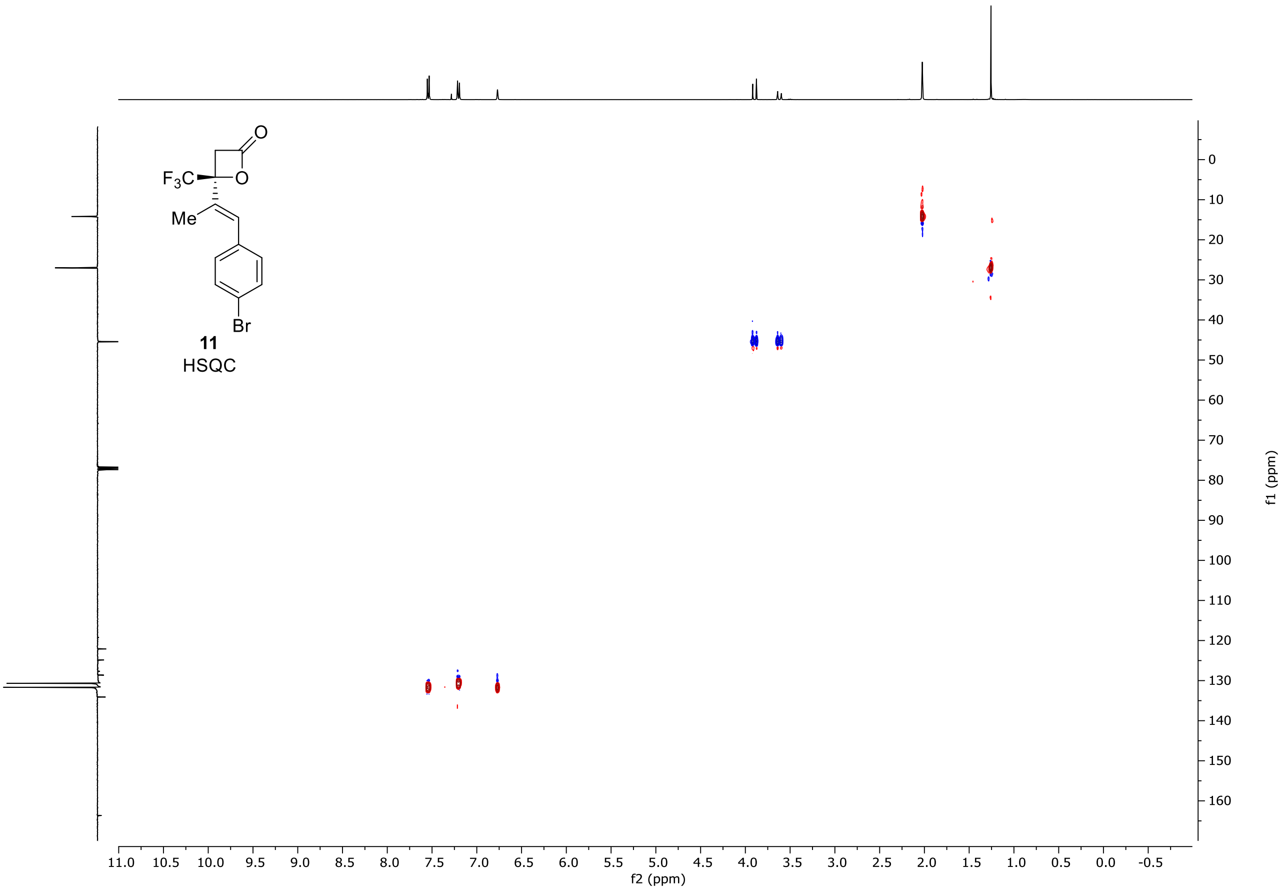
F3C
O
O
Me
Br
11
HSQC
f1 (ppm)
f2 (ppm)

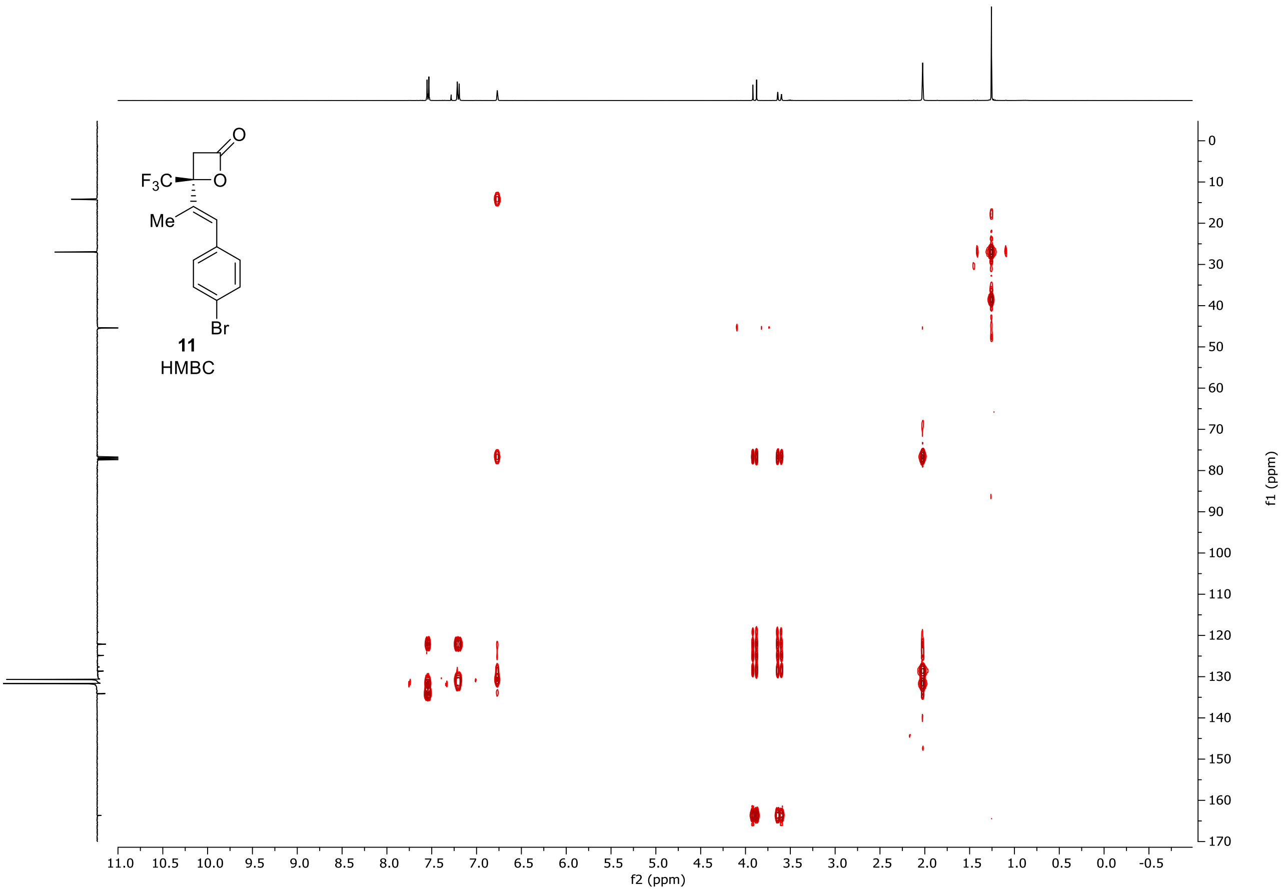
F3C
O
O
Me
Br
11
HMBC
f1 (ppm)
f2 (ppm)

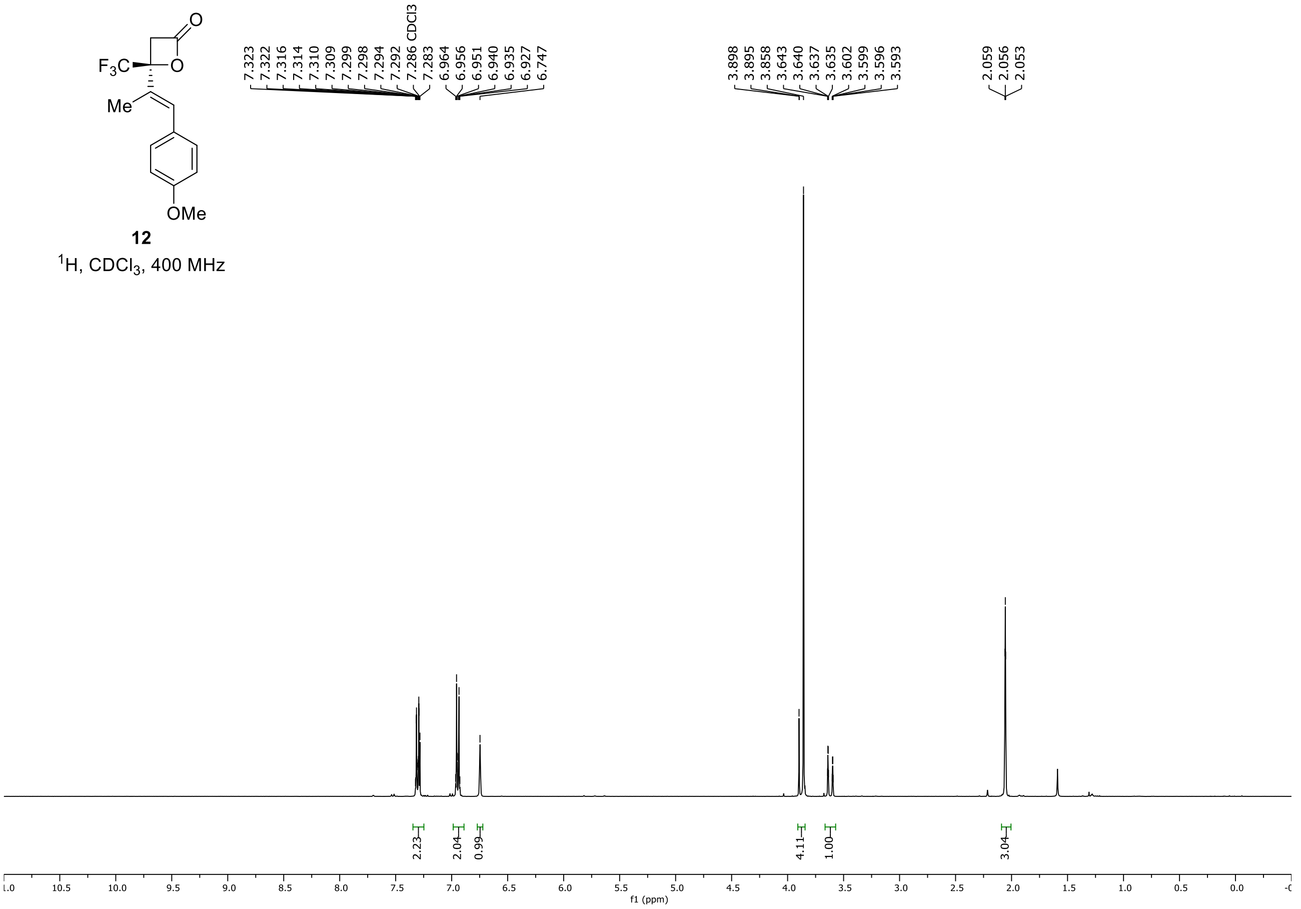
F3C
O
O
Me
OMe
12
1H, CDCl3, 400 MHz
7.323
7.322
7.316
7.314
7.310
7.309
7.299
7.298
7.294
7.292
7.286 CDCl3
7.283
6.964
6.956
6.951
6.940
6.935
6.927
6.747
3.898
3.895
3.858
3.643
3.640
3.637
3.635
3.602
3.599
3.596
3.593
2.059
2.056
2.053
2.23
2.04
0.99
4.11
1.00
3.04
f1 (ppm)

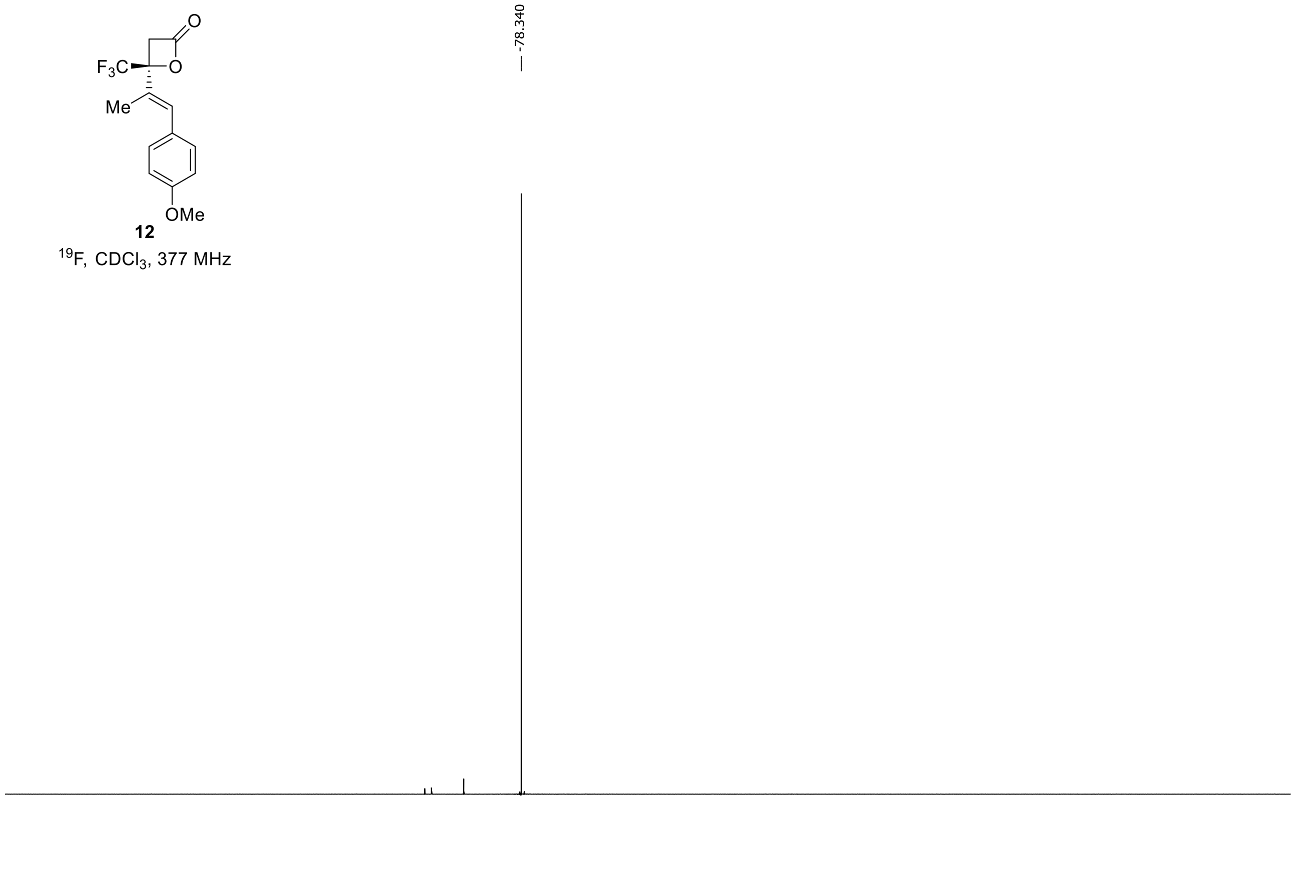
F3C
O
O
Me
OMe
12
19F, CDCl3, 377 MHz
-78.340
f1 (ppm)

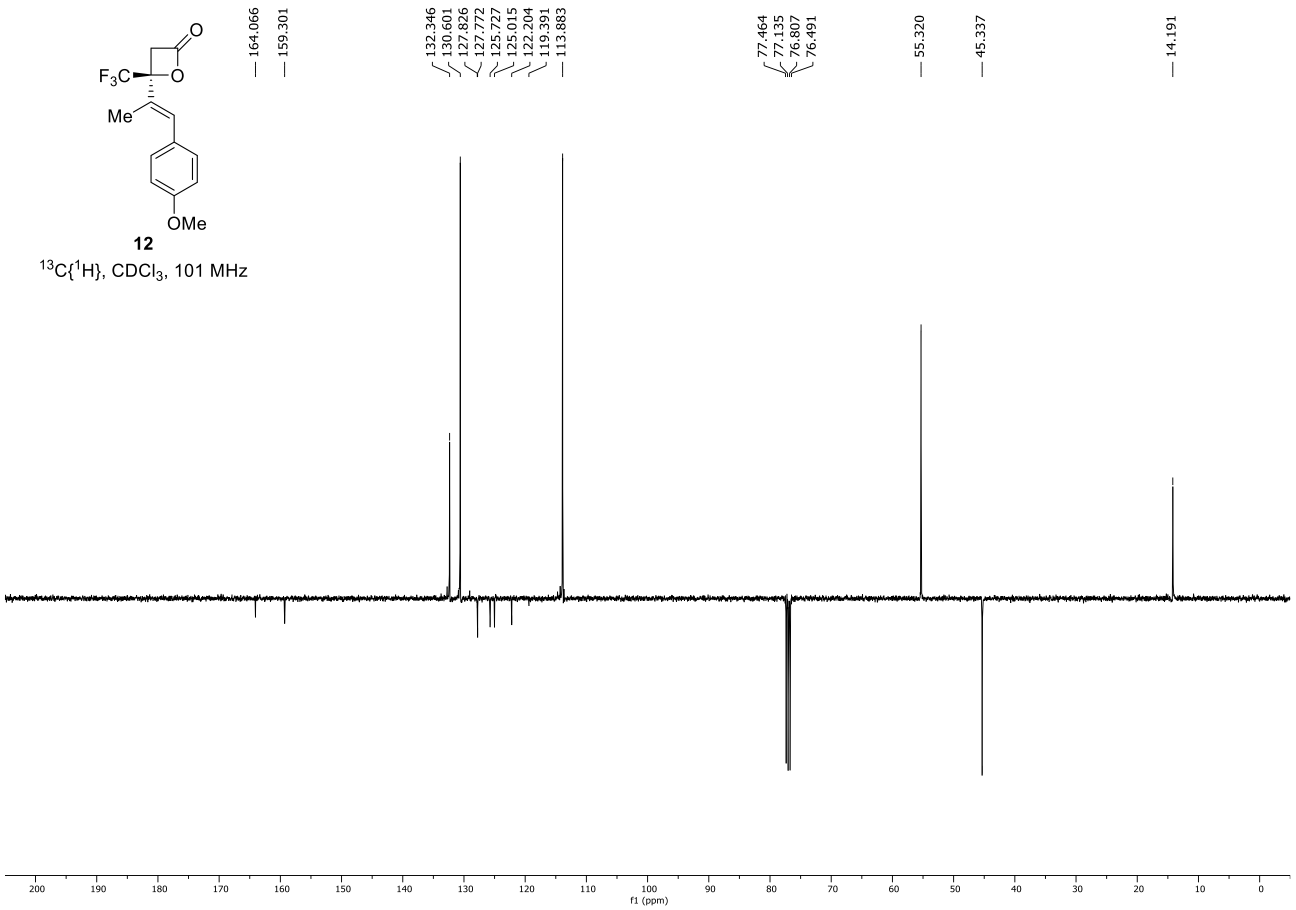
F3C
O
O
Me
OMe
12
13C{1H}, CDCl3, 101 MHz
164.066
159.301
132.346
130.601
127.826
127.772
125.727
125.015
122.204
119.391
113.883
77.464
77.135
76.807
76.491
55.320
45.337
14.191
f1 (ppm)

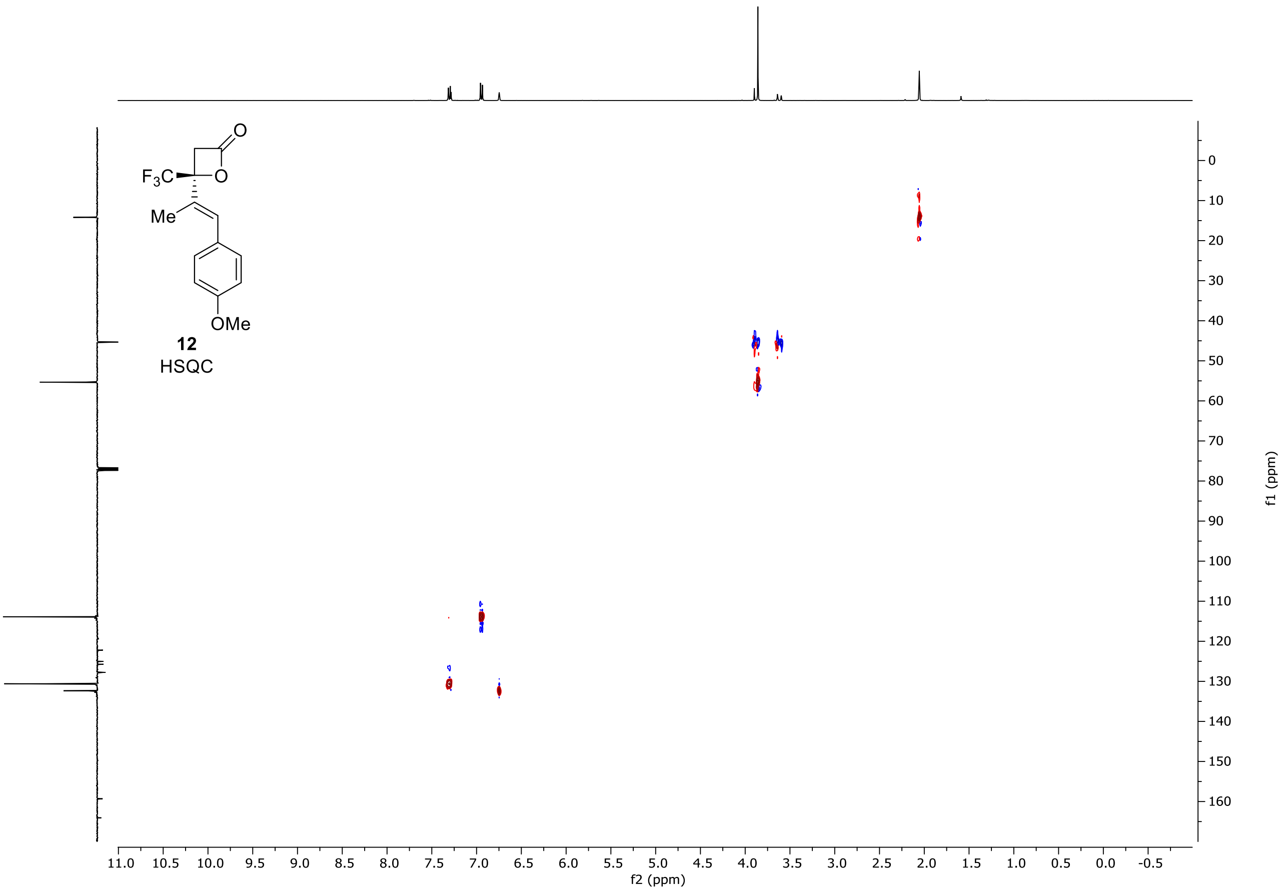
F3C
O
O
Me
OMe
12
HSQC
f2 (ppm)
f1 (ppm)

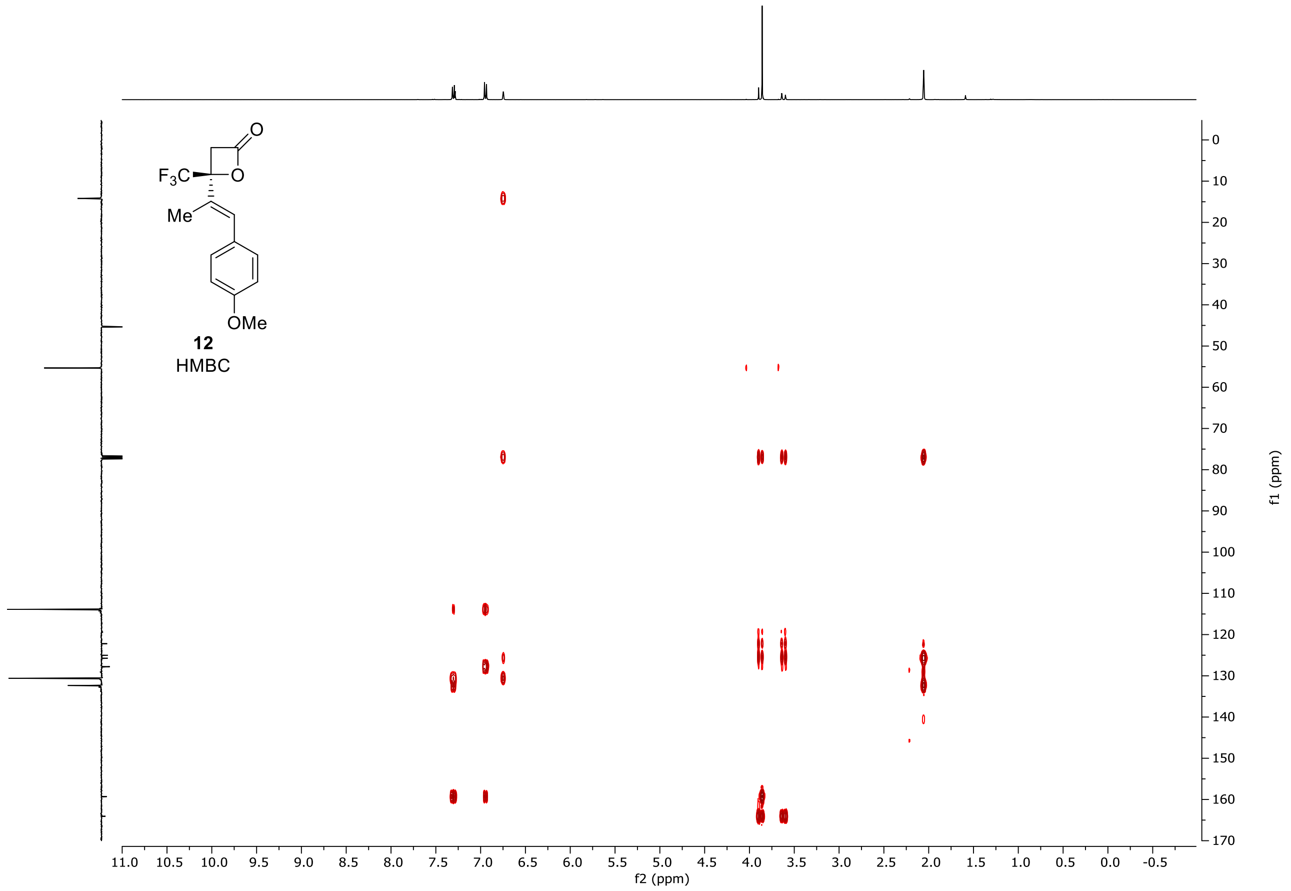
12
HMBC
f2 (ppm)
f1 (ppm)

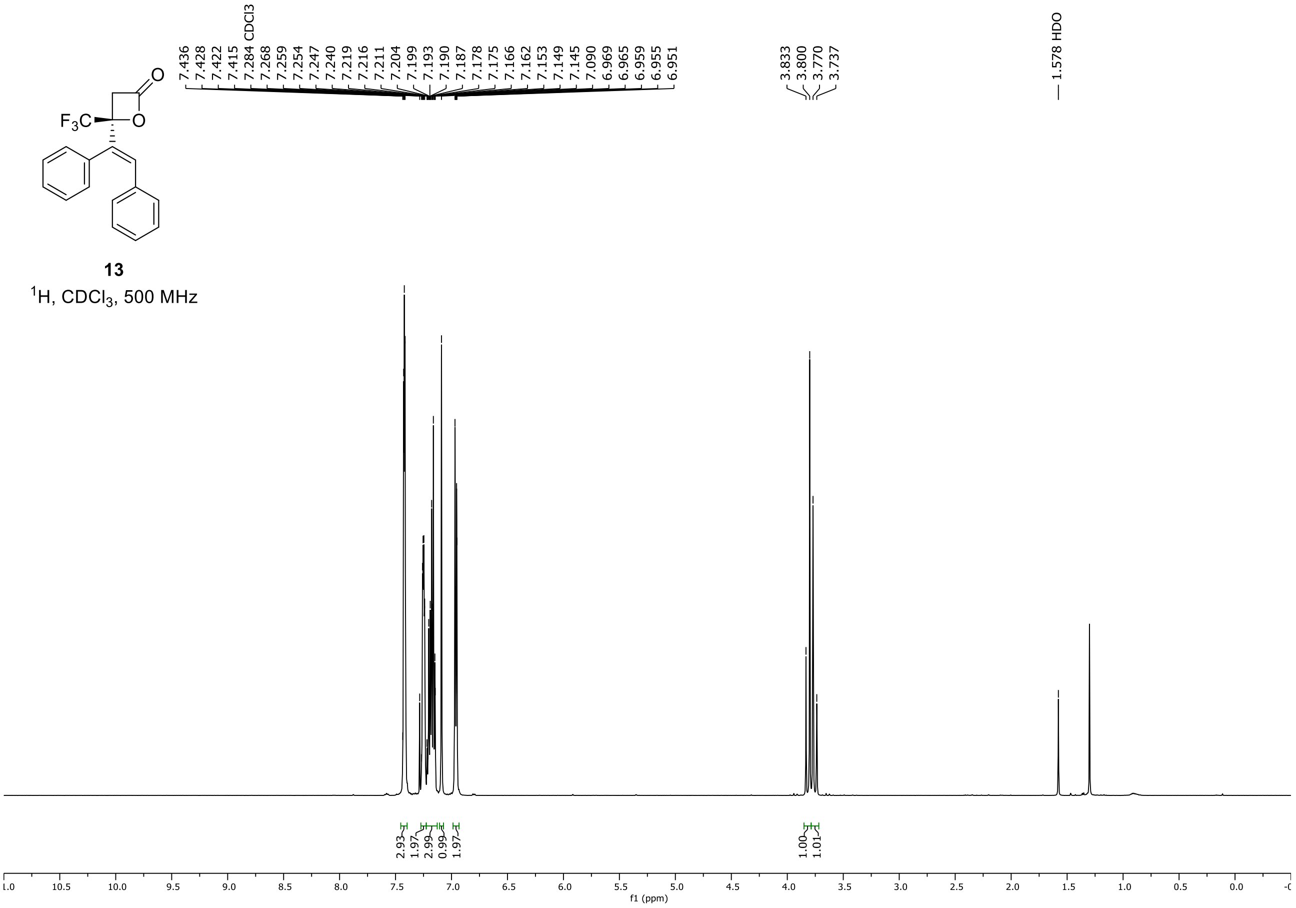
13
^{1}H, CDCl$_3$, 500 MHz
7.436 7.428 7.422 7.415 7.284 CDCl3 7.268 7.259 7.254 7.247 7.240 7.219 7.216 7.211 7.204 7.199 7.193 7.190 7.187 7.178 7.175 7.166 7.162 7.153 7.149 7.145 7.090 6.969 6.965 6.959 6.955 6.951
3.833 3.800 3.770 3.737
1.578 HDO
2.93 1.97 2.99 0.99 1.97
1.00 1.01
f1 (ppm)

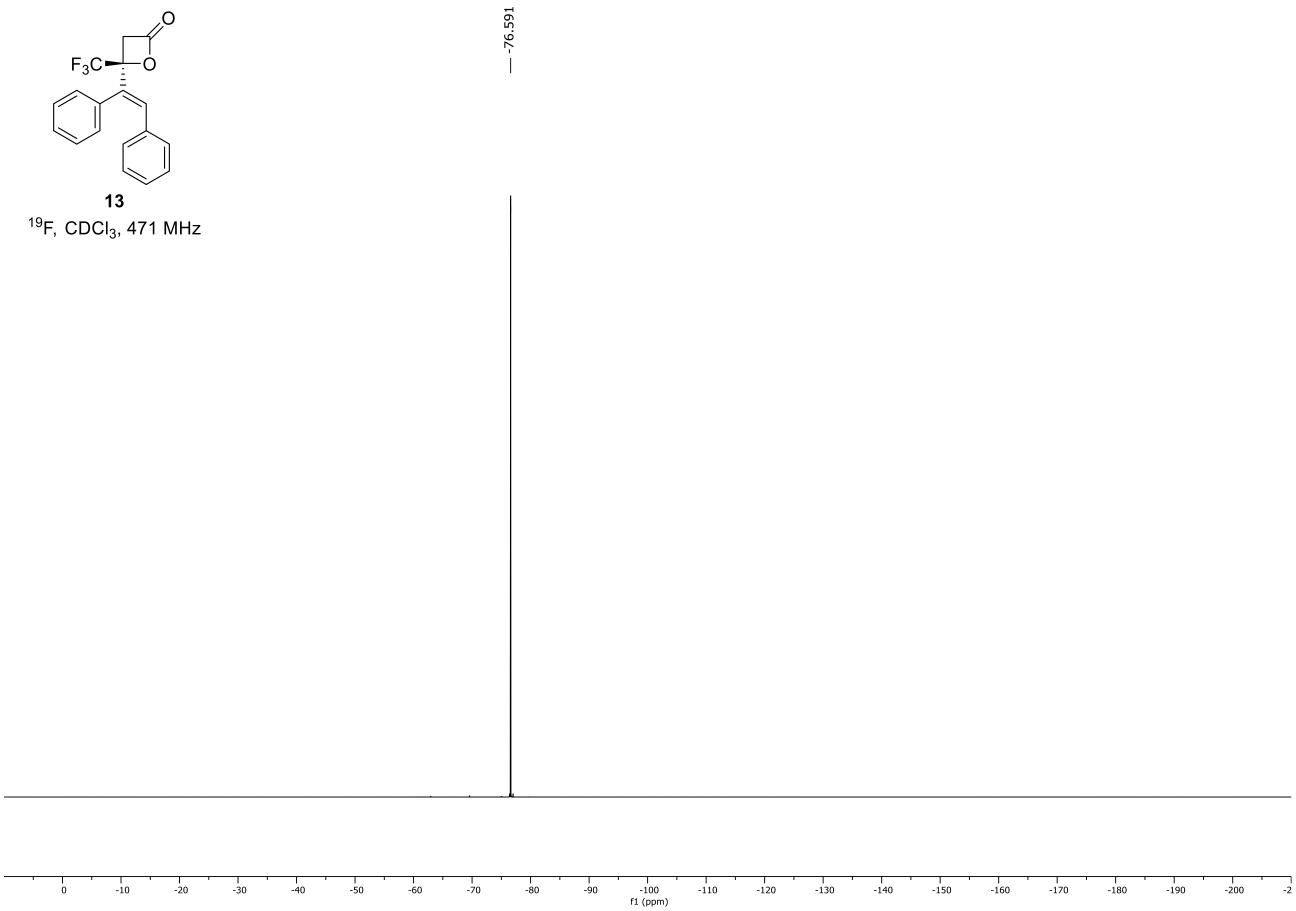
F3C
O
O
13
19F, CDCl3, 471 MHz
-76.591
0
-10
-20
-30
-40
-50
-60
-70
-80
-90
-100
-110
-120
-130
-140
-150
-160
-170
-180
-190
-200
f1 (ppm)

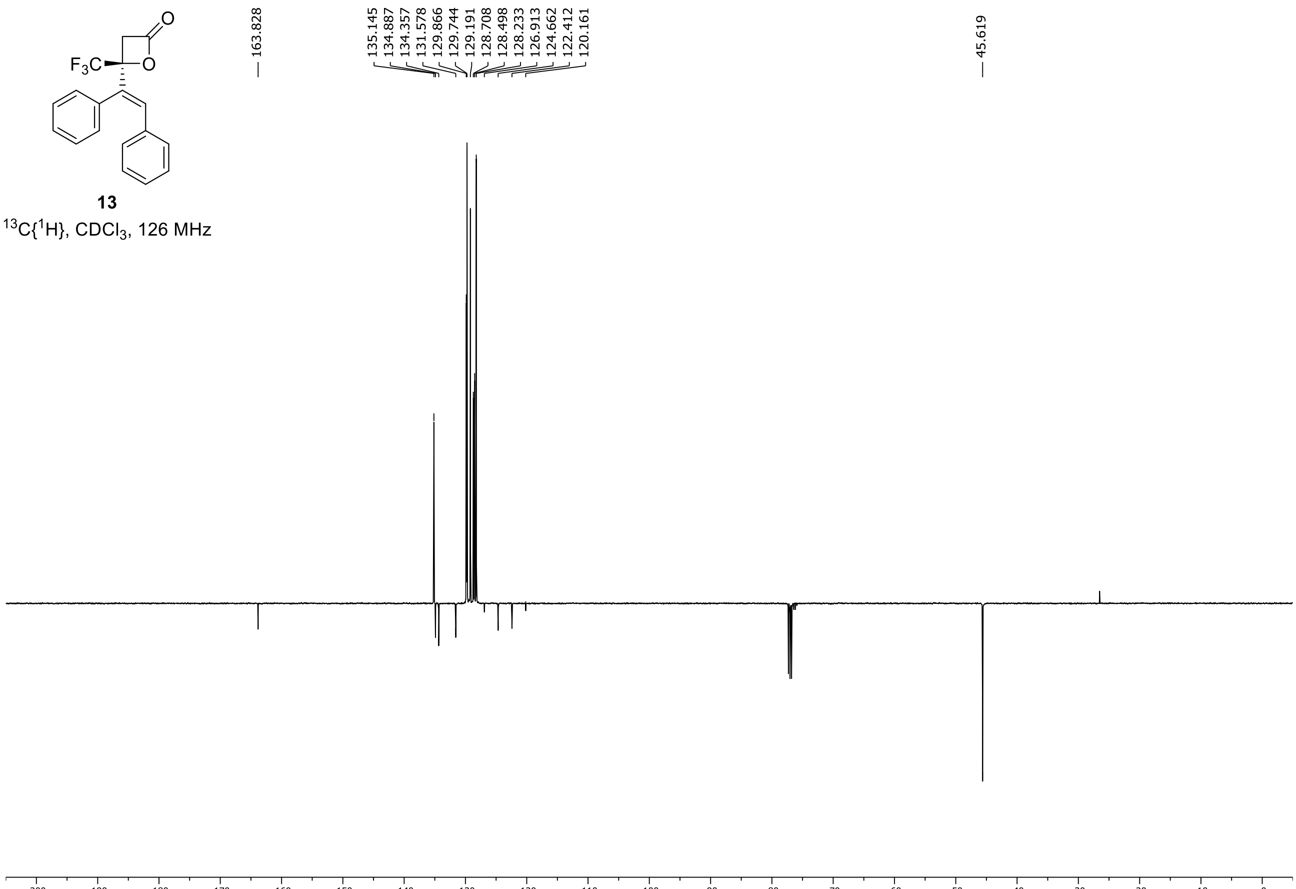
F3C
O
O
13
13C{1H}, CDCl3, 126 MHz
163.828
135.145
134.887
134.357
131.578
129.866
129.744
129.191
128.708
128.498
128.233
126.913
124.662
122.412
120.161
45.619
200
190
180
170
160
150
140
130
120
110
100
90
80
70
60
50
40
30
20
10
0
f1 (ppm)

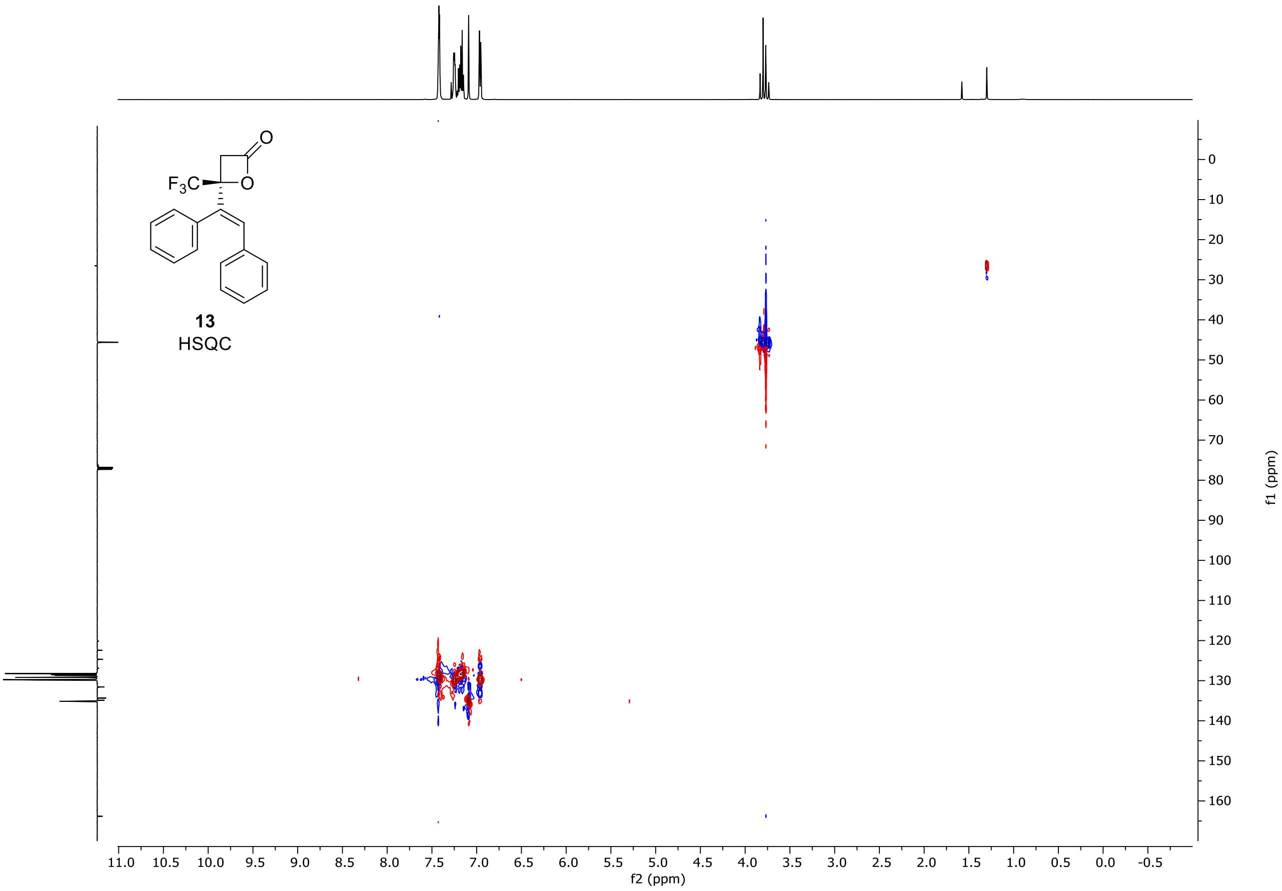
F3C
O
O
13
HSQC
f1 (ppm)
f2 (ppm)

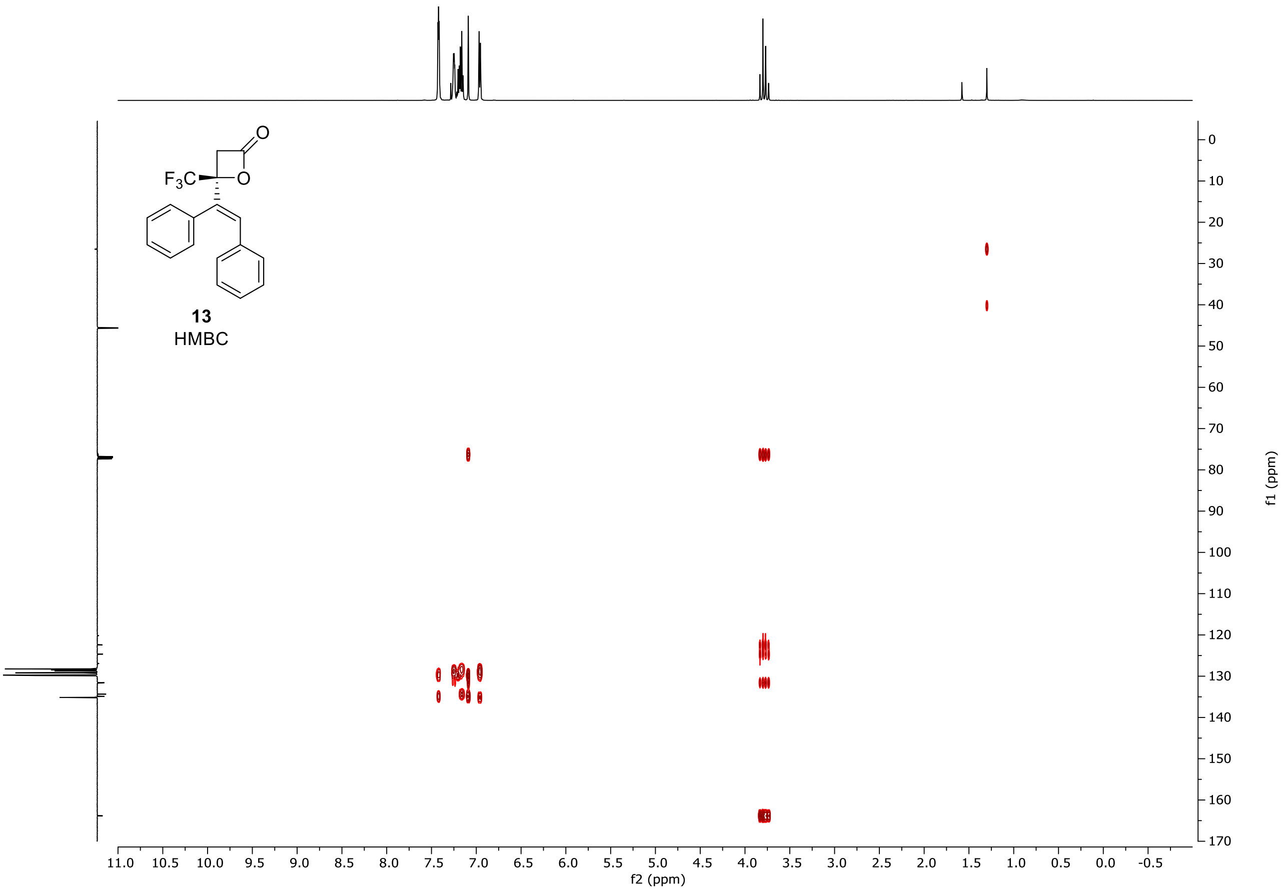
F3C
O
O
13
HMBC
f1 (ppm)
f2 (ppm)

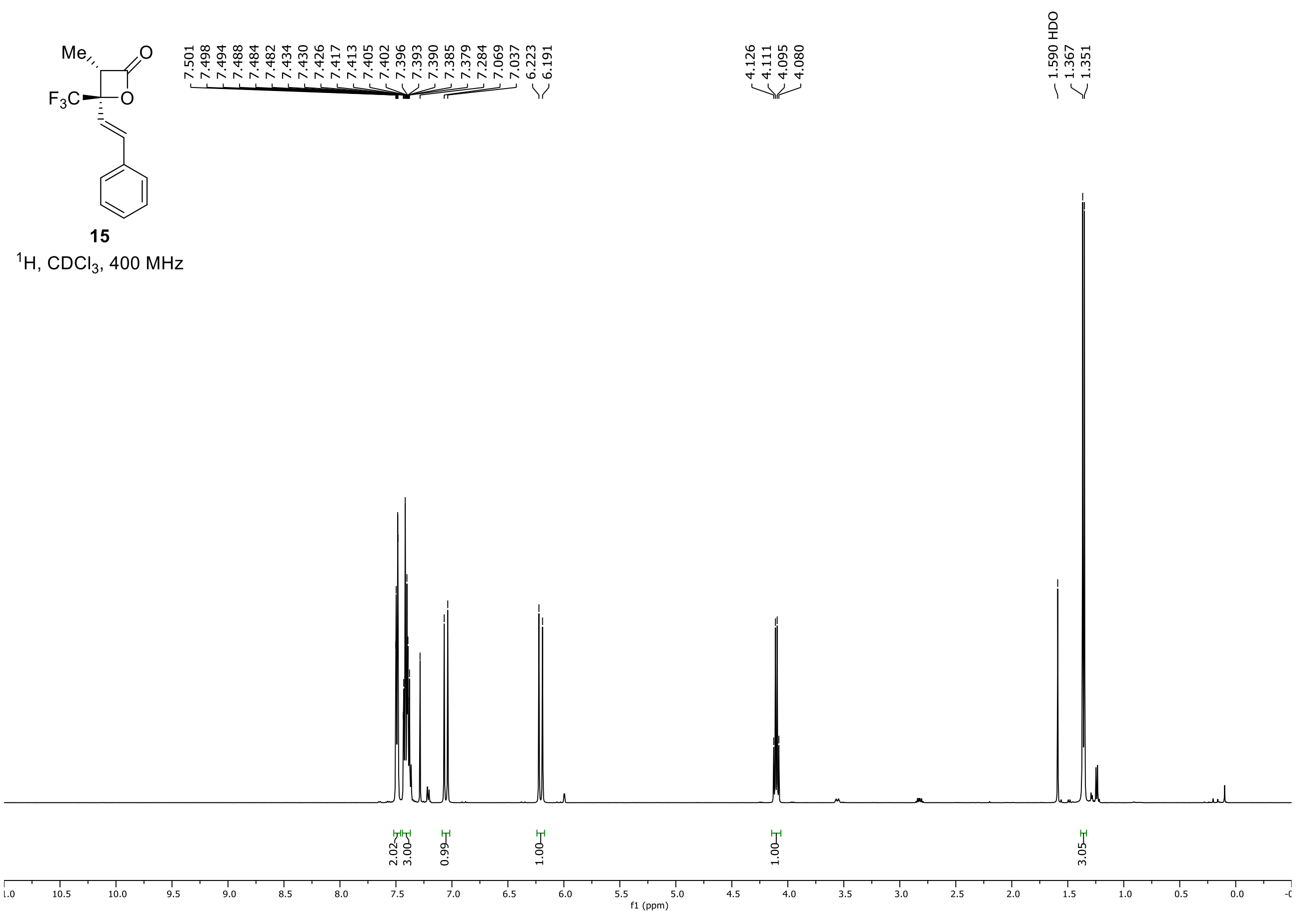

15

^{1}H, $CDCl_3$, 400 MHz

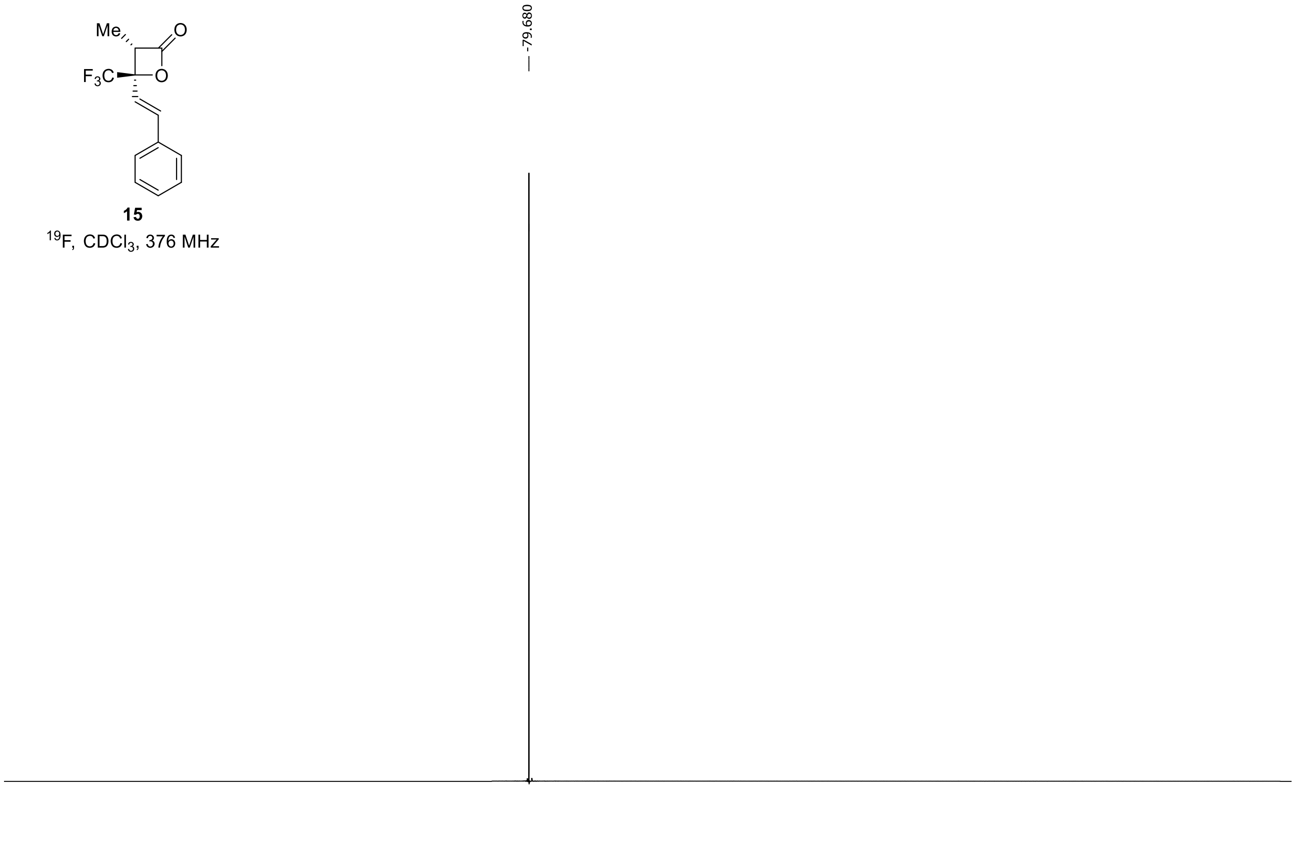

15

^{19}F, $CDCl_3$, 376 MHz

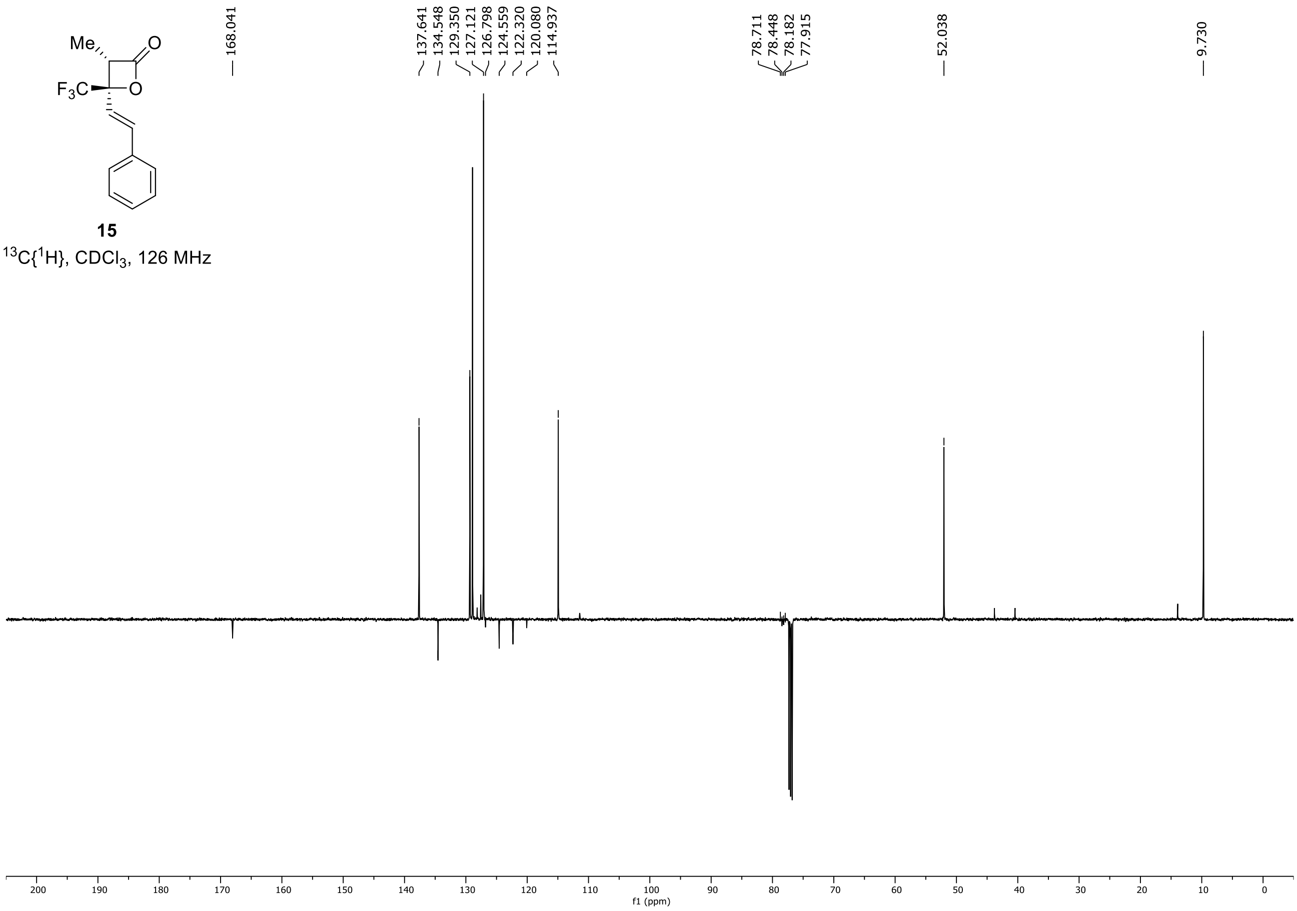

15

$^{13}C\{^{1}H\}$, $CDCl_3$, 126 MHz

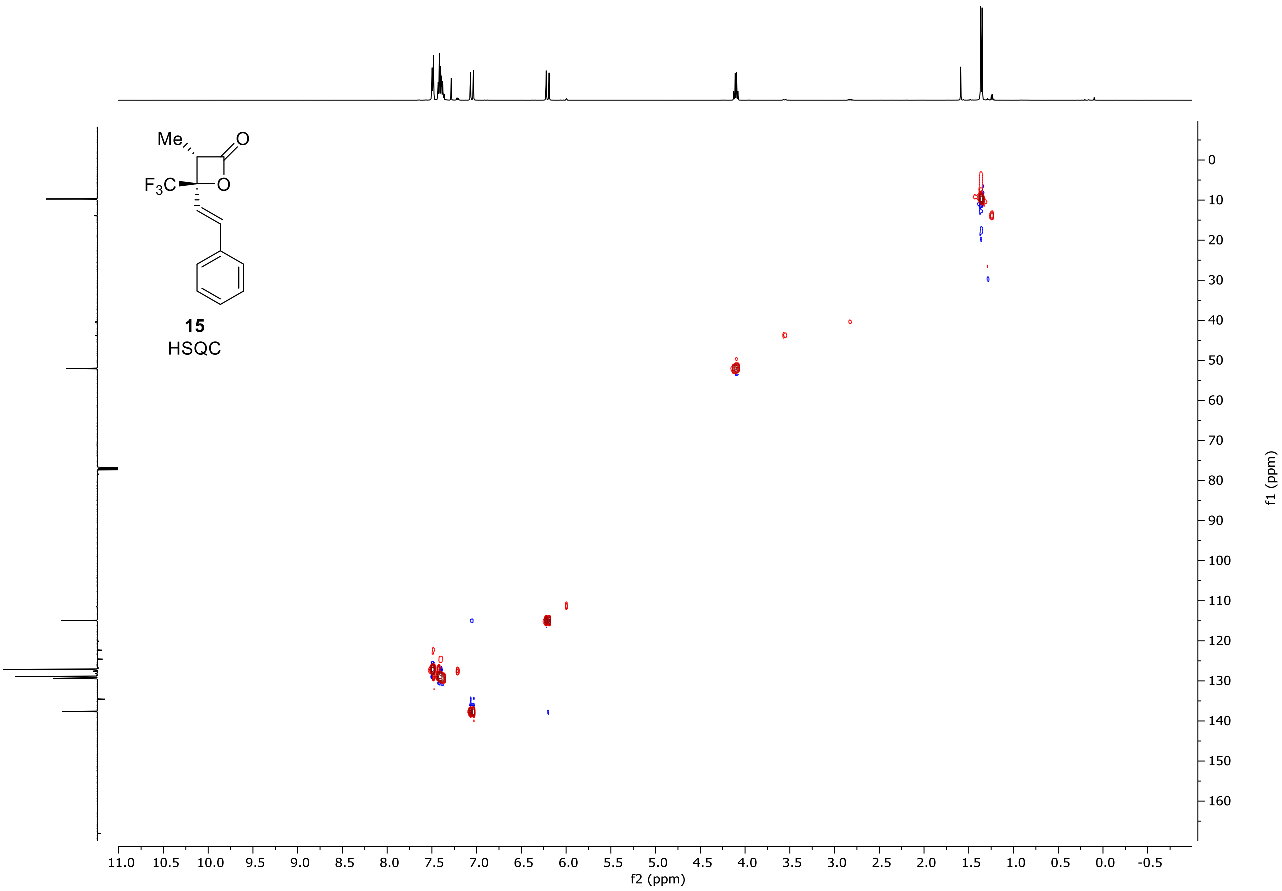

15

HSQC

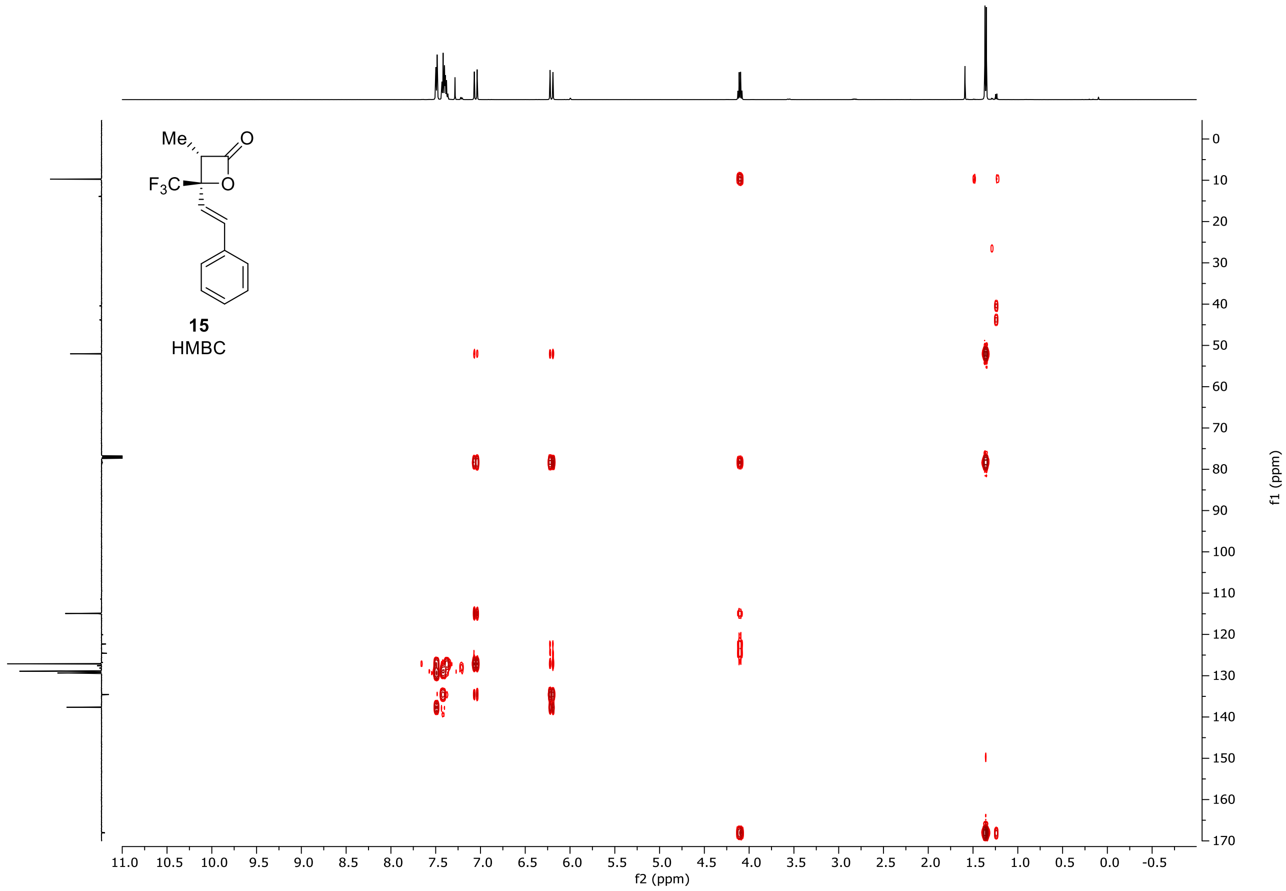
Me
O
F3C
O
15
HMBC
f2 (ppm)
f1 (ppm)

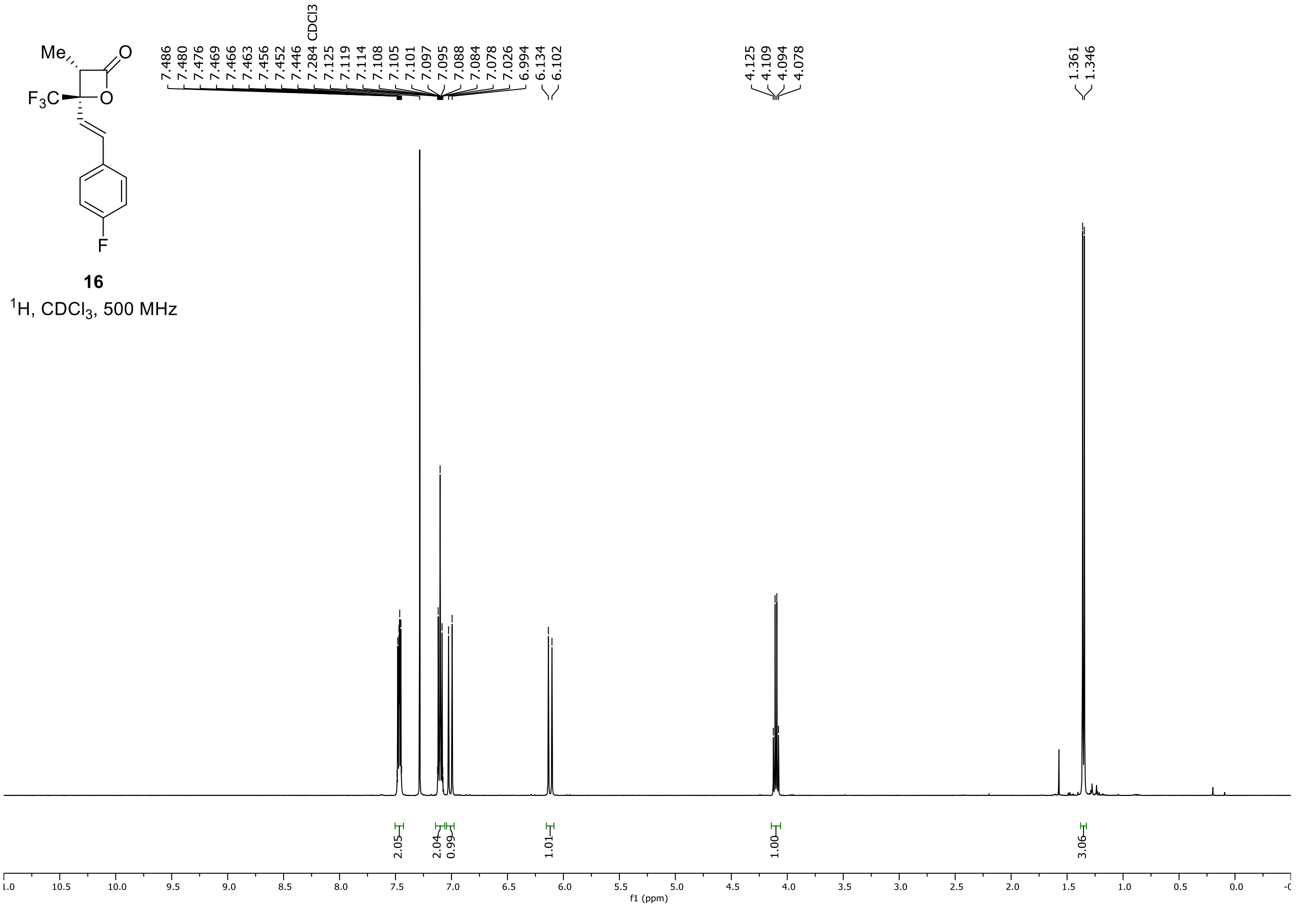
Me
O
F3C
O
F
16
1H, CDCl3, 500 MHz
7.486
7.480
7.476
7.469
7.466
7.463
7.456
7.452
7.446
7.284 CDCl3
7.125
7.119
7.114
7.108
7.105
7.101
7.097
7.095
7.088
7.084
7.078
7.026
6.994
6.134
6.102
4.125
4.109
4.094
4.078
1.361
1.346
2.05
2.04
0.99
1.01
1.00
3.06
f1 (ppm)

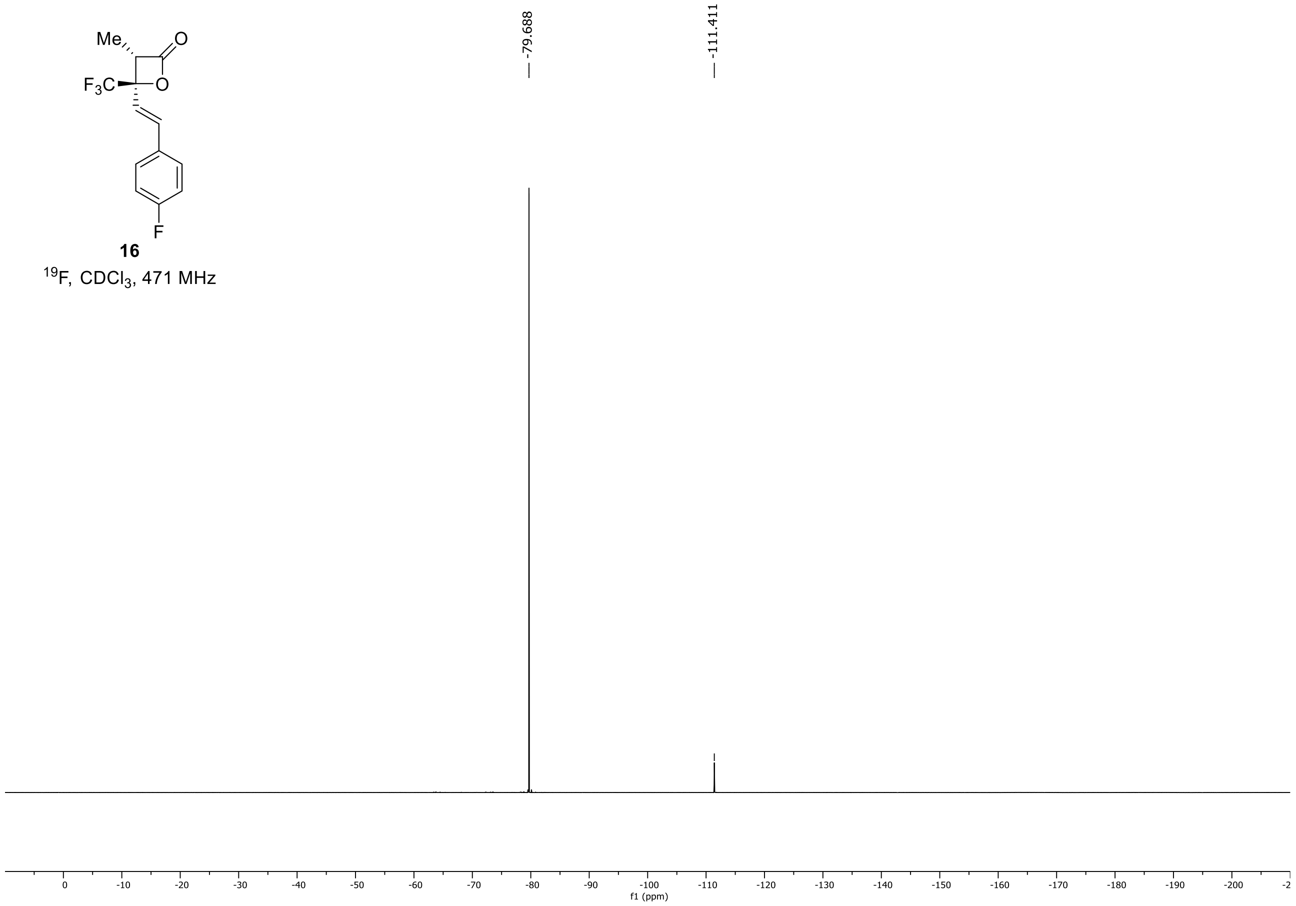
Me
O
F3C
O
F
16
19F, CDCl3, 471 MHz
-79.688
-111.411
f1 (ppm)

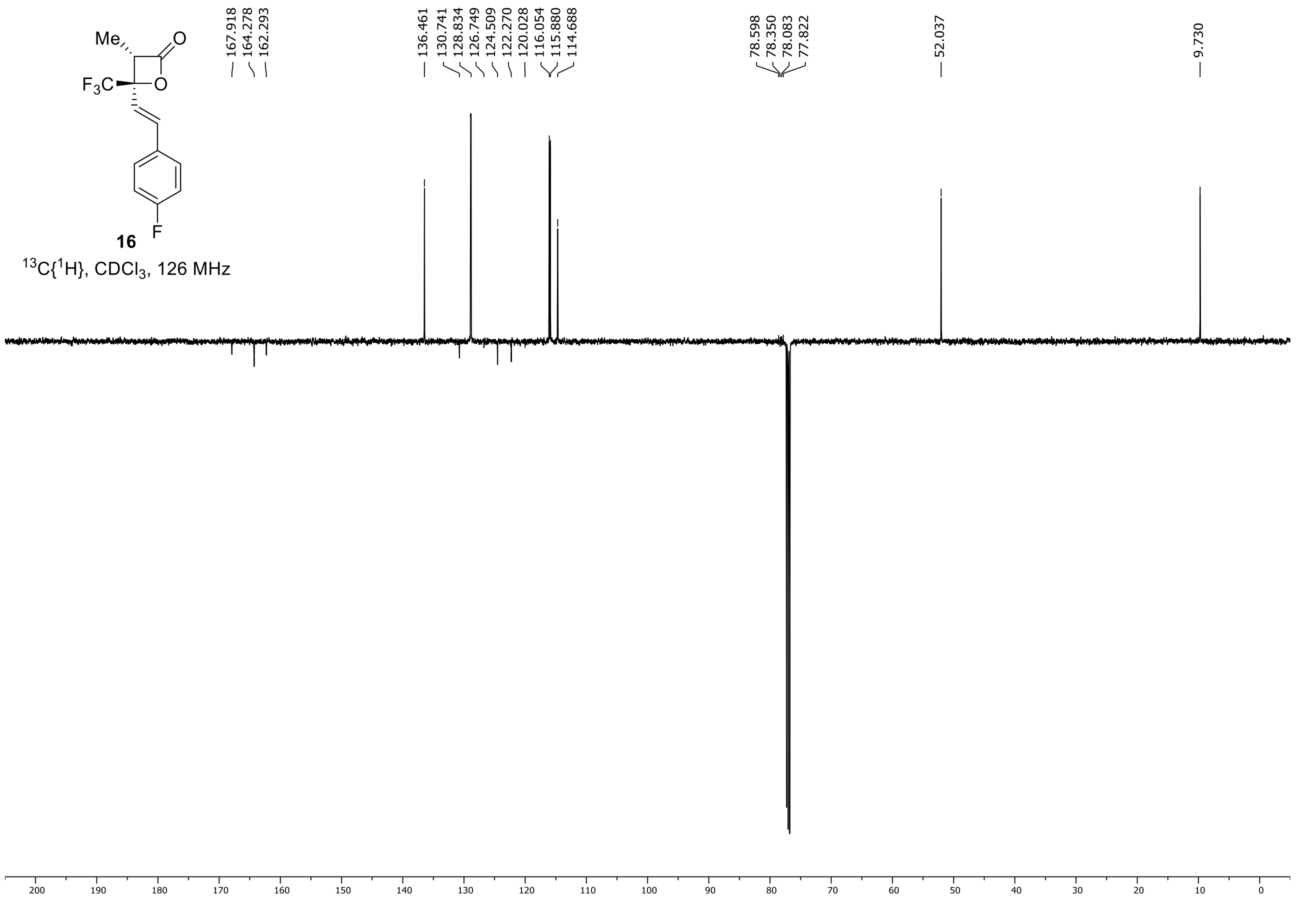
Me
O
F3C
O
F
16
13C{1H}, CDCl3, 126 MHz
167.918
164.278
162.293
136.461
130.741
128.834
126.749
124.509
122.270
120.028
116.054
115.880
114.688
78.598
78.350
78.083
77.822
52.037
9.730
f1 (ppm)

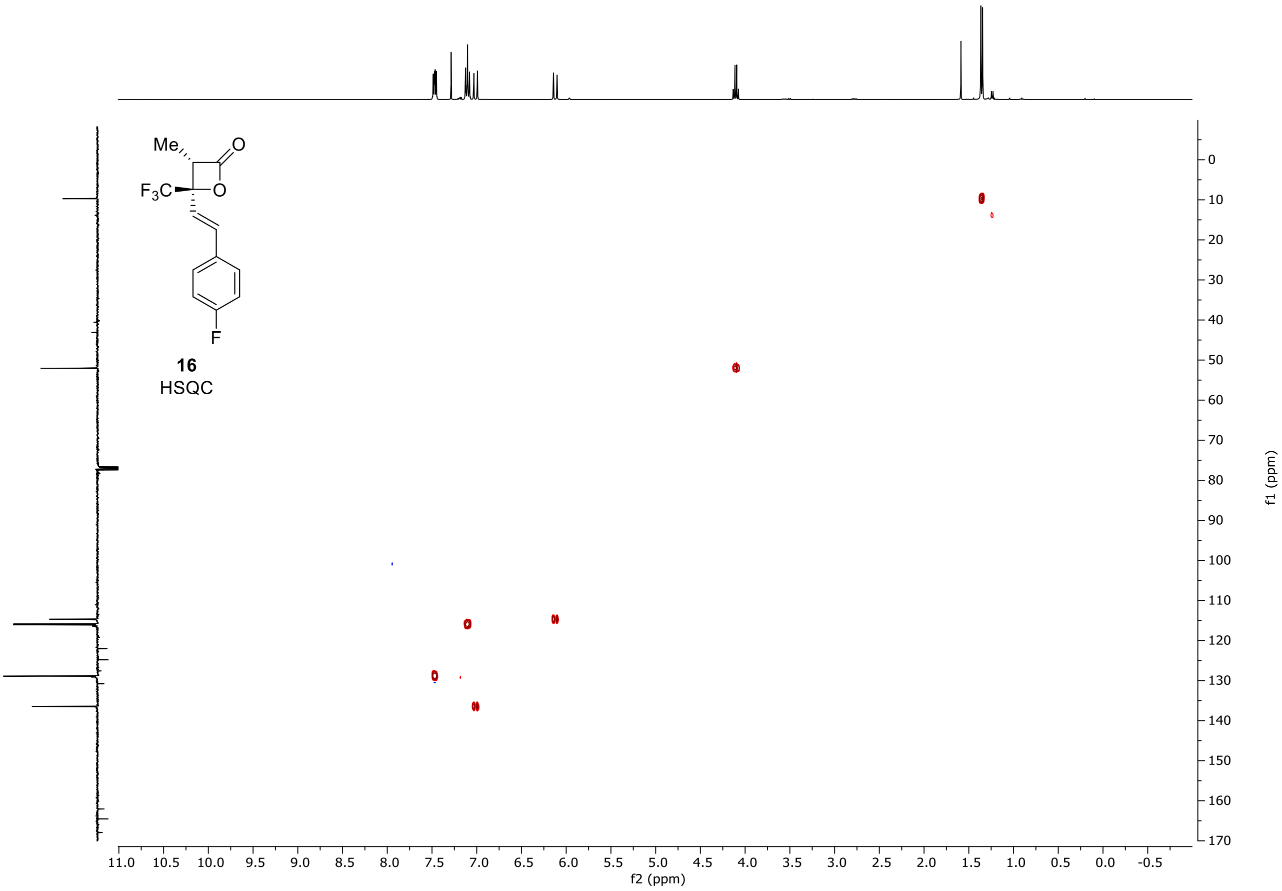
Me
O
F3C
O
F
16
HSQC
f1 (ppm)
f2 (ppm)

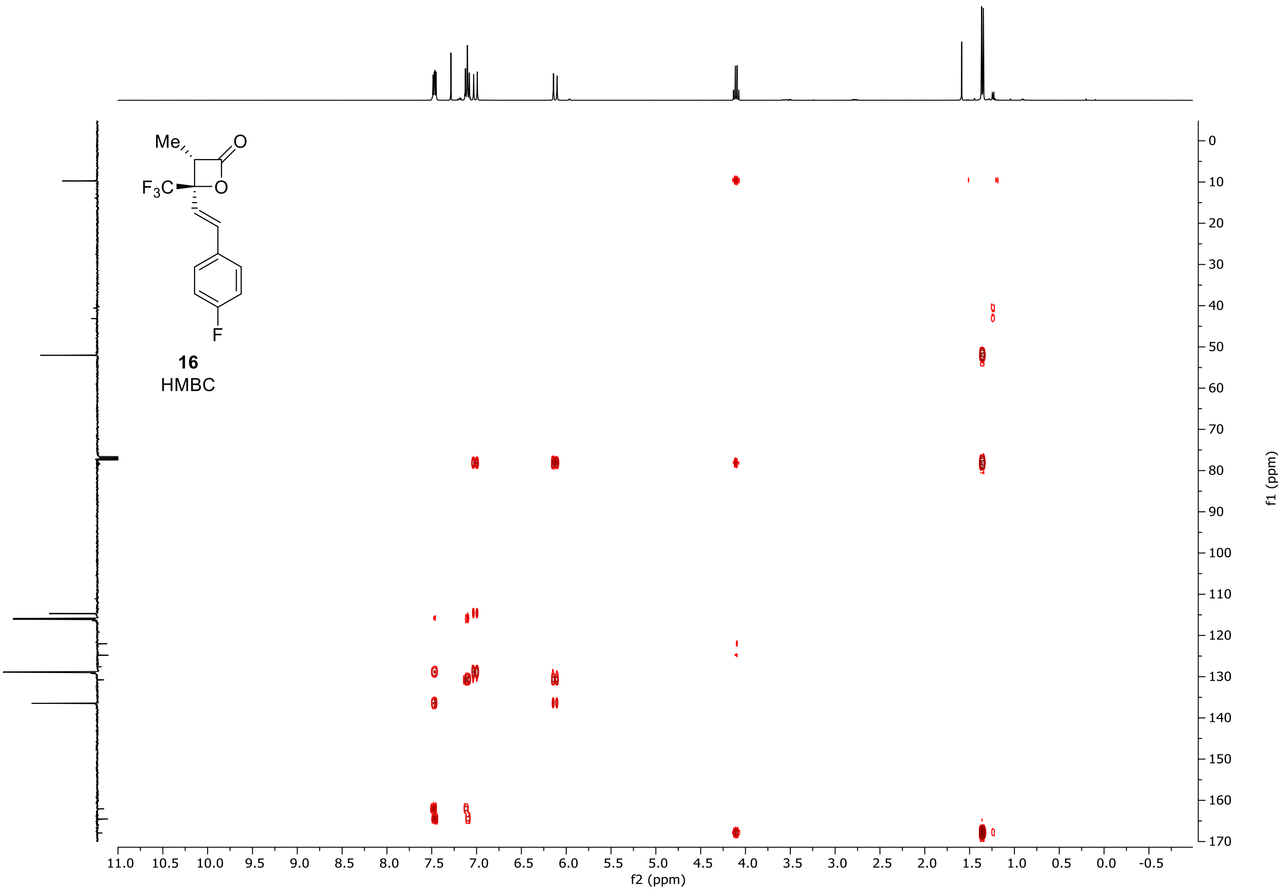
Me
O
F3C
O
F
16
HMBC
f1 (ppm)
f2 (ppm)

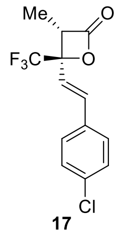

17

^{1}H, $CDCl_3$, 500 MHz

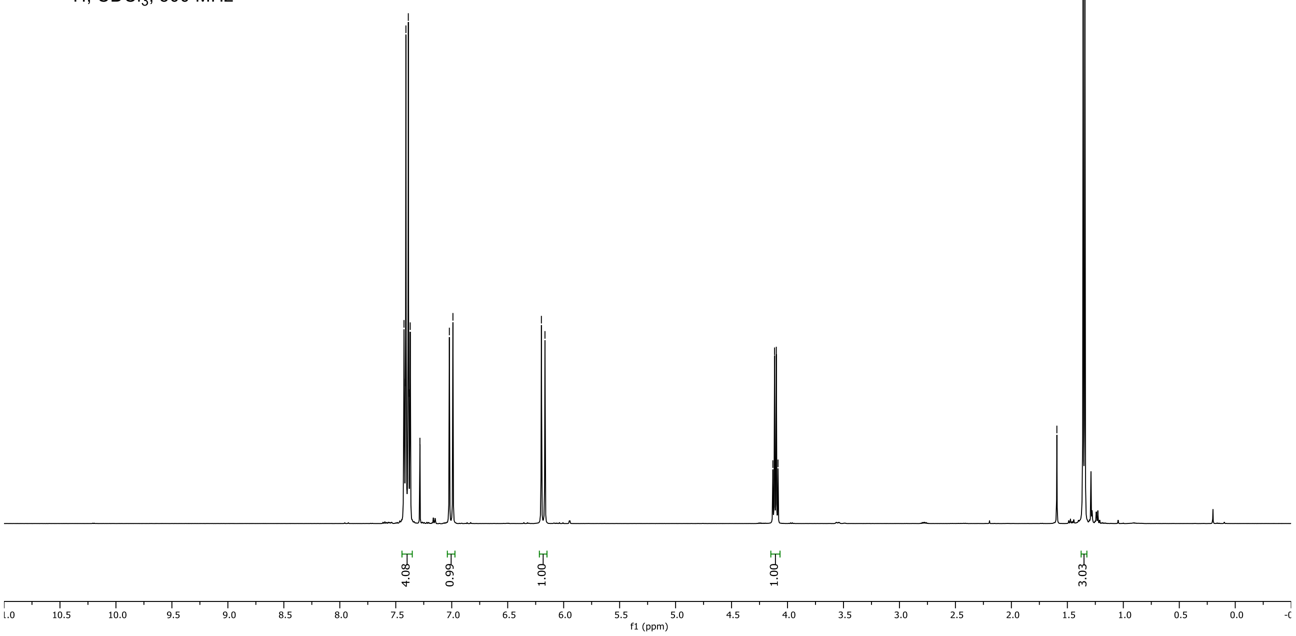

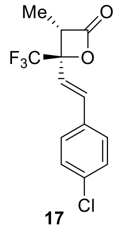

17

^{19}F, $CDCl_3$, 376 MHz

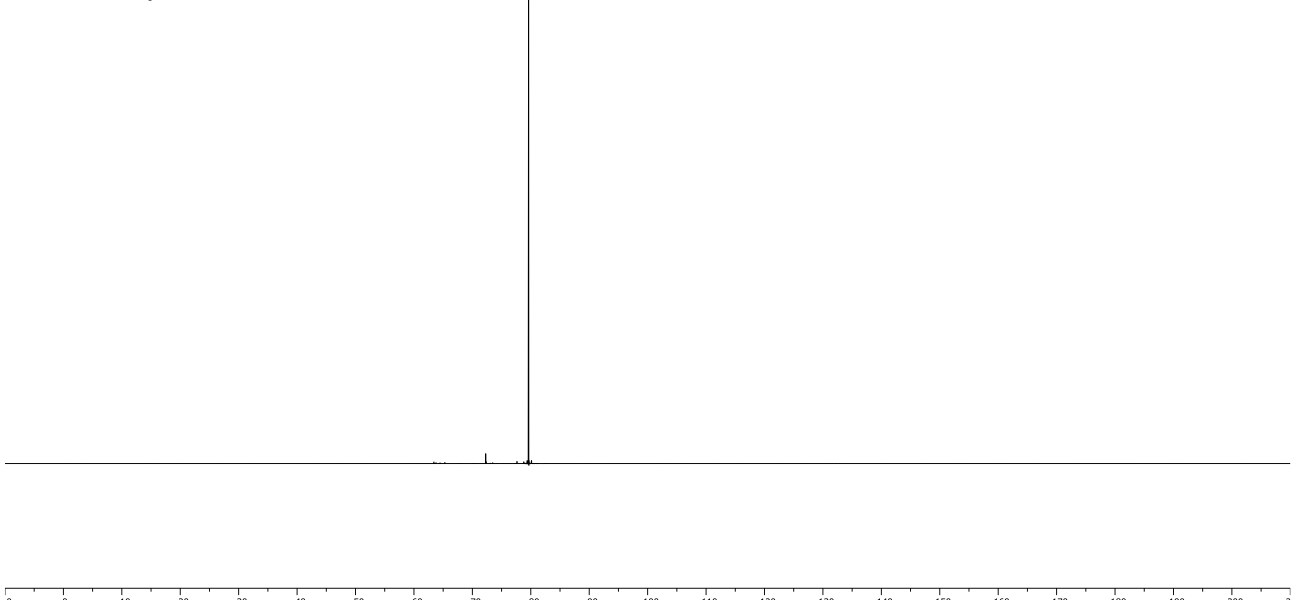

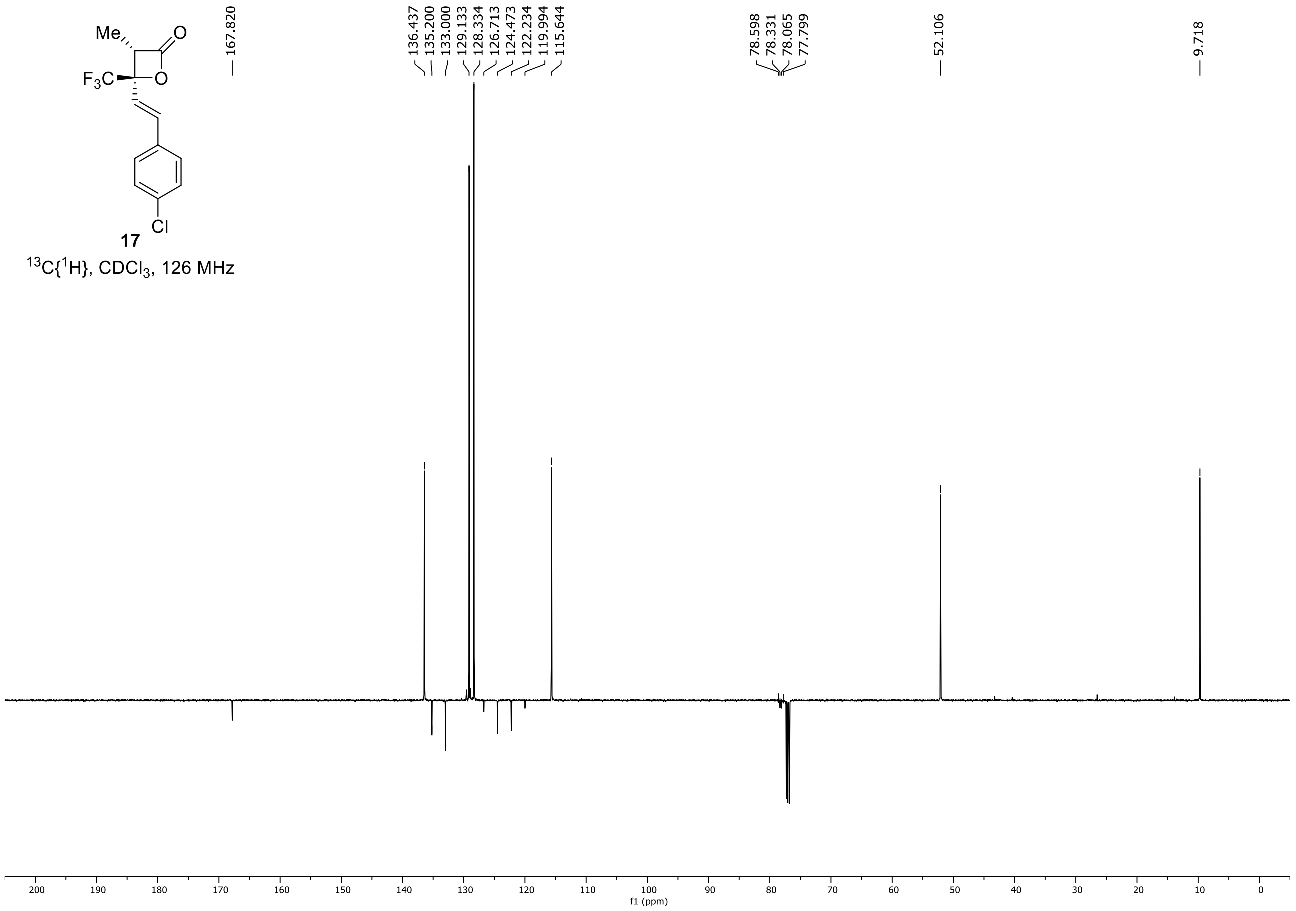
Me
O
F3C
O
Cl
17
13C{1H}, CDCl3, 126 MHz
167.820
136.437
135.200
133.000
129.133
128.334
126.713
124.473
122.234
119.994
115.644
78.598
78.331
78.065
77.799
52.106
9.718
f1 (ppm)

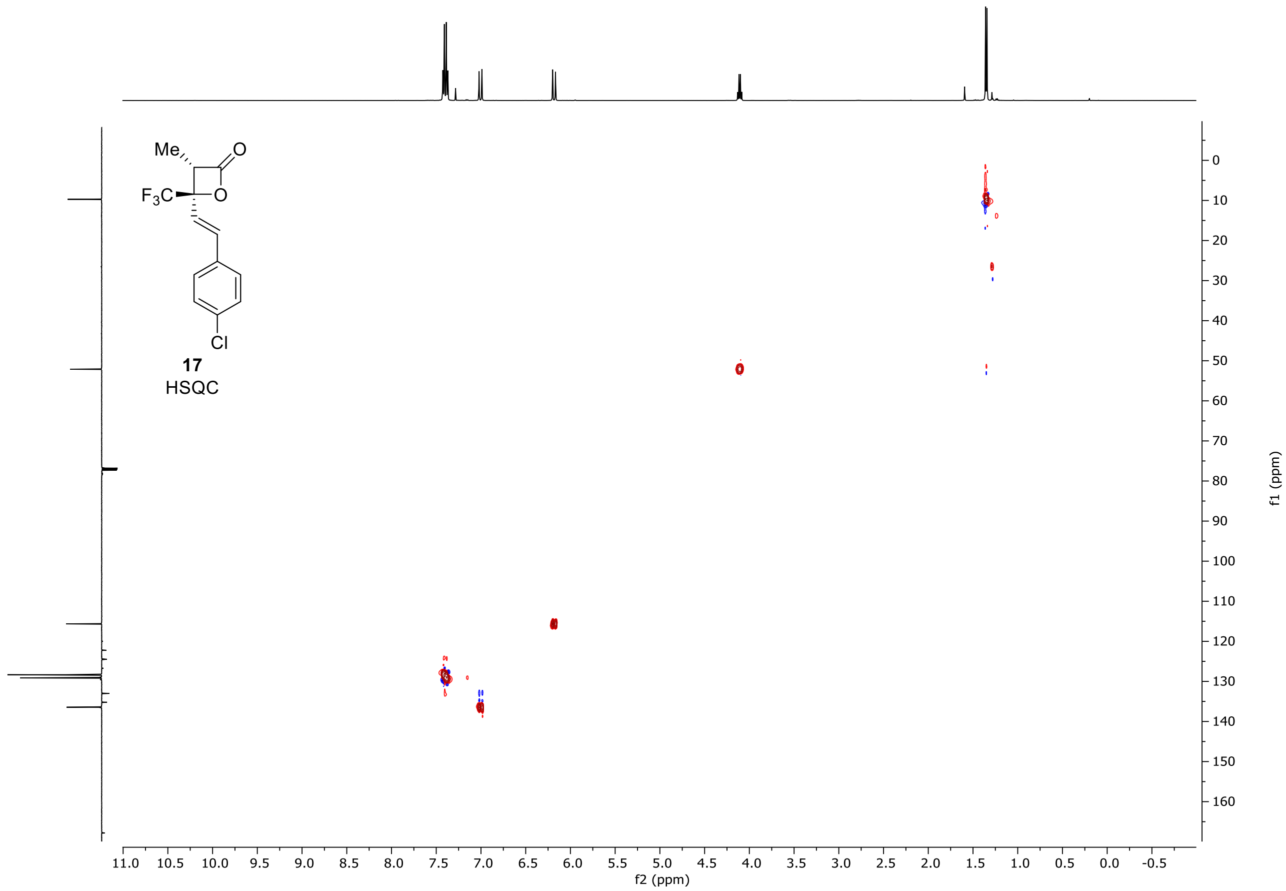
Me
O
F3C
O
Cl
17
HSQC
f2 (ppm)
f1 (ppm)

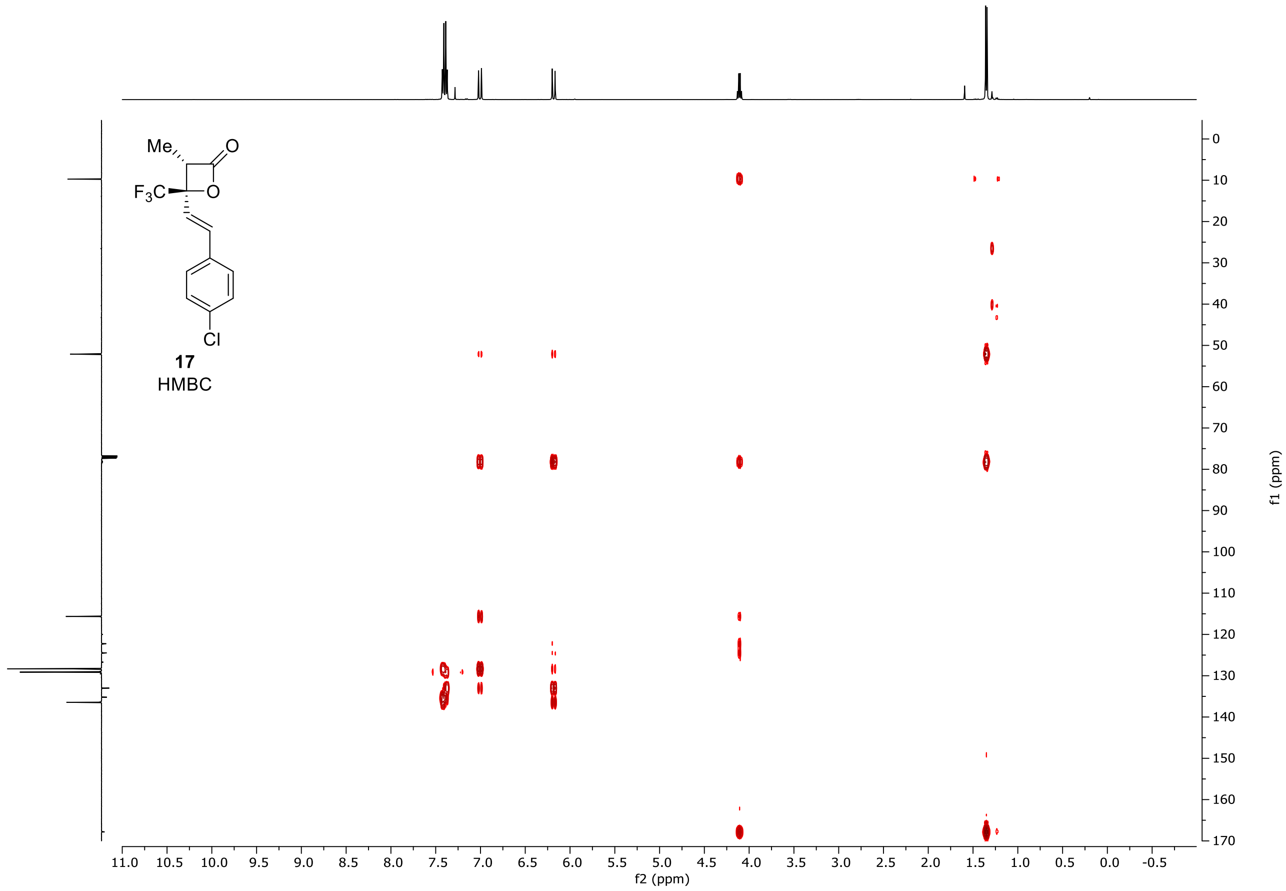
Me
O
F3C
O
Cl
17
HMBC
f2 (ppm)
f1 (ppm)

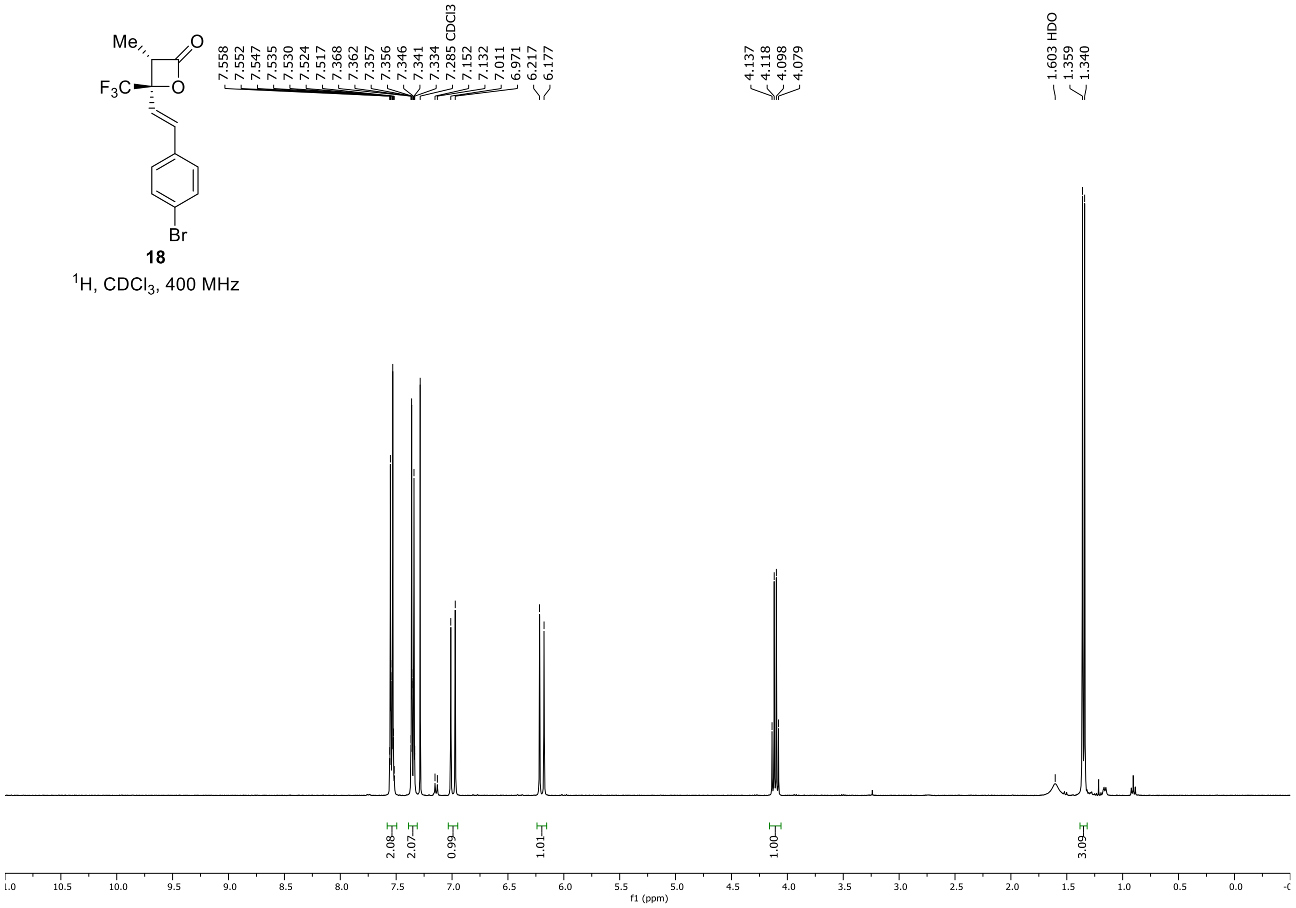
Me
O
F3C
O
Br
18
1H, CDCl3, 400 MHz
7.558
7.552
7.547
7.535
7.530
7.524
7.517
7.368
7.362
7.357
7.356
7.346
7.341
7.334
7.285 CDCl3
7.152
7.132
7.011
6.971
6.217
6.177
4.137
4.118
4.098
4.079
1.603 HDO
1.359
1.340
2.08
2.07
0.99
1.01
1.00
3.09
f1 (ppm)

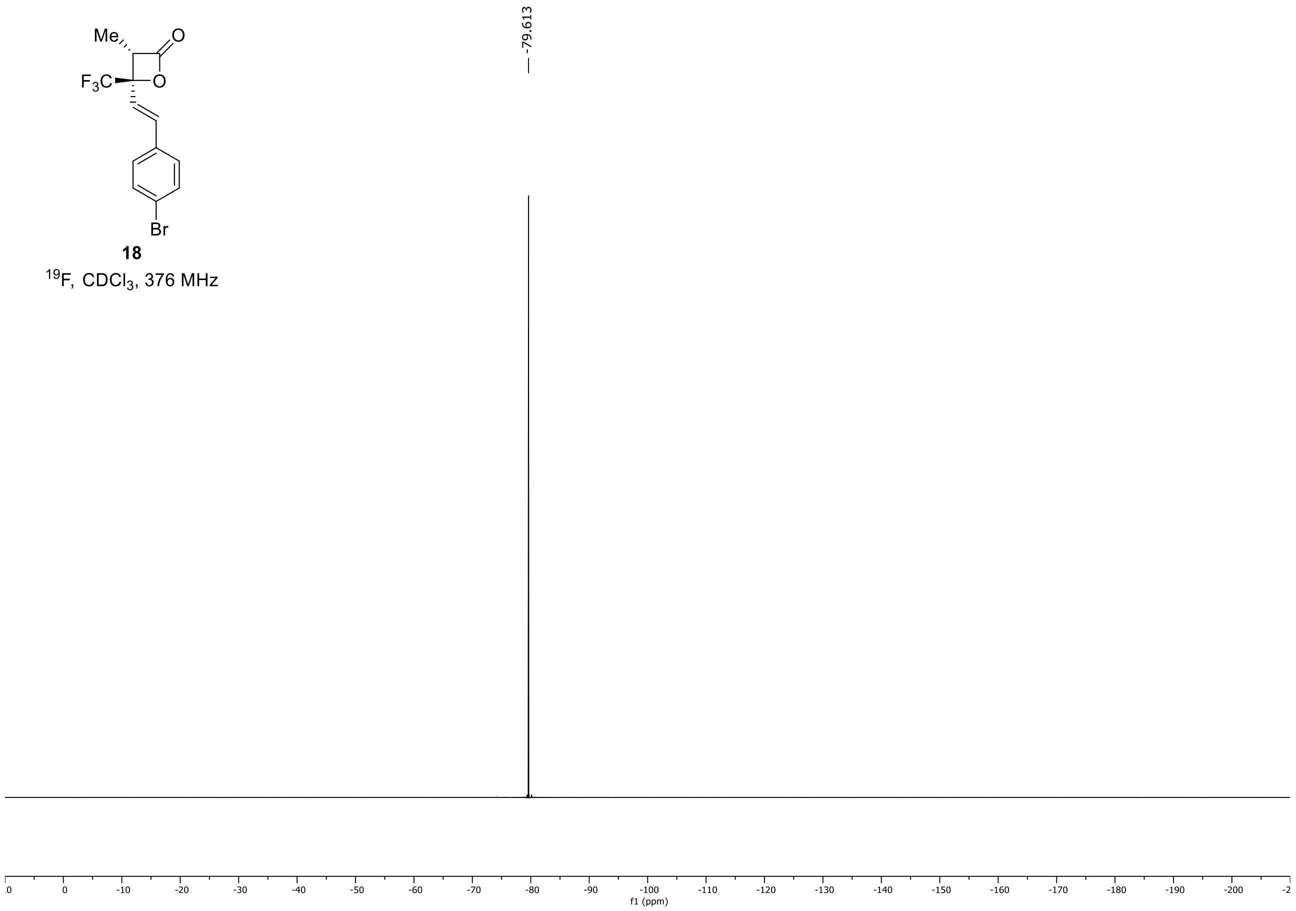
Me
O
F3C
O
Br
18
19F, CDCl3, 376 MHz
-79.613
0
0
-10
-20
-30
-40
-50
-60
-70
-80
-90
-100
-110
-120
-130
-140
-150
-160
-170
-180
-190
-200
f1 (ppm)

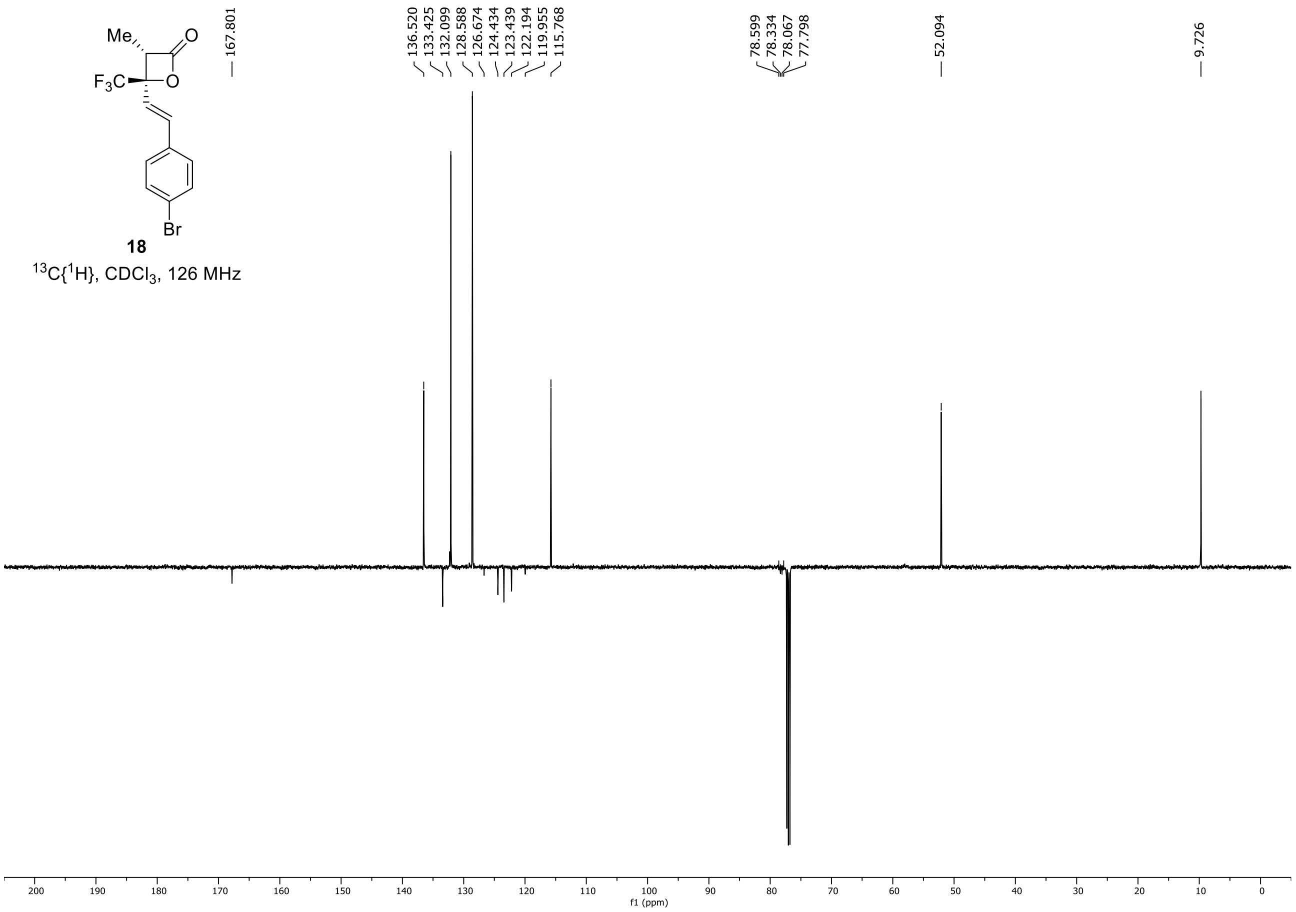
Me
O
F3C
O
Br
18
13C{1H}, CDCl3, 126 MHz
167.801
136.520
133.425
132.099
128.588
126.674
124.434
123.439
122.194
119.955
115.768
78.599
78.334
78.067
77.798
52.094
9.726
200
190
180
170
160
150
140
130
120
110
100
90
80
70
60
50
40
30
20
10
0
f1 (ppm)

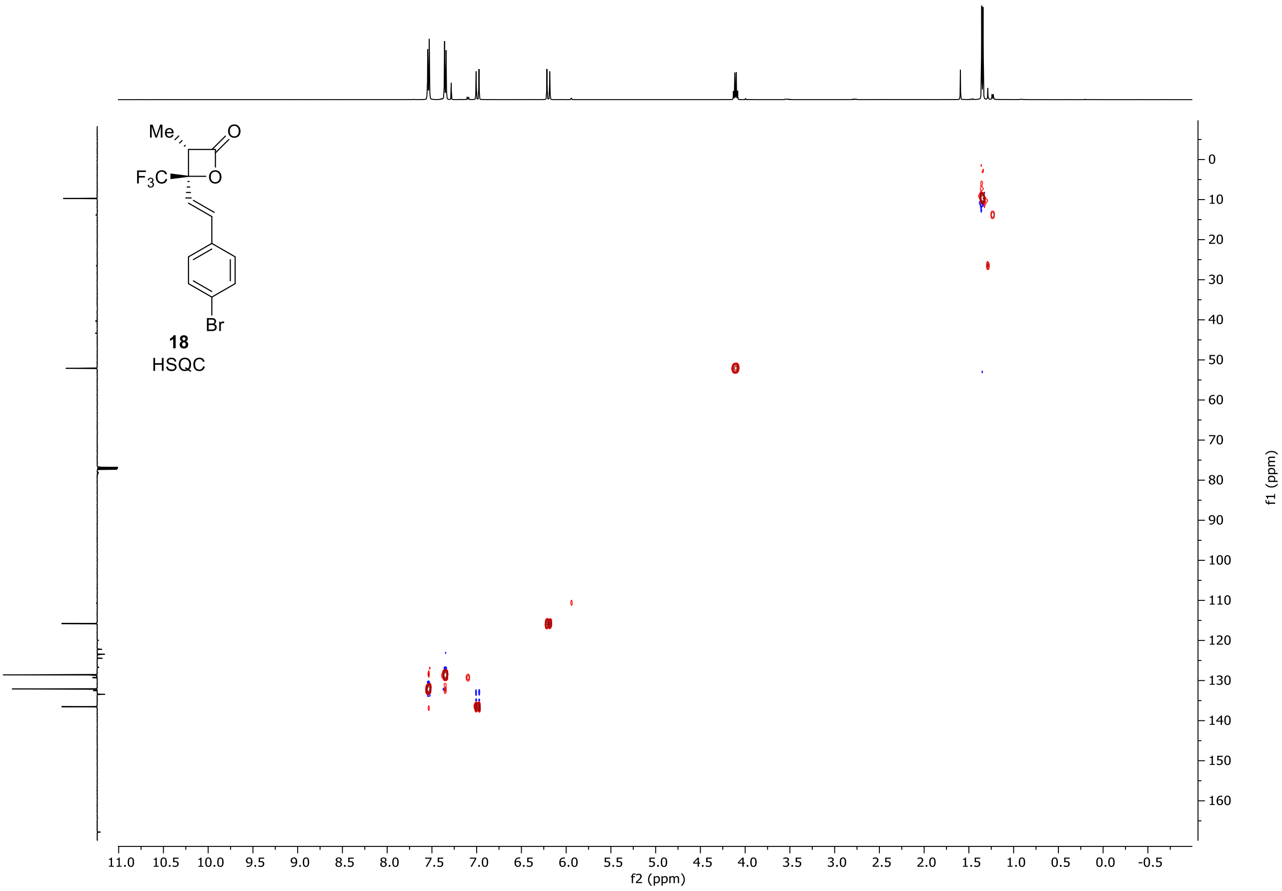

Me
O
F3C
O
Br
18
HSQC
f1 (ppm)
f2 (ppm)

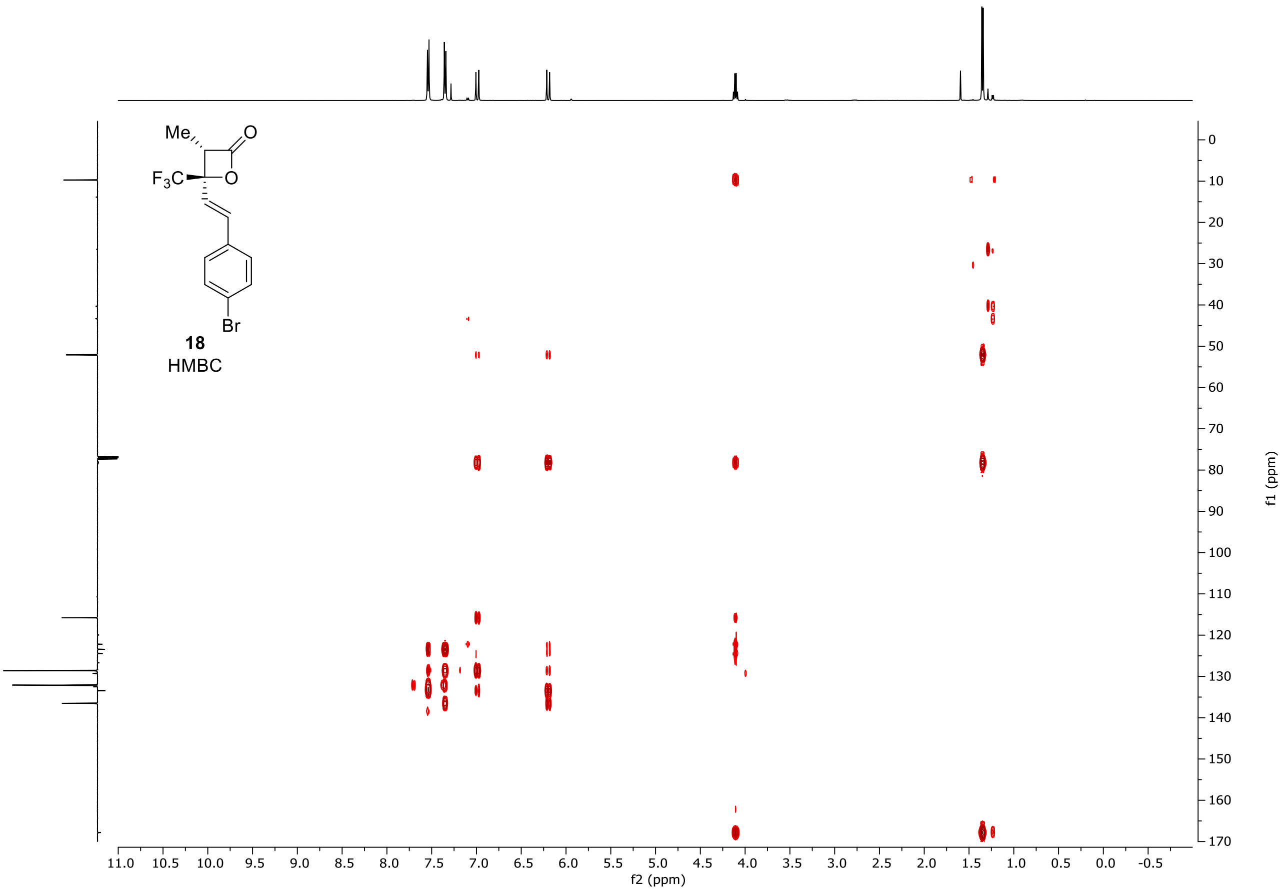

Me
O
F3C
O
Br
18
HMBC
f1 (ppm)
f2 (ppm)

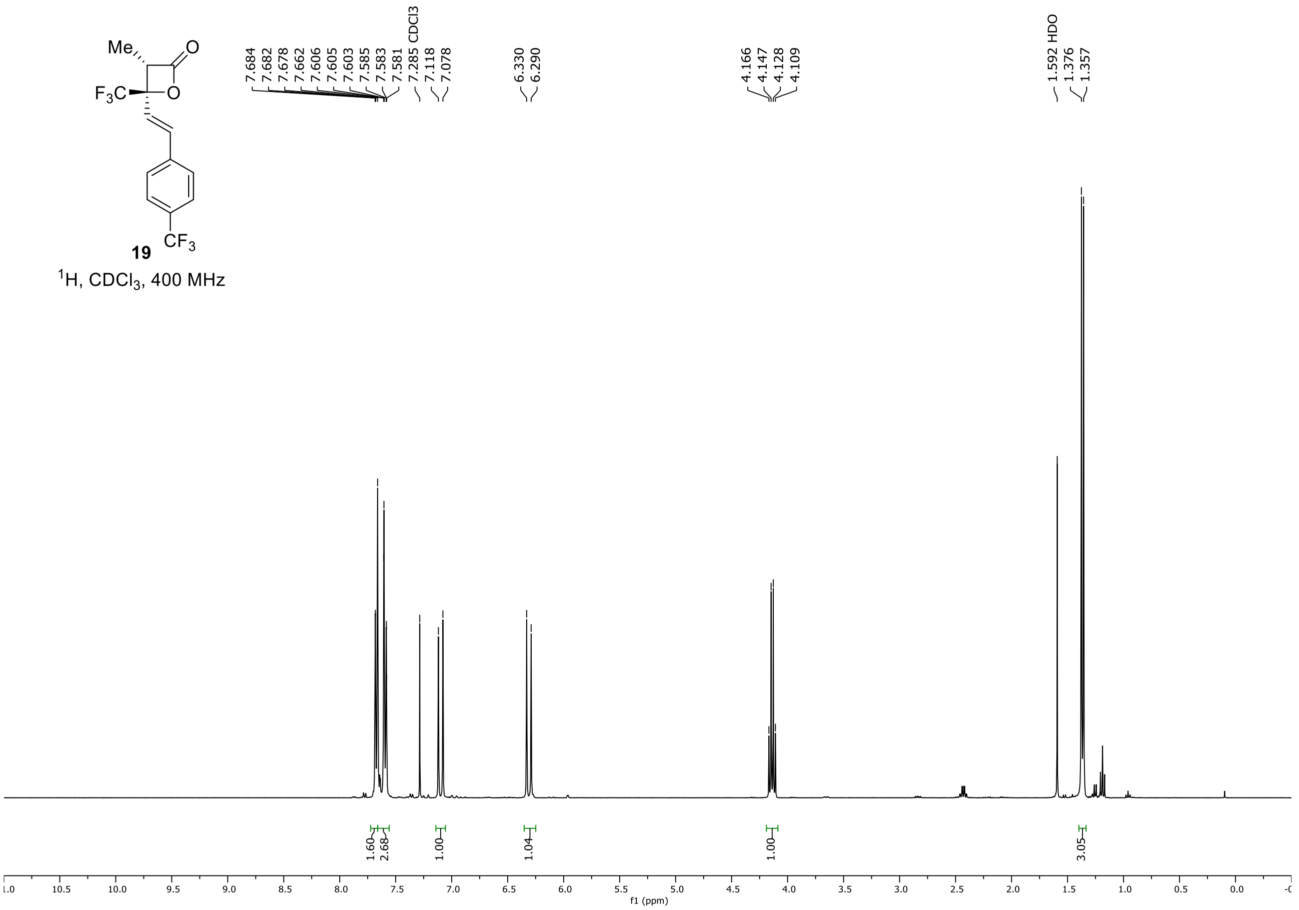
Me
O
F3C
O
CF3
19
1H, CDCl3, 400 MHz
7.684
7.682
7.678
7.662
7.606
7.605
7.603
7.585
7.583
7.581
7.285 CDCl3
7.118
7.078
6.330
6.290
4.166
4.147
4.128
4.109
1.592 HDO
1.376
1.357
1.60
2.68
1.00
1.04
1.00
3.05
f1 (ppm)

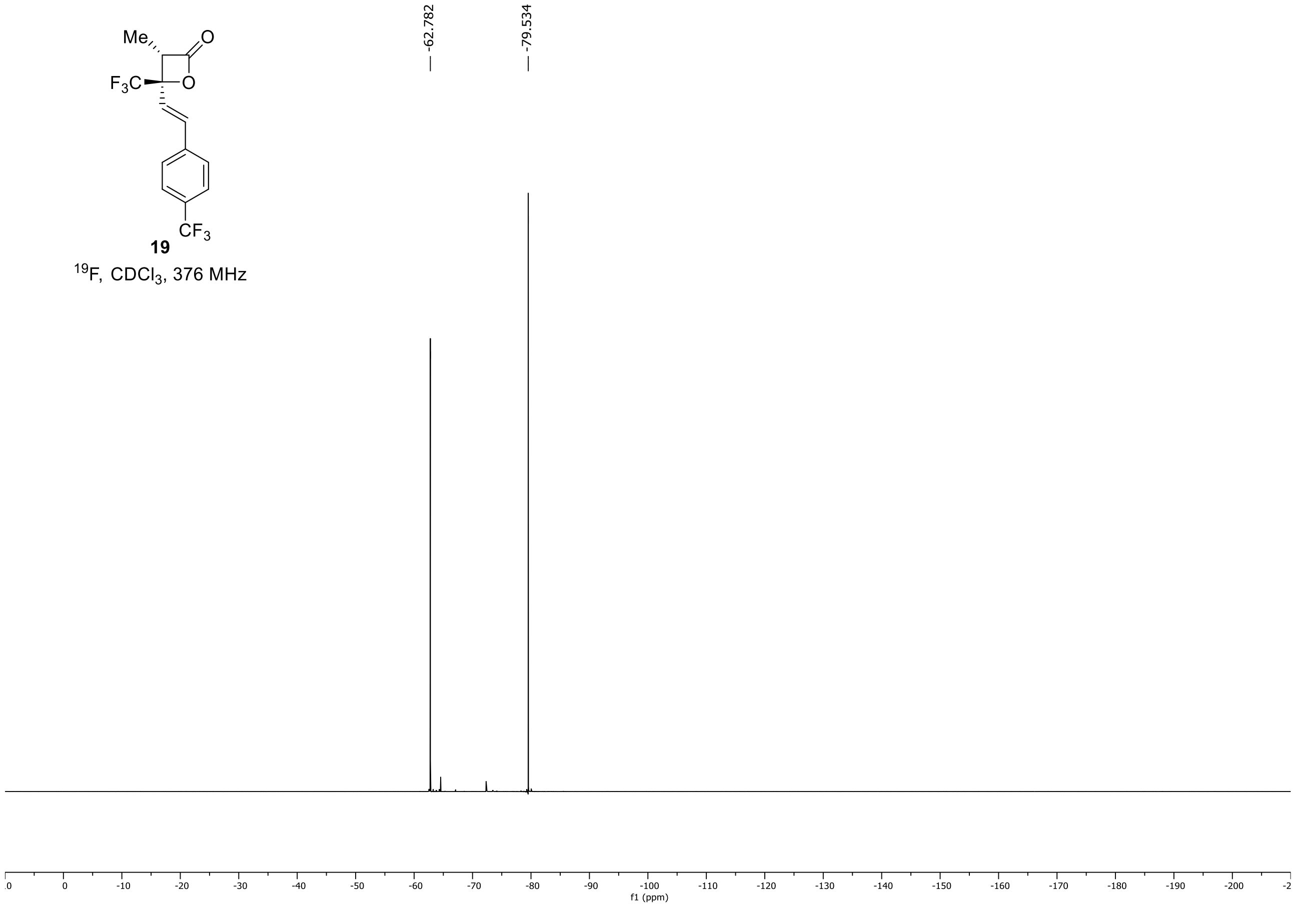
Me
O
F3C
O
CF3
19
19F, CDCl3, 376 MHz
-62.782
-79.534
f1 (ppm)

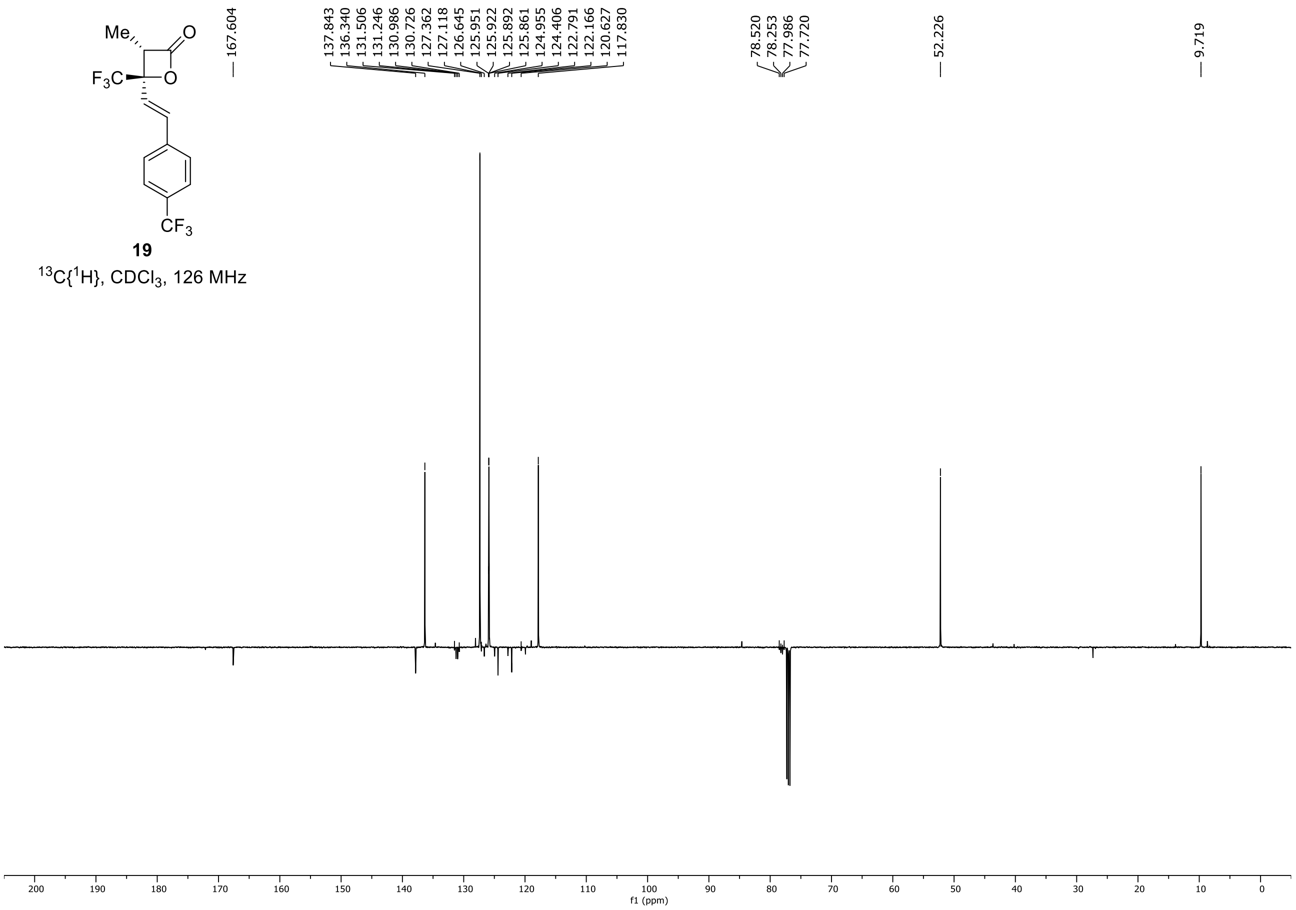
167.604
137.843
136.340
131.506
131.246
130.986
130.726
127.362
127.118
126.645
125.951
125.922
125.892
125.861
124.955
124.406
122.791
122.166
120.627
117.830
78.520
78.253
77.986
77.720
52.226
9.719
19
$^{13}C\{^{1}H\}$, $CDCl_3$, 126 MHz
f1 (ppm)

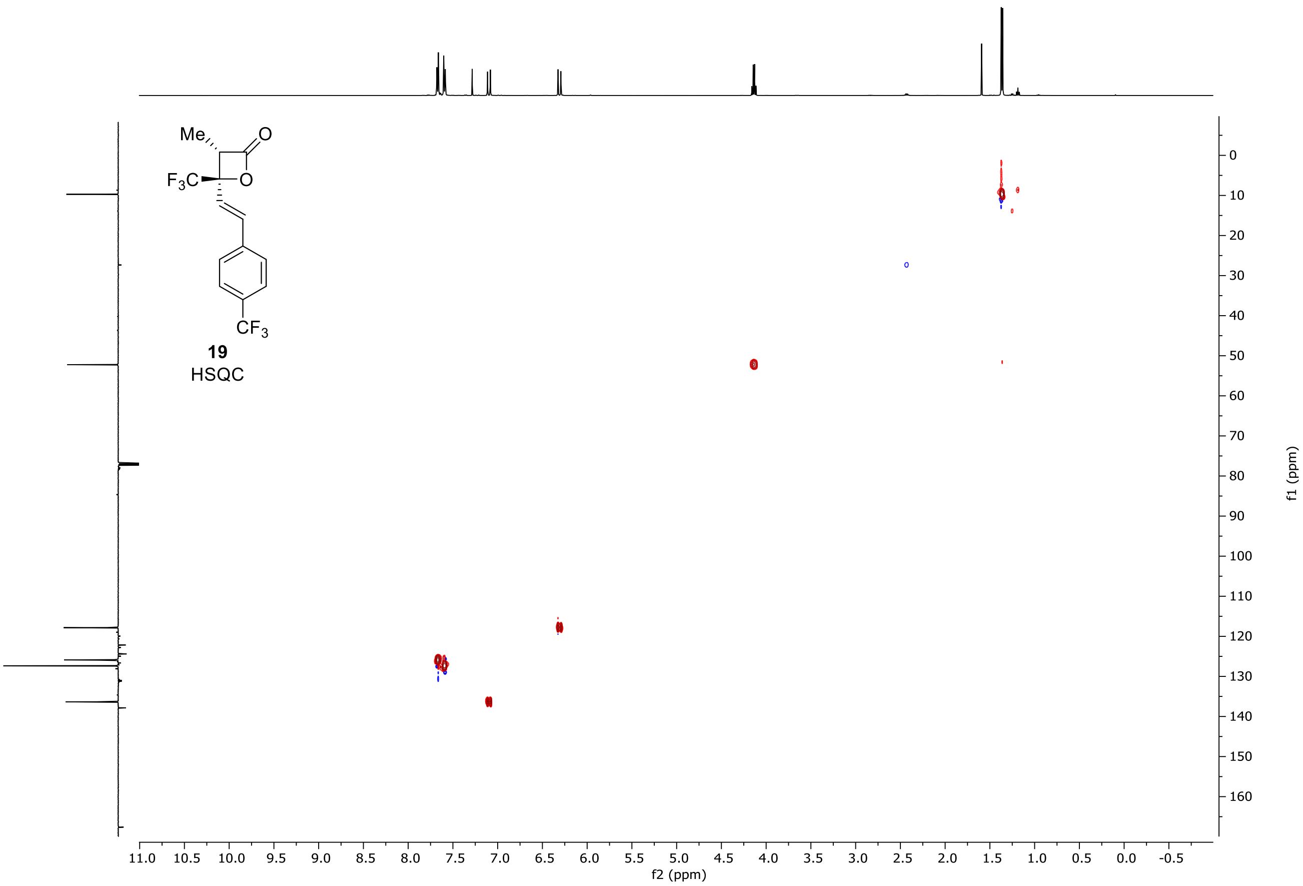
19
HSQC
f2 (ppm)
f1 (ppm)

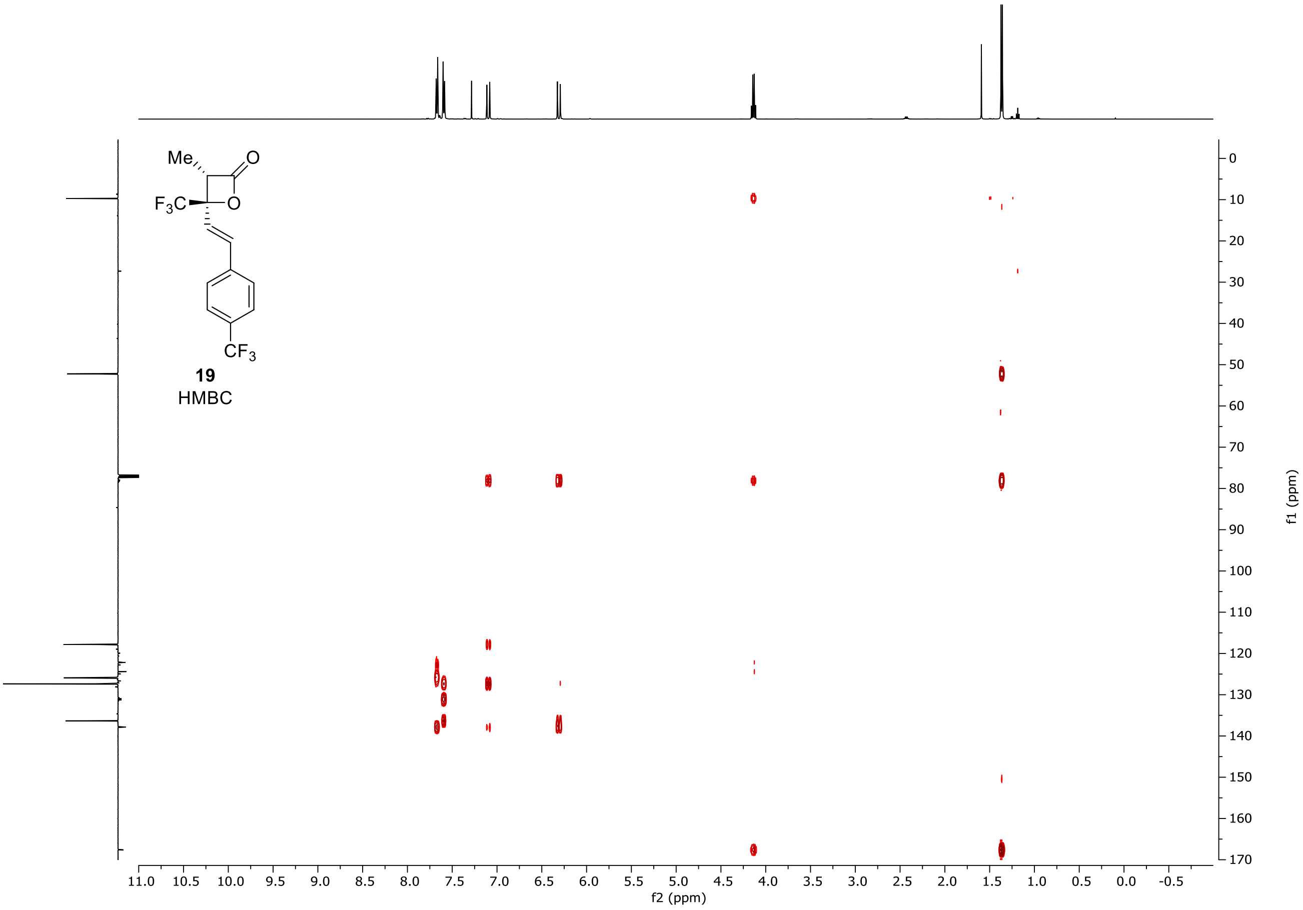
Me
O
F3C
O
CF3
19
HMBC
f2 (ppm)
f1 (ppm)

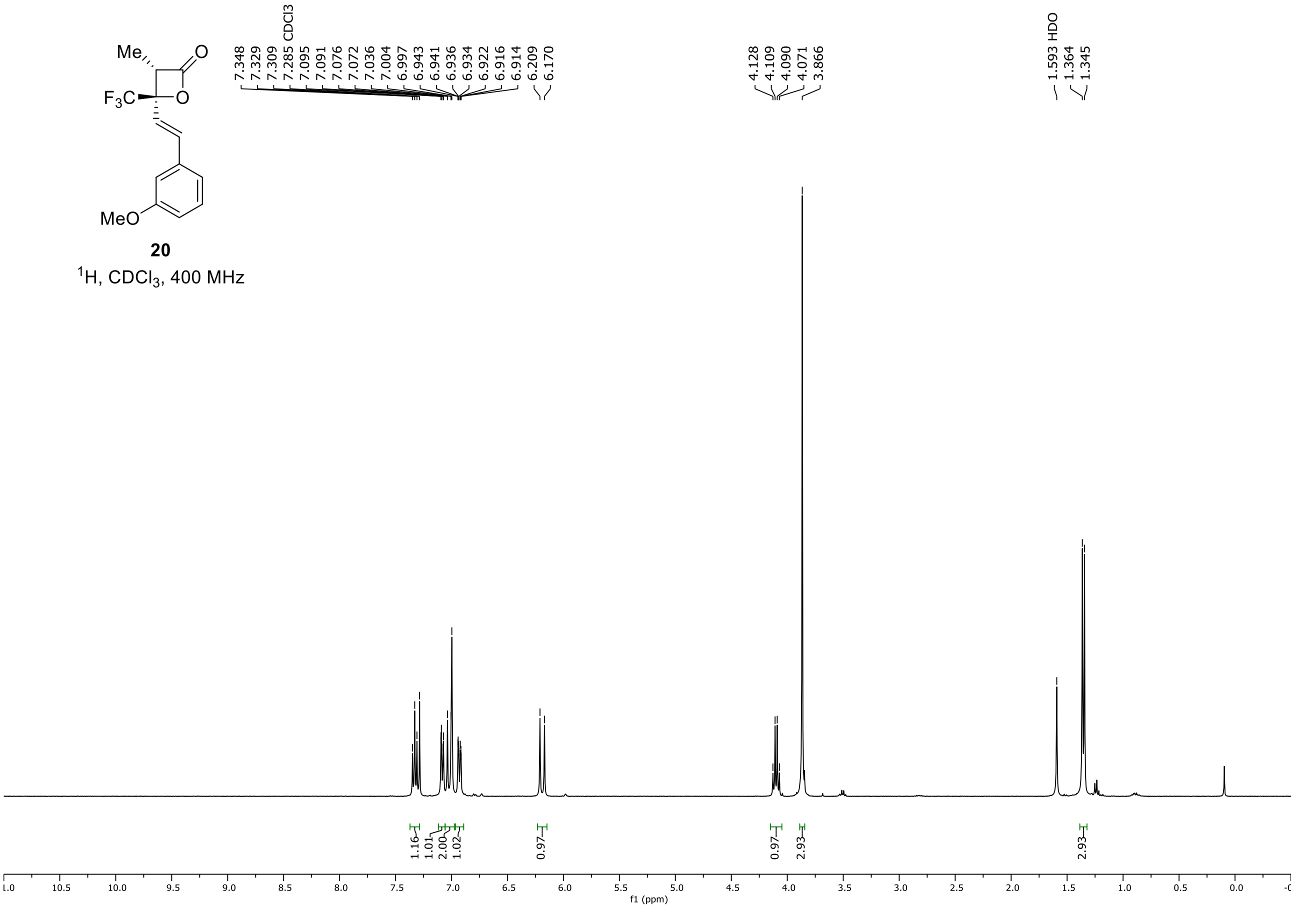
Me
O
F3C
O
MeO
20
1H, CDCl3, 400 MHz
7.348
7.329
7.309
7.285 CDCl3
7.095
7.091
7.076
7.072
7.036
7.004
6.997
6.943
6.941
6.936
6.934
6.922
6.916
6.914
6.209
6.170
4.128
4.109
4.090
4.071
3.866
1.593 HDO
1.364
1.345
1.16
1.01
2.00
1.02
0.97
0.97
2.93
2.93
f1 (ppm)

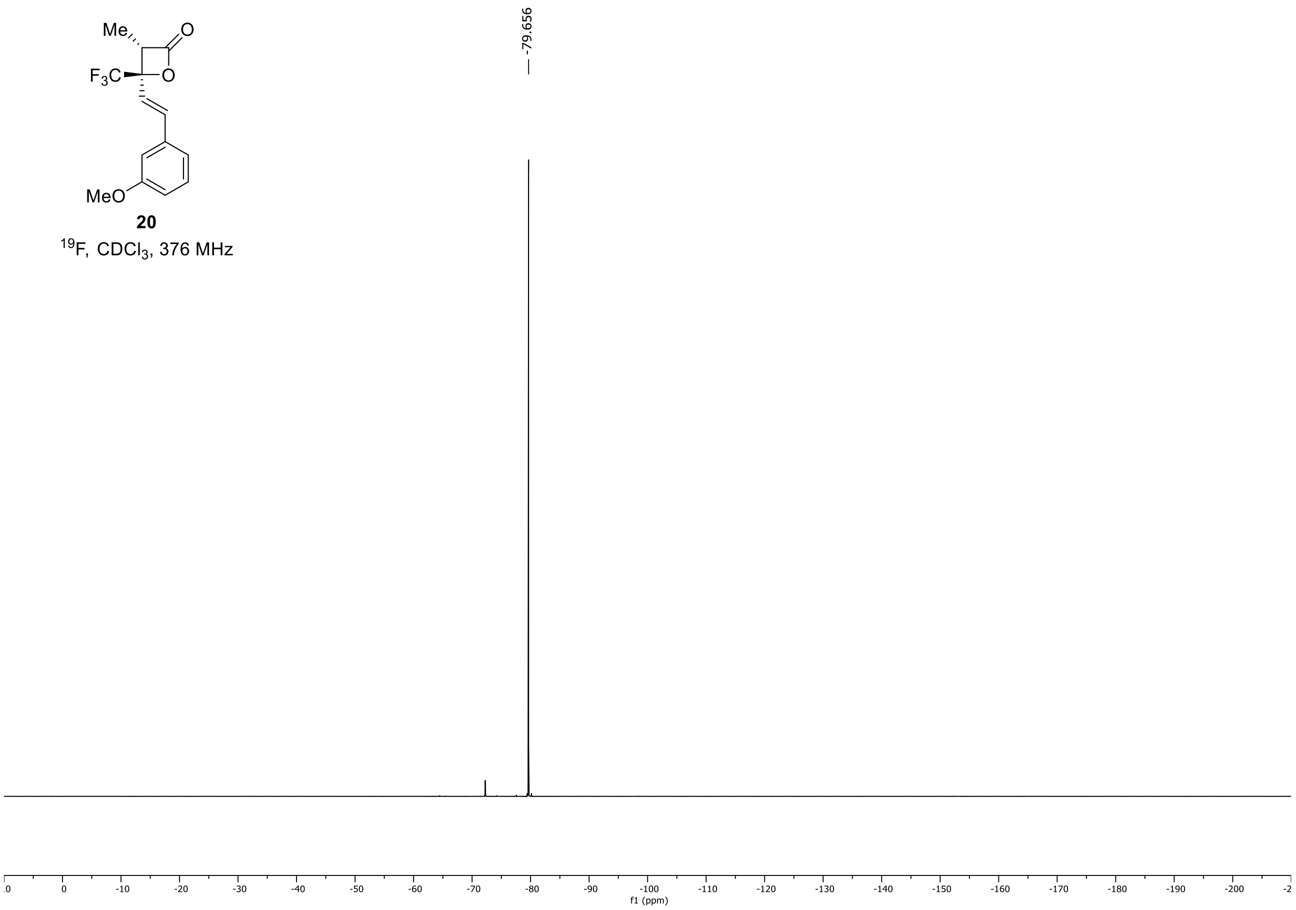
Me
O
F3C
O
MeO
20
19F, CDCl3, 376 MHz
-79.656
f1 (ppm)

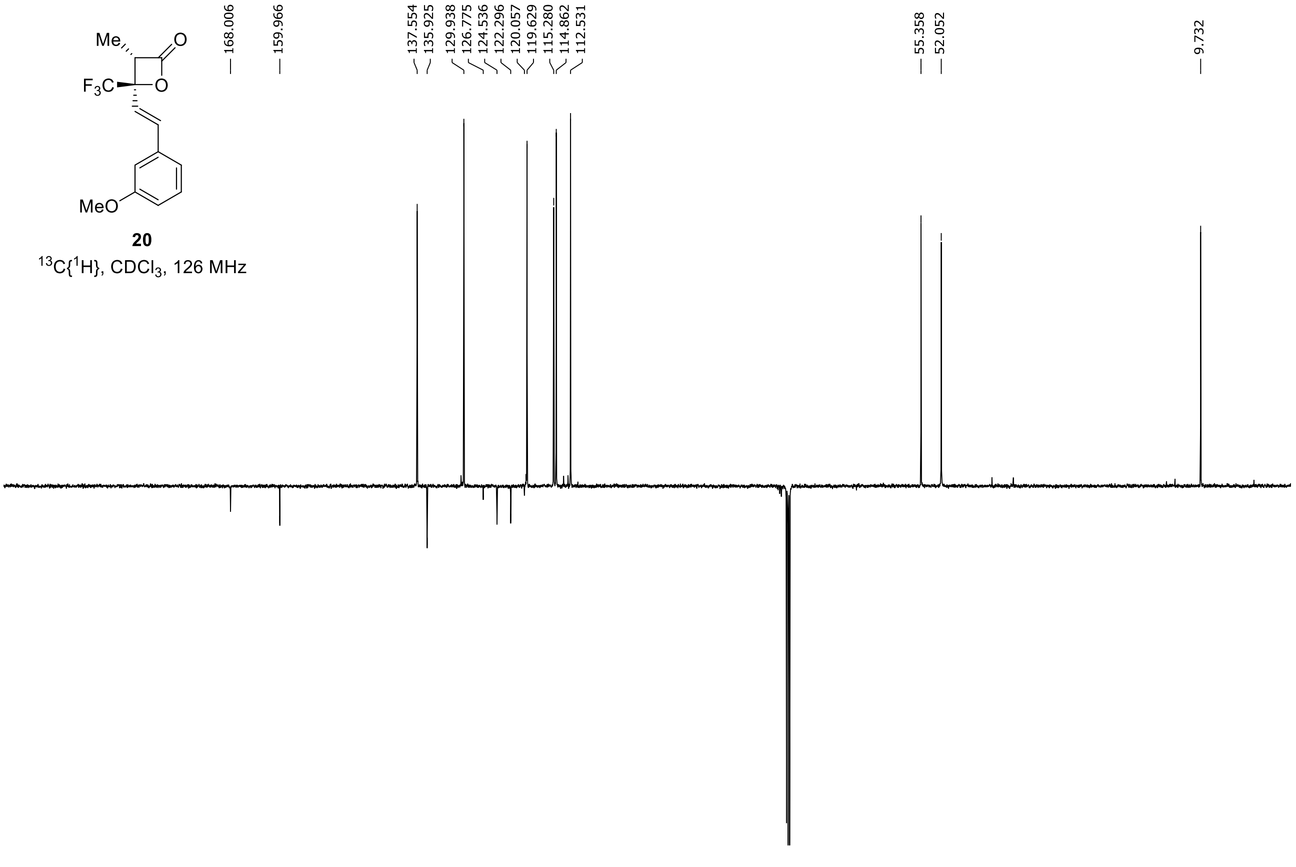
Me
O
F3C
O
MeO
20
13C{1H}, CDCl3, 126 MHz
168.006
159.966
137.554
135.925
129.938
126.775
124.536
122.296
120.057
119.629
115.280
114.862
112.531
55.358
52.052
9.732
f1 (ppm)

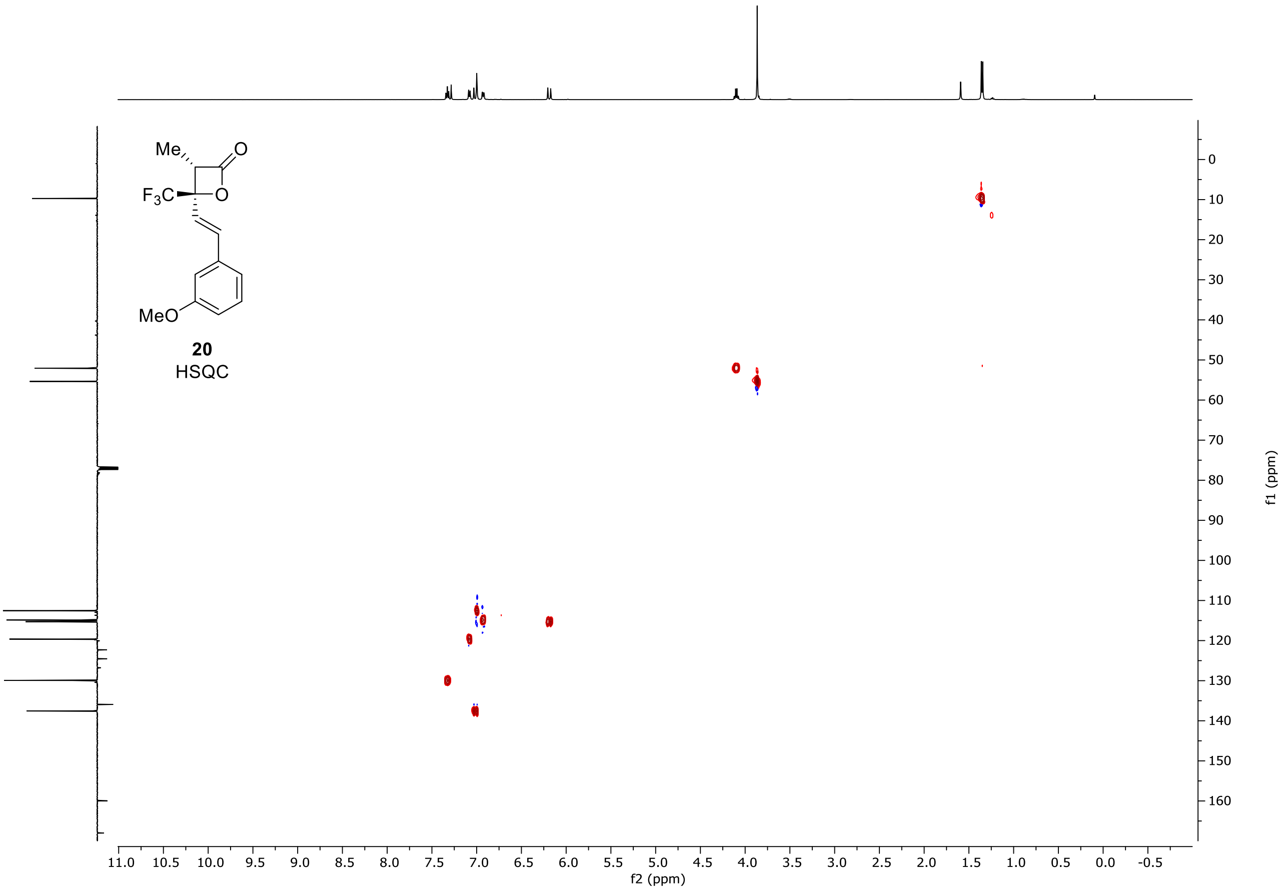
Me
O
F3C
O
MeO
20
HSQC
f1 (ppm)
f2 (ppm)

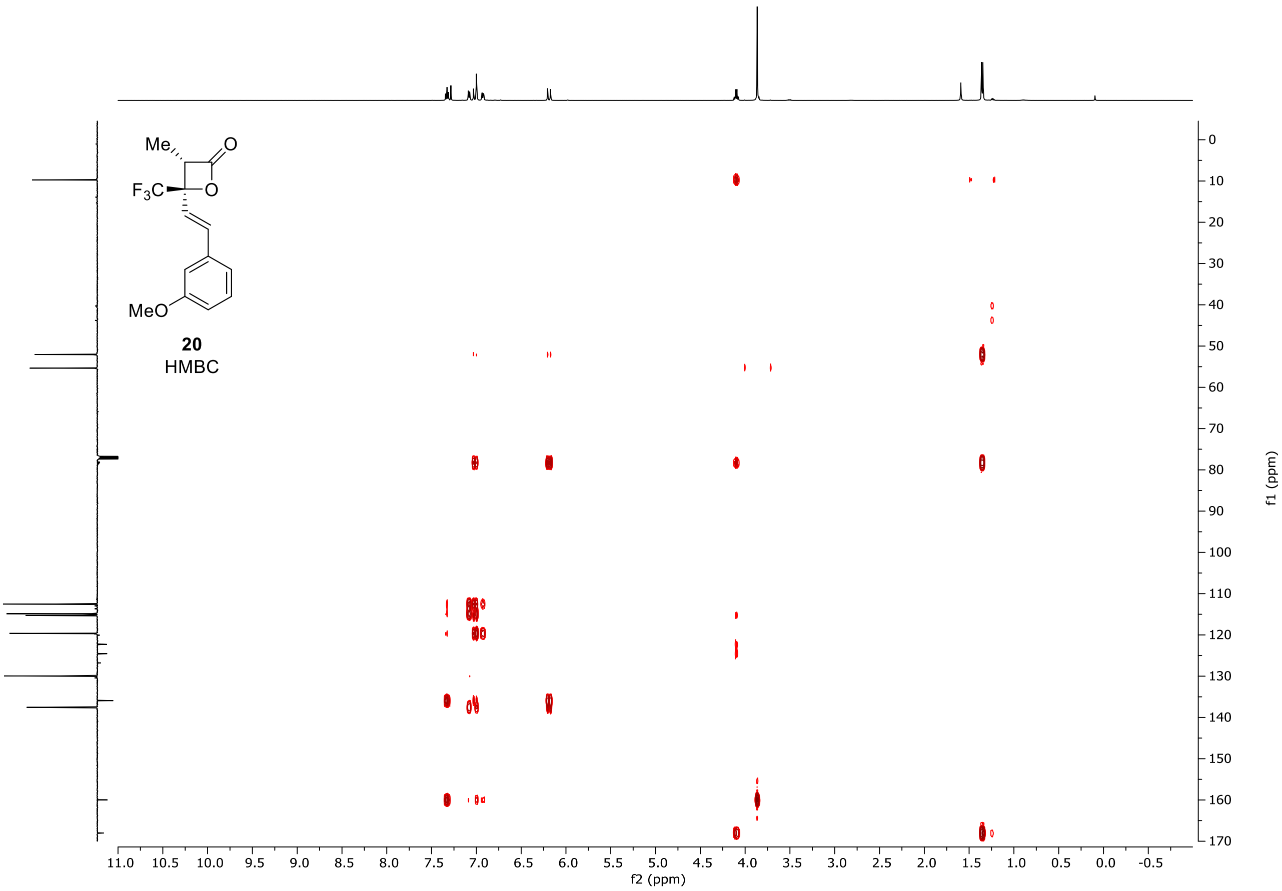
Me
O
F3C
O
MeO
20
HMBC
f1 (ppm)
f2 (ppm)

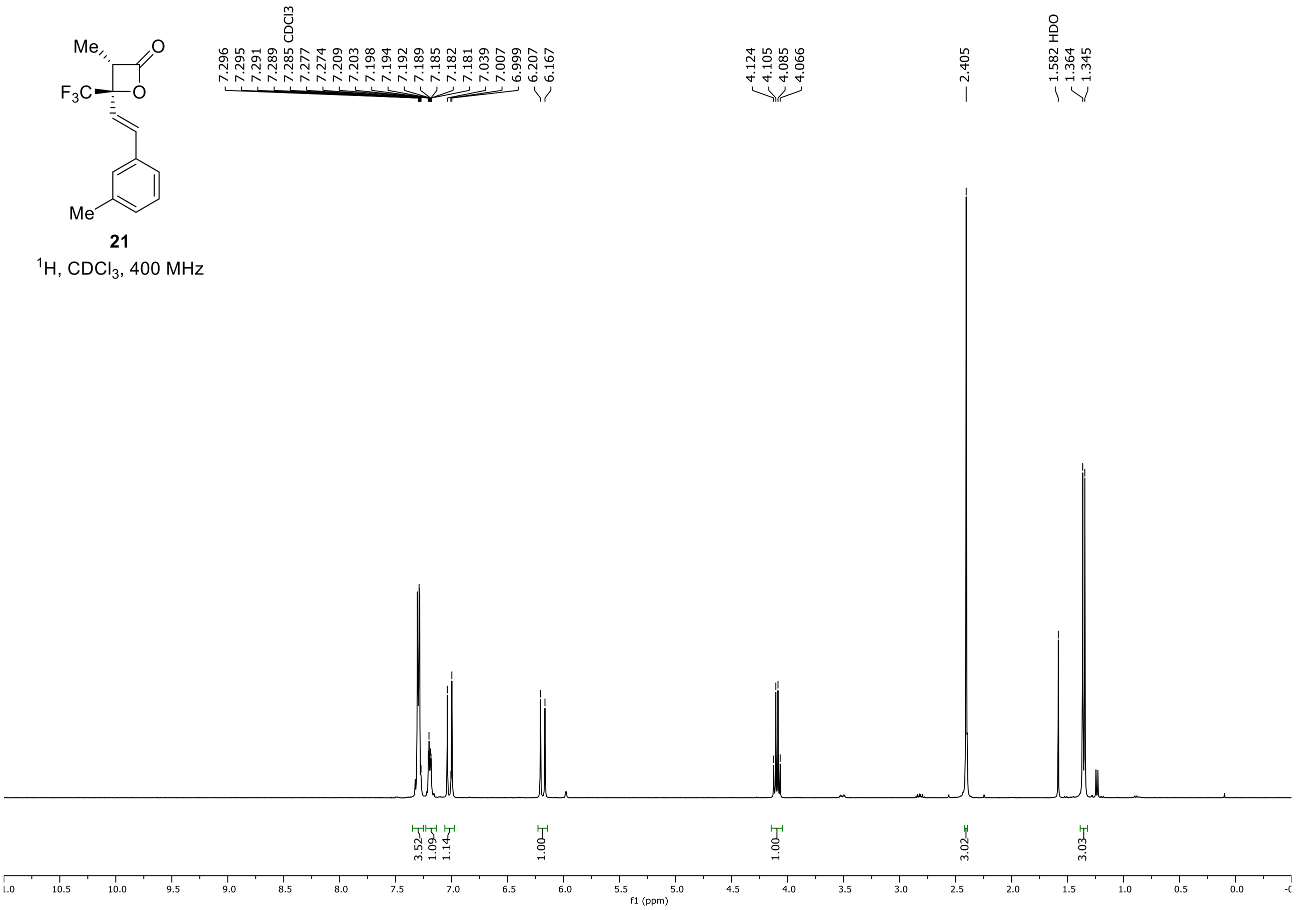
Me
O
F3C
O
Me
21
1H, CDCl3, 400 MHz
7.296 7.295 7.291 7.289 7.285 CDCl3 7.277 7.274 7.209 7.203 7.198 7.194 7.192 7.189 7.185 7.182 7.181 7.039 7.007 6.999 6.207 6.167
4.124 4.105 4.085 4.066
2.405
1.582 HDO 1.364 1.345
3.52 1.09 1.14 1.00 1.00 3.02 3.03
f1 (ppm)

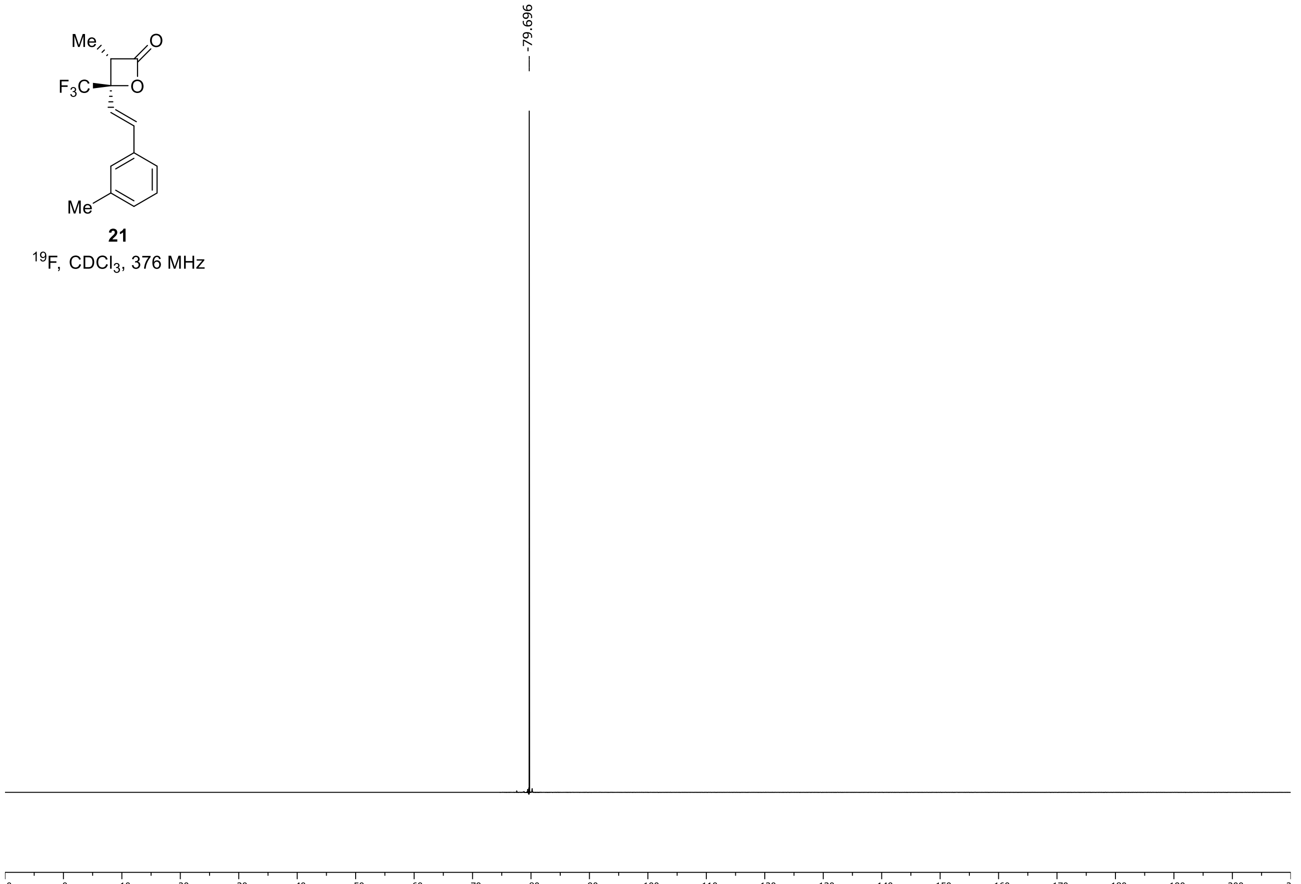
Me
O
F3C
O
Me
21
19F, CDCl3, 376 MHz
-79.696
f1 (ppm)

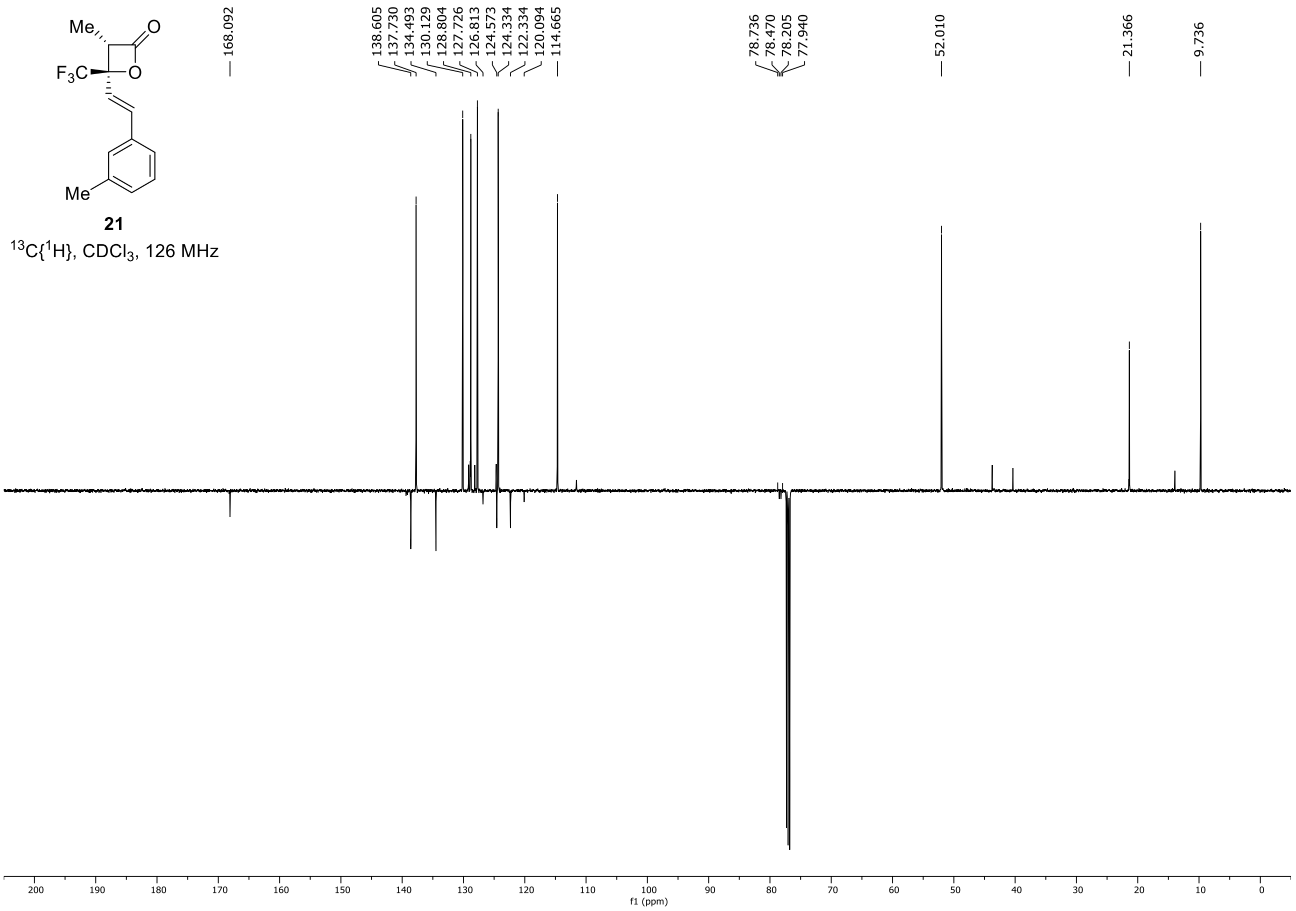
Me
O
F3C
O
Me
21
13C{1H}, CDCl3, 126 MHz
168.092
138.605
137.730
134.493
130.129
128.804
127.726
126.813
124.573
124.334
122.334
120.094
114.665
78.736
78.470
78.205
77.940
52.010
21.366
9.736
f1 (ppm)

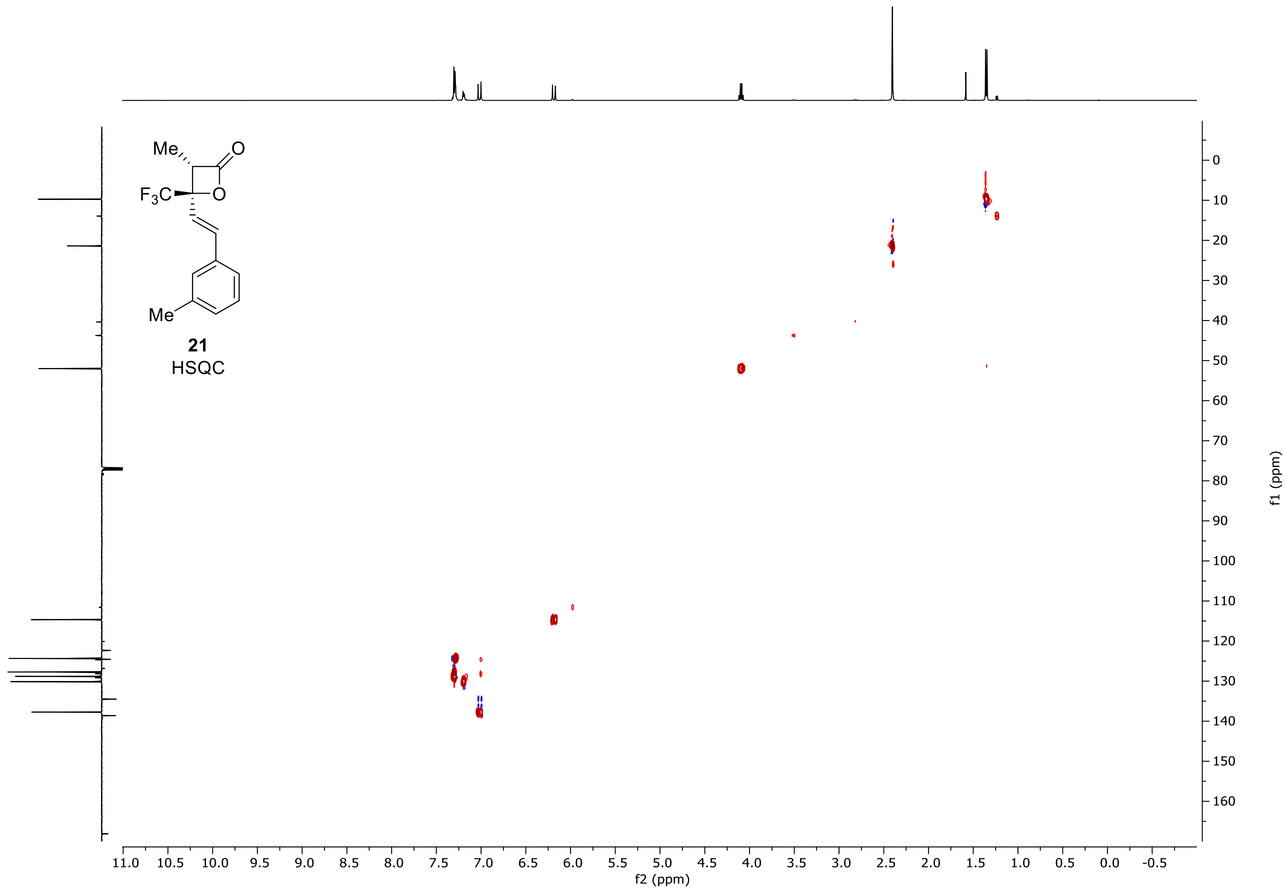
Me
O
F3C
O
Me
21
HSQC
f2 (ppm)
f1 (ppm)

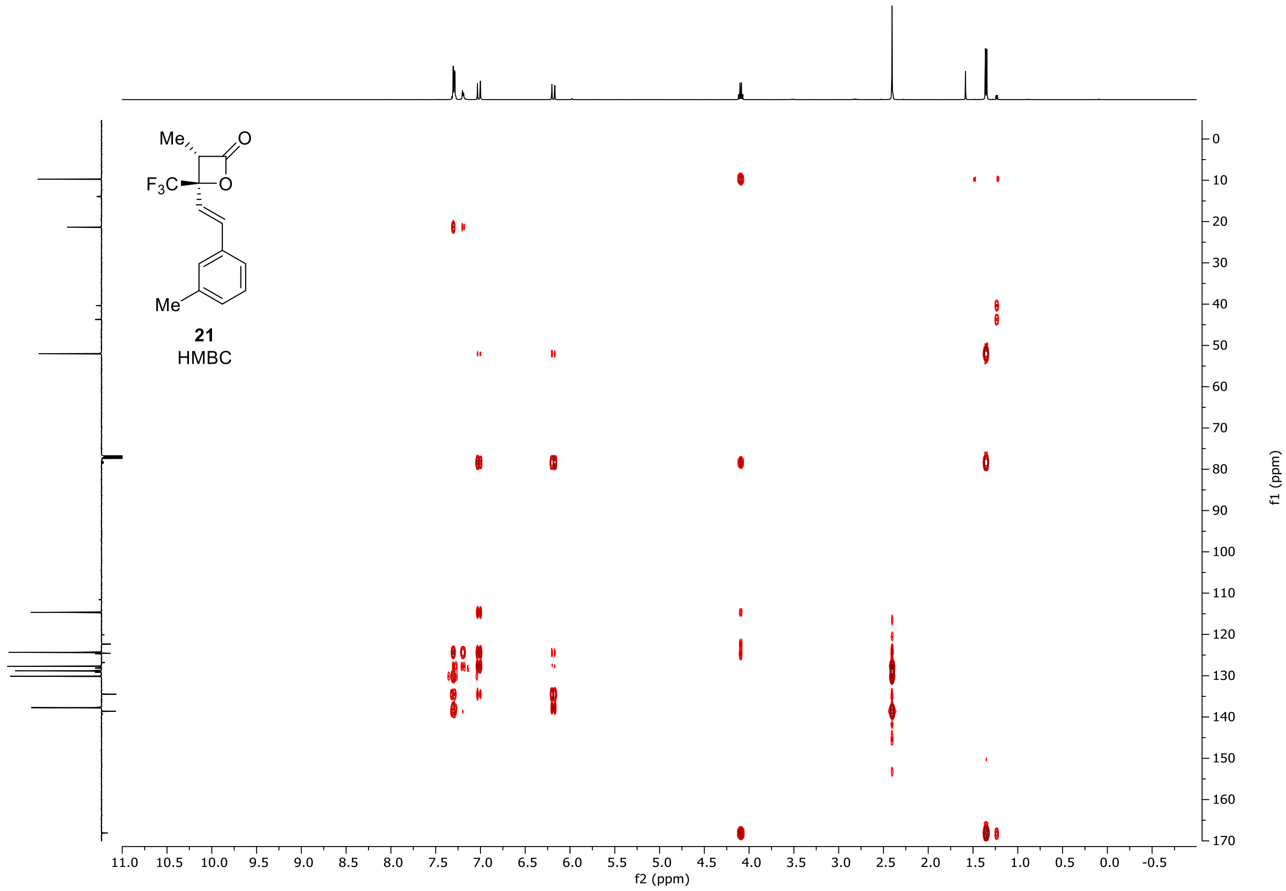
Me
O
F_3C
O
Me
21
HMBC
f1 (ppm)
f2 (ppm)

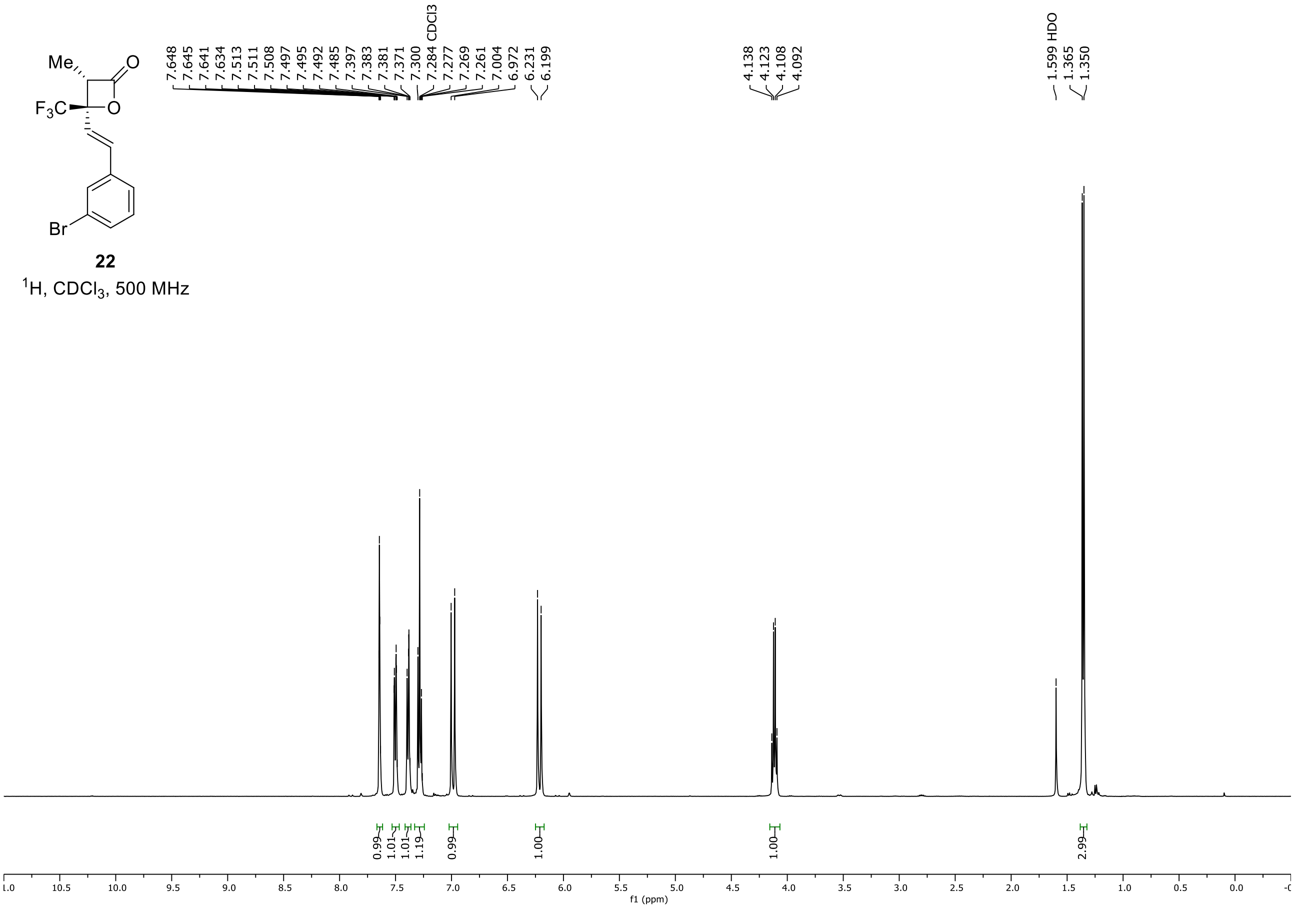
Me
O
F_3C
O
Br
22
^{1}H, $CDCl_3$, 500 MHz
7.648
7.645
7.641
7.634
7.513
7.511
7.508
7.497
7.495
7.492
7.485
7.397
7.383
7.381
7.371
7.300
7.284 CDCl3
7.277
7.269
7.261
7.004
6.972
6.231
6.199
4.138
4.123
4.108
4.092
1.599 HDO
1.365
1.350
0.99
1.01
1.01
1.19
0.99
1.00
1.00
2.99
f1 (ppm)

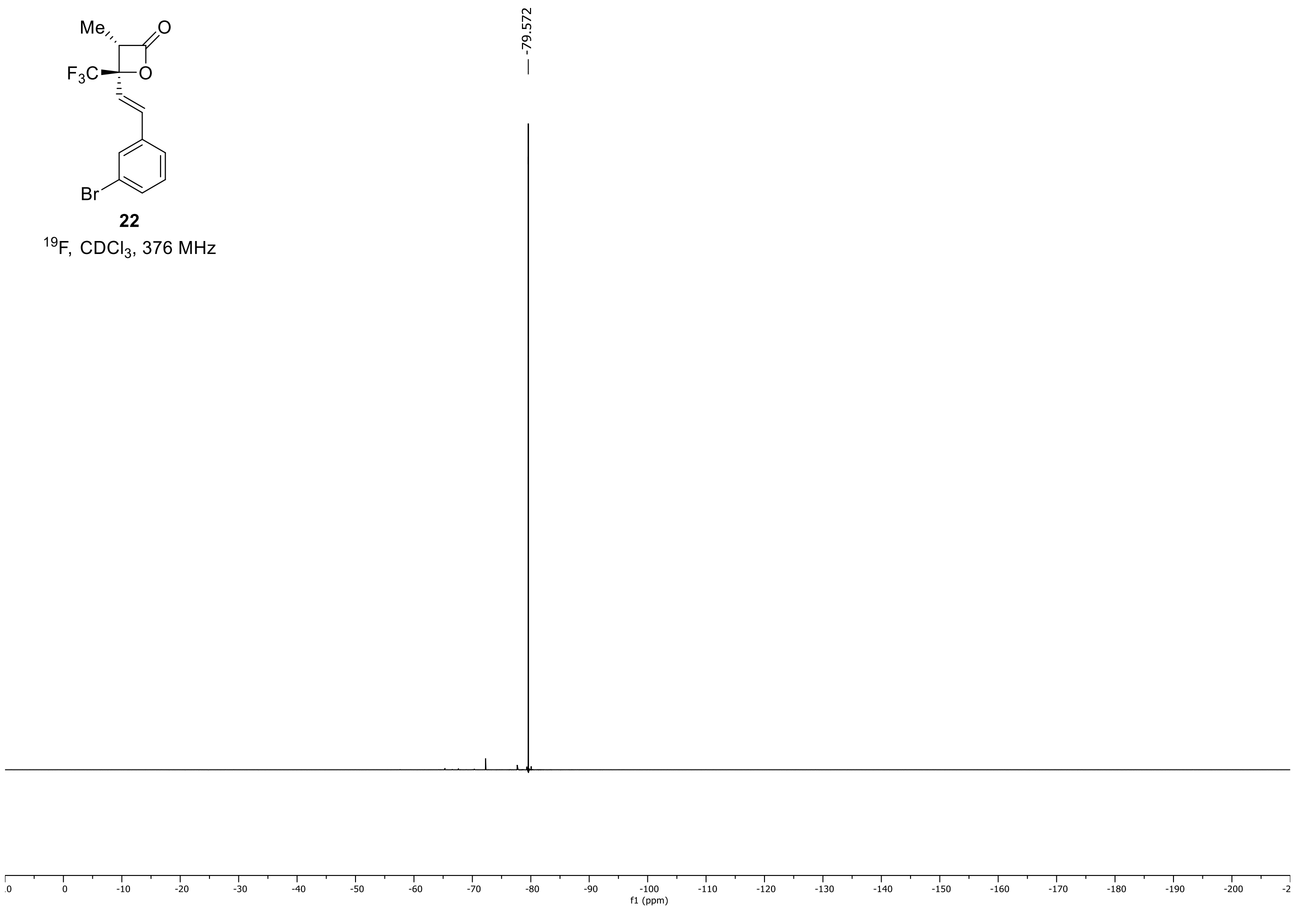

22

^{19}F, $CDCl_3$, 376 MHz

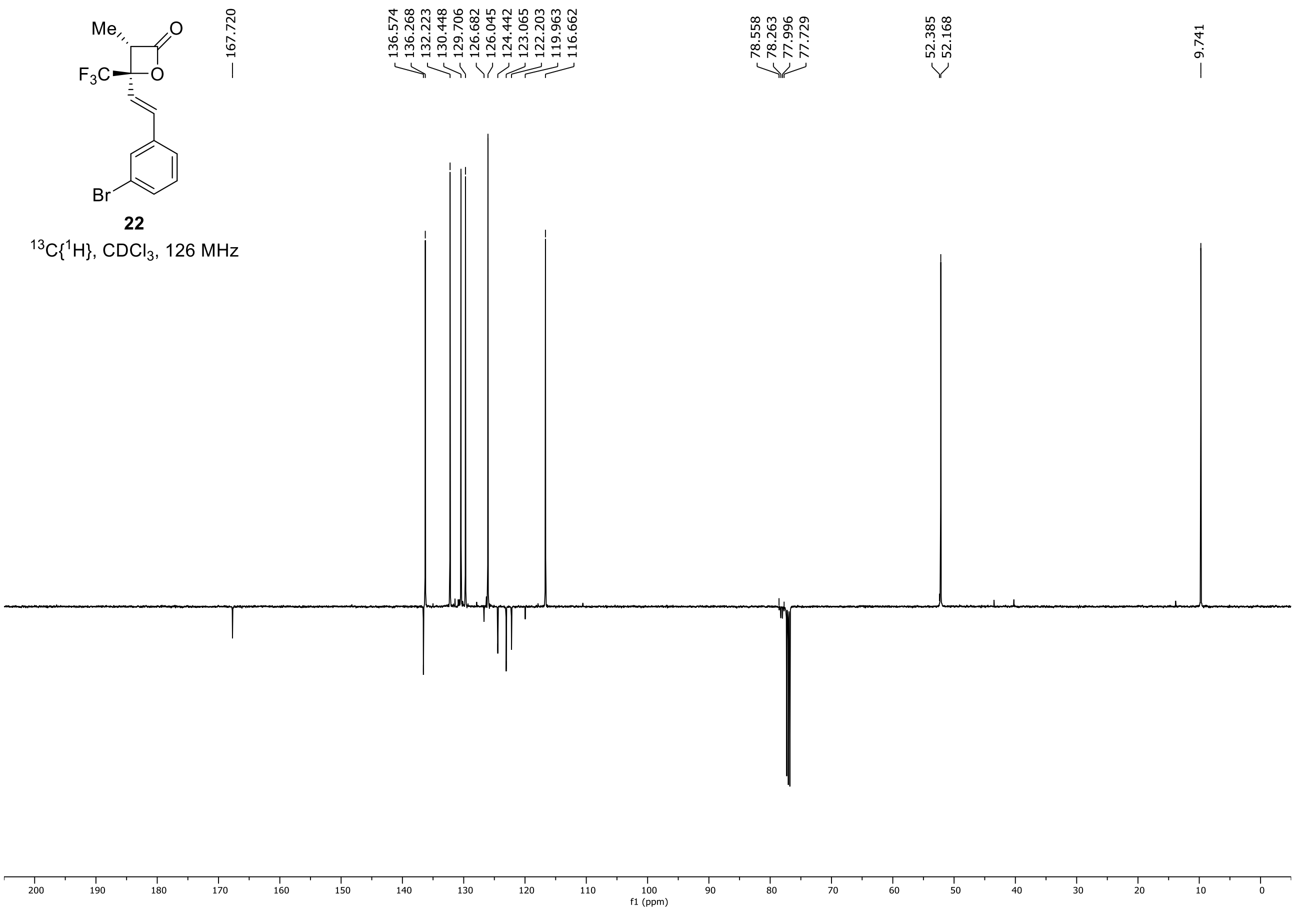

22

$^{13}C\{^1H\}$, $CDCl_3$, 126 MHz

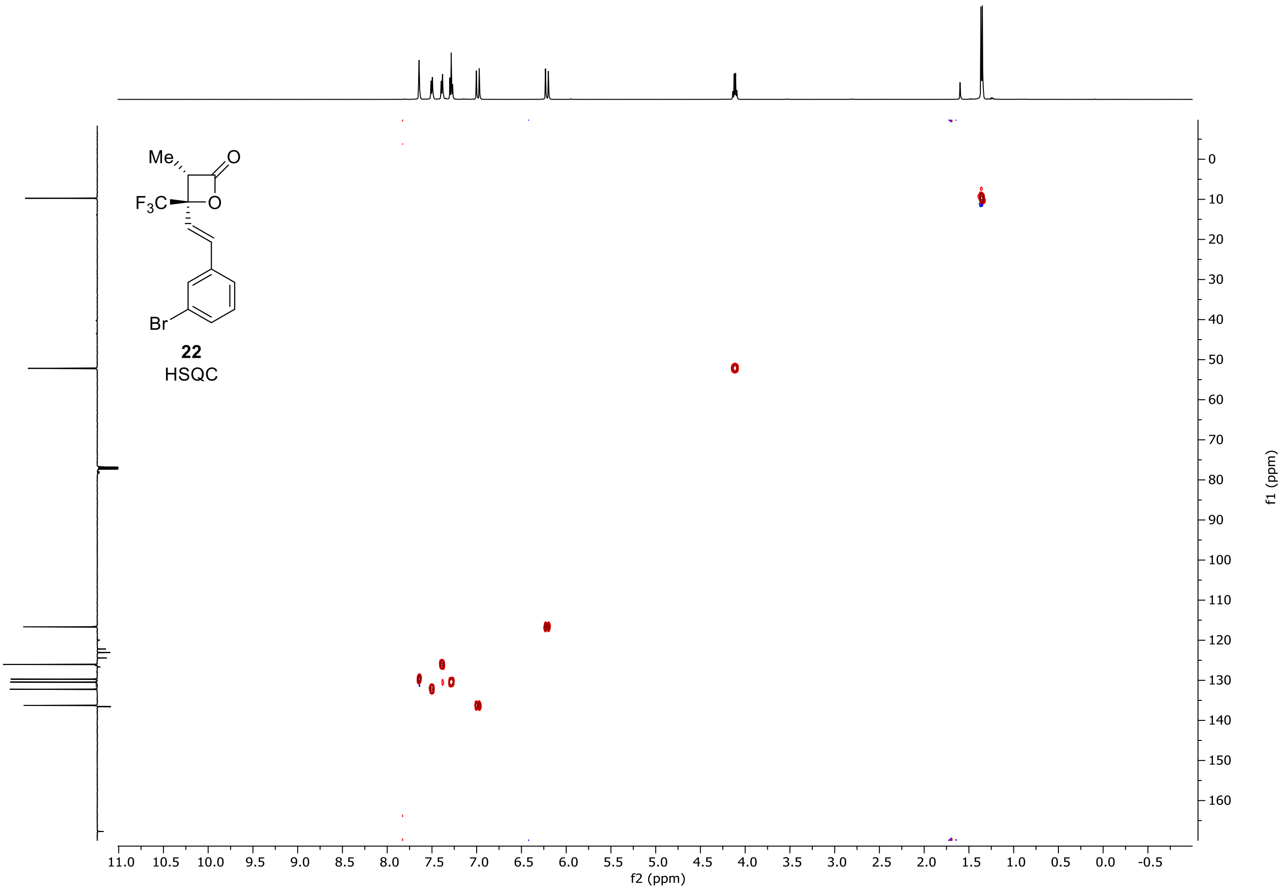

Me
O
F3C
O
Br
22
HSQC
f1 (ppm)
f2 (ppm)

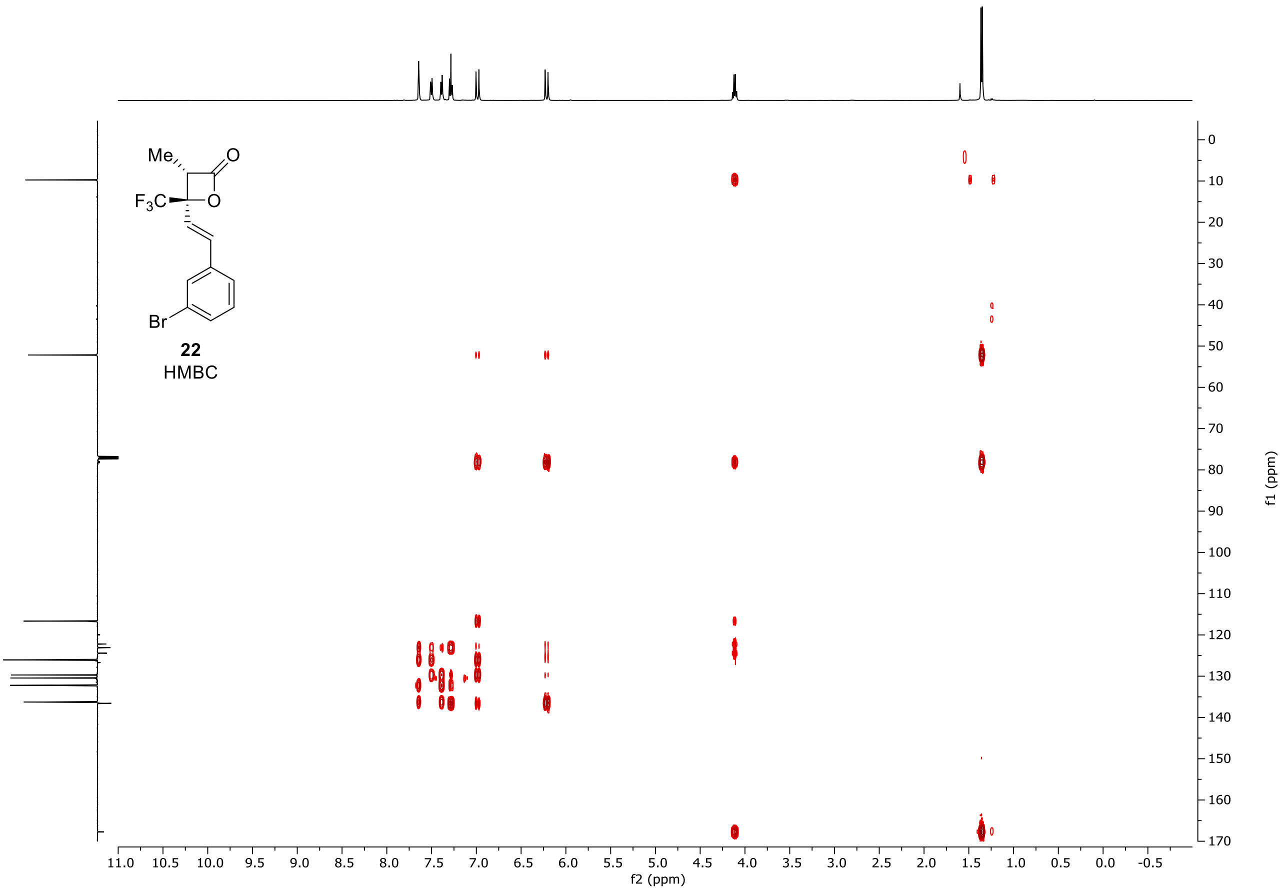

Me
O
F3C
O
Br
22
HMBC
f1 (ppm)
f2 (ppm)

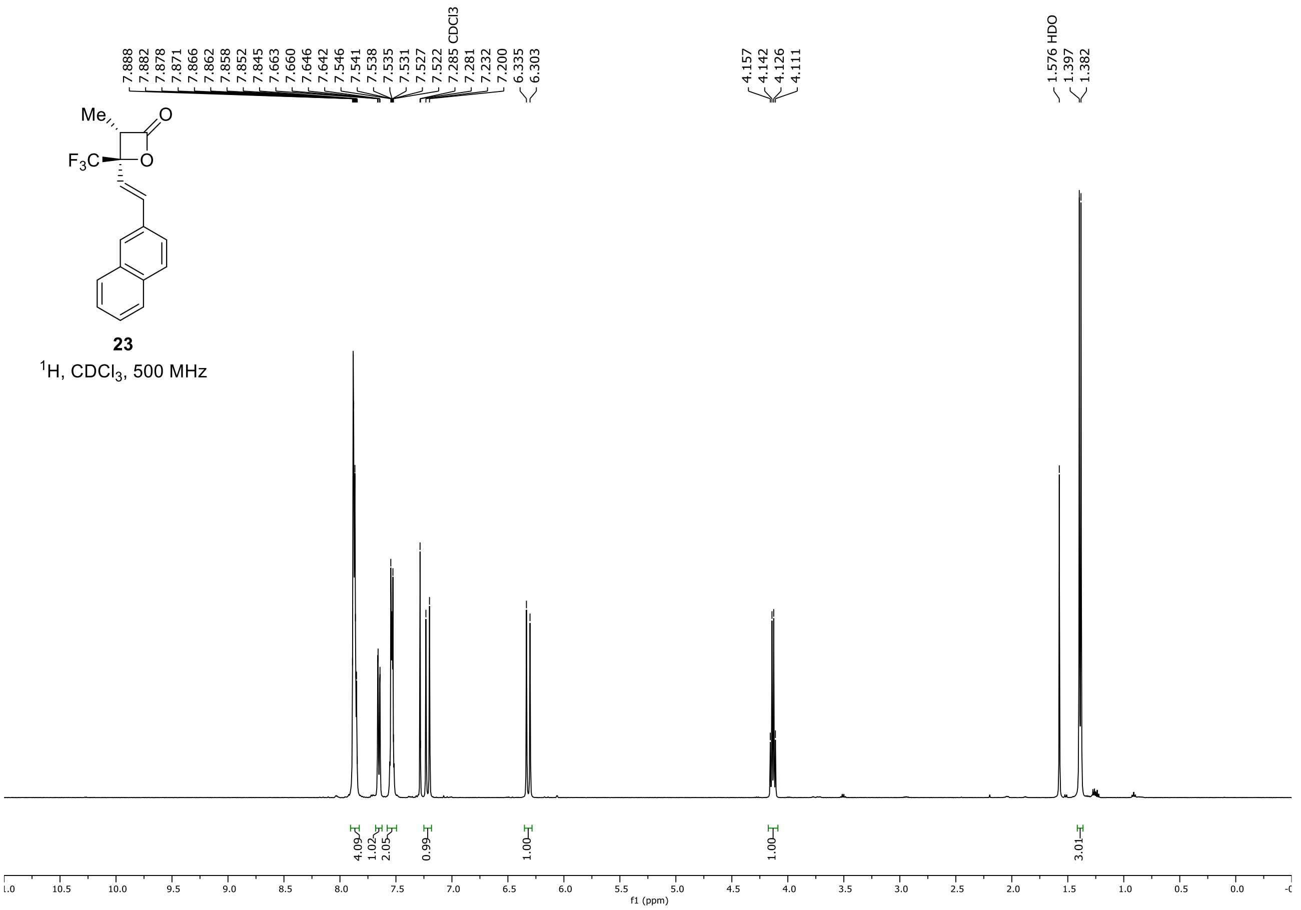

23

^{1}H, $CDCl_3$, 500 MHz

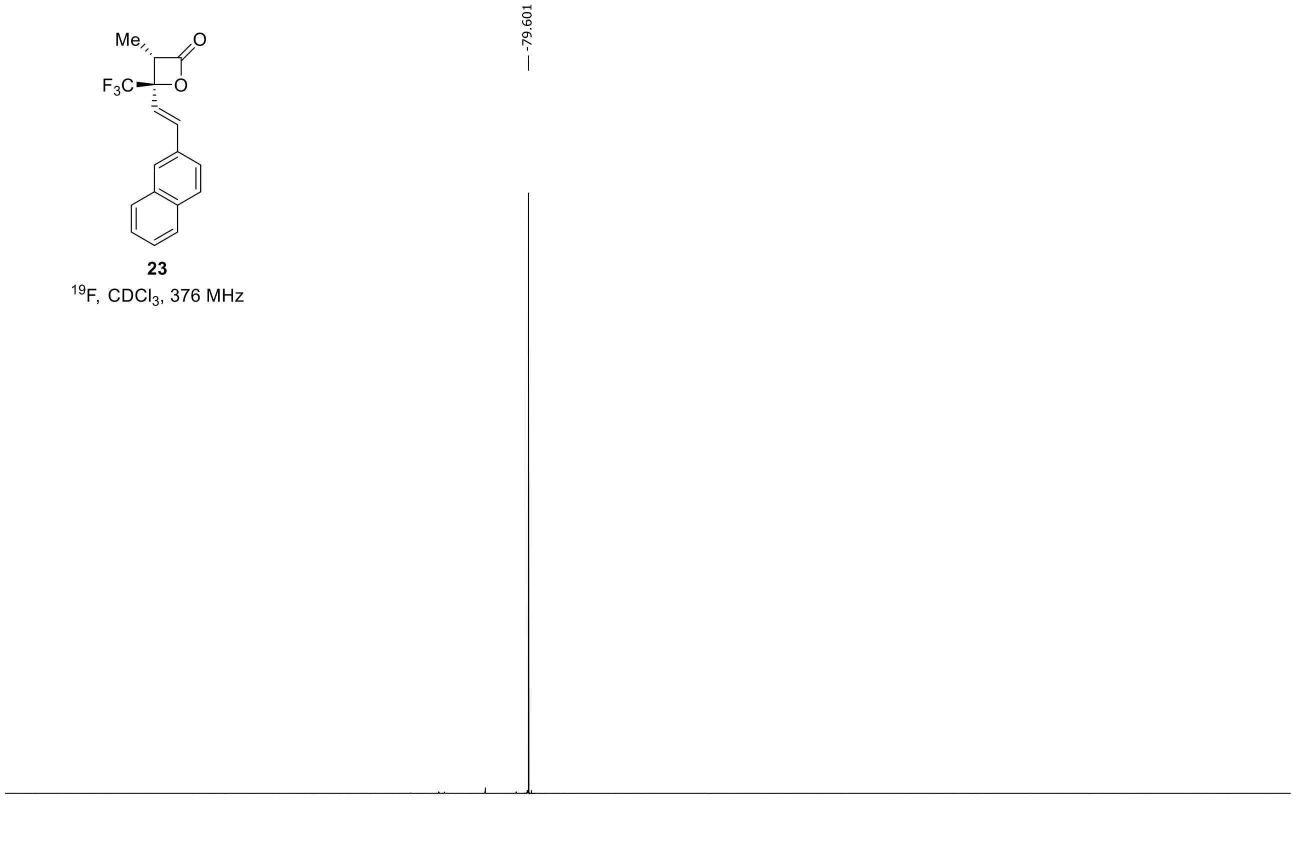

23

^{19}F, $CDCl_3$, 376 MHz

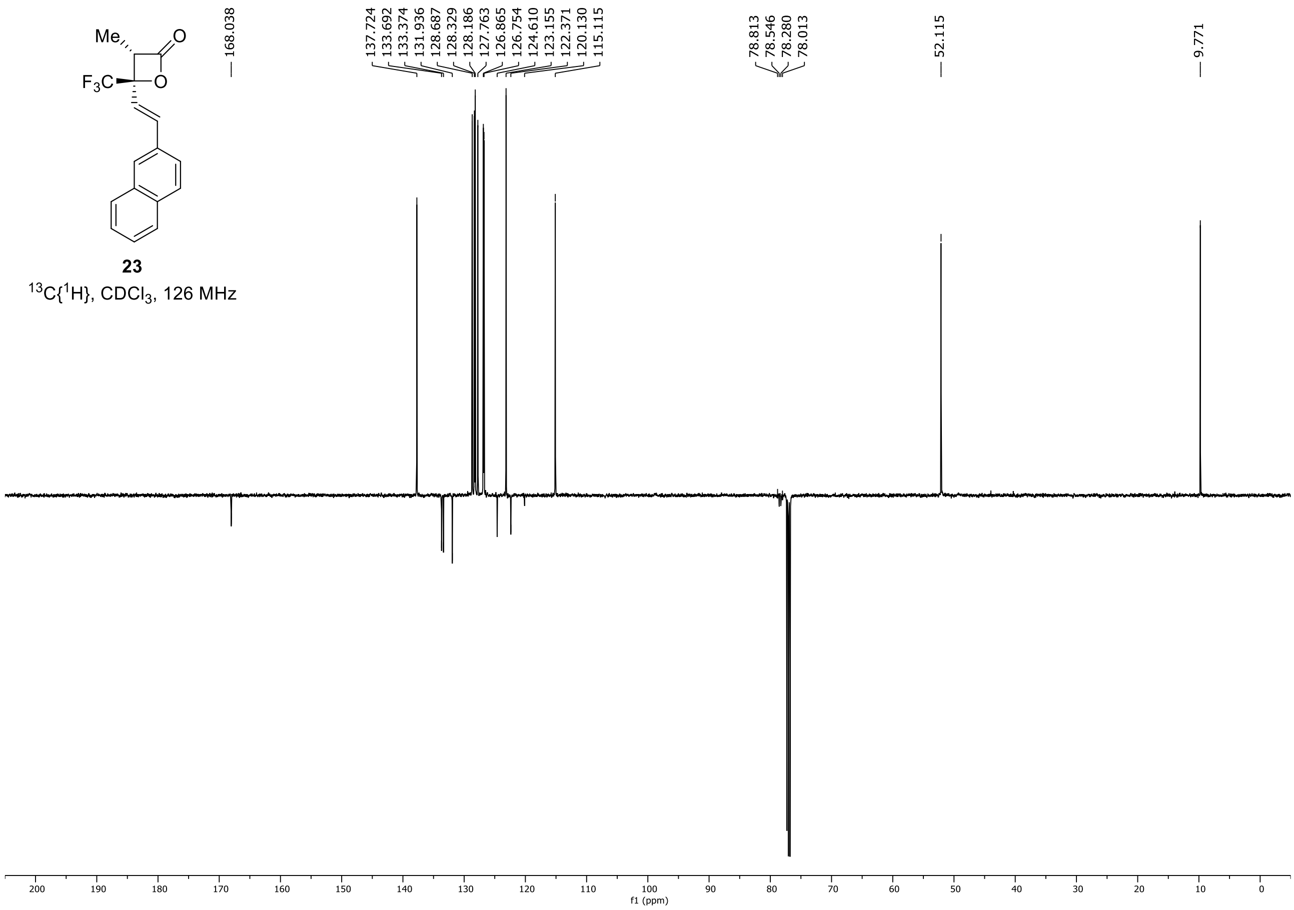

23

$^{13}C\{^{1}H\}$, $CDCl_3$, 126 MHz

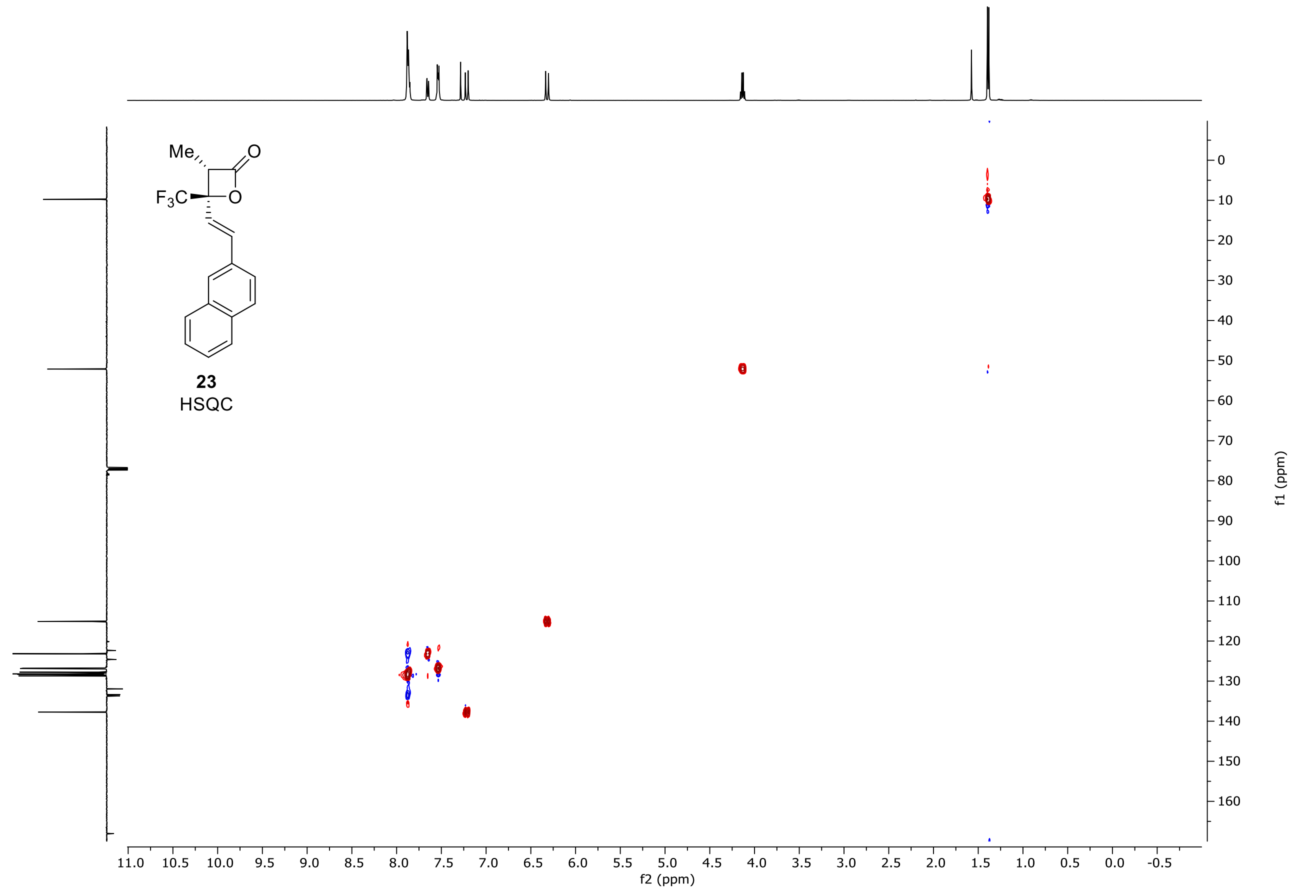

23

HSQC

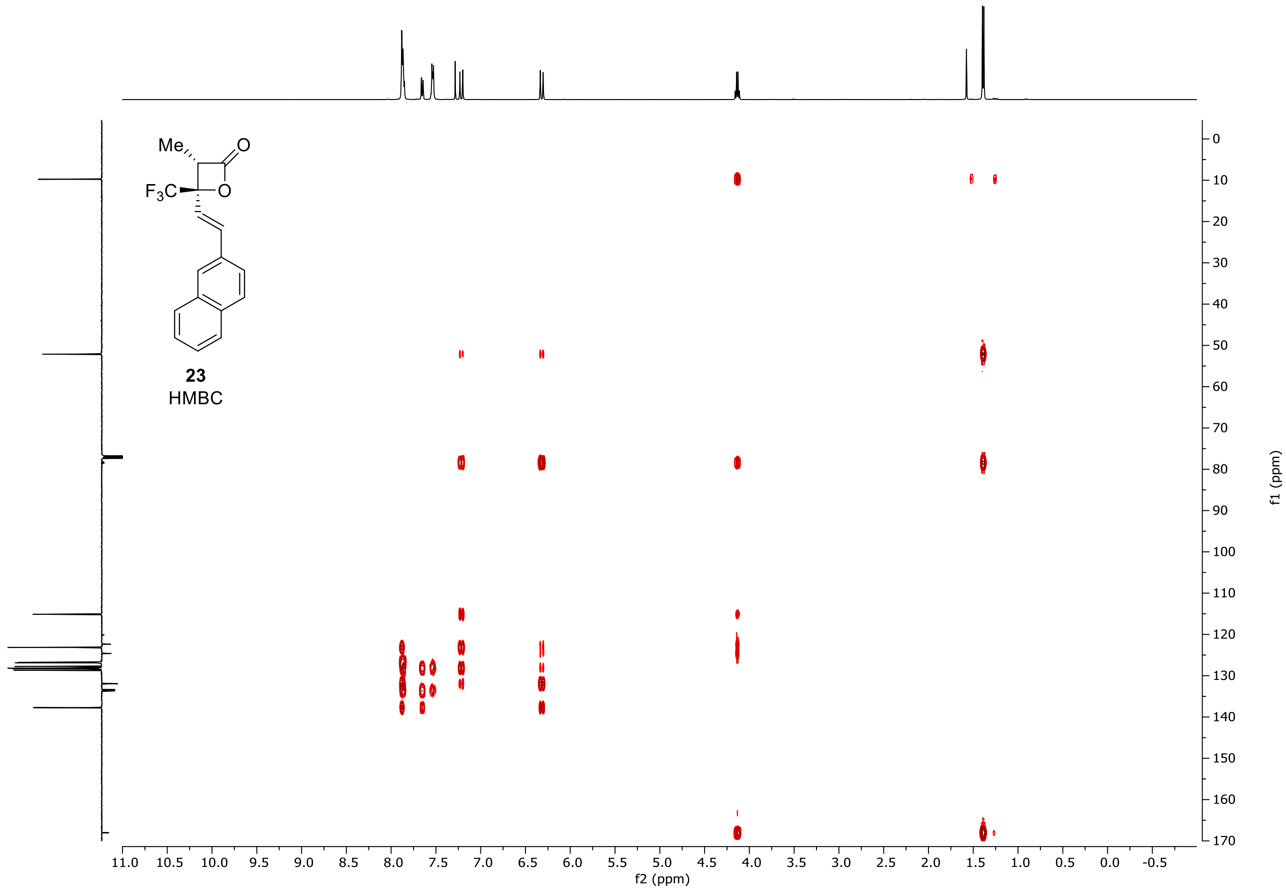
Me
O
F3C
O
23
HMBC
f2 (ppm)
f1 (ppm)

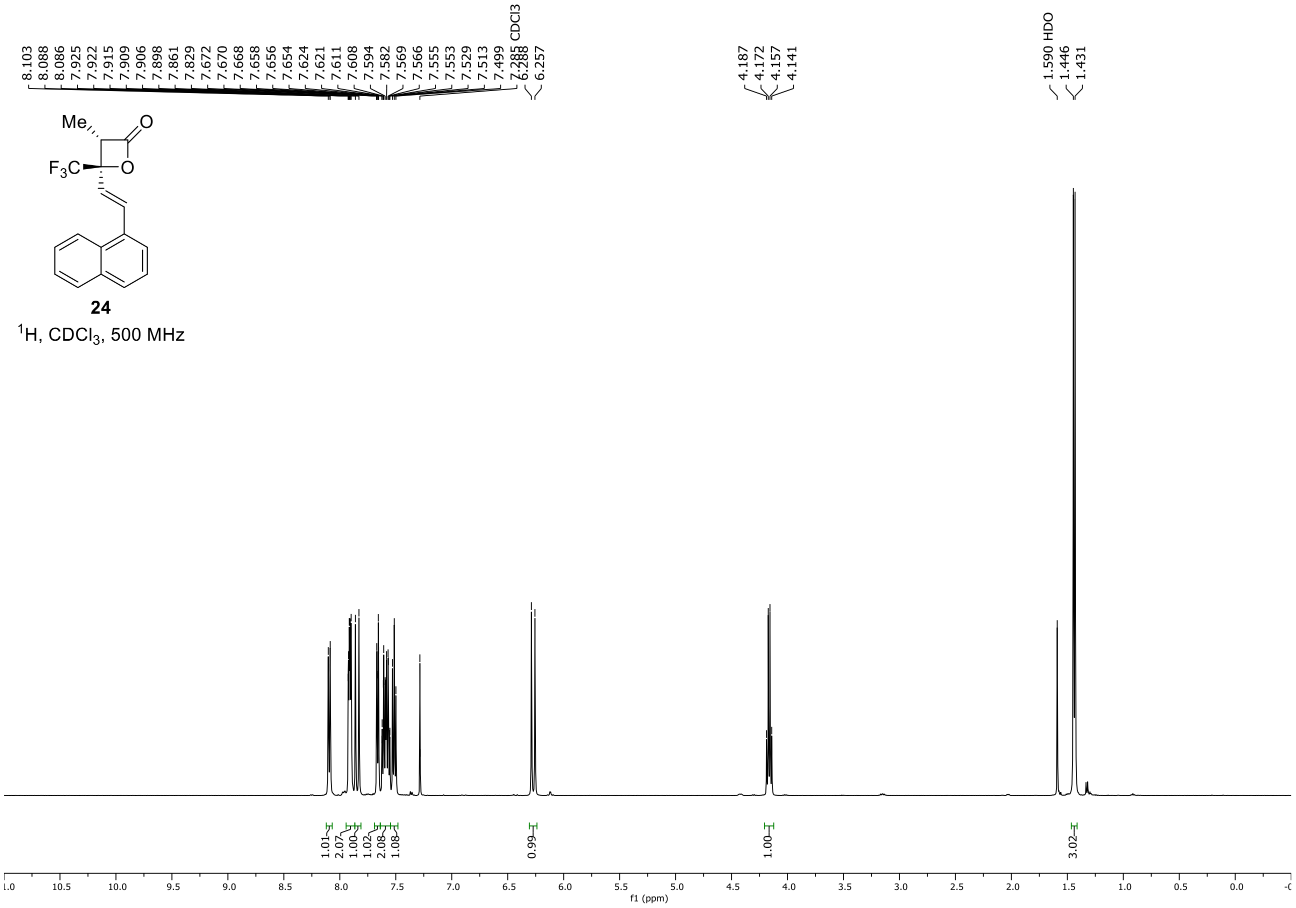
8.103
8.088
8.086
7.925
7.922
7.915
7.909
7.906
7.898
7.861
7.829
7.672
7.670
7.668
7.658
7.656
7.654
7.624
7.621
7.611
7.608
7.594
7.582
7.569
7.566
7.555
7.553
7.529
7.513
7.499
7.285 CDCl3
6.288
6.257
4.187
4.172
4.157
4.141
1.590 HDO
1.446
1.431
Me
O
F3C
O
24
1H, CDCl3, 500 MHz
1.01
2.07
1.00
1.02
2.08
1.08
0.99
1.00
3.02
f1 (ppm)

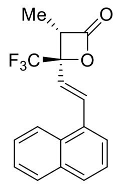

24

^{19}F, $CDCl_3$, 376 MHz

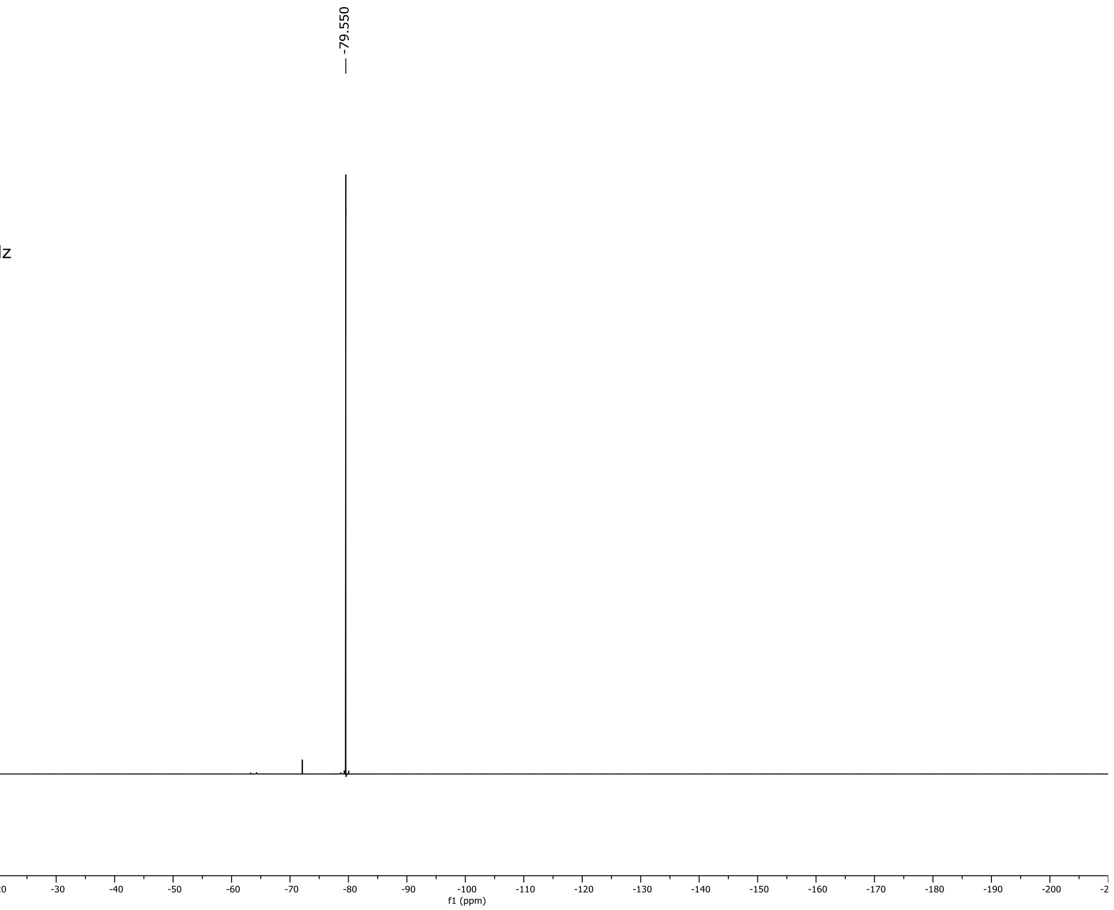

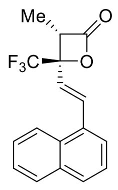

24

$^{13}C\{^1H\}$, $CDCl_3$, 126 MHz

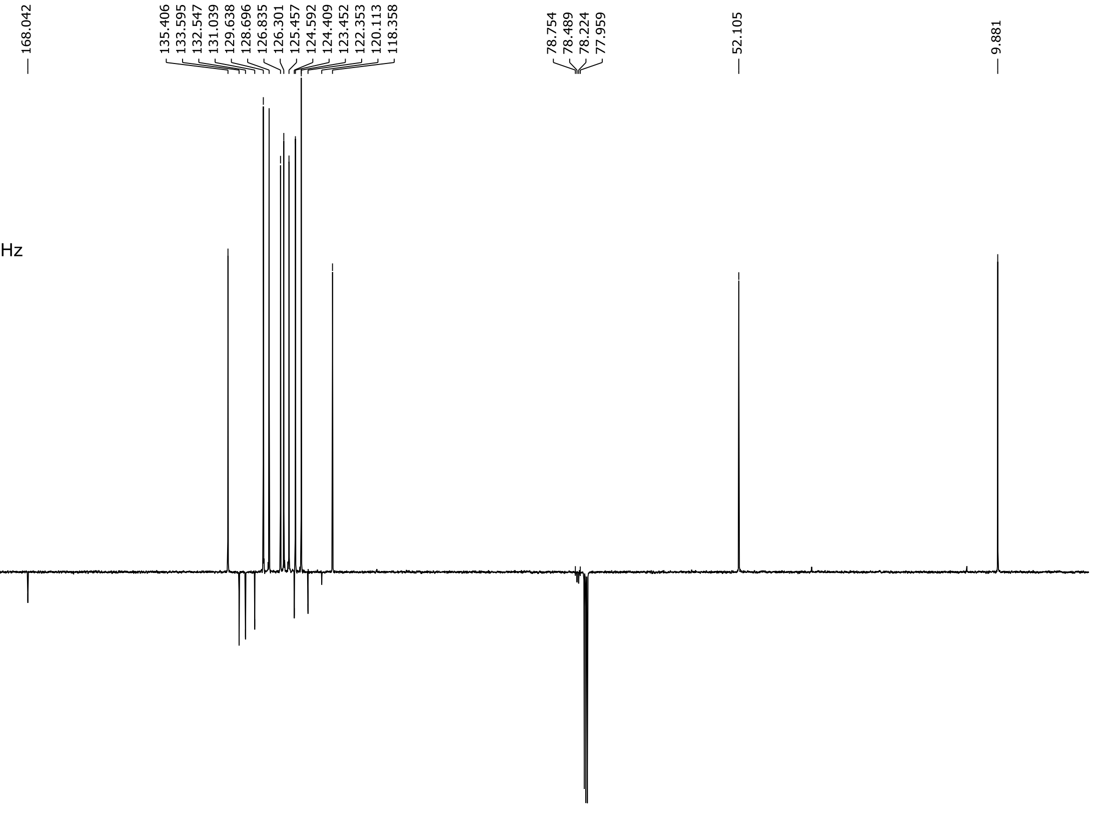

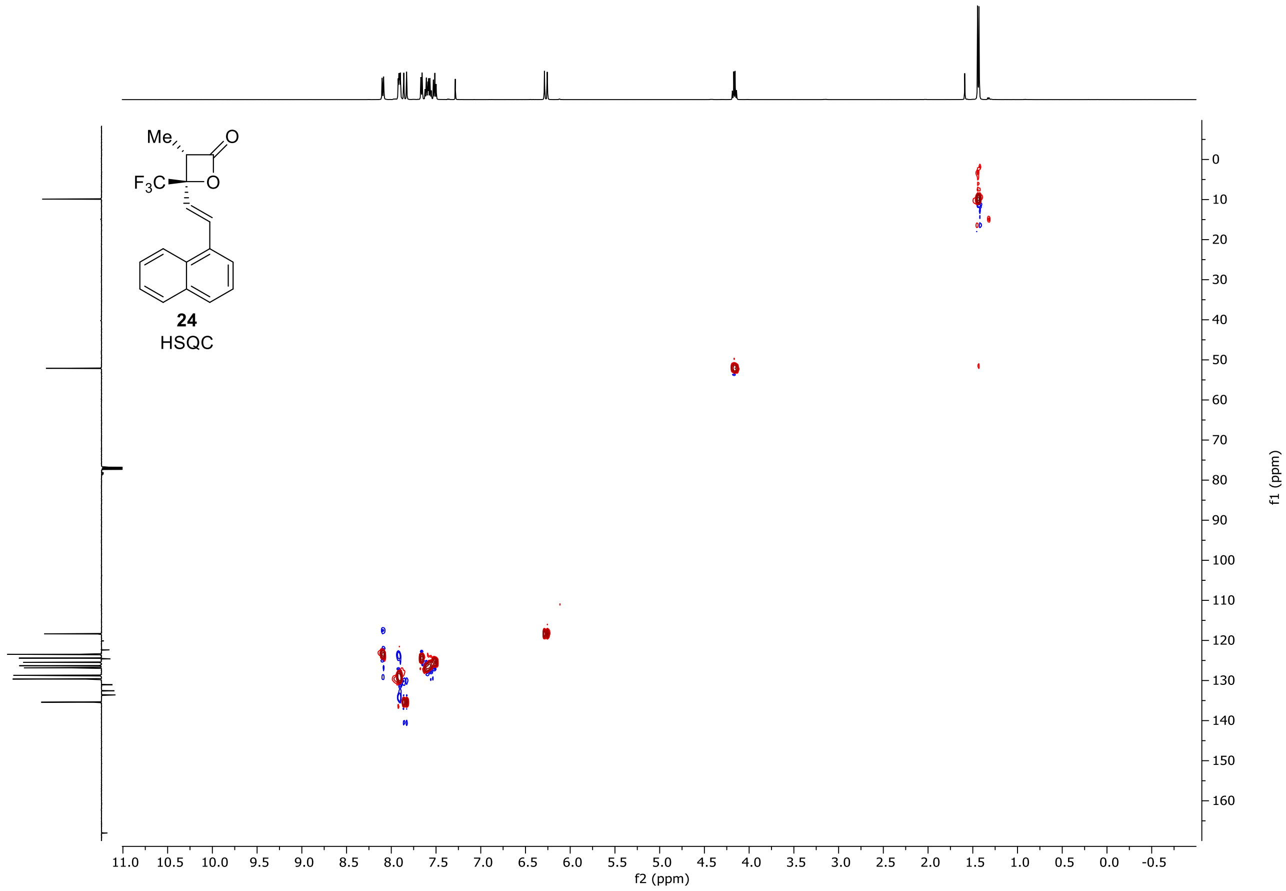

Me
O
F3C
O
24
HSQC
f2 (ppm)
f1 (ppm)

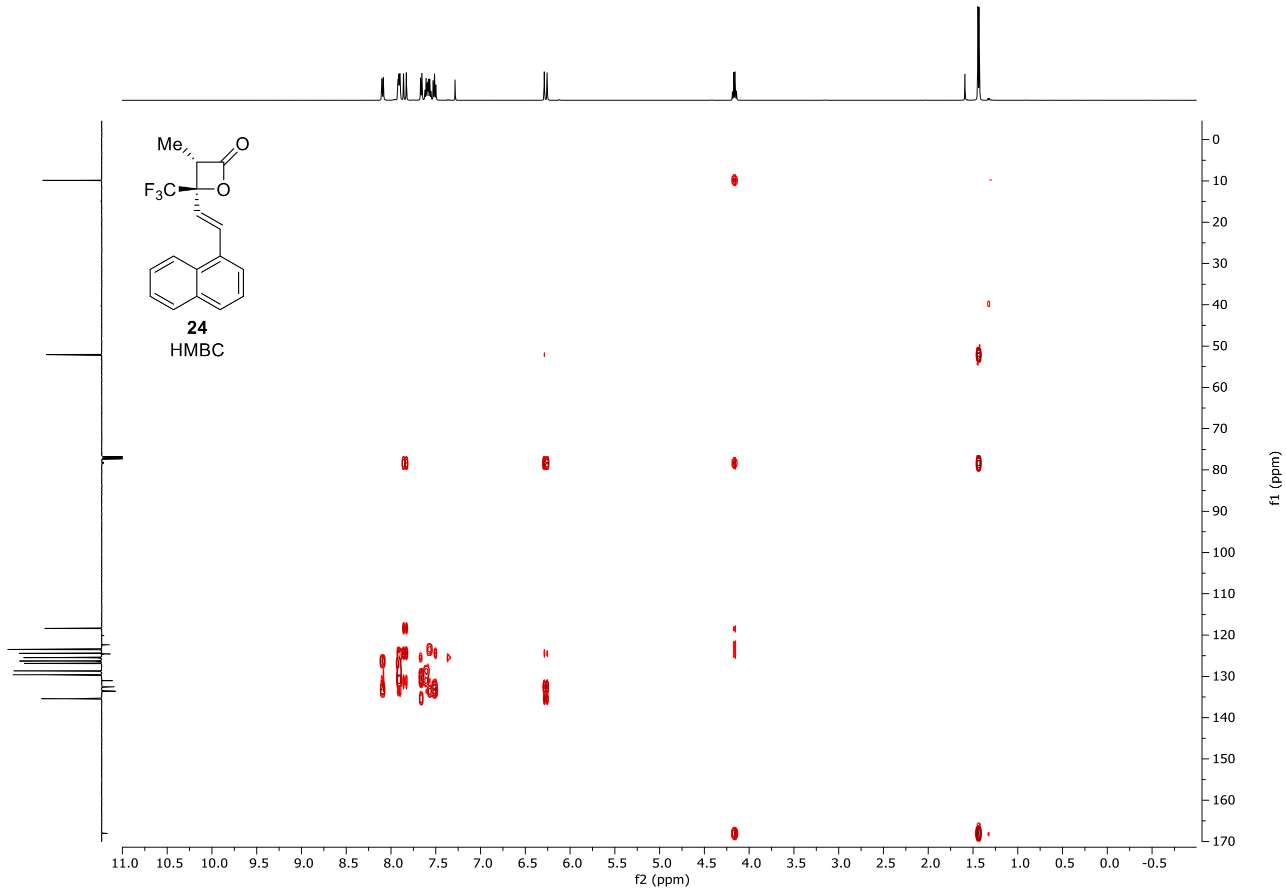

Me
O
F3C
O
24
HMBC
f2 (ppm)
f1 (ppm)

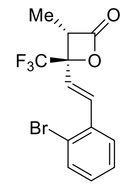

25

^{1}H, $CDCl_3$, 400 MHz

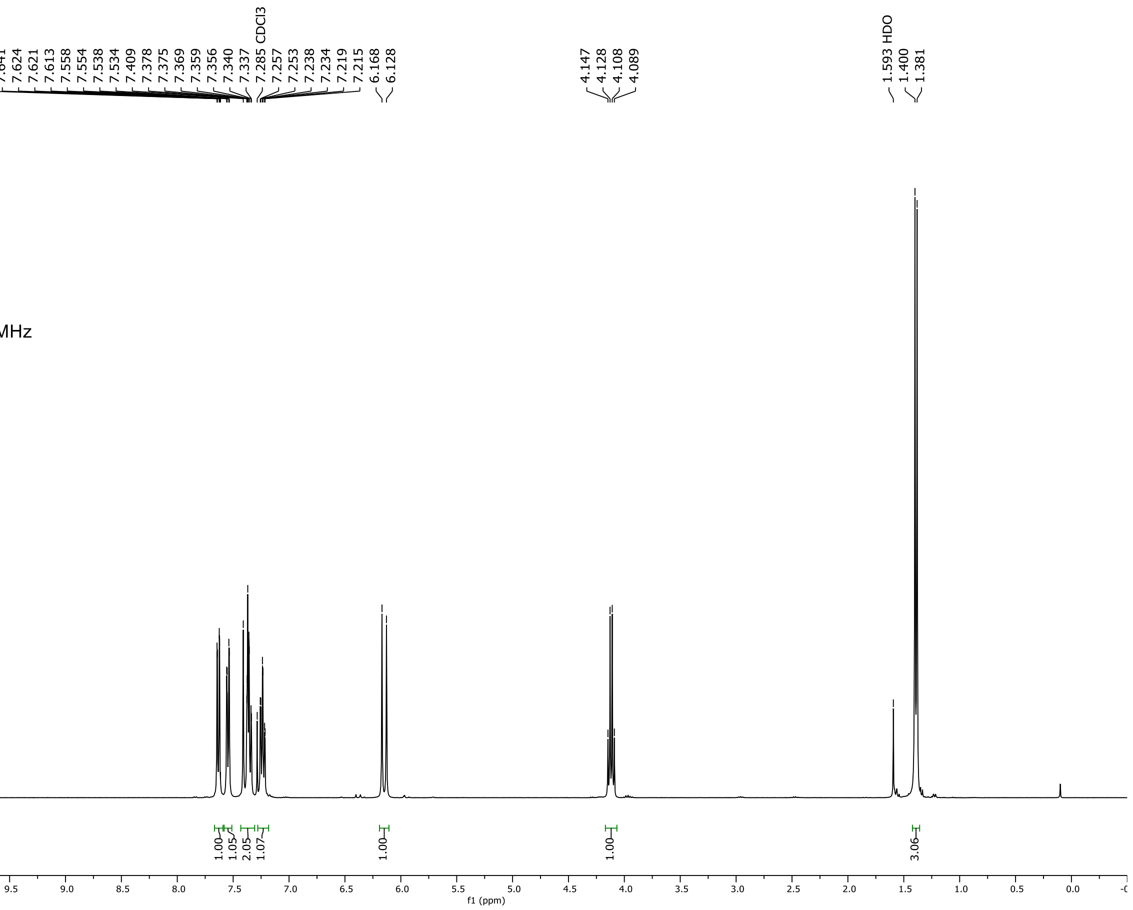

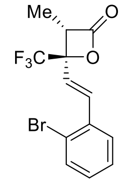

25

^{19}F, $CDCl_3$, 376 MHz

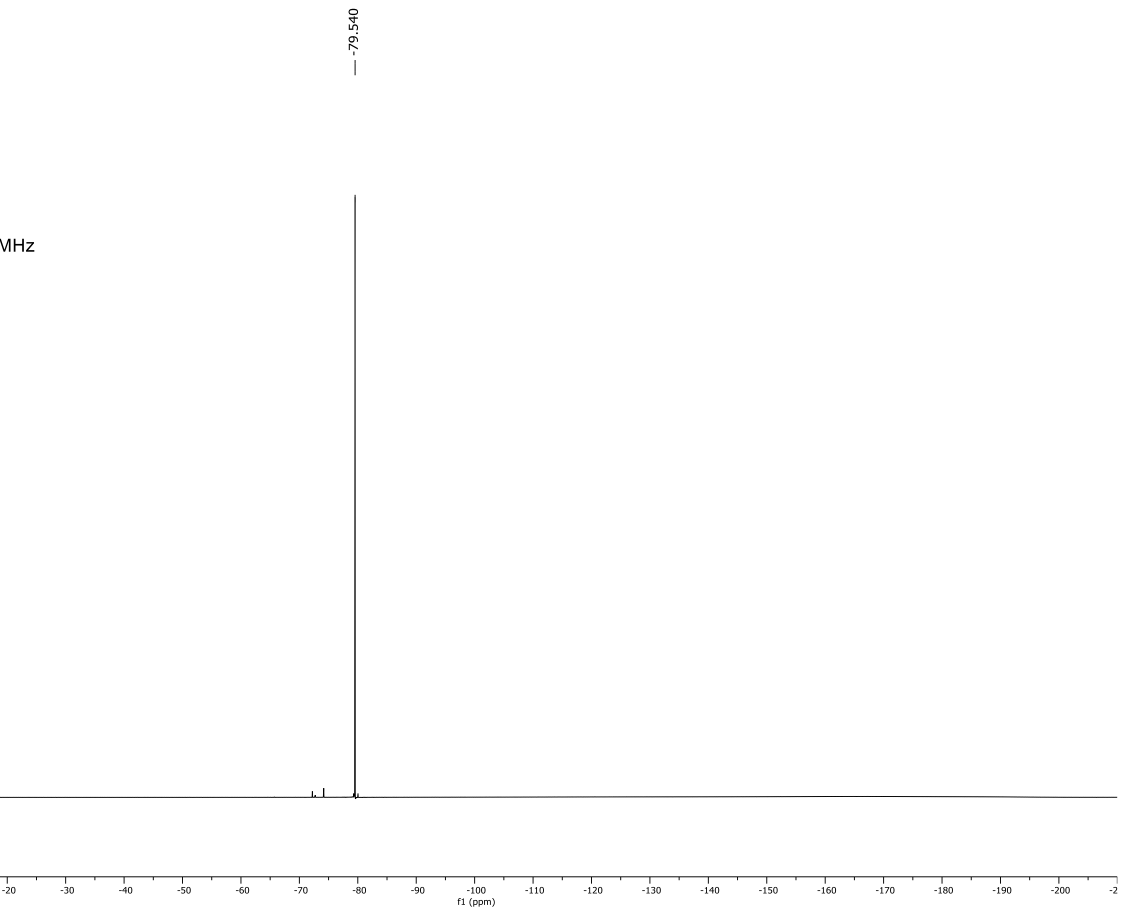

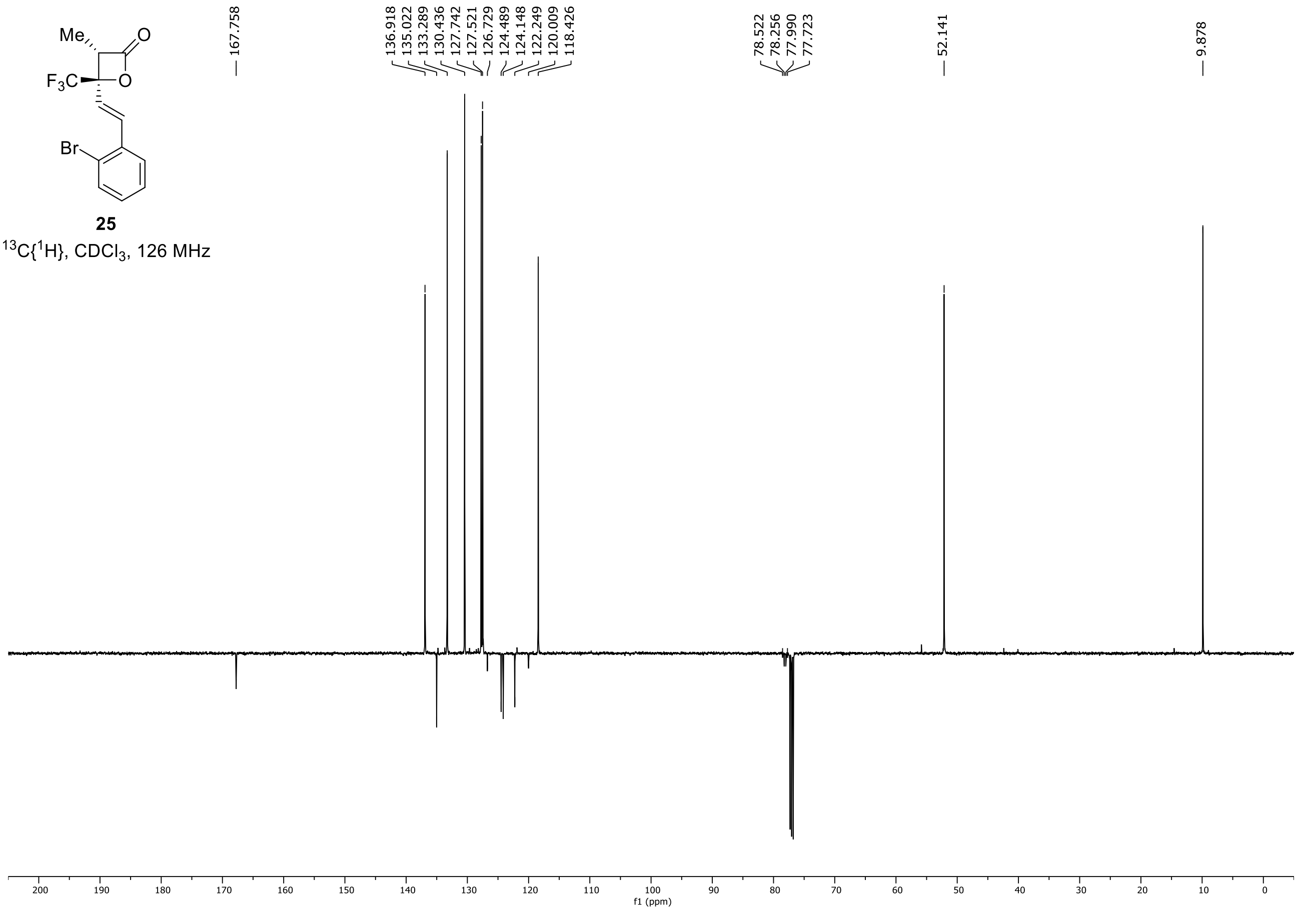
Me
O
F3C
O
Br
25
13C{1H}, CDCl3, 126 MHz
167.758
136.918
135.022
133.289
130.436
127.742
127.521
126.729
124.489
124.148
122.249
120.009
118.426
78.522
78.256
77.990
77.723
52.141
9.878
f1 (ppm)

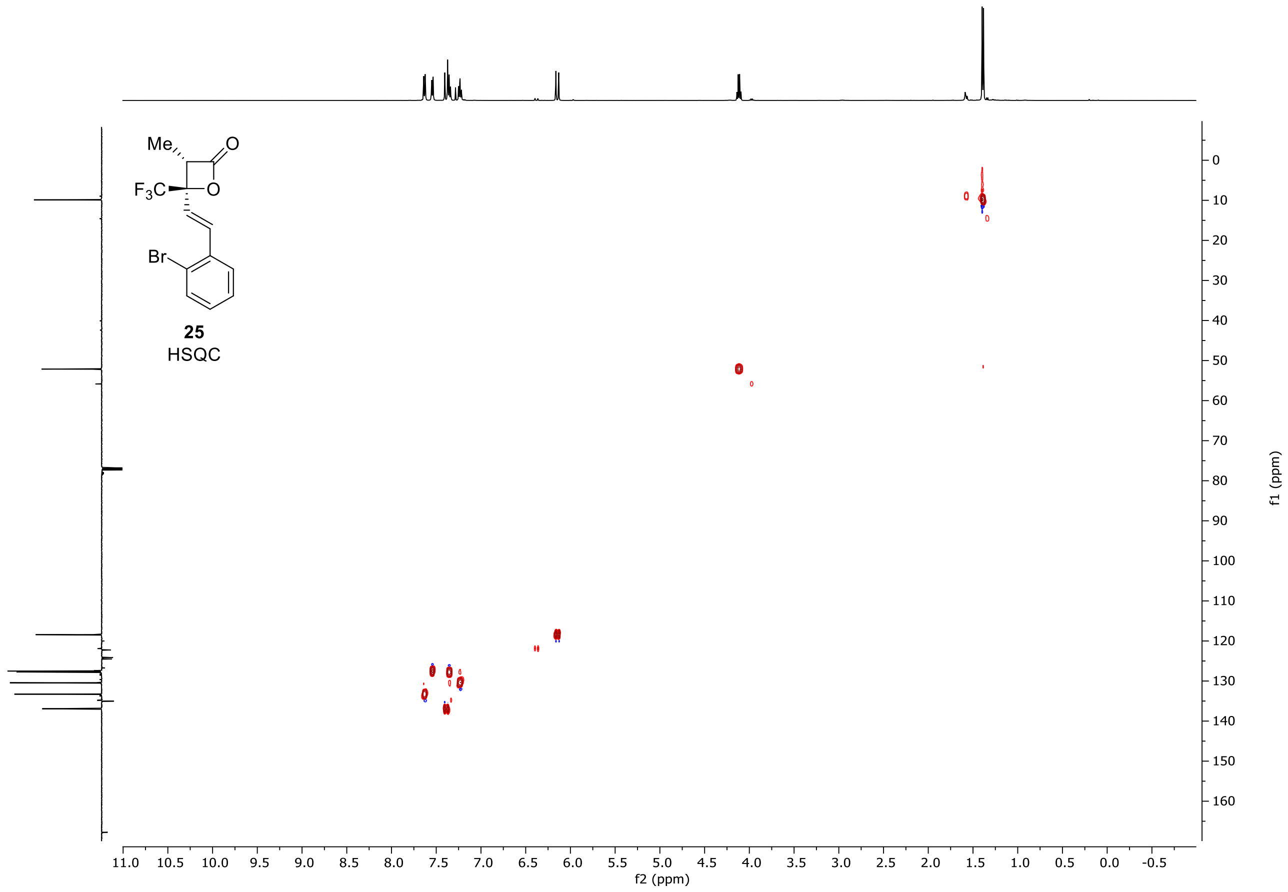
Me
O
F3C
O
Br
25
HSQC
f2 (ppm)
f1 (ppm)

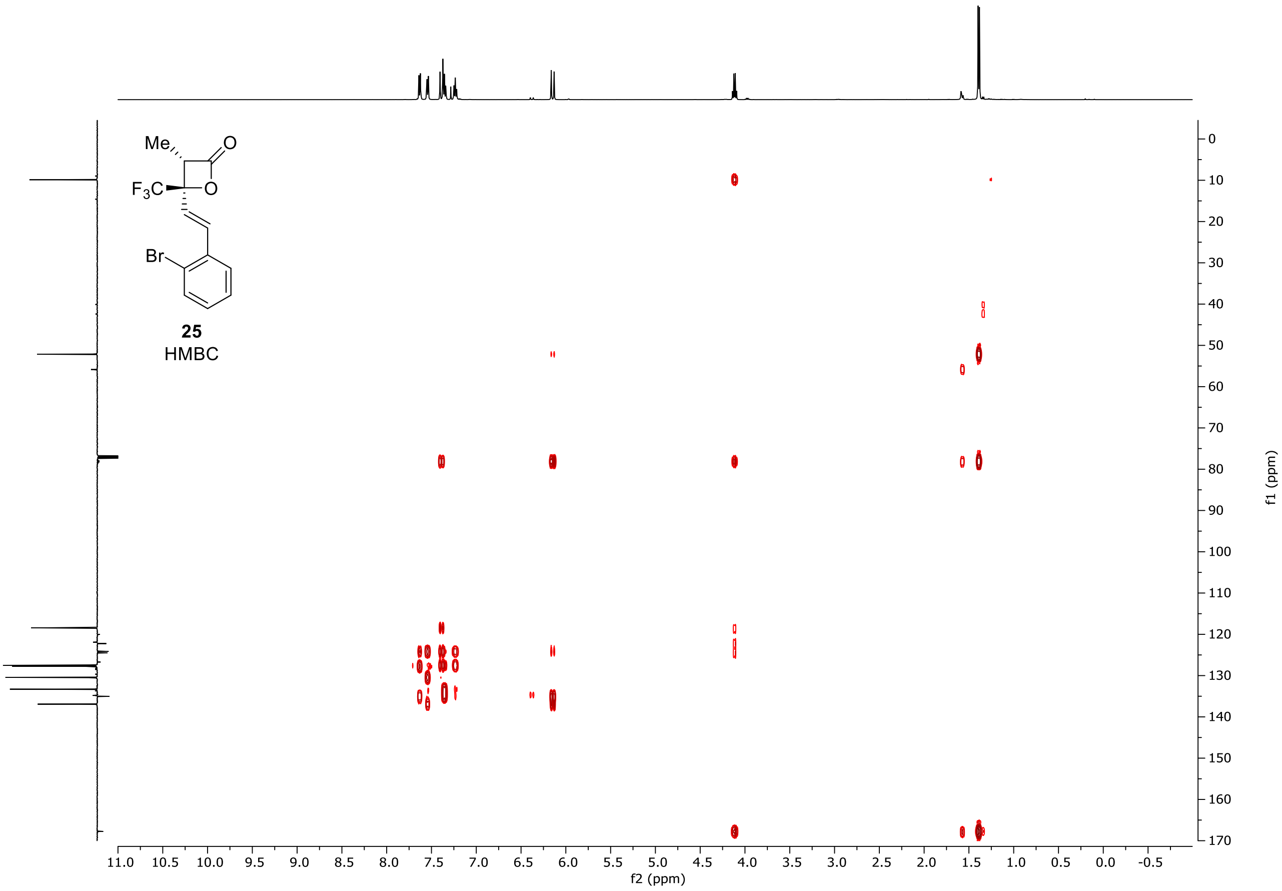
Me
O
F3C
O
Br
25
HMBC
f2 (ppm)
f1 (ppm)

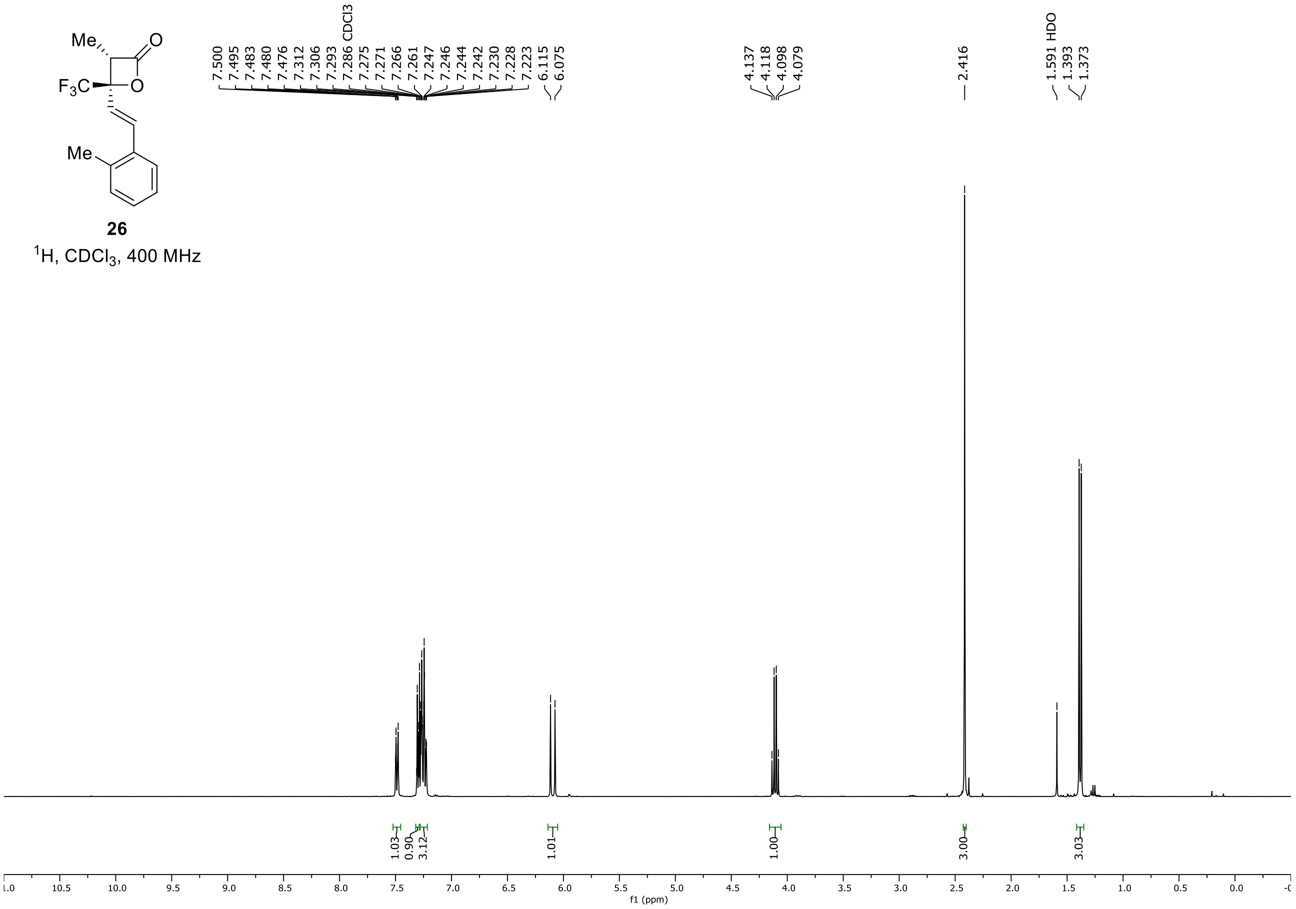
Me
O
F3C
O
Me
26
1H, CDCl3, 400 MHz
7.500
7.495
7.483
7.480
7.476
7.312
7.306
7.293
7.286 CDCl3
7.275
7.271
7.266
7.261
7.247
7.246
7.244
7.242
7.230
7.228
7.223
6.115
6.075
4.137
4.118
4.098
4.079
2.416
1.591 HDO
1.393
1.373
1.03
0.90
3.12
1.01
1.00
3.00
3.03
f1 (ppm)

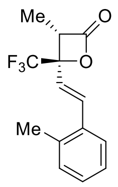

26

^{19}F, $CDCl_3$, 376 MHz

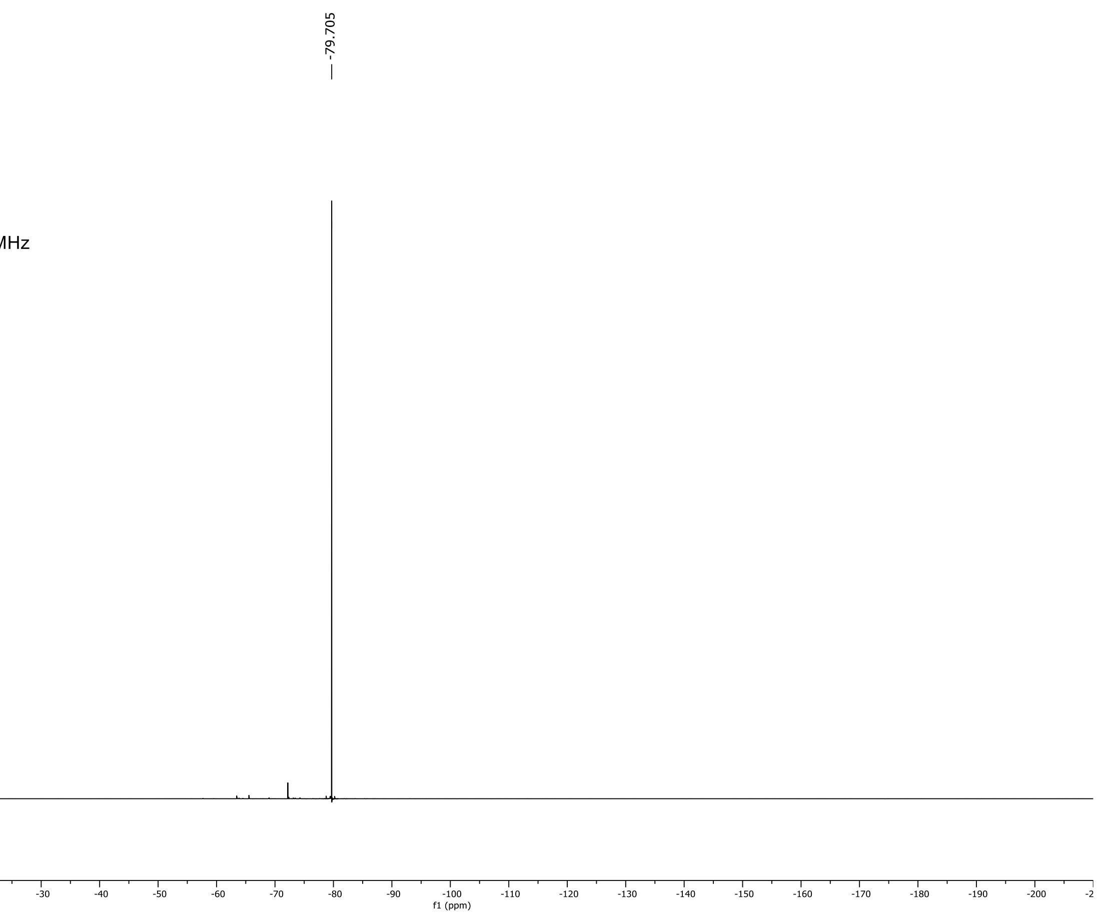

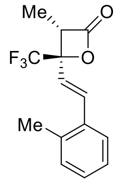

26

$^{13}C\{^1H\}$, $CDCl_3$, 126 MHz

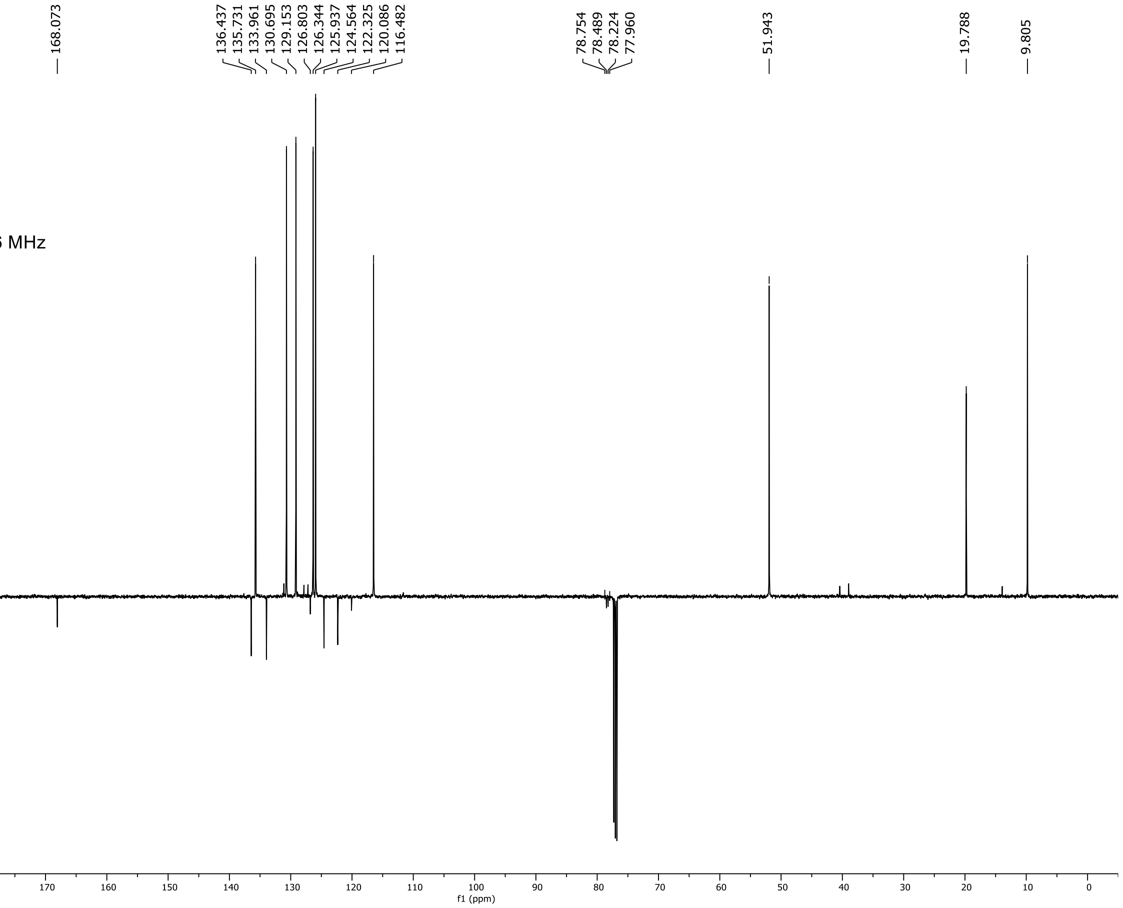

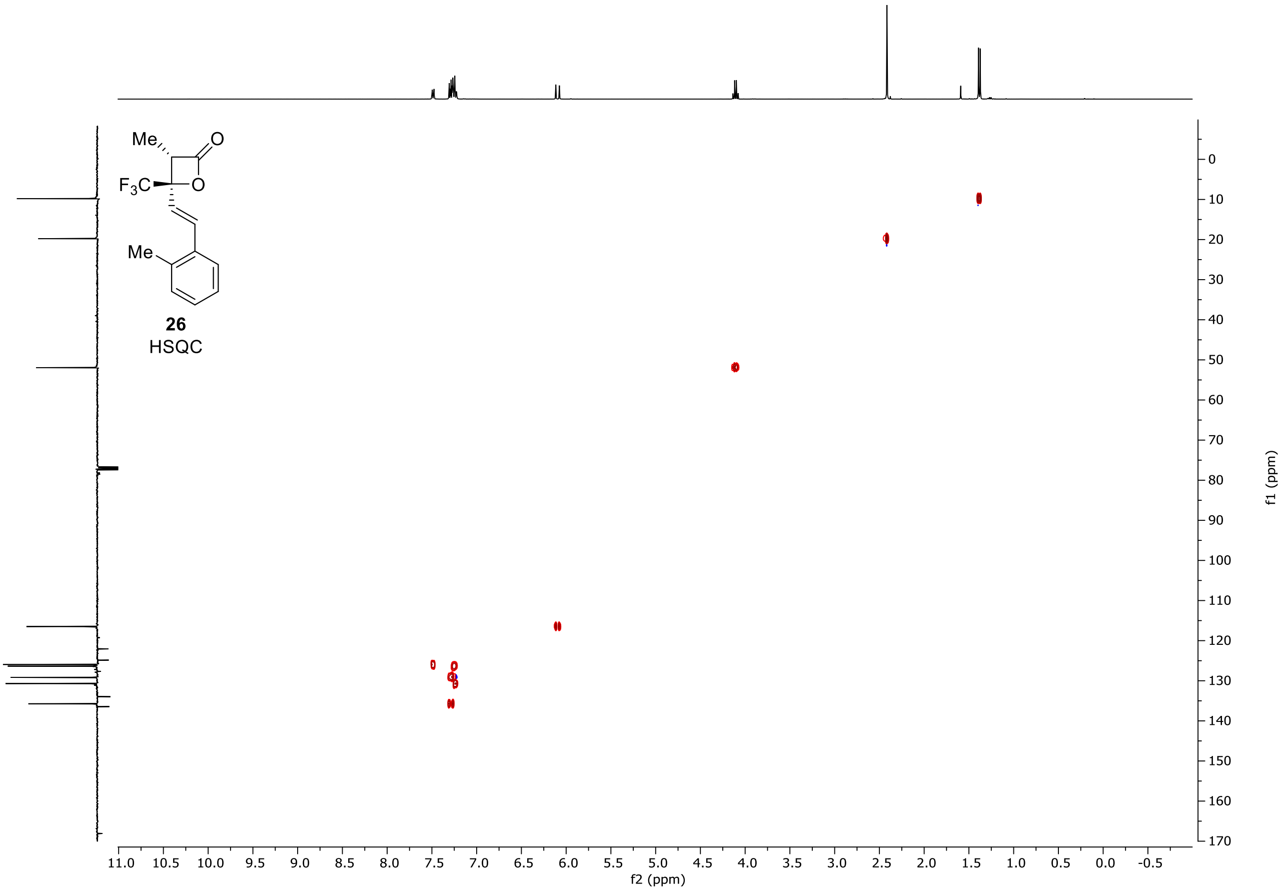
Me
O
F3C
O
Me
26
HSQC
f2 (ppm)
f1 (ppm)

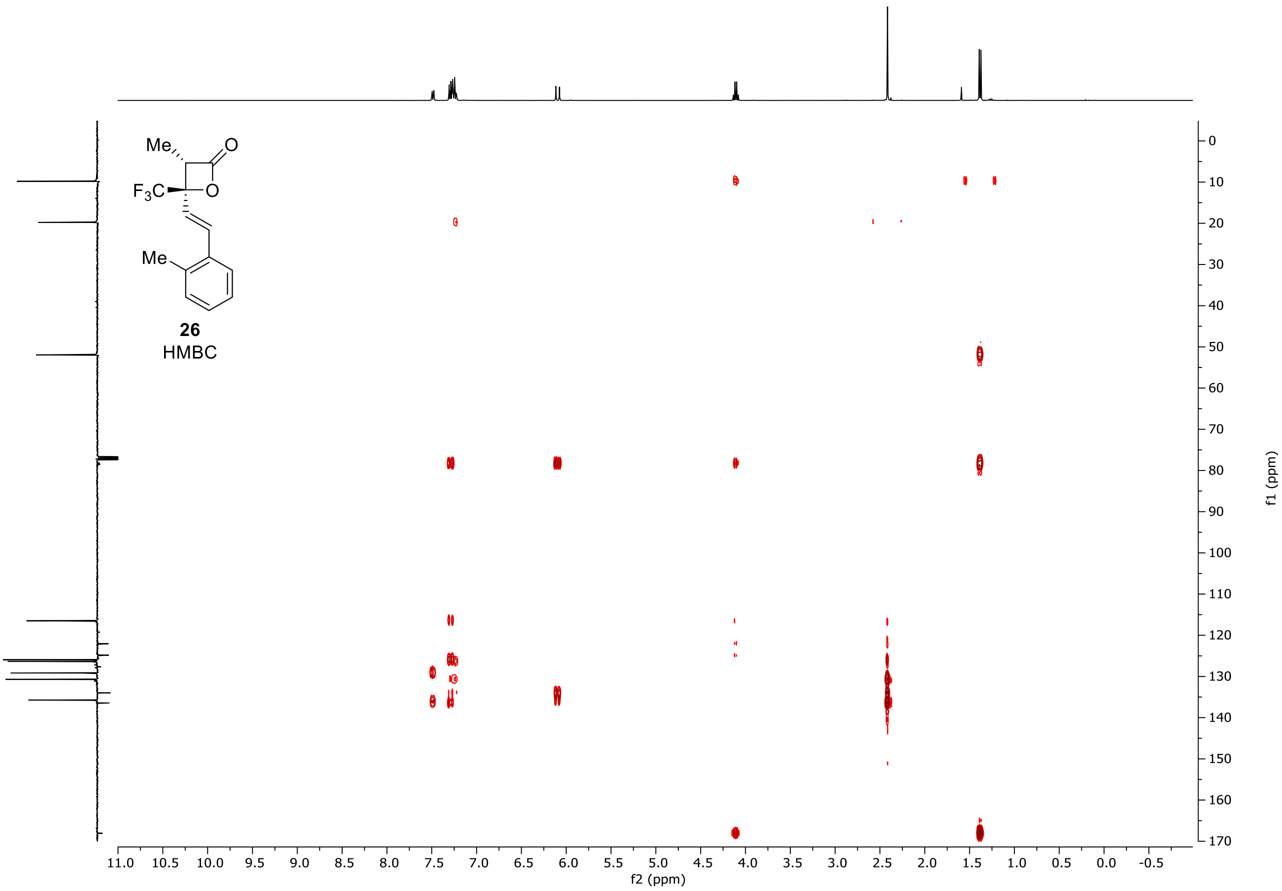
Me
O
F3C
O
Me
26
HMBC
f2 (ppm)
f1 (ppm)

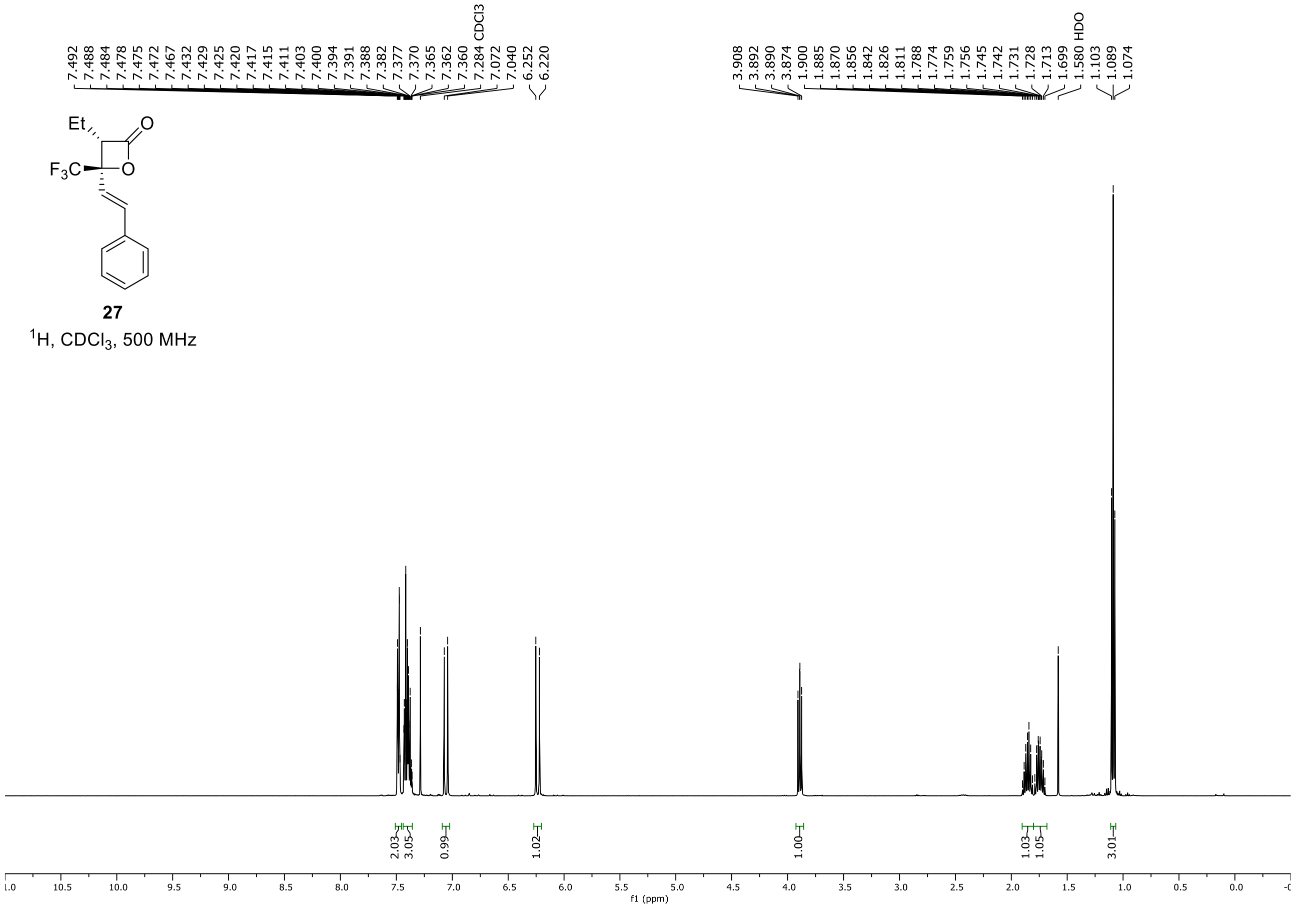
7.492 7.488 7.484 7.478 7.475 7.472 7.467 7.432 7.429 7.425 7.420 7.417 7.415 7.411 7.403 7.400 7.394 7.391 7.388 7.382 7.377 7.370 7.365 7.362 7.360 7.284 CDCl3 7.072 7.040 6.252 6.220
3.908 3.892 3.890 3.874 1.900 1.885 1.870 1.856 1.842 1.826 1.811 1.788 1.774 1.759 1.756 1.745 1.742 1.731 1.728 1.713 1.699 1.580 HDO 1.103 1.089 1.074
Et
O
F3C
O
27
1H, CDCl3, 500 MHz
2.03 3.05 0.99 1.02 1.00 1.03 1.05 3.01
f1 (ppm)

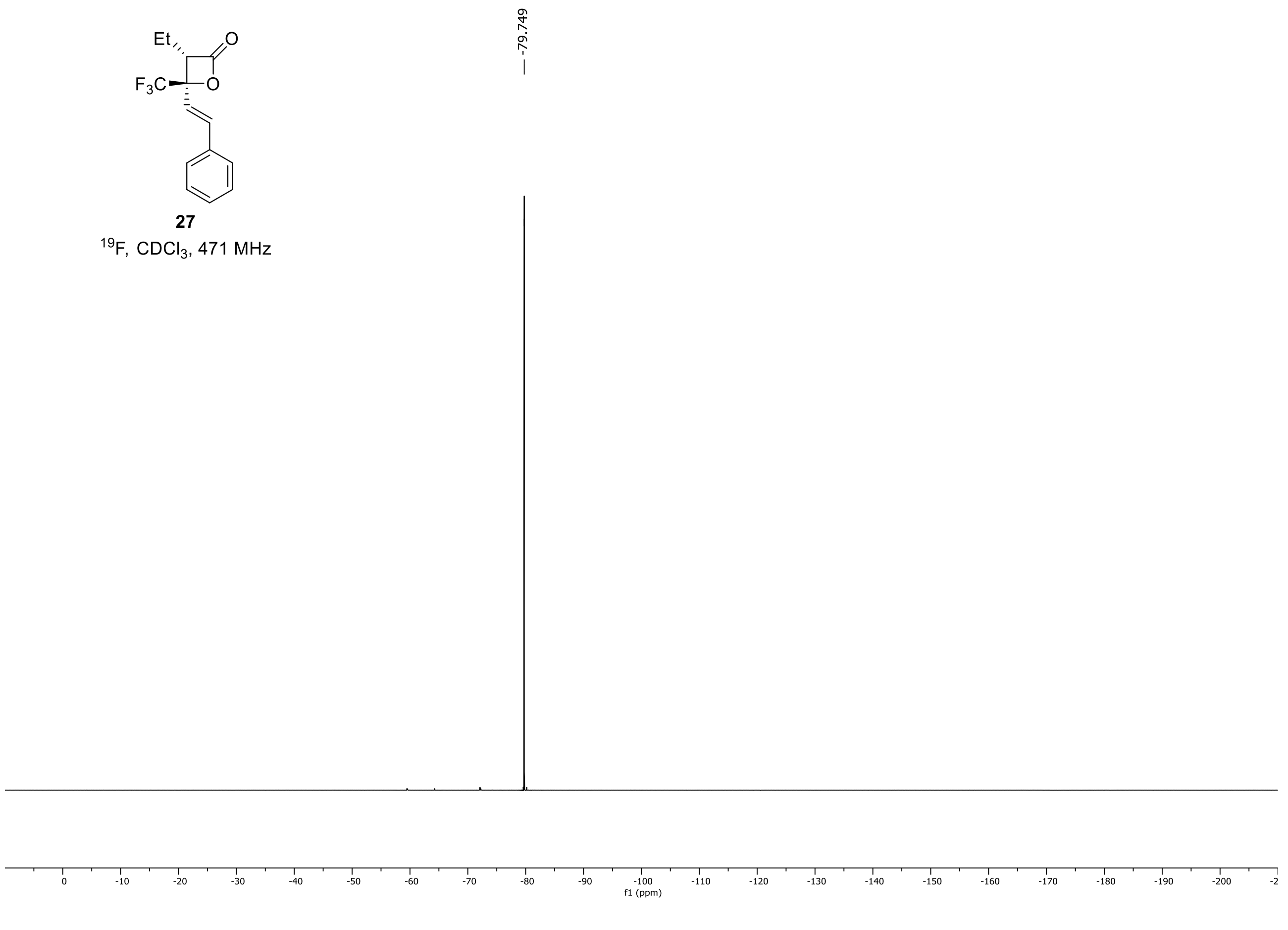
-79.749
Et
O
F3C
O
27
19F, CDCl3, 471 MHz
f1 (ppm)

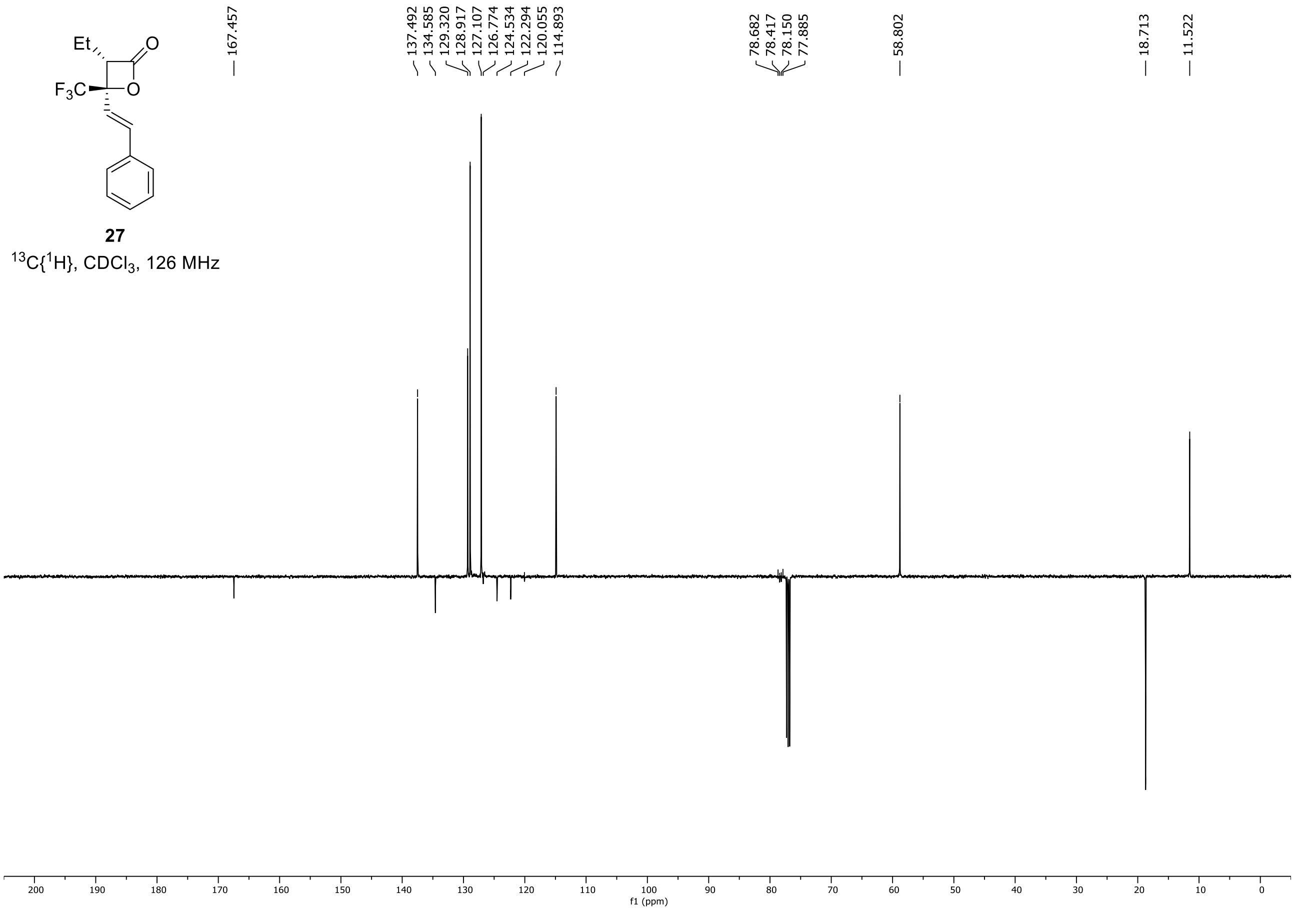
27
$^{13}C\{^1H\}$, $CDCl_3$, 126 MHz
167.457
137.492
134.585
129.320
128.917
127.107
126.774
124.534
122.294
120.055
114.893
78.682
78.417
78.150
77.885
58.802
18.713
11.522
f1 (ppm)

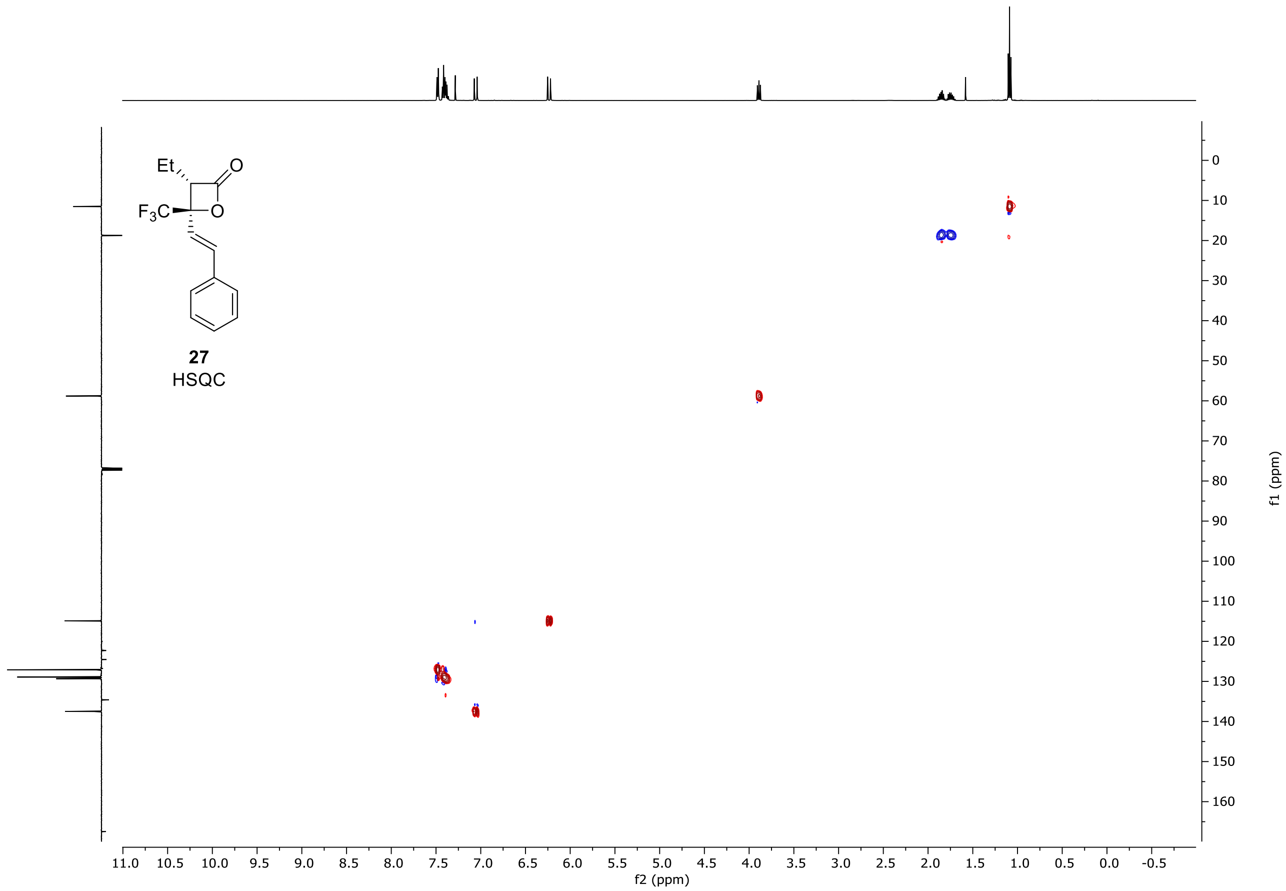
27
HSQC
f2 (ppm)
f1 (ppm)

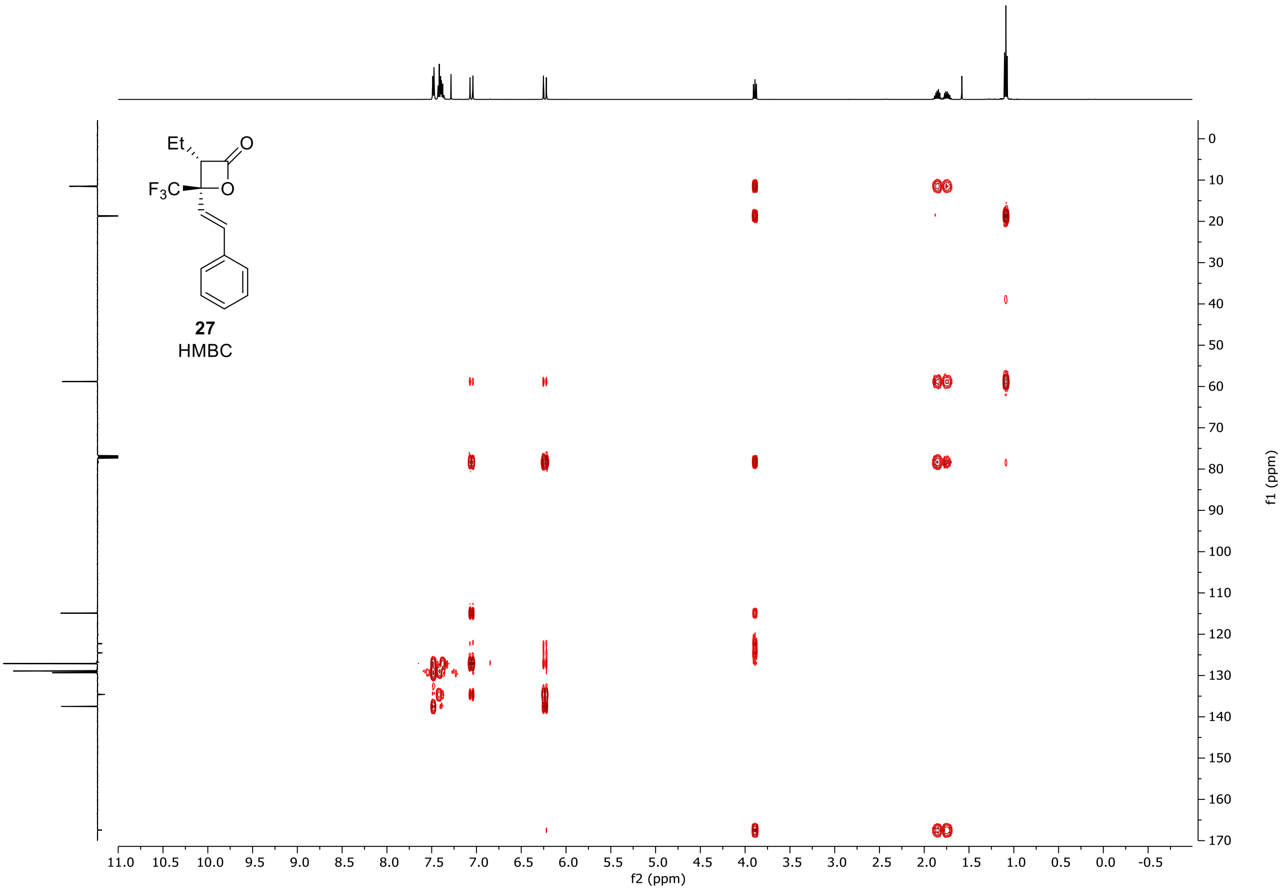
Et
O
F_3C
O
27
HMBC
f1 (ppm)
f2 (ppm)

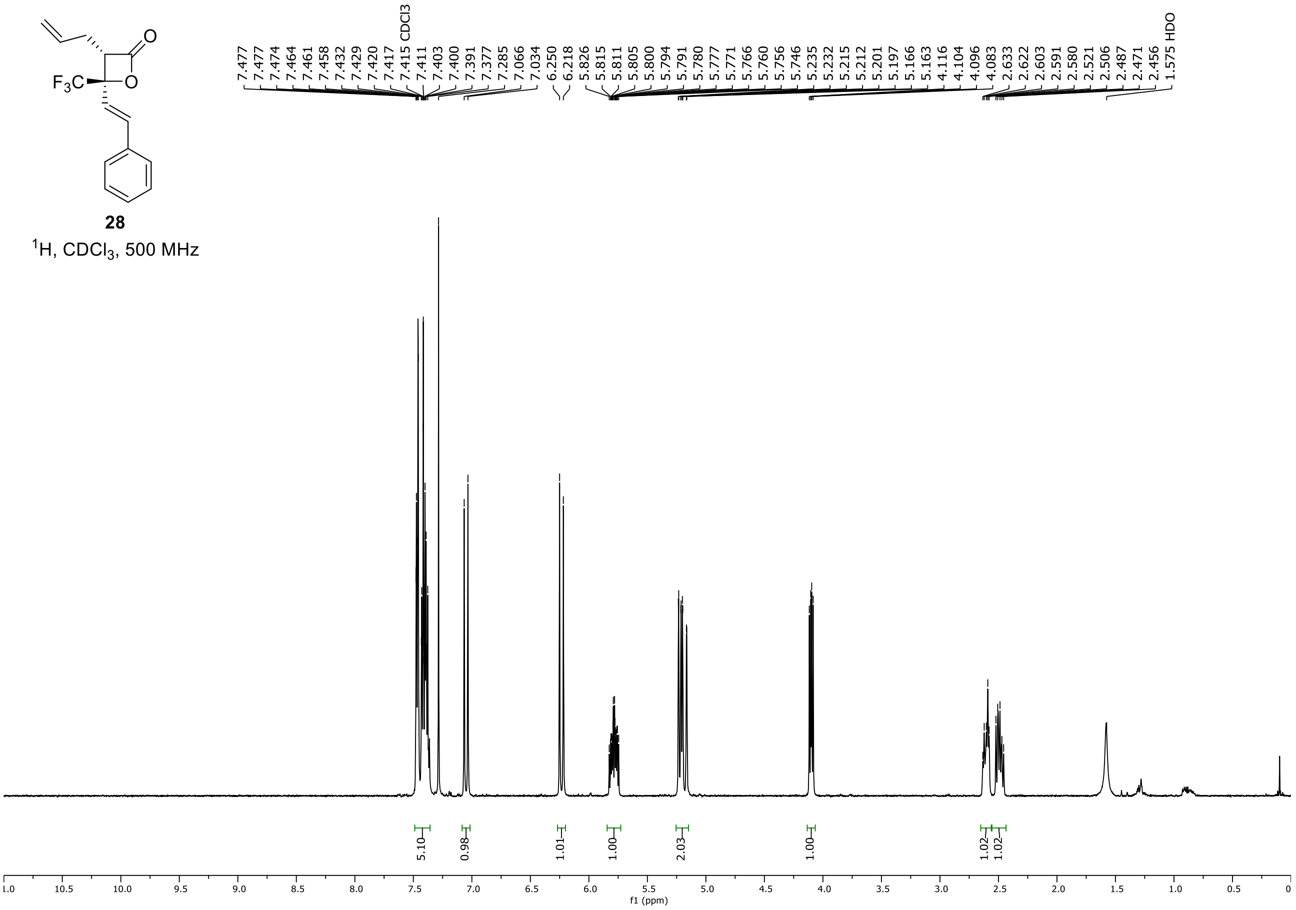
O
F_3C
O
28
^{1}H, $CDCl_3$, 500 MHz
7.477
7.477
7.474
7.464
7.461
7.458
7.432
7.429
7.420
7.417
7.415 CDCl3
7.411
7.403
7.400
7.391
7.377
7.285
7.066
7.034
6.250
6.218
5.826
5.815
5.811
5.805
5.800
5.794
5.791
5.780
5.777
5.771
5.766
5.760
5.756
5.746
5.235
5.232
5.215
5.212
5.201
5.197
5.166
5.163
4.116
4.104
4.096
4.083
2.633
2.622
2.603
2.591
2.580
2.521
2.506
2.487
2.471
2.456
1.575 HDO
5.10
0.98
1.01
1.00
2.03
1.00
1.02
1.02
f1 (ppm)

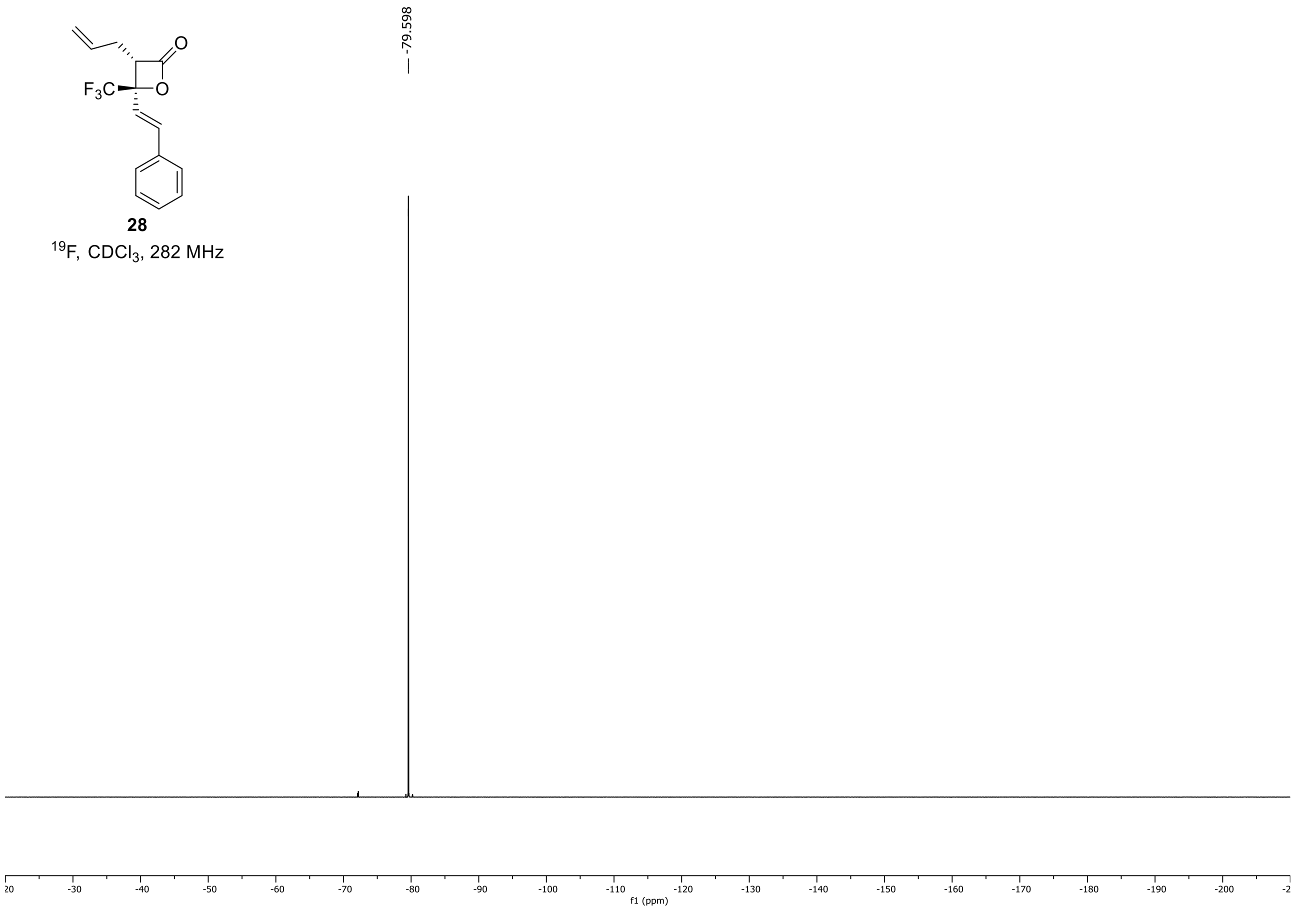

28
^{19}F, $CDCl_3$, 282 MHz

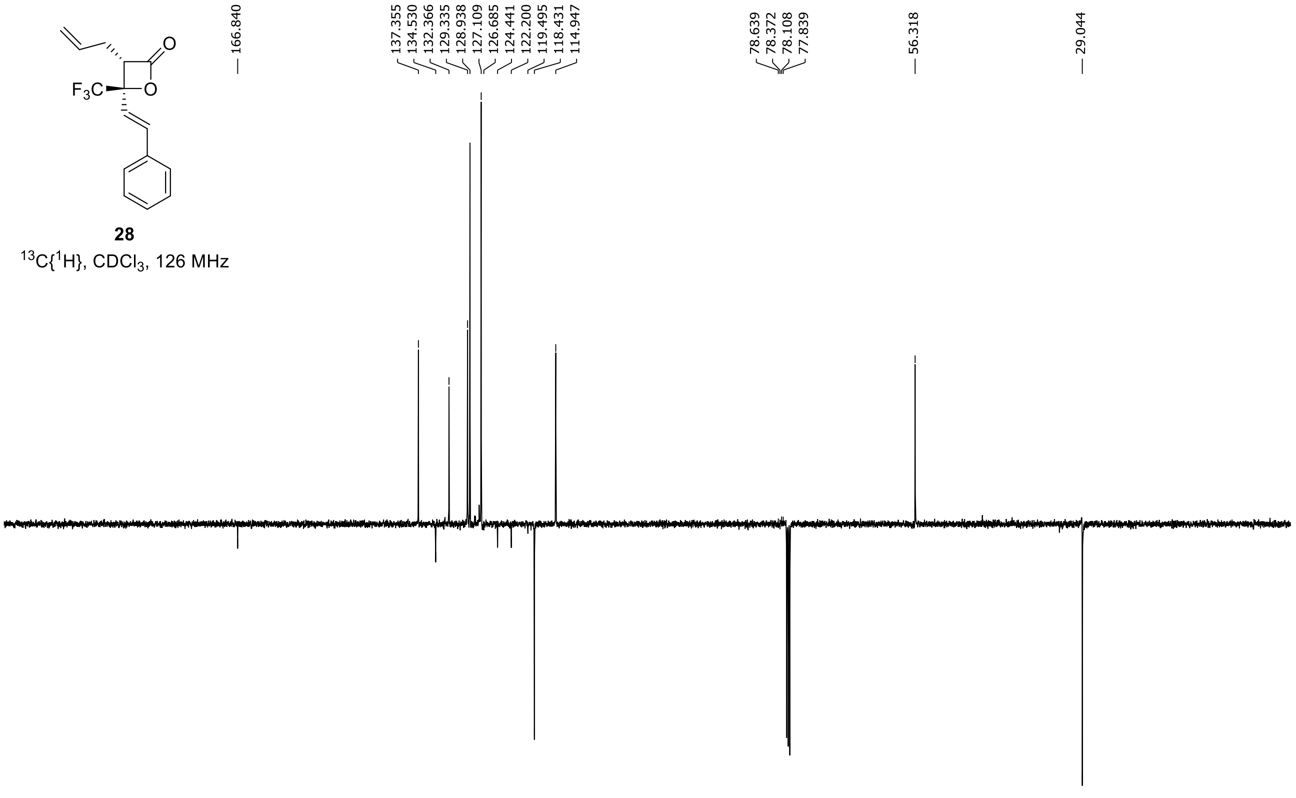

28
$^{13}C\{^1H\}$, $CDCl_3$, 126 MHz

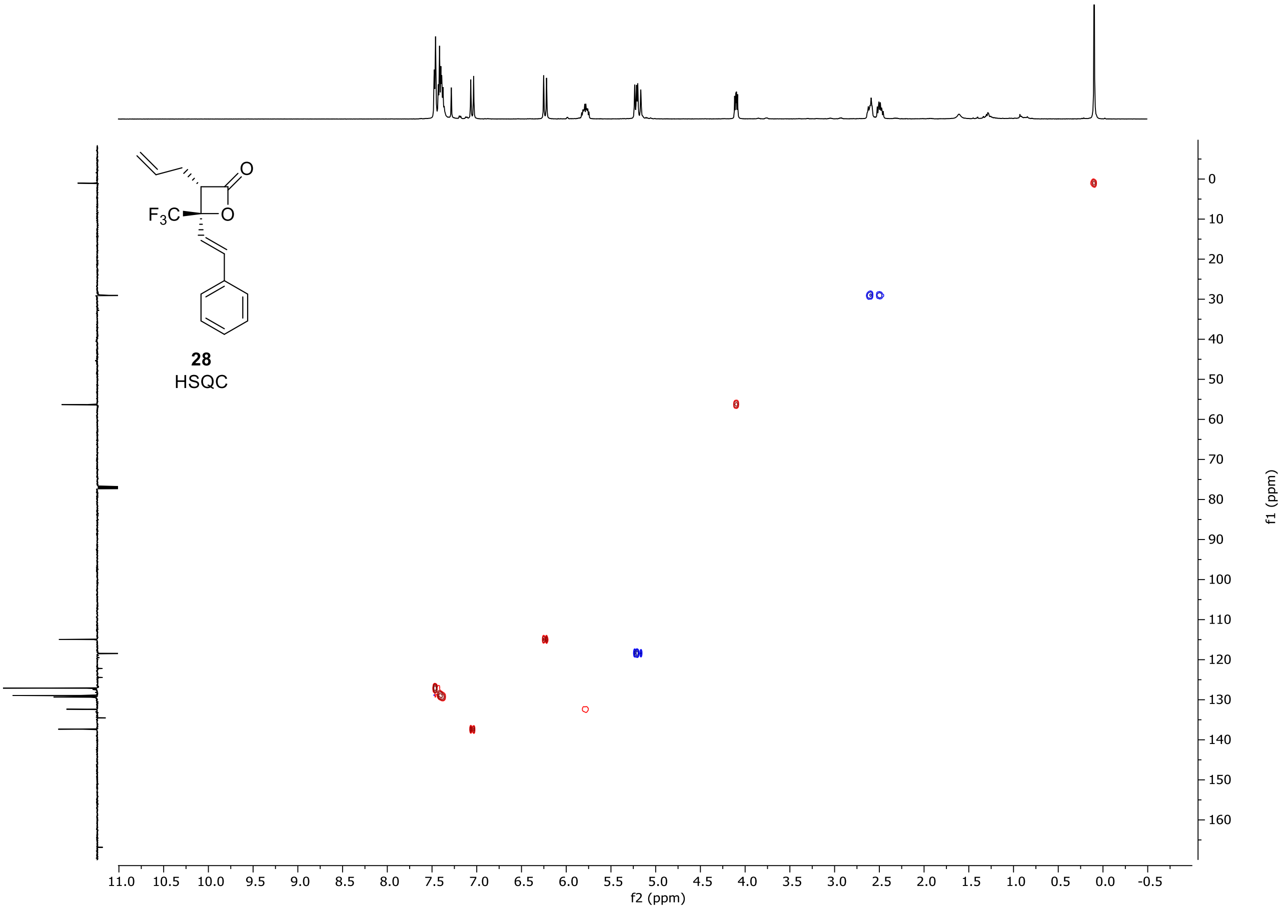

F3C
O
O
28
HSQC
f2 (ppm)
f1 (ppm)

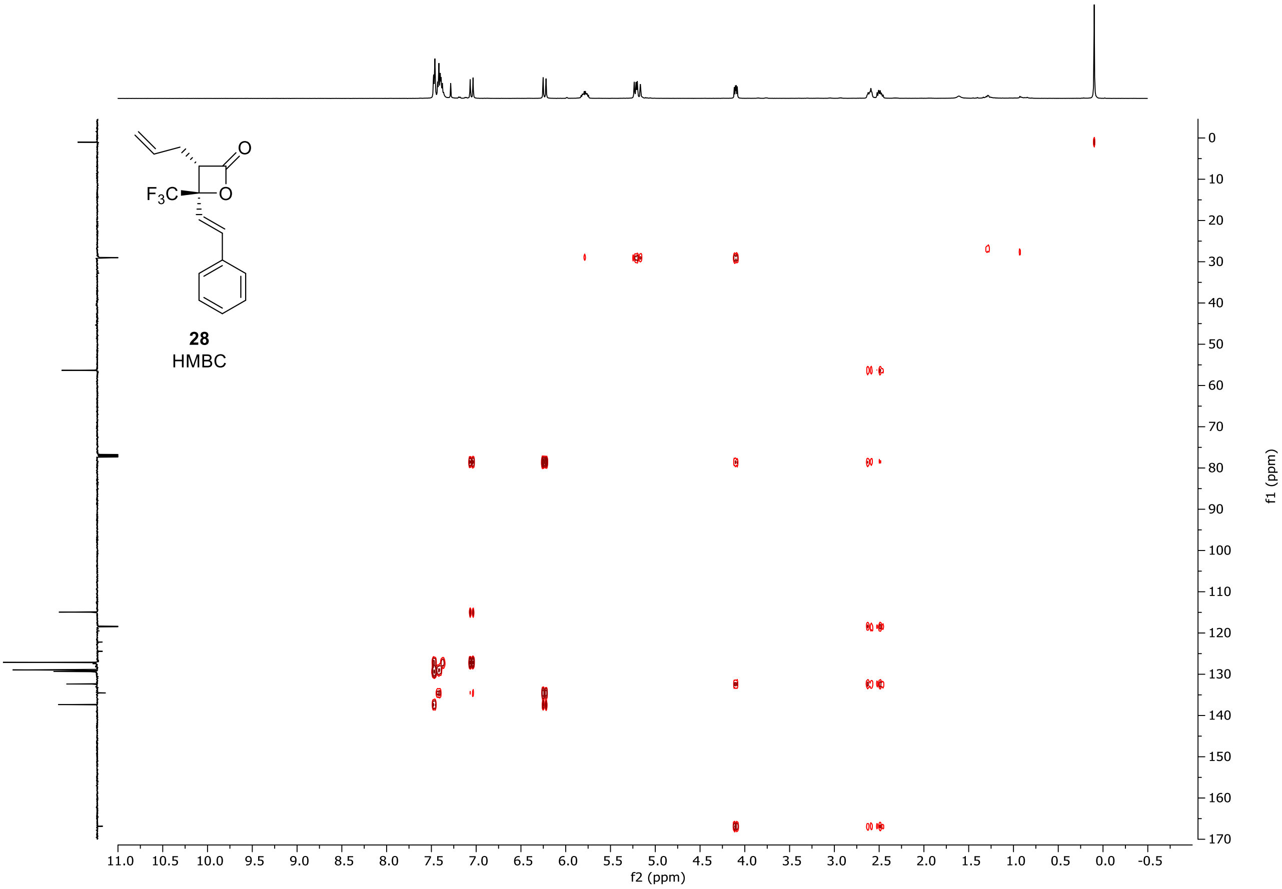

F3C
O
O
28
HMBC
f2 (ppm)
f1 (ppm)

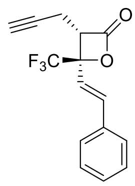

29

^{1}H, $CDCl_3$, 500 MHz

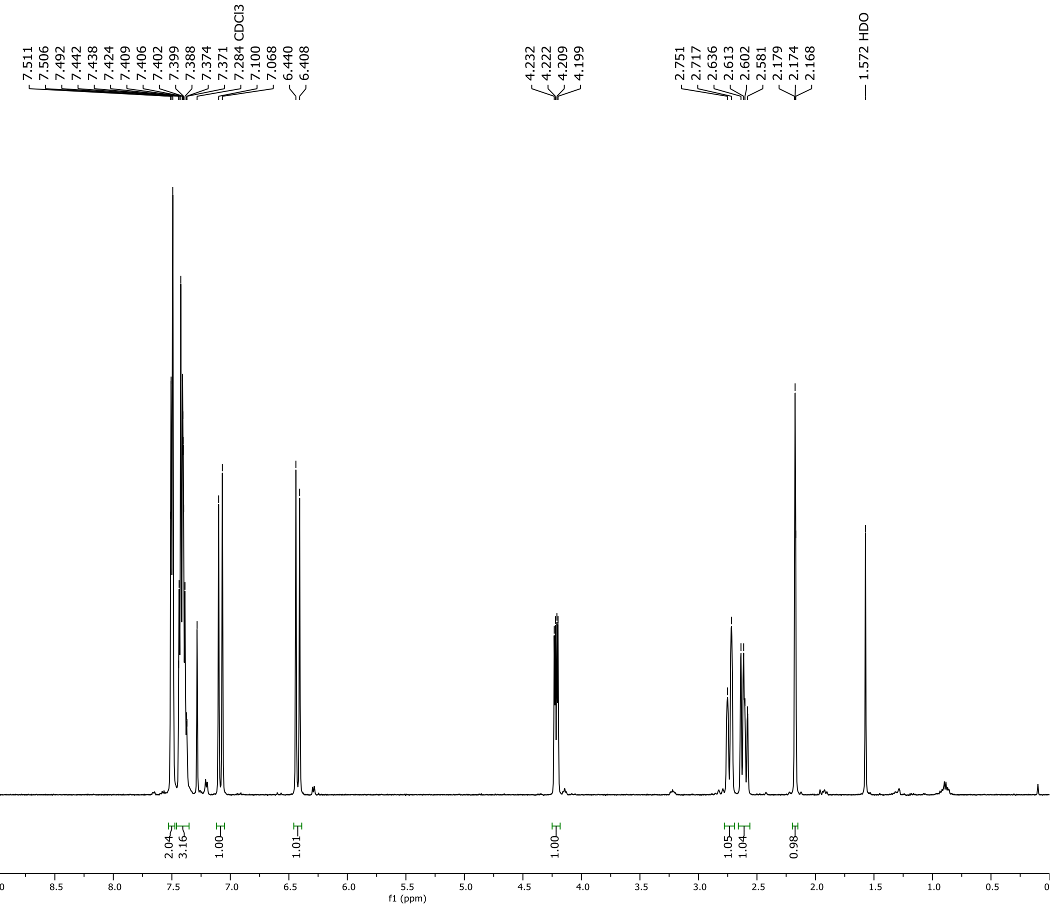

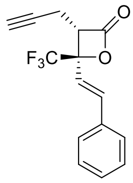

29

^{19}F, $CDCl_3$, 282 MHz

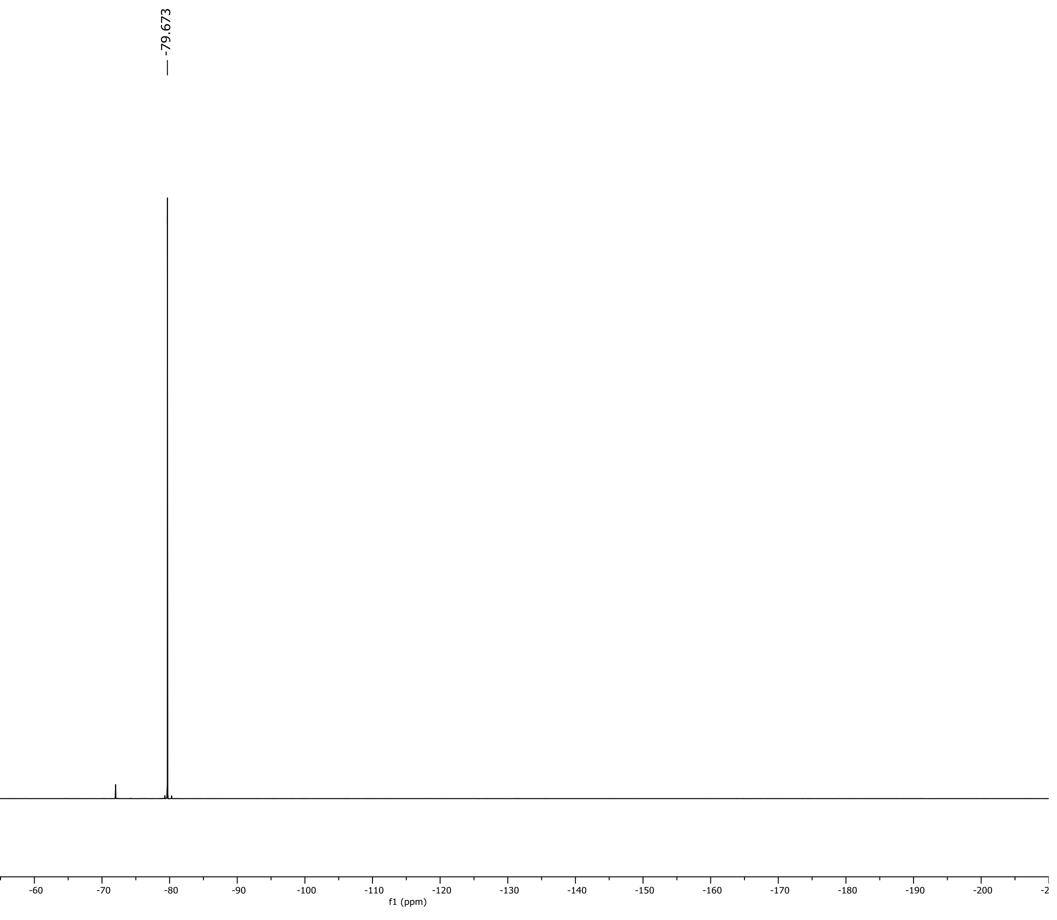

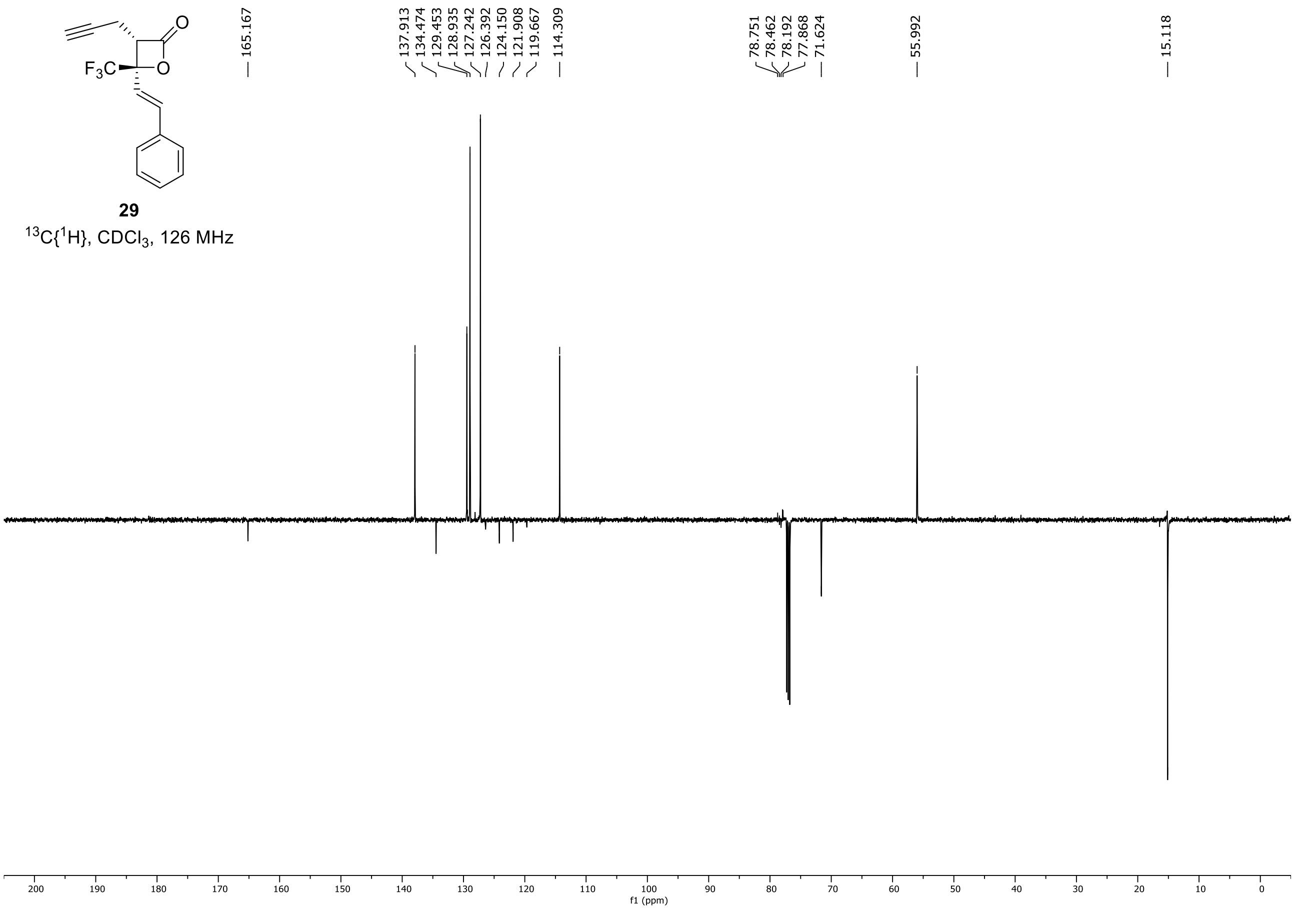
F3C
O
O
29
13C{1H}, CDCl3, 126 MHz
165.167
137.913
134.474
129.453
128.935
127.242
126.392
124.150
121.908
119.667
114.309
78.751
78.462
78.192
77.868
71.624
55.992
15.118
f1 (ppm)

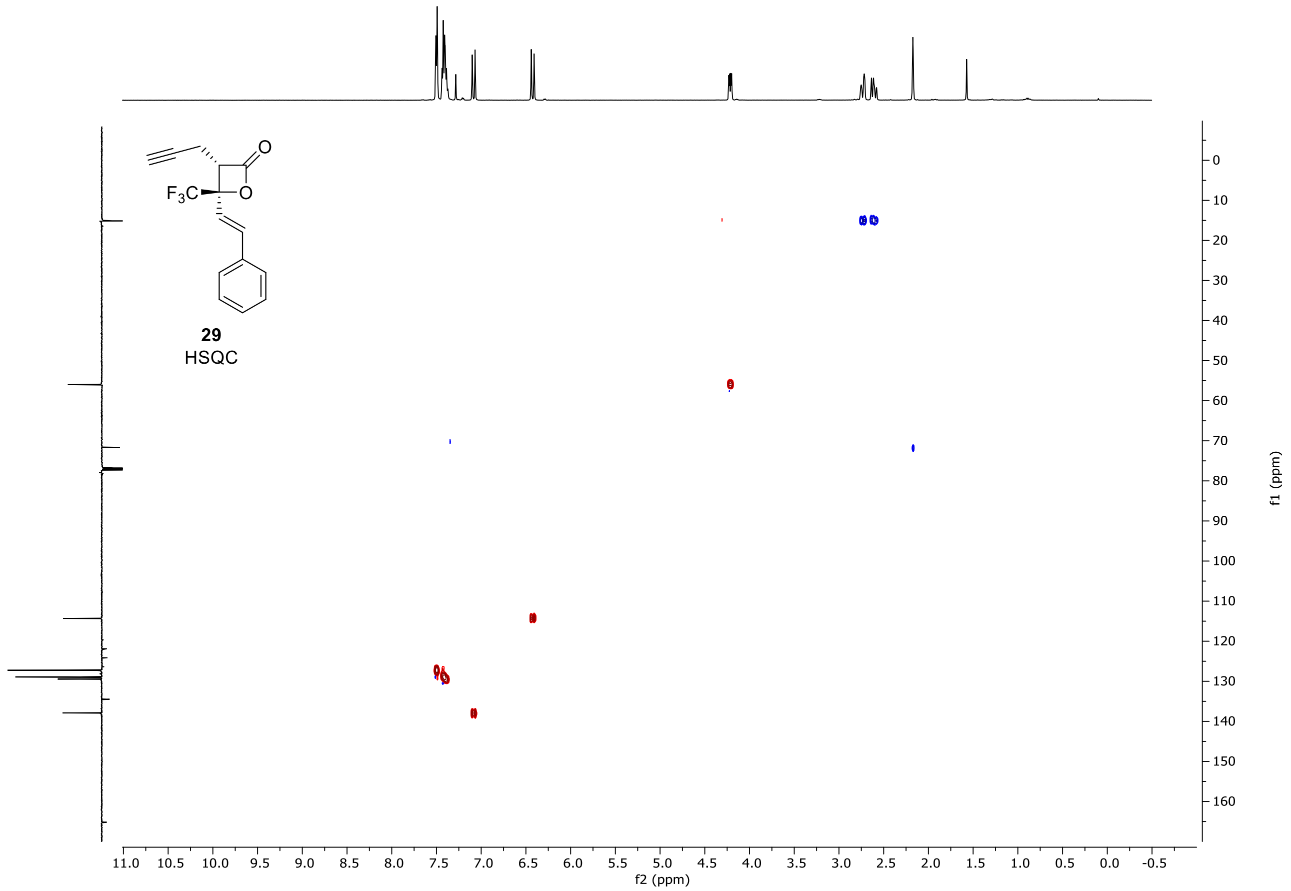
F3C
O
O
29
HSQC
f2 (ppm)
f1 (ppm)

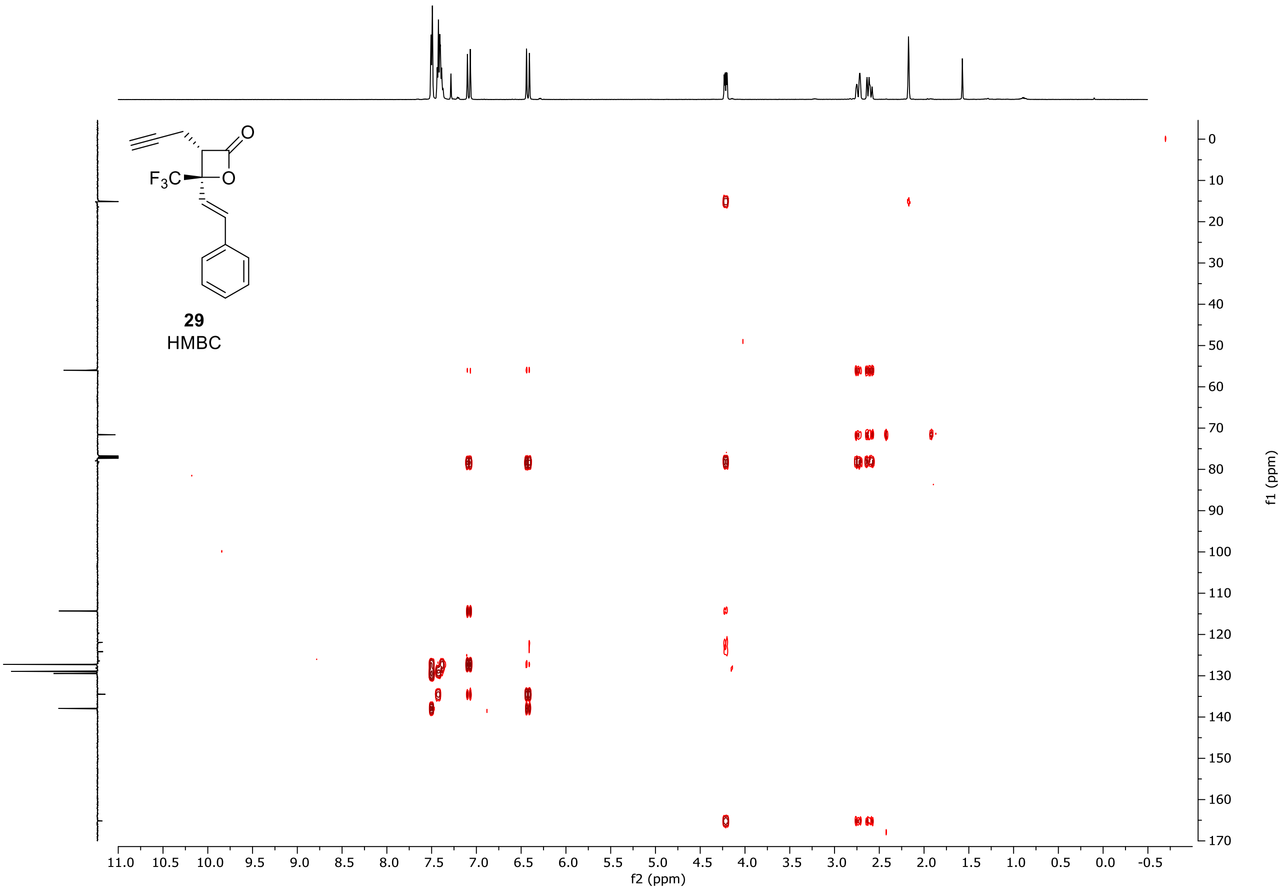

F3C
O
O
29
HMBC
f2 (ppm)
f1 (ppm)

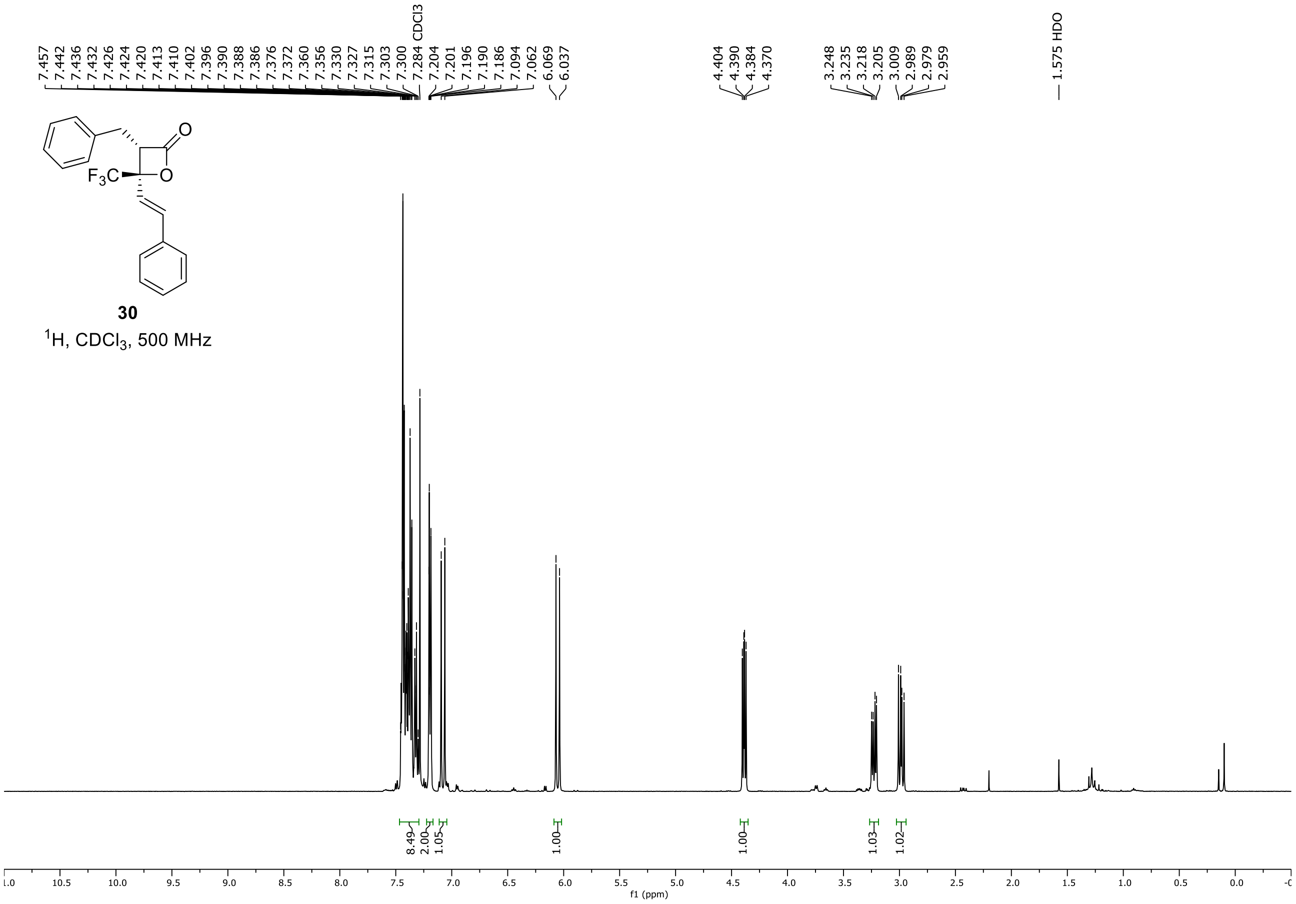

7.457 7.442 7.436 7.432 7.426 7.424 7.420 7.413 7.410 7.402 7.396 7.390 7.388 7.386 7.376 7.372 7.360 7.356 7.330 7.327 7.315 7.303 7.300 7.284 CDCl3 7.204 7.201 7.196 7.190 7.186 7.094 7.062 6.069 6.037
4.404 4.390 4.384 4.370
3.248 3.235 3.218 3.205 3.009 2.989 2.979 2.959
1.575 HDO
F3C
O
O
30
1H, CDCl3, 500 MHz
8.49 2.00 1.05 1.00 1.00 1.03 1.02
f1 (ppm)

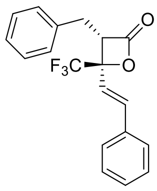

30

^{19}F, $CDCl_3$, 376 MHz

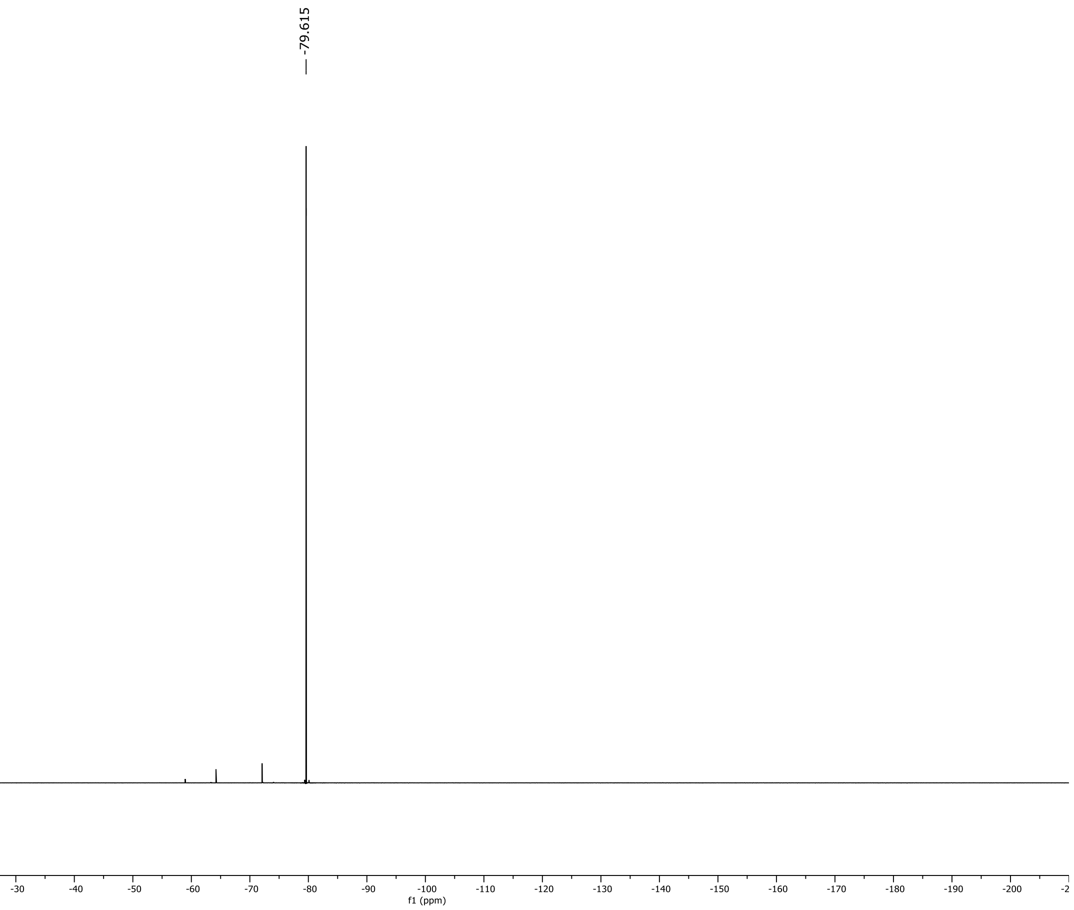

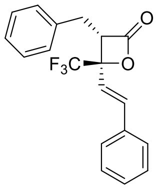

30

$^{13}C\{^1H\}$, $CDCl_3$, 126 MHz

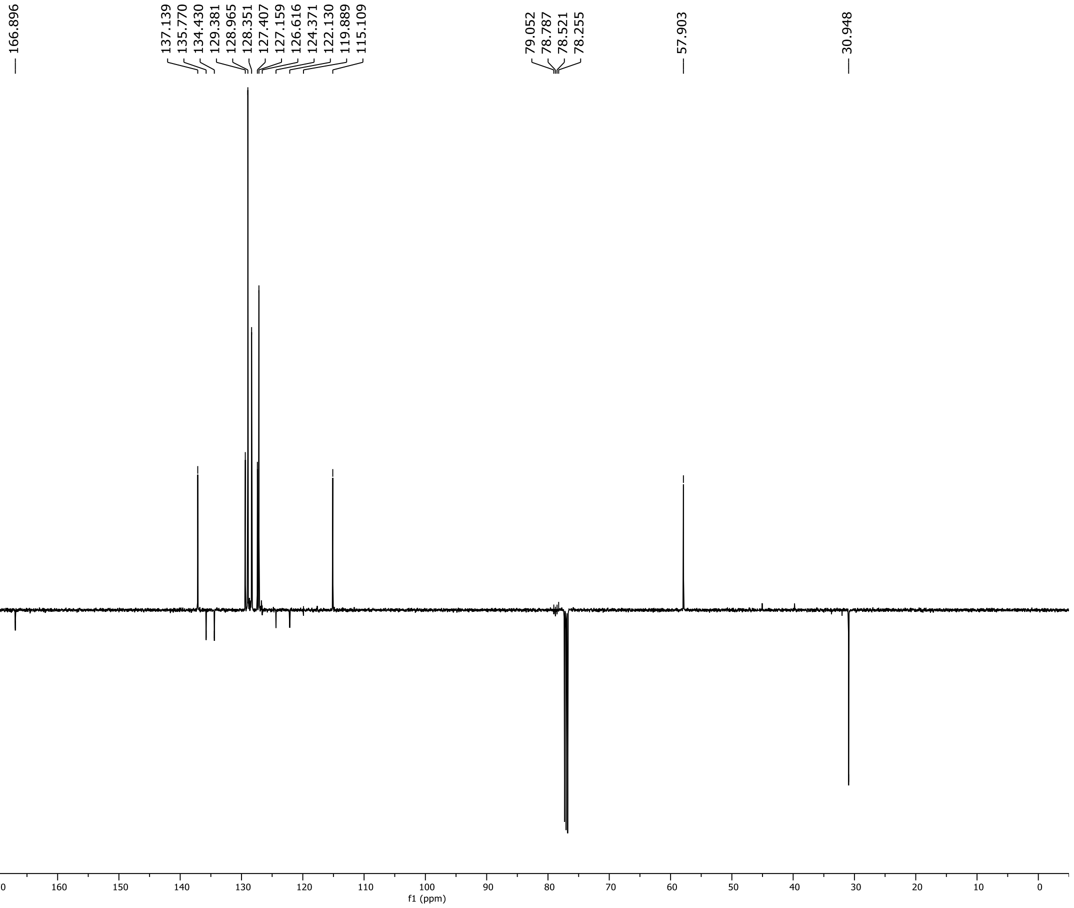

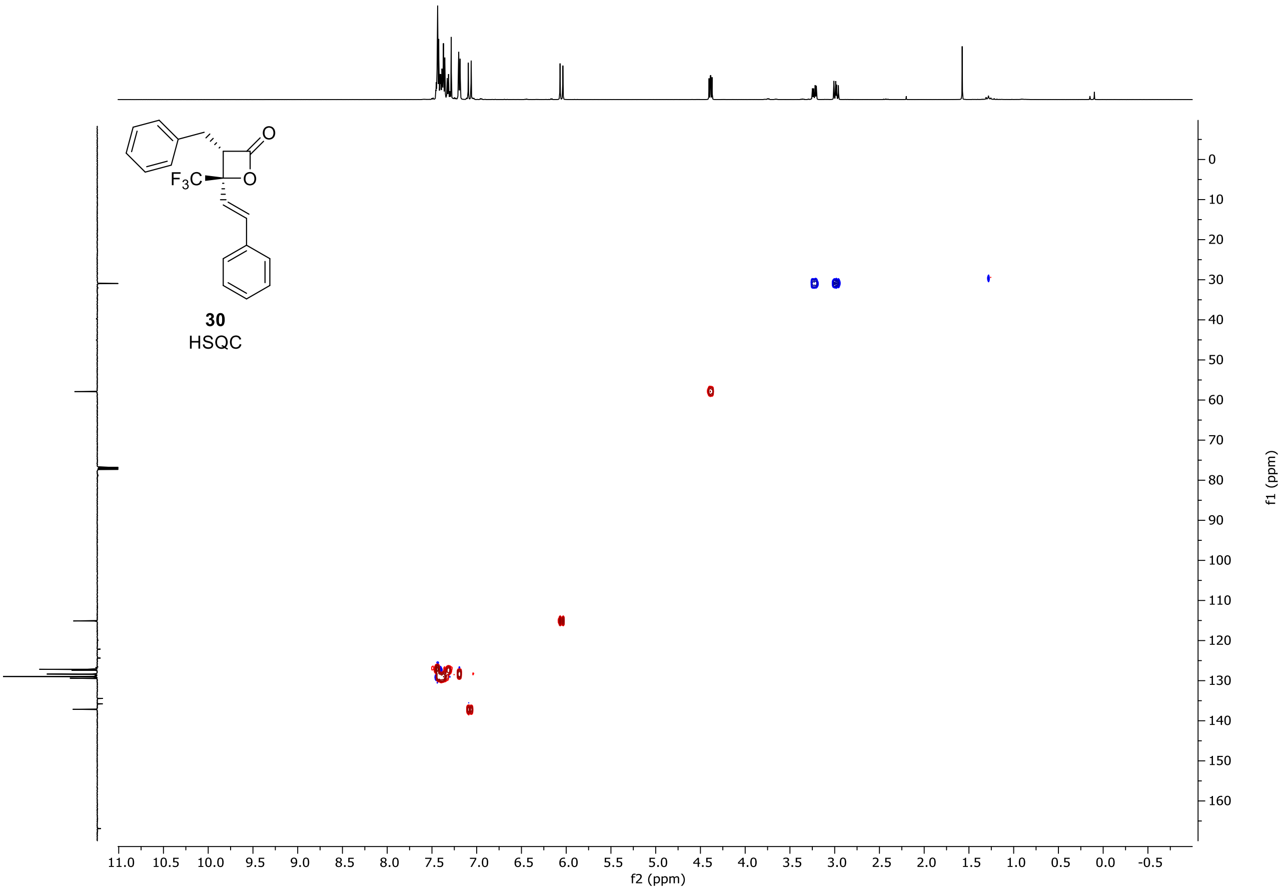
F3C
O
O
30
HSQC
f2 (ppm)
f1 (ppm)

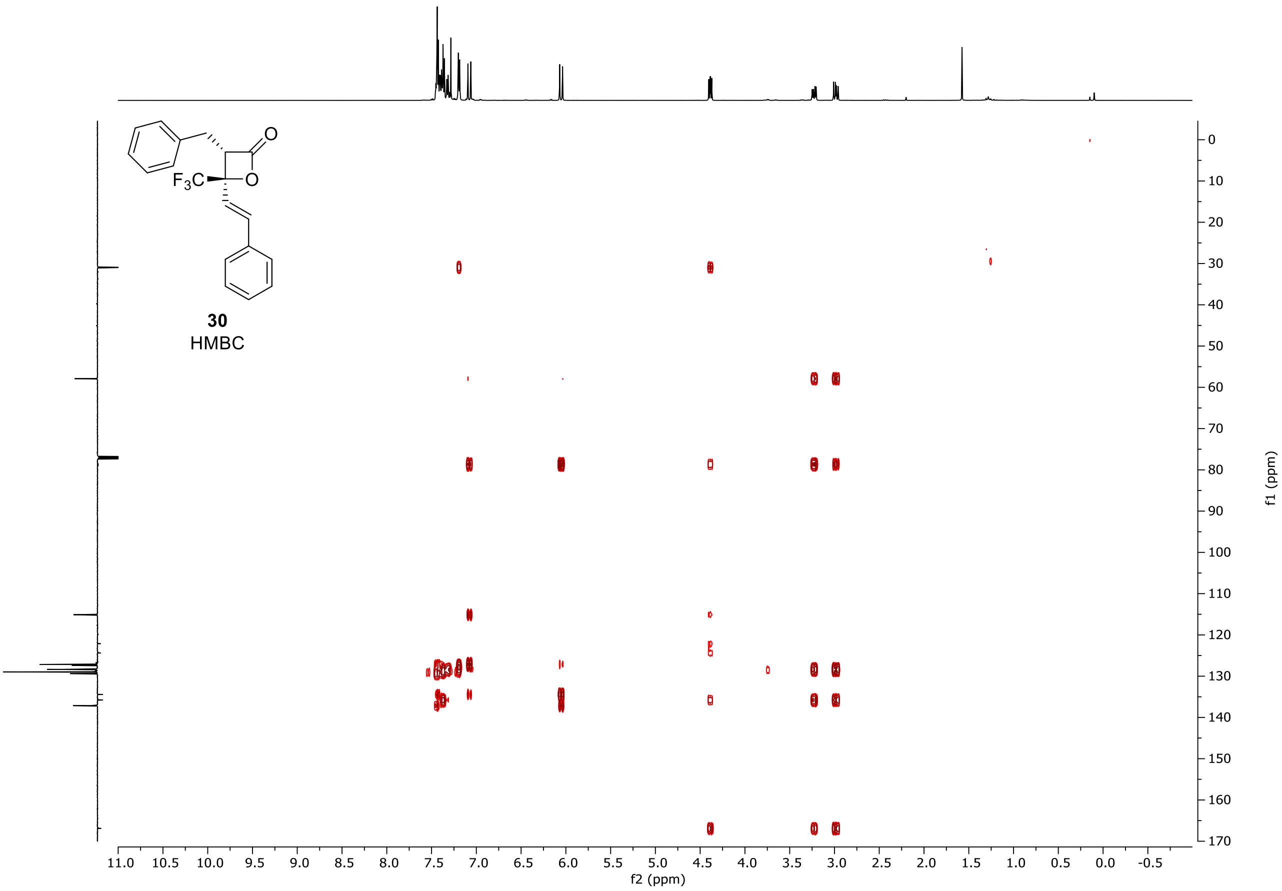
F3C
O
O
30
HMBC
f2 (ppm)
f1 (ppm)

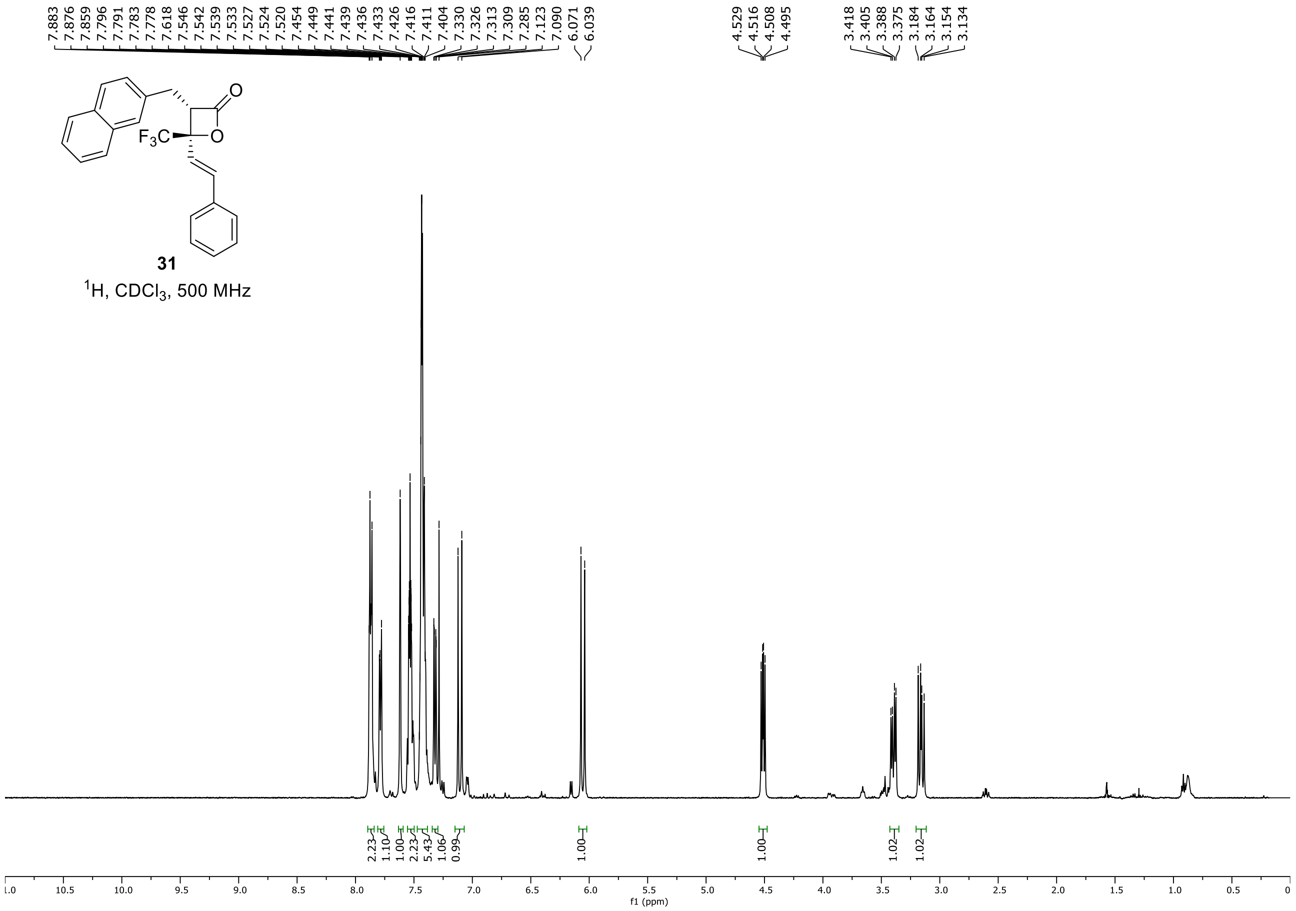
7.883
7.876
7.859
7.796
7.791
7.783
7.778
7.618
7.546
7.542
7.539
7.533
7.527
7.524
7.520
7.454
7.449
7.441
7.439
7.436
7.433
7.426
7.416
7.411
7.404
7.330
7.326
7.313
7.309
7.285
7.123
7.090
6.071
6.039
4.529
4.516
4.508
4.495
3.418
3.405
3.388
3.375
3.184
3.164
3.154
3.134
F_3C
O
O
31
^1H, $CDCl_3$, 500 MHz
2.23
1.10
1.00
2.23
5.43
1.06
0.99
1.00
1.00
1.02
1.02
f1 (ppm)

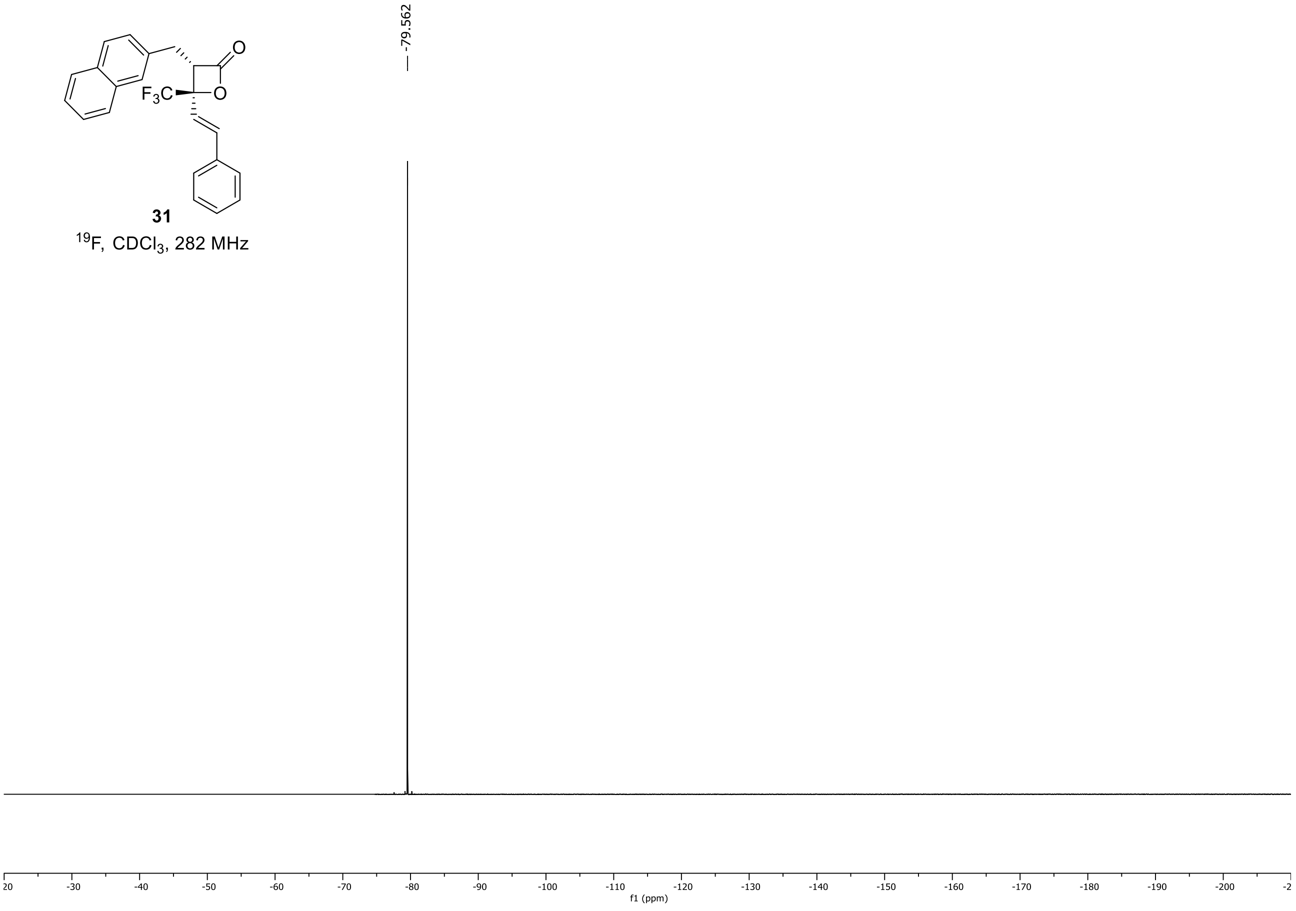
-79.562
F_3C
O
O
31
^{19}F, $CDCl_3$, 282 MHz
f1 (ppm)

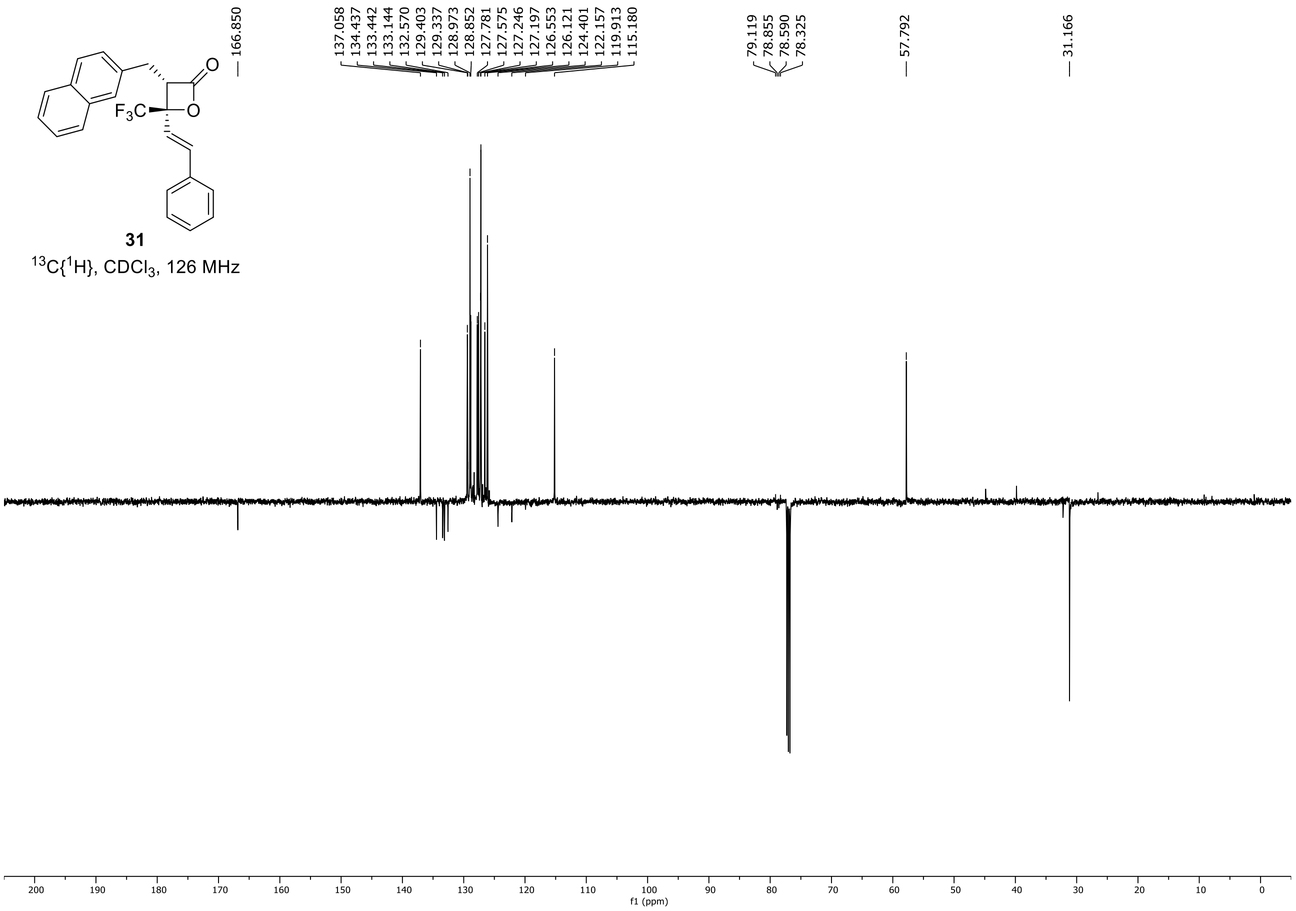

166.850
137.058
134.437
133.442
133.144
132.570
129.403
129.337
128.973
128.852
127.781
127.575
127.246
127.197
126.553
126.121
124.401
122.157
119.913
115.180
79.119
78.855
78.590
78.325
57.792
31.166
F3C
O
31
13C{1H}, CDCl3, 126 MHz
f1 (ppm)

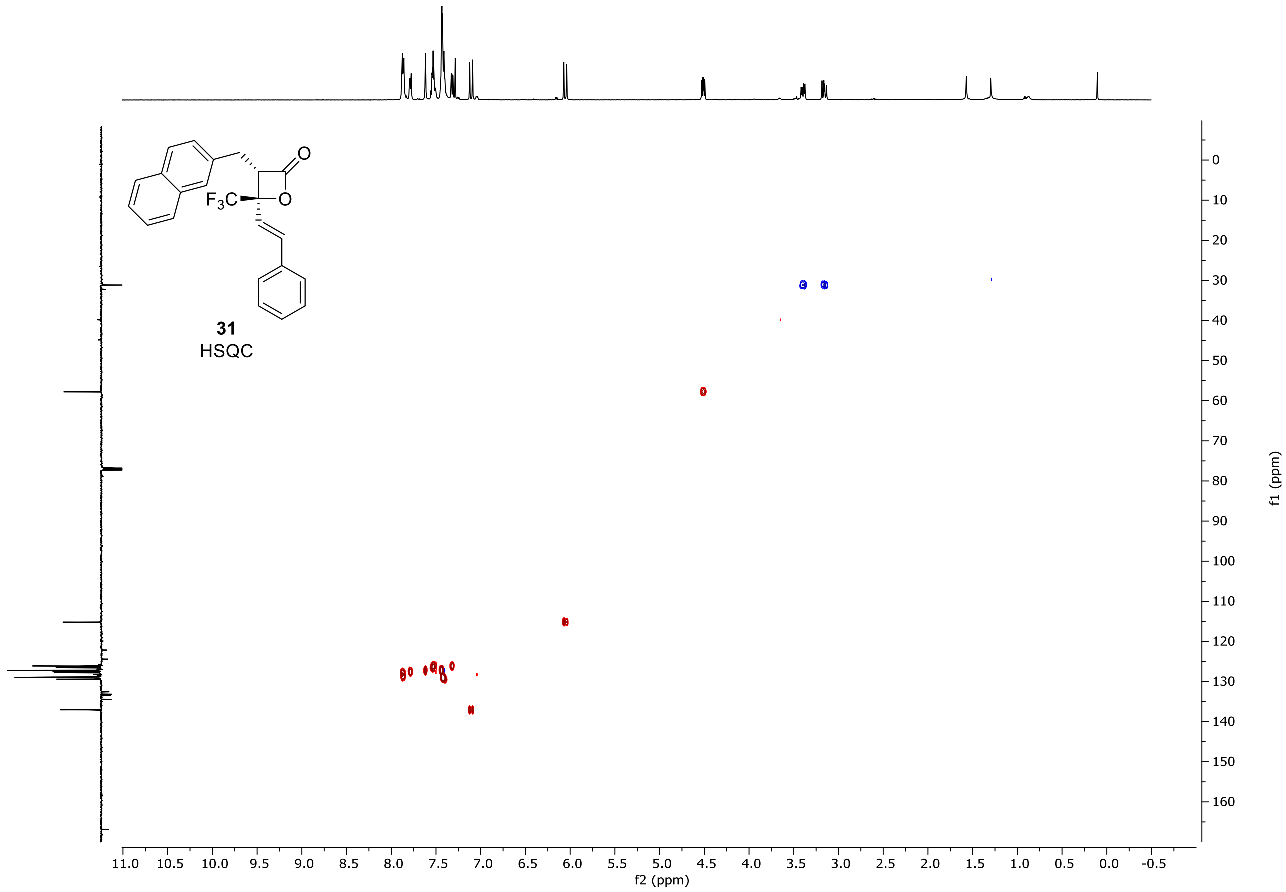

F3C
O
31
HSQC
f2 (ppm)
f1 (ppm)

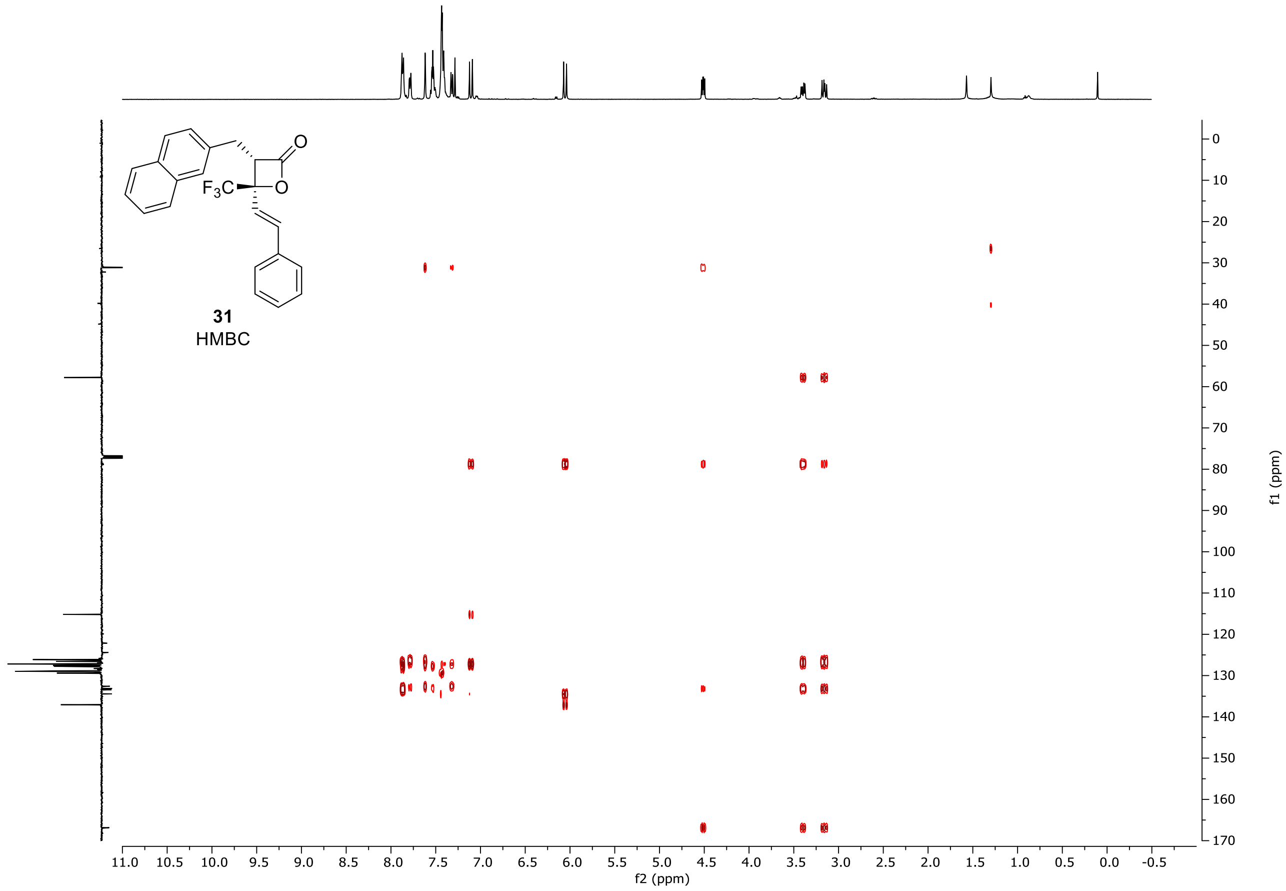
31
HMBC
f2 (ppm)
f1 (ppm)

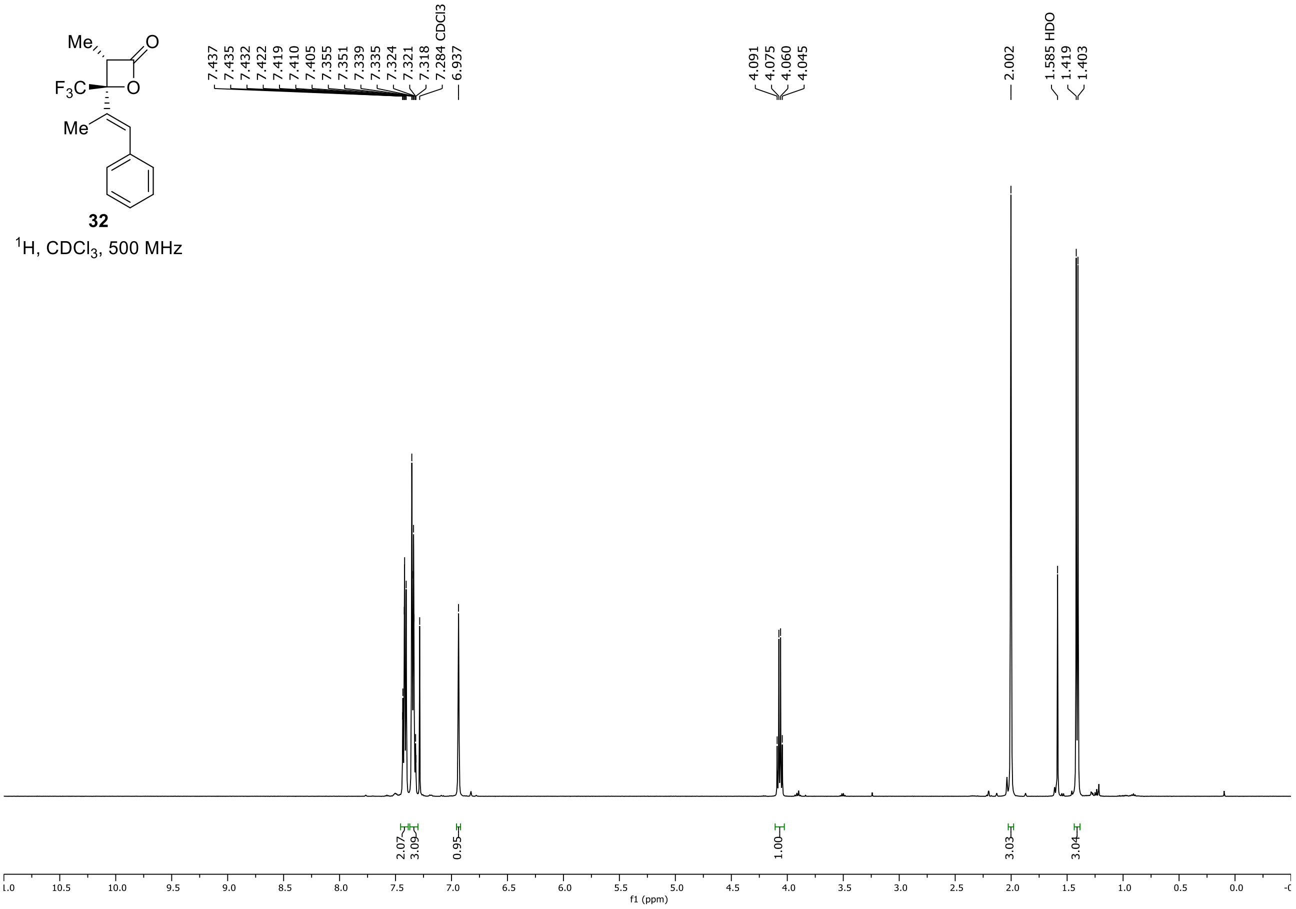
32
^{1}H, CDCl$_3$, 500 MHz
7.437
7.435
7.432
7.422
7.419
7.410
7.405
7.355
7.351
7.339
7.335
7.324
7.321
7.318
7.284 CDCl3
6.937
4.091
4.075
4.060
4.045
2.002
1.585 HDO
1.419
1.403
2.07
3.09
0.95
1.00
3.03
3.04
f1 (ppm)

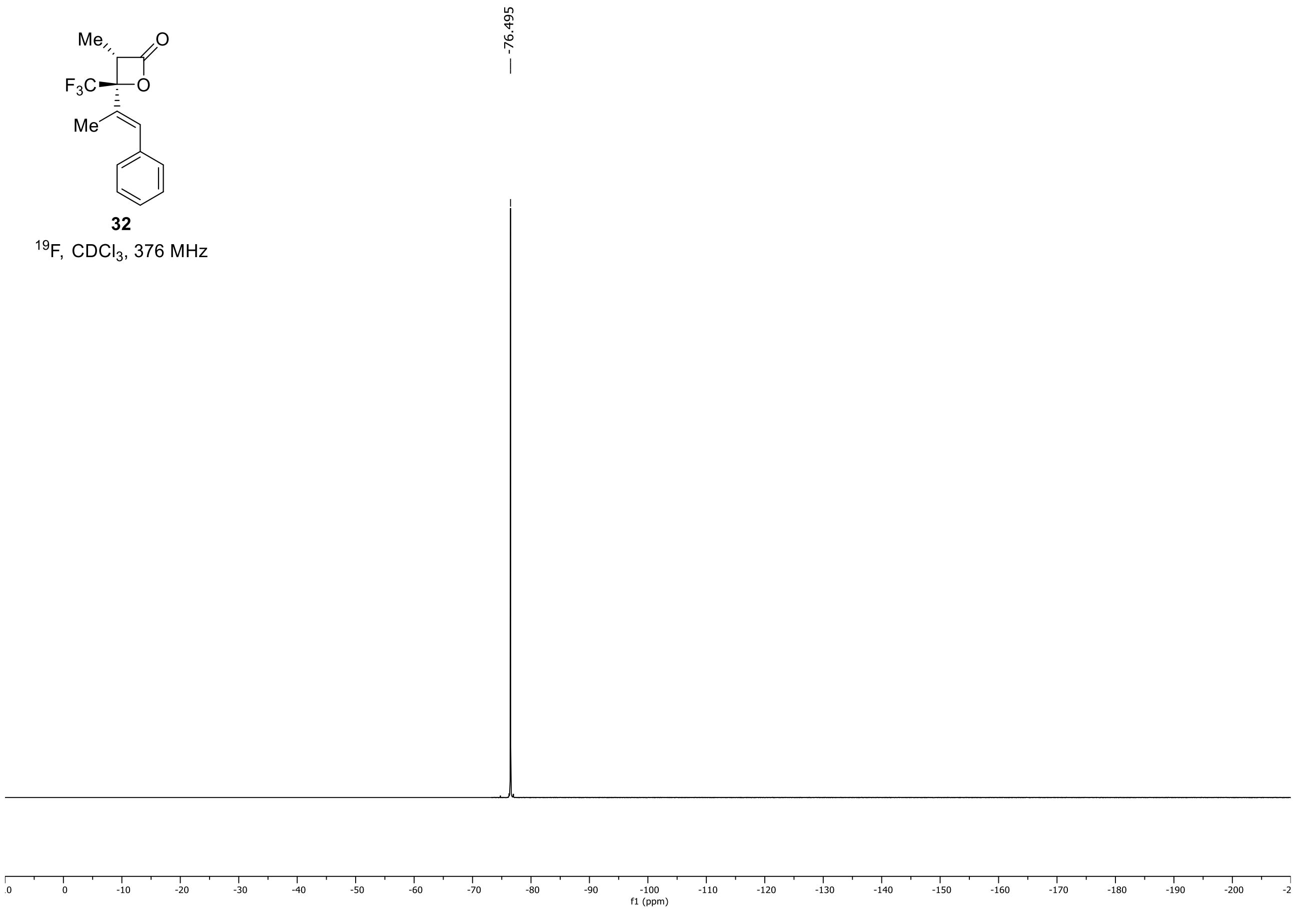

32

^{19}F, $CDCl_3$, 376 MHz

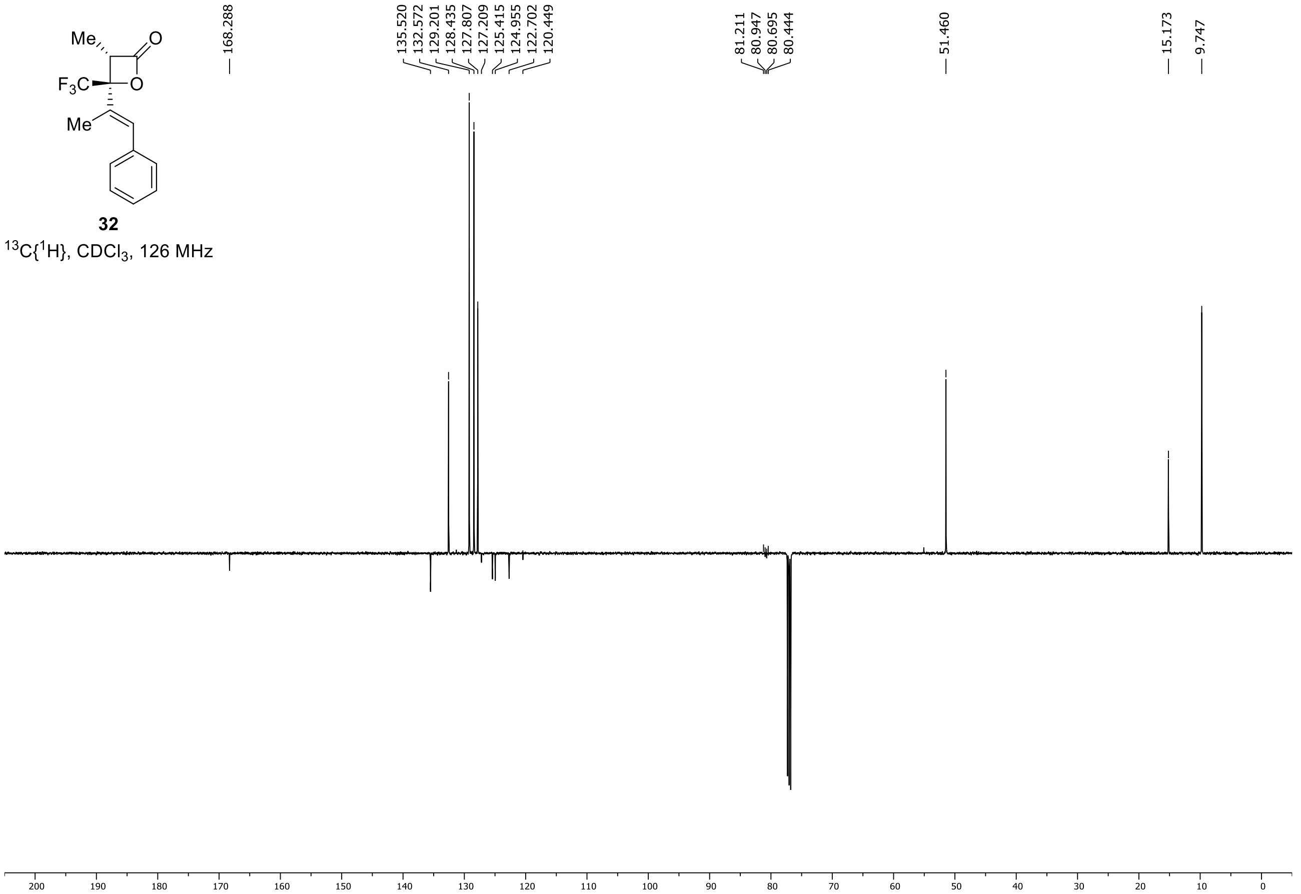

32

^{13}C{^{1}H}, $CDCl_3$, 126 MHz

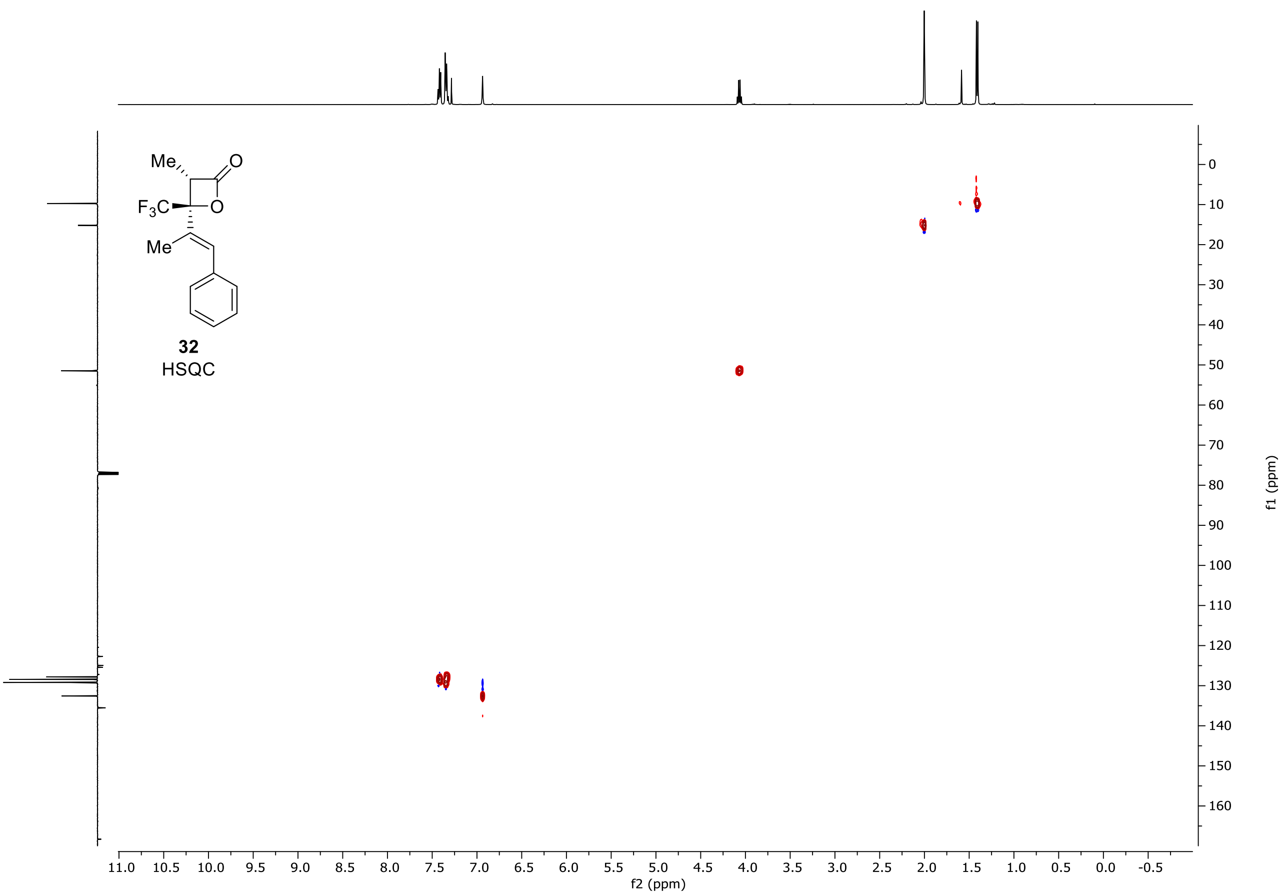
Me
O
F3C
O
Me
32
HSQC
f2 (ppm)
f1 (ppm)

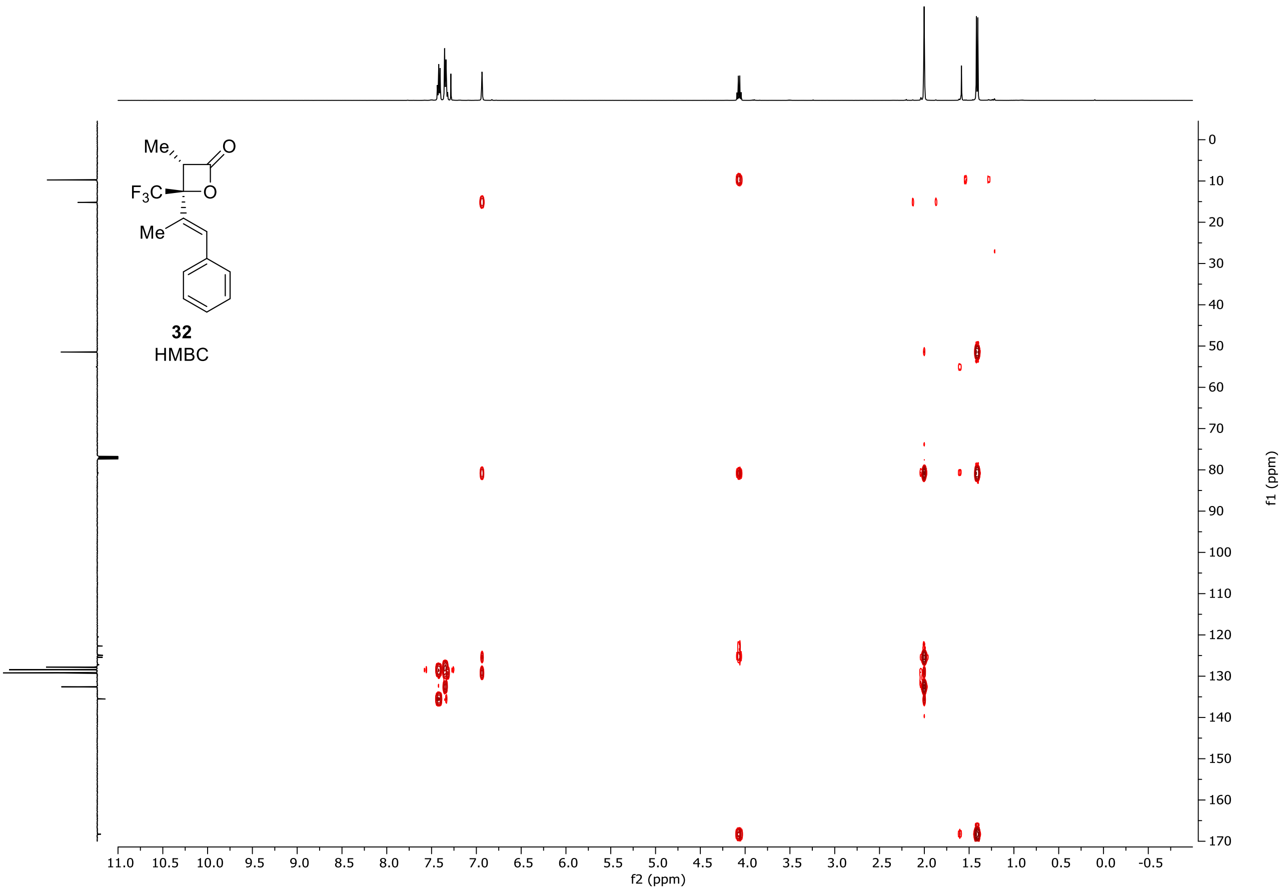
Me
O
F3C
O
Me
32
HMBC
f2 (ppm)
f1 (ppm)

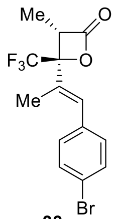

33

^1H, $CDCl_3$, 500 MHz

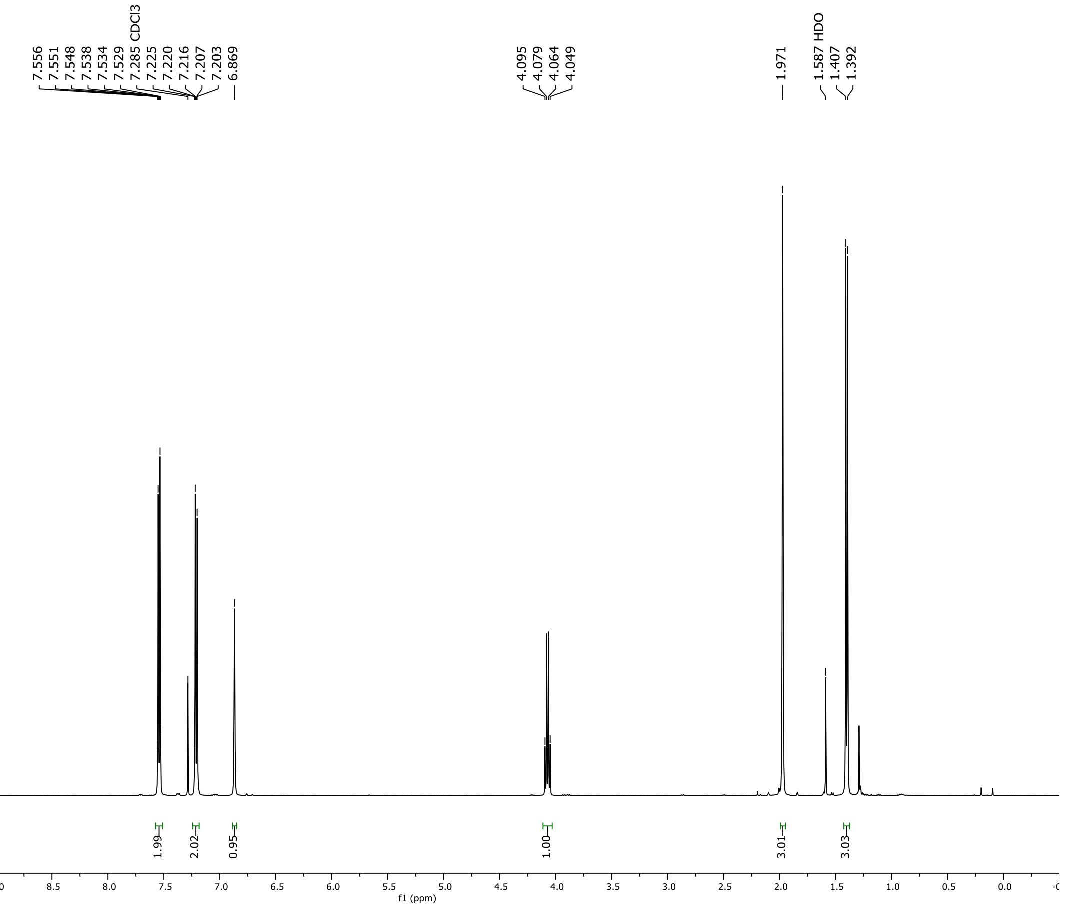

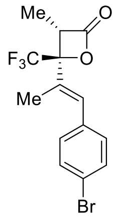

33

^{19}F, $CDCl_3$, 376 MHz

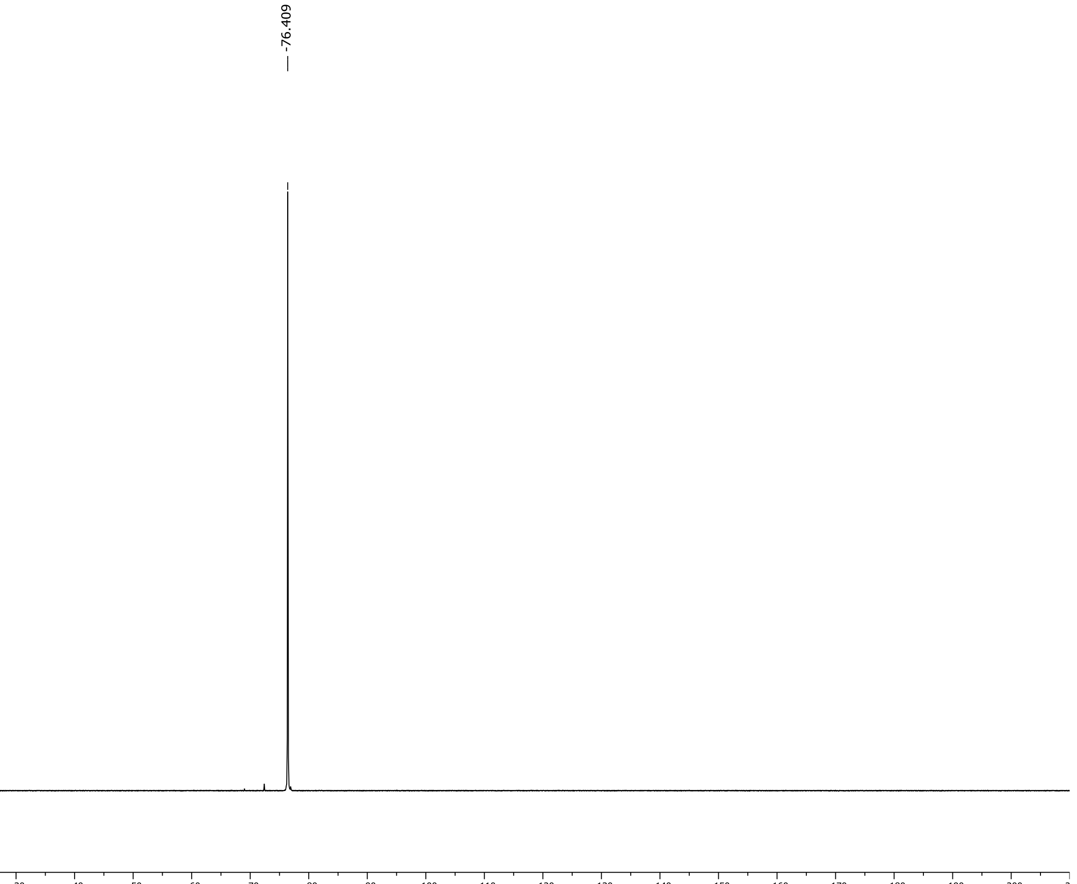

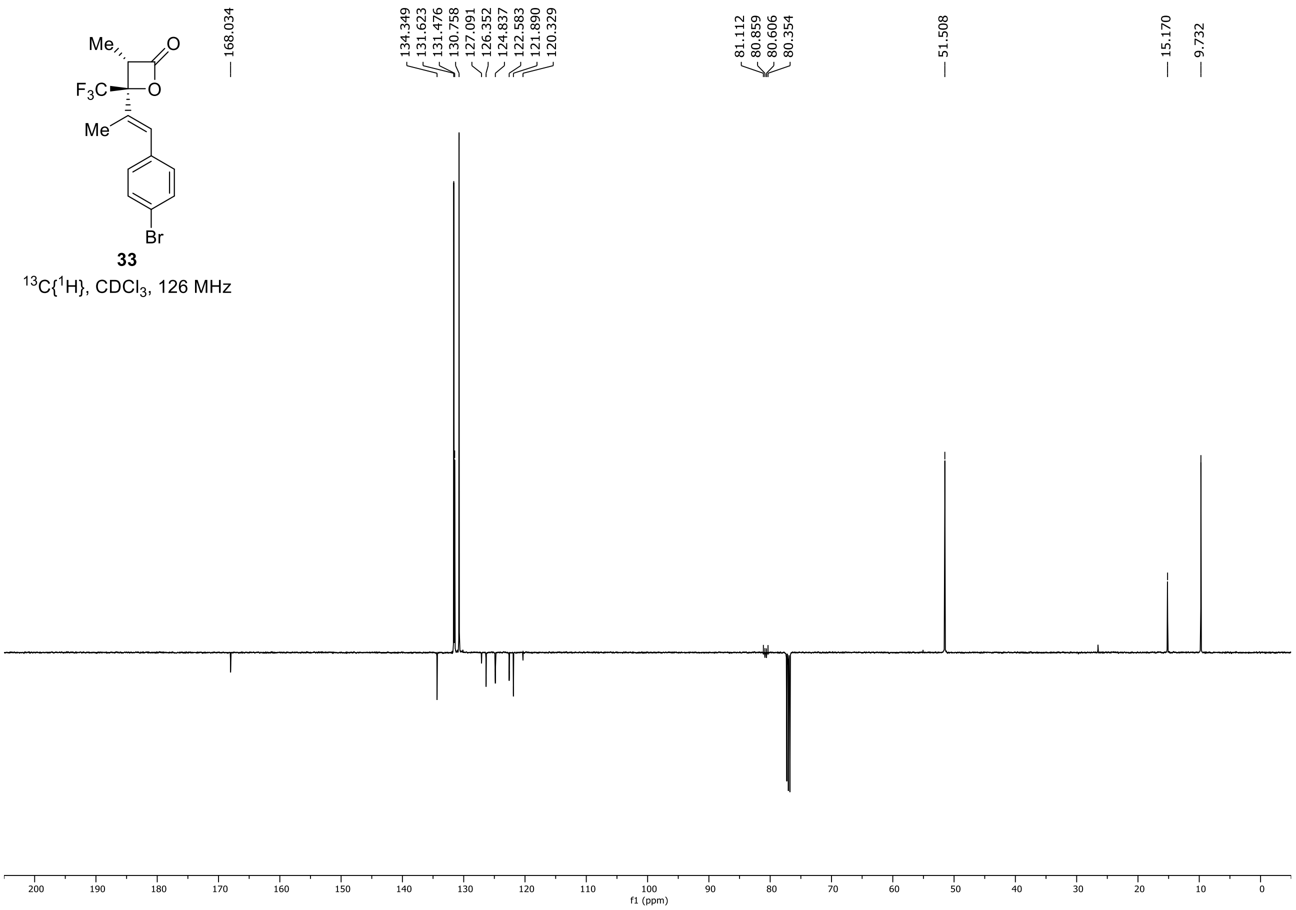

Me
O
F3C
O
Me
Br
33
13C{1H}, CDCl3, 126 MHz
168.034
134.349
131.623
131.476
130.758
127.091
126.352
124.837
122.583
121.890
120.329
81.112
80.859
80.606
80.354
51.508
15.170
9.732
f1 (ppm)

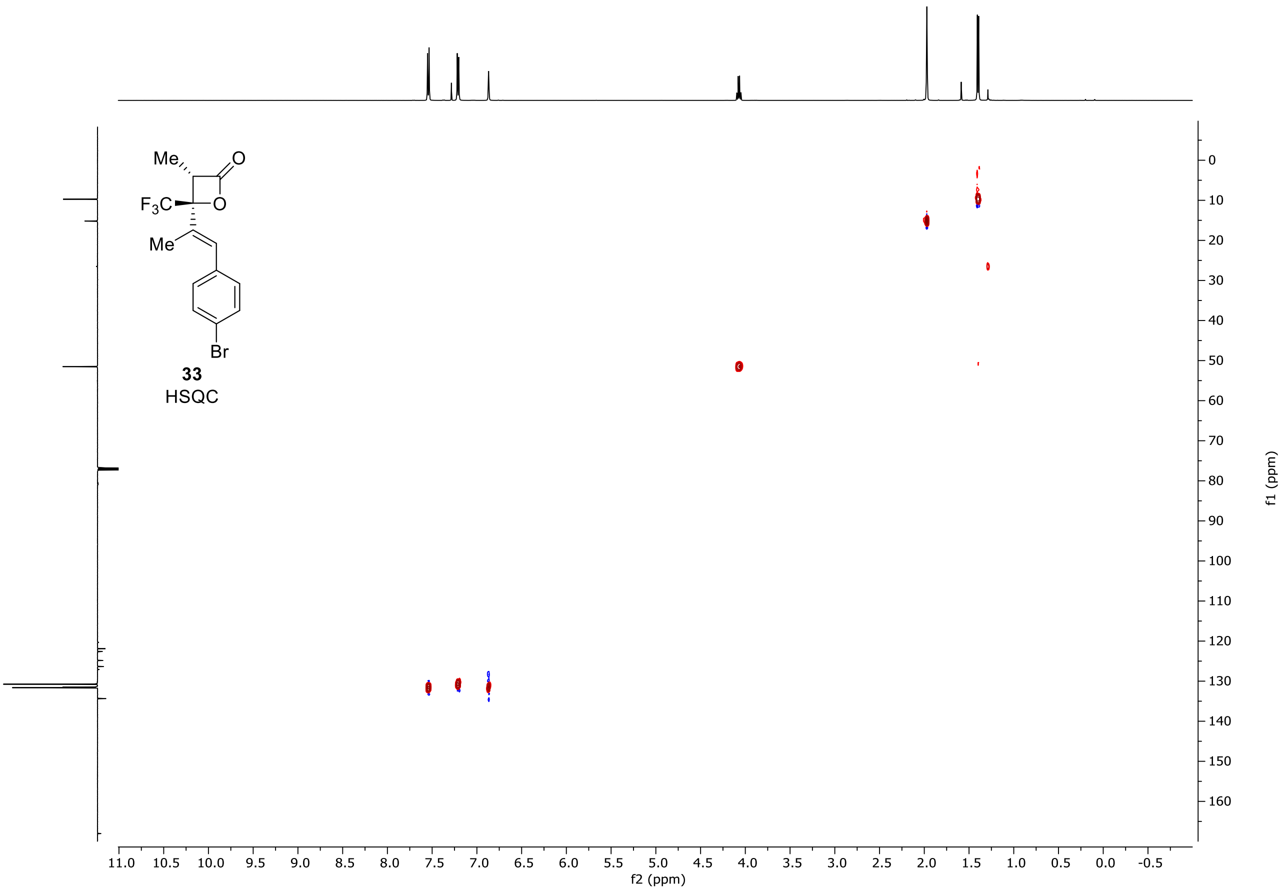

Me
O
F3C
O
Me
Br
33
HSQC
f2 (ppm)
f1 (ppm)

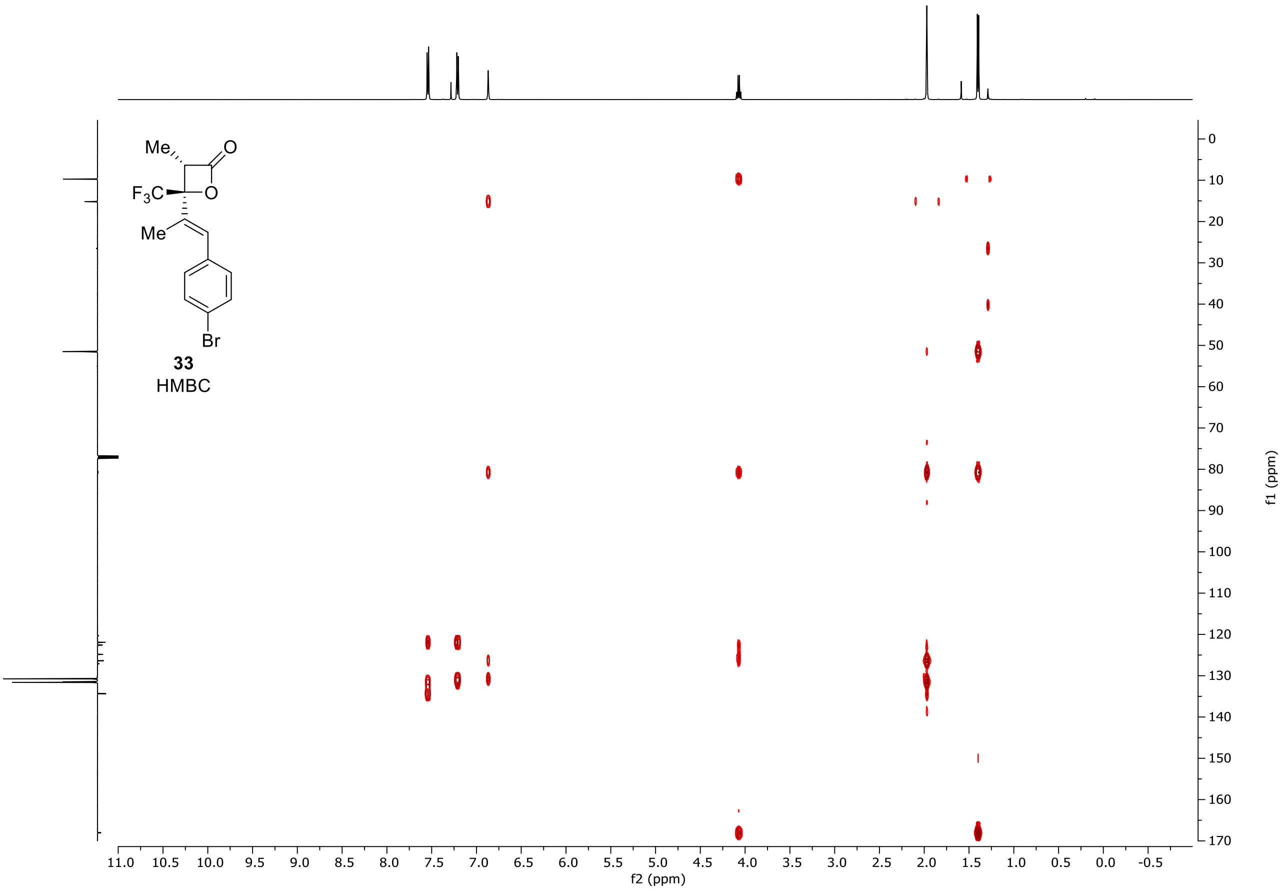

33
HMBC

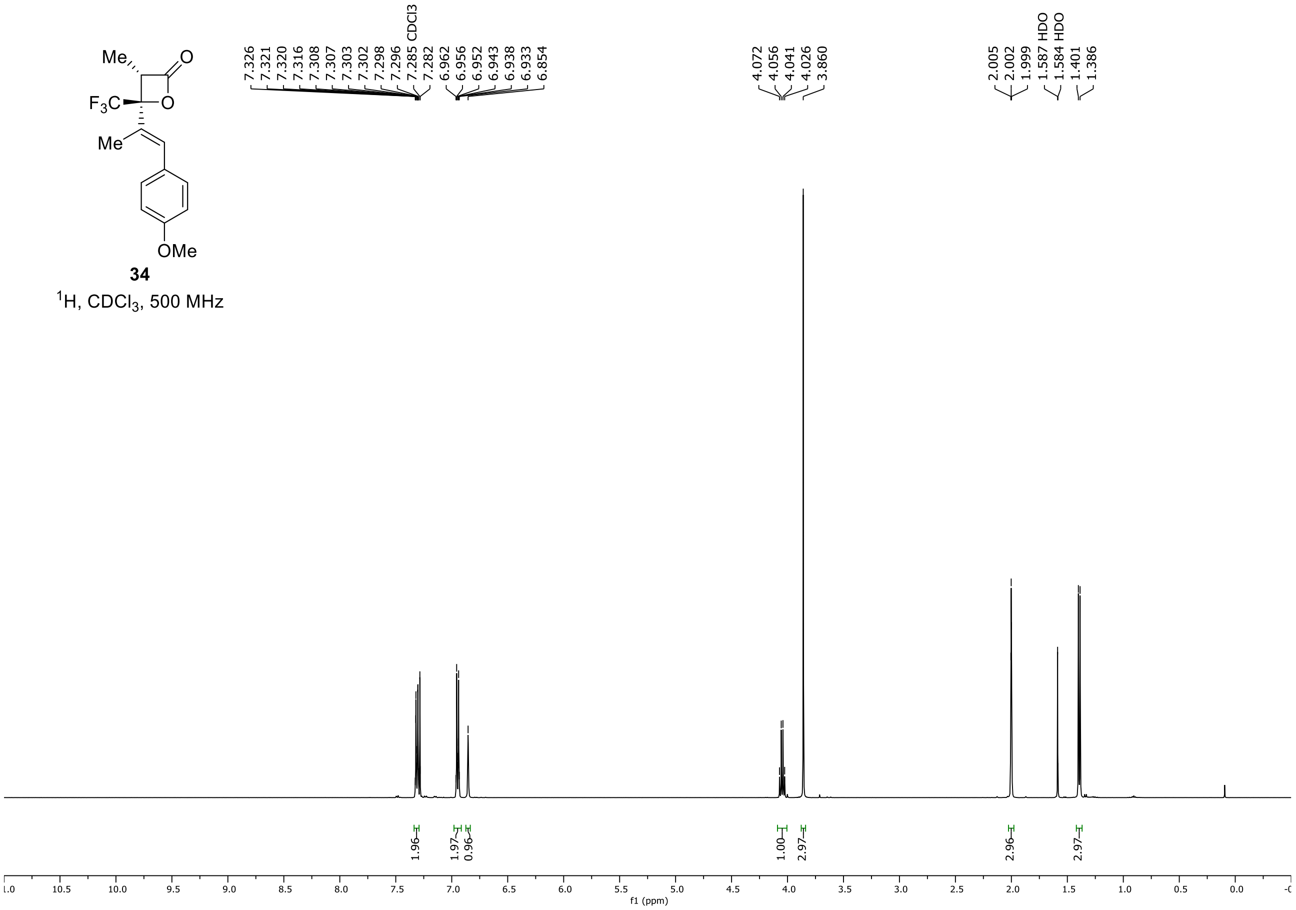

34
^{1}H, $CDCl_3$, 500 MHz

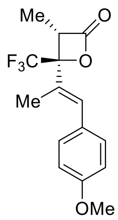

34

^{19}F, $CDCl_3$, 376 MHz

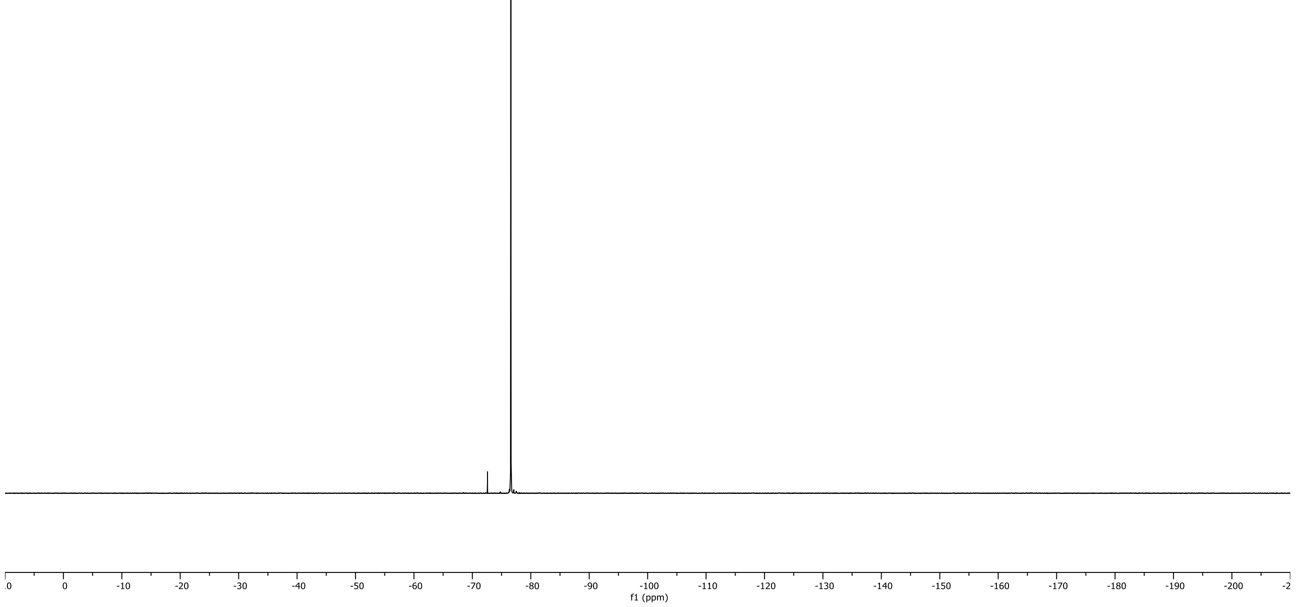

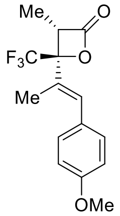

34

$^{13}C\{^1H\}$, $CDCl_3$, 126 MHz

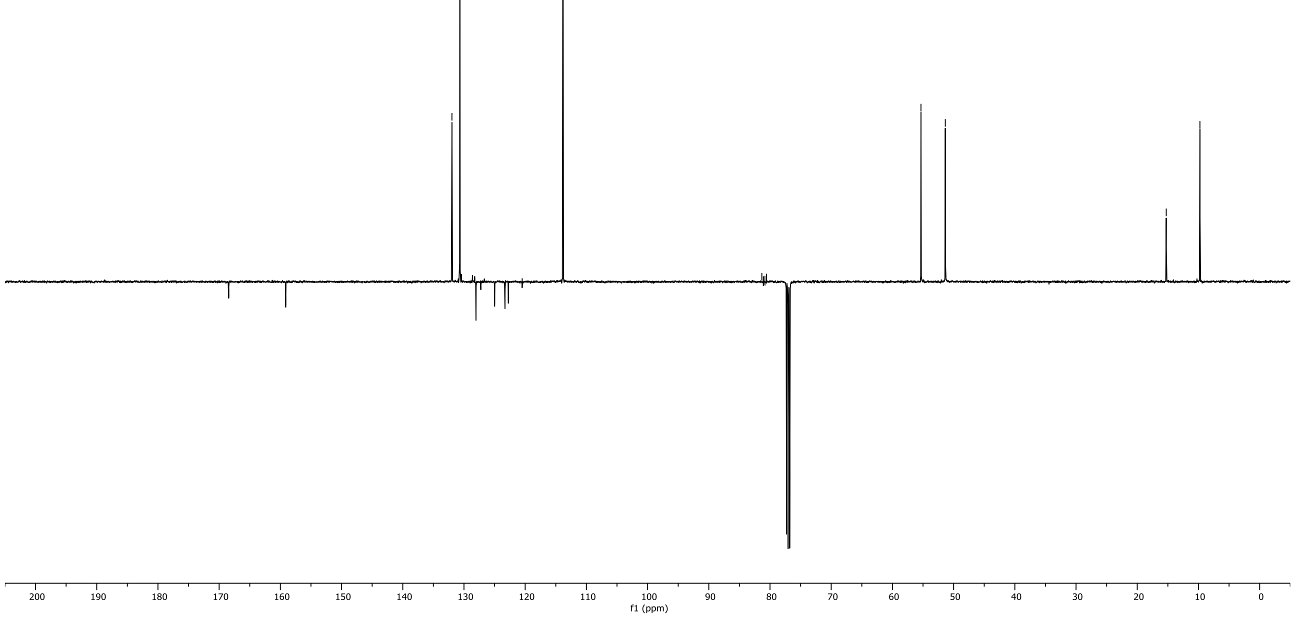

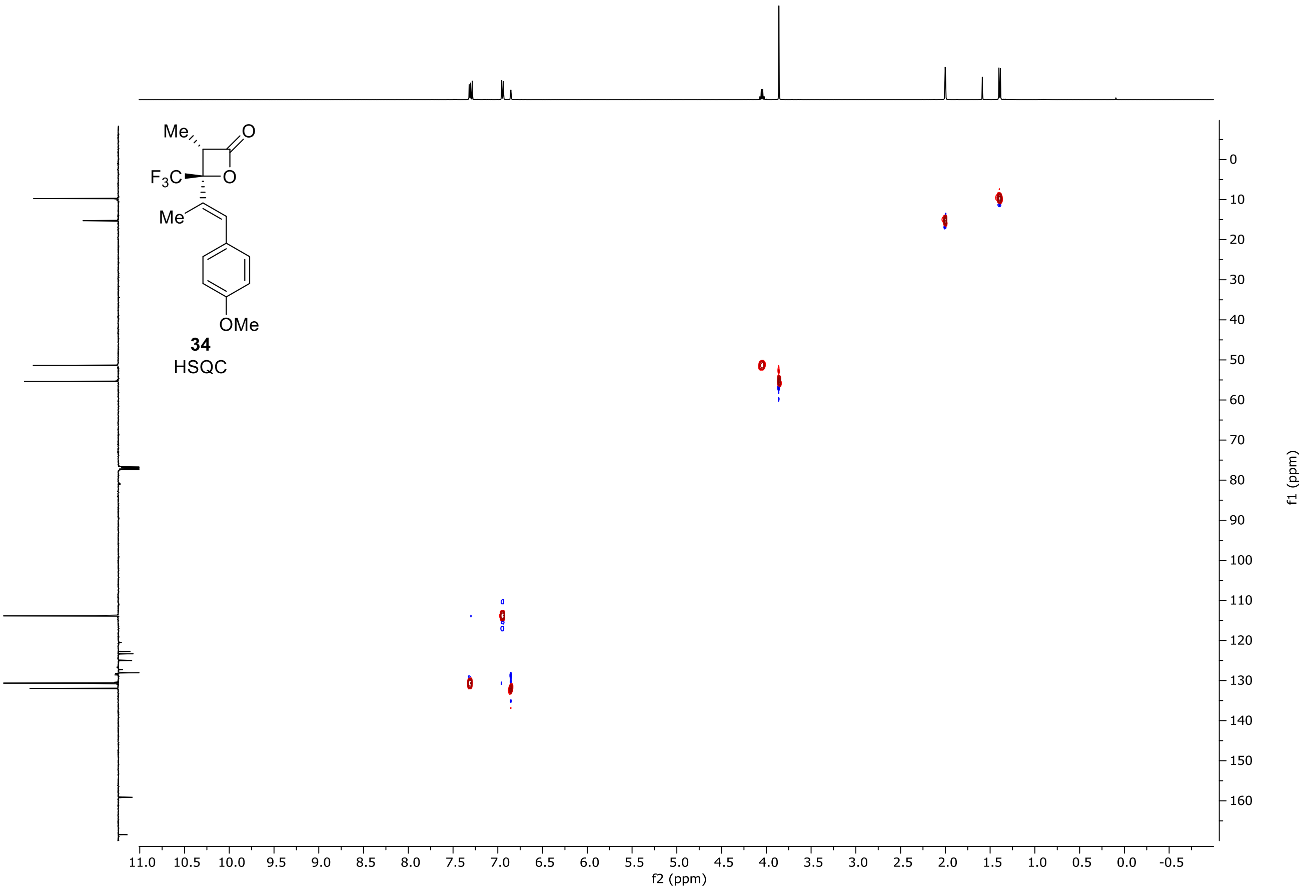
Me
O
F3C
O
Me
OMe
34
HSQC
f2 (ppm)
f1 (ppm)

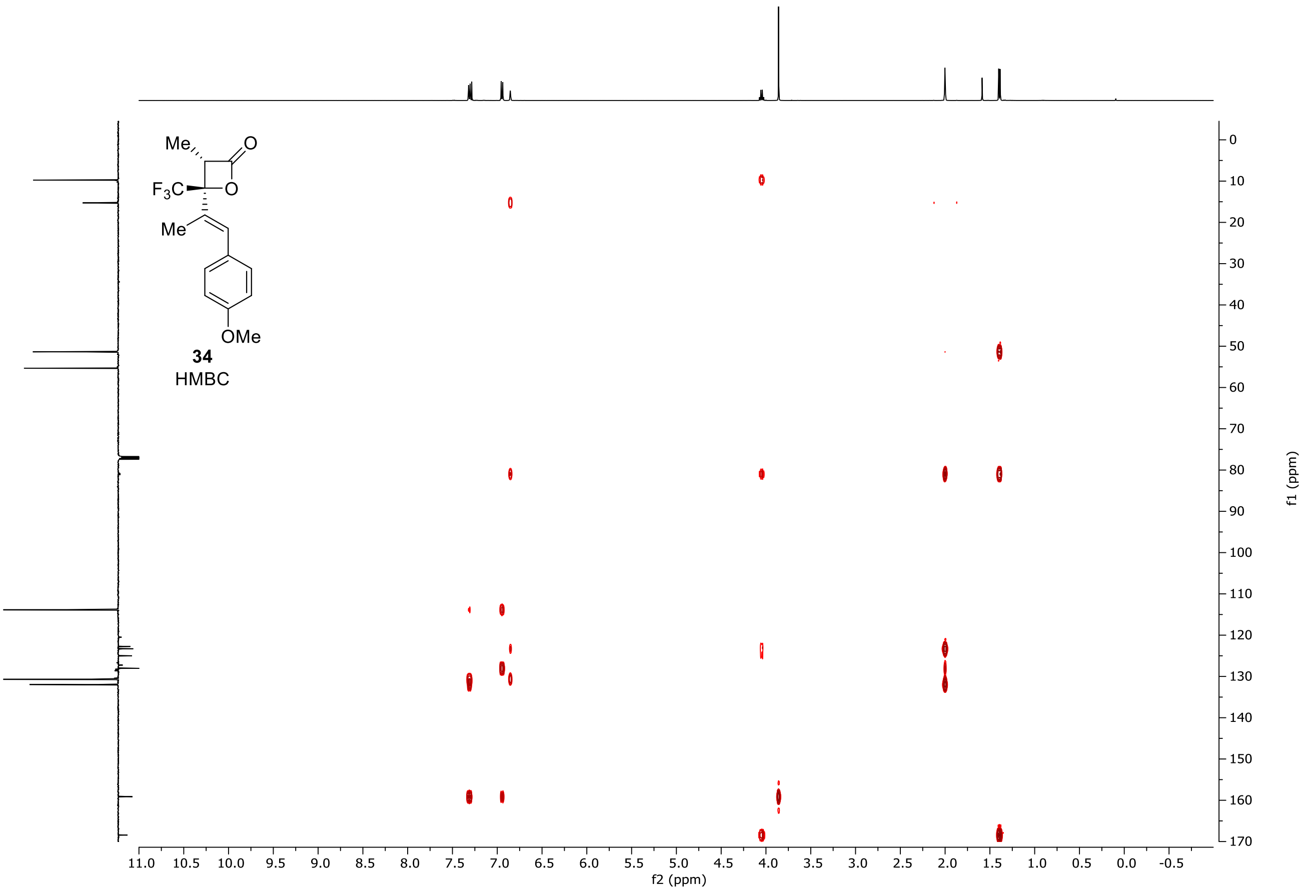
Me
O
F3C
O
Me
OMe
34
HMBC
f2 (ppm)
f1 (ppm)

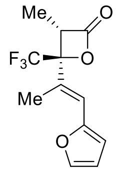

35

^{1}H, $CDCl_3$, 500 MHz

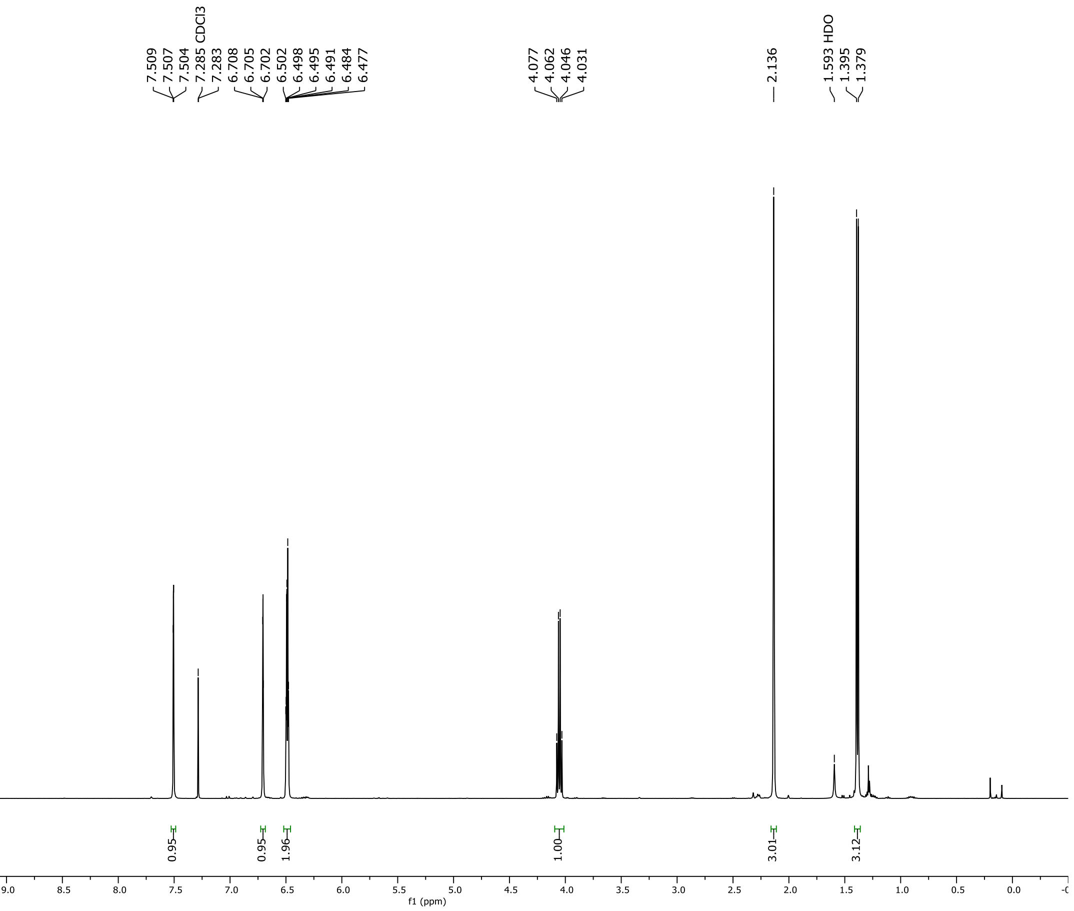

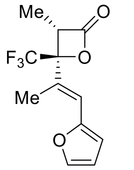

35

^{19}F, $CDCl_3$, 376 MHz

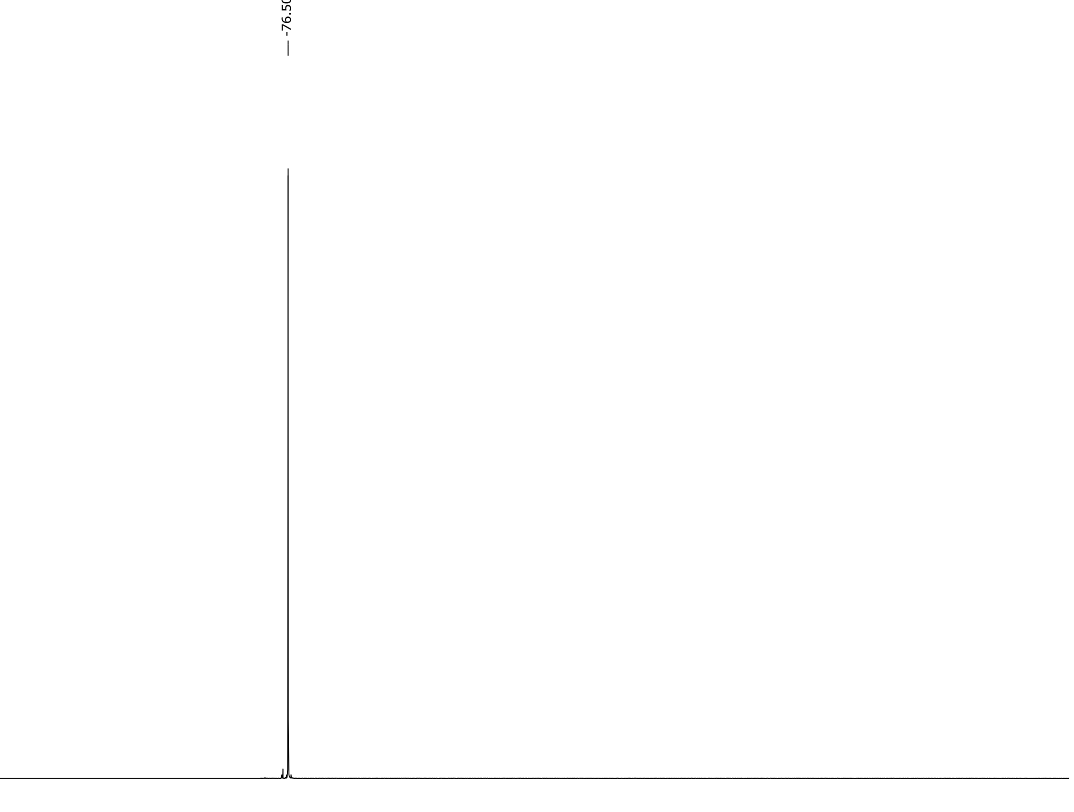

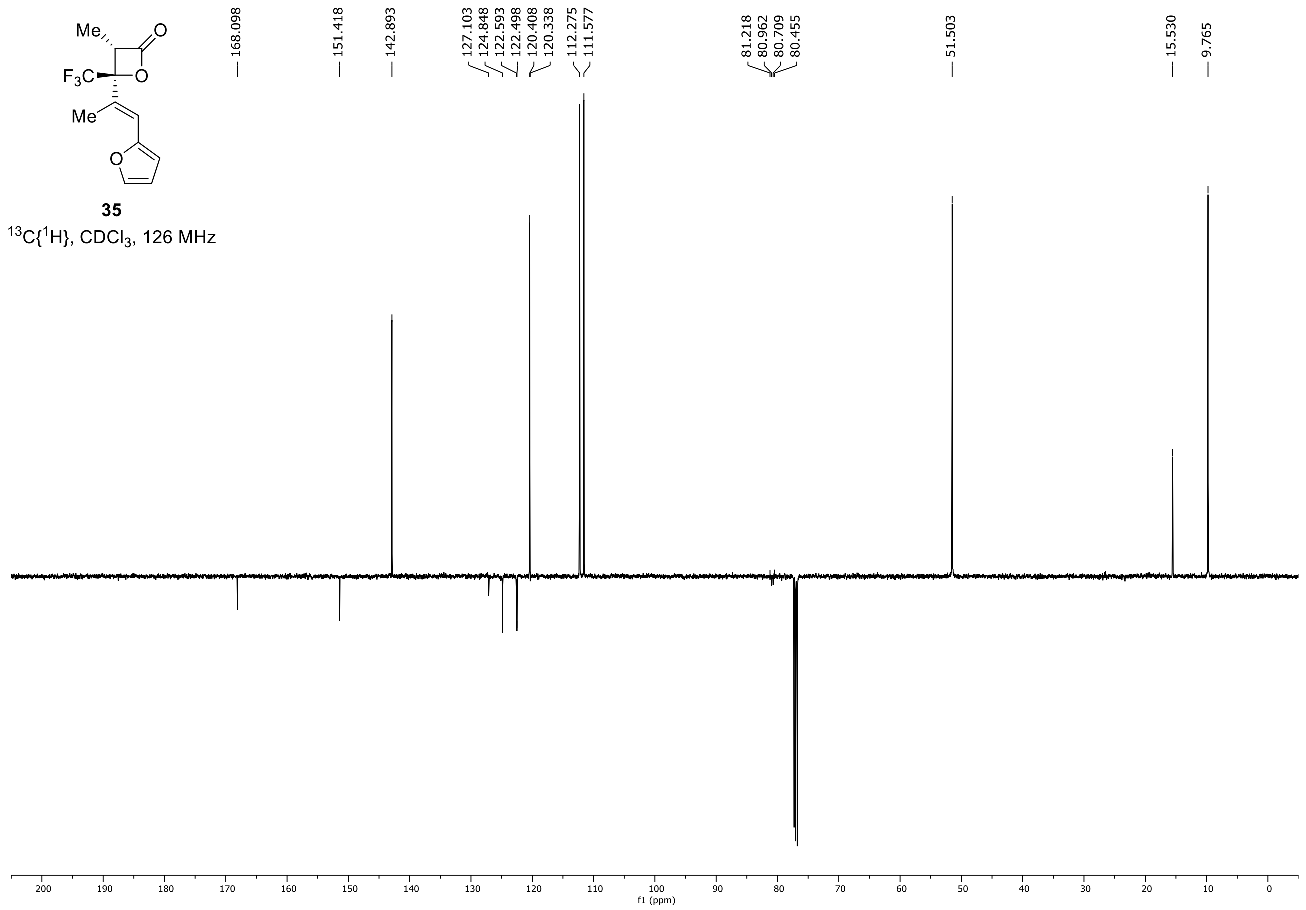
Me
O
F3C
O
Me
O
35
13C{1H}, CDCl3, 126 MHz
168.098
151.418
142.893
127.103
124.848
122.593
122.498
120.408
120.338
112.275
111.577
81.218
80.962
80.709
80.455
51.503
15.530
9.765
f1 (ppm)

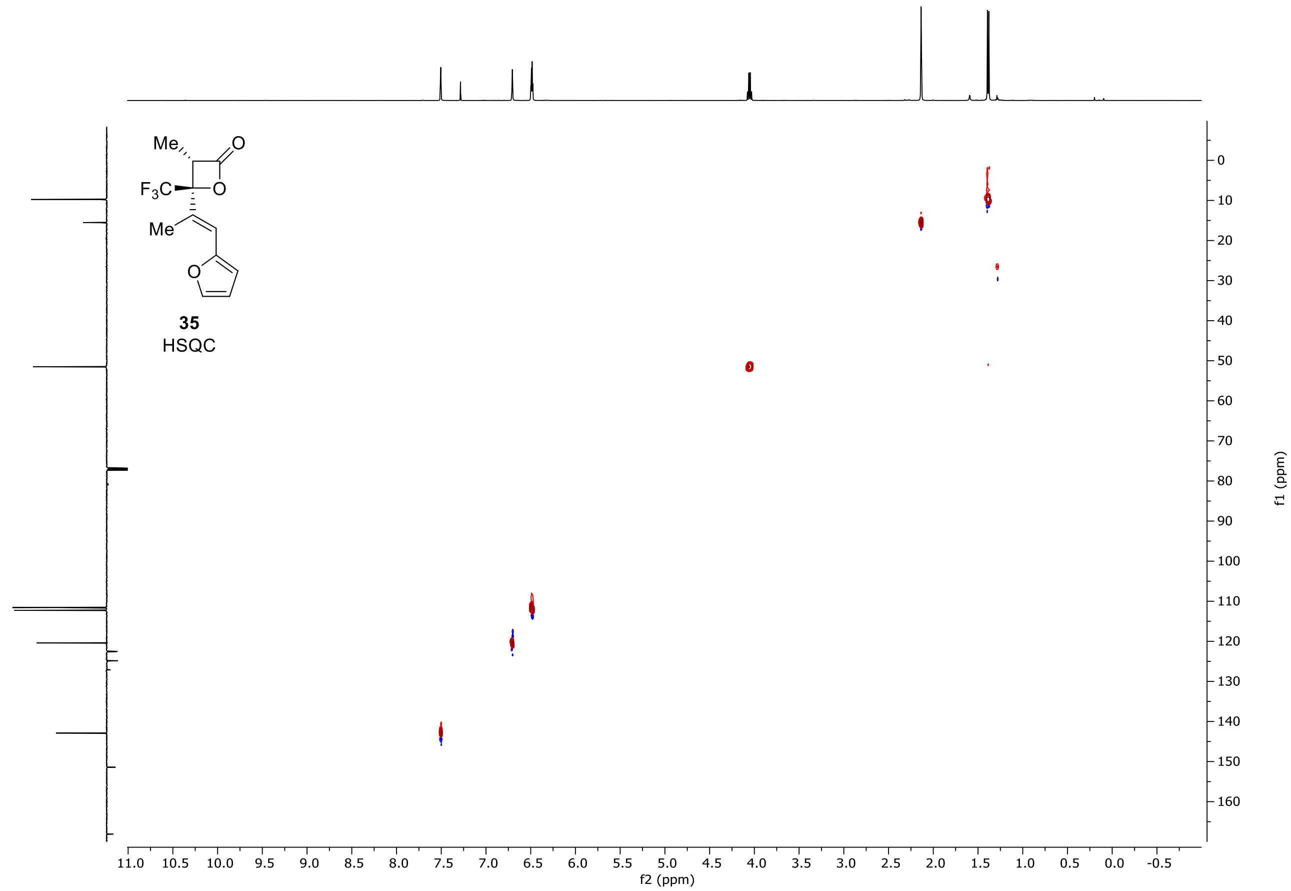
Me
O
F3C
O
Me
O
35
HSQC
f2 (ppm)
f1 (ppm)

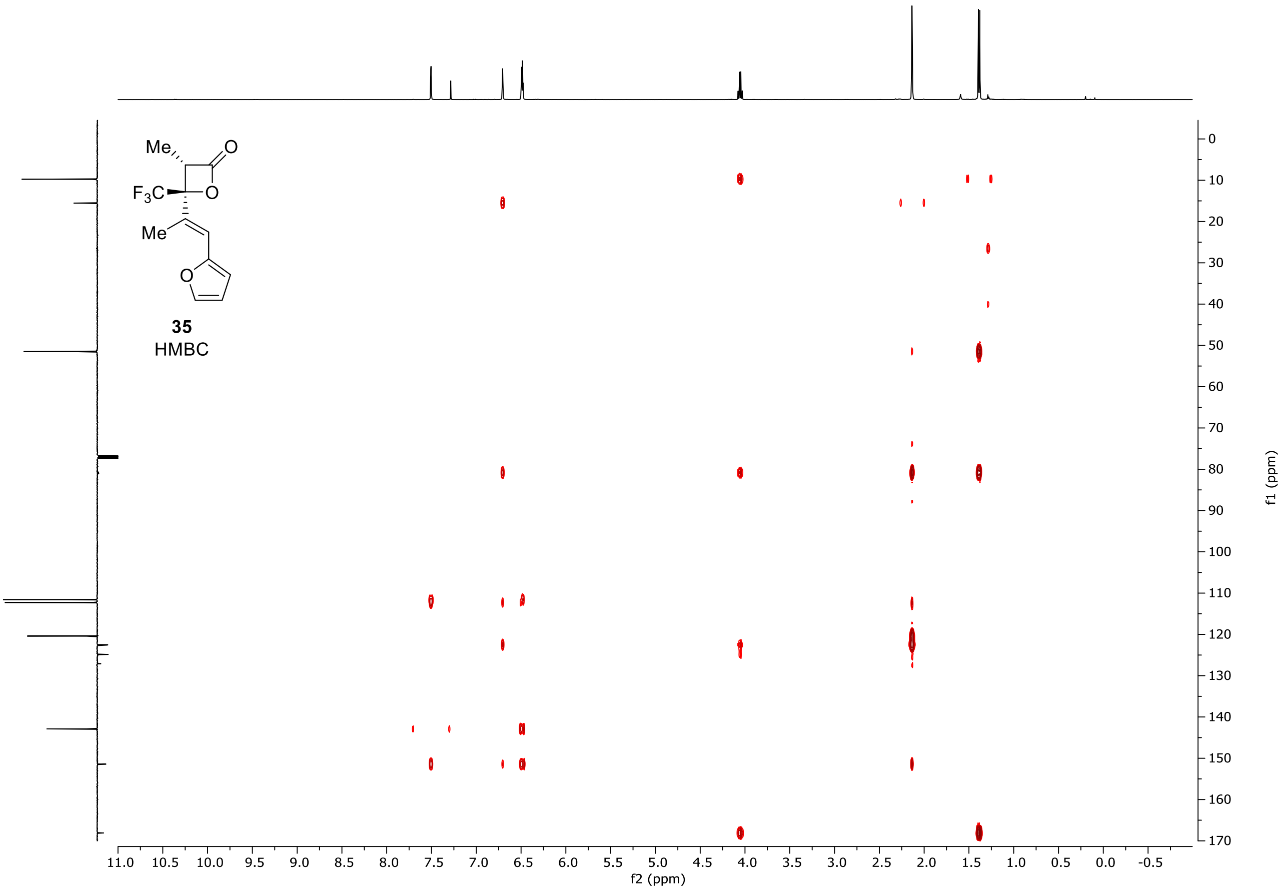

35
HMBC

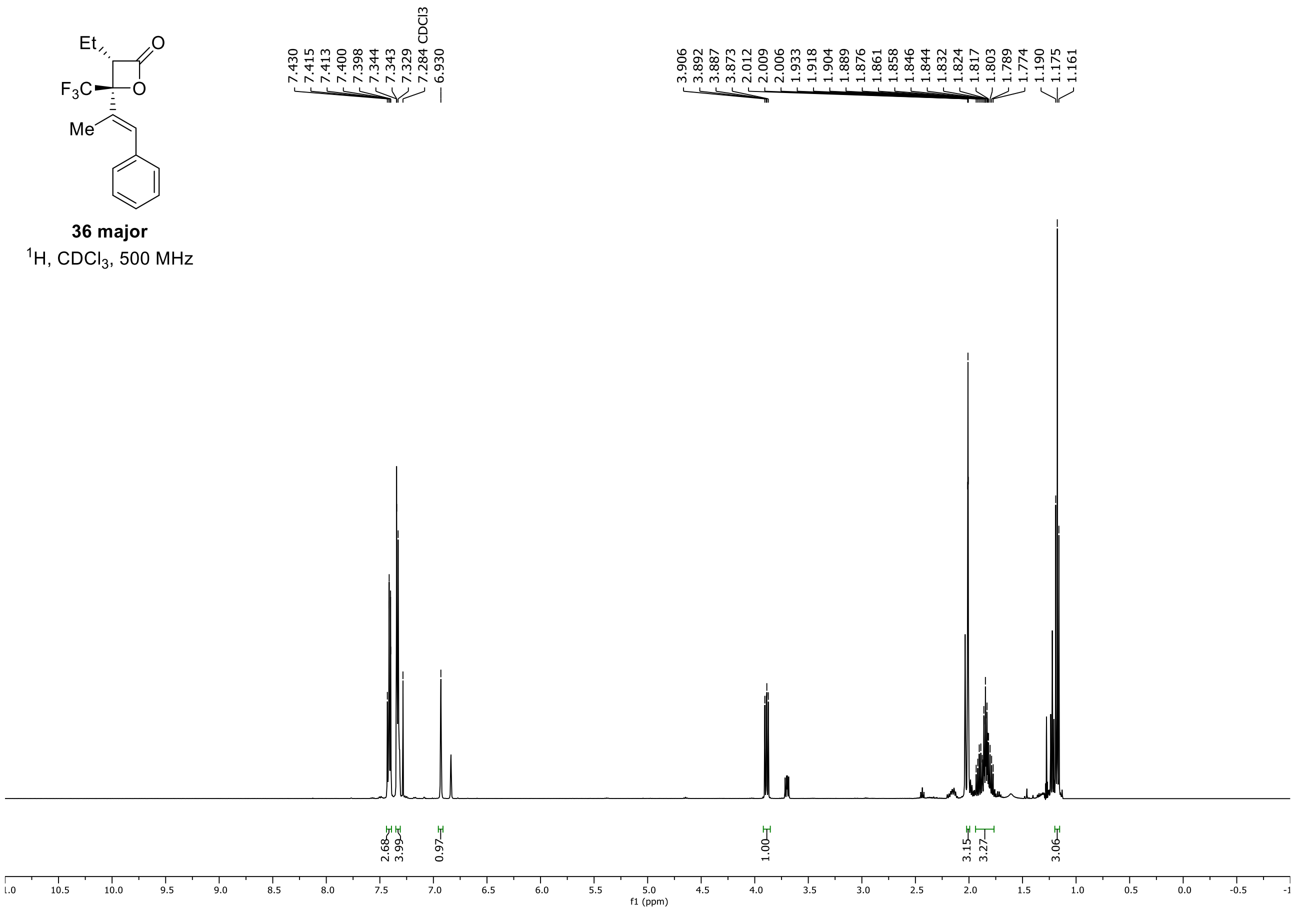

36 major
^{1}H, $CDCl_3$, 500 MHz

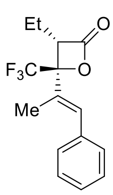

36 major

^{19}F, $CDCl_3$, 376 MHz

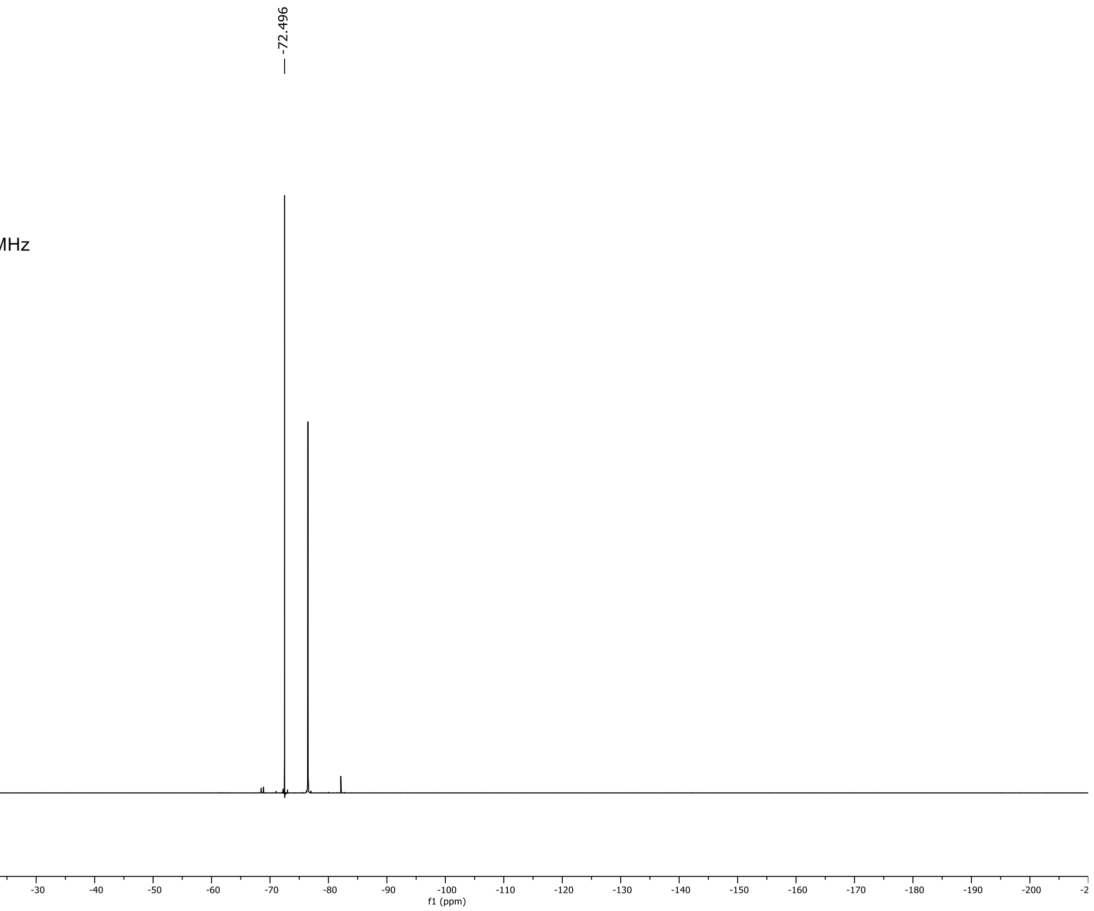

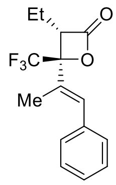

36 major

$^{13}C\{^1H\}$, $CDCl_3$, 126 MHz

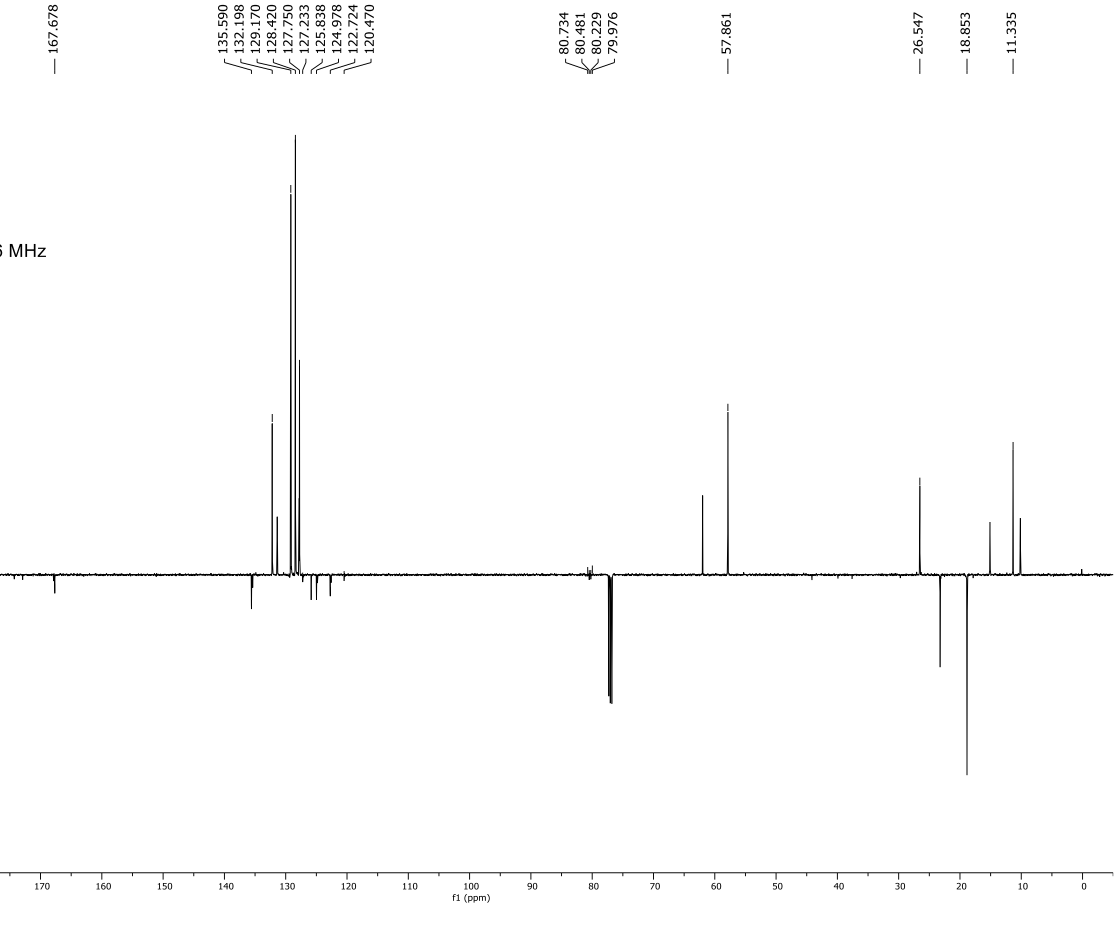

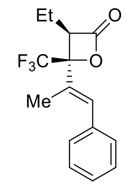

36 minor

^{1}H, $CDCl_3$, 500 MHz

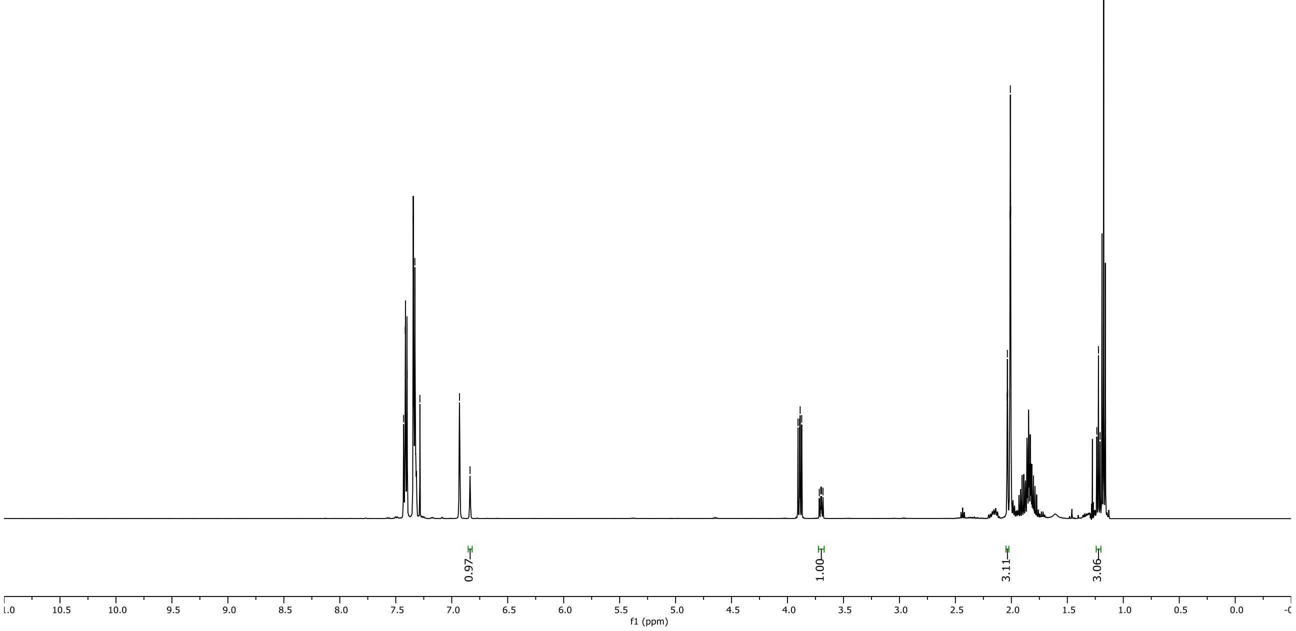

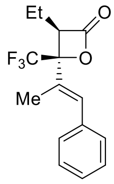

36 minor

^{19}F, $CDCl_3$, 376 MHz

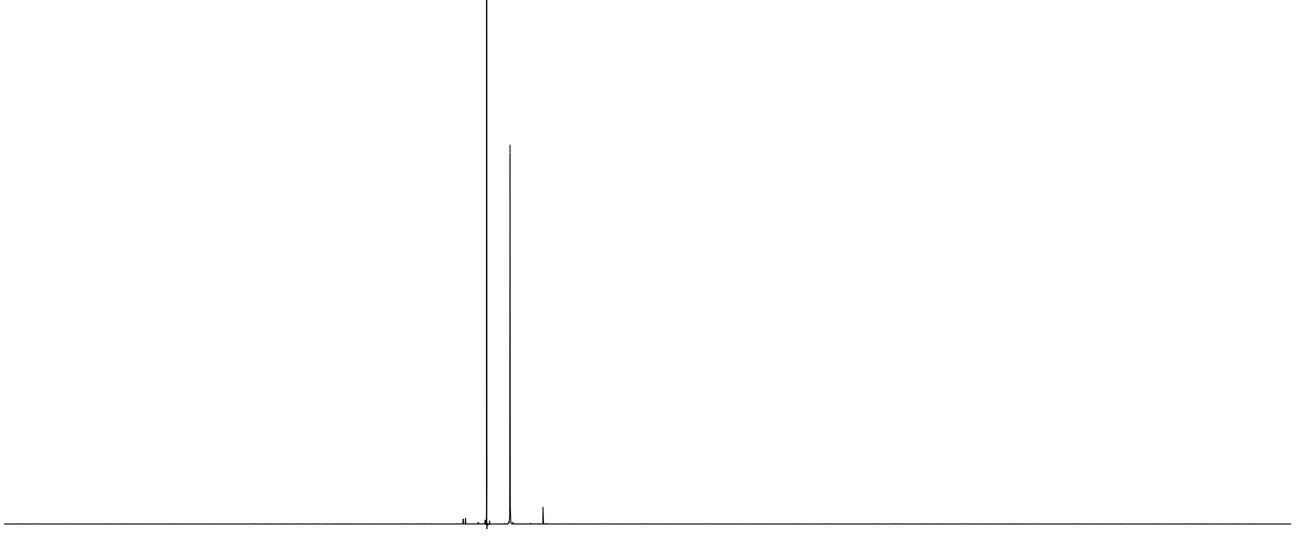

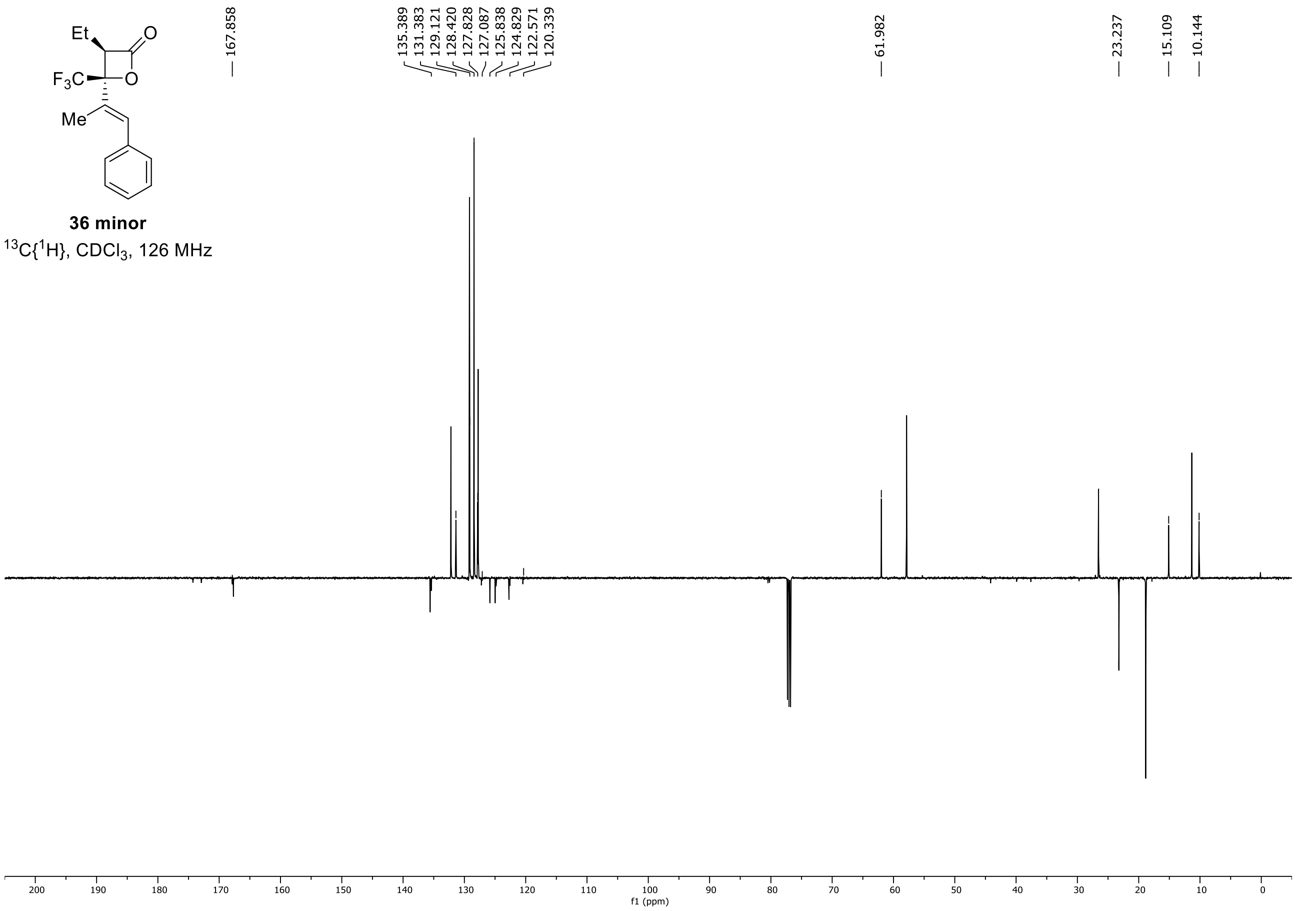

Et
O
F3C
O
Me
36 minor
13C{1H}, CDCl3, 126 MHz
167.858
135.389
131.383
129.121
128.420
127.828
127.087
125.838
124.829
122.571
120.339
61.982
23.237
15.109
10.144
f1 (ppm)

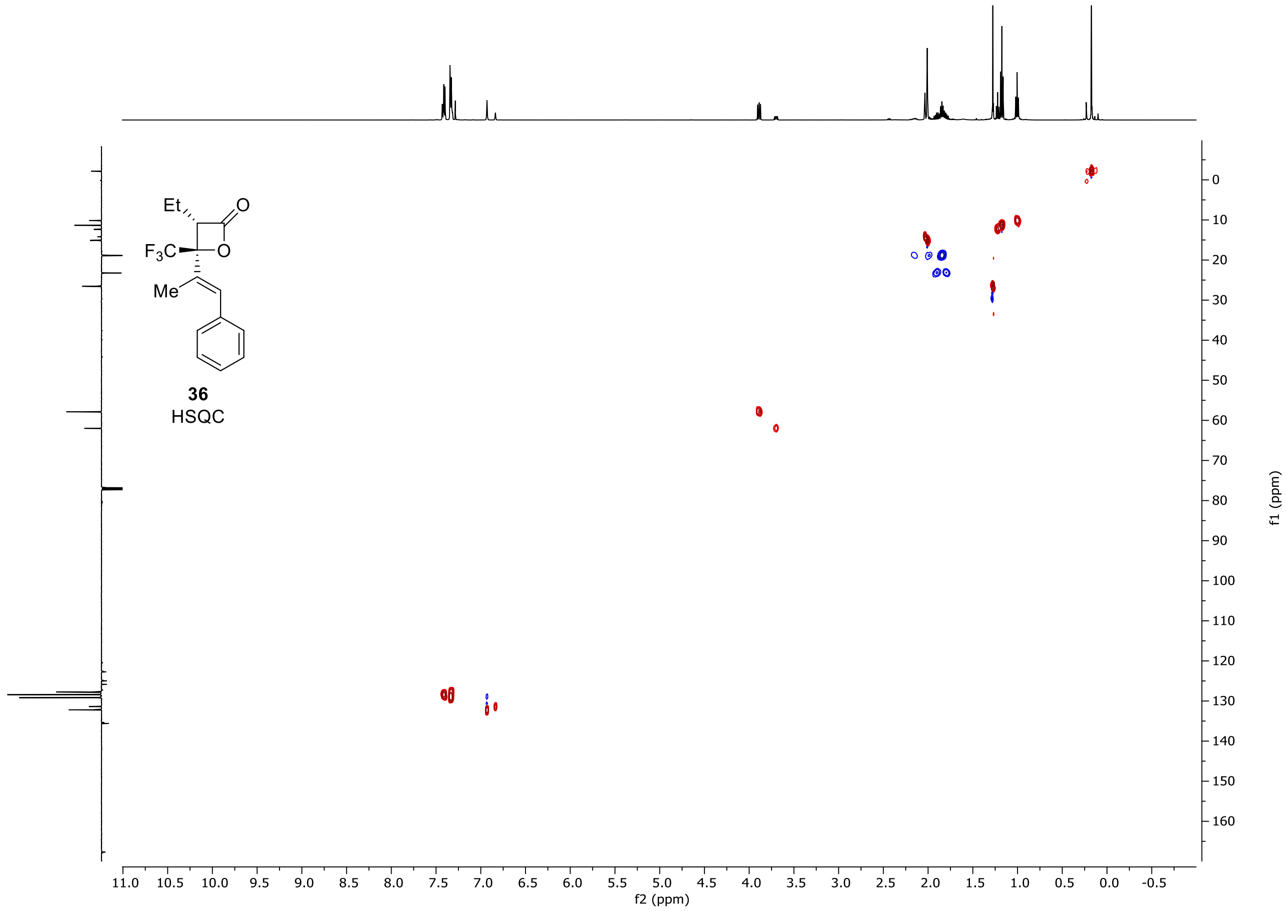

Et
O
F3C
O
Me
36
HSQC
f2 (ppm)
f1 (ppm)

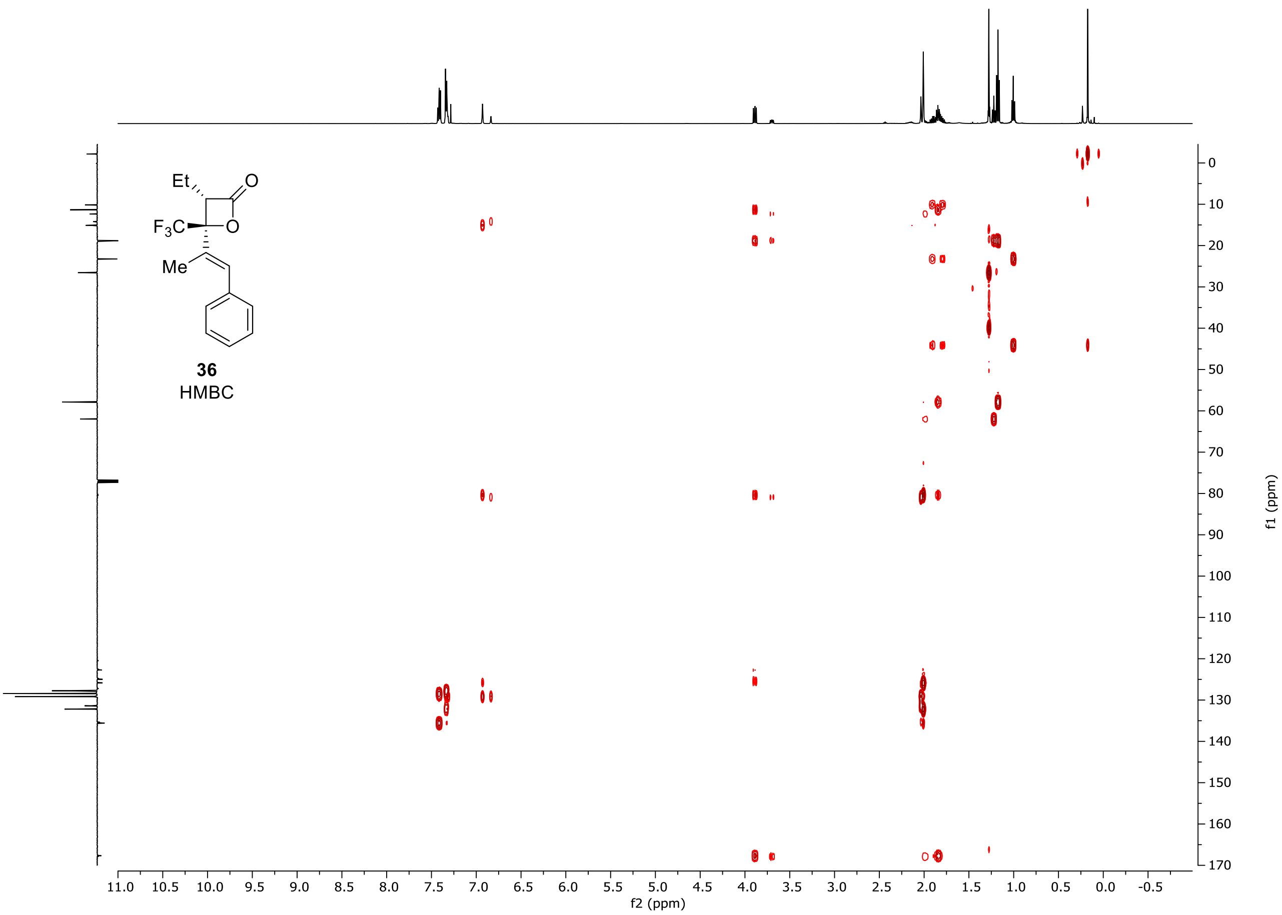

Et
O
F3C
O
Me
36
HMBC
f2 (ppm)
f1 (ppm)

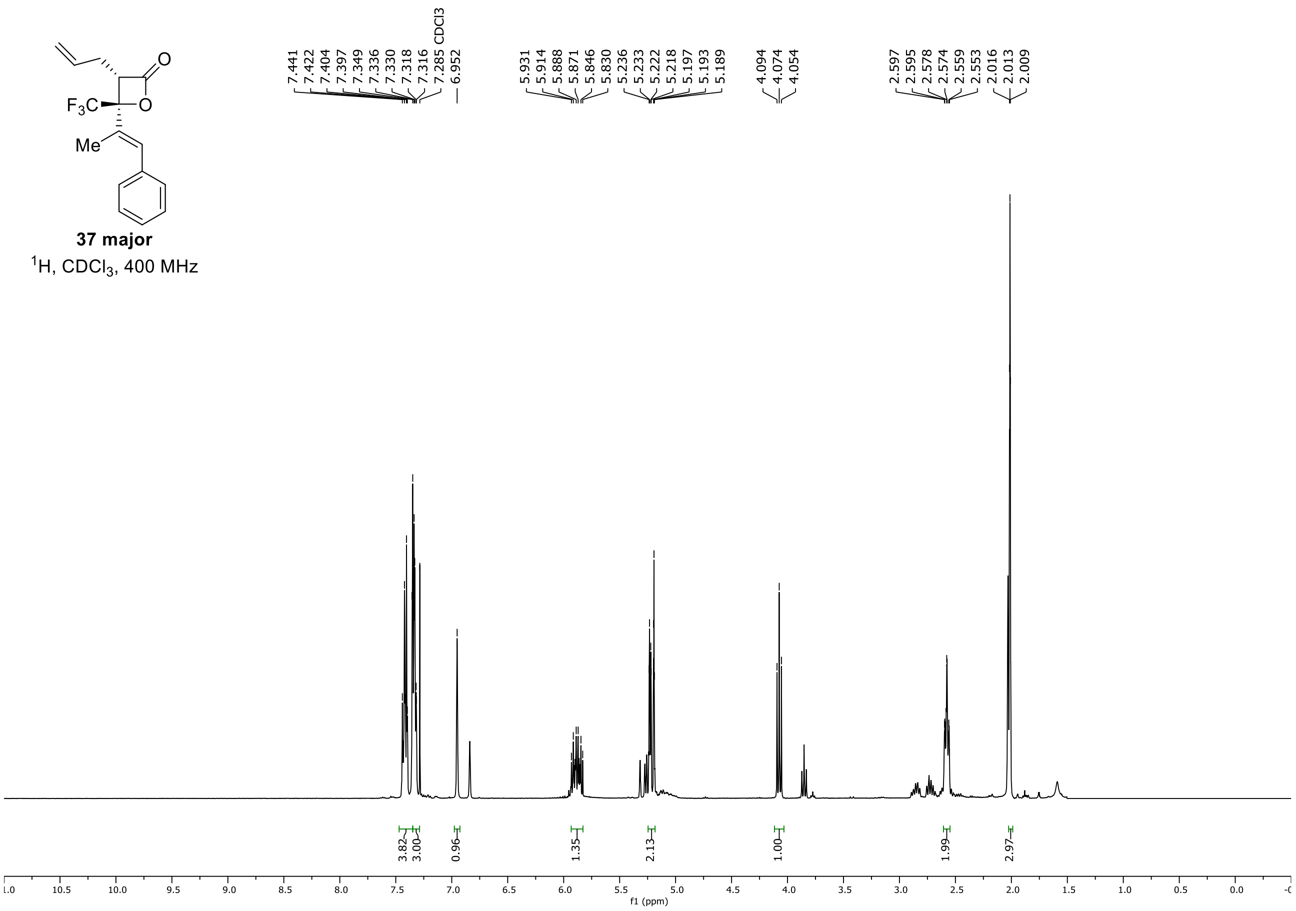

F3C
O
O
Me
37 major
1H, CDCl3, 400 MHz
7.441
7.422
7.404
7.397
7.349
7.336
7.330
7.318
7.316
7.285 CDCl3
6.952
5.931
5.914
5.888
5.871
5.846
5.830
5.236
5.233
5.222
5.218
5.197
5.193
5.189
4.094
4.074
4.054
2.597
2.595
2.578
2.574
2.559
2.553
2.016
2.013
2.009
3.82
3.00
0.96
1.35
2.13
1.00
1.99
2.97
f1 (ppm)

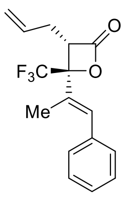

37 major
^{19}F, $CDCl_3$, 376 MHz

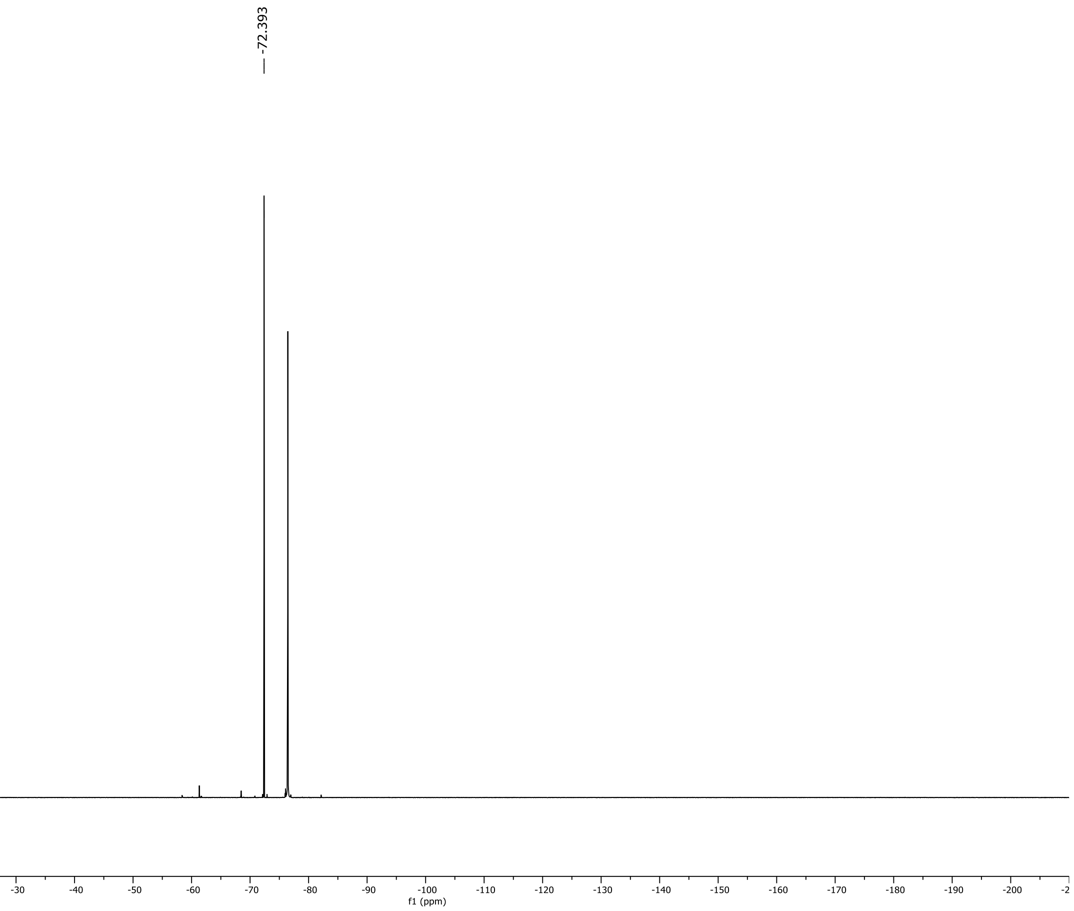

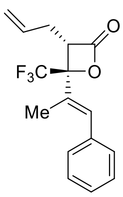

37 major
^{13}C{^{1}H}, $CDCl_3$, 126 MHz

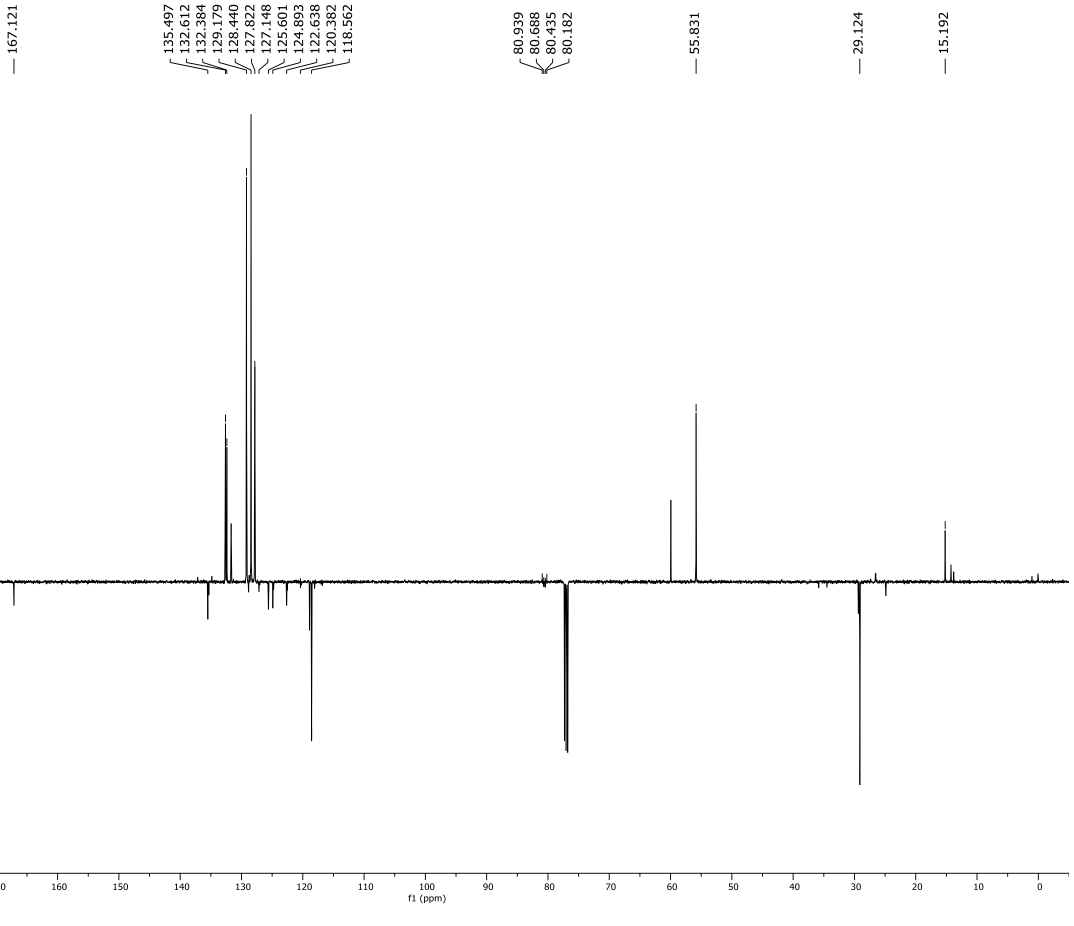

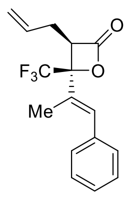

37 minor
^{1}H, $CDCl_3$, 400 MHz

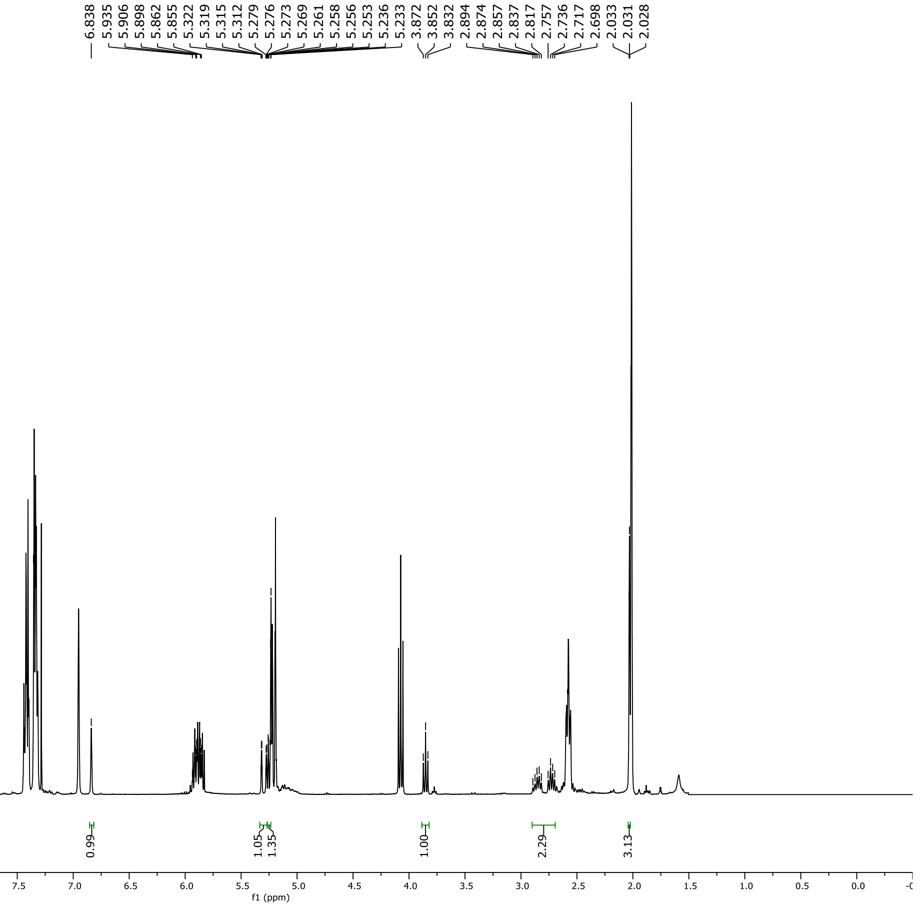

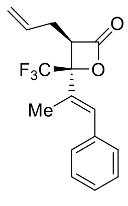

37 minor
^{19}F, $CDCl_3$, 376 MHz

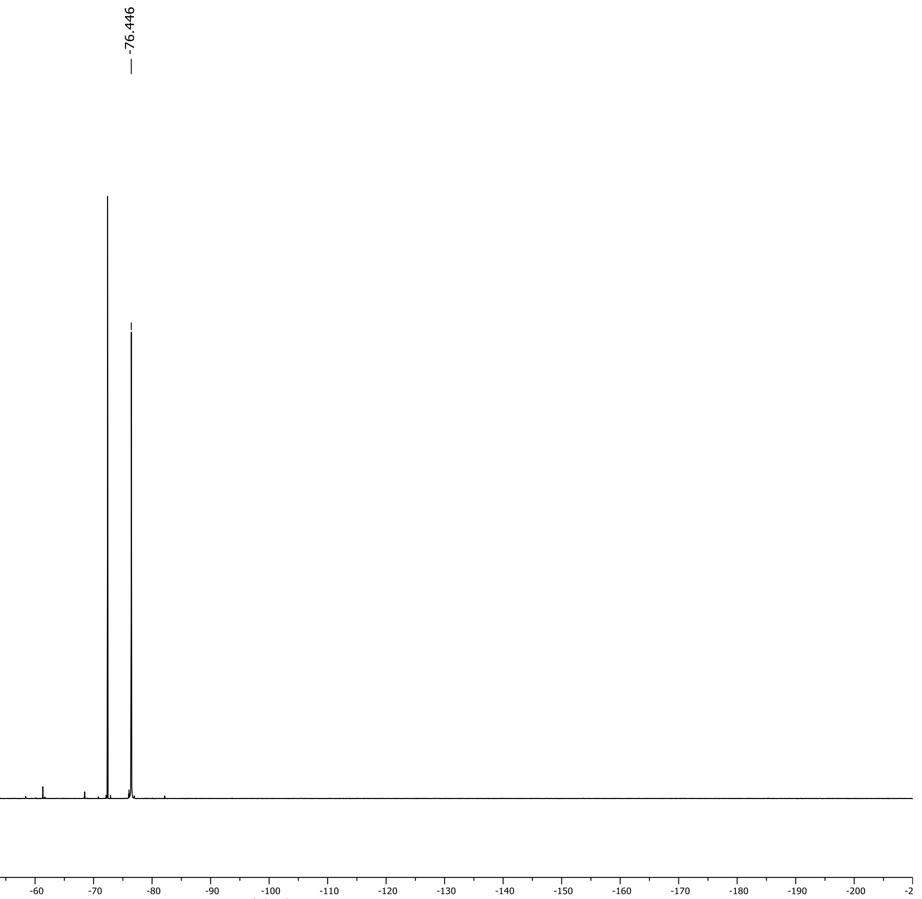

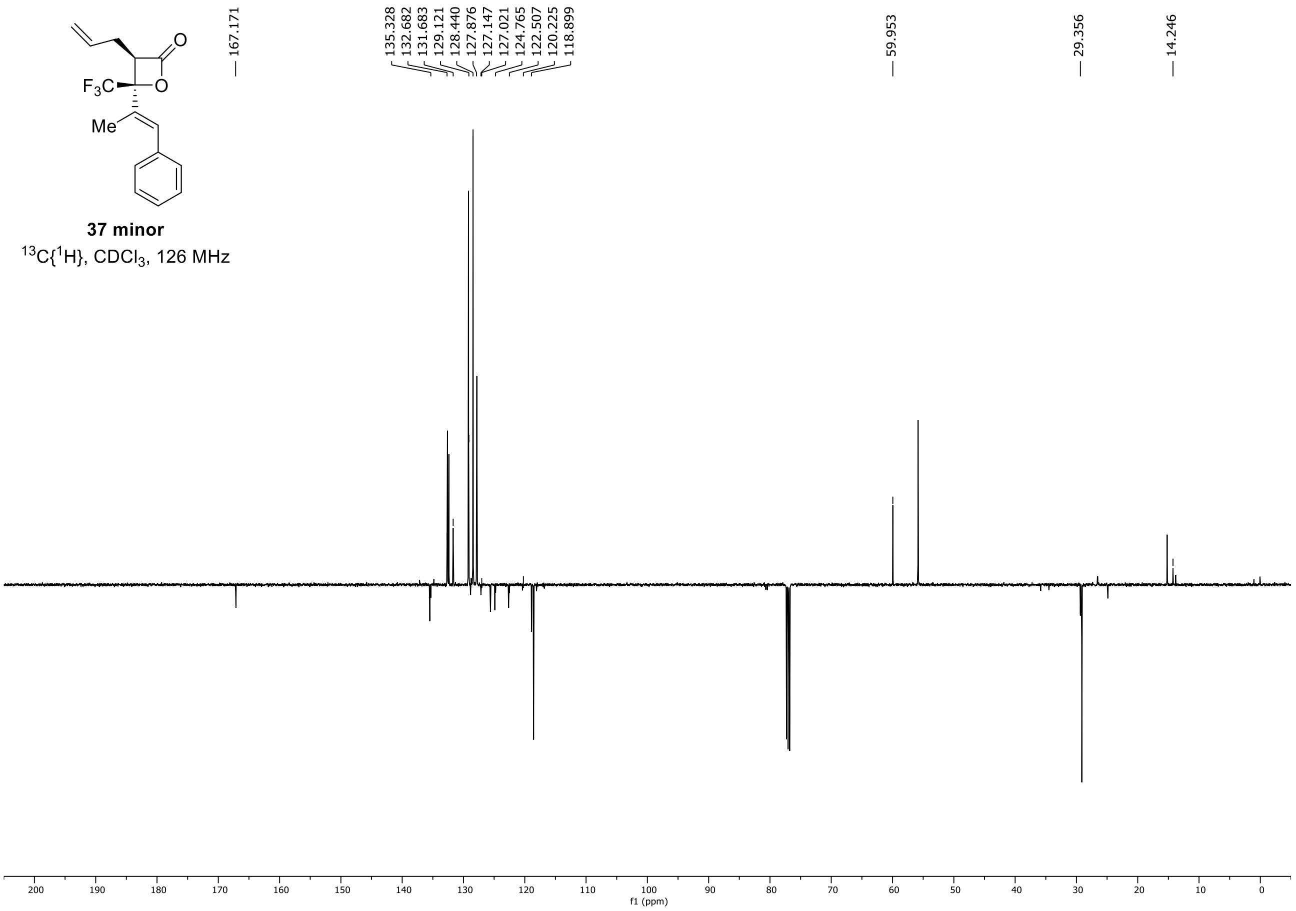
37 minor
$^{13}C\{^{1}H\}$, $CDCl_3$, 126 MHz
167.171
135.328
132.682
131.683
129.121
128.440
127.876
127.147
127.021
124.765
122.507
120.225
118.899
59.953
29.356
14.246
f1 (ppm)

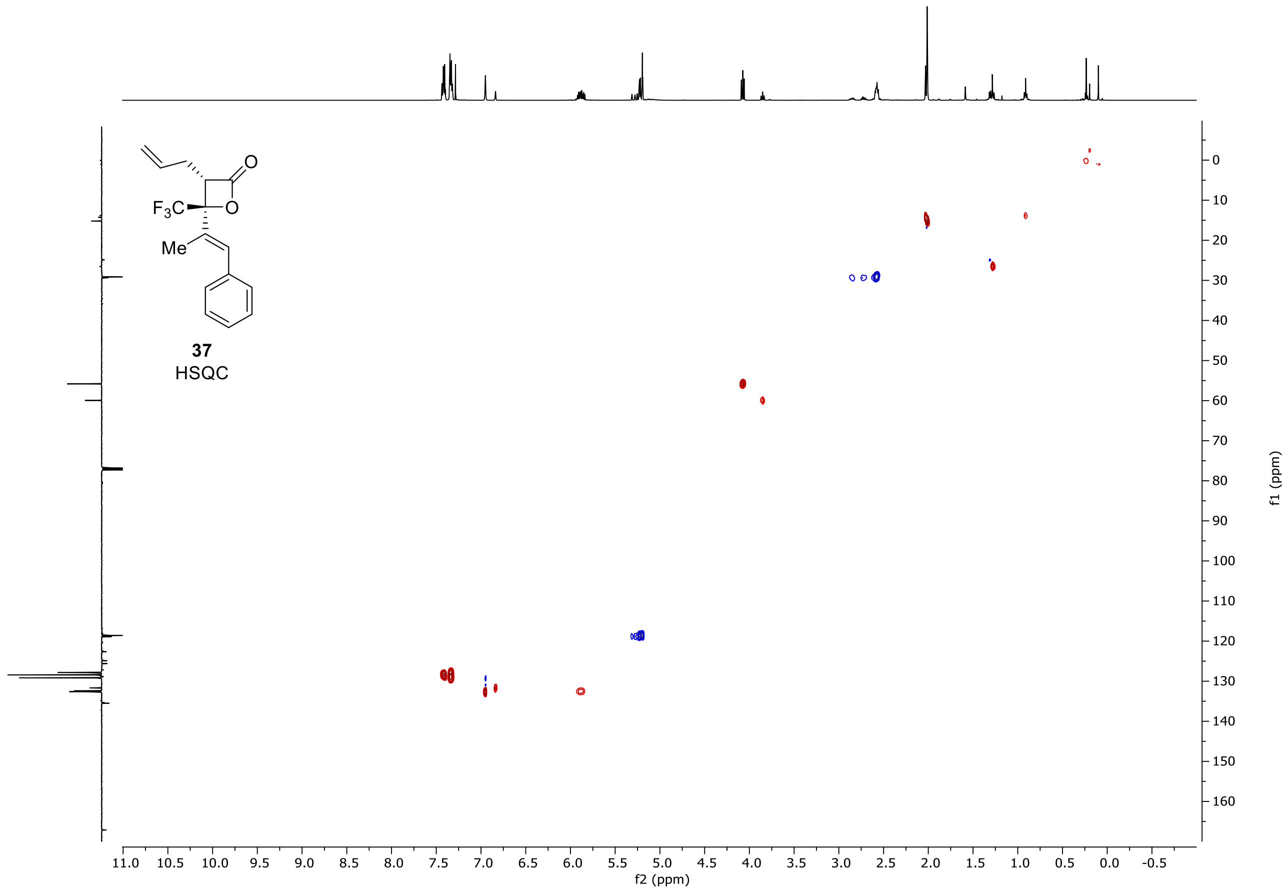
37
HSQC
f2 (ppm)
f1 (ppm)

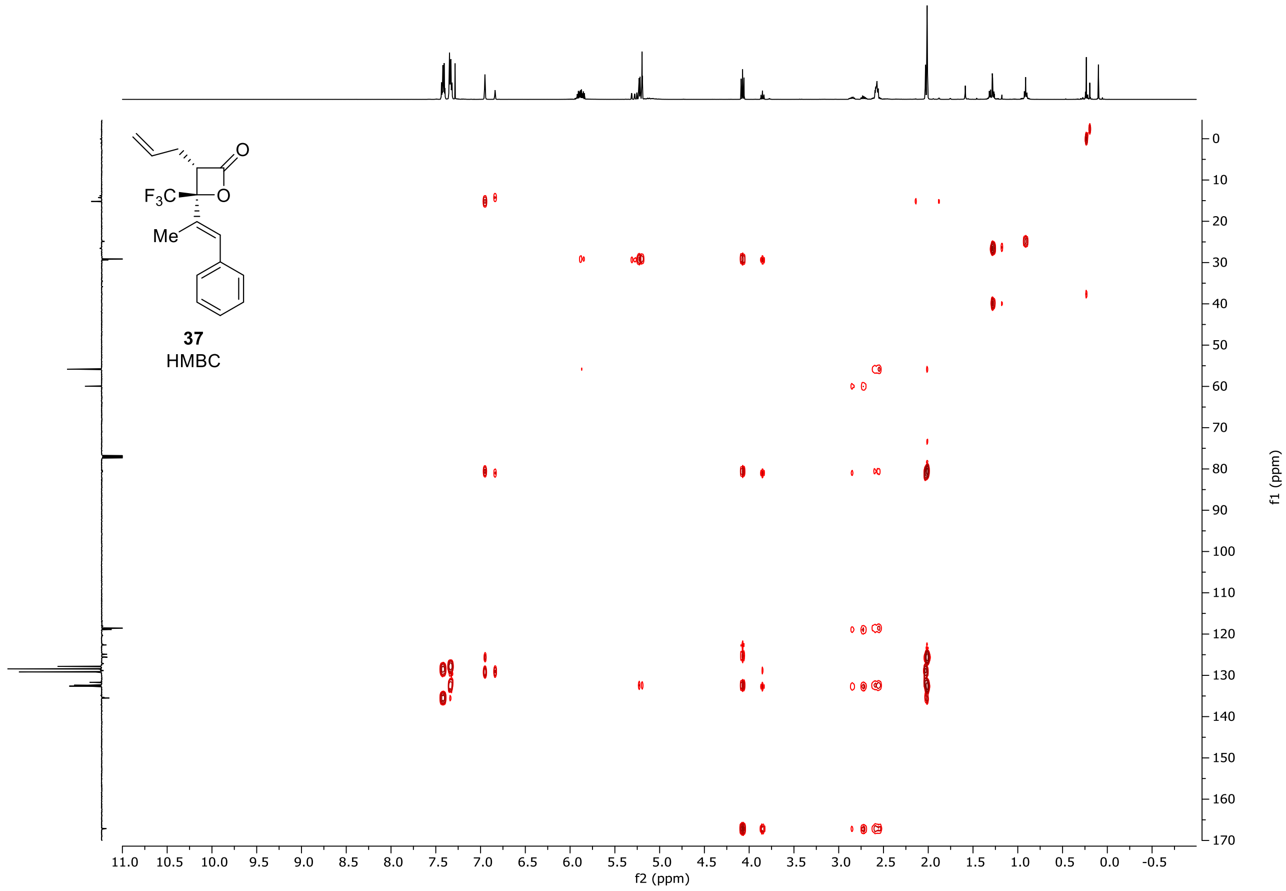
F3C
O
O
Me
37
HMBC
f2 (ppm)
f1 (ppm)

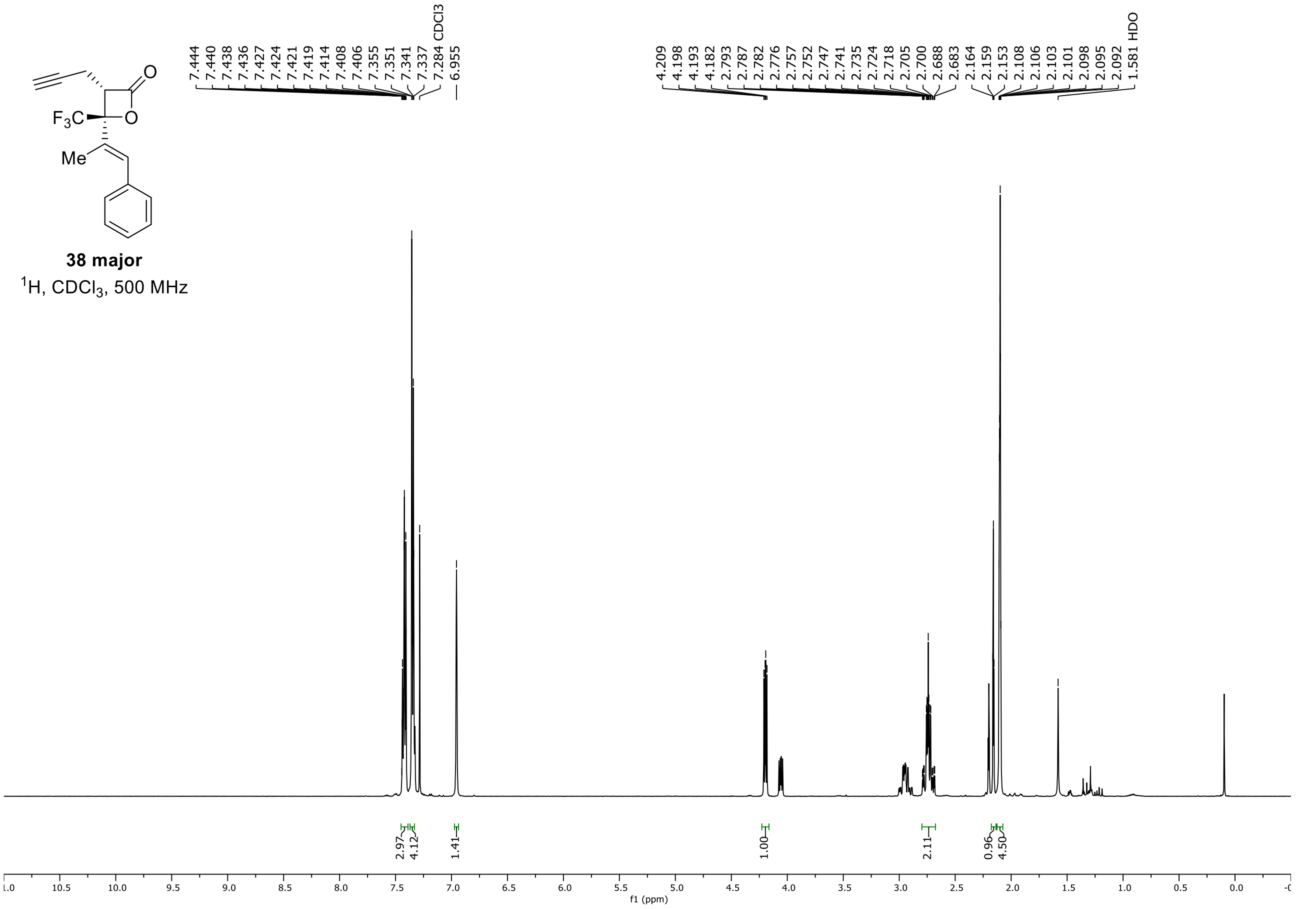
7.444
7.440
7.438
7.436
7.427
7.424
7.421
7.419
7.414
7.408
7.406
7.355
7.351
7.341
7.337
7.284 CDCl3
6.955
4.209
4.198
4.193
4.182
2.793
2.787
2.782
2.776
2.757
2.752
2.747
2.741
2.735
2.724
2.718
2.705
2.700
2.688
2.683
2.164
2.159
2.153
2.108
2.106
2.103
2.101
2.098
2.095
2.092
1.581 HDO
F3C
O
O
Me
38 major
1H, CDCl3, 500 MHz
2.97
4.12
1.41
1.00
2.11
0.96
4.50
f1 (ppm)

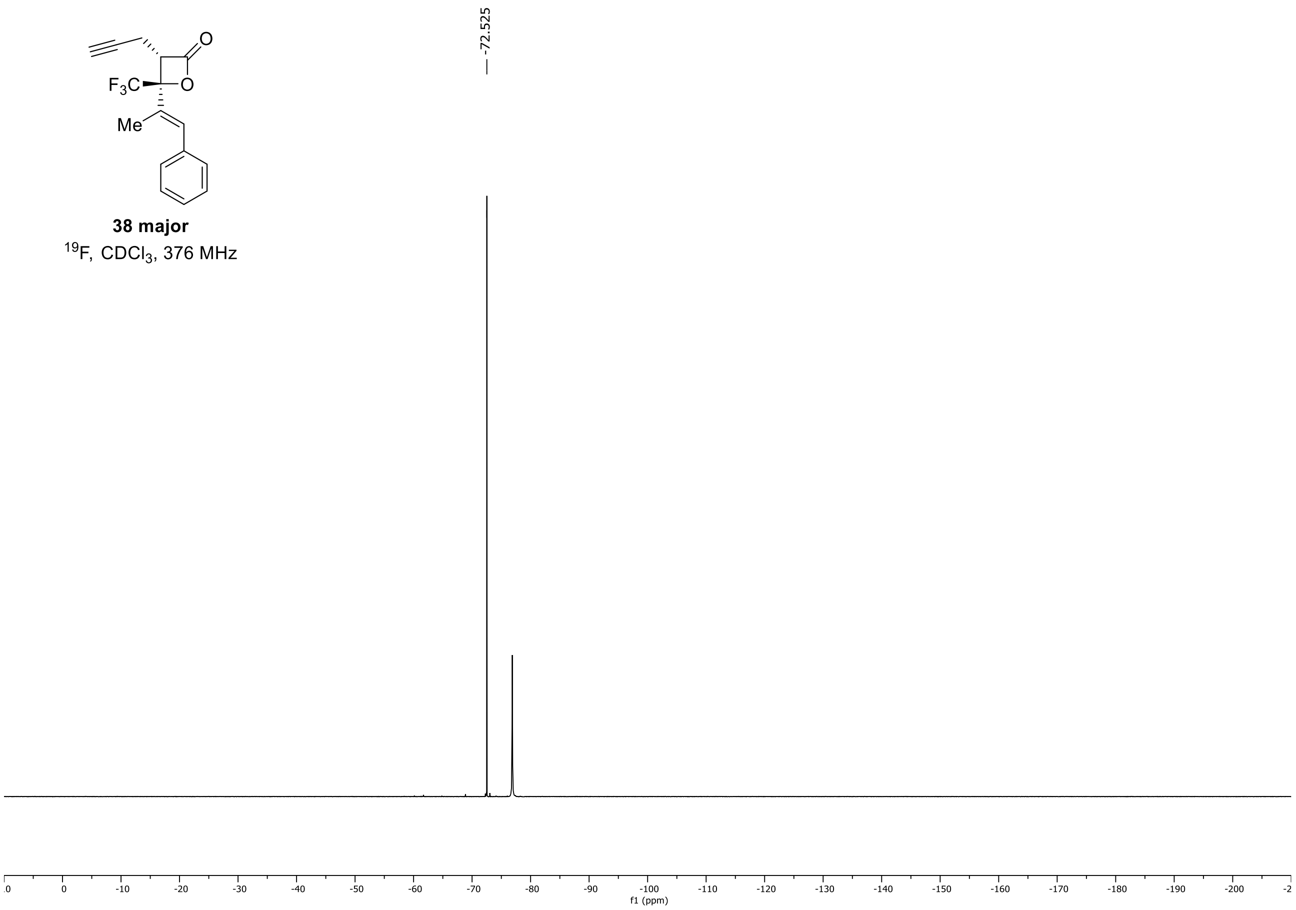

38 major
^{19}F, CDCl$_3$, 376 MHz
-72.525
f1 (ppm)

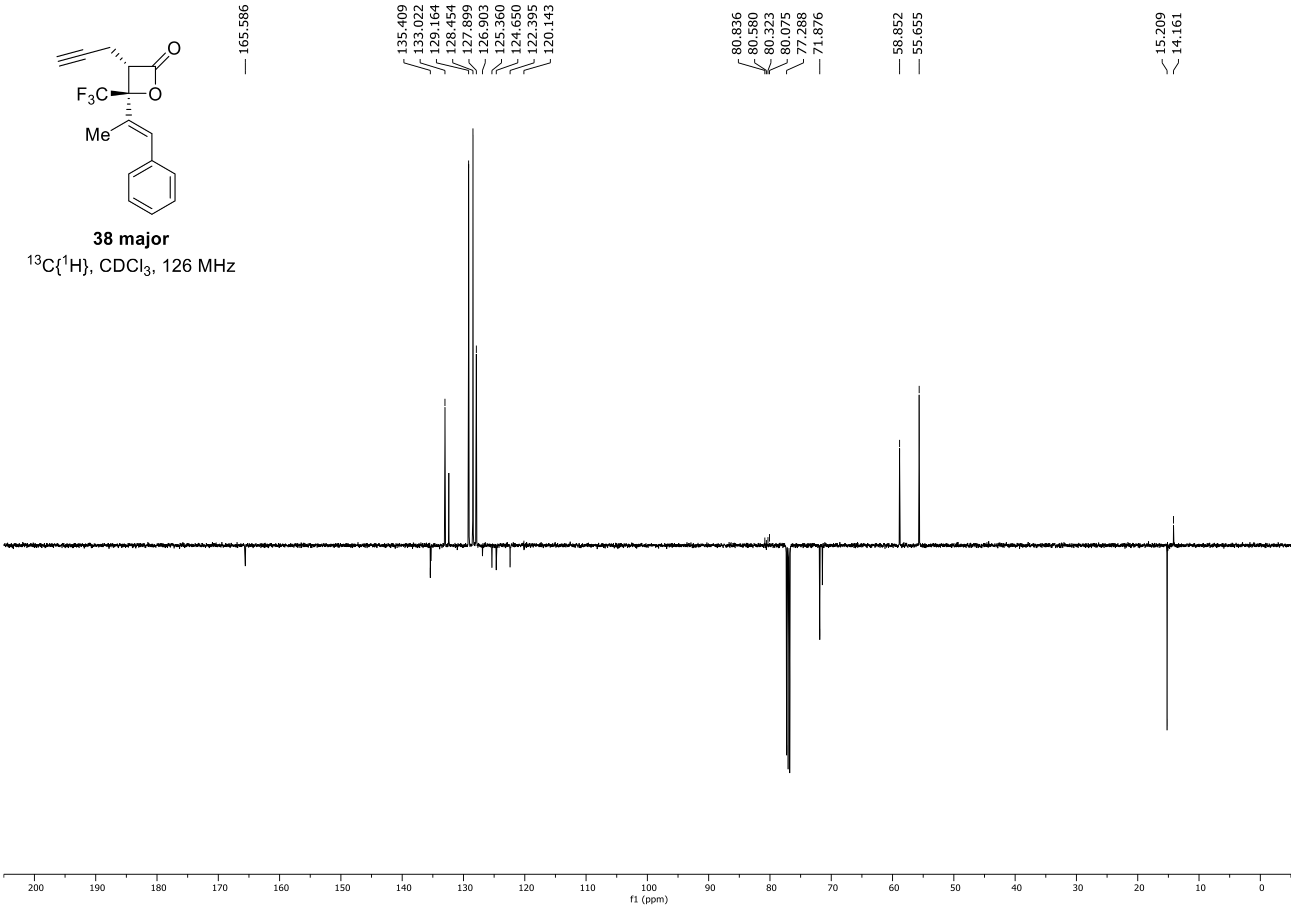

38 major
^{13}C{^{1}H}, CDCl$_3$, 126 MHz
165.586
135.409
133.022
129.164
128.454
127.899
126.903
125.360
124.650
122.395
120.143
80.836
80.580
80.323
80.075
77.288
71.876
58.852
55.655
15.209
14.161
f1 (ppm)

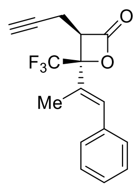

38 minor
^{1}H, $CDCl_3$, 500 MHz

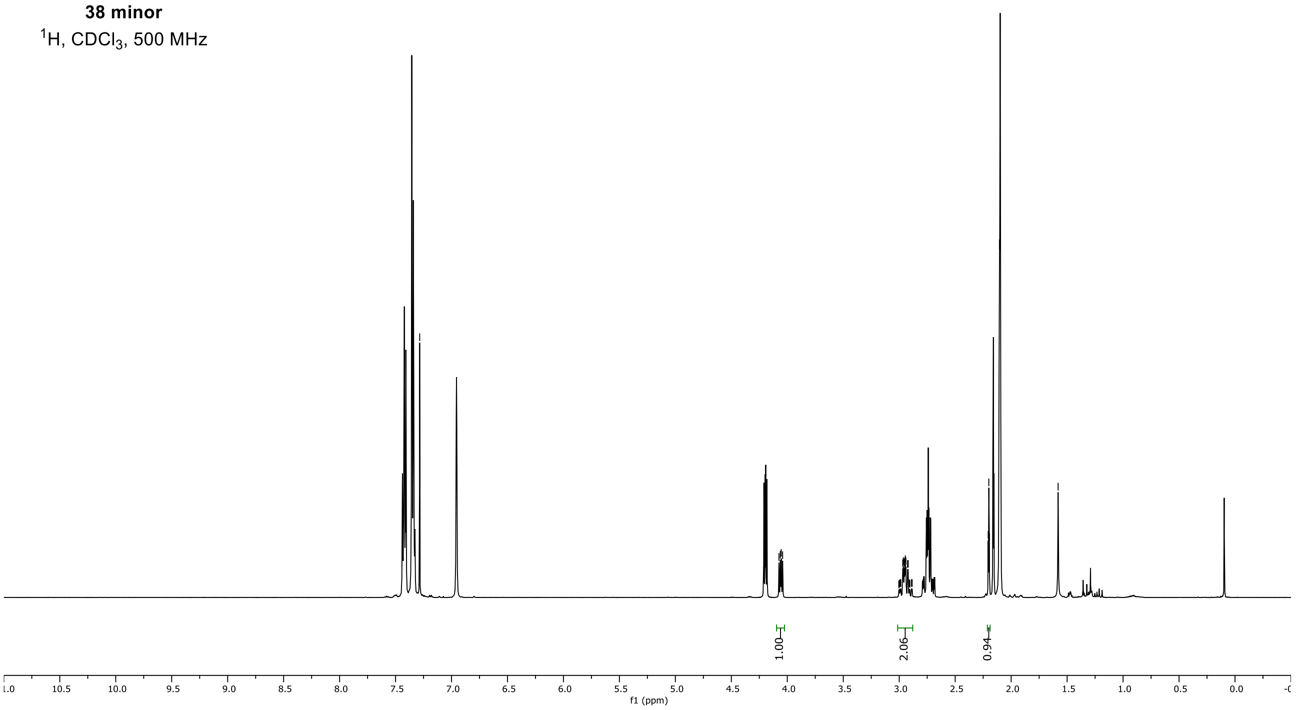

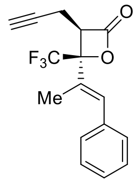

38 minor
^{19}F, $CDCl_3$, 376 MHz

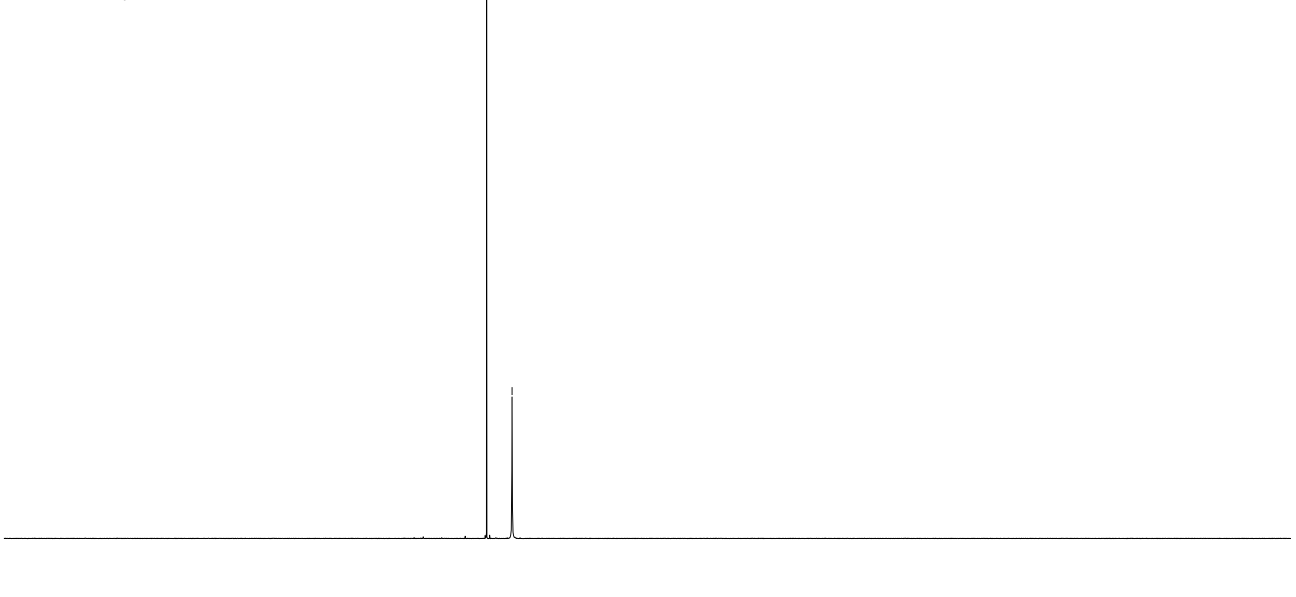

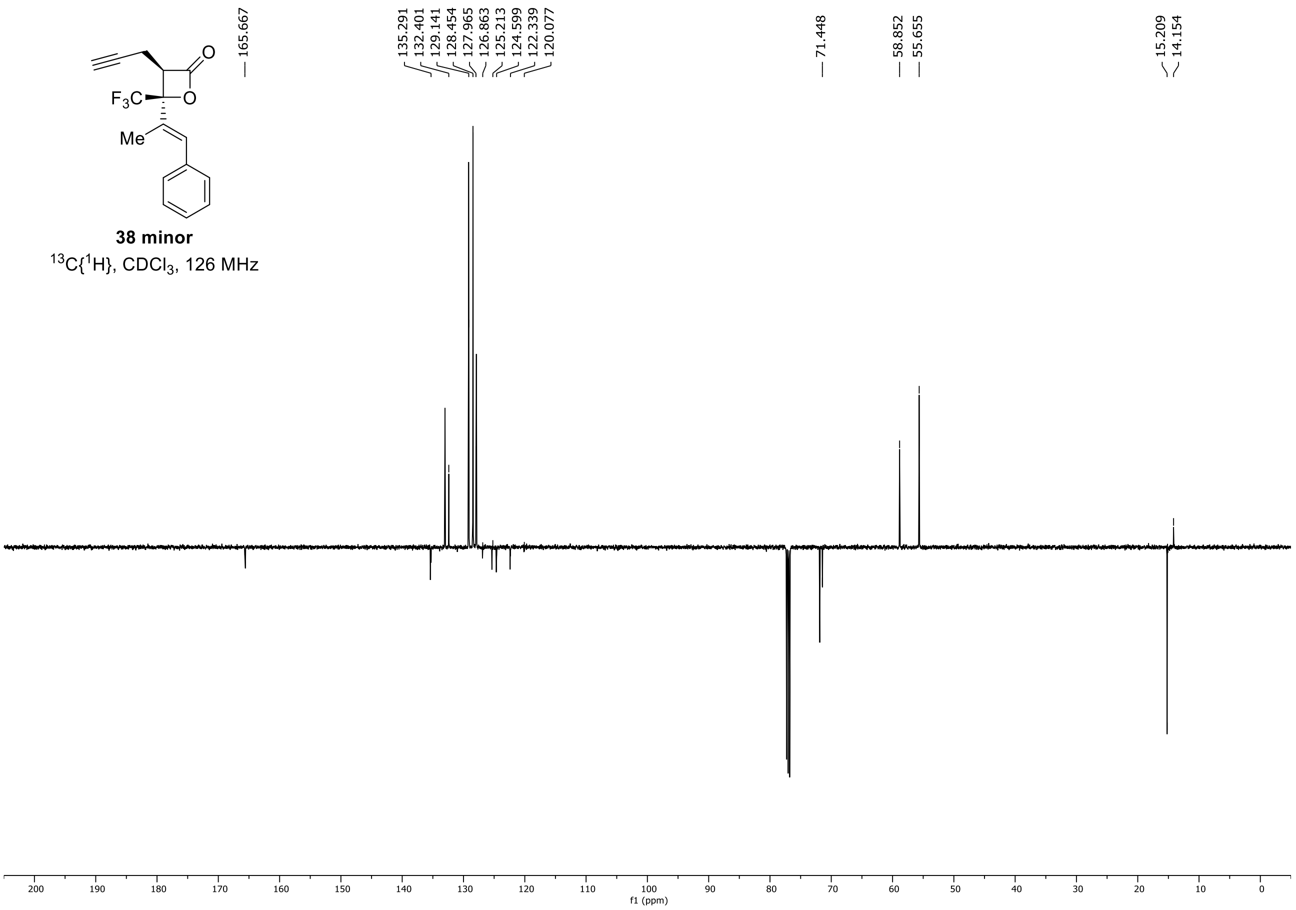
F3C
O
O
Me
38 minor
13C{1H}, CDCl3, 126 MHz
165.667
135.291
132.401
129.141
128.454
127.965
126.863
125.213
124.599
122.339
120.077
71.448
58.852
55.655
15.209
14.154
f1 (ppm)

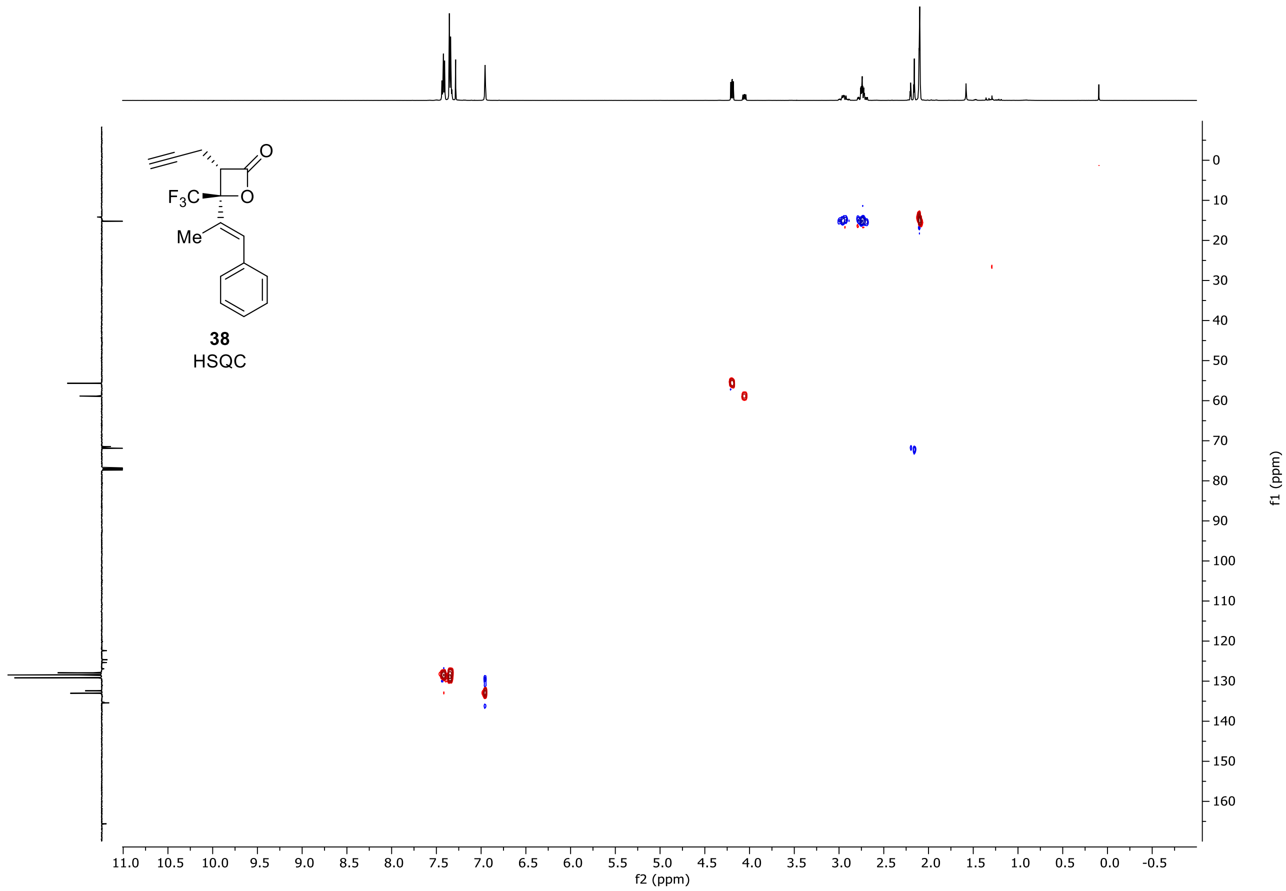
F3C
O
O
Me
38
HSQC
f2 (ppm)
f1 (ppm)

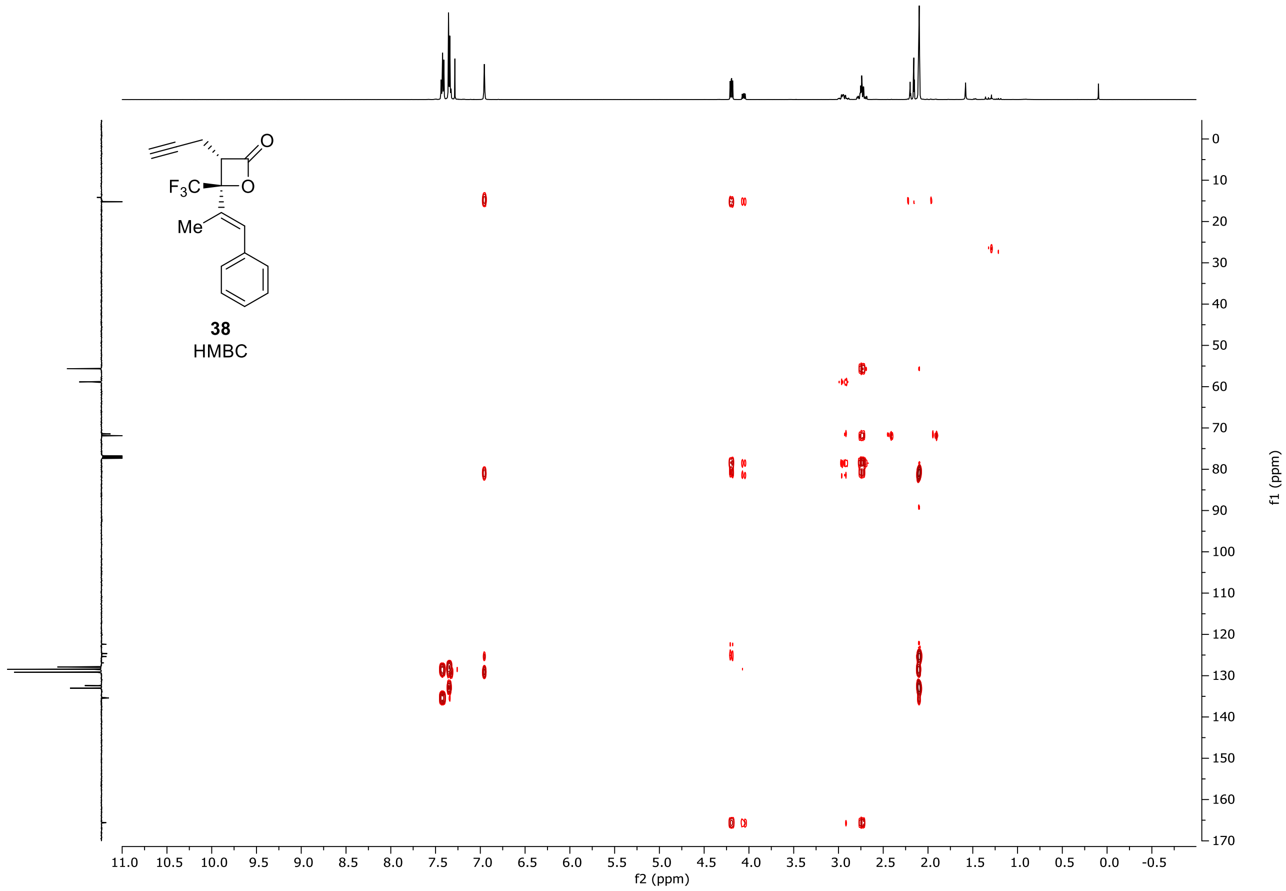

F3C
O
O
Me
38
HMBC
f2 (ppm)
f1 (ppm)

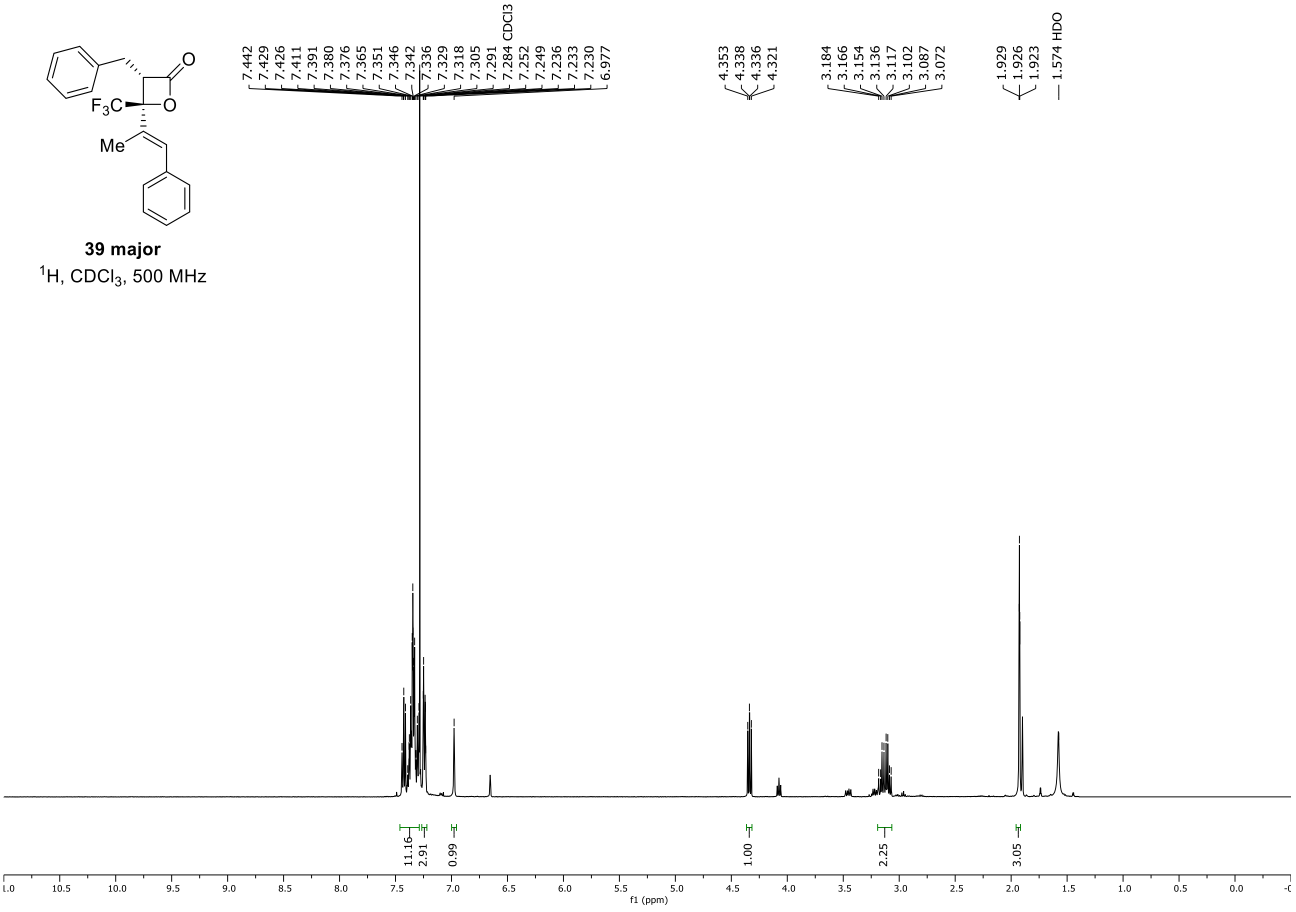

F3C
O
O
Me
39 major
1H, CDCl3, 500 MHz
7.442 7.429 7.426 7.411 7.391 7.380 7.376 7.365 7.351 7.346 7.342 7.336 7.329 7.318 7.305 7.291 7.284 CDCl3 7.252 7.249 7.236 7.233 7.230 6.977
4.353 4.338 4.336 4.321
3.184 3.166 3.154 3.136 3.117 3.102 3.087 3.072
1.929 1.926 1.923
1.574 HDO
11.16 2.91 0.99 1.00 2.25 3.05
f1 (ppm)

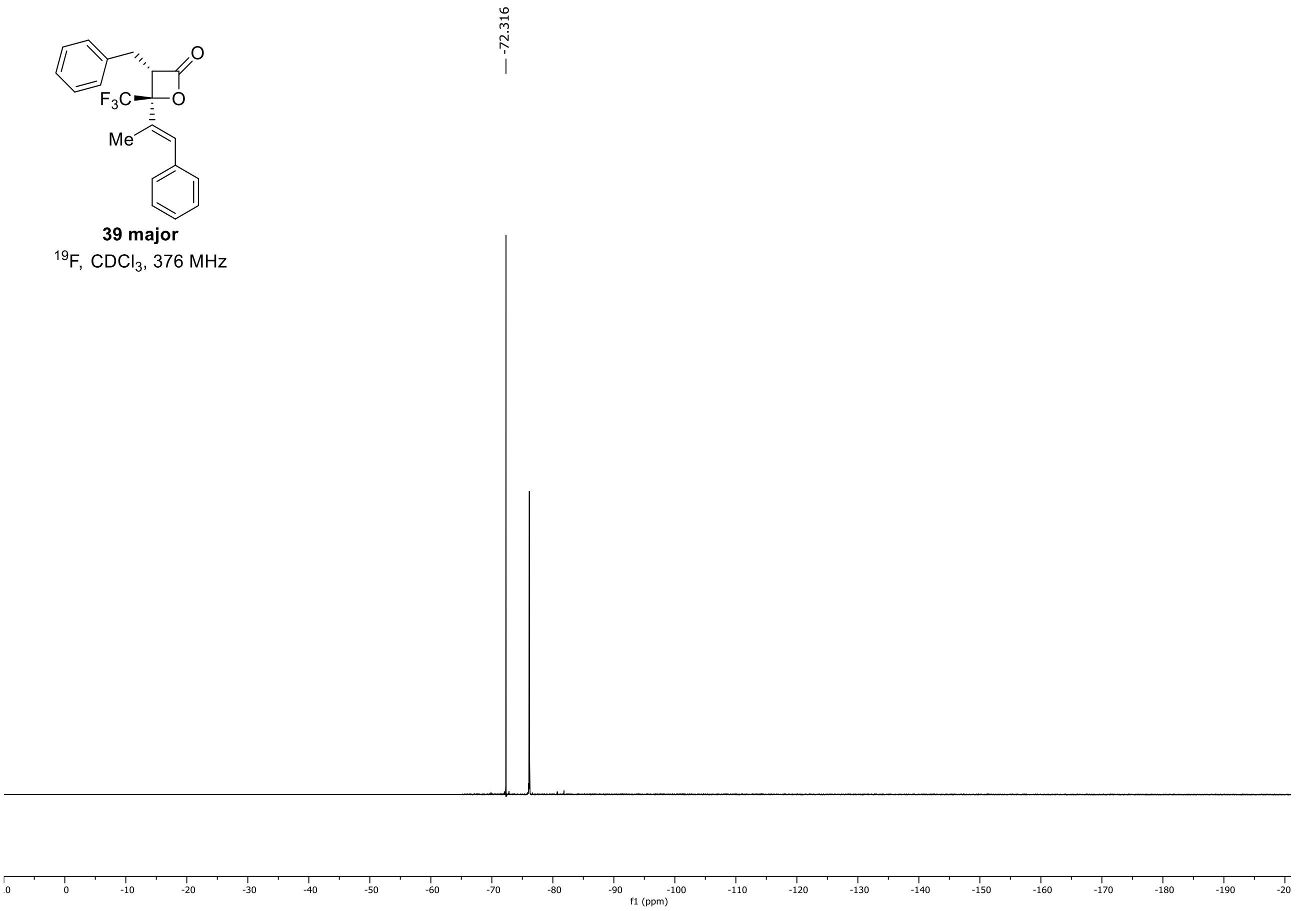

39 major
^{19}F, CDCl$_3$, 376 MHz

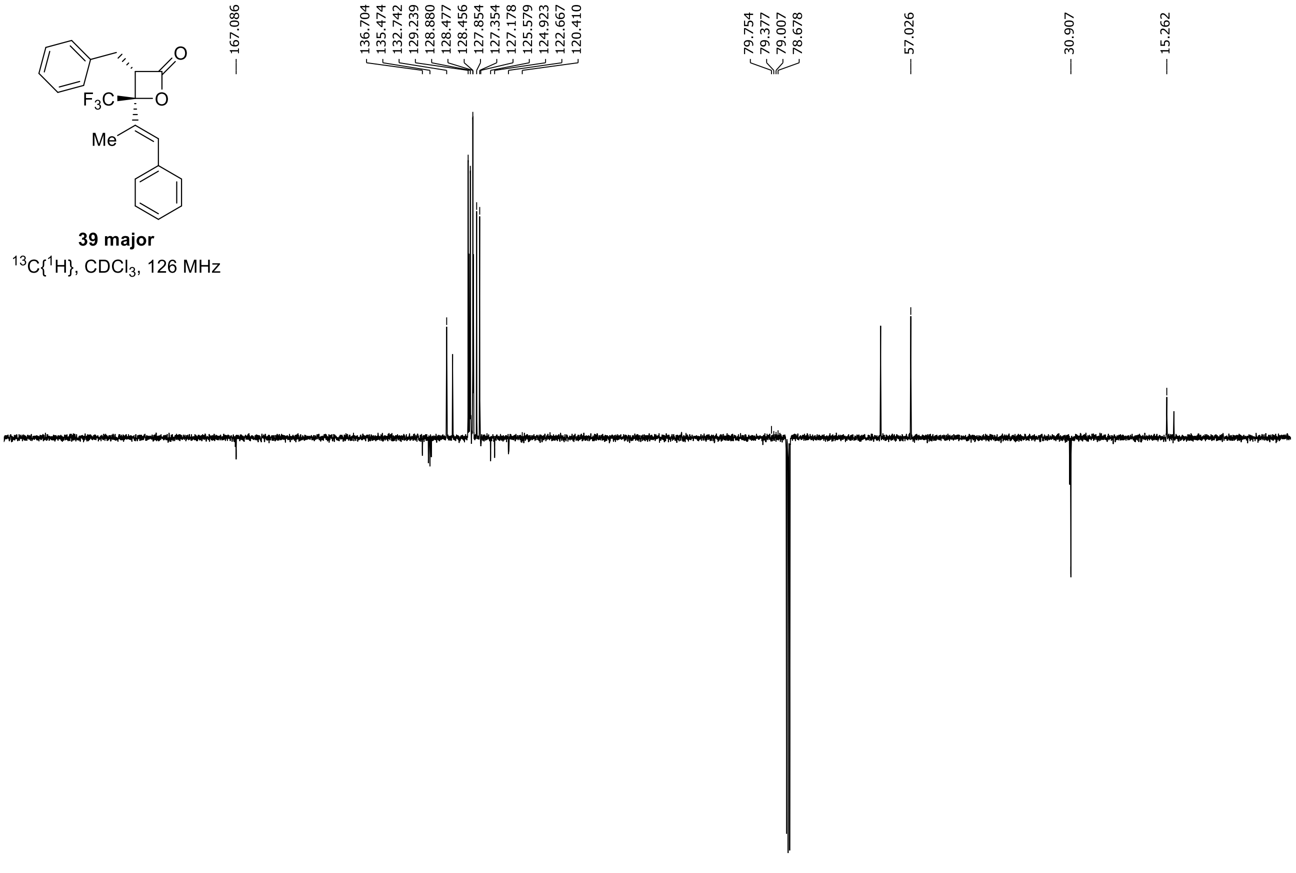

39 major
^{13}C{^{1}H}, CDCl$_3$, 126 MHz

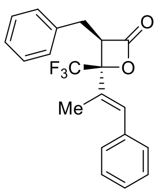

39 minor

^{1}H, $CDCl_3$, 500 MHz

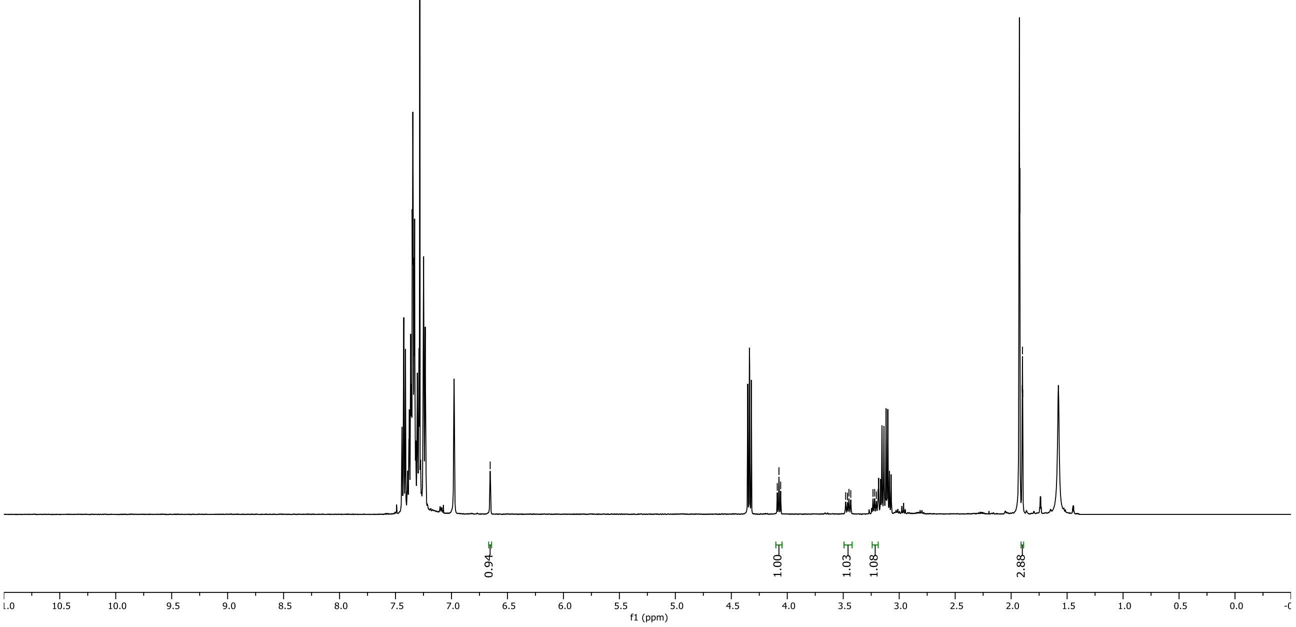

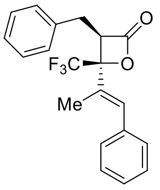

39 minor

^{19}F, $CDCl_3$, 376 MHz

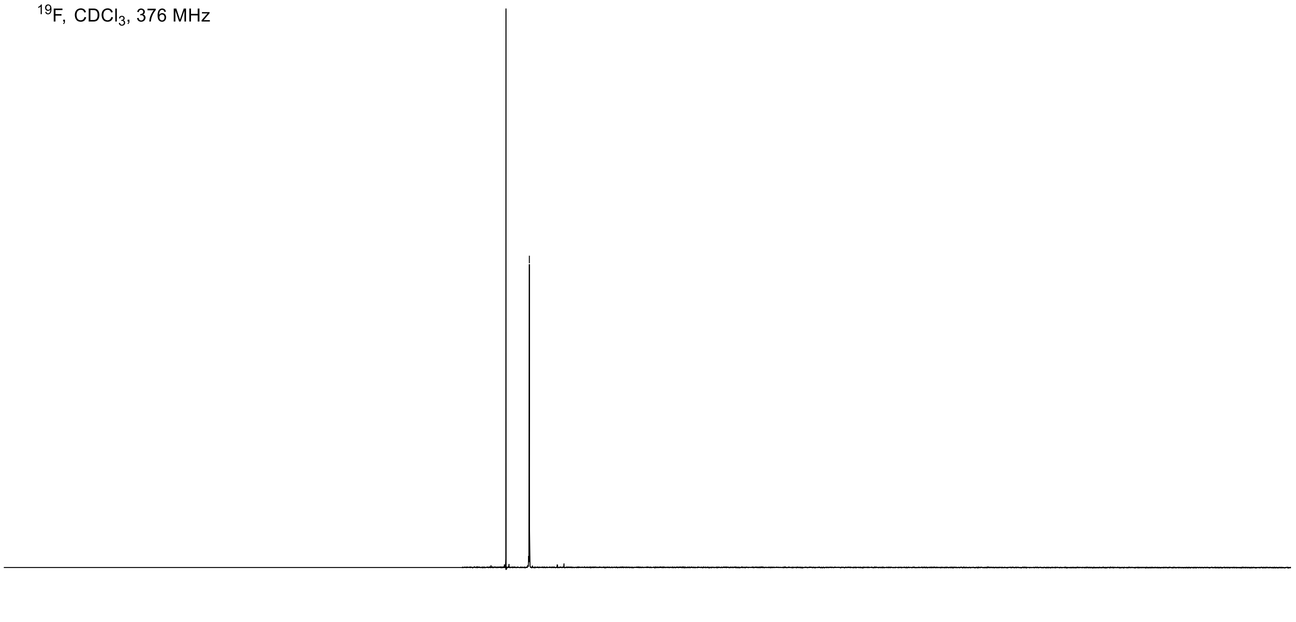

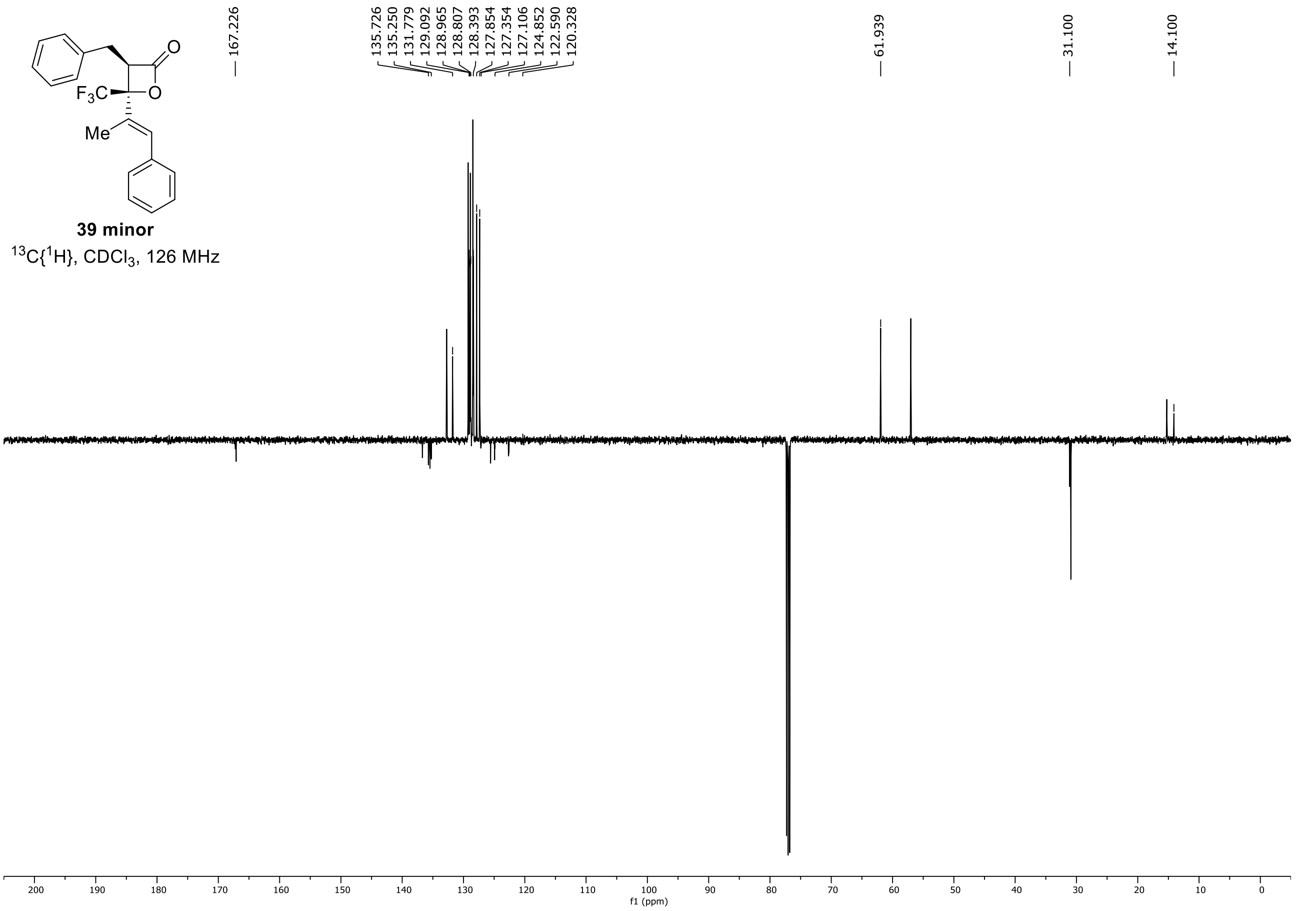
39 minor
$^{13}C\{^{1}H\}$, $CDCl_3$, 126 MHz
167.226
135.726
135.250
131.779
129.092
128.965
128.807
128.393
127.854
127.354
127.106
124.852
122.590
120.328
61.939
31.100
14.100
f1 (ppm)

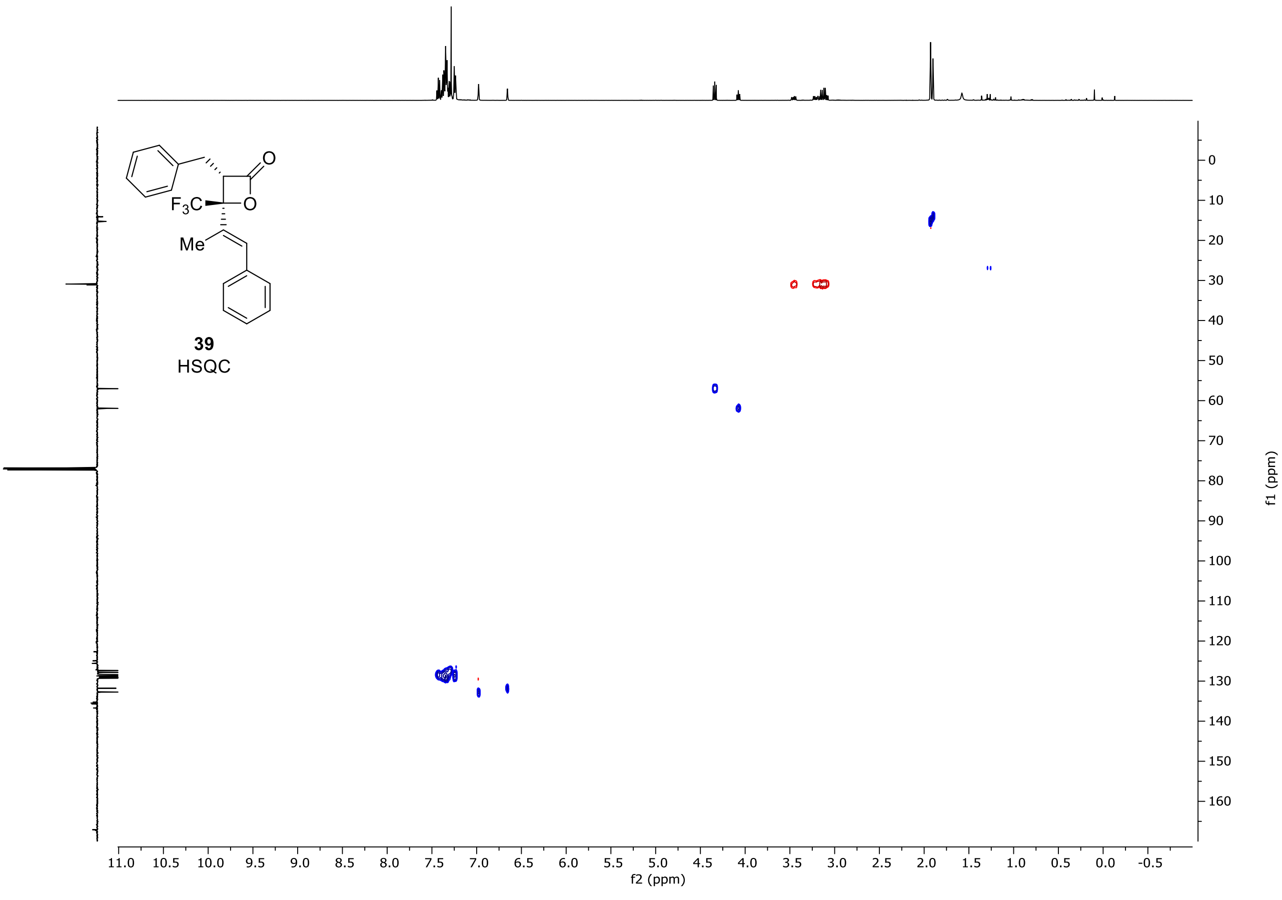
39
HSQC
f2 (ppm)
f1 (ppm)

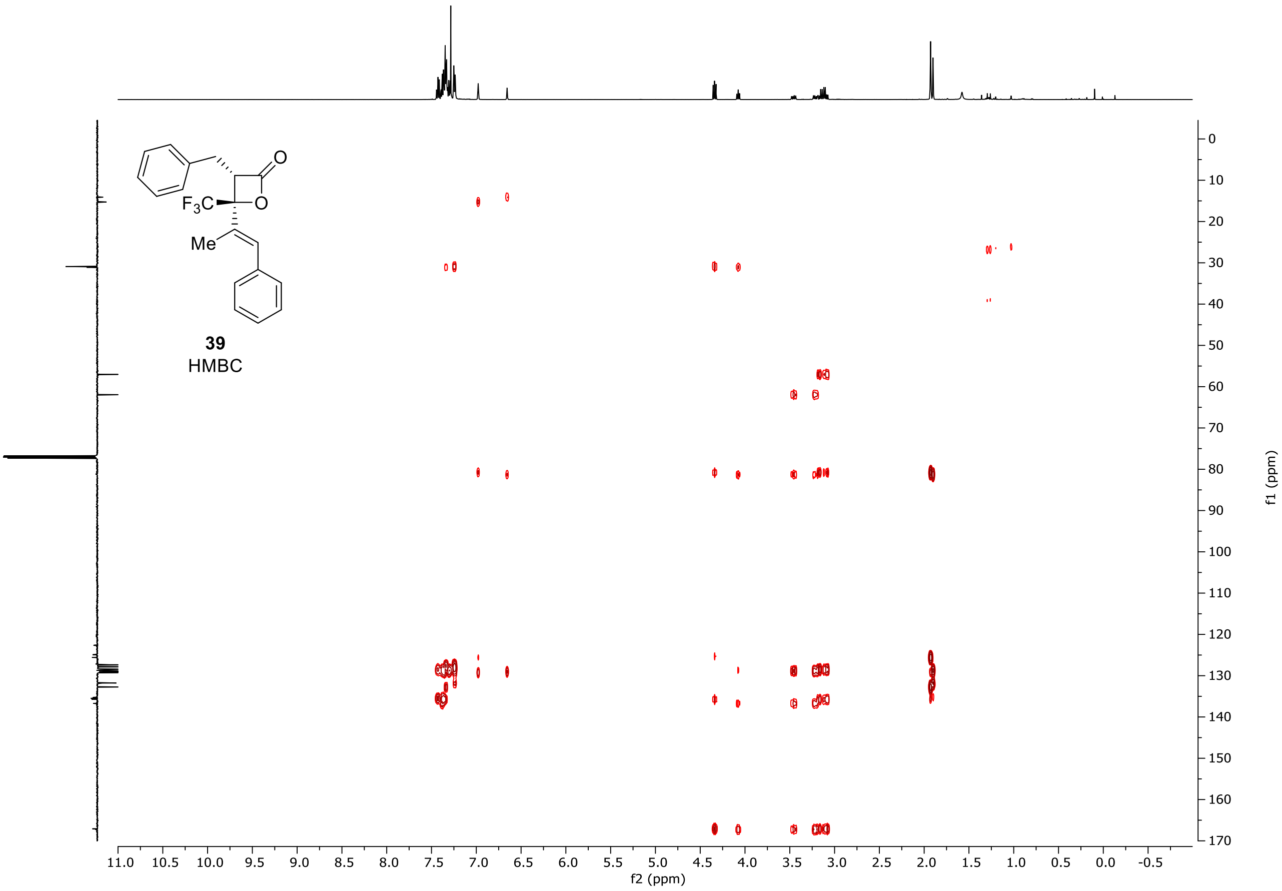

F3C
O
Me
39
HMBC
f2 (ppm)
f1 (ppm)

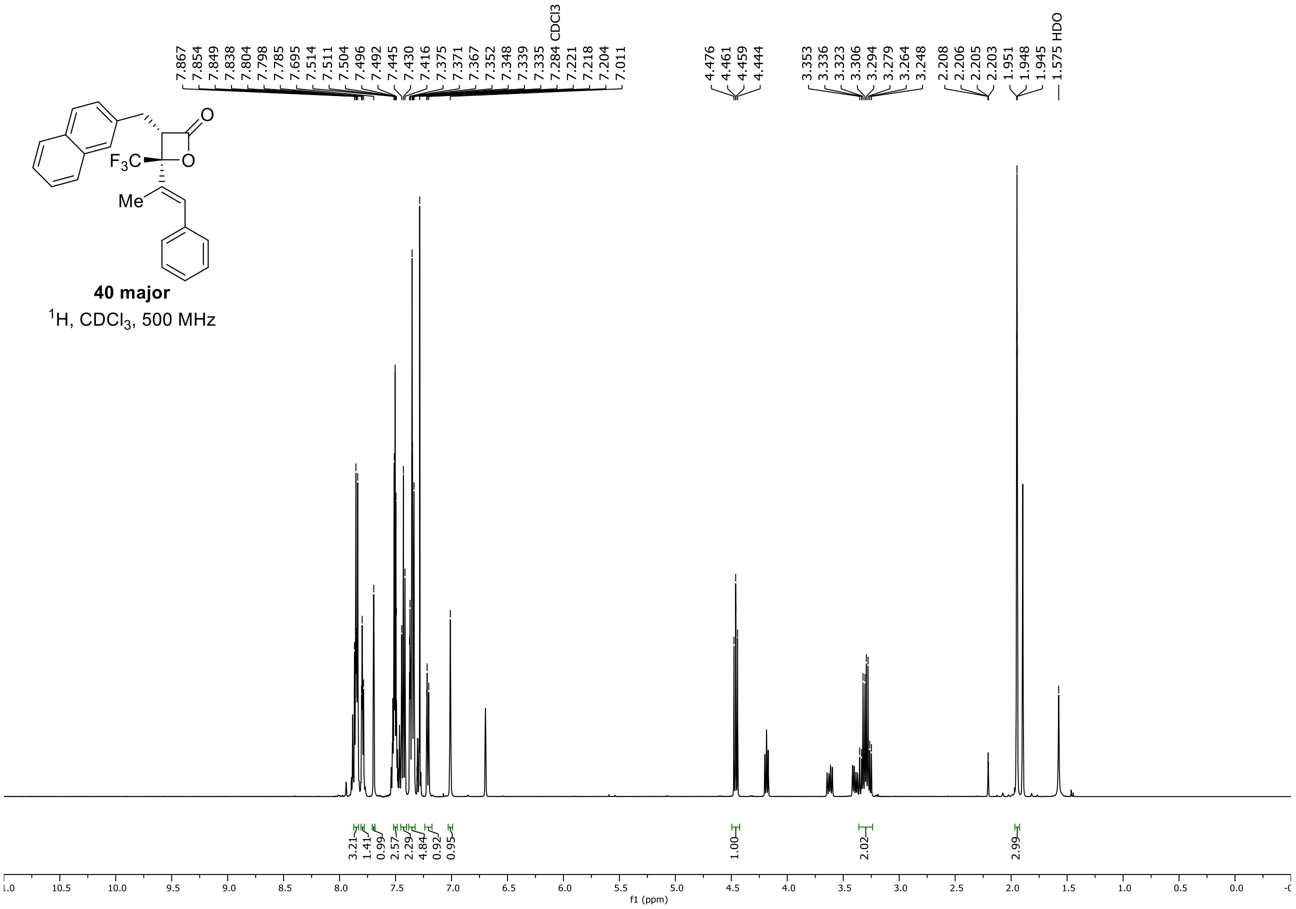

F3C
O
Me
40 major
1H, CDCl3, 500 MHz
CDCl3
1.575 HDO
f1 (ppm)

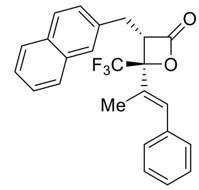

40 major

^{19}F, $CDCl_3$, 376 MHz

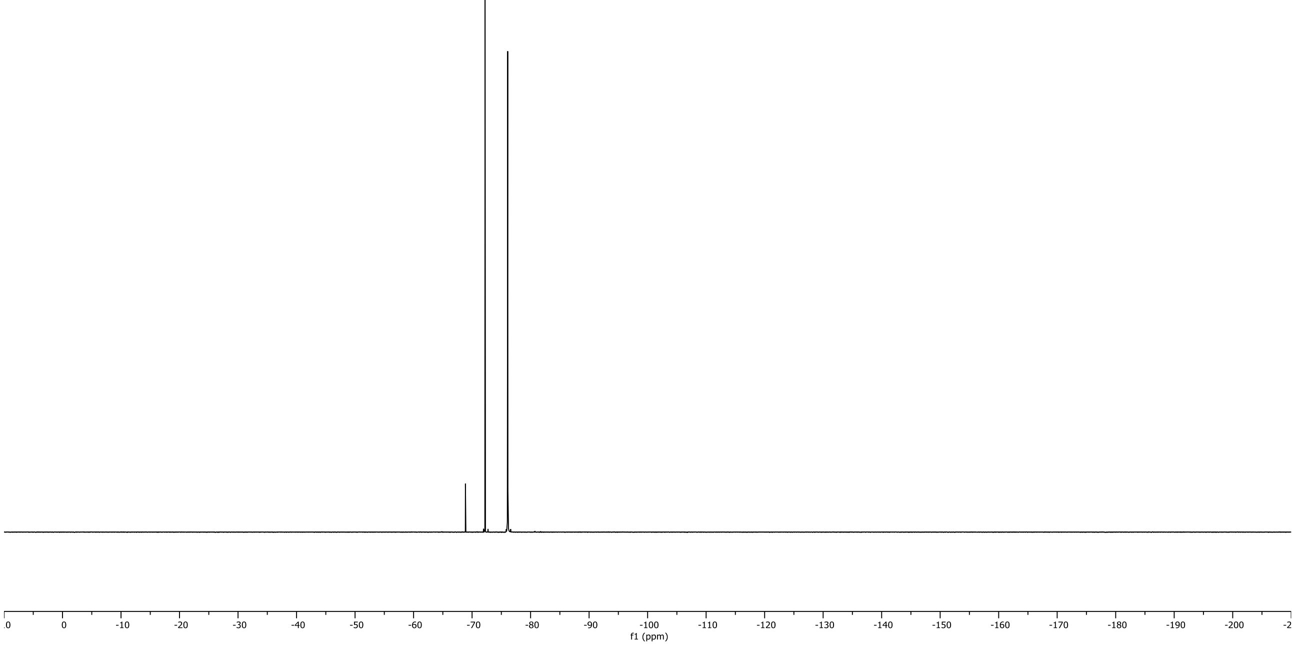

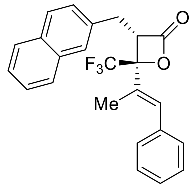

40 major

$^{13}C\{^1H\}$, $CDCl_3$, 126 MHz

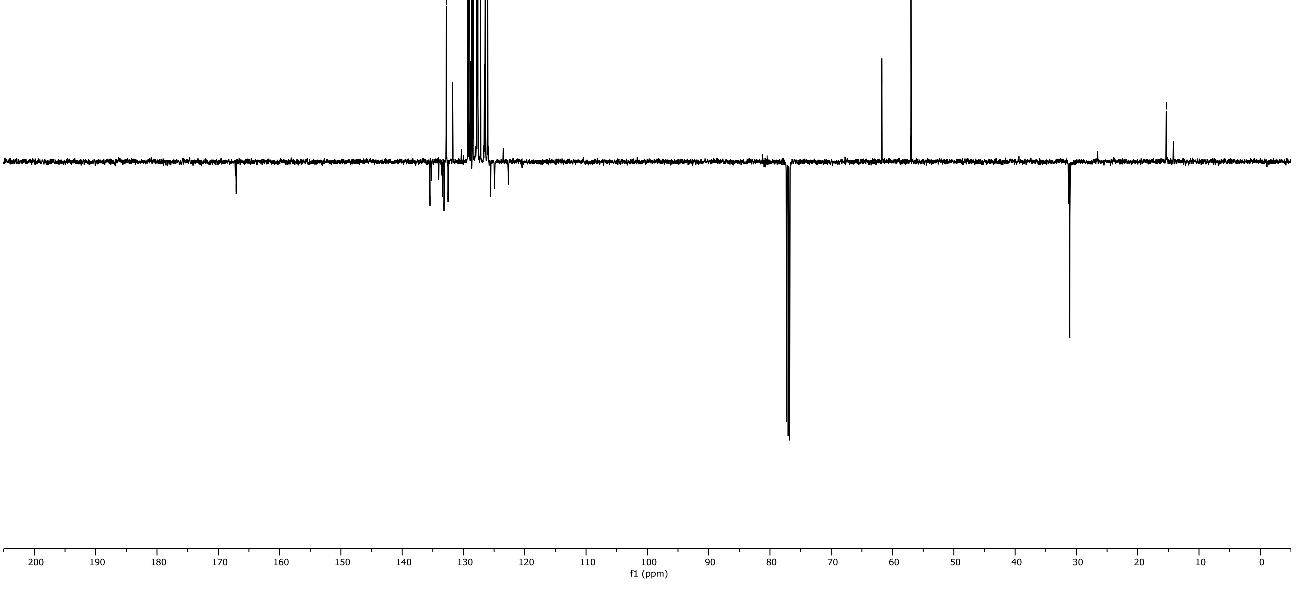

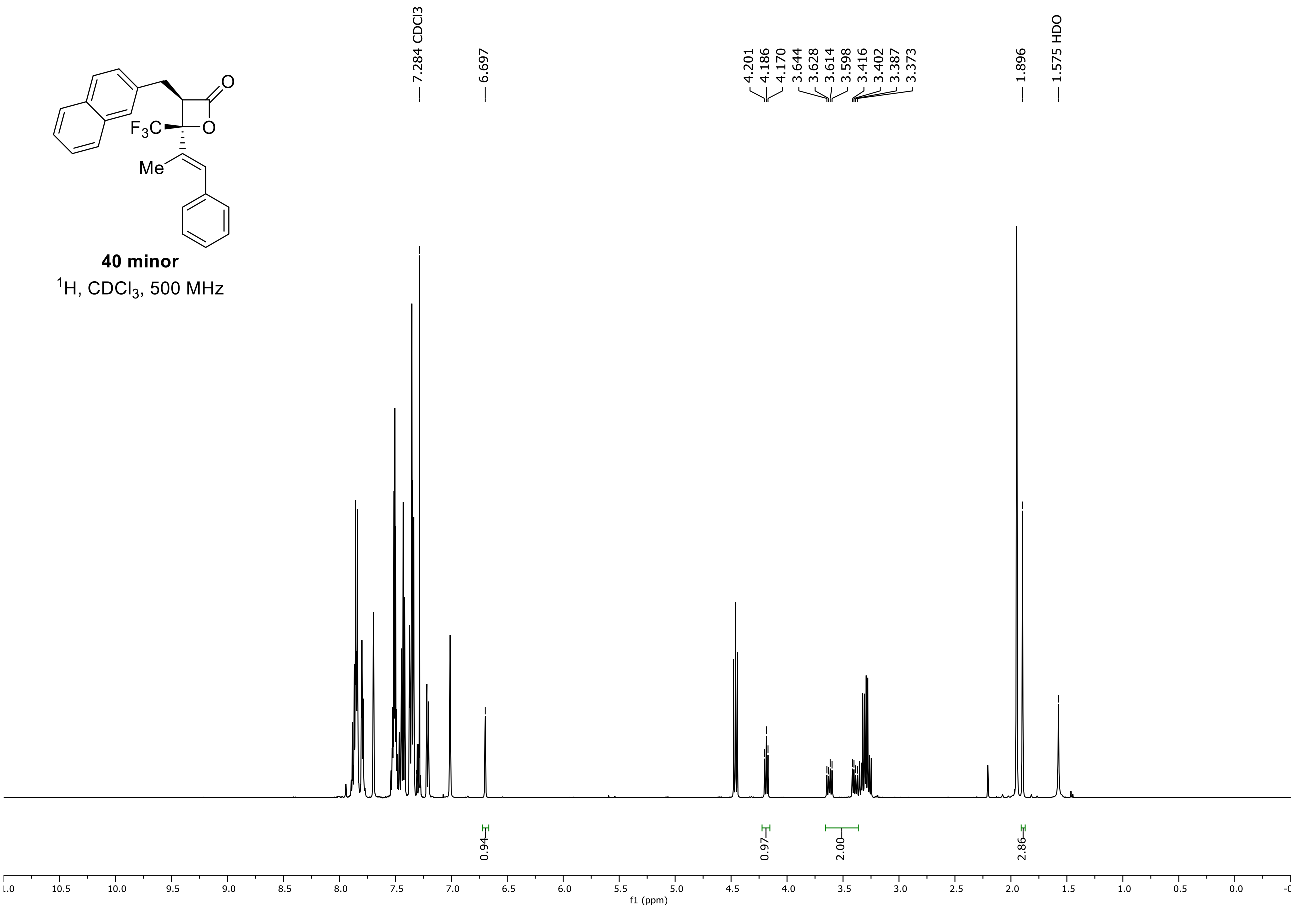
40 minor
^{1}H, $CDCl_3$, 500 MHz
F_3C
Me
O
7.284 CDCl3
6.697
4.201
4.186
4.170
3.644
3.628
3.614
3.598
3.416
3.402
3.387
3.373
1.896
1.575 HDO
0.94
0.97
2.00
2.86
f1 (ppm)

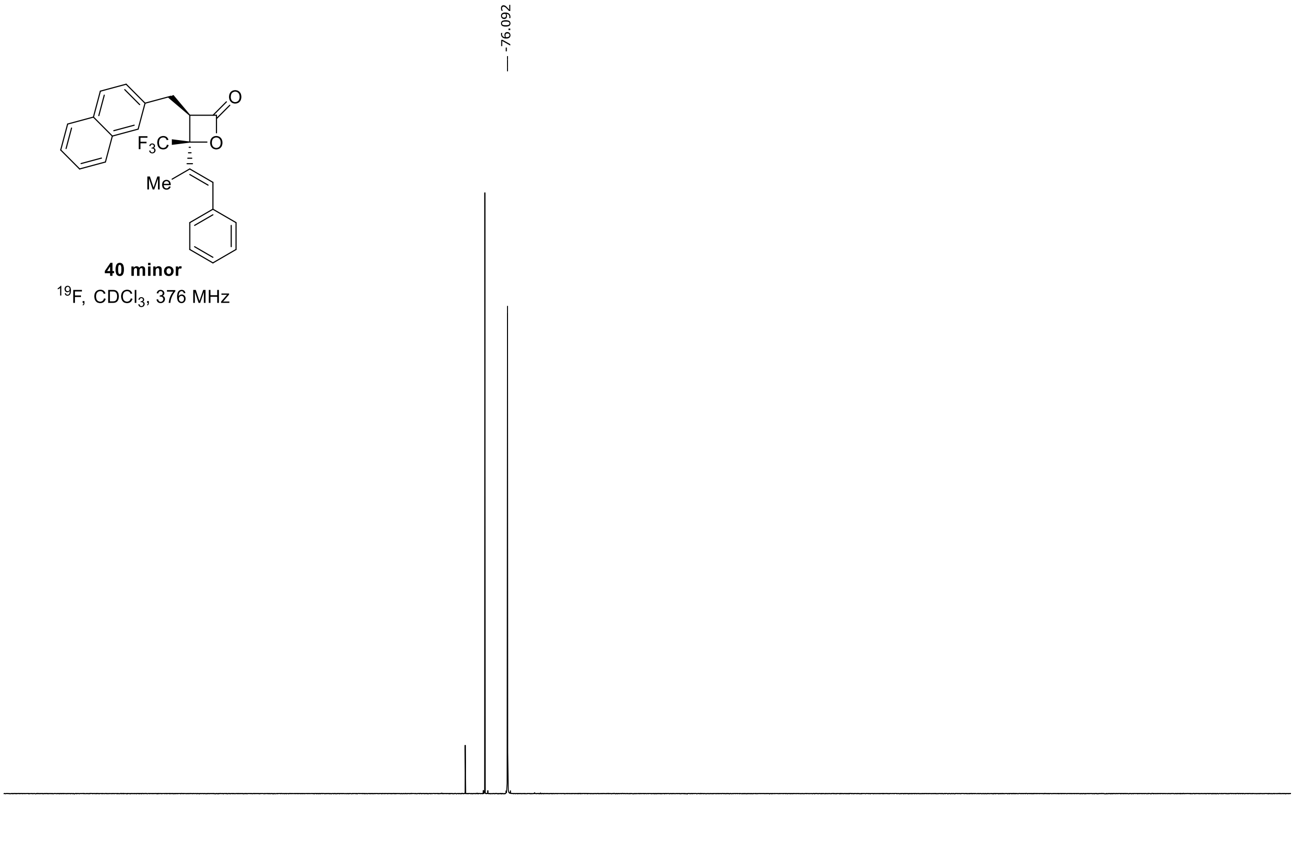
40 minor
^{19}F, $CDCl_3$, 376 MHz
F_3C
Me
O
-76.092
f1 (ppm)

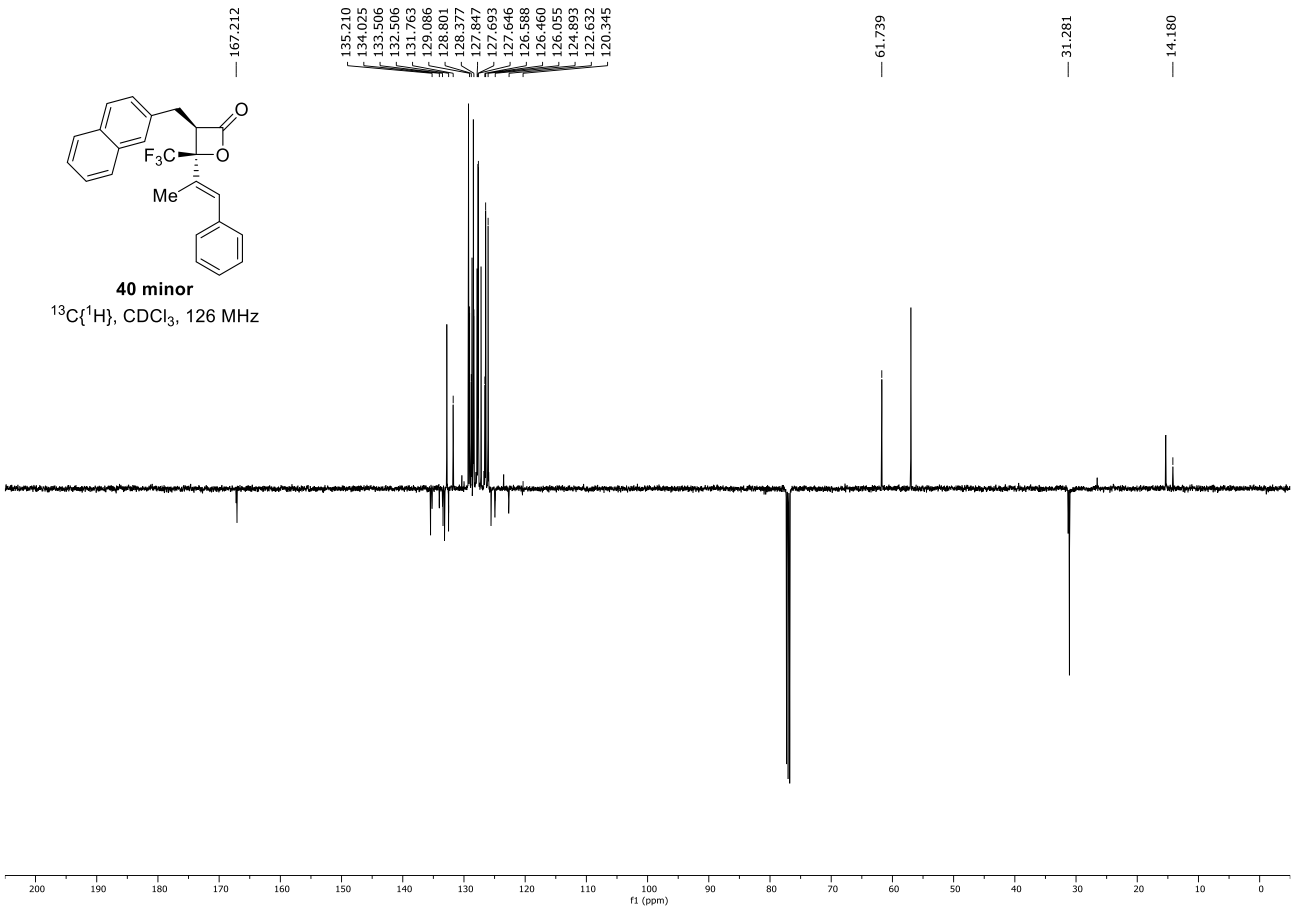

40 minor
$^{13}C\{^1H\}$, $CDCl_3$, 126 MHz

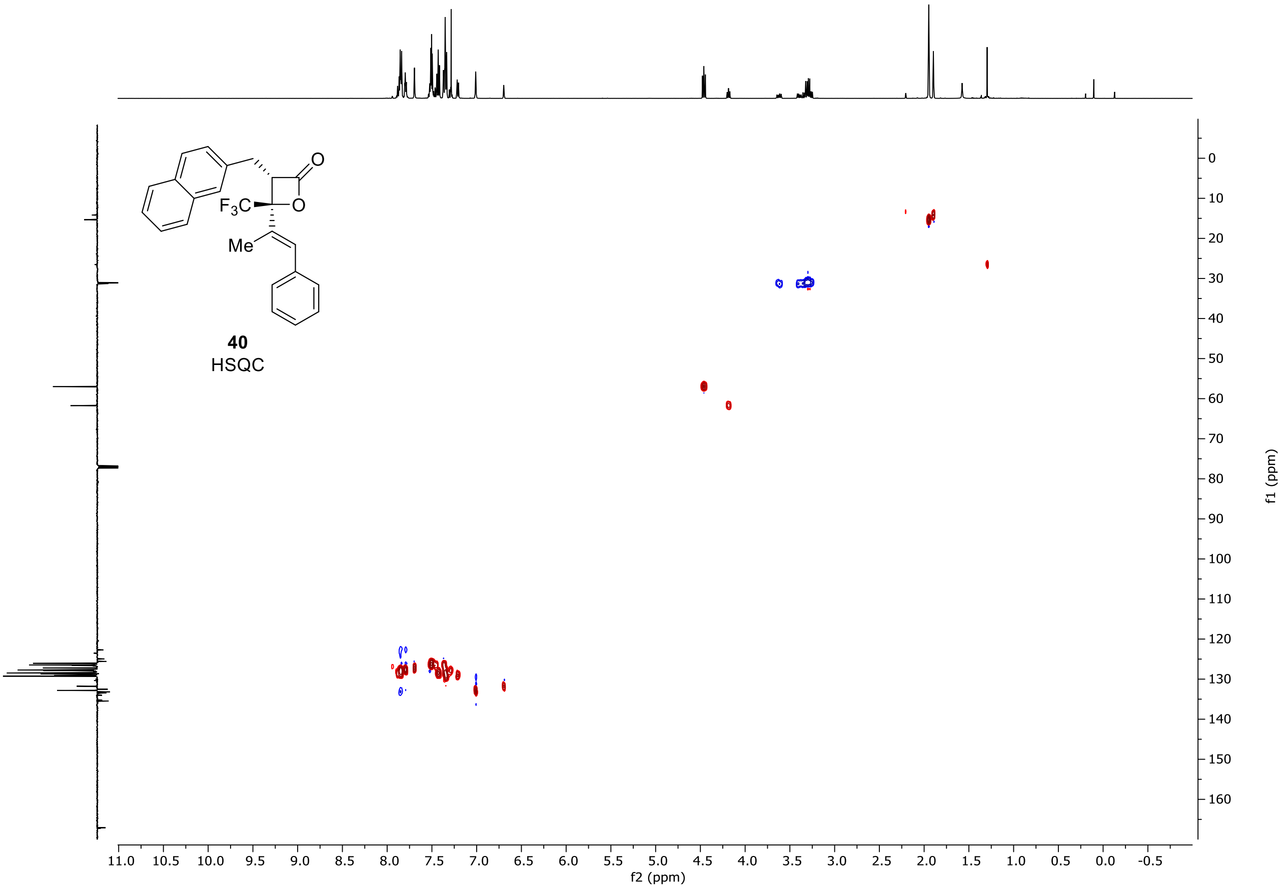

40
HSQC

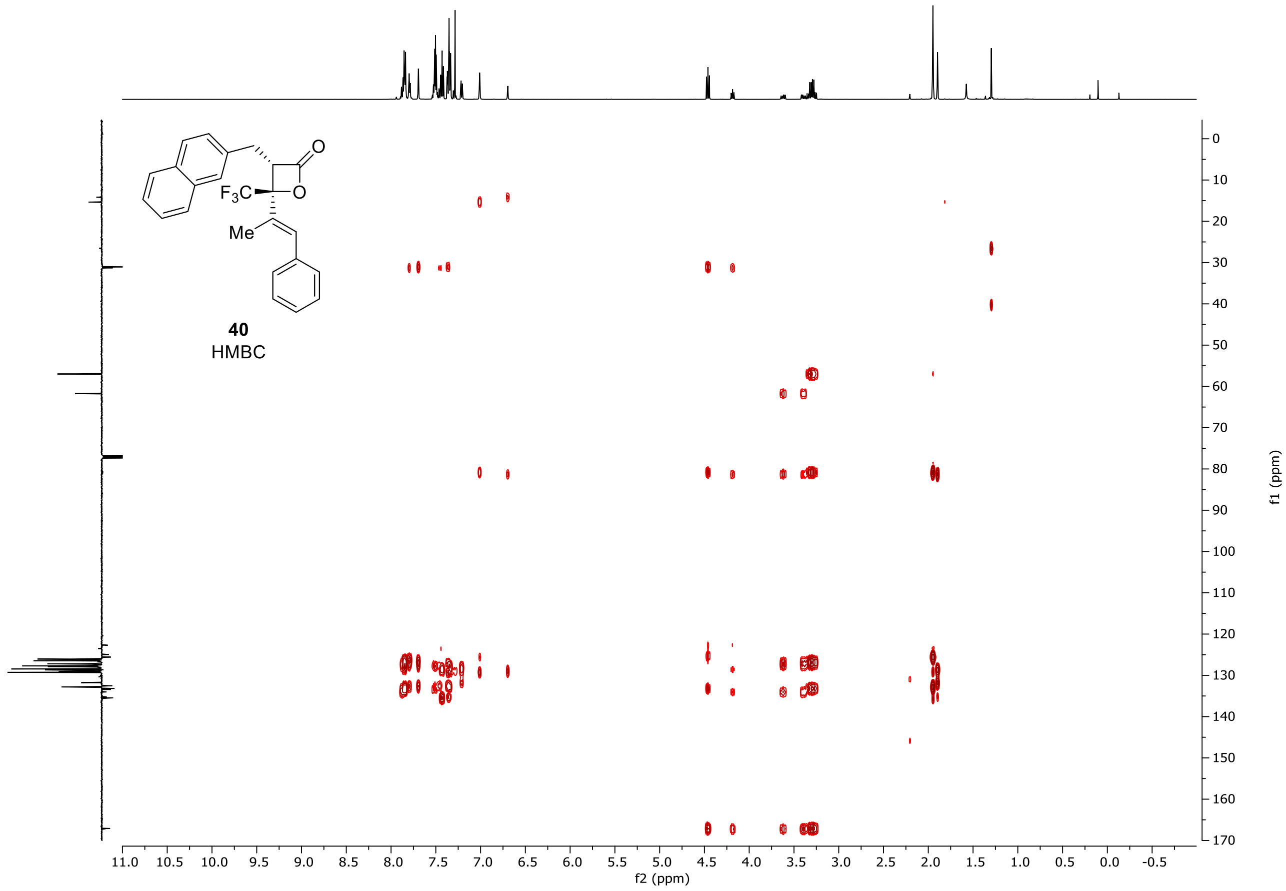
F3C
O
Me
40
HMBC
f2 (ppm)
f1 (ppm)

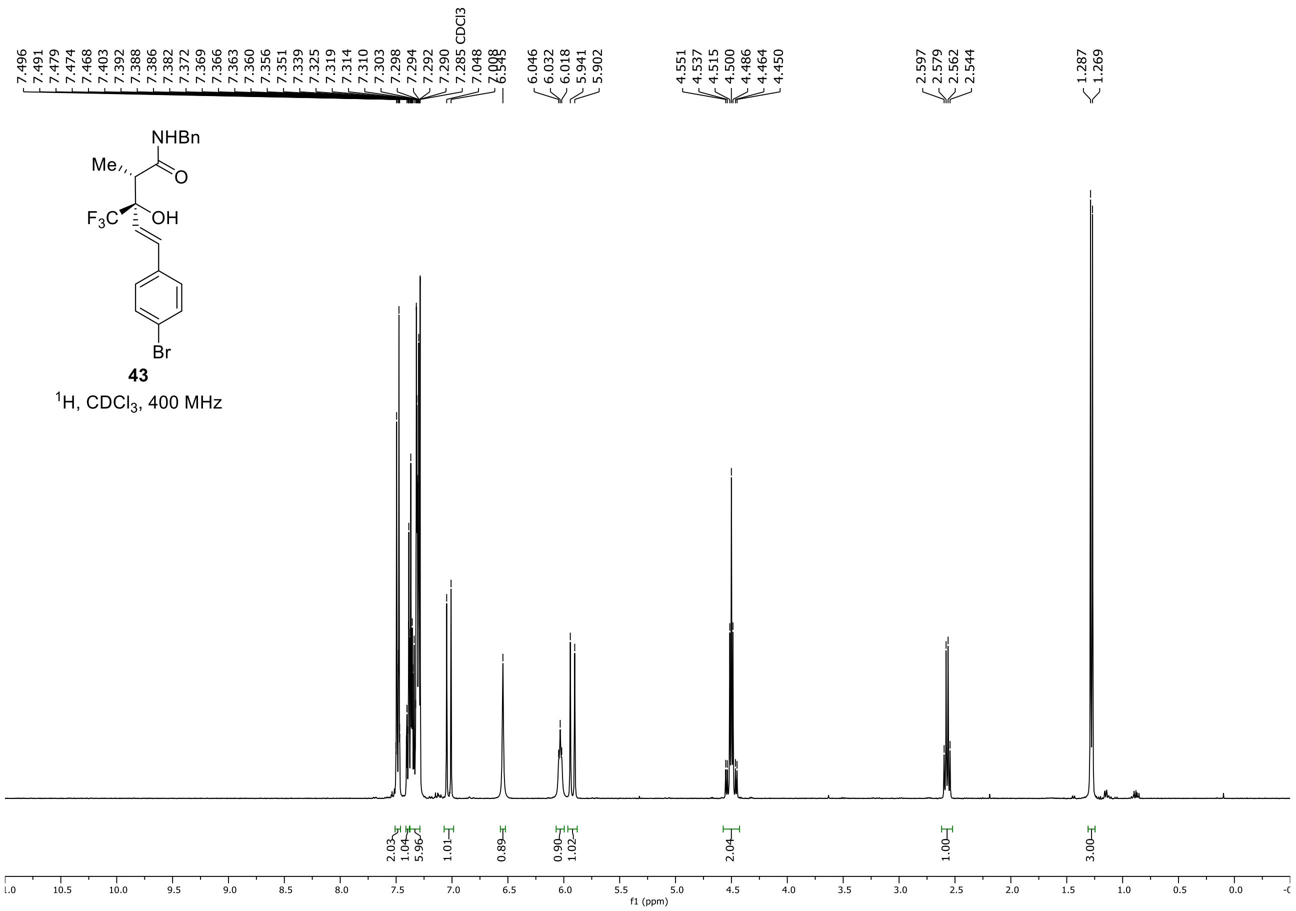
NHBn
Me
O
F3C
OH
Br
43
1H, CDCl3, 400 MHz
CDCl3
f1 (ppm)

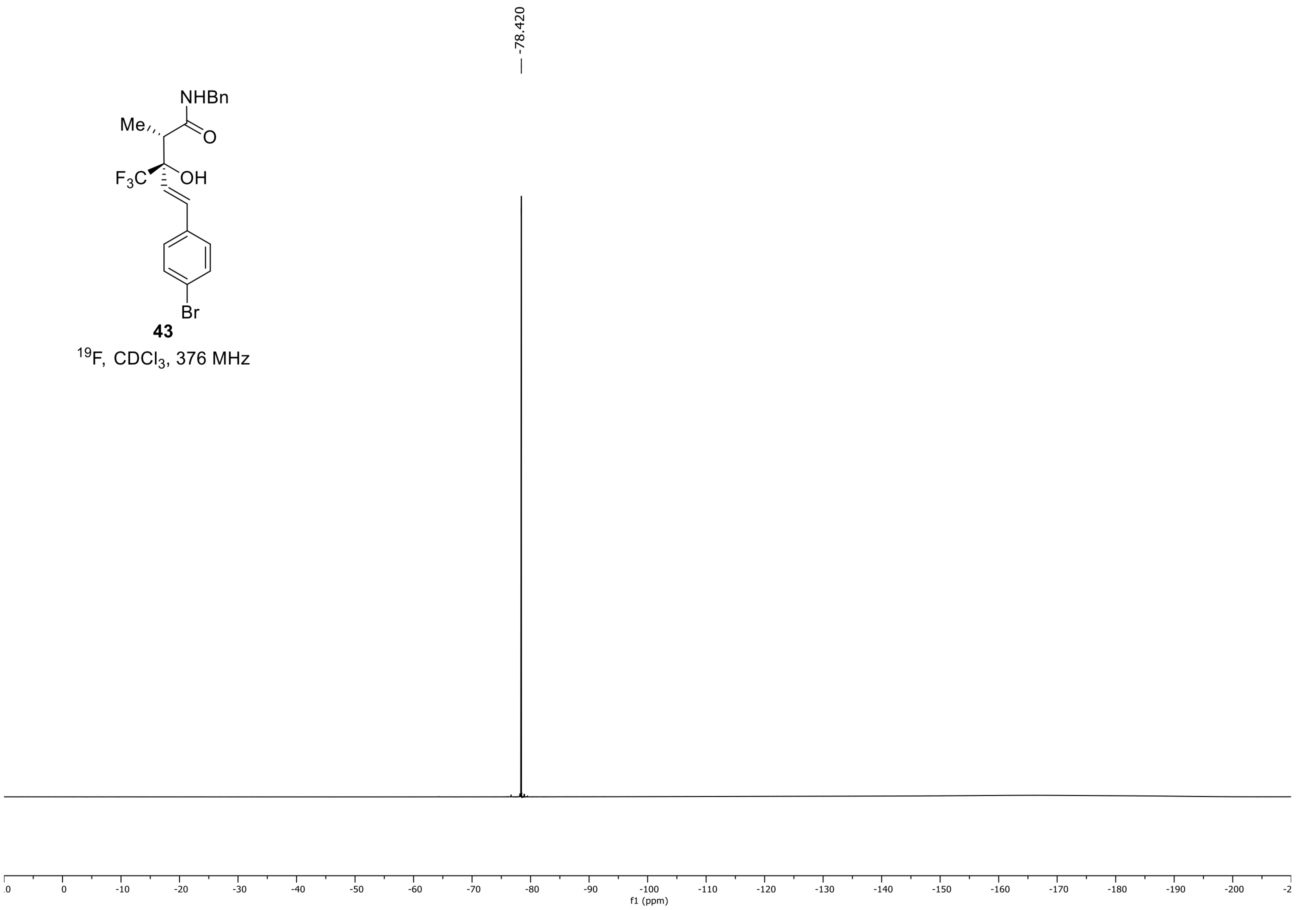

NHBn
Me
O
F_3C
OH
Br
43
^{19}F, $CDCl_3$, 376 MHz
-78.420
f1 (ppm)

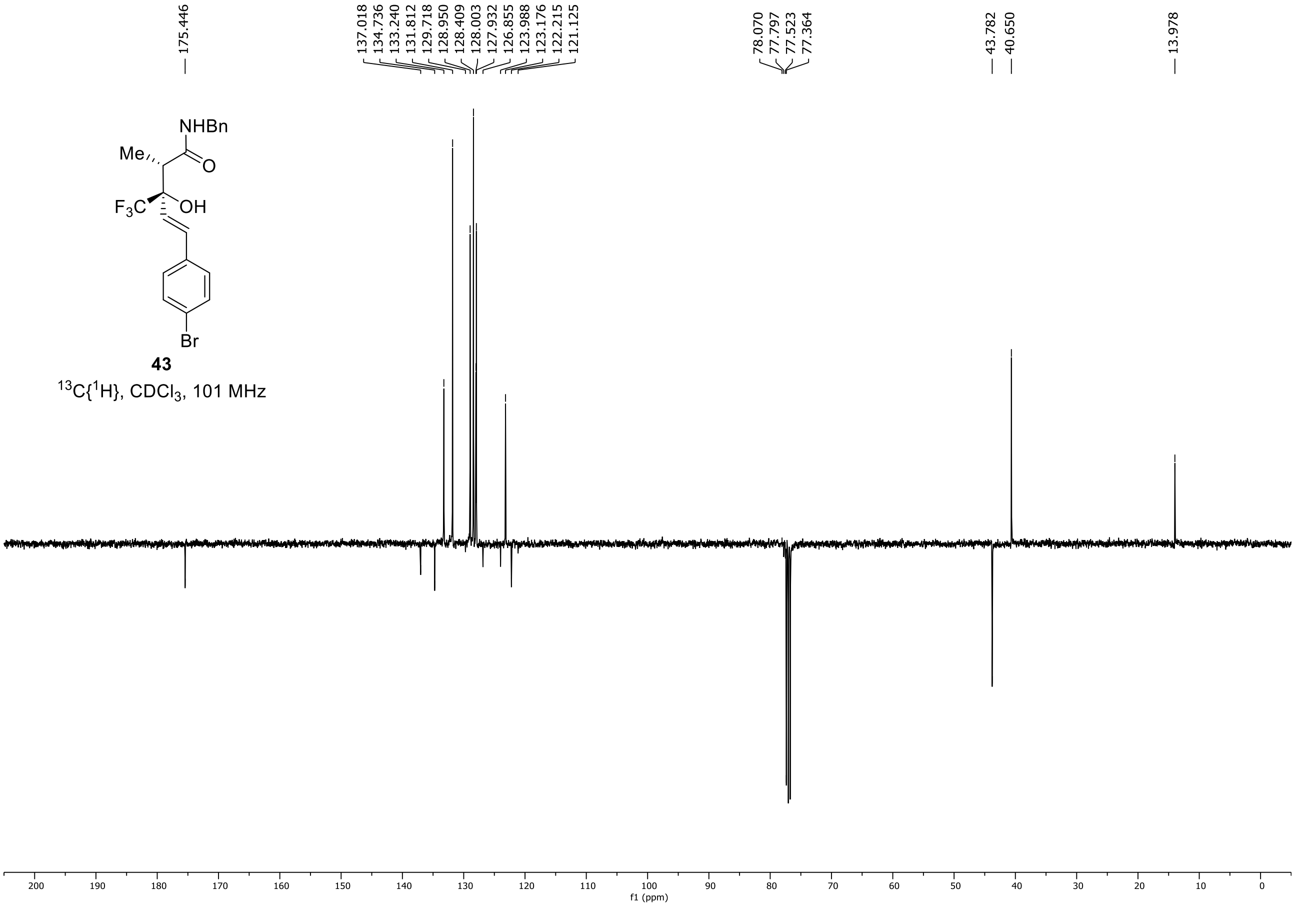

NHBn
Me
O
F_3C
OH
Br
43
$^{13}C\{^1H\}$, $CDCl_3$, 101 MHz
175.446
137.018
134.736
133.240
131.812
129.718
128.950
128.409
128.003
127.932
126.855
123.988
123.176
122.215
121.125
78.070
77.797
77.523
77.364
43.782
40.650
13.978
f1 (ppm)

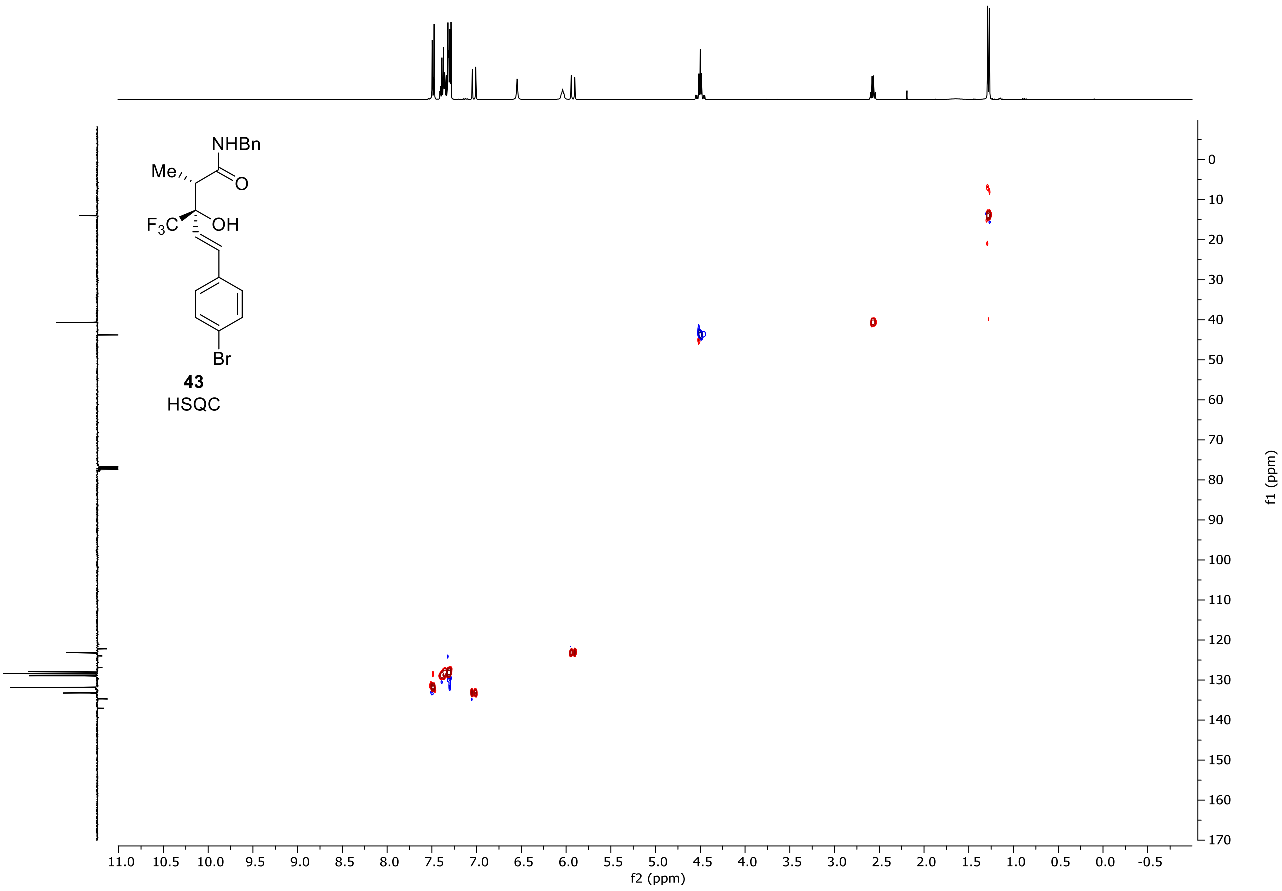
NHBn
Me
O
F3C
OH
Br
43
HSQC
f1 (ppm)
f2 (ppm)

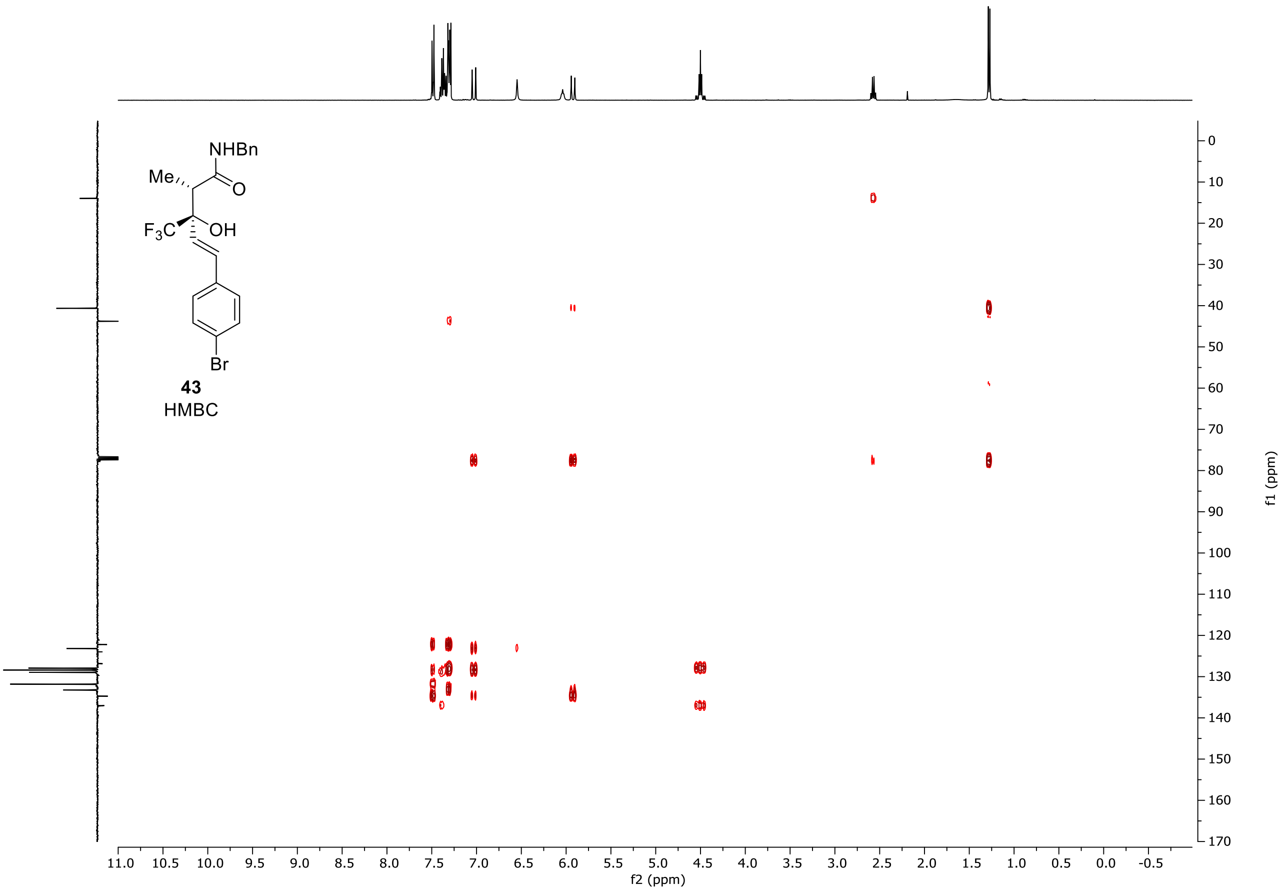
NHBn
Me
O
F3C
OH
Br
43
HMBC
f1 (ppm)
f2 (ppm)

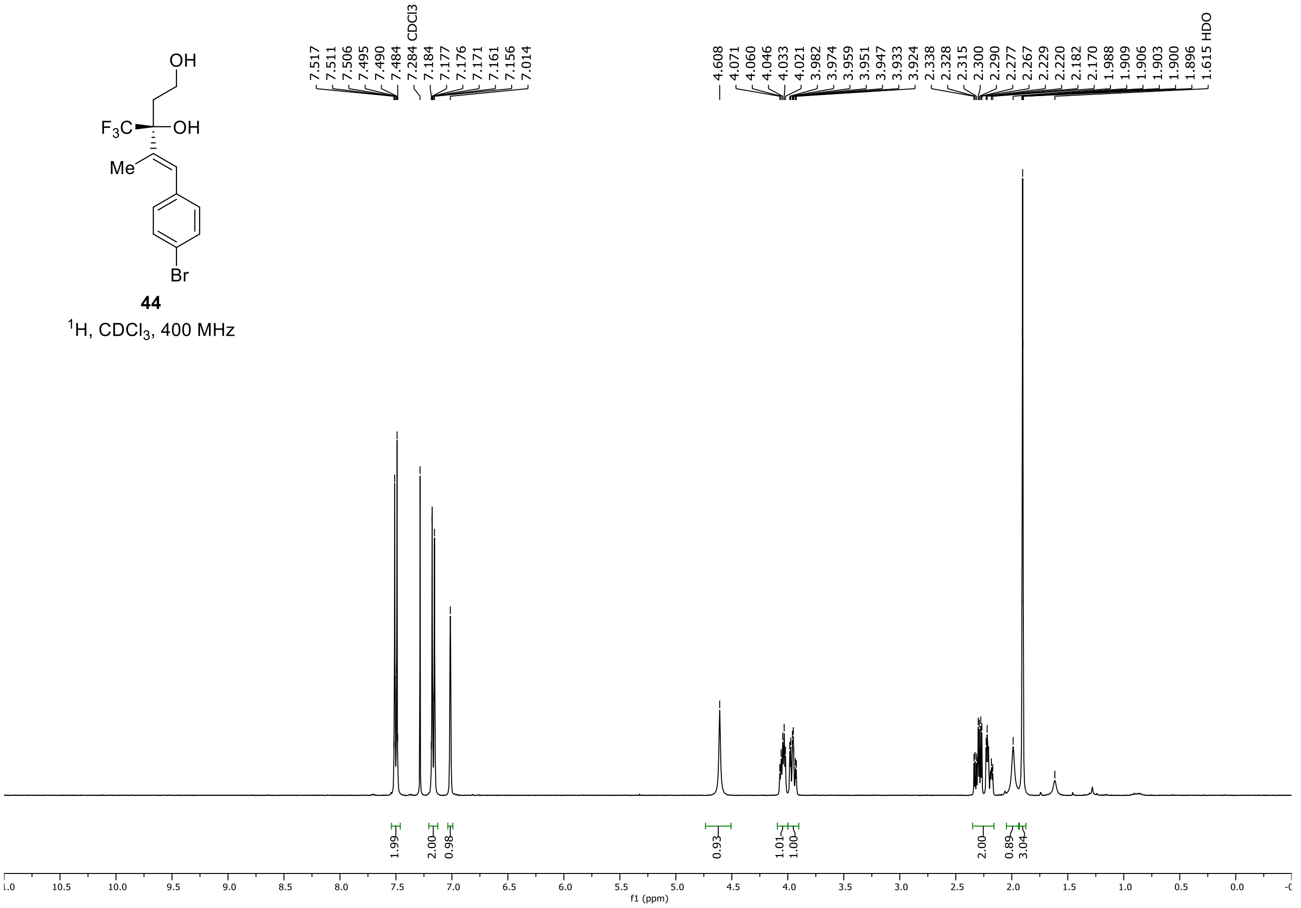

44

^{1}H, $CDCl_3$, 400 MHz

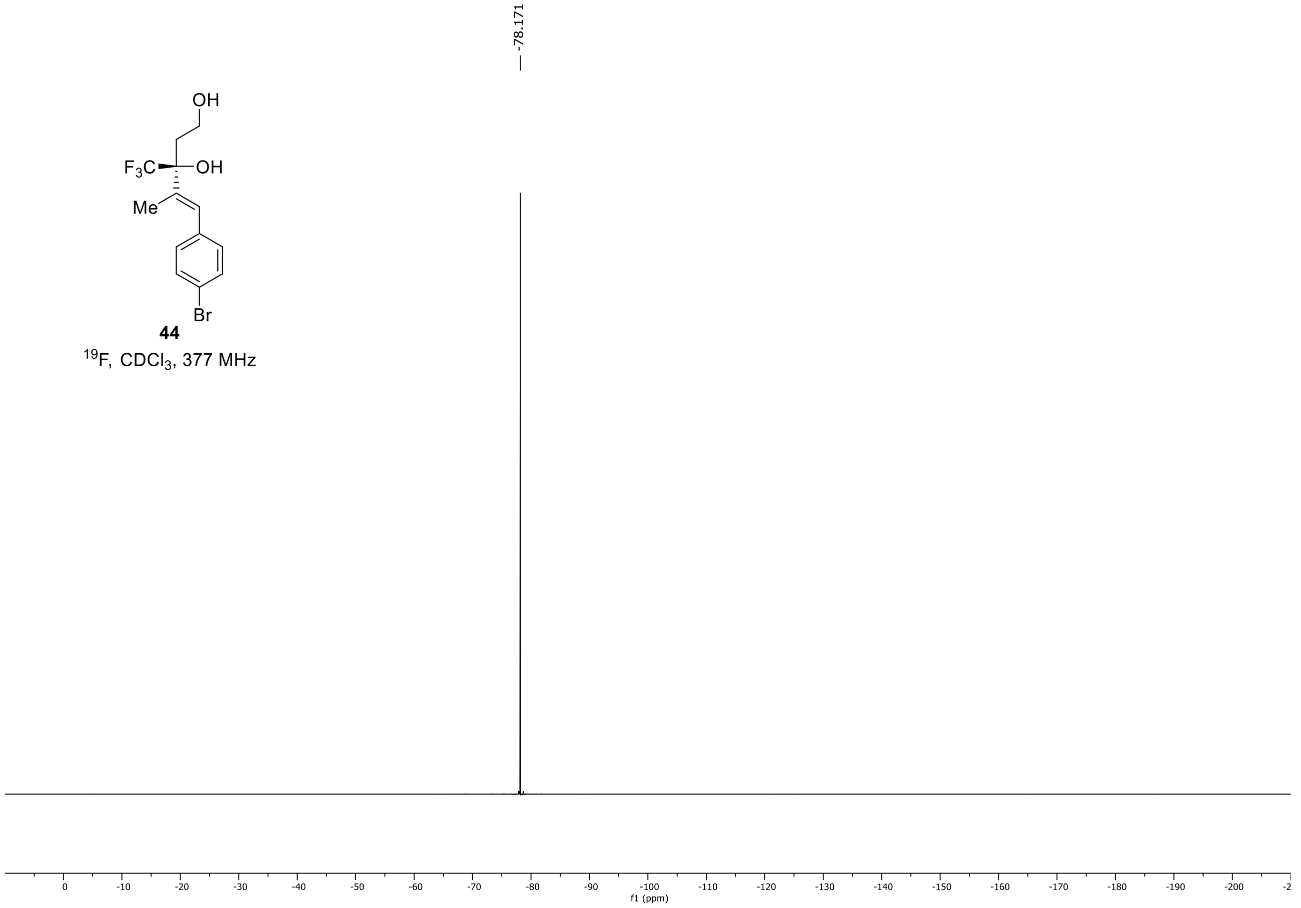

44

^{19}F, $CDCl_3$, 377 MHz

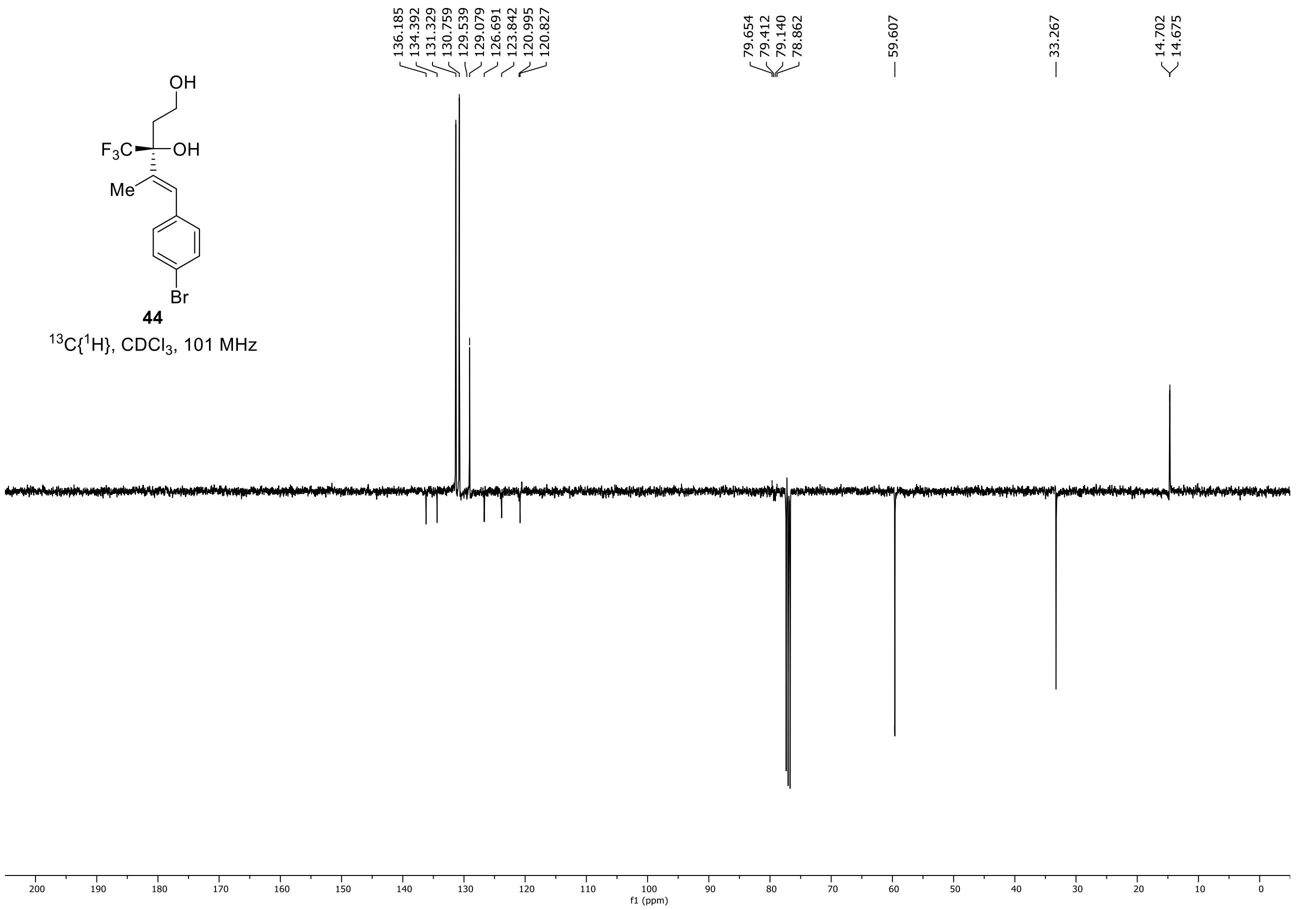
136.185
134.392
131.329
130.759
129.539
129.079
126.691
123.842
120.995
120.827
79.654
79.412
79.140
78.862
59.607
33.267
14.702
14.675
OH
F3C
OH
Me
Br
44
13C{1H}, CDCl3, 101 MHz
f1 (ppm)

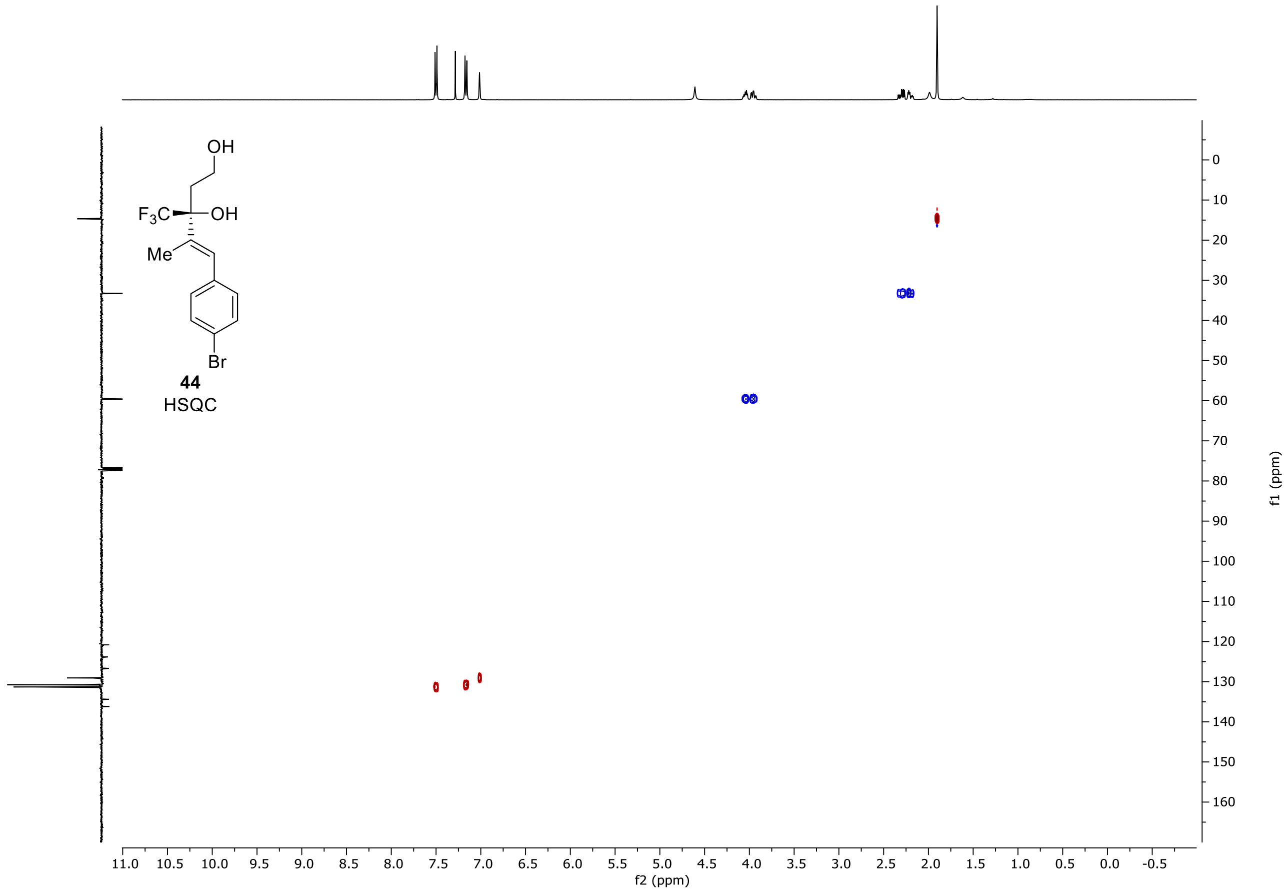
OH
F3C
OH
Me
Br
44
HSQC
f2 (ppm)
f1 (ppm)

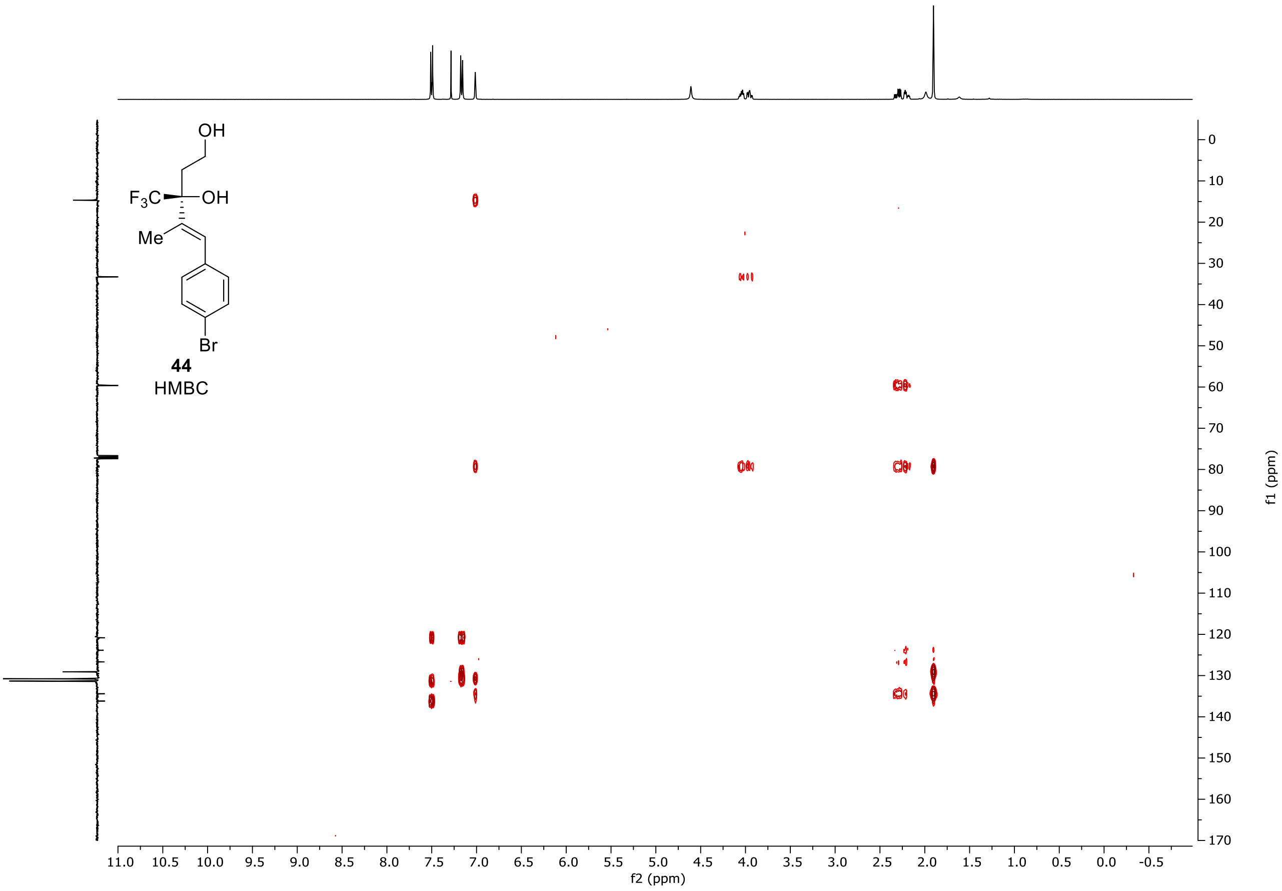
OH
F_3C
OH
Me
Br
44
HMBC
f2 (ppm)
f1 (ppm)

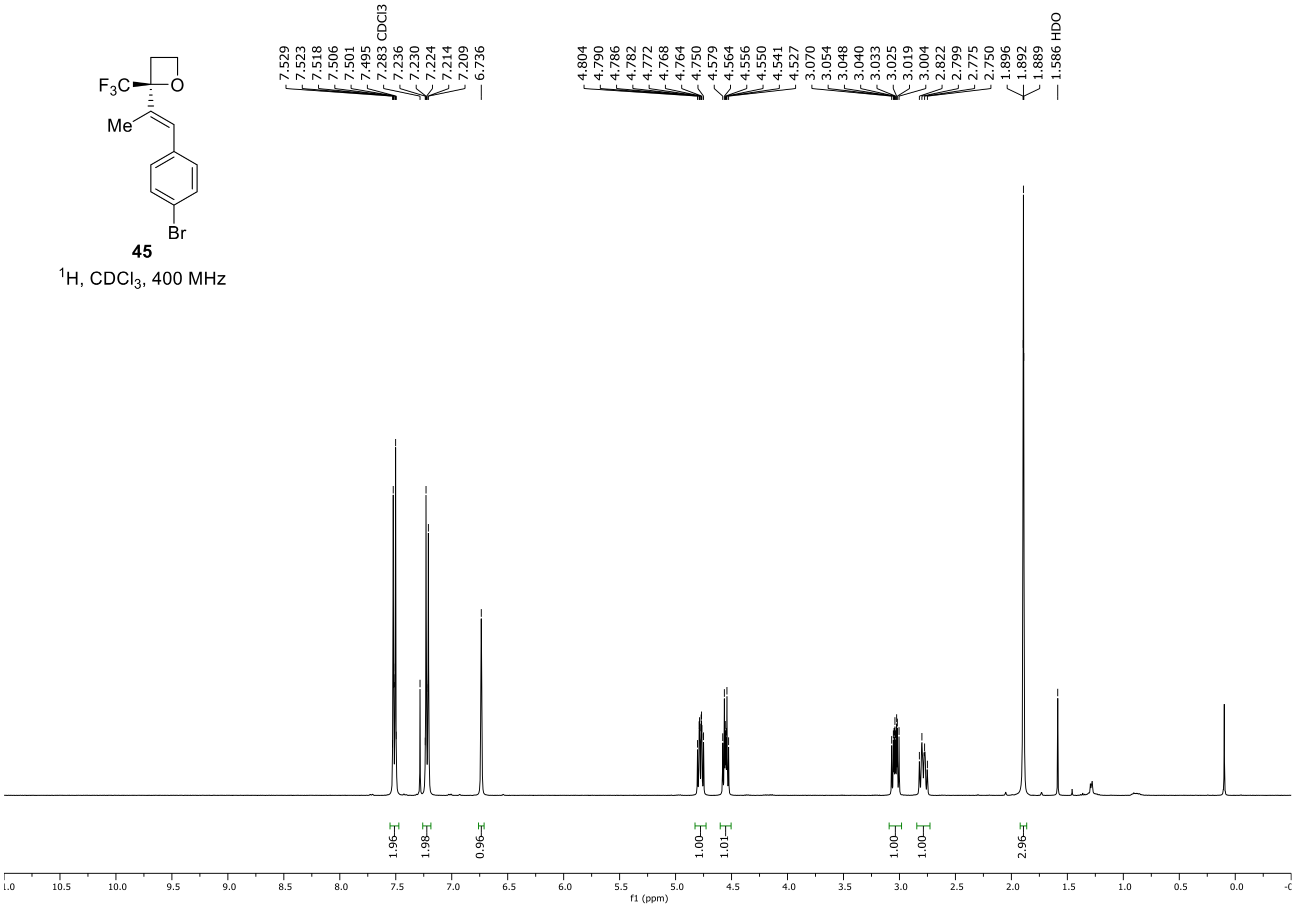
F_3C
O
Me
Br
45
^1H, $CDCl_3$, 400 MHz
7.529
7.523
7.518
7.506
7.501
7.495
7.283 CDCl3
7.236
7.230
7.224
7.214
7.209
6.736
4.804
4.790
4.786
4.782
4.772
4.768
4.764
4.750
4.579
4.564
4.556
4.550
4.541
4.527
3.070
3.054
3.048
3.040
3.033
3.025
3.019
3.004
2.822
2.799
2.775
2.750
1.896
1.892
1.889
1.586 HDO
1.96
1.98
0.96
1.00
1.01
1.00
1.00
2.96
f1 (ppm)

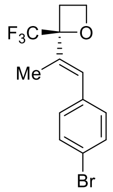

45

^{19}F, $CDCl_3$, 377 MHz

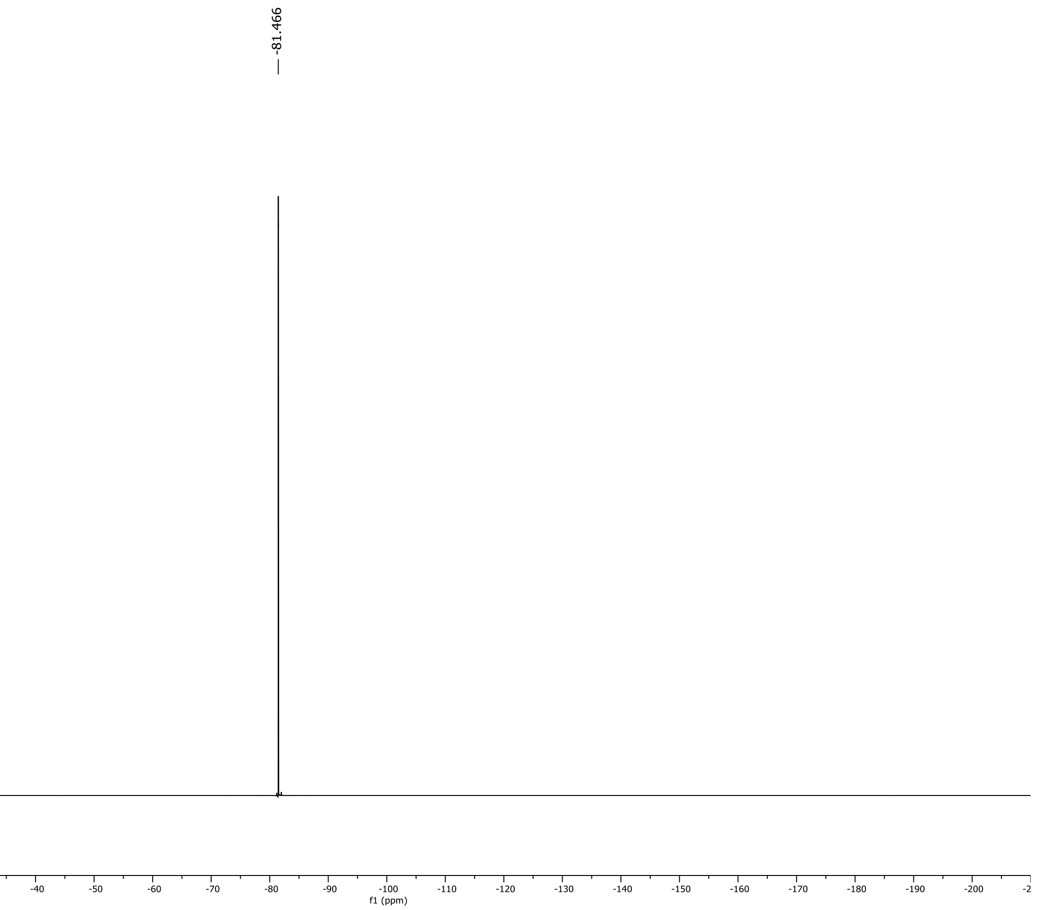

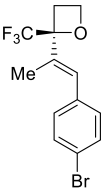

45

$^{13}C\{^1H\}$, $CDCl_3$, 101 MHz

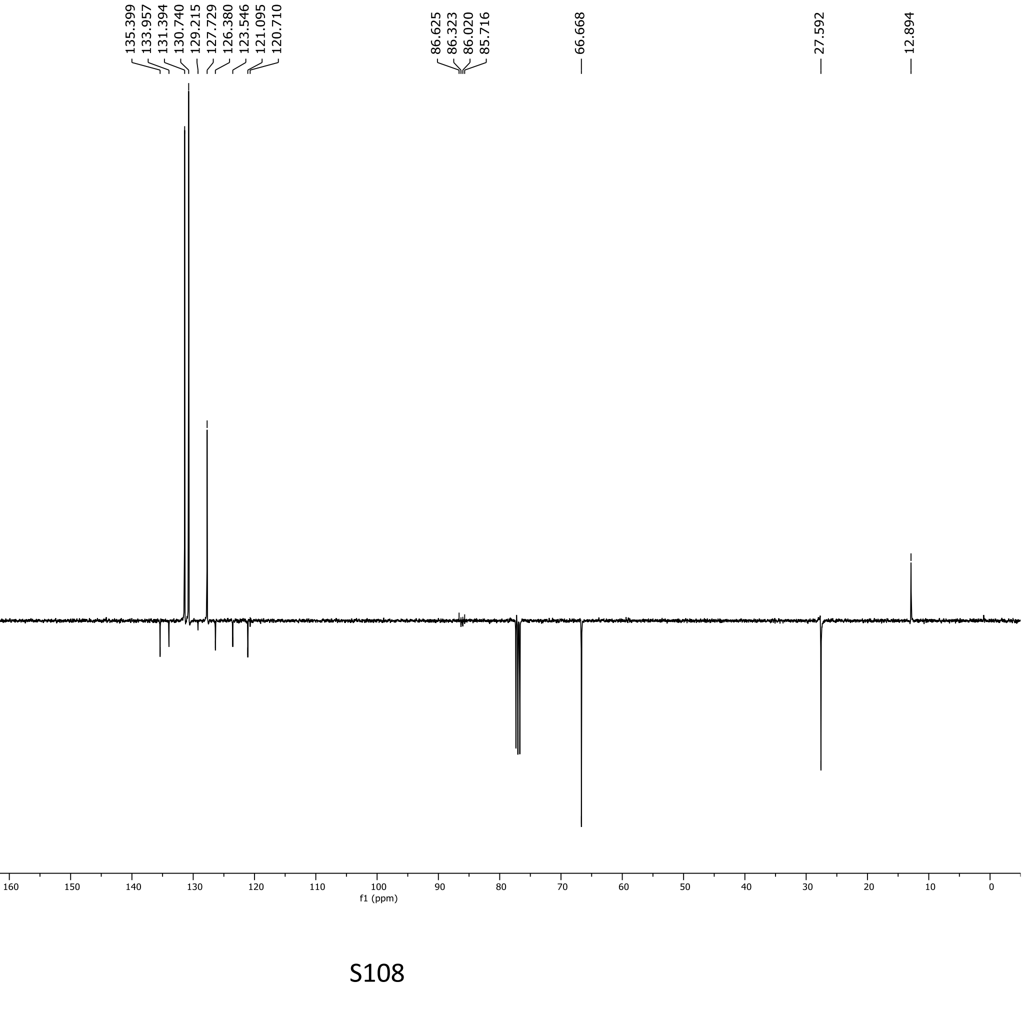

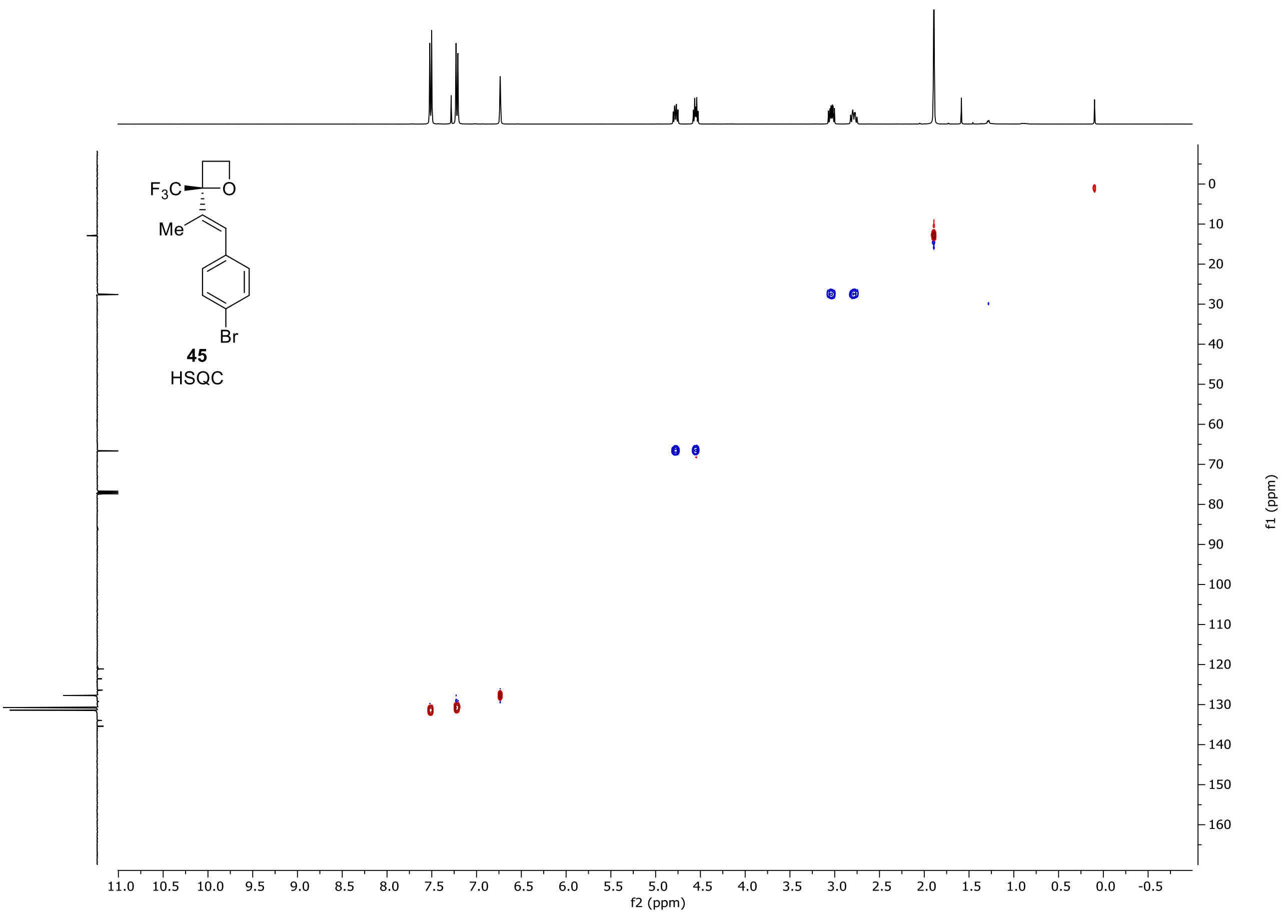
F3C
O
Me
Br
45
HSQC
f2 (ppm)
f1 (ppm)

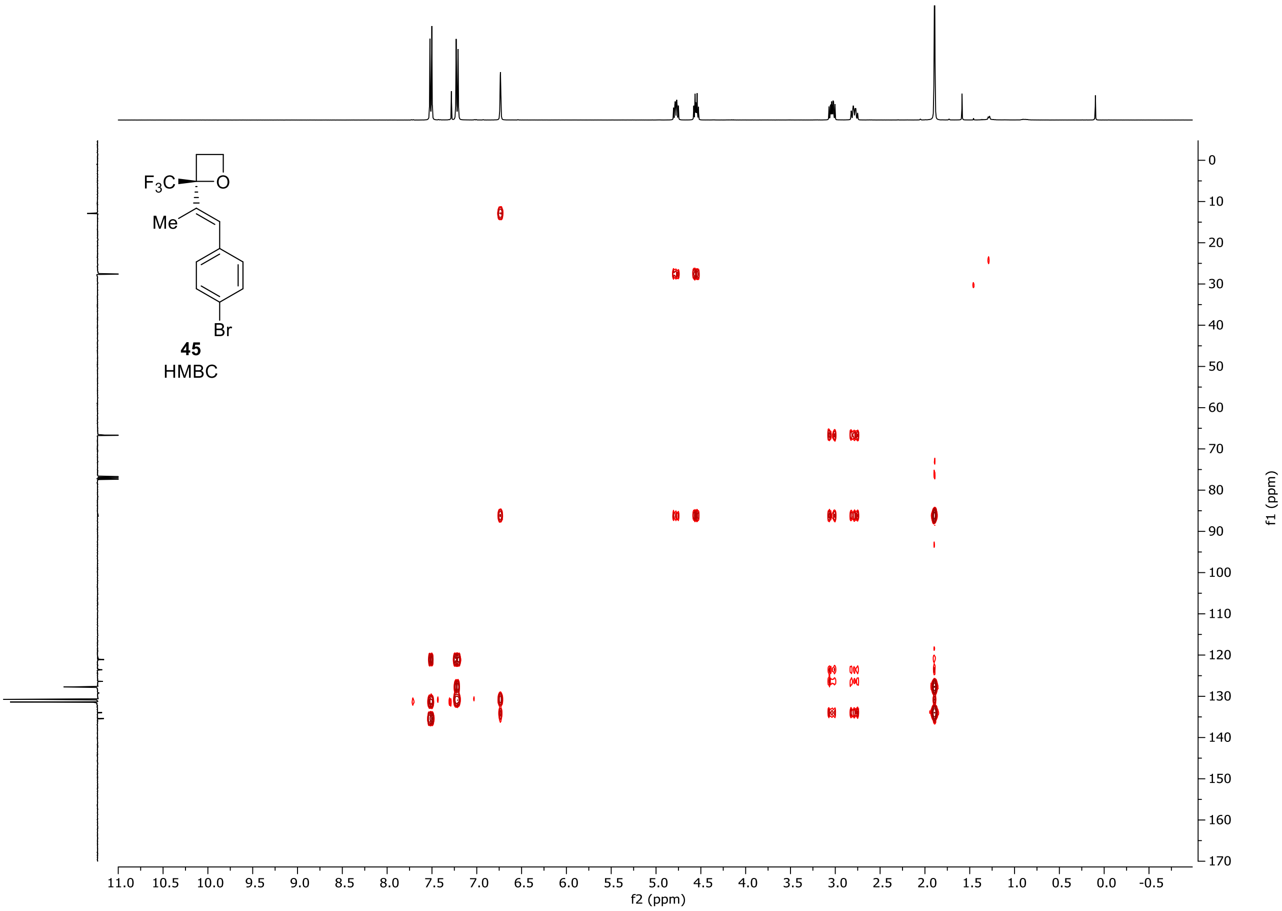
F3C
O
Me
Br
45
HMBC
f2 (ppm)
f1 (ppm)

Appendix II. HPLC Traces

HPLC Data for **3**: Chiralpak IB (99.3:0.7 hexane:IPA, flow rate 1.0 mL min^{-1}, 254 nm, 30 °C), t_R (major): 16.1 min, t_R (minor): 24.9 min, 92:8 er.

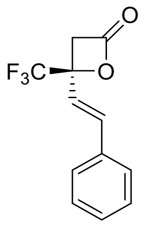

3

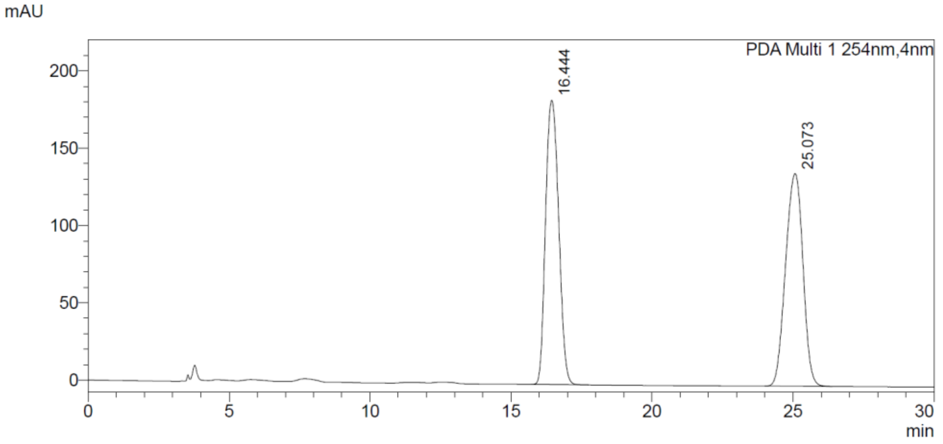

<Peak Table>

PDA Ch1 254nm

Peak#	Ret. Time	Area%
1	16.444	50.222
2	25.073	49.778
Total		100.000

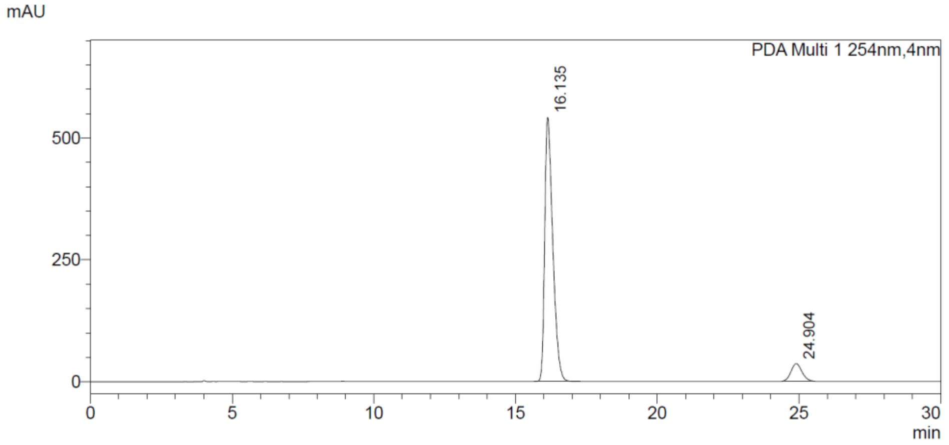

<Peak Table>

PDA Ch1 254nm

Peak#	Ret. Time	Area%
1	16.135	91.869
2	24.904	8.131
Total		100.000

HPLC Data for **7**: **Chiral HPLC analysis**, Chiralpak IB (99:1 hexane:IPA, flow rate 1.0 mLmin^{-1}, 254 nm, 30 °C), t_R (major): 12.7 min, t_R (minor): 15.6 min, 92:8 er.

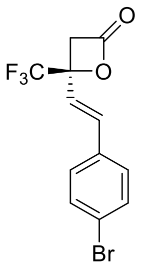

7

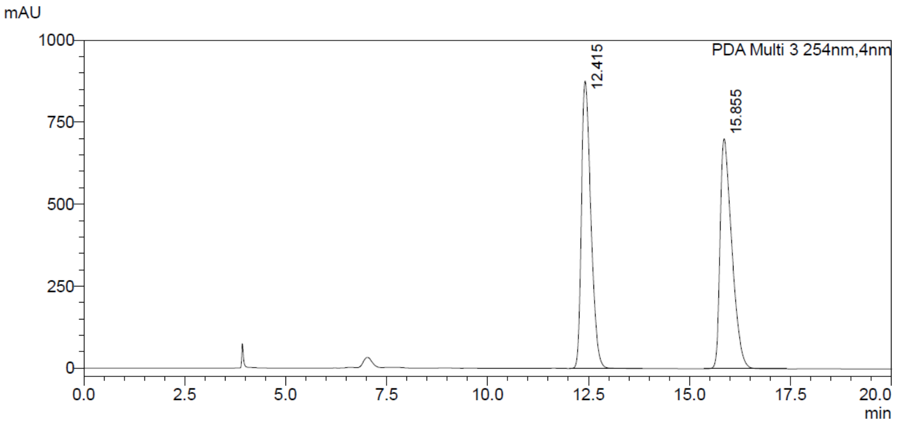

<Peak Table>

PDA Ch3 254nm

Peak#	Ret. Time	Area%
1	12.415	50.127
2	15.855	49.873
Total		100.000

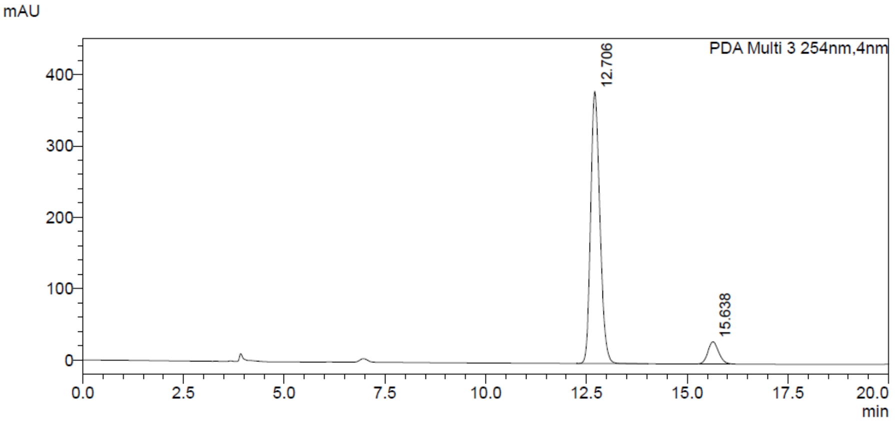

<Peak Table>

PDA Ch3 254nm

Peak#	Ret. Time	Area%
1	12.706	91.688
2	15.638	8.312
Total		100.000

HPLC Data for **8**: **Chiral HPLC analysis**, Chiralpak IB (99:1 hexane:IPA, flow rate 1.0 mLmin^{-1}, 254 nm, 30 °C), t_R (minor): 13.5 min, t_R (major): 16.6 min, 90:10 er.

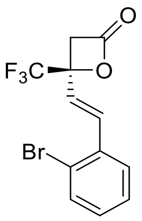

8

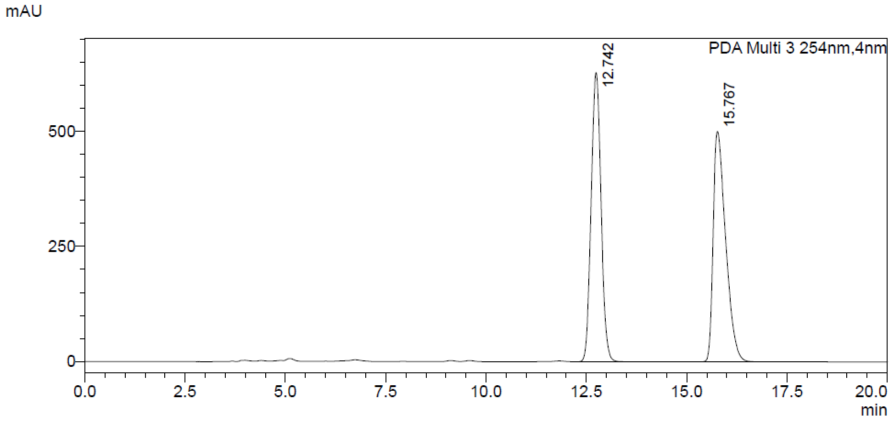

<Peak Table>

PDA Ch3 254nm

Peak#	Ret. Time	Area%
1	12.742	49.959
2	15.767	50.041
Total		100.000

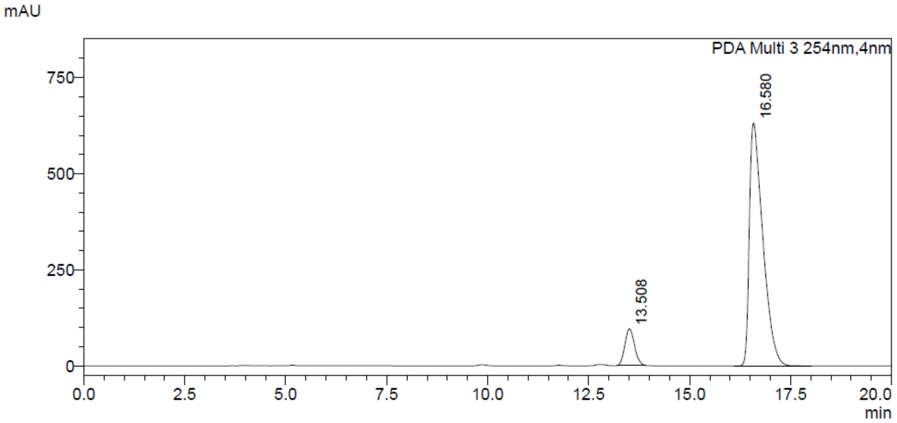

<Peak Table>

PDA Ch3 254nm

Peak#	Ret. Time	Area%
1	13.508	9.746
2	16.580	90.254
Total		100.000

HPLC Data for **9**: **Chiral HPLC analysis**, Chiralpak AS-H (99.5:0.5 hexane:IPA, flow rate 1.0 mL min^{-1}, 254 nm, 30 °C), t_R (minor): 11.1 min, t_R (major): 15.3 min, 89:11 er.

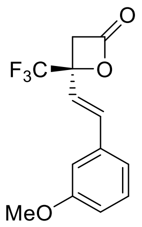

9

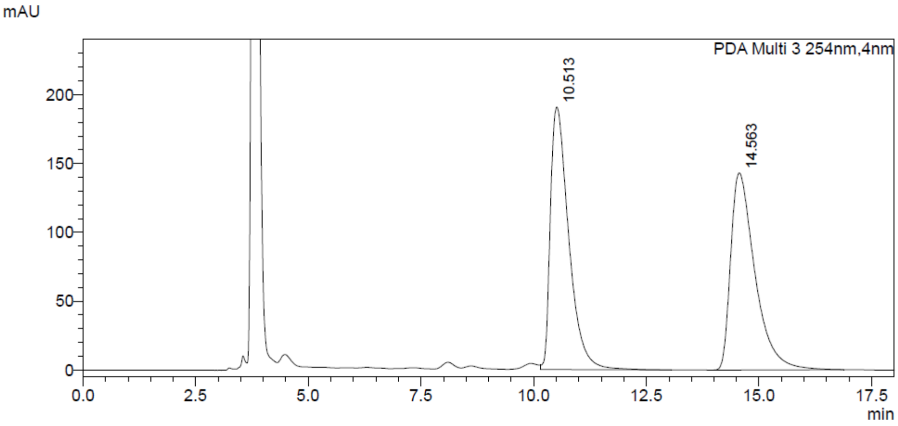

<Peak Table>

PDA Ch3 254nm

Peak#	Ret. Time	Area%
1	10.513	50.290
2	14.563	49.710
Total		100.000

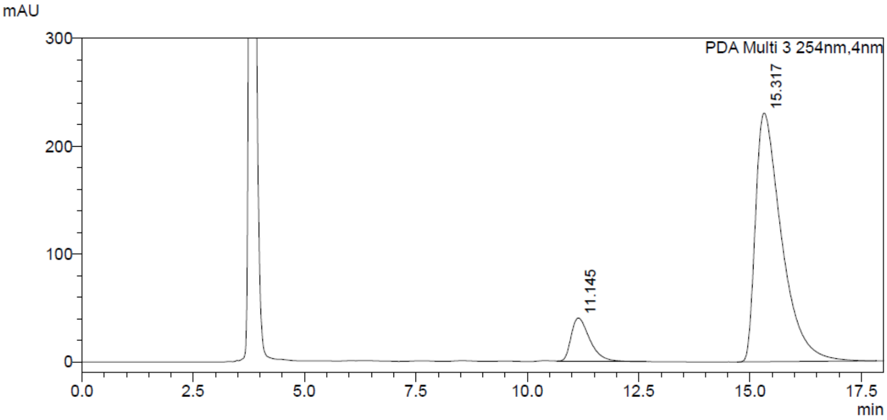

<Peak Table>

PDA Ch3 254nm

Peak#	Ret. Time	Area%
1	11.145	11.213
2	15.317	88.787
Total		100.000

HPLC Data for **10**: **Chiral HPLC analysis**, Chiralcel OJ-H (99.8:0.2 hexane:IPA, flow rate 1.0 mLmin^{-1}, 254 nm, 30 °C), t_R (minor): 34.5 min, t_R (major): 38.3 min, 92:8 er.

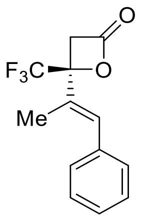

10

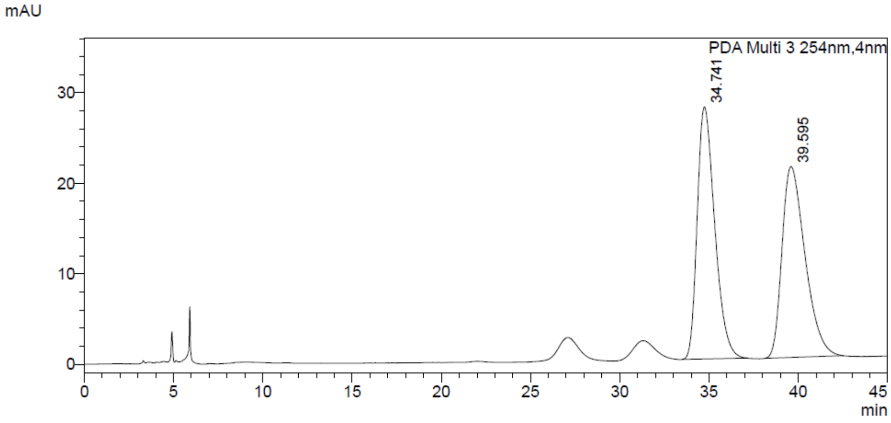

<Peak Table>

PDA Ch3 254nm

Peak#	Ret. Time	Area%
1	34.741	50.102
2	39.595	49.898
Total		100.000

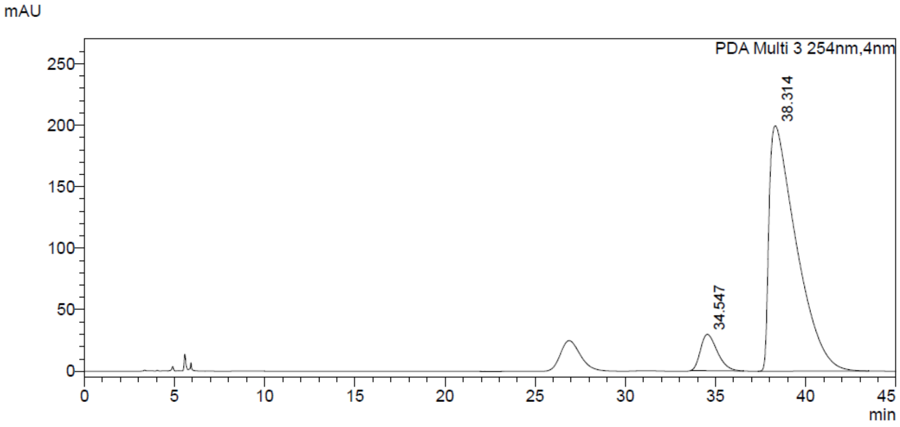

<Peak Table>

PDA Ch3 254nm

Peak#	Ret. Time	Area%
1	34.547	8.354
2	38.314	91.646
Total		100.000

HPLC Data for **11**: **Chiral HPLC analysis**, Chiralpak AS-H (99.5:0.5 hexane:IPA, flow rate 1.0 mLmin^{-1}, 254 nm, 30 °C), t_R (minor): 7.9 min, t_R (major): 9.7 min, 94:6 er.

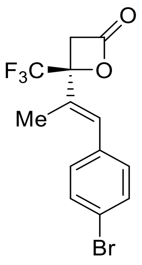

11

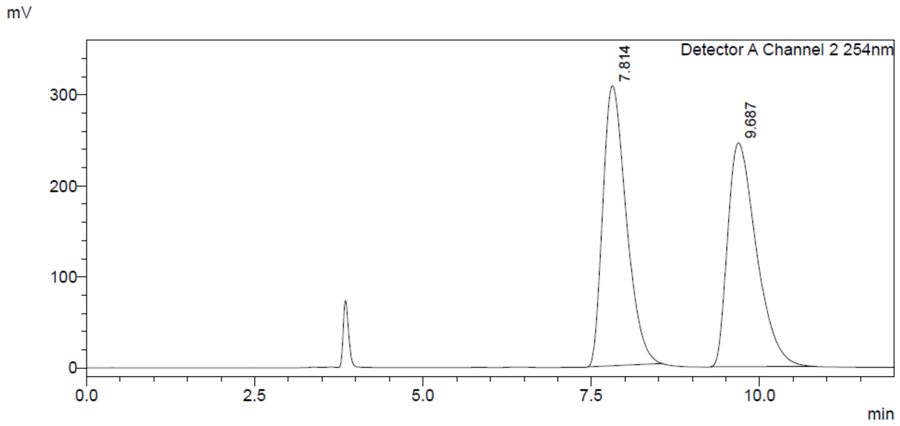

<Peak Table>

Detector A Channel 2 254nm

Peak#	Ret. Time	Area%
1	7.814	50.471
2	9.687	49.529
Total		100.000

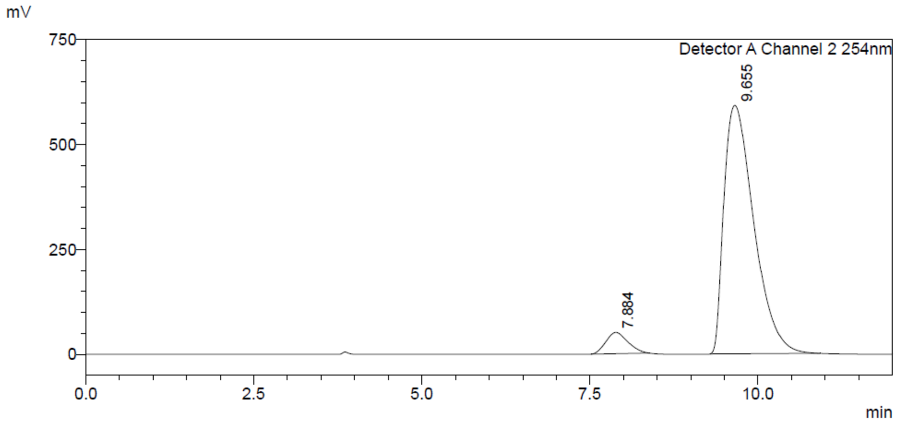

<Peak Table>

Detector A Channel 2 254nm

Peak#	Ret. Time	Area%
1	7.884	5.937
2	9.655	94.063
Total		100.000

HPLC Data for **12**: **Chiral HPLC analysis**, Chiralcel OD-H (99.5:0.5 hexane:IPA, flow rate 1.0 mLmin^{-1}, 254 nm, 30 °C), t_R (minor): 29.0 min, t_R (major): 35.4 min, 93:7 er.

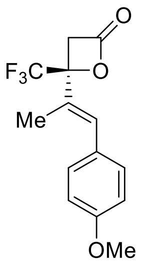

12

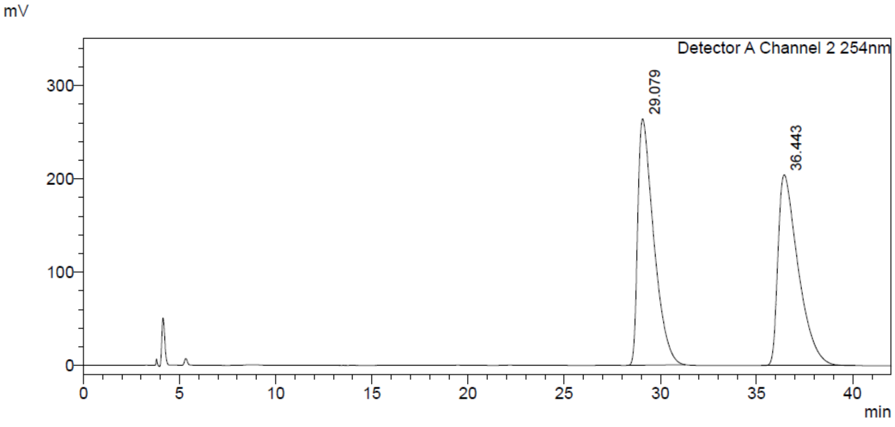

<Peak Table>

Detector A Channel 2 254nm

Peak#	Ret. Time	Area%
1	29.079	50.492
2	36.443	49.508
Total		100.000

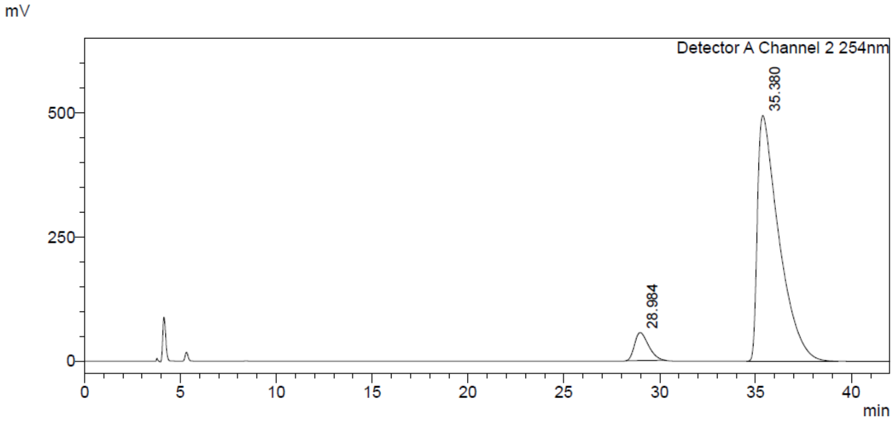

<Peak Table>

Detector A Channel 2 254nm

Peak#	Ret. Time	Area%
1	28.984	6.907
2	35.380	93.093
Total		100.000

HPLC Data for **13**: **Chiral HPLC analysis**, Chiralpak IB (99.5:0.5 hexane:IPA, flow rate 1.0 mLmin^{-1}, 254 nm, 30 °C), t_R (major): 16.9 min, t_R (minor): 20.7 min, 91:9 er.

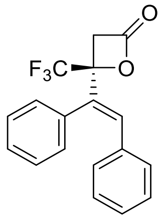

13

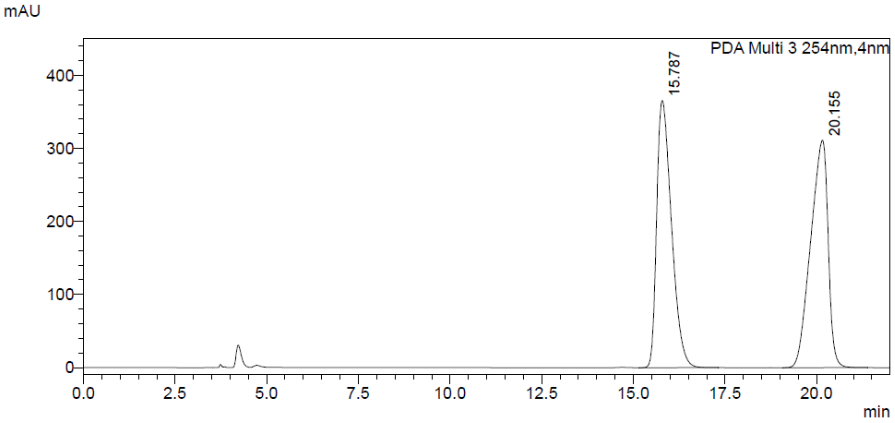

<Peak Table>

PDA Ch3 254nm

Peak#	Ret. Time	Area%
1	15.787	49.941
2	20.155	50.059
Total		100.000

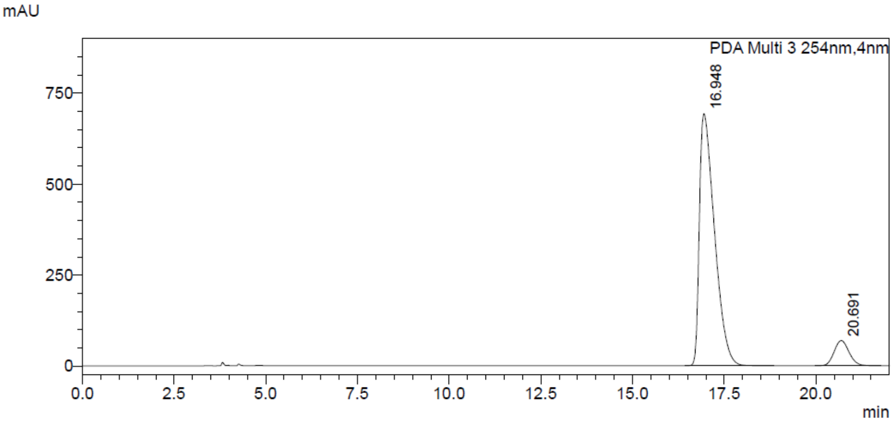

<Peak Table>

PDA Ch3 254nm

Peak#	Ret. Time	Area%
1	16.948	90.937
2	20.691	9.063
Total		100.000

HPLC Data for **15**: **major diastereoisomer**: Chiralpak IB (99.5:0.5 hexane:IPA, flow rate 0.7 mLmin^{-1}, 254 nm, 30 °C), t_R (minor): 7.3 min, t_R (major): 9.2 min, 99:1 er.

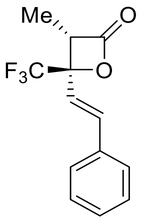

15

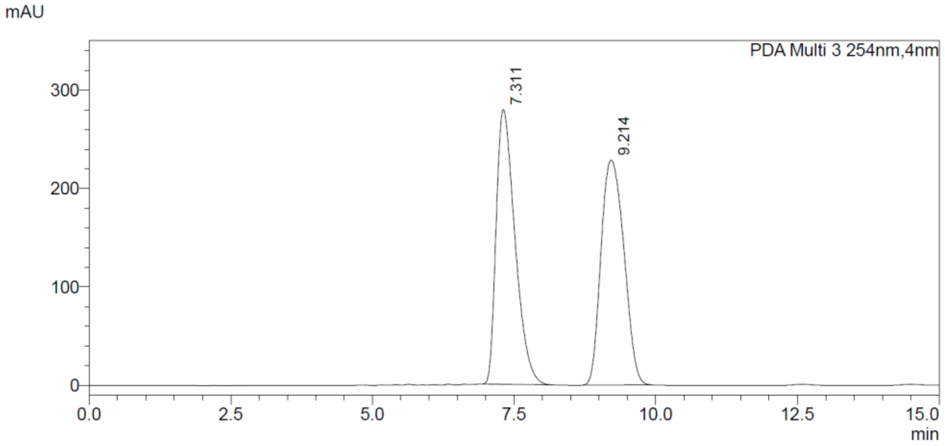

<Peak Table>

PDA Ch3 254nm

Peak#	Ret. Time	Area%
1	7.311	49.988
2	9.214	50.012
Total		100.000

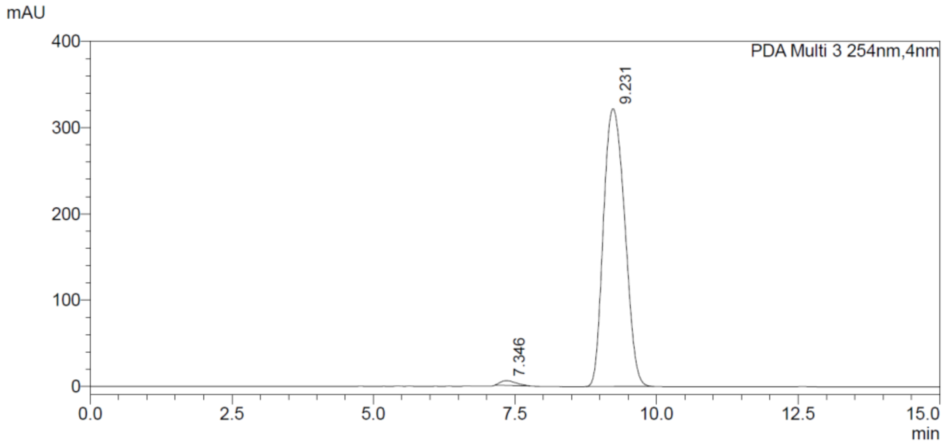

<Peak Table>

PDA Ch3 254nm

Peak#	Ret. Time	Area%
1	7.346	1.219
2	9.231	98.781
Total		100.000

HPLC Data for **15**: **Minor diastereoisomer**: Chiralpak IB (99.5:0.5 hexane:IPA, flow rate 0.7 mLmin^{-1}, 211 nm, 30 °C), t_R (major): 12.1 min, t_R (minor): 13.9 min, 77:23 er.

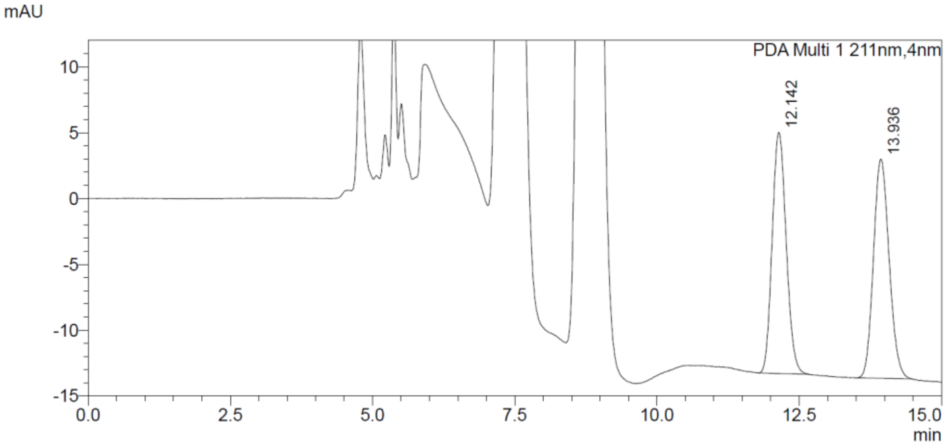

<Peak Table>

PDA Ch1 211nm

Peak#	Ret. Time	Area%
1	12.142	49.603
2	13.936	50.397
Total		100.000

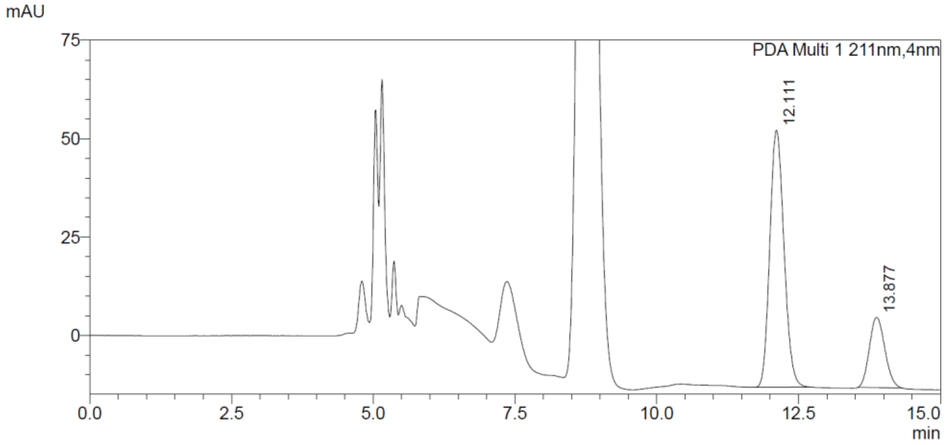

<Peak Table>

PDA Ch1 211nm

Peak#	Ret. Time	Area%
1	12.111	77.036
2	13.877	22.964
Total		100.000

HPLC Data for **16**: **Chiral HPLC analysis**, Chiralcel OJ-H (99.5:0.5 hexane:IPA, flow rate 1.0 mLmin^{-1}, 254 nm, 30 °C), t_R(minor): 15.3 min, t_R(major): 25.6 min, 99:1 er.

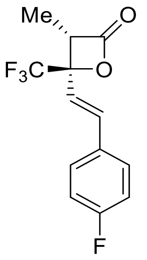

16

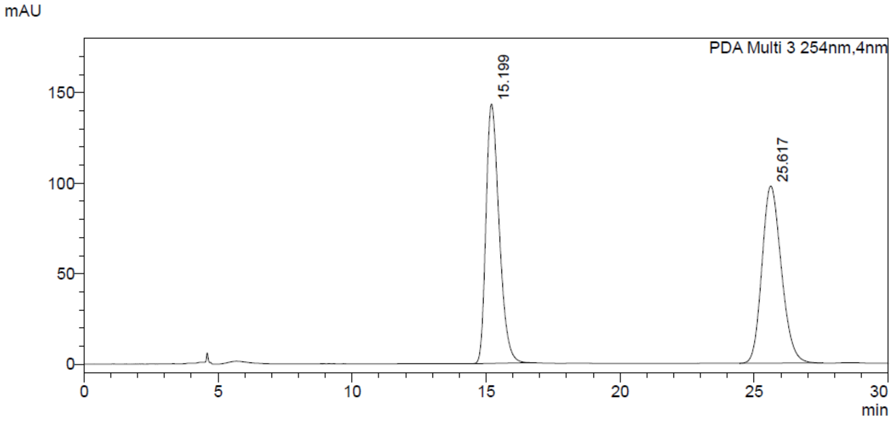

<Peak Table>

PDA Ch3 254nm

Peak#	Ret. Time	Area%
1	15.199	49.809
2	25.617	50.191
Total		100.000

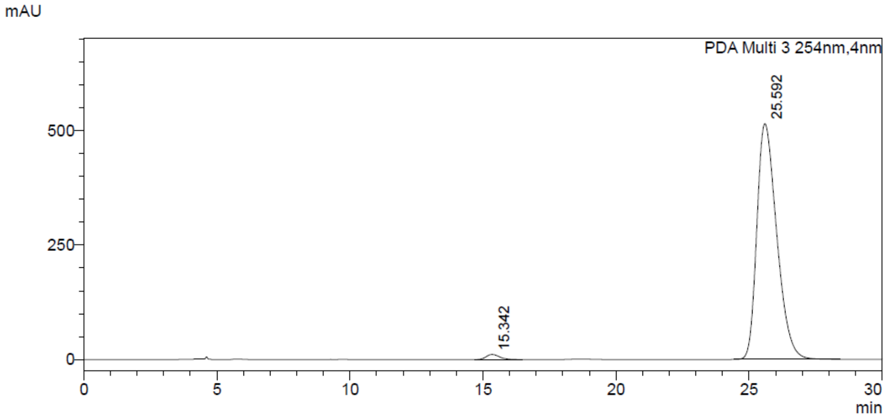

<Peak Table>

PDA Ch3 254nm

Peak#	Ret. Time	Area%
1	15.342	1.420
2	25.592	98.580
Total		100.000

HPLC Data for **17**: **Chiral HPLC analysis**, Chiralcel OJ-H (99.5:0.5 hexane:IPA, flow rate 1.0 mLmin^{-1}, 254 nm, 30 °C), t_R (minor): 22.4 min, t_R (major): 34.0 min, 99:1 er.

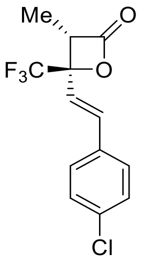

17

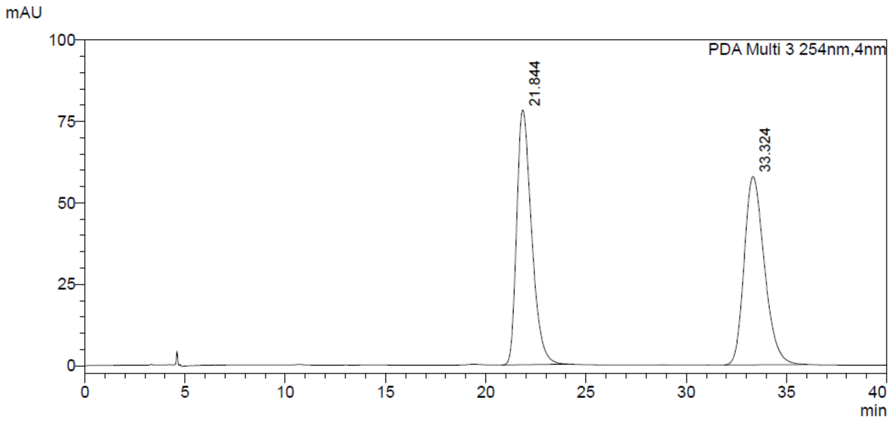

<Peak Table>

PDA Ch3 254nm

Peak#	Ret. Time	Area%
1	21.844	49.912
2	33.324	50.088
Total		100.000

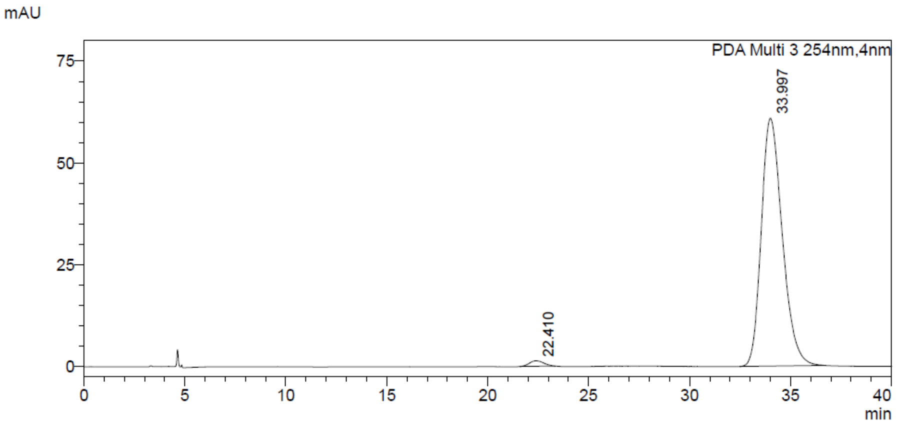

<Peak Table>

PDA Ch3 254nm

Peak#	Ret. Time	Area%
1	22.410	1.499
2	33.997	98.501
Total		100.000

HPLC Data for **18: Chiral HPLC analysis**, Chiralcel OJ-H (99.5:0.5 hexane:IPA, flow rate 1.0 mLmin^{-1}, 254 nm, 30 °C), t_R (minor): 28.6 min, t_R (major): 42.1 min, >99:1 er.

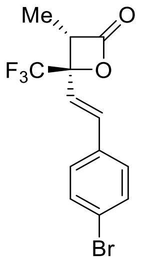

18

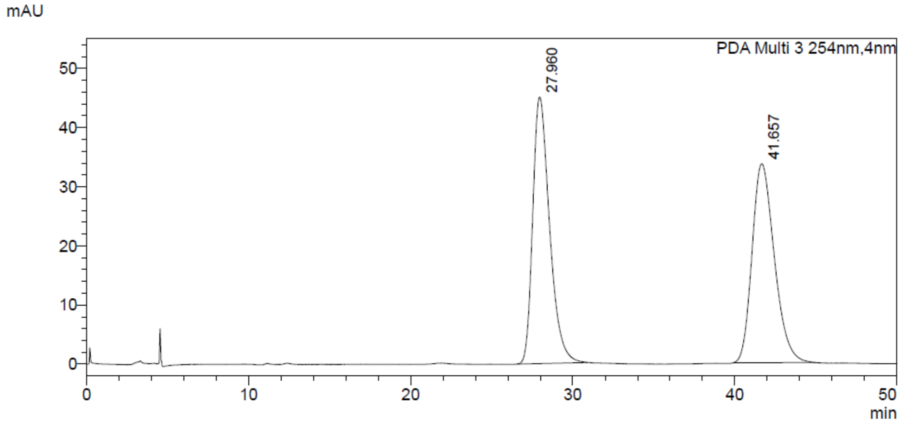

<Peak Table>

PDA Ch3 254nm

Peak#	Ret. Time	Area%
1	27.960	50.228
2	41.657	49.772
Total		100.000

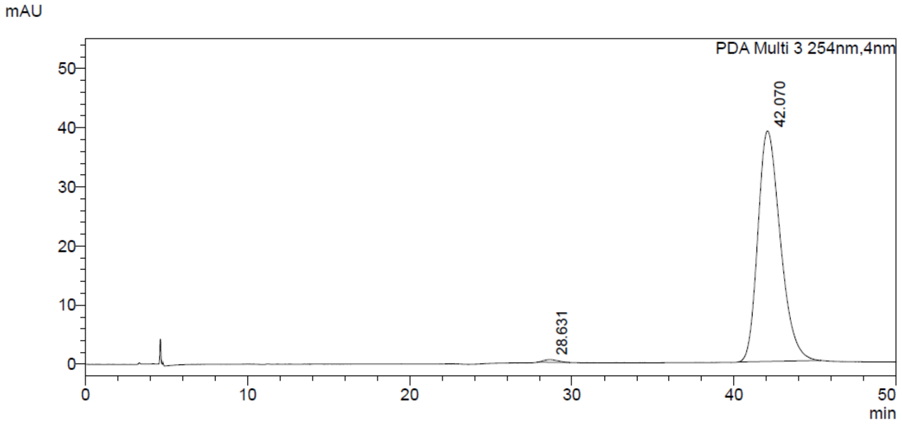

<Peak Table>

PDA Ch3 254nm

Peak#	Ret. Time	Area%
1	28.631	0.755
2	42.070	99.245
Total		100.000

HPLC Data for **19**: **Chiral HPLC analysis**, Chiralcel OJ-H (99.5:0.5 hexane:IPA, flow rate 1.0 mLmin^{-1}, 254 nm, 30 °C), t_R (minor): 18.1 min, t_R (major): 20.7 min, 99:1 er.

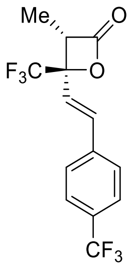

19

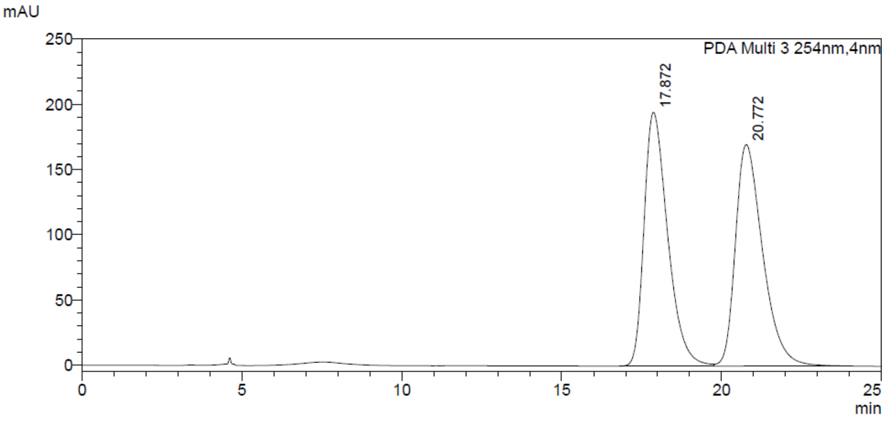

<Peak Table>

PDA Ch3 254nm

Peak#	Ret. Time	Area%
1	17.872	49.846
2	20.772	50.154
Total		100.000

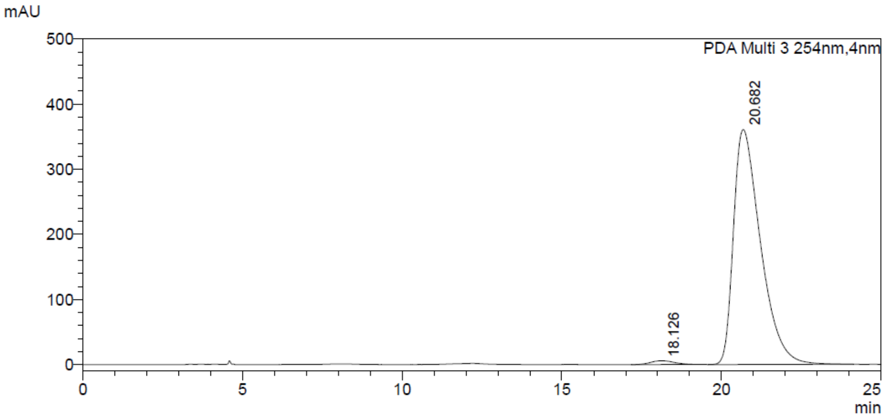

<Peak Table>

PDA Ch3 254nm

Peak#	Ret. Time	Area%
1	18.126	1.429
2	20.682	98.571
Total		100.000

HPLC Data for **20**: **Chiral HPLC analysis**, Chiralcel OJ-H (99.5:0.5 hexane:IPA, flow rate 1.0 mLmin^{-1}, 254 nm, 30 °C), t_R (minor): 21.5 min, t_R (major): 33.8 min, >99:1 er.

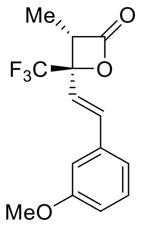

20

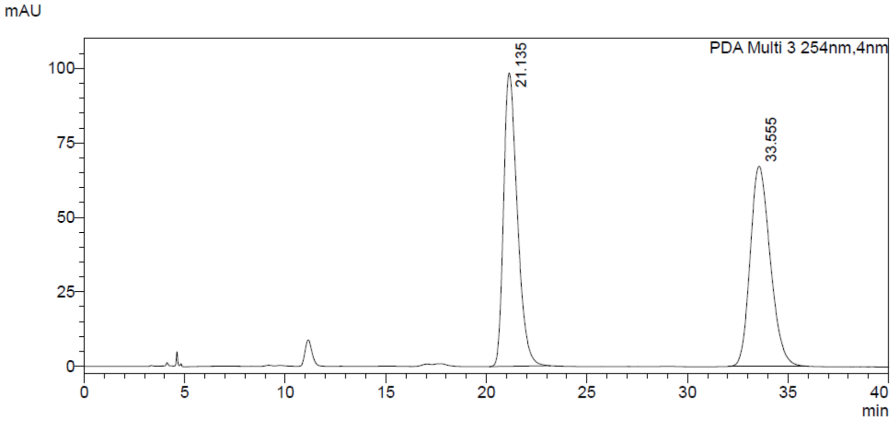

<Peak Table>

PDA Ch3 254nm

Peak#	Ret. Time	Area%
1	21.135	50.034
2	33.555	49.966
Total		100.000

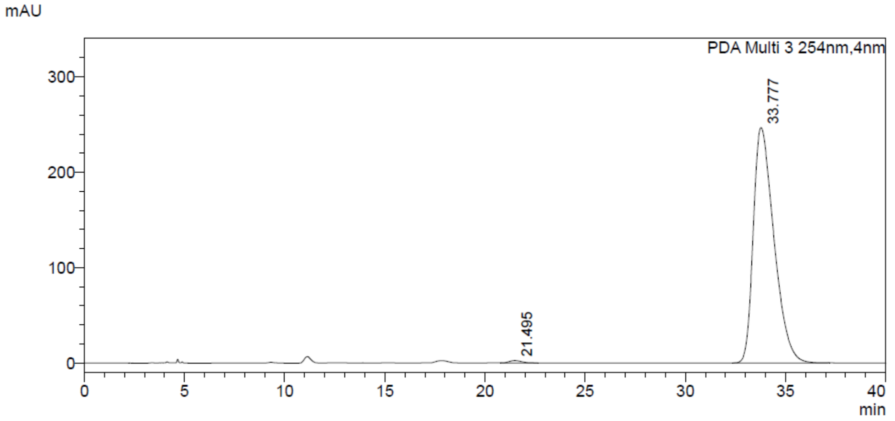

<Peak Table>

PDA Ch3 254nm

Peak#	Ret. Time	Area%
1	21.495	0.656
2	33.777	99.344
Total		100.000

HPLC Data for **21**: **Chiral HPLC analysis**, Chiralcel OJ-H (99.5:0.5 hexane:IPA, flow rate 1.0 mLmin^{-1}, 254 nm, 30 °C), t_R (minor): 10.2 min, t_R (major): 15.2 min, >99:1 er.

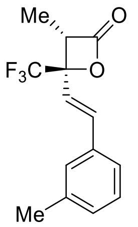

21

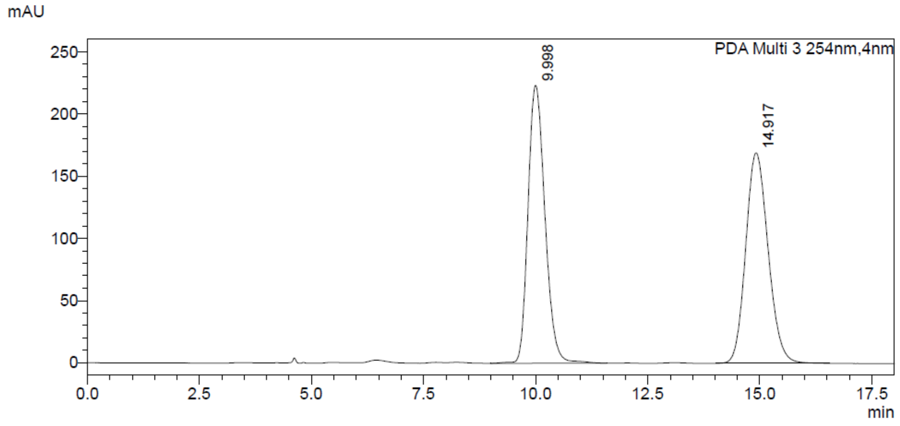

<Peak Table>

PDA Ch3 254nm

Peak#	Ret. Time	Area%
1	9.998	49.969
2	14.917	50.031
Total		100.000

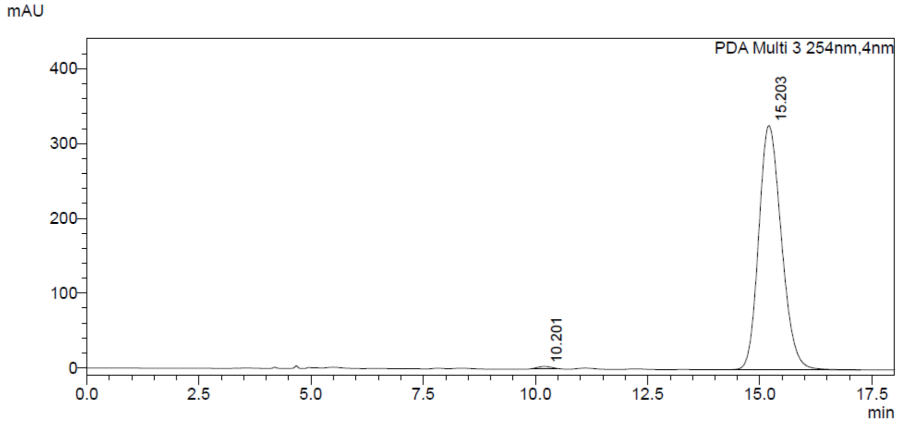

<Peak Table>

PDA Ch3 254nm

Peak#	Ret. Time	Area%
1	10.201	0.449
2	15.203	99.551
Total		100.000

HPLC Data for **22**: **Chiral HPLC analysis**, Chiralcel OJ-H (99.5:0.5 hexane:IPA, flow rate 1.0 mLmin^{-1}, 254 nm, 30 °C), t_R (minor): 24.2 min, t_R (major): 43.1 min, 99:1 er.

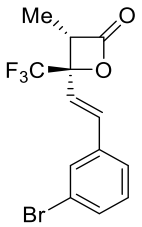

22

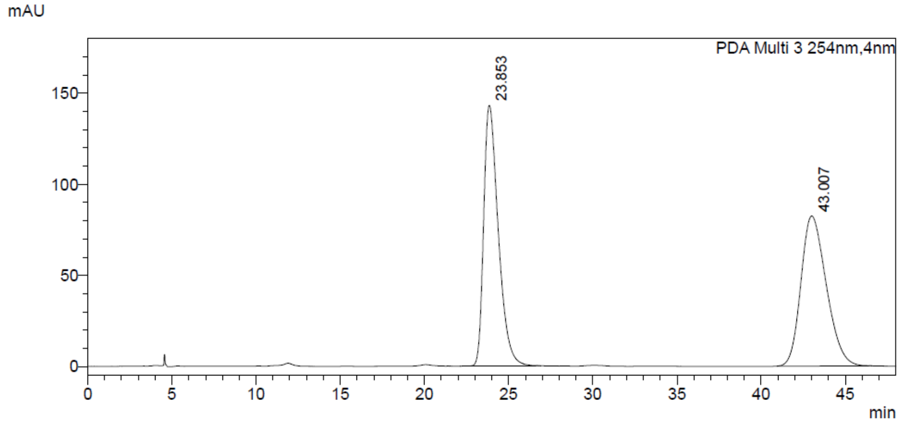

<Peak Table>

PDA Ch3 254nm

Peak#	Ret. Time	Area%
1	23.853	49.972
2	43.007	50.028
Total		100.000

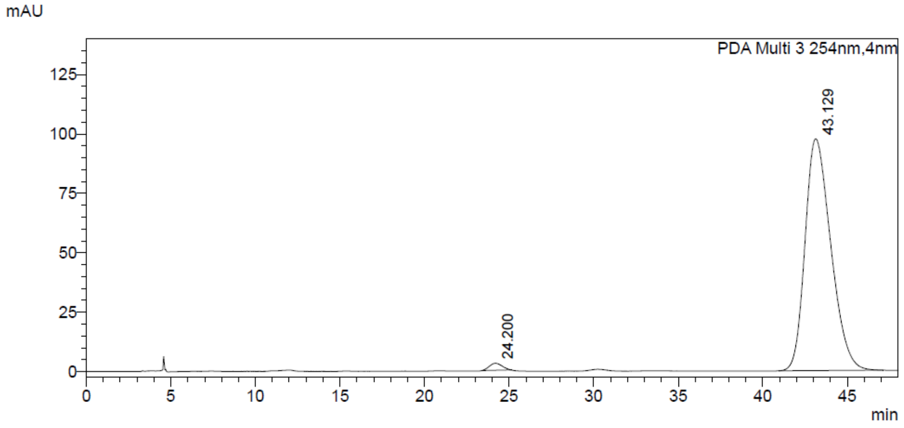

<Peak Table>

PDA Ch3 254nm

Peak#	Ret. Time	Area%
1	24.200	1.493
2	43.129	98.507
Total		100.000

HPLC Data for **23**: **Chiral HPLC analysis**, Chiralcel OJ-H (99.5:0.5 hexane:IPA, flow rate 1.0 mLmin^{-1}, 254 nm, 30 °C), t_R (minor): 20.7 min, t_R (major): 40.4 min, >99:1 er.

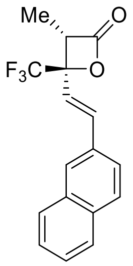

23

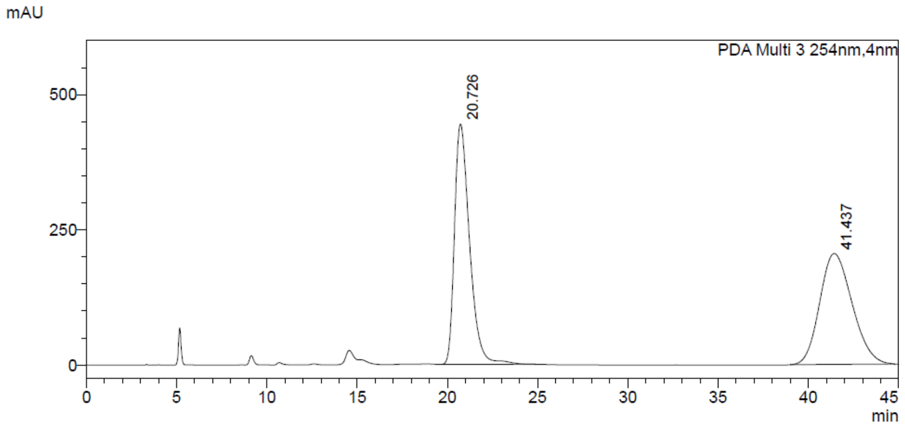

<Peak Table>

PDA Ch3 254nm

Peak#	Ret. Time	Area%
1	20.726	50.265
2	41.437	49.735
Total		100.000

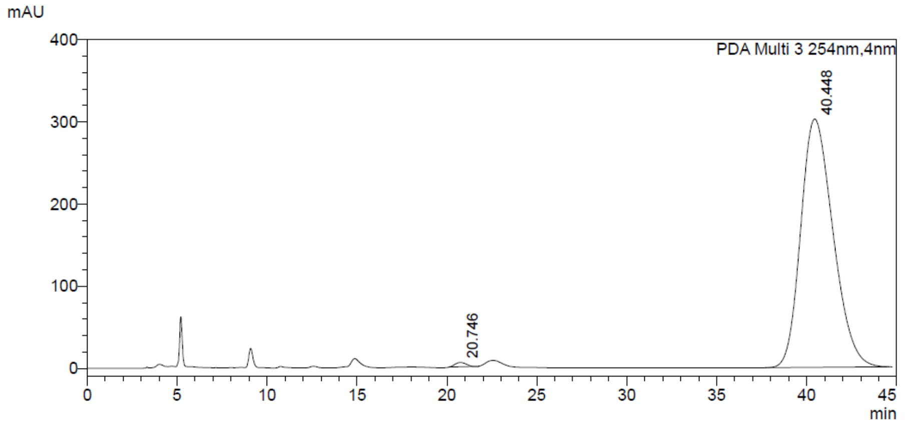

<Peak Table>

PDA Ch3 254nm

Peak#	Ret. Time	Area%
1	20.746	0.588
2	40.448	99.412
Total		100.000

HPLC Data for **24**: **Chiral HPLC analysis**, Chiralcel OJ-H (99.5:0.5 hexane:IPA, flow rate 1.0 mLmin^{-1}, 211 nm, 30 °C), t_R (minor): 23.3 min, t_R (major): 32.8 min, >99:1 er.

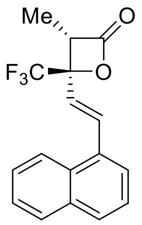

24

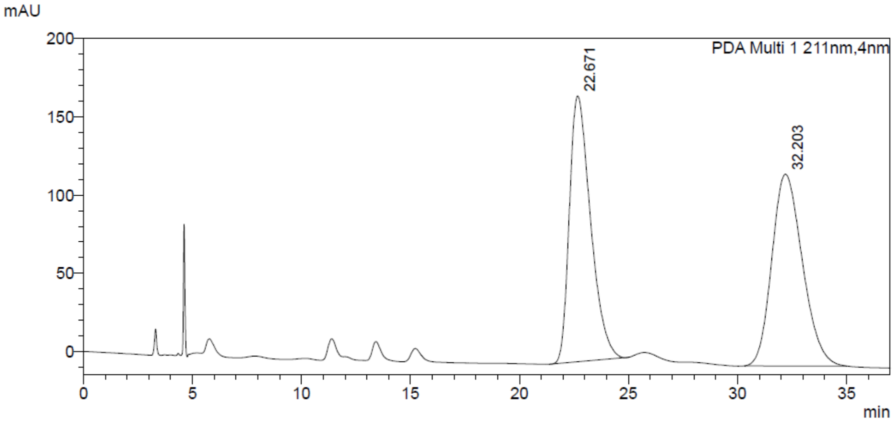

<Peak Table>

PDA Ch1 211nm

Peak#	Ret. Time	Area%
1	22.671	49.017
2	32.203	50.983
Total		100.000

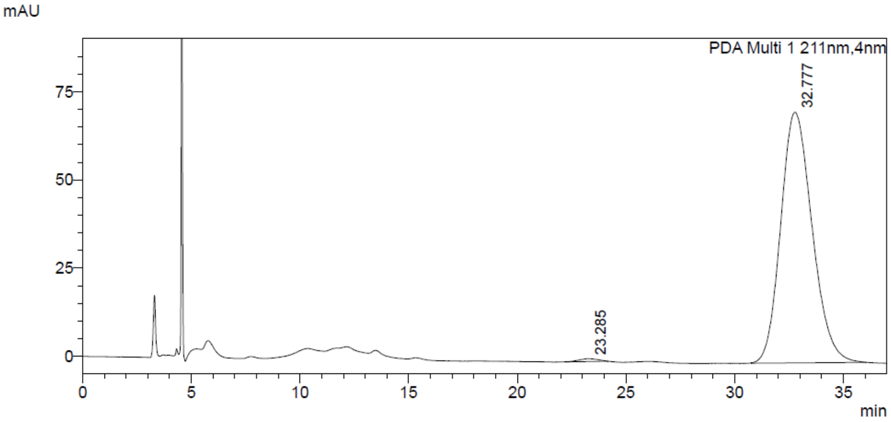

<Peak Table>

PDA Ch1 211nm

Peak#	Ret. Time	Area%
1	23.285	0.638
2	32.777	99.362
Total		100.000

HPLC Data for **25**: **Chiral HPLC analysis**, Chiralcel OD-H (99.9:0.1 hexane:IPA, flow rate 1.0 mLmin^{-1}, 254 nm, 30 °C), t_R (major): 15.0 min, >99:1 er.

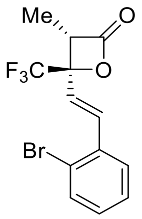

25

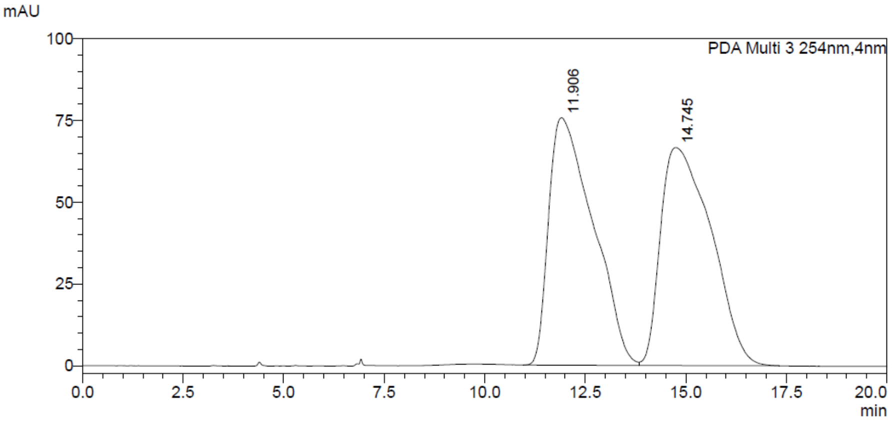

<Peak Table>

PDA Ch3 254nm

Peak#	Ret. Time	Area%
1	11.906	49.845
2	14.745	50.155
Total		100.000

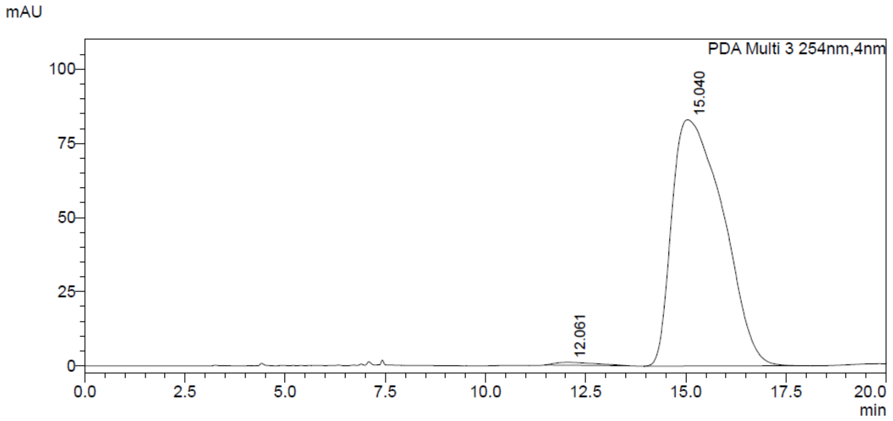

<Peak Table>

PDA Ch3 254nm

Peak#	Ret. Time	Area%
1	12.061	0.921
2	15.040	99.079
Total		100.000

HPLC Data for **26**: **Chiral HPLC analysis**, Chiralcel OJ-H (99.5:0.5 hexane:IPA, flow rate 1.0 mLmin^{-1}, 254 nm, 30 °C), t_R (minor): 9.6 min, t_R (major): 11.0 min, 98:2 er.

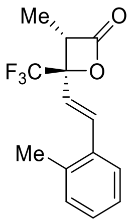

26

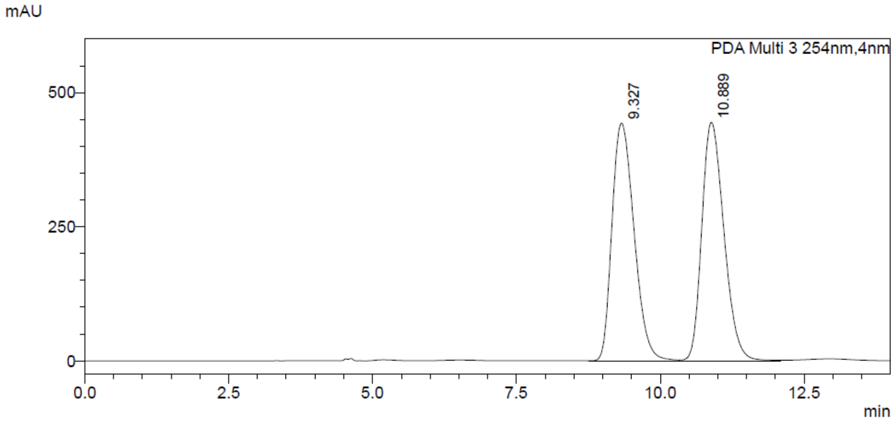

<Peak Table>

PDA Ch3 254nm

Peak#	Ret. Time	Area%
1	9.327	49.853
2	10.889	50.147
Total		100.000

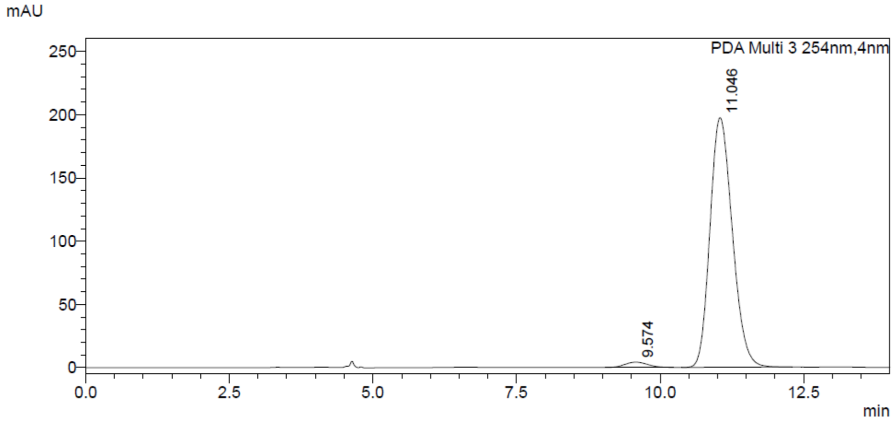

<Peak Table>

PDA Ch3 254nm

Peak#	Ret. Time	Area%
1	9.574	2.081
2	11.046	97.919
Total		100.000

HPLC Data for **27**: **Chiral HPLC analysis**, Chiralpak IB (99.5:0.5 hexane:IPA, flow rate 1.0 mLmin^{-1}, 254 nm, 30 °C), t_R (minor): 4.7 min, t_R (major): 5.4 min, 99:1 er.

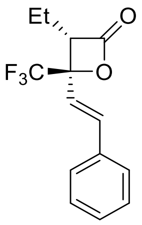

27

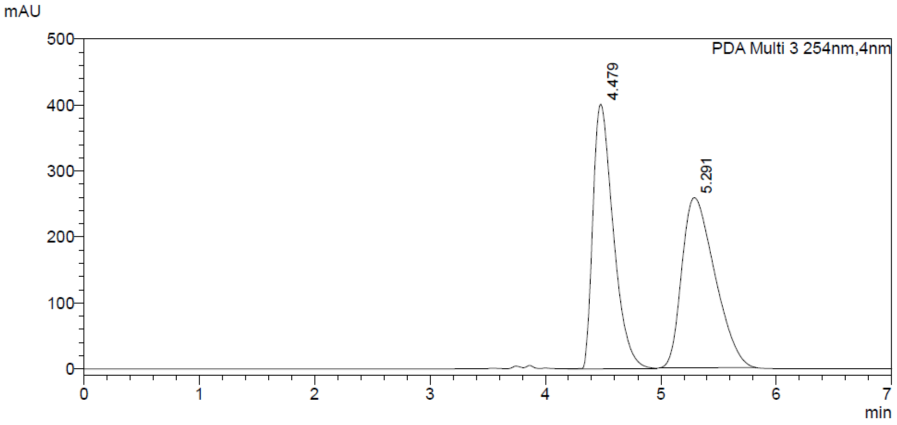

<Peak Table>

PDA Ch3 254nm

Peak#	Ret. Time	Area%
1	4.479	49.046
2	5.291	50.954
Total		100.000

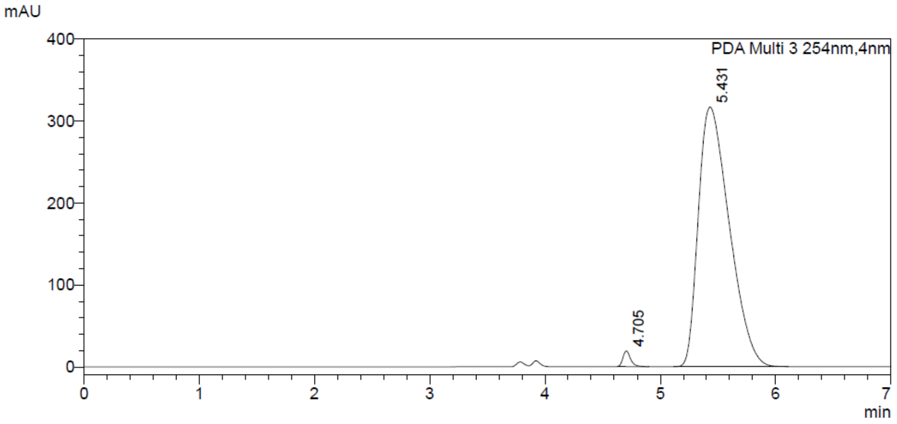

<Peak Table>

PDA Ch3 254nm

Peak#	Ret. Time	Area%
1	4.705	1.456
2	5.431	98.544
Total		100.000

HPLC Data for **28**: **Chiral HPLC analysis**, Chiralcel OJ-H (99.8:0.2 hexane:IPA, flow rate 0.5 mLmin^{-1}, 254 nm, 30 °C), t_R (major): 25.3 min, >99:1 er.

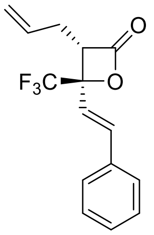

28

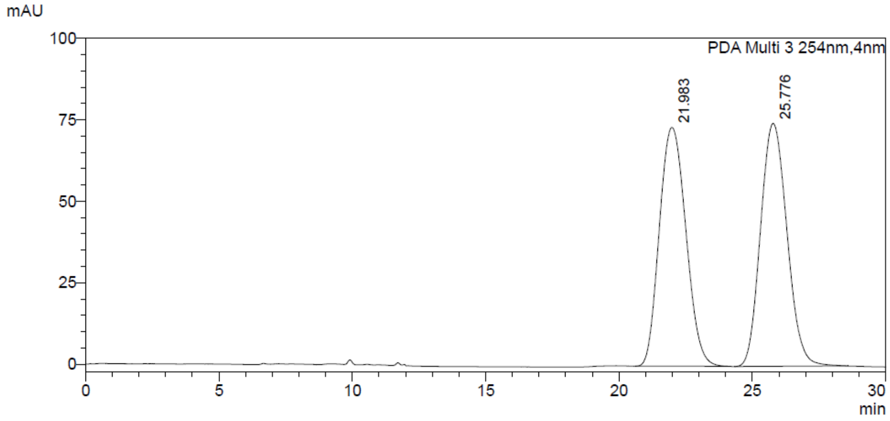

<Peak Table>

PDA Ch3 254nm

Peak#	Ret. Time	Area%
1	21.983	49.694
2	25.776	50.306
Total		100.000

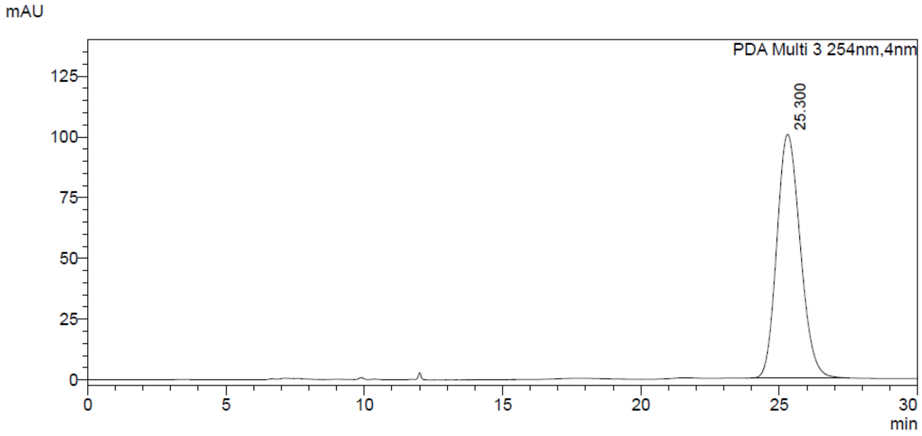

<Peak Table>

PDA Ch3 254nm

Peak#	Ret. Time	Area%
1	25.300	100.000
Total		100.000

HPLC Data for **29**: **Chiral HPLC analysis**, Chiralcel OD-H (99.5:0.5 hexane:IPA, flow rate 1.0 mLmin^{-1}, 254 nm, 30 °C), t_R (minor): 7.4 min, t_R (major): 10.0 min, 98:2 er.

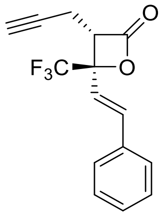

29

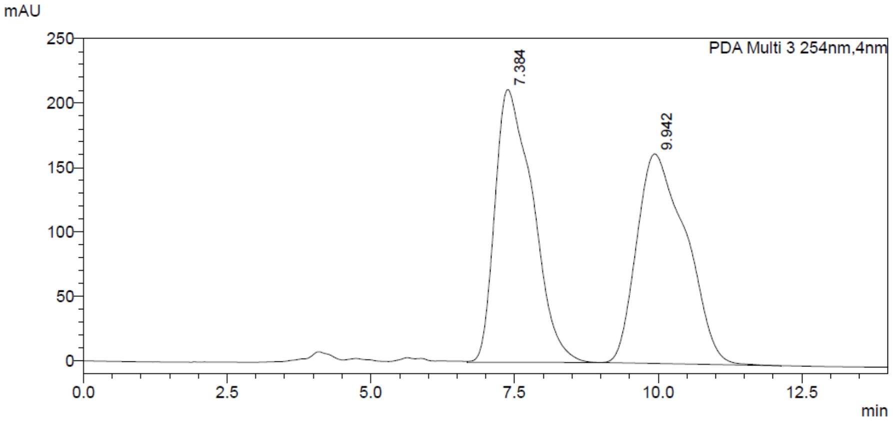

<Peak Table>

PDA Ch3 254nm

Peak#	Ret. Time	Area%
1	7.384	50.007
2	9.942	49.993
Total		100.000

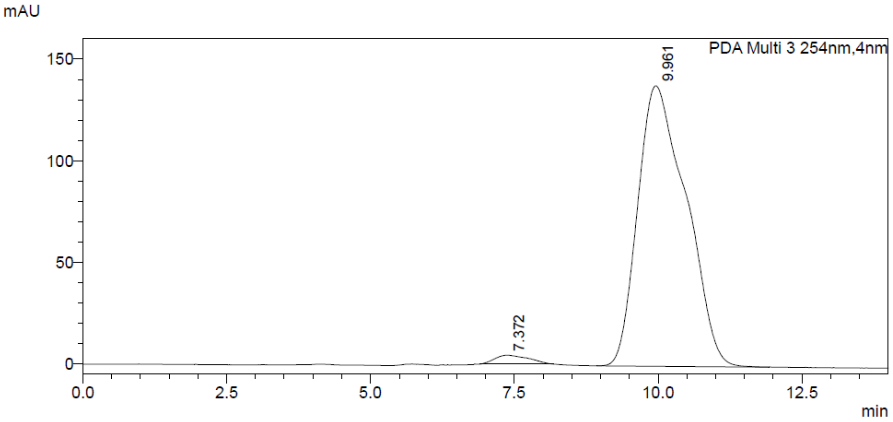

<Peak Table>

PDA Ch3 254nm

Peak#	Ret. Time	Area%
1	7.372	2.152
2	9.961	97.848
Total		100.000

HPLC Data for **30**: **Chiral HPLC analysis**, Chiralpak IB (99.5:0.5 hexane:IPA, flow rate 0.7 mLmin^{-1}, 254 nm, 30 °C), t_R (minor): 16.7min, t_R (major):22.4min, 99:1 er.

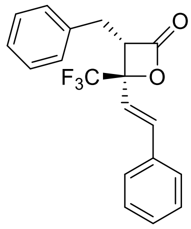

30

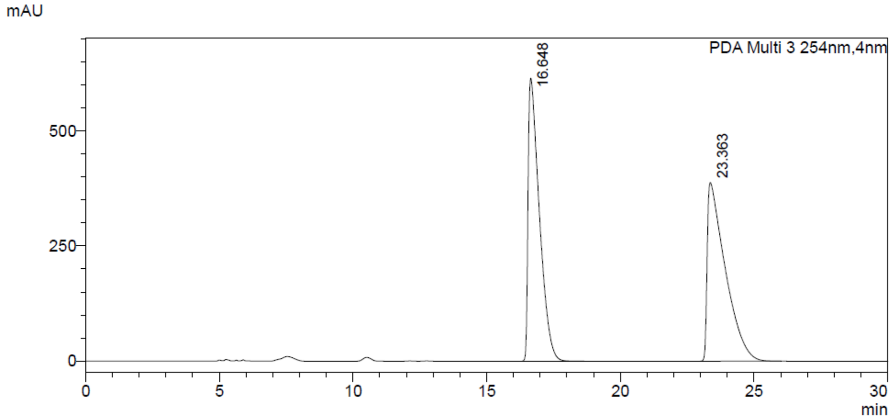

<Peak Table>

PDA Ch3 254nm

Peak#	Ret. Time	Area%
1	16.648	49.712
2	23.363	50.288
Total		100.000

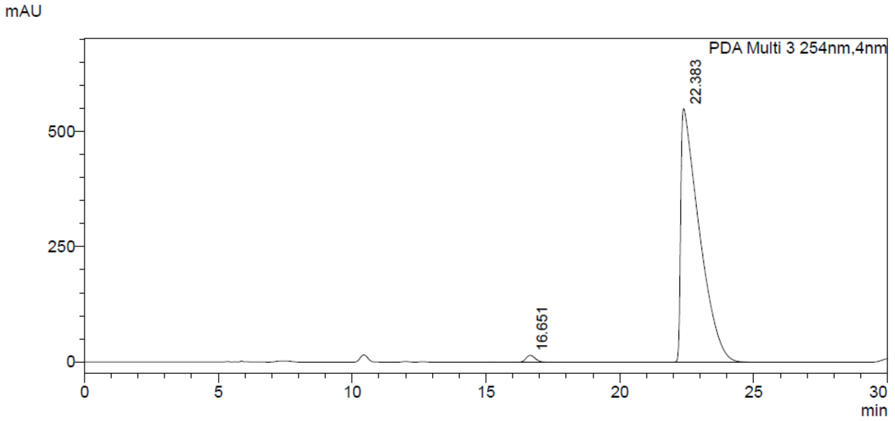

<Peak Table>

PDA Ch3 254nm

Peak#	Ret. Time	Area%
1	16.651	1.354
2	22.383	98.646
Total		100.000

HPLC Data for **31**: **Chiral HPLC analysis**, Chiralcel OD-H (95:5 hexane:IPA, flow rate 1.0 mLmin^{-1}, 254 nm, 30 °C), t_R (minor): 19.4 min, t_R (major): 34.5 min, 95:5 er.

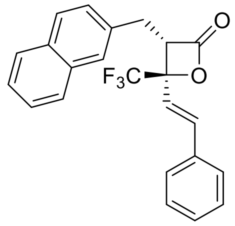

31

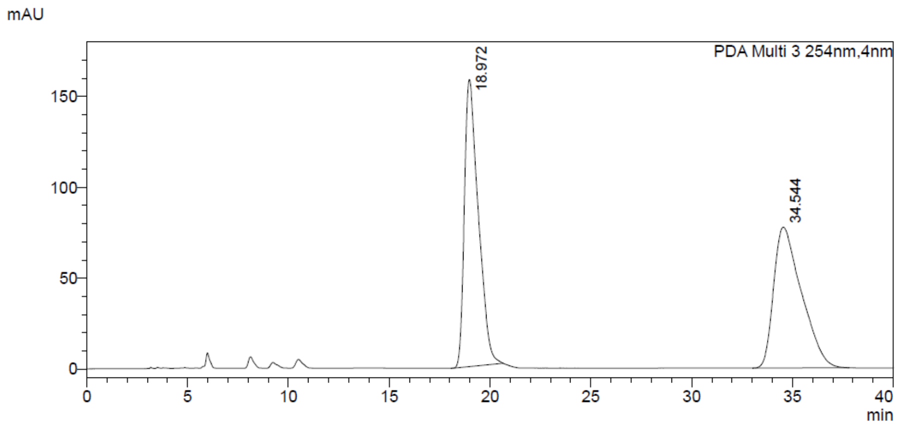

<Peak Table>

PDA Ch3 254nm

Peak#	Ret. Time	Area%
1	18.972	50.469
2	34.544	49.531
Total		100.000

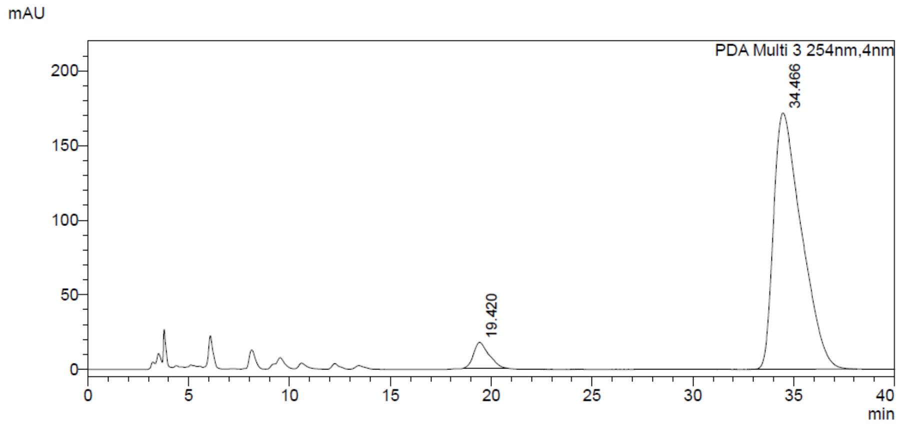

<Peak Table>

PDA Ch3 254nm

Peak#	Ret. Time	Area%
1	19.420	5.267
2	34.466	94.733
Total		100.000

HPLC Data for **32**: Chiralcel OJ-H (99.8:0.2 hexane:IPA, flow rate 1.0 mLmin^{-1}, 254 nm, 30 °C), t_R (minor): 13.1 min, t_R (major): 17.4 min, >99:1 er.

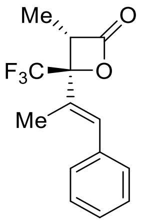

32

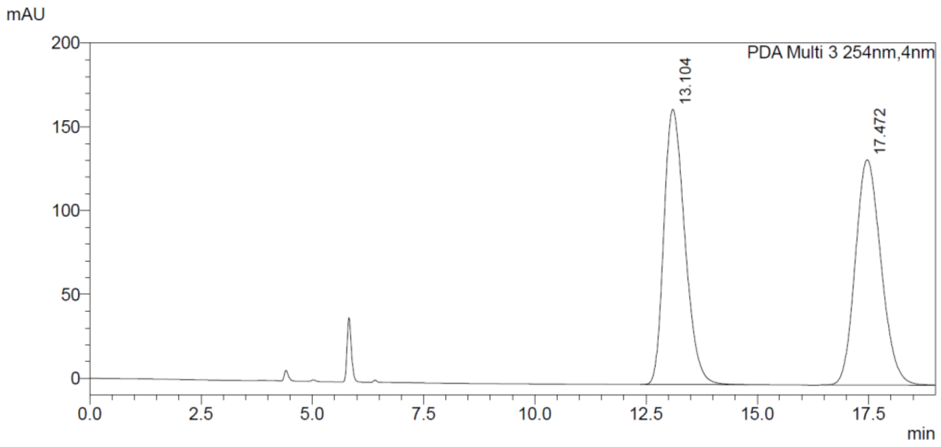

<Peak Table>

PDA Ch3 254nm

Peak#	Ret. Time	Area%
1	13.104	49.862
2	17.472	50.138
Total		100.000

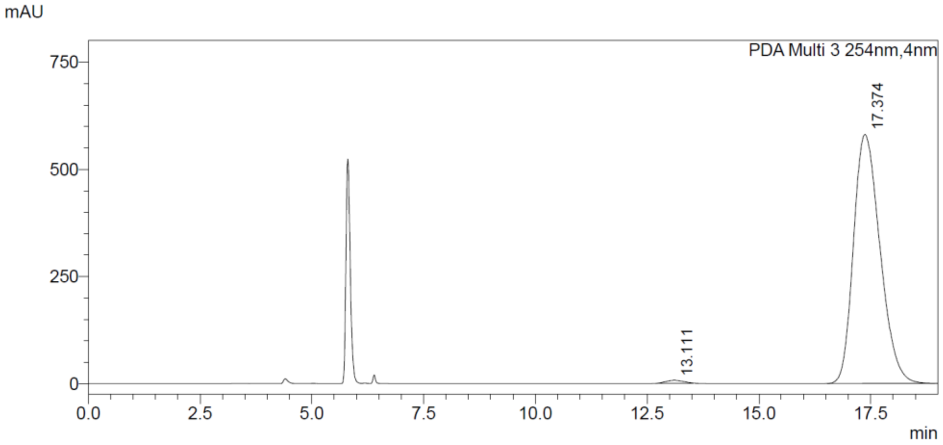

<Peak Table>

PDA Ch3 254nm

Peak#	Ret. Time	Area%
1	13.111	0.688
2	17.374	99.312
Total		100.000

HPLC Data for **33**: **Chiral HPLC analysis**, Chiralcel OJ-H (99.5:0.5 hexane:IPA, flow rate 1.0 mLmin^{-1}, 254 nm, 30 °C), t_R (minor): 24.6 min, t_R (major): 39.4 min, >99:1 er.

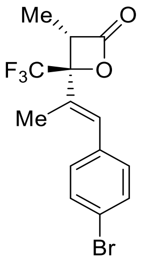

33

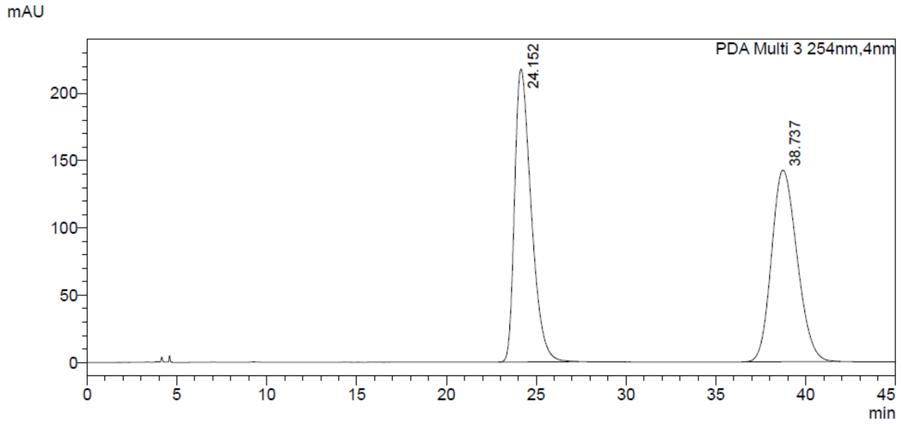

<Peak Table>

PDA Ch3 254nm

Peak#	Ret. Time	Area%
1	24.152	49.739
2	38.737	50.261
Total		100.000

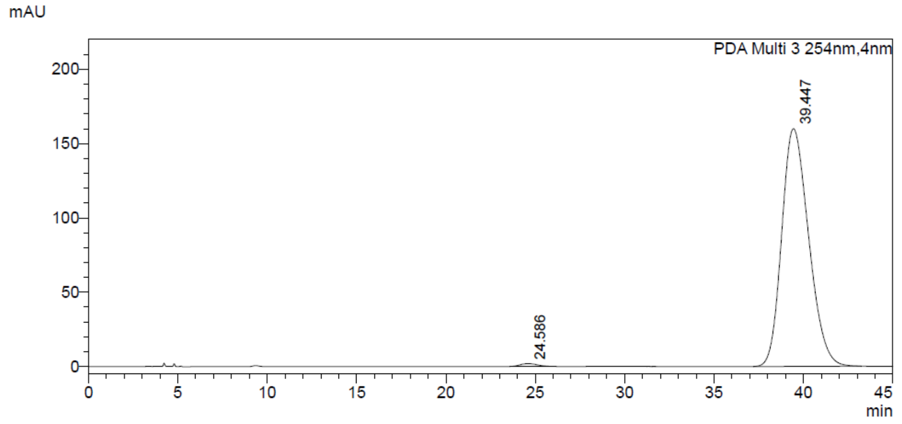

<Peak Table>

PDA Ch3 254nm

Peak#	Ret. Time	Area%
1	24.586	0.754
2	39.447	99.246
Total		100.000

HPLC Data for **34**: **Chiral HPLC analysis**, Chiralpak AS-H (99.5:0.5 hexane:IPA, flow rate 1.0 mLmin^{-1}, 254 nm, 30 °C), t_R (minor): 6.0 min, t_R (major): 7.8 min, 99:1 er.

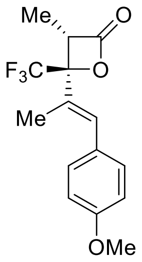

34

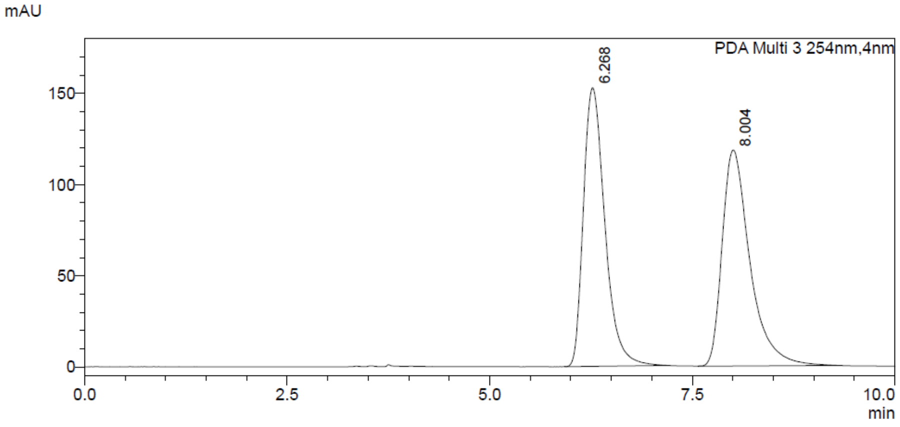

<Peak Table>

PDA Ch3 254nm

Peak#	Ret. Time	Area%
1	6.268	49.953
2	8.004	50.047
Total		100.000

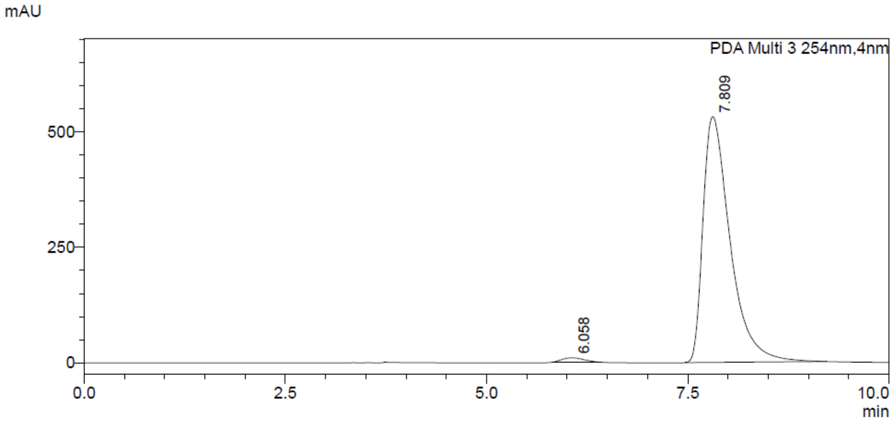

<Peak Table>

PDA Ch3 254nm

Peak#	Ret. Time	Area%
1	6.058	1.295
2	7.809	98.705
Total		100.000

HPLC Data for **35**: **Chiral HPLC analysis**, Chiralpak AD-H (99.9:0.1 hexane:IPA, flow rate 1.0 mLmin^{-1}, 254 nm, 30 °C), t_R (major): 6.4 min, >99:1 er.

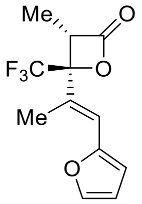

35

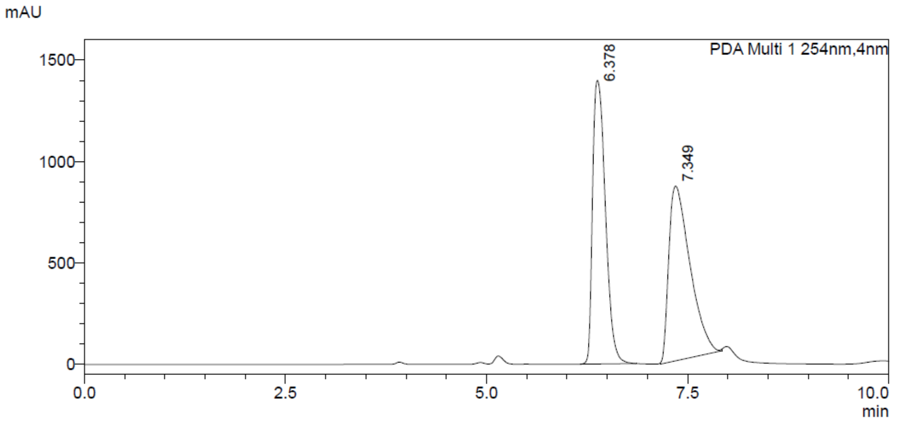

<Peak Table>

PDA Ch1 254nm

Peak#	Ret. Time	Area%
1	6.378	49.463
2	7.349	50.537
Total		100.000

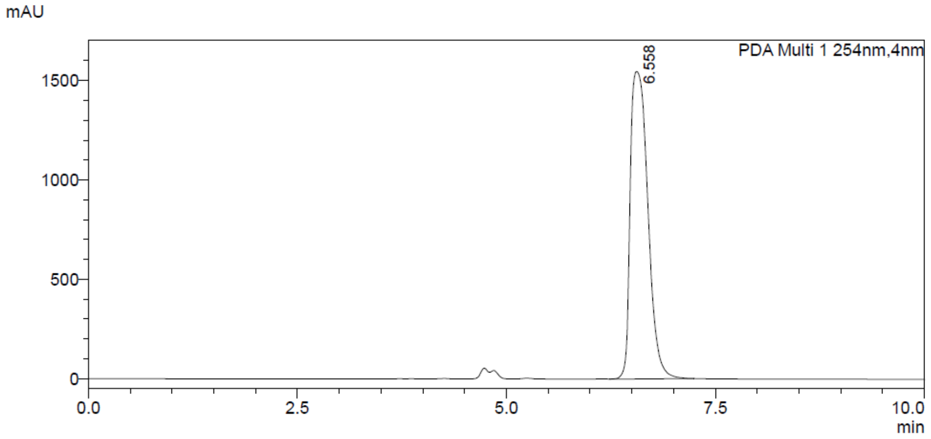

<Peak Table>

PDA Ch1 254nm

Peak#	Ret. Time	Area%
1	6.558	100.000
Total		100.000

HPLC Data for **36**: **Chiral HPLC analysis**, Chiralcel OD-H (99.9:0.1 hexane:IPA, flow rate 1.0 mLmin^{-1}, 254 nm, 30 °C), **major diastereoisomer**: t_R (major): 7.0 min, t_R (minor): 7.4 min, 97:3 er;

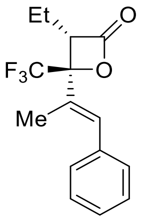

36

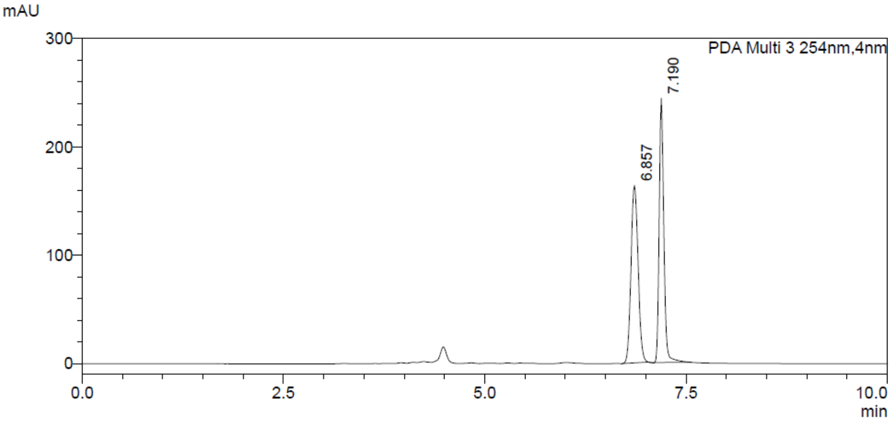

<Peak Table>

PDA Ch3 254nm

Peak#	Ret. Time	Area%
1	6.857	50.349
2	7.190	49.651
Total		100.000

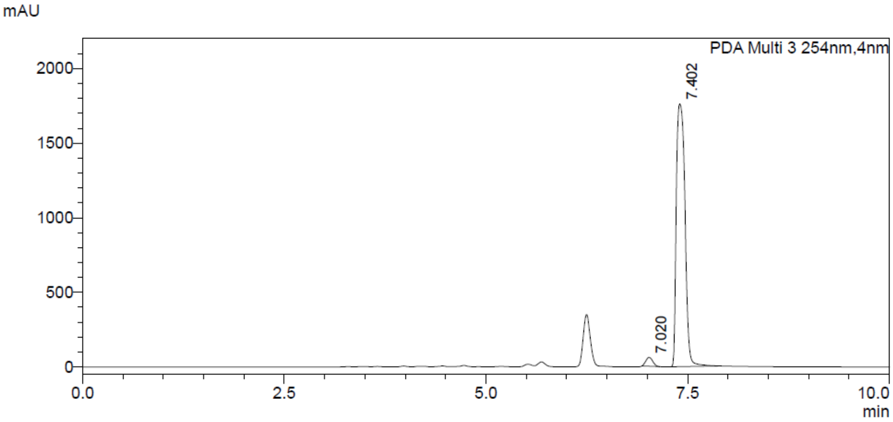

<Peak Table>

PDA Ch3 254nm

Peak#	Ret. Time	Area%
1	7.020	2.599
2	7.402	97.401
Total		100.000

minor diastereoisomer: t_R(major): 13.9 min, t_R(minor): 20.2 min,90:10 er.

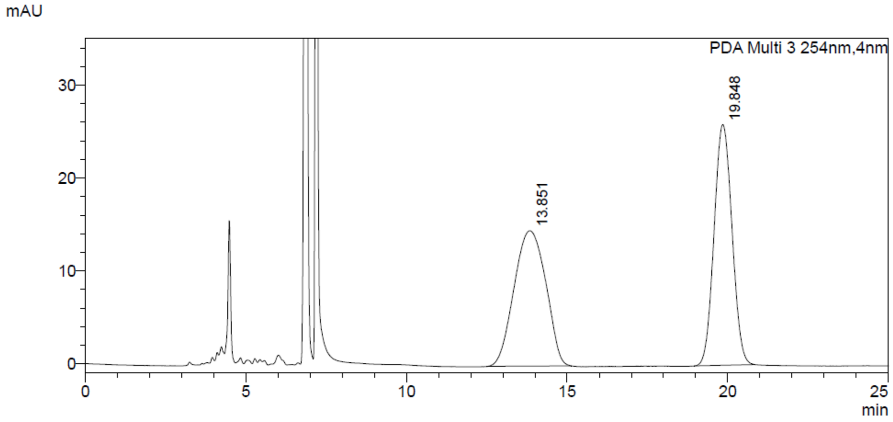

<Peak Table>

PDA Ch3 254nm

Peak#	Ret. Time	Area%
1	13.851	49.916
2	19.848	50.084
Total		100.000

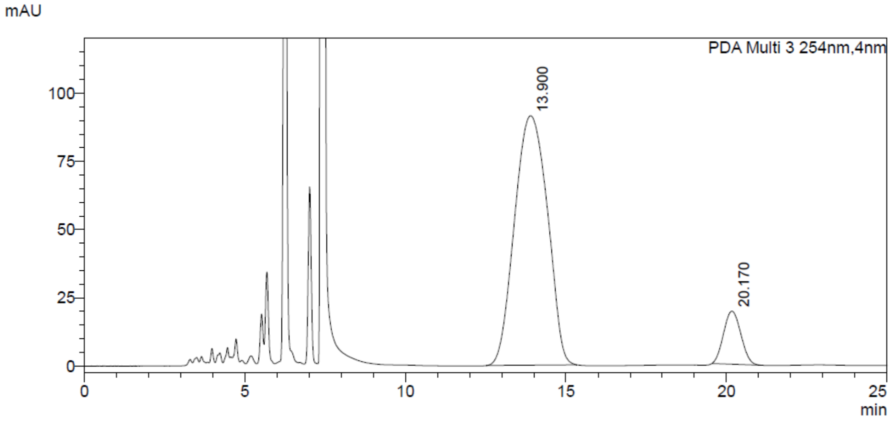

<Peak Table>

PDA Ch3 254nm

Peak#	Ret. Time	Area%
1	13.900	89.943
2	20.170	10.057
Total		100.000

HPLC Data for **37**: **Chiral HPLC analysis**, Chiralcel OJ-H (99.8:0.2 hexane:IPA, flow rate 1.0 mLmin^{-1}, 254 nm, 30 °C), t_R (minor): 10.2 min, t_R (major): 13.1 min, 99:1 er.

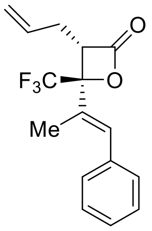

37

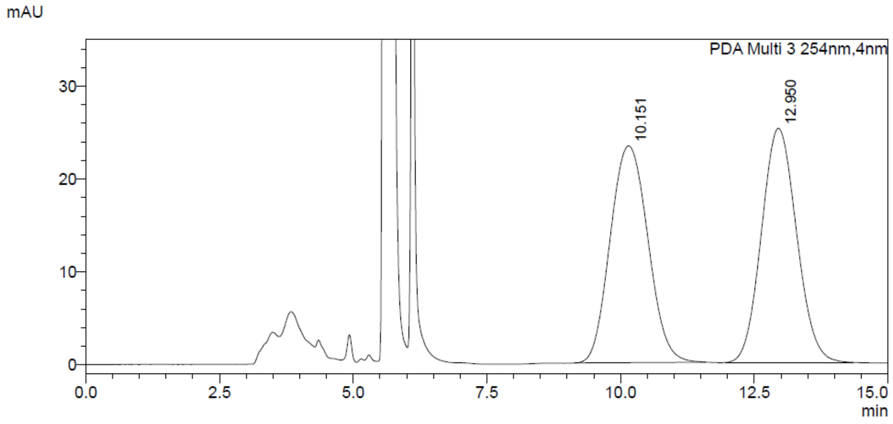

<Peak Table>

PDA Ch3 254nm

Peak#	Ret. Time	Area%
1	10.151	49.850
2	12.950	50.150
Total		100.000

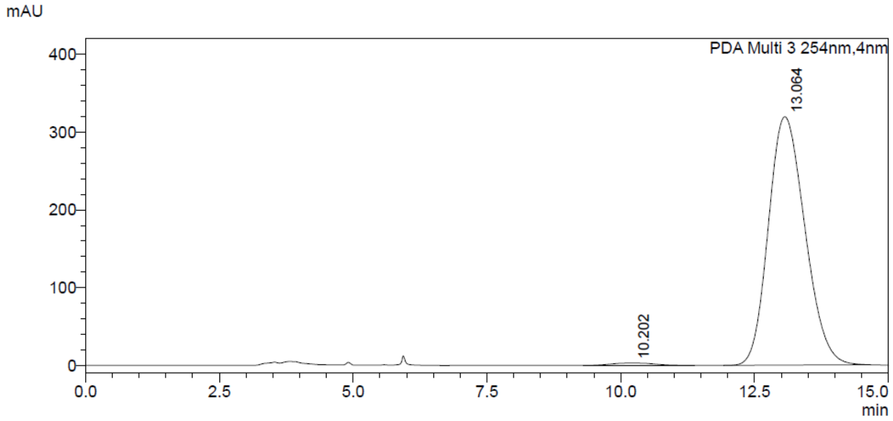

<Peak Table>

PDA Ch3 254nm

Peak#	Ret. Time	Area%
1	10.202	1.096
2	13.064	98.904
Total		100.000

HPLC Data for **38**: **Chiral HPLC analysis**, Chiralcel OJ-H (99.8:0.2 hexane:IPA, flow rate 1.0 mLmin^{-1}, 254 nm, 30 °C), **major diastereoisomer**: t_R (major): 26.5 min, t_R (minor): 31.3 min, 99:1 er;

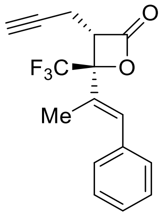

38

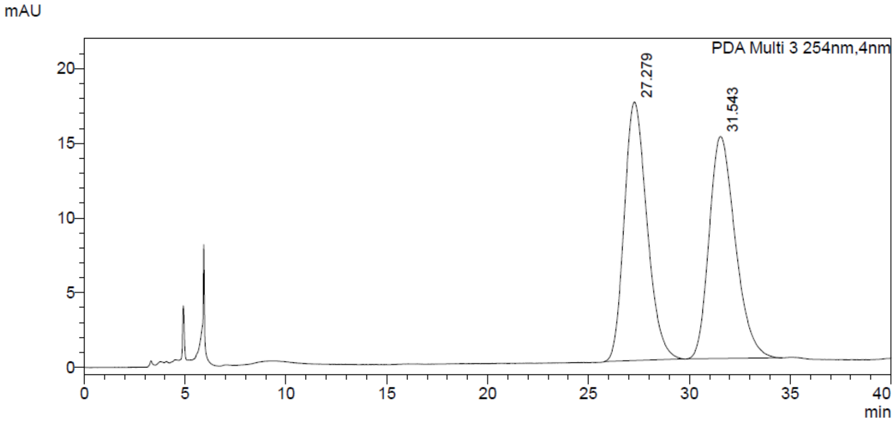

<Peak Table>

PDA Ch3 254nm

Peak#	Ret. Time	Area%
1	27.279	50.322
2	31.543	49.678
Total		100.000

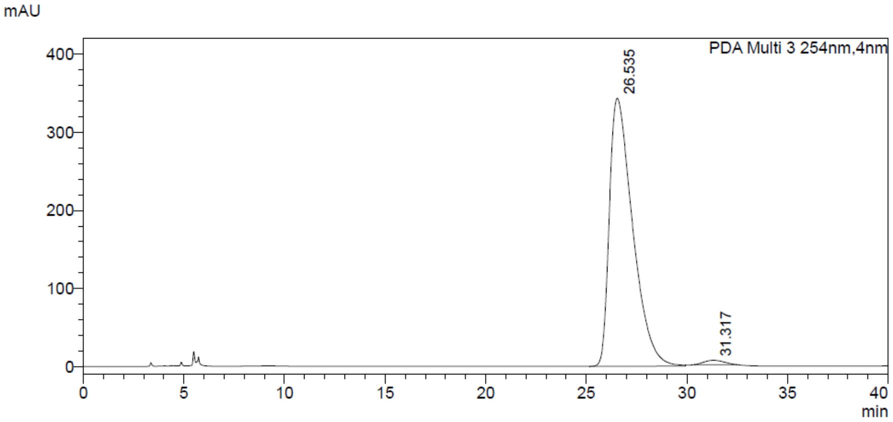

<Peak Table>

PDA Ch3 254nm

Peak#	Ret. Time	Area%
1	26.535	98.633
2	31.317	1.367
Total		100.000

minor diastereoisomer: Chiralcel OD-H (99.9:0.1 hexane:IPA, flow rate 1.0 mLmin^{-1}, 254 nm, 30 °C), t_R (minor): 24.8 min, t_R (major): 28.9 min,90:10 er.

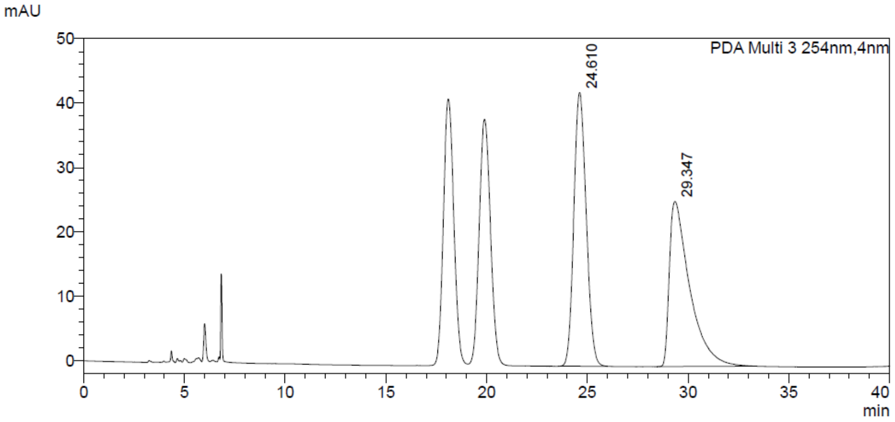

<Peak Table>

PDA Ch3 254nm

Peak#	Ret. Time	Area%
1	24.610	50.191
2	29.347	49.809
Total		100.000

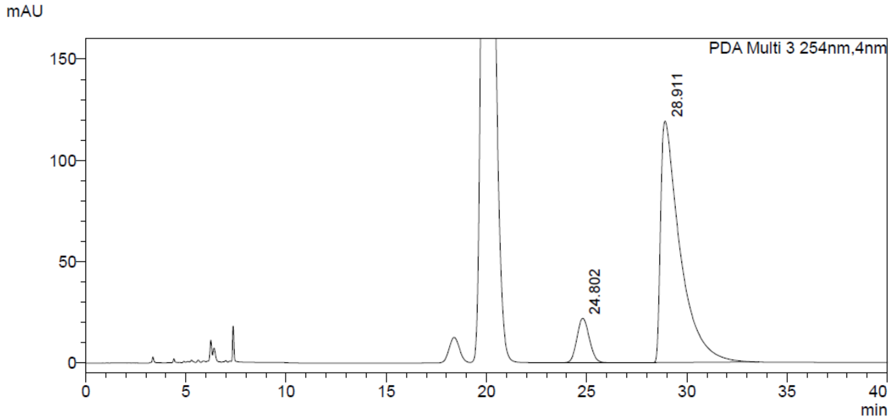

<Peak Table>

PDA Ch3 254nm

Peak#	Ret. Time	Area%
1	24.802	10.379
2	28.911	89.621
Total		100.000

HPLC Data for **39**: **Chiral HPLC analysis**, Chiralcel OD-H (99.9:0.1 hexane:IPA, flow rate 0.5 mLmin^{-1}, 211 nm, 30 °C), **major diastereoisomer**: t_R (major): 83.6 min, t_R (minor): 101.5 min, >99:1 er;

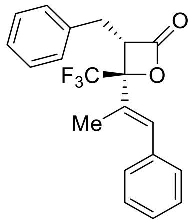

39

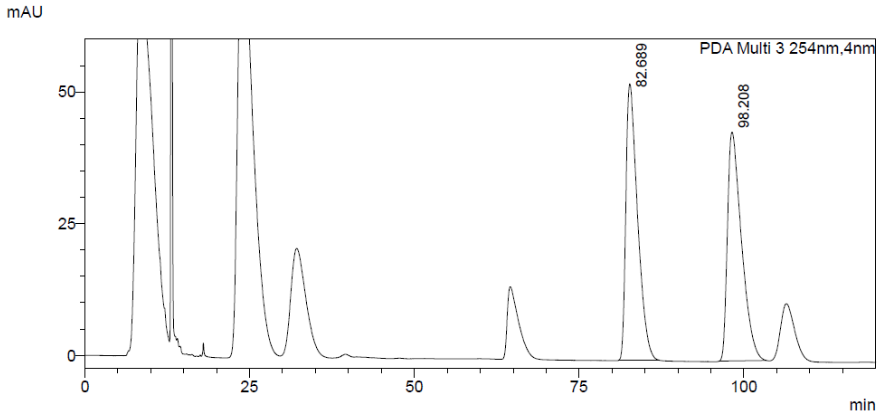

<Peak Table>

PDA Ch3 254nm

Peak#	Ret. Time	Area%
1	82.689	50.074
2	98.208	49.926
Total		100.000

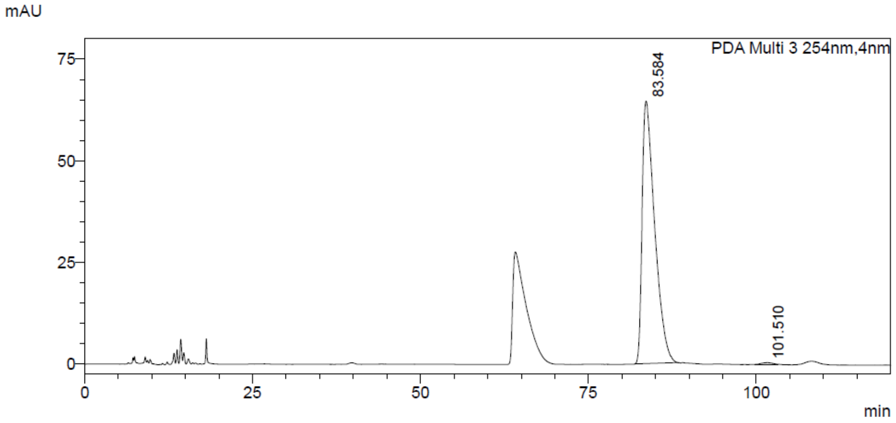

<Peak Table>

PDA Ch3 254nm

Peak#	Ret. Time	Area%
1	83.584	99.443
2	101.510	0.557
Total		100.000

minor diastereoisomer: Chiralcel OD-H (99.9:0.1 hexane:IPA, flow rate 0.5 mLmin^{-1}, 254 nm, 30 °C), t_R(major): 64.1 min, t_R(minor): 108.3 min,97:3 er.

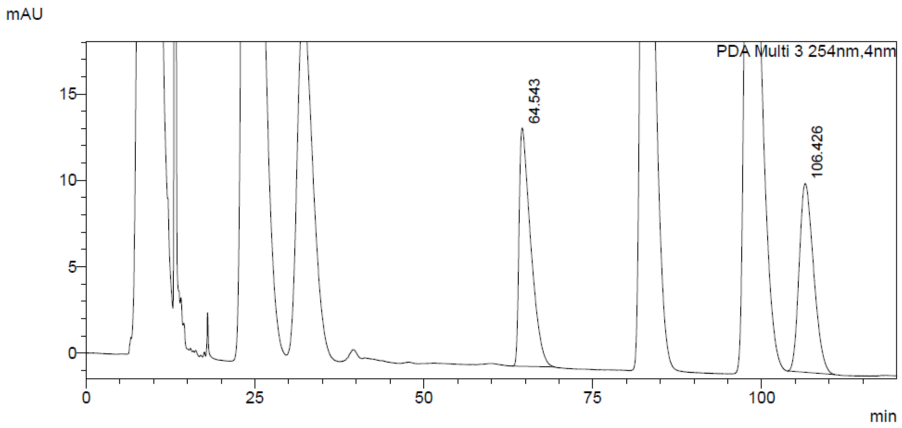

<Peak Table>

PDA Ch3 254nm

Peak#	Ret. Time	Area%
1	64.543	50.412
2	106.426	49.588
Total		100.000

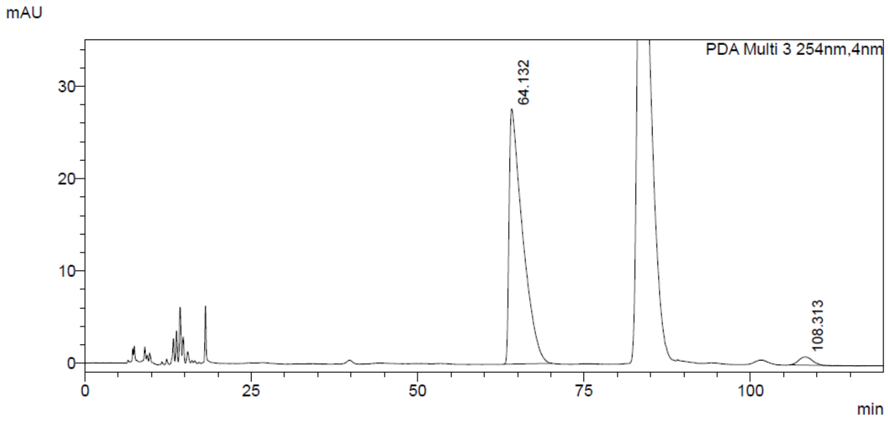

<Peak Table>

PDA Ch3 254nm

Peak#	Ret. Time	Area%
1	64.132	96.811
2	108.313	3.189
Total		100.000

HPLC Data for **40**: **Chiral HPLC analysis**, Chiralcel OD-H (95:5 hexane:IPA, flow rate 1.0 mLmin^{-1}, 254 nm, 30 °C), **major diastereoisomer**: t_R (major): 17.2 min, t_R (minor): 47.7 min, >99:1 er;

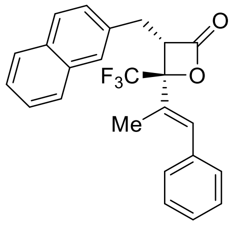

40

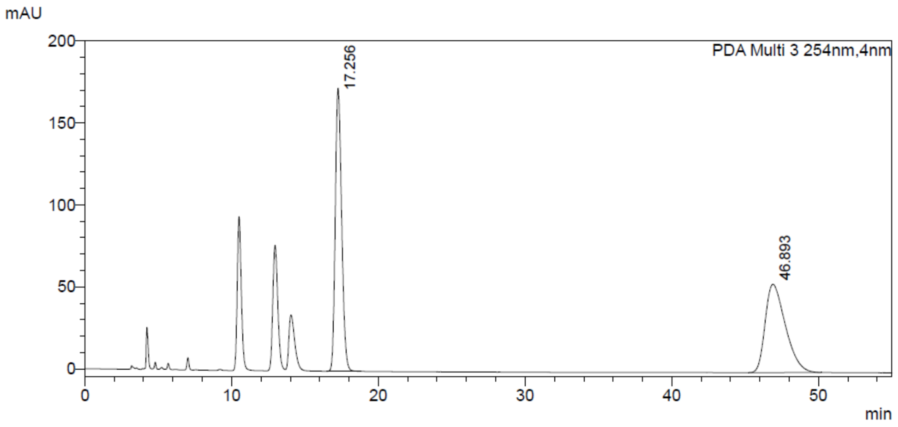

<Peak Table>

PDA Ch3 254nm

Peak#	Ret. Time	Area%
1	17.256	50.231
2	46.893	49.769
Total		100.000

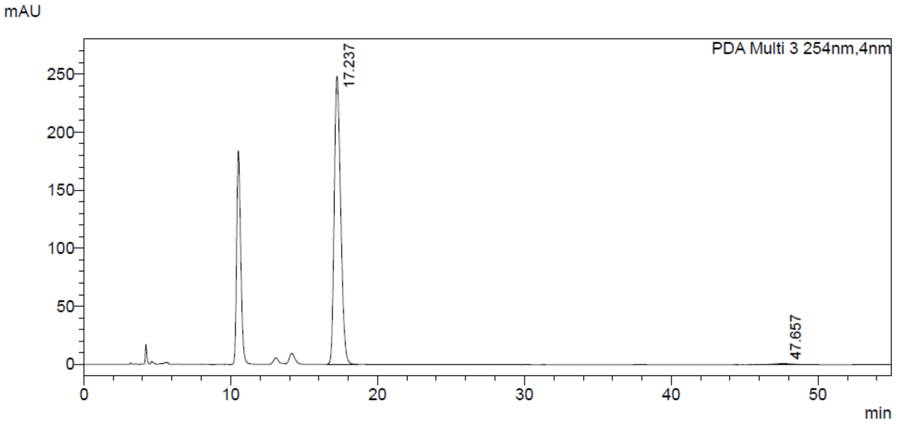

<Peak Table>

PDA Ch3 254nm

Peak#	Ret. Time	Area%
1	17.237	99.054
2	47.657	0.946
Total		100.000

minor diastereoisomer: t_R (major): 10.5 min, t_R (minor): 13.1 min,97:3 er.

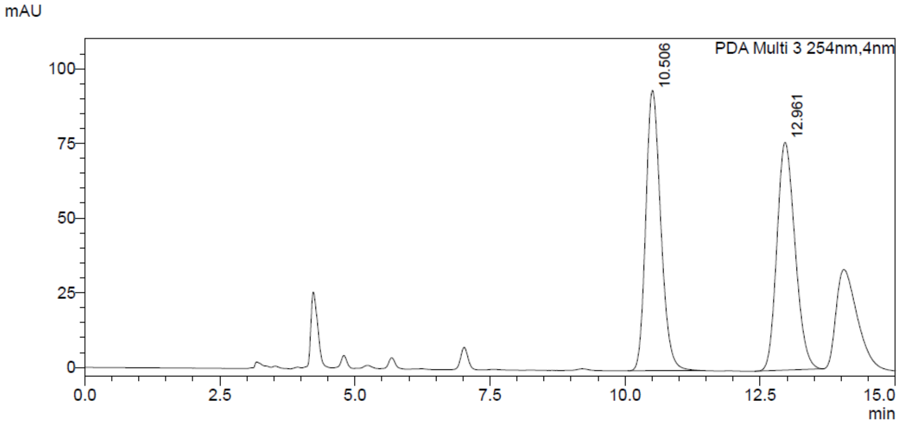

<Peak Table>

PDA Ch3 254nm

Peak#	Ret. Time	Area%
1	10.506	50.356
2	12.961	49.644
Total		100.000

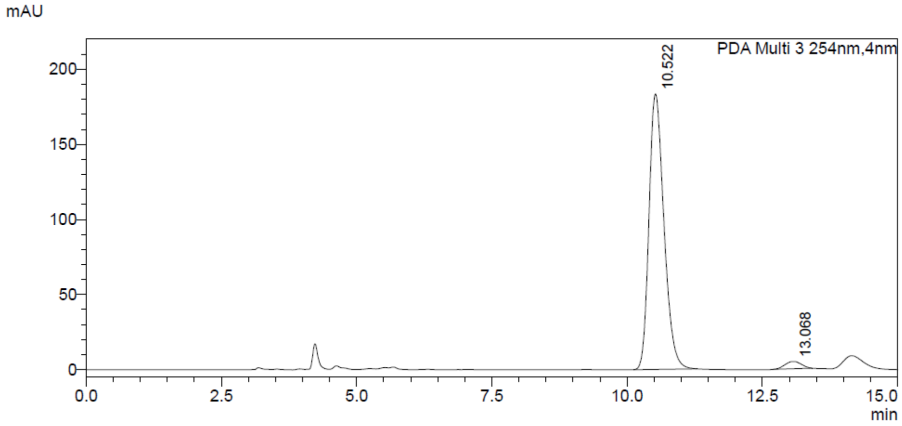

<Peak Table>

PDA Ch3 254nm

Peak#	Ret. Time	Area%
1	10.522	97.189
2	13.068	2.811
Total		100.000

HPLC Data for **43**: **Chiral HPLC analysis**, Chiralcel OD-H (95:5 hexane:IPA, flow rate 1.0 mLmin^{-1}, 254 nm, 30 °C), t_R (minor): 46.9 min, t_R (major): 51.7 min, >99:1 er.

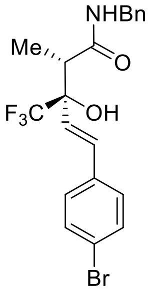

43

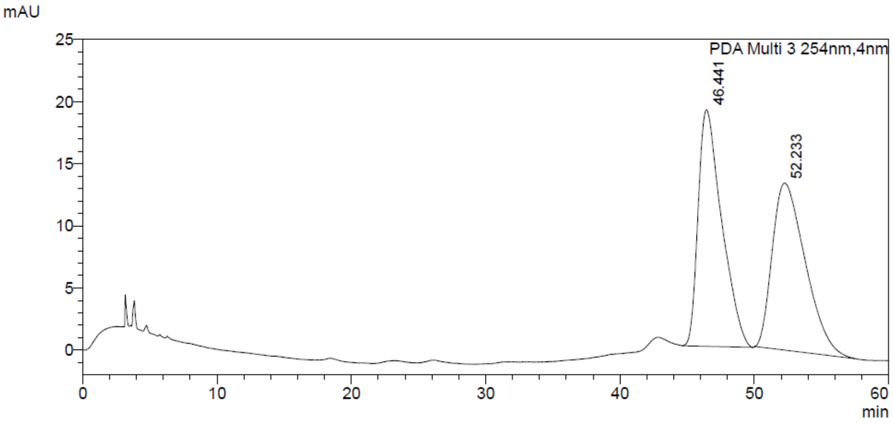

<Peak Table>

PDA Ch3 254nm

Peak#	Ret. Time	Area%
1	46.441	50.221
2	52.233	49.779
Total		100.000

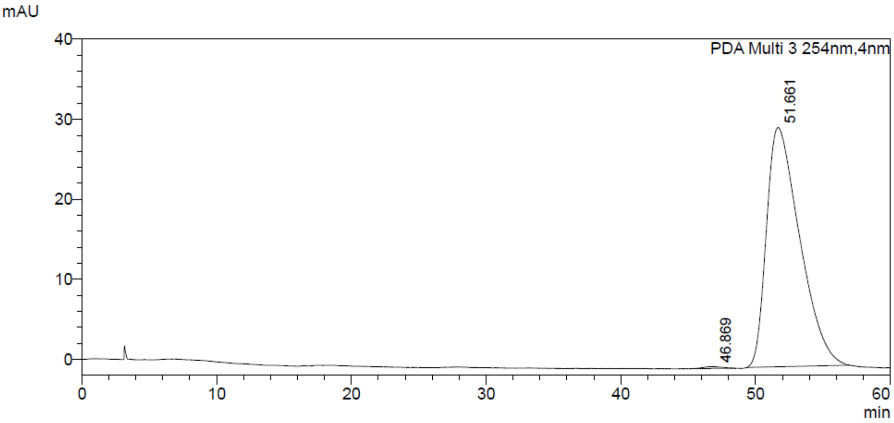

<Peak Table>

PDA Ch3 254nm

Peak#	Ret. Time	Area%
1	46.869	0.408
2	51.661	99.592
Total		100.000

HPLC Data for **44**: **Chiral HPLC analysis**, Chiralpak AS-H (98:2 hexane:IPA, flow rate 1.0 mLmin^{-1}, 254 nm, 30 °C), t_R (minor): 14.1 min, t_R (major): 17.0 min, 95:5 er.

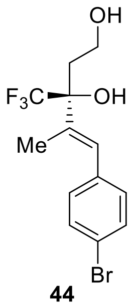

44

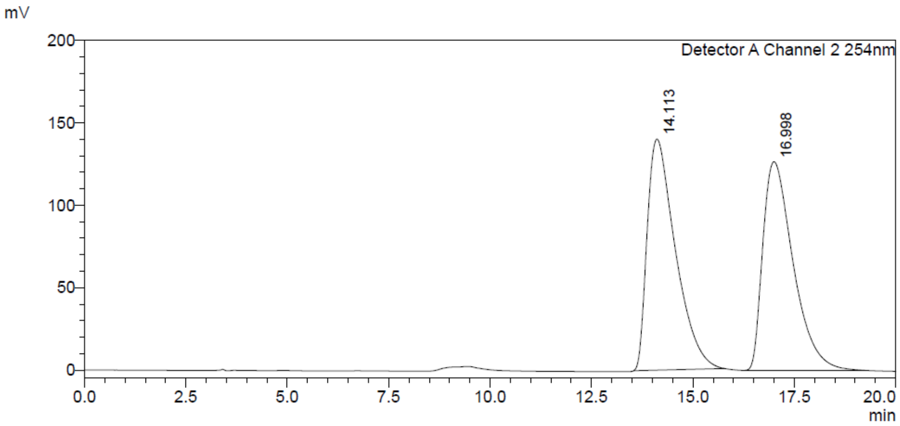

<Peak Table>

Detector A Channel 2 254nm

Peak#	Ret. Time	Area%
1	14.113	50.653
2	16.998	49.347
Total		100.000

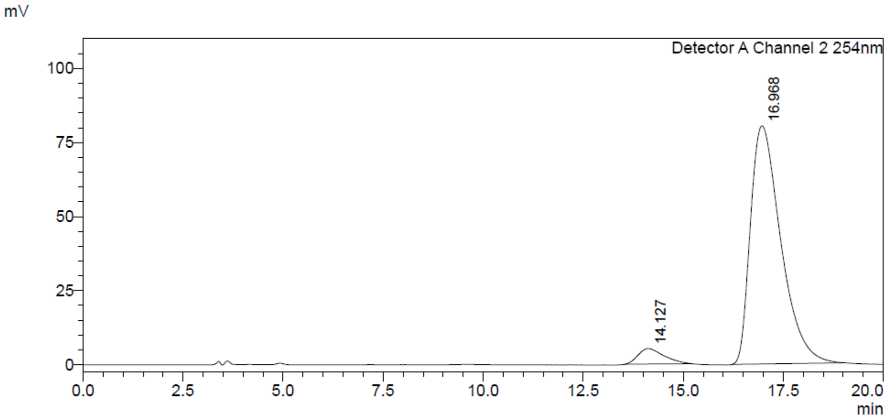

<Peak Table>

Detector A Channel 2 254nm

Peak#	Ret. Time	Area%
1	14.127	5.193
2	16.968	94.807
Total		100.000

HPLC Data for **45**: **Chiral HPLC analysis**, Chiralpak AD-H (99:0.2 hexane:IPA, flow rate 1.0 mLmin^{-1}, 254 nm, 30 °C), t_R (minor): 5.7 min, t_R (major): 6.0 min, 96:4 er.

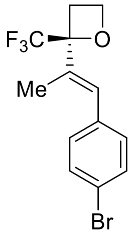

45

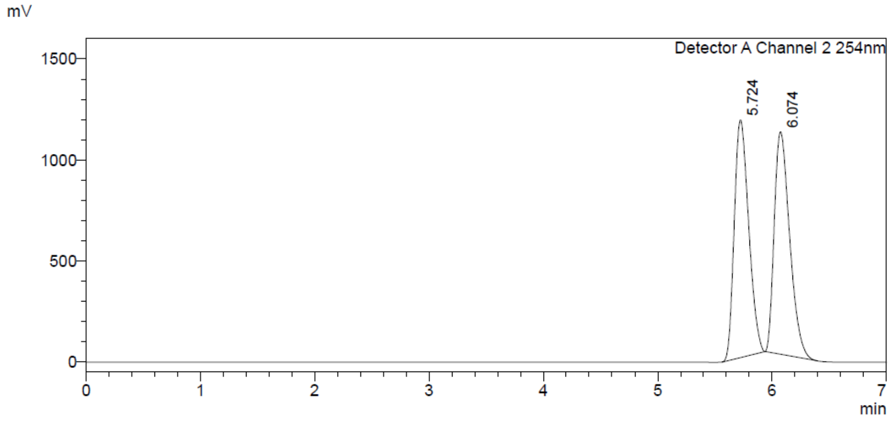

<Peak Table>

Detector A Channel 2 254nm

Peak#	Ret. Time	Area%
1	5.724	49.960
2	6.074	50.040
Total		100.000

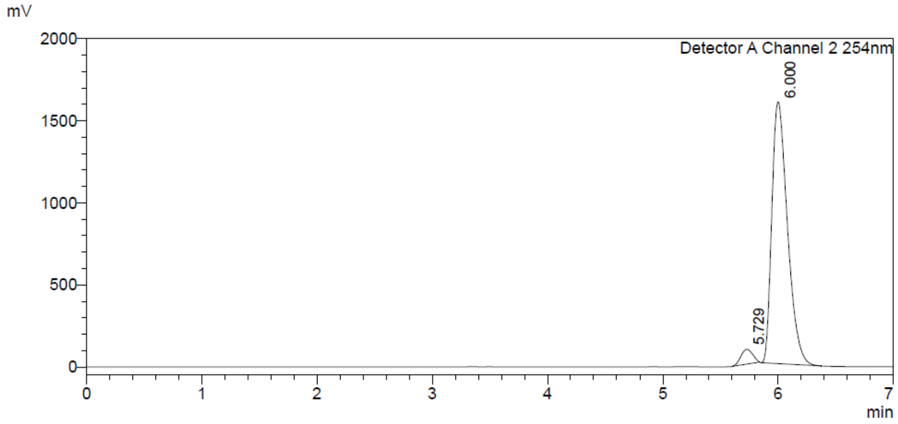

<Peak Table>

Detector A Channel 2 254nm

Peak#	Ret. Time	Area%
1	5.729	4.051
2	6.000	95.949
Total		100.000

HPLC Data for **54**: Chiralpak AD-H (95:5 hexane:IPA, flow rate 1.0 mLmin⁻¹, 211 nm, 30 °C), **major diastereoisomer**: t_R (major): 8.8 min, t_R (minor): 9.6 min, 95:5 er.

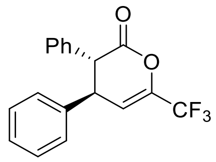

54

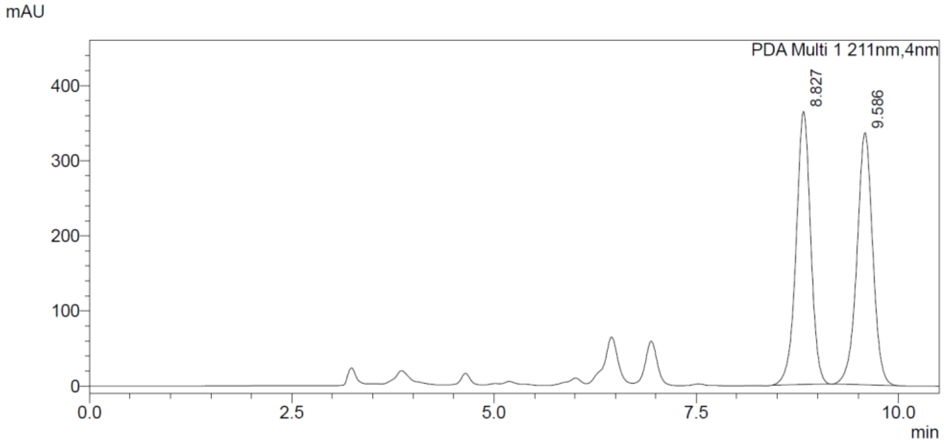

<Peak Table>

PDA Ch1 211nm

Peak#	Ret. Time	Area%
1	8.827	49.927
2	9.586	50.073
Total		100.000

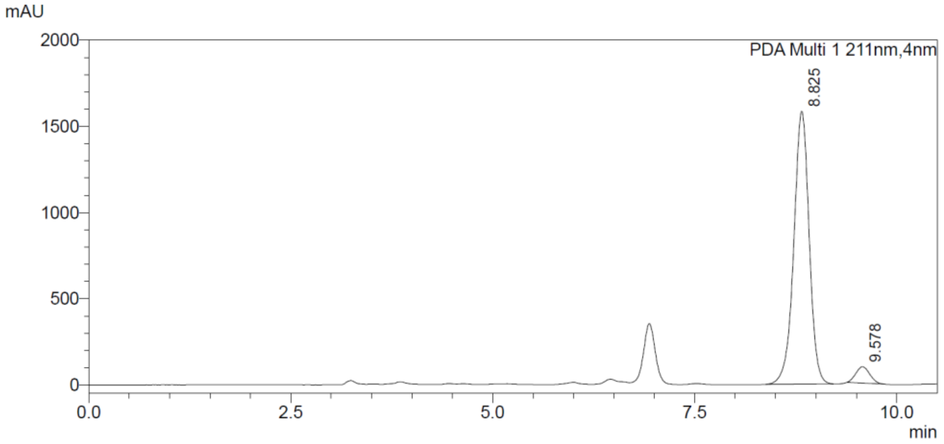

<Peak Table>

PDA Ch1 211nm

Peak#	Ret. Time	Area%
1	8.825	94.884
2	9.578	5.116
Total		100.000

Minor diastereoisomer: t_R (minor): 6.5 min, t_R (major): 6.9 min, 93:7 er.

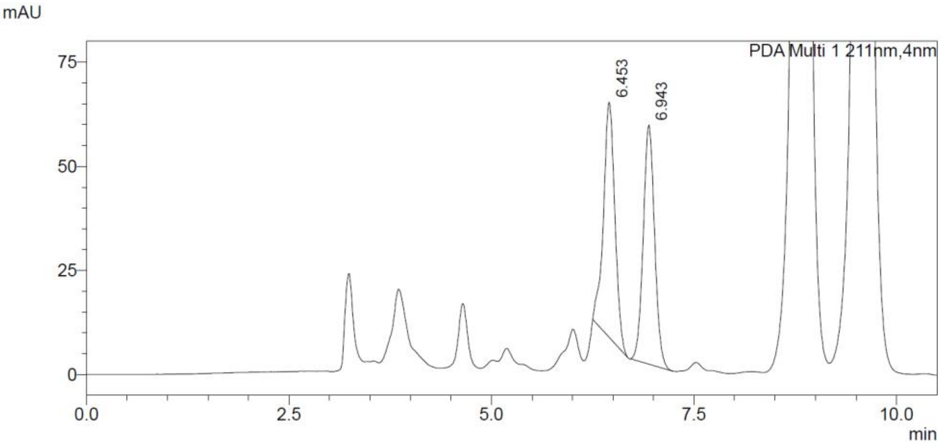

<Peak Table>

PDA Ch1 211nm

Peak#	Ret. Time	Area%
1	6.453	49.568
2	6.943	50.432
Total		100.000

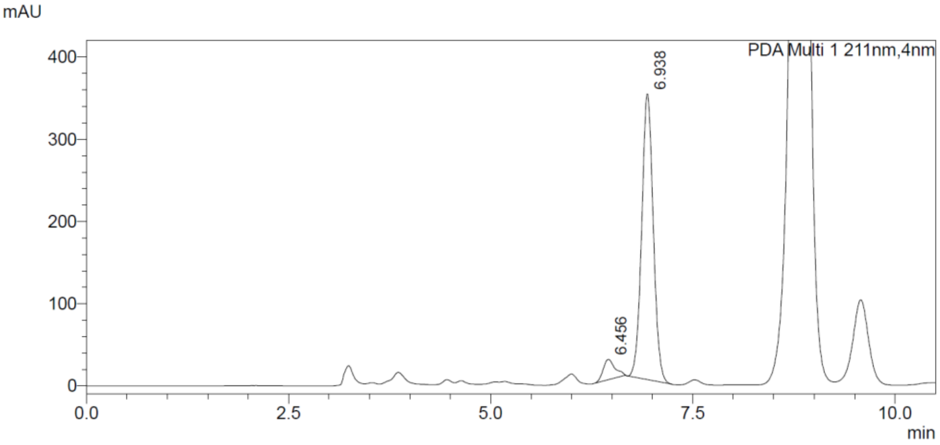

<Peak Table>

PDA Ch1 211nm

Peak#	Ret. Time	Area%
1	6.456	6.751
2	6.938	93.249
Total		100.000
